# Catalytic asymmetric acetalization of carboxylic acids for access to chiral phthalidyl ester prodrugs

**DOI:** 10.1038/s41467-019-09445-x

**Published:** 2019-04-11

**Authors:** Yingguo Liu, Qiao Chen, Chengli Mou, Lutai Pan, Xiaoyong Duan, Xingkuan Chen, Hongzhong Chen, Yanli Zhao, Yunpeng Lu, Zhichao Jin, Yonggui Robin Chi

**Affiliations:** 1 Division of Chemistry & Biological Chemistry, School of Physical & Mathematical Sciences, Nanyang Technological University, Singapore, 637371 Singapore; 2 School of Pharmacy, Guiyang University of Chinese Medicine, Huaxi District, Guiyang, 550025 China; 3 Laboratory Breeding Base of Green Pesticide and Agricultural Bioengineering, Key Laboratory of Green Pesticide and Agricultural Bioengineering, Ministry of Education, Guizhou University, Huaxi District, Guiyang, 550025 China

## Abstract

Carboxylic acids are common moieties in medicines. They can be converted to phthalidyl esters as prodrugs. Unfortunately, phthalidyl esters are now mostly prepared in racemic forms. This is not desirable because the two enantiomers of phthalidyl esters likely have different pharmacological effects. Here we address the synthetic challenges in enantioselective modification of carboxylic acids via asymmetric acetalizations. The key reaction step involves asymmetric addition of a carboxylic acid to the catalyst-bound intermediate. This addition step enantioselectively constructs a chiral acetal unit that lead to optically enriched phthalidyl esters. A broad range of carboxylic acids react effectively under mild and transition metal-free conditions. Preliminary bioactivity studies show that the two enantiomers of chlorambucil phthalidyl esters exhibit different anti-cancer activities to inhibit the growth of Hela cells. Our catalytic strategy of asymmetric acetalizations of carboxylic acids shall benefit future development of chiral phthalidyl ester prodrugs and related molecules.

## Abstract (web summary)

Phthalidyl esters, commonly used prodrug moieties, are mostly prepared as a racemate. Here, the authors report an N-heterocylcic carbene-catalysed enantioselective acetalization of carboxylic acids and dialdehydes to give phthalidyl esters in high yields with good enantioselectivity.

## Introduction

**Fig. 1:**
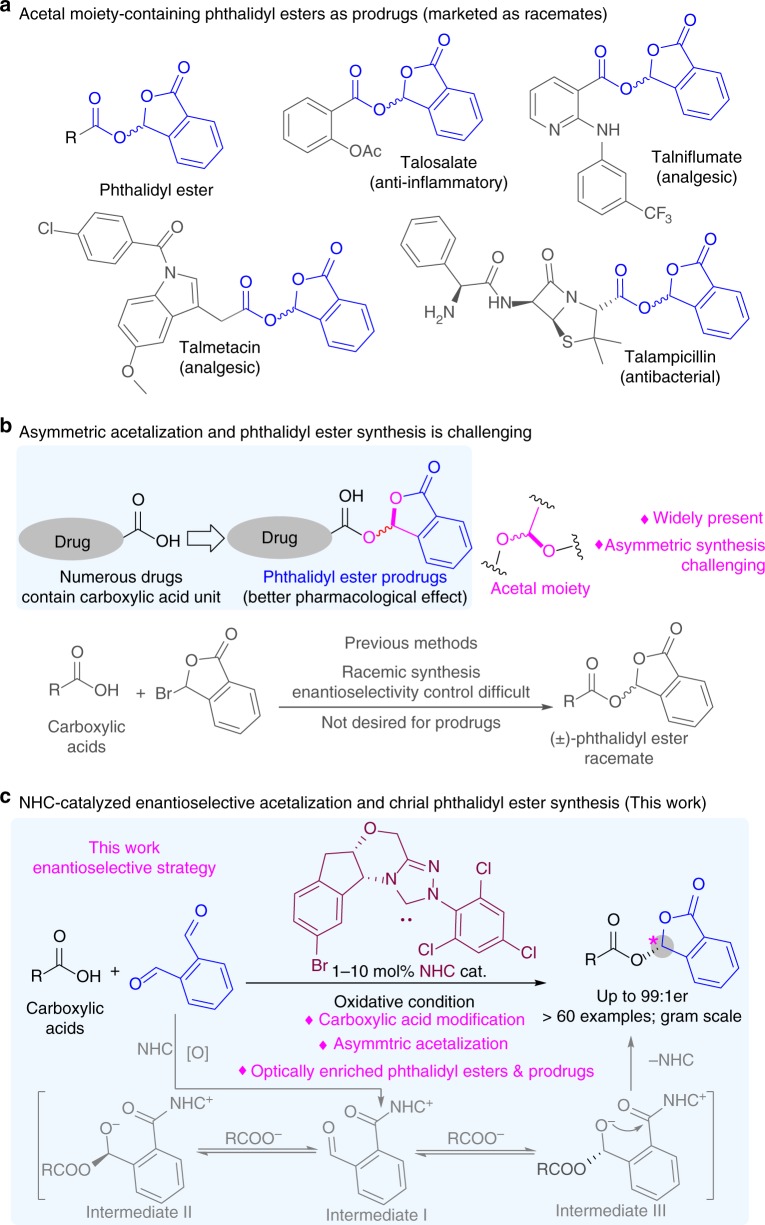
Phthalidyl ester prodrugs and synthetic methods. **a** Acetal moiety-containing phthalidyl esters as prodrugs (marketed as racemates) **b** Asymmetric acetalization and phthalidyl ester synthesis is challenging. **c** NHC-catalyzed enantioselective acetalization and chrial phtalidyl ester synthesis (this work)

Chemical modification on medicinally significant natural products or drug molecules^1–5^ is a proven strategy to develop chemical entities and prodrugs for improving drug performance or introducing alternative clinical applications^6–8^. Approximately one out of five chemical entity approvals by FDA are prodrugs in recent three years^9,10^. Carboxylic acids are among the most common moieties in pharmaceuticals. They are typically converted to the corresponding esters for better drug efficacy and/or lower side effects^11^. Among the different types of esters, phthalidyl esters as promoieties were found with impressive success^12^ (Fig. 1a). Representative examples of phthalidyl ester prodrugs include talosalate, talniflumate, talampicilin, and talmetacin^13–15^. These phthalidyl esters contain an acetal moiety with a stereogenic carbon center that is difficult to be installed enantioselectively (Fig. 1b). To date, phthalidyl esters are routinely prepared via reactions of carboxylic acids with 3-bromophthalides (Fig. 1b)^16^. This method is efficient but unfortunately has no controls over the stereoselectivity for the newly created chiral center. It is challenging to directly modify carboxylic acid and related heteroatom functional groups in an enantioselective manner to form chiral acetal moieties and their analogs^17–19^. Thus, the phthalidyl esters are afforded as a racemic mixture of two enantiomers. Related efforts for enantioselective synthesis of phthalidyl esters also remain unsuccessful. The limited examples via kinetic resolutions used carboxylic anhydrides as the substrates and gave poor to moderate enantioselectivities with narrow substrate scopes^20^.

Recent studies have further shown that such prodrugs exhibit medicinal applications. For example, talniflumate, an anti-inflammatory phthalidyl ester drug sold on the market for over thirty years, has extended its use in treatment of rheumatoid arthritis to cystic fibrosis, chronic obstructive pulmonary disease (COPD) and asthma, and is now identified as a novel inhibitor that improves responsiveness of pancreatic tumors to gefitinib^21–24^. In these cases, phthalidyl esters were used in racemic form while FDA guidelines and policies have required that each enantiomer shall be meticulously studied in pharmacology and toxicology before reaching the market^25,26^. It’s worthy to note that chiral phthalidyl esters are also found in bioactive natural products isolated from the marine plants, such as *luteorosin* and *macfarlandin A*^27^. Unfortunately, efficient methods for asymmetric access to optically enriched phthalidyl esters are not available.

Here we disclose a highly enantioselective organic catalytic strategy for carboxylic acid functionalization and efficient access to optically enriched phthalidyl esters (Fig. 1c). Our approach involves an N-heterocylcic carbene (NHC)-catalyzed activation of a phthalaldehyde that subsequently reacts with a carboxylic acid to form phthalidyl ester products. The main pathway involves a dynamic kinetic resolution process (Fig. 1c). Oxidation of the one aldehyde moiety-derived Breslow intermediate generates azolium ester intermediate I. An addition of carboxylic acid to another aldehyde moiety of chiral NHC-bound intermediate I affords diastereomeric intermediate II and III. Intermediate III preferentially undergoes the intermolecular annulation to give chiral phthalidyl esters with high enantiomeric ratio. The reaction enantioselectivity is controlled by the NHC catalyst. In our approach the operational condition is mild, and no transition metals are involved. A broad range of substrates and functional groups are well tolerated. Both aryl and aliphatic carboxylic acids bearing various substituents work effectively. Several natural products and drug molecules (e.g., valproic acid, chlorambucil, and naproxen) bearing carboxylic acid moieties can be enantioselectively modified using this approach. We also performed preliminary evaluations on the bioactivities of the phthalidyl ester derivatives of chlorambucil, an anti-cancer agent and chemotherapy drug. Our results show that the (*R*)-enantiomer of the phthalidyl ester derivative have better activities to inhibit the growth of Hela cells than the corresponding (*S*)-enantiomer, racemic mixture and the unmodified chlorambucil. With our catalytic approach we achieve enantioselective addition of carboxylic acid to catalytically generated NHC-bound intermediates. Given the wide presence of carboxylic acid moieties in bioactive molecules, we expect our enantioselective approach for phthalidyl ester synthesis to bring significant values for both discovery and manufacturing of better pharmaceuticals.

## Results

### Condition optimization

We chose aspirin (acetylsalicylic acid, **1**) as a model carboxylic acid to react with *o*-phthalaldehyde (**2**) in searching for suitable NHC catalysts and conditions (Table 1). Both aspirin and the corresponding phthalidyl ester (talosalate) are commercial drugs for several decades. Key results from extensive optimizations (see Supplementary Table 1, 2 and 3) are shown in Table 1. A typical reaction was performed with azolium salt as an NHC pre-catalyst, quinone as an oxidant^28^, and K_2_CO_3_ as the base. When N-mesityl substituted triazolium salt A^29^ was used as the NHC pre-catalyst, the reaction carried out in CH_2_Cl_2_ at room temperature with LiCl as an additive gave desired phthalidyl ester product **3** in 66% yield and encouraging 60:40 er value (entry 1). Replacing the NHC pre-catalyst **A** with N-trichlorophenyl substituted triazolium **B**^30^ led to product **3** with improvements on both yield and er value (84% yield, 77:23 er; entry 2). Encouraged by this improvement, we modified the pre-catalyst B to obtain a pre-catalyst **C**, the congener earlier reported by Yamada^31^. Changing the pre-catalyst to **C** resulted in a further increase on er value (85:15, entry 3). Additional optimizations were then performed with **C** as a pre-catalyst. Replacing CH_2_Cl_2_ with CHCl_3_ as the solvent improved the er of **3** to 90:10 (entry 4). Decreasing the reaction temperature to −20 °C led to product **3** with 89% yield and 95:5 er (entry 5). The presence of NHC pre-catalyst was essential for this reaction (entry 6). The use of LiCl as an additive^32–35^ offered a small while consistent increase on product er value (entry 7). The quinone oxidant could be replaced by MnO_2_ with reaction yield and er values remained (entry 8).

**Table 1:** Condition optimization


Entry	condition	Isolated yield(%)	e.r.
1	A, CH_2_Cl_2_, RT	66	60:40
2	B, CH_2_Cl_2_, RT	84	77:23
3	C, CH_2_Cl_2_, RT	92	85:15
4	C, CHCl_3_, RT	91	90:10
5	C, CHCl_3_, −20 °C	89	95:5
6	Without NHC	n.r.	—
7	As entry 5, w/o LiCl additive	85	93:7
8	As entry 5, except MnO_2_ as oxidant	83	94:6

Reaction conditions: **1**(0.1 mmol), **2** (0.15 mmol), solvent (2 mL), rt, 12 h. Yields determined by isolation*er* determined by HPL, *n.r.* no reaction

### Substrate scope

**Fig. 2:**
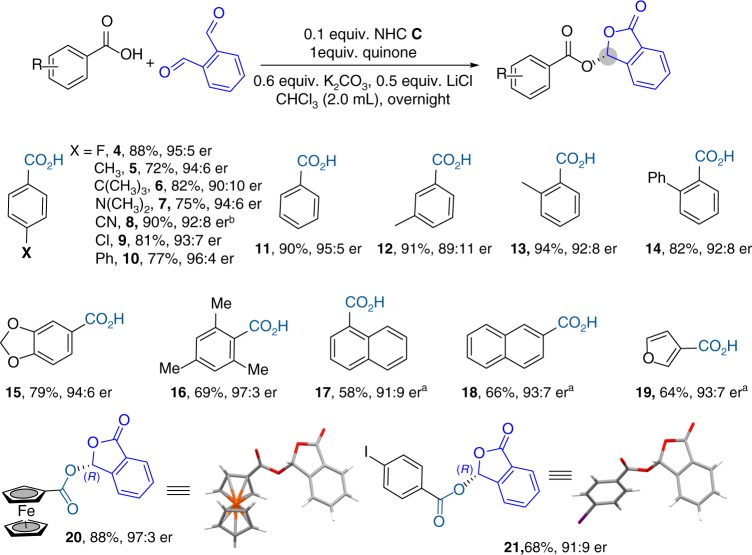
Scope of aromatic acids. Reaction conditions: aromatic acid (0.1 mmol), *o*-phthalaldehyde (0.15 mmol), 0.1 equiv. NHC **C**, 1 equiv. quinone, 0.6 equiv. K_2_CO_3_, 0.5 equiv. LiCl, CHCl_3_ (2 mL), rt, 12 h. Yields determined by isolation. er determined by HPLC. ^a^under −30 °C. ^b^toluene as solvent

With an optimized set of conditions in hand, we evaluated the generality of our reactions (Fig. 2). We first studied aryl carboxylic acids (**4**–**21**). Various substituents (such as halogen and amine moieties) or substitution patterns for benzoic acid were all well tolerated (**4**–**14**). Multiple substituents can be present on the benzoic acid (**15**, **16**). The use of sterically bulky aryl carboxylic acid could typically give higher product er values (e.g., **16**; 97:3 er). Hetero aryl carboxylic acids worked effectively (**19**). The absolute configurations of our catalytic reaction products were confirmed based on X-ray structures of ferrocenecarboxylic acid (**20**) and para-iodobenzoic acid (**21**).

**Fig. 3:**
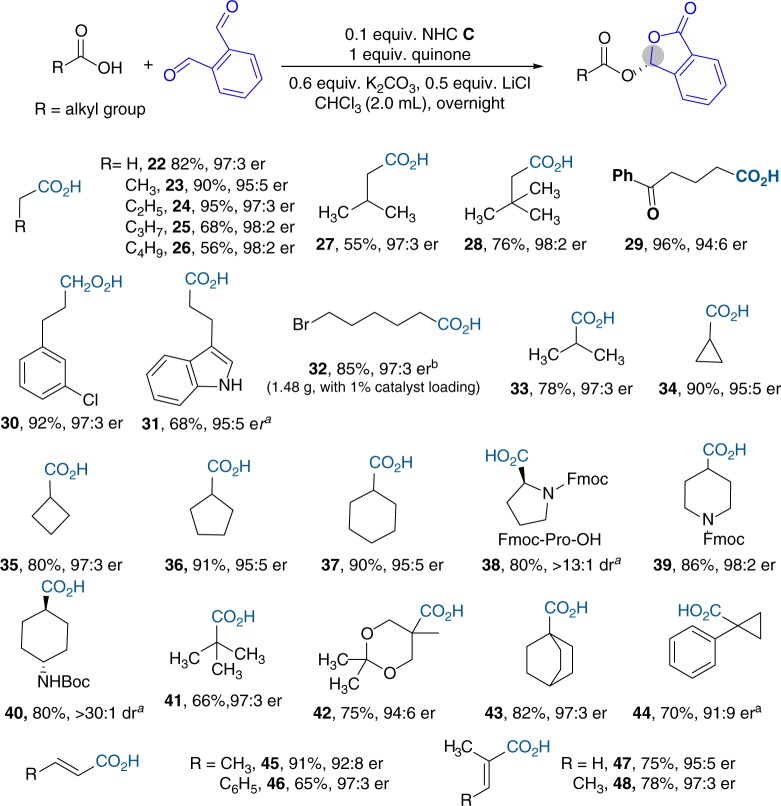
Scope of alkyl acids. Reaction conditions: alkyl acid (0.1 mmol), *o*-phthalaldehyde (0.15 mmol), 0.1 equiv. NHC **C**, 1 equiv. quinone, 0.6 equiv. K_2_CO_3_, 0.5 equiv. LiCl, CHCl_3_ (2 mL), rt, 12 h. Yields determined by isolation. er determined by HPLC, ^a^under −30 °C. ^b^under −10 °C

We next evaluated aliphatic acids (**22**–**44**) (Fig. 3). Notably, aliphatic acids are widely present in small molecule pharmaceuticals, peptides and proteins. They are increasingly recognized as signaling molecules and hold significant therapeutic potentials^36^. We were very delighted to find that higher enantioselectivities were obtained with aliphatic acids, when compared with aryl carboxylic acids. For example, when acetic acid is the substrate (**22**), the corresponding phthalidyl ester was obtained with 82% yield and 97:3 er. The length of the alkyl chain in the acids has little influence on the reaction yield and er values (**22**–**26**). Primary (**22**–**32**), secondary (**33**–**40**), and tertiary (**41**–**44**) alkyl carboxylic acids all worked well to give the corresponding products with excellent enantioselectivities. For cyclic (**34**–**37**, **40**, and **44**), heterocyclic (**38**, **39**, **42**), and bridging (**43**) alkyl acids, the ring size and steric hindrance have little influence on the reaction outcomes. Unsaturated carboxylic acids are effective substrates as well (**45**–**48**). Our catalytic reactions are amenable for scalable synthesis with low catalyst loadings. We demonstrated that gram scale of acid **32** could be effectively transformed to the corresponding ester with 97:3 er by using 1 mol% of NHC catalyst. However, when we tried stronger acids, such as trifluoracetic acid, the reaction became messy and no desired product was obtained (see Supplementary Fig. 187). The main reasons likely include lower nucleophilicity of the trifluoracetic anion, and the poor stabilities of the intermediates resulted from reactions between trifluoracetic anion and the dialdehyde substrates.

**Fig. 4:**
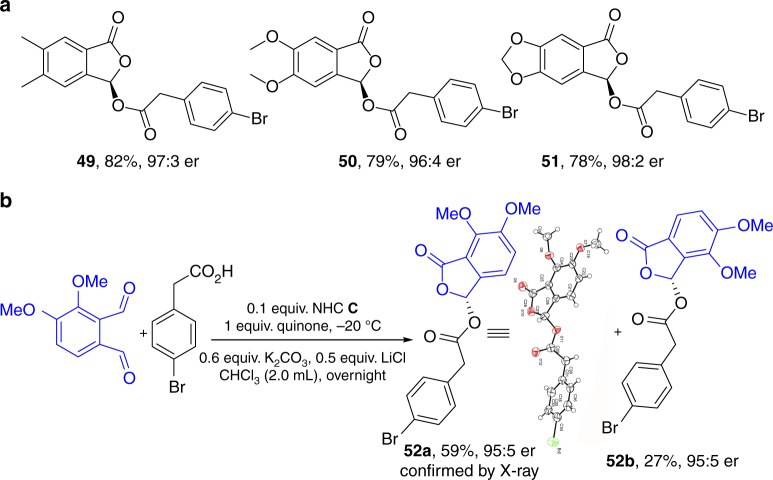
Scope of dialdehydes and regioselectivity study. **a** substituted aldehydes. **b** unsymmetic dialdehyde (regioselectivity study)

Substituted phthaldehydes tested here all reacted effectively to give the corresponding ester products with excellent er values and good yields (**49**–**52**). Regioselectivity studies of unsymmetrical dialdehydes were investigated (Fig. 4b **52a**:**52b** = 2.2:1, 95:5 er), The addition of carboxylate to aldehyde favors the sterically less congested site of the dialdehydes. For the reaction between aldehyde and NHC, (to eventually form azolium ester intermediate under oxidative condition), the carbene addition step is not the rate-determine step and thus the substituents have little influence. These results (Figs. 2, 3, and 4) clearly illustrate the broad applicability of our strategy for enantioselective modification of various carboxylic acids.

**Fig. 5:**
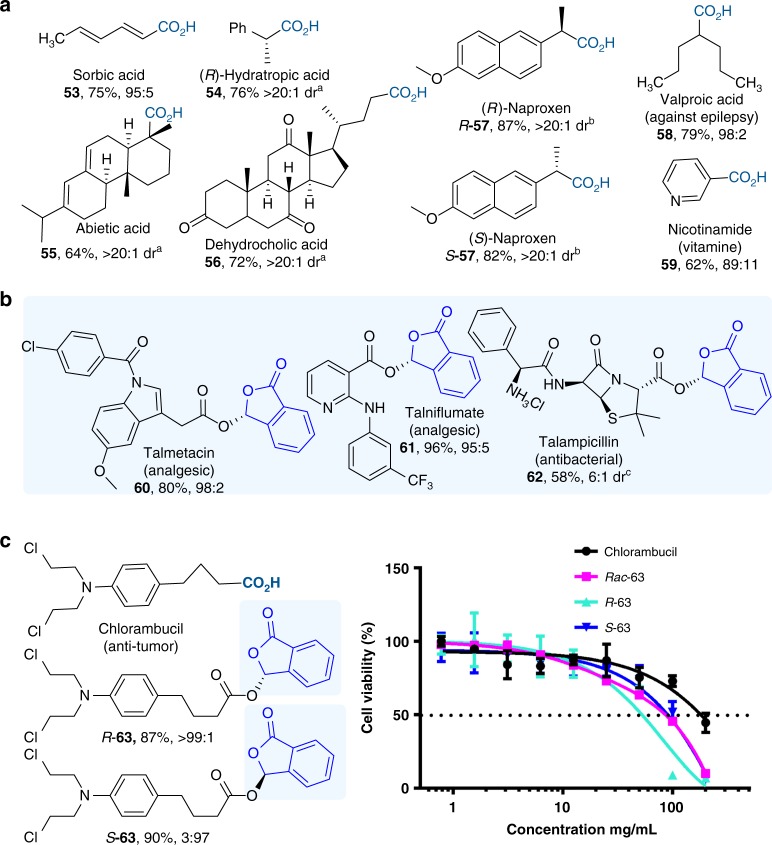
Natural products and medicinal molecules. **a** Natural products modified using our approach. **b** Marketed prodrugs synthesized via our approach. Reaction condition: acid (0.1 mmol), *o*-phthalaldehyde (0.15 mmol), 0.1 equiv. NHC **C**, 1 equiv. quinone, 0.6 equiv. K_2_CO_3_, 0.5 equiv. LiCl, CHCl_3_ (2 mL), rt, 12 h. ^a^dr determined by ^1^H NMR; ^b^dr determined by HPLC. ^c^with acetone as solvent, yield for 2 steps;. **c** Bioevaluation of chiral phthalidyl ester prodrugs against the growth of Hela cells (*n* = 3 biological replicates, Mean ± SD), Rac-**63**(*R*-**63**:*S*-**63** = 1:1), IC_50_ for Chlorambucil: 157.0 mg/mL; Rac-**63**: 83.6 mg/mL; *R*-**63**: 53.6 mg/mL, *S*-**63**: 91.7 mg/mL. Source data are provided as a Source Data file

To further demonstrate the generality and utility of our strategy, several natural products bearing carboxylic acid moieties (Fig. 5) were modified under our standard catalytic condition (**53**–**55**). Multiple commercially used drug molecules bearing carboxylic acid moieties were also modified to give the corresponding ester products (**56**–**59**, **63**). In all cases, the reaction worked effectively. The newly created chiral center during phthalidyl ester formation is well-controlled by the NHC catalyst. The chiral centers present in the natural products and drugs have nearly no influence over the stereochemistry during the catalytic reaction. For example, the use of drug *R*-**57** and *S*-**57** under otherwise identical catalytic conditions both give the corresponding ester products with exceptional diastereoselectivities and identical chirality for the newly formed center. Relatively sophisticated natural products and drugs (abietic acid **55**, dehydrocholic acid **56**) with multiple fused rings and chiral centers are well tolerated in our condition. In addition to talosalate (**3**), we have also demonstrated that our methods can be used to prepare optically enriched versions of several phthalidyl ester prodrugs sold on the market, including talmetacin, talniflumate, and talampicillin (**60**–**62**) (Fig. 5b).

### Bioevaluation

It is well established that the two enantiomers of a molecule can have different pharmacological effects^37^. Chlorambucil is an anti-cancer drug with applications mainly in chronic lymphocytic leukemia (CLL), Hodgkin’s lymphoma (HL), and non-Hodgkin lymphoma (NHL)^38,39^. We performed preliminary bioactivity studies using the two phthalidyl ester enantiomers of Chlorambucil (Fig. 5c). Our results show that at the concentration of 100 μg/mL (See Supplementary Fig. 188). The (*R*)-enantiomer (*R*-**63**, >99:1 er, IC_50_: 53.6 mg/mL) phthalidyl ester is more effective to inhibit Hela cells than the corresponding (*S*)-enantiomer (*S*-**63**, 3:97 er, IC_50_: 83.6 mg/mL), racemate (Rac-63, IC_50_: 91.7 mg/mL) and unmodified chlorambucil (IC_50_: 157.0 mg/mL). The different pharmacological effects might be attributed to better penetrations through cell membranes or different interactions with the target DNA brought by the chiral modifications.

## Discussion

In summary, we have addressed the challenges in enantioselective acetalization of carboxylic acids for quick access to optically enriched phthalidyl esters. A wide range of carboxylic acids, including natural products and pharmaceuticals, reacted effectively, and stereo-selectively under our conditions. Carboxylic acids are among the most common functional groups in bioactive molecules and medicines. Phthalidyl esters are proven prodrugs of carboxylic acids. We expect our method to bring significant values for the discovery and development of better chiral prodrugs in enantiomerically enriched forms. Our study shall also benefit future development on enantioselective acetalization and related reactions for asymmetric functionalization of heteroatoms.

## Methods

### General approach to chiral phthalidyl esters

To a 10 ml-round bottomed flask were added the acid (0.1 mmol), phthaldehyde (0.15 mmol), NHC C (0.01 mmol), 3,3′,5,5′-Tetra-tert-butyldiphenoquinone (0.1 mmol, or 5eq MnO_2_) and LiCl (0.05 mmol), then 2 ml of CHCl_3_ (unless otherwise noted) was added at − 20 °C (unless otherwise noted) followed by addition of K_2_CO_3_ powder (0.06 mmol). The reaction was stirred overnight or until the red brown color faded into light yellow or colorless. The solvent was removed under vacuum and the resulting residue was applied on the column chromatography (eluent, hexane: ethyl acetate = 3:1) to give the corresponding phthalidyl esters.

Compound *R*-**63** was also subject to the general procedure with some minor change on reaction conditions. The substrate ampicillin was protected with benzaldehyde in presence of TMEDA following the reported reference^13,40^. Then the obtained D-α-benzylideneaminobenzylpenicillin salt was applied in the general procedure with acetone as solvent (eluent, hexane: ethyl acetate = 1:1). After that, the enamine-protecting group was removed from this product by dissolving it in aqueous acetone (1:1, 2 mL) and vigorously stirring this solution at pH 2.5 for 30 min (1 drop of 1 *N* HCl). Acetone was removed in vacuo and the product *R*-**63** was salted out of the aqueous phase as a sticky gum. The material was dissolved in ethyl acetate, washed with H_2_O, and dried. Careful addition of dry ether to the ethyl acetate solution of the penicillin ester afforded compound *R*-**63** as an off-white amorphous solid.

### Bioevaluation

MTT (3-(4,5-dimethylthiazol-2-yl)-2,5-diphenyltetrazolium bromide) assay was used to investigate the cytotoxicity of prodrugs including *R*-enantiomer (*R*-**63**), *S*-enantiomer (*S*-**63**) racemate (Rac-**63**) and free drug chlorambucil. The HeLa cells (obtained from ATCC, Rockville, MD, maintained in Dulbecco’s Modified Eagle’s Medium) were seeded in 96-well plates (100 μL of medium) and incubated for 24 h. After the cell density reached 60–70%, the cells were fed with the prodrugs and chlorambucil at the concenration of 0, 0.78, 1.56, 3.13, 6.25, 12.5, 25, 50 100, 200 mg/mL, and incubated with 48 h. After the medium removed, the fresh medium with 10% MTT was added, and incubated with for another 4 h. The medium was removed carefully, followed by adding 100 μL of DMSO. Finally, optical densities of the samples were measured using a microplate reader with the double wavelength of 570 nm and 490 nm.

### Reporting Summary

Further information on experimental design is available in the Nature Research Reporting Summary linked to this article.

## Supplementary information

Supplementary Information

Reporting Summary

## Data Availability

The X-ray crystallographic coordinates for NHC **C**, compound **20**, **21** and **52a** reported in this study have been deposited at the Cambridge Crystallographic Data Centre (CCDC), under CCDC 1866589, 1866428, 1866429, and 1893685. These data can be obtained free of charge from The Cambridge Crystallographic Data Centre via www.ccdc.cam.ac.uk/data_request/cif. The raw data underlying Fig. 5c, Supplementary Fig. 188 and Supplementary Fig. 189 are provided as a Source Data file. Full experimental details for new compounds, and their spectroscopic, chromatographic data, and bioevaluation data, can be found in the supplementary information.
