# Supplementary material for: Catalytic asymmetric acetalization of carboxylic acids for access to chiral phthalidyl ester prodrugs
Source: Nat Commun. 2019 Apr 11;10:1675. doi: 10.1038/s41467-019-09445-x (PMC6459872; doi:10.1038/s41467-019-09445-x)
Supplement: Supplementary file 1 — Supplementary Information [file 41467_2019_9445_MOESM1_ESM.pdf]

Supplementary Information

Catalytic Asymmetric Acetalization of Carboxylic Acids for Access to Chiral Phthalidyl Ester Prodrugs

Liu et al

Supplementary Figures

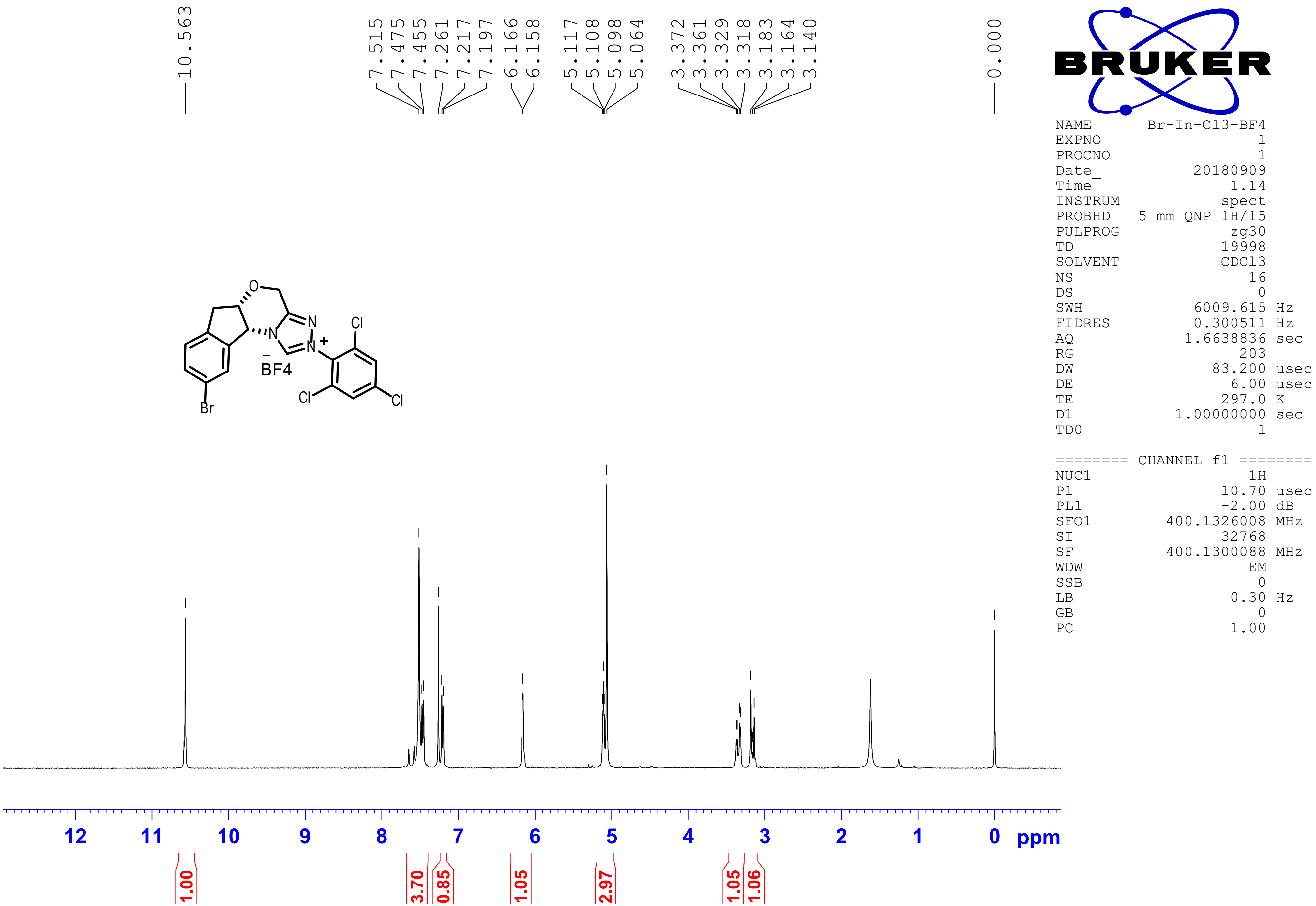

Supplementary Figure 1 . H NMR spectrum of NHC C

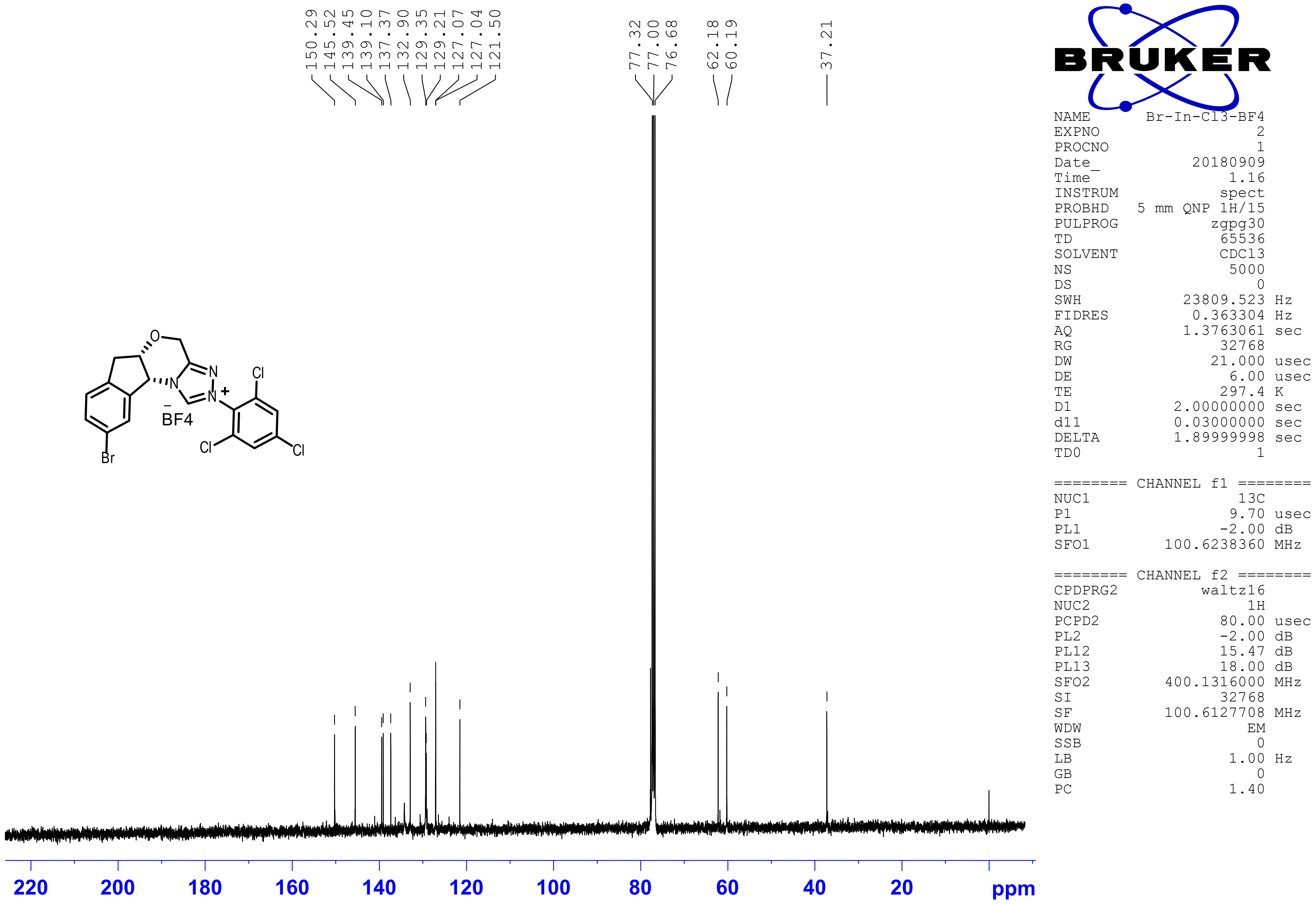

Supplementary Figure 2 ^{13}C NMR spectrum of NHC **C**

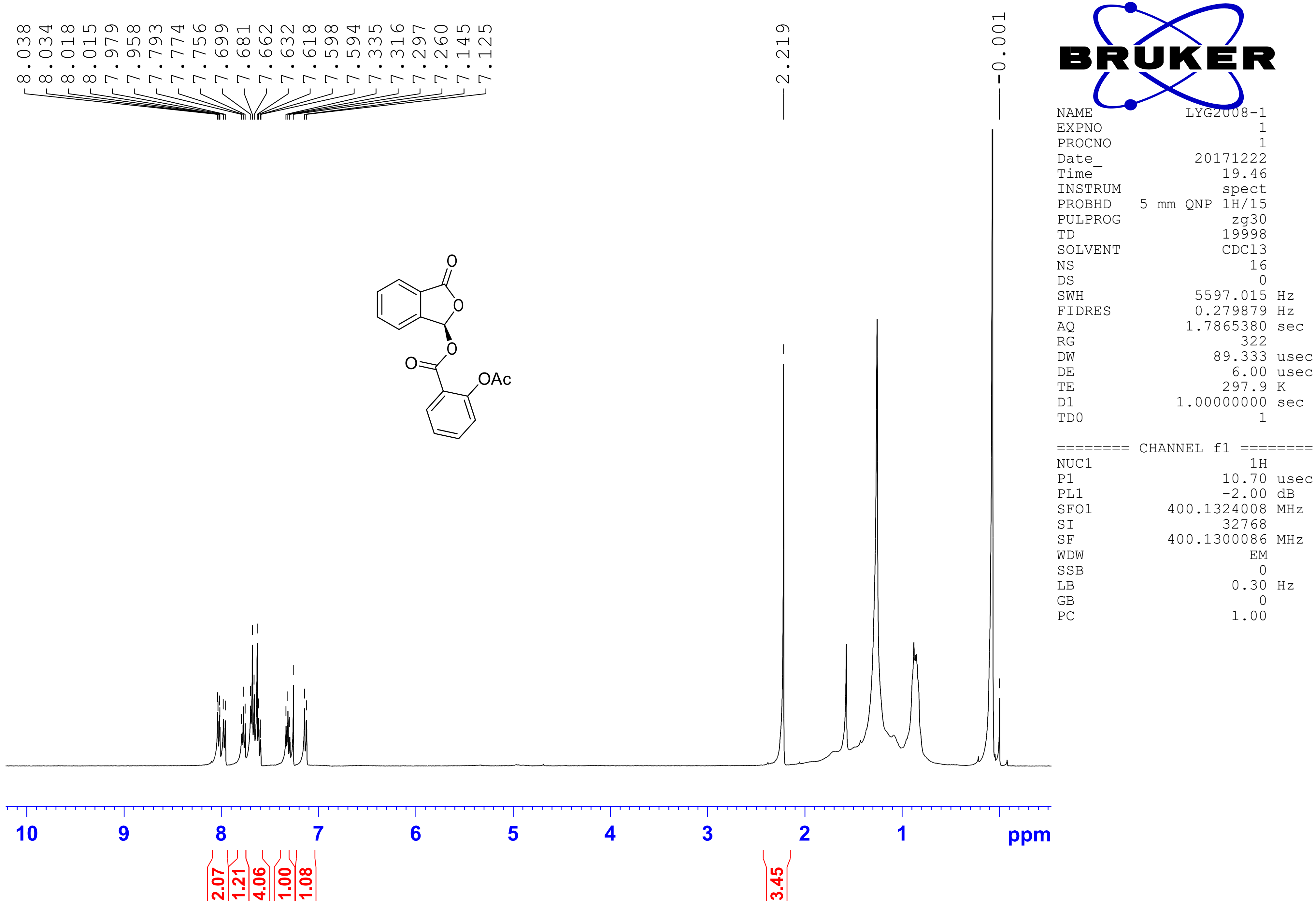

Supplementary Figure 3 ^{1}H NMR spectrum of **3**

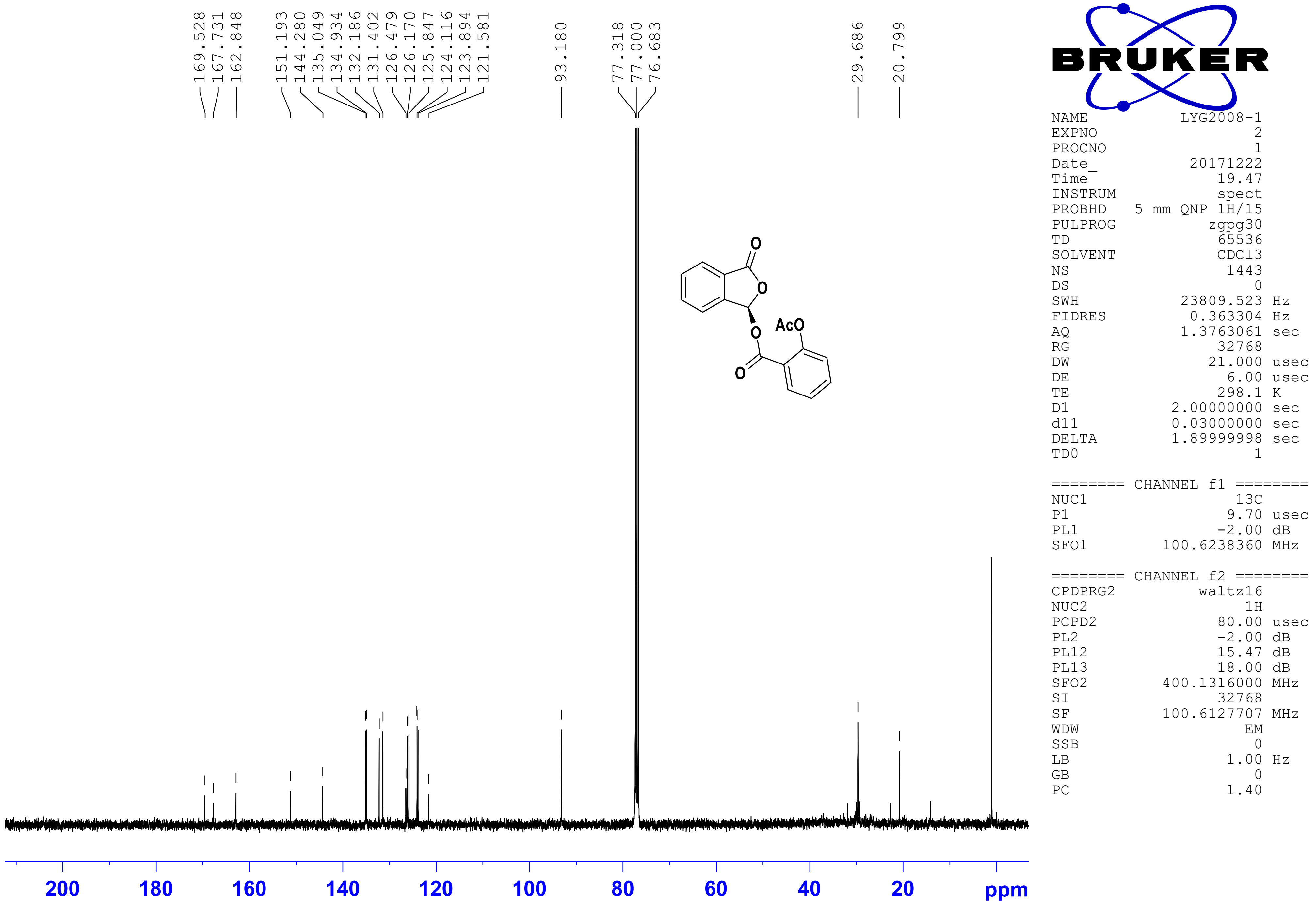

Supplementary Figure 4 ^{13}C NMR spectrum of **3**

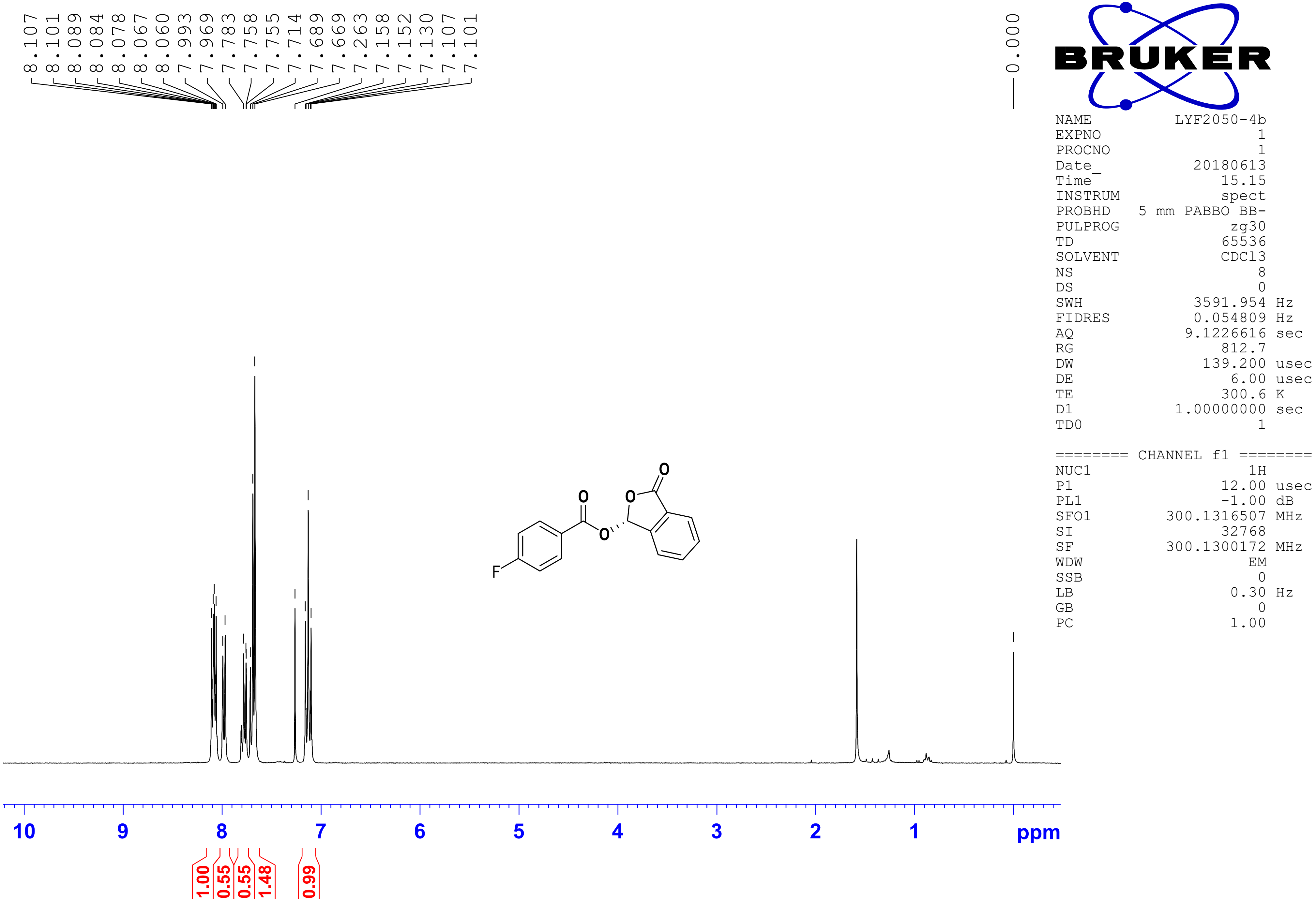

Supplementary Figure 5 ^{1}H NMR spectrum of **4**

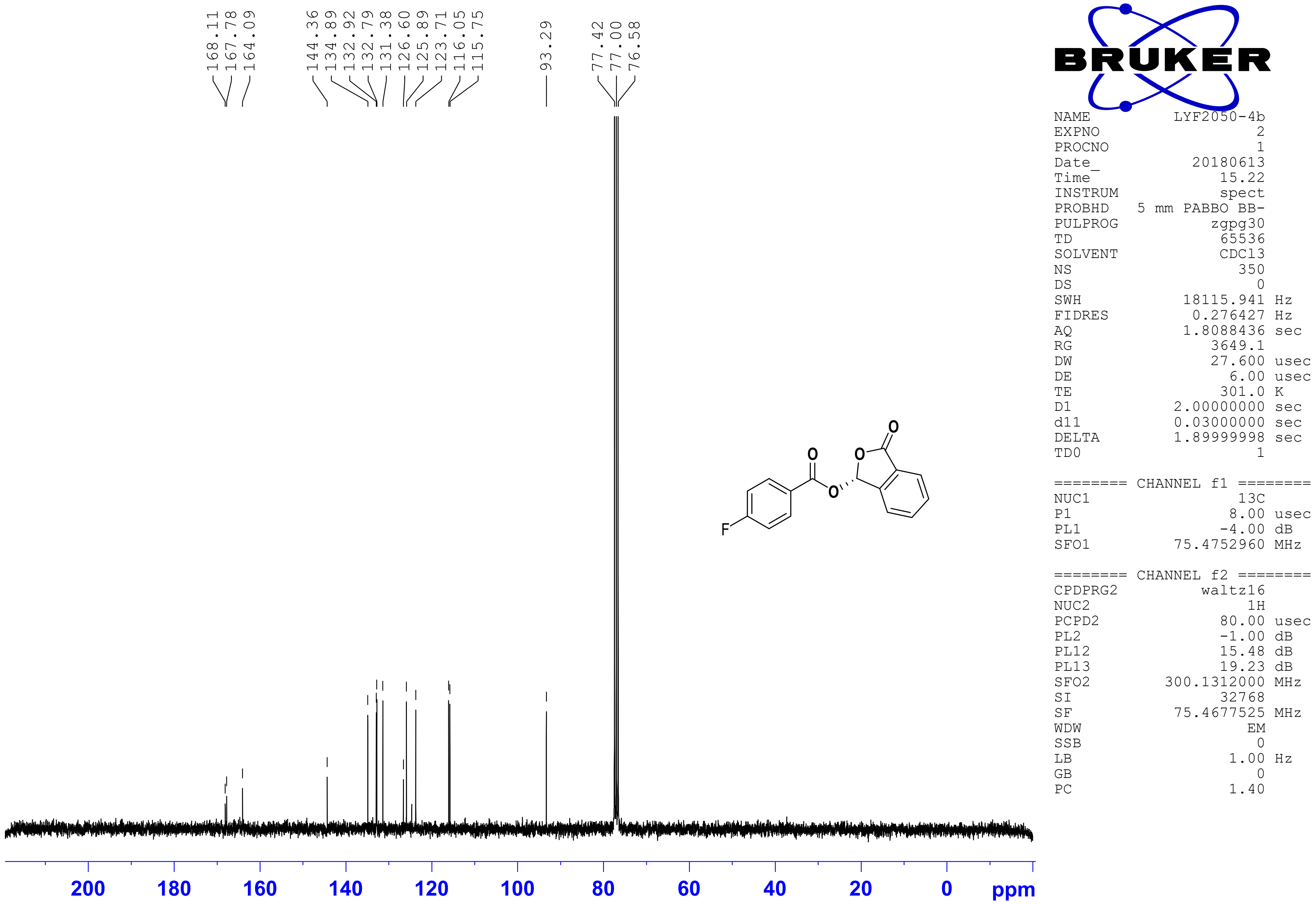

Supplementary Figure 6 ^{13}C NMR spectrum of **4**

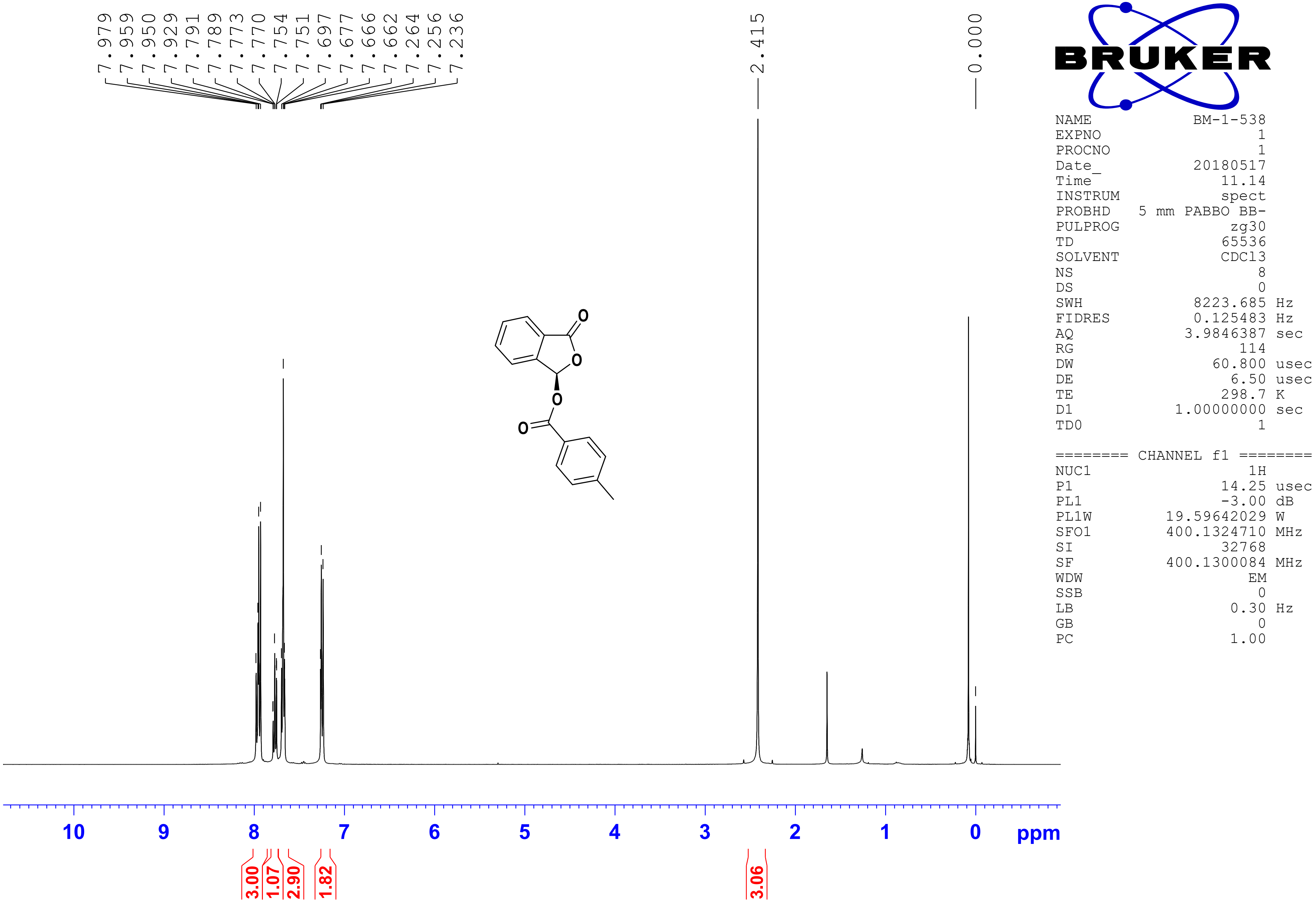

Supplementary Figure 7 ^{1}H NMR spectrum of **5**

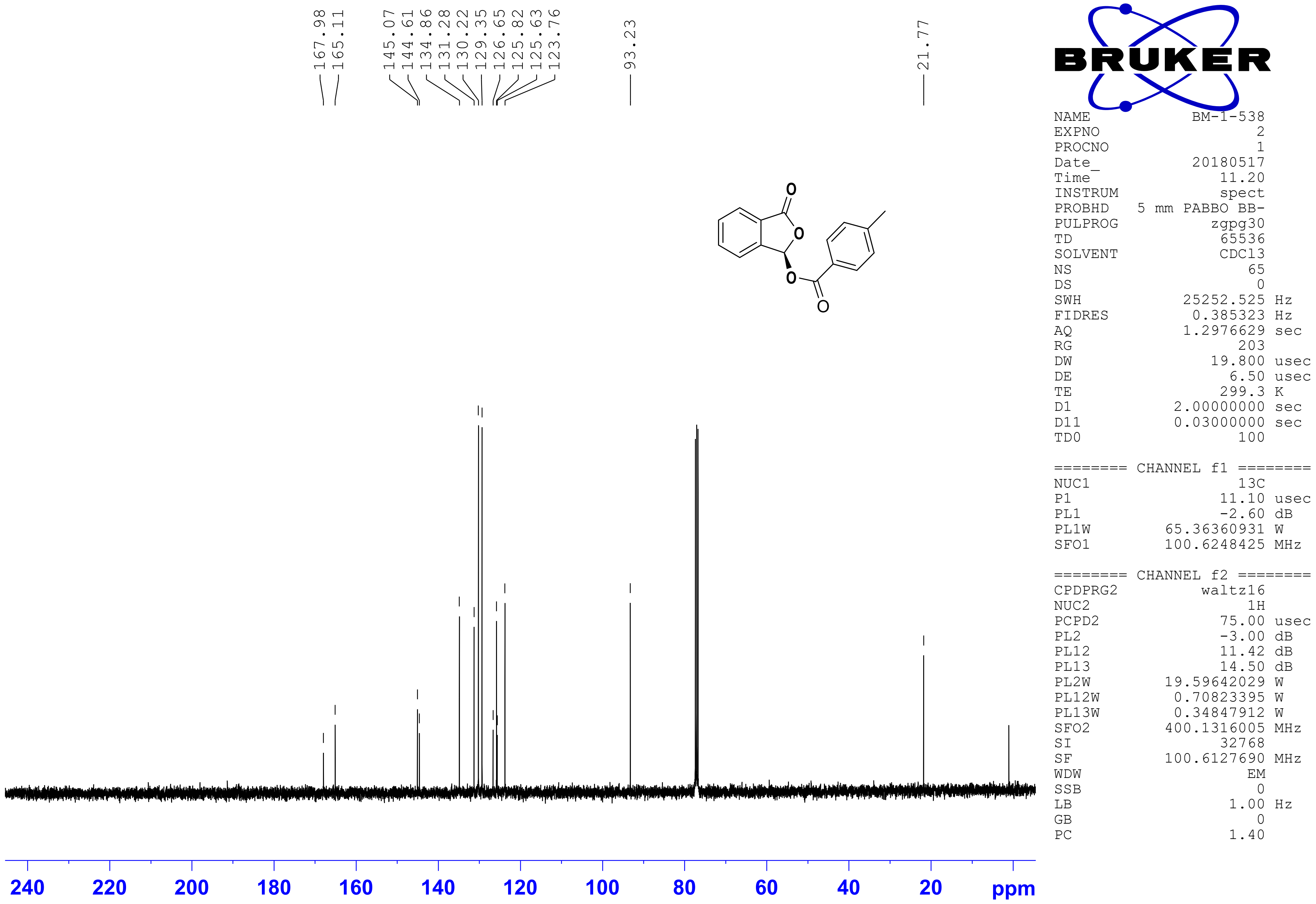

Supplementary Figure 8 ^{13}C NMR spectrum of **5**

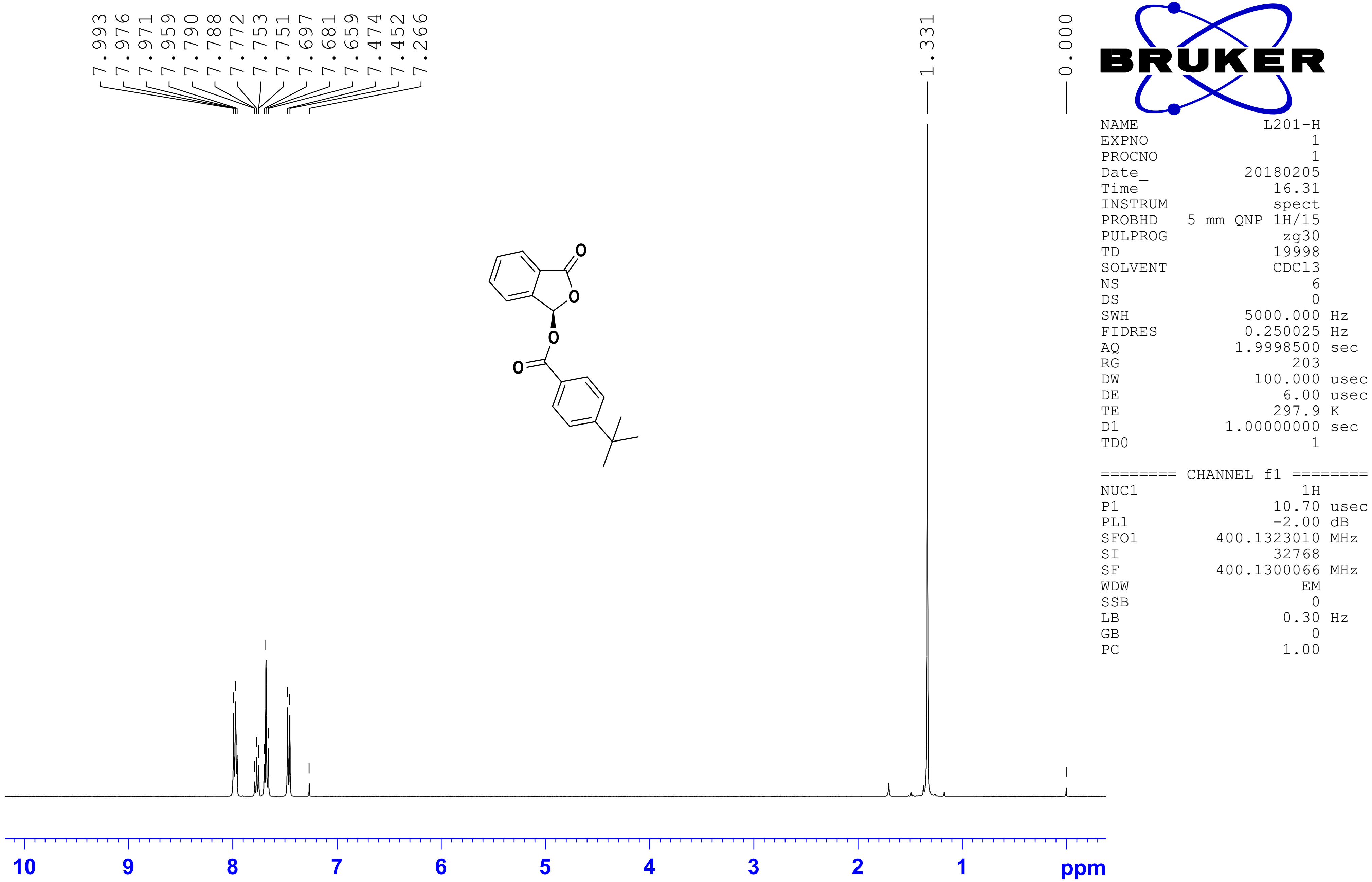

Supplementary Figure 9 ^{1}H NMR spectrum of **6**

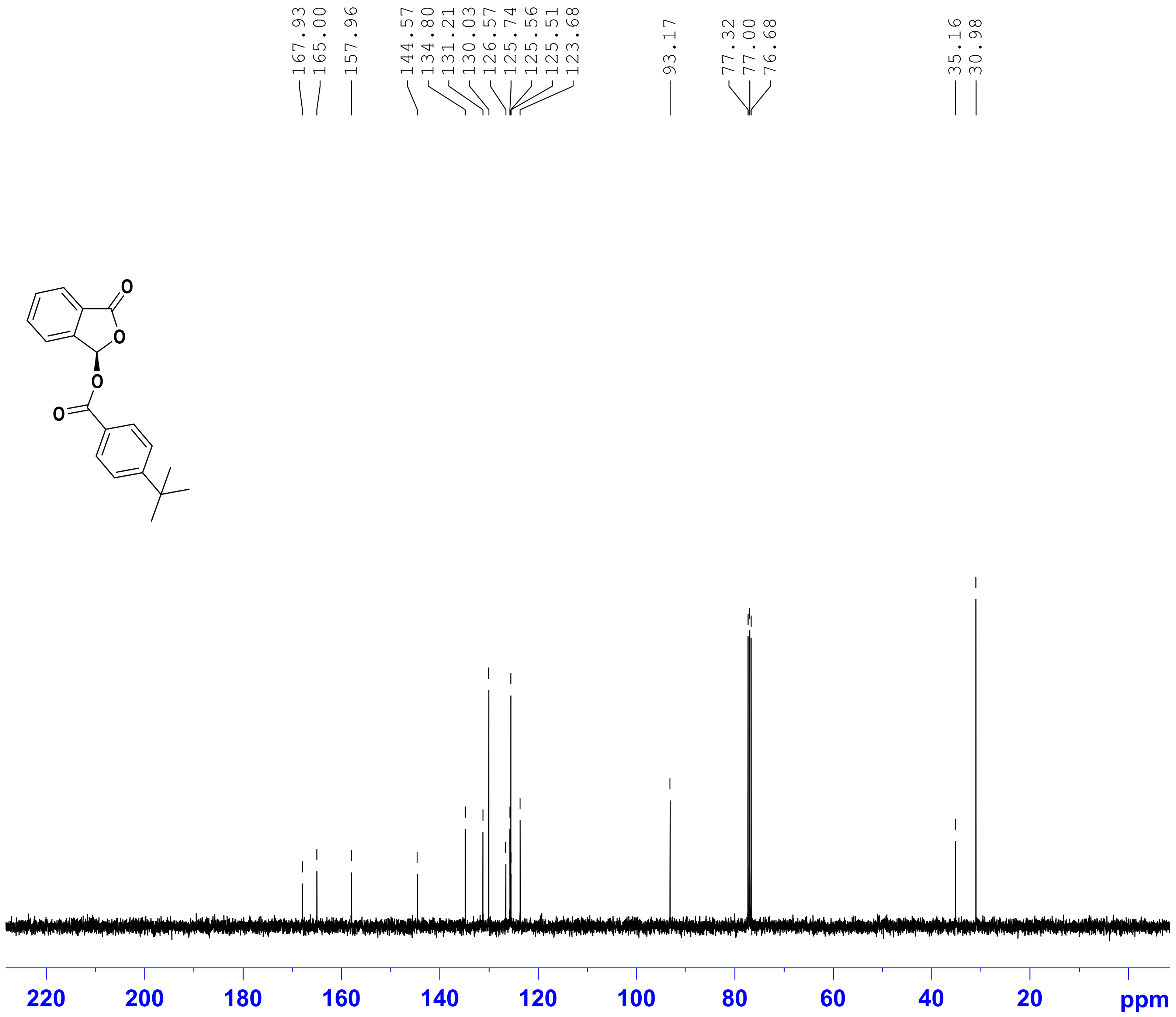

NAME	L201-C
EXPNO	1
PROCNO	1
Date_	20180205
Time	16.33
INSTRUM	spect
PROBHD	5 mm QNP 1H/15
PULPROG	zgpg30
TD	65536
SOLVENT	CDCl3
NS	45
DS	0
SWH	23809.523 Hz
FIDRES	0.363304 Hz
AQ	1.3763061 sec
RG	32768
DW	21.000 usec
DE	6.00 usec
TE	298.0 K
D1	2.00000000 sec
d11	0.03000000 sec
DELTA	1.89999998 sec
TD0	1

======== CHANNEL f1 ========

NUC1	13C
P1	9.70 usec
PL1	-2.00 dB
SFO1	100.6238360 MHz

======== CHANNEL f2 ========

CPDPRG2	waltz16
NUC2	1H
PCPD2	80.00 usec
PL2	-2.00 dB
PL12	15.47 dB
PL13	18.00 dB
SFO2	400.1316000 MHz
SI	32768
SF	100.6127755 MHz
WDW	EM
SSB	0
LB	1.00 Hz
GB	0
PC	1.40

Supplementary Figure 10 ^{13}C NMR spectrum of **6**

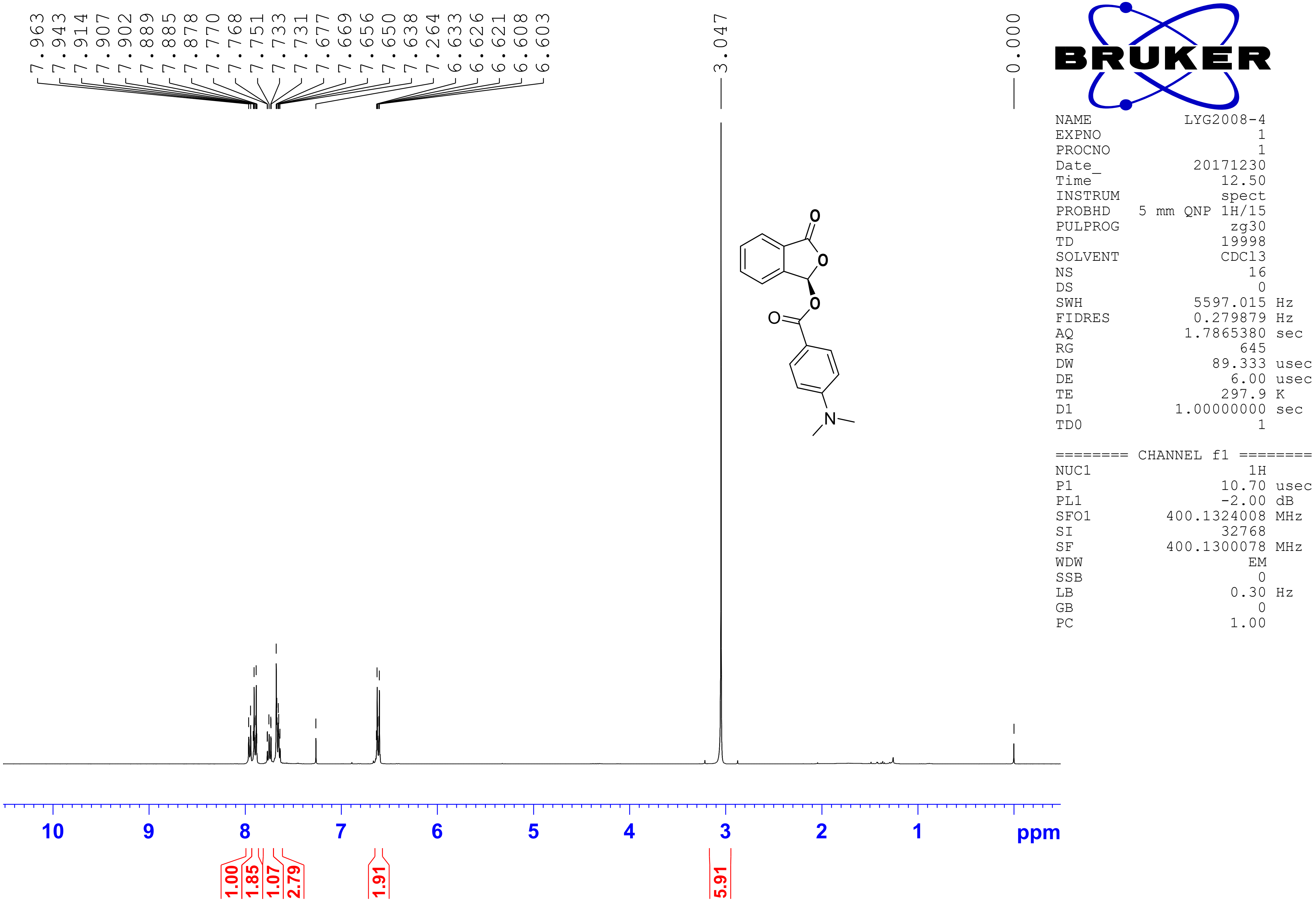

Supplementary Figure 11 ^{1}H NMR spectrum of **7**

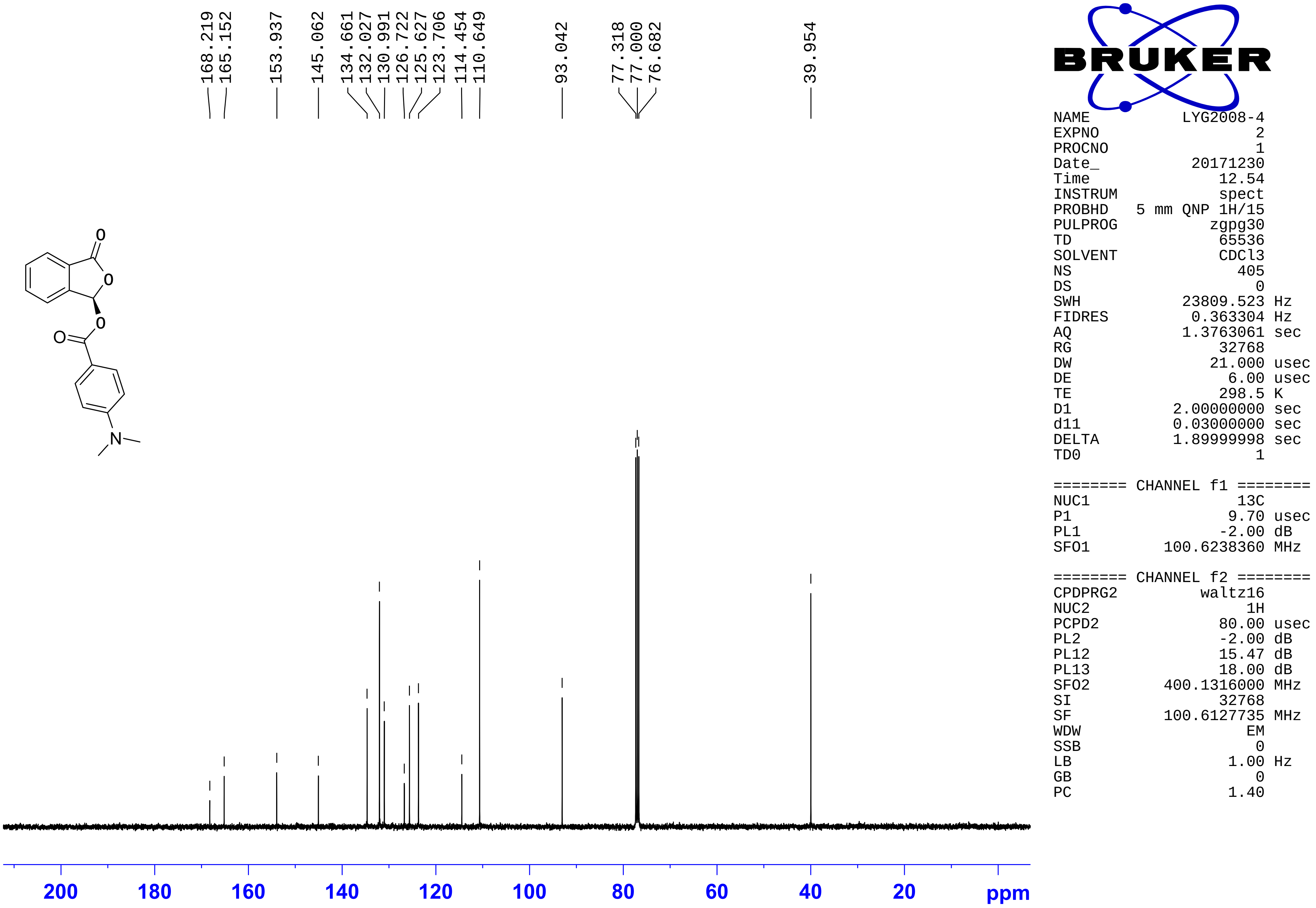

Supplementary Figure 12 ^{13}C NMR spectrum of **7**

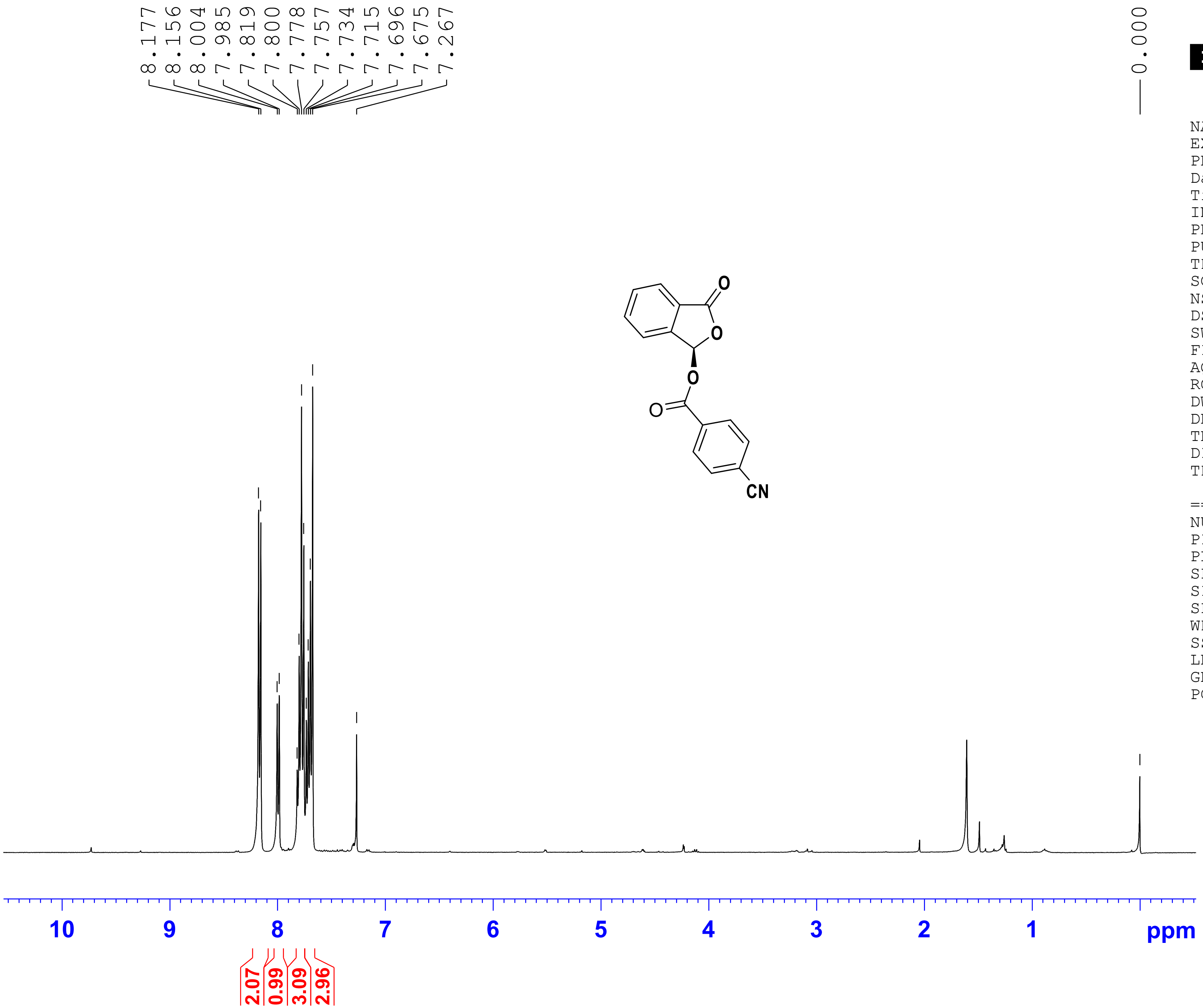

NAME	LYG2046-2a
EXPNO	1
PROCNO	1
Date_	20180612
Time	19.52
INSTRUM	spect
PROBHD	5 mm QNP 1H/15
PULPROG	zg30
TD	19998
SOLVENT	CDCl3
NS	16
DS	0
SWH	5000.000 Hz
FIDRES	0.250025 Hz
AQ	1.9998500 sec
RG	812
DW	100.000 usec
DE	6.00 usec
TE	299.5 K
D1	1.00000000 sec
TD0	1

======== CHANNEL f1 ========

NUC1	1H
P1	10.70 usec
PL1	-2.00 dB
SFO1	400.1322007 MHz
SI	32768
SF	400.1300062 MHz
WDW	EM
SSB	0
LB	0.30 Hz
GB	0
PC	1.00

Supplementary Figure 13 ^{1}H NMR spectrum of **8**

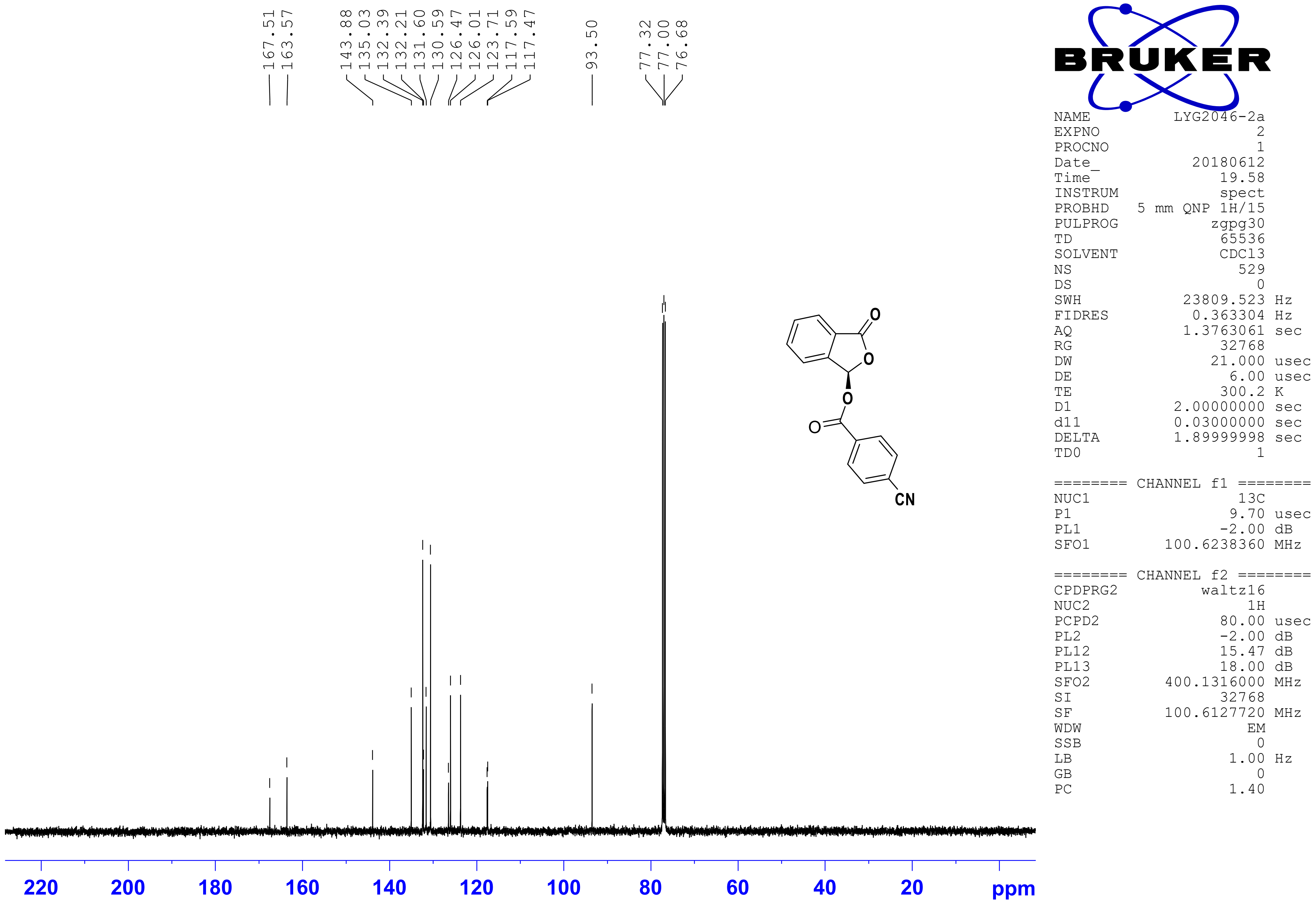

Supplementary Figure 14 ^{13}C NMR spectrum of **8**

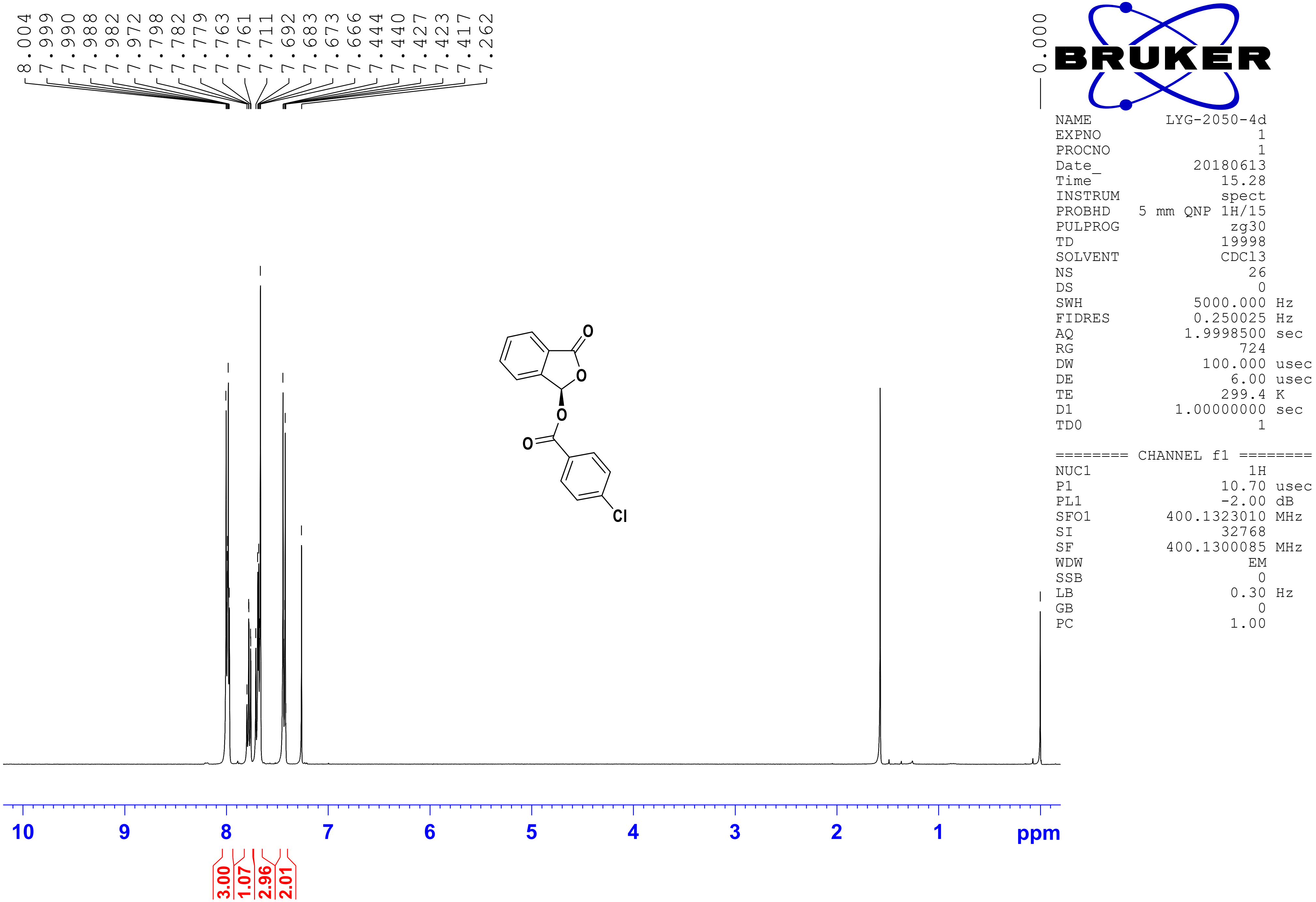

Supplementary Figure 15 ^{1}H NMR spectrum of **9**

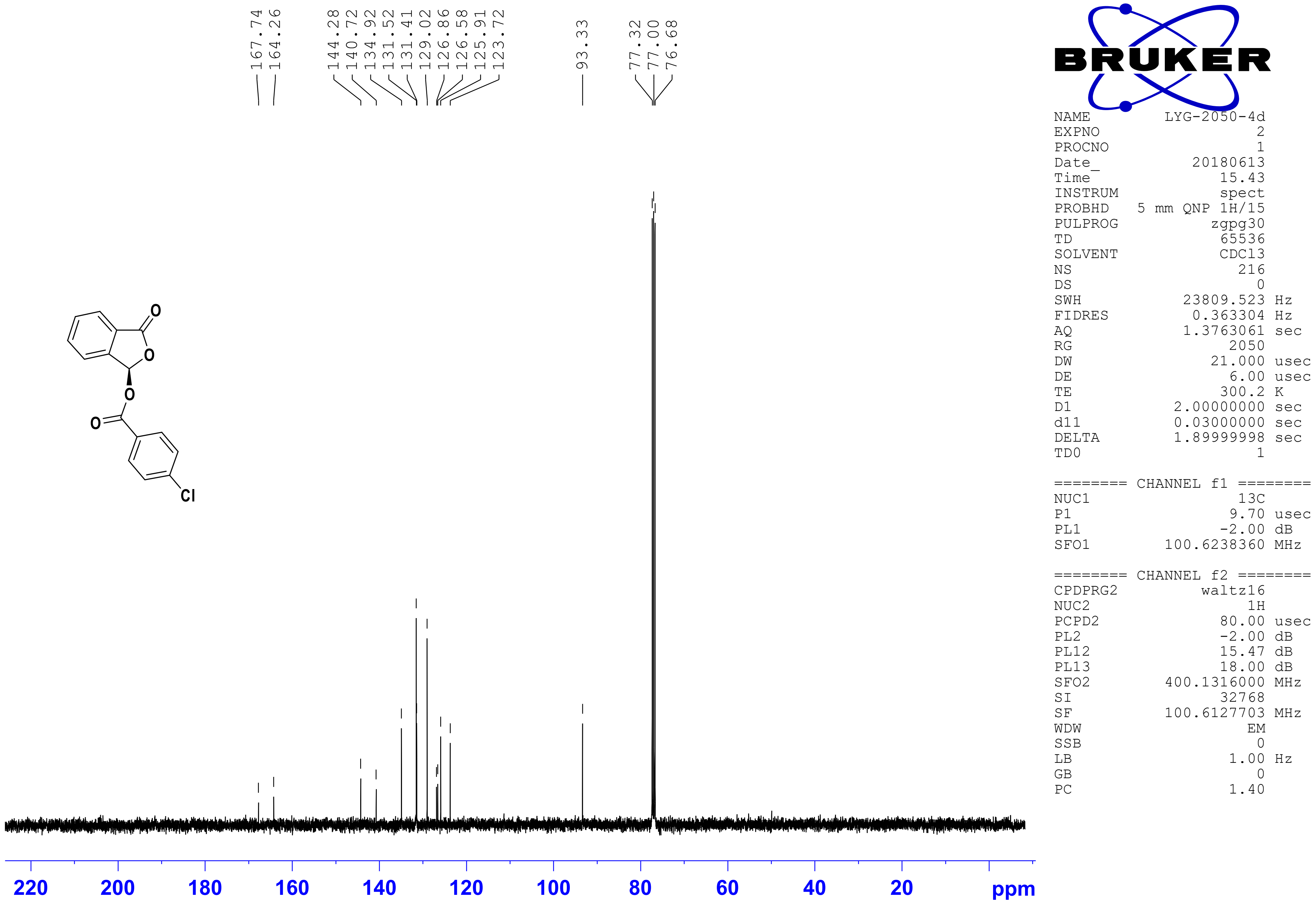

Supplementary Figure 16 ^{13}C NMR spectrum of **9**

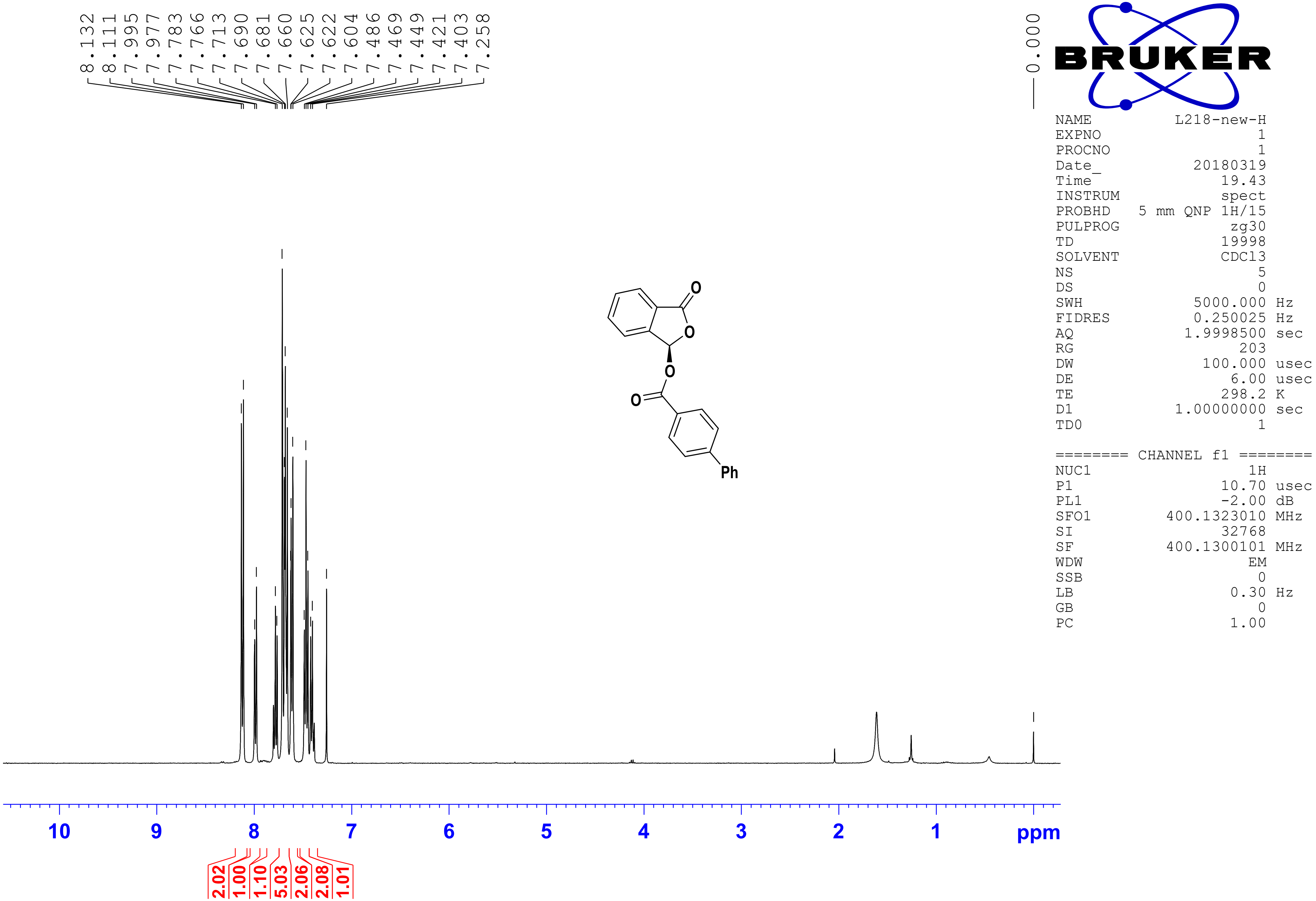

Supplementary Figure 17 ^{1}H NMR spectrum of **10**

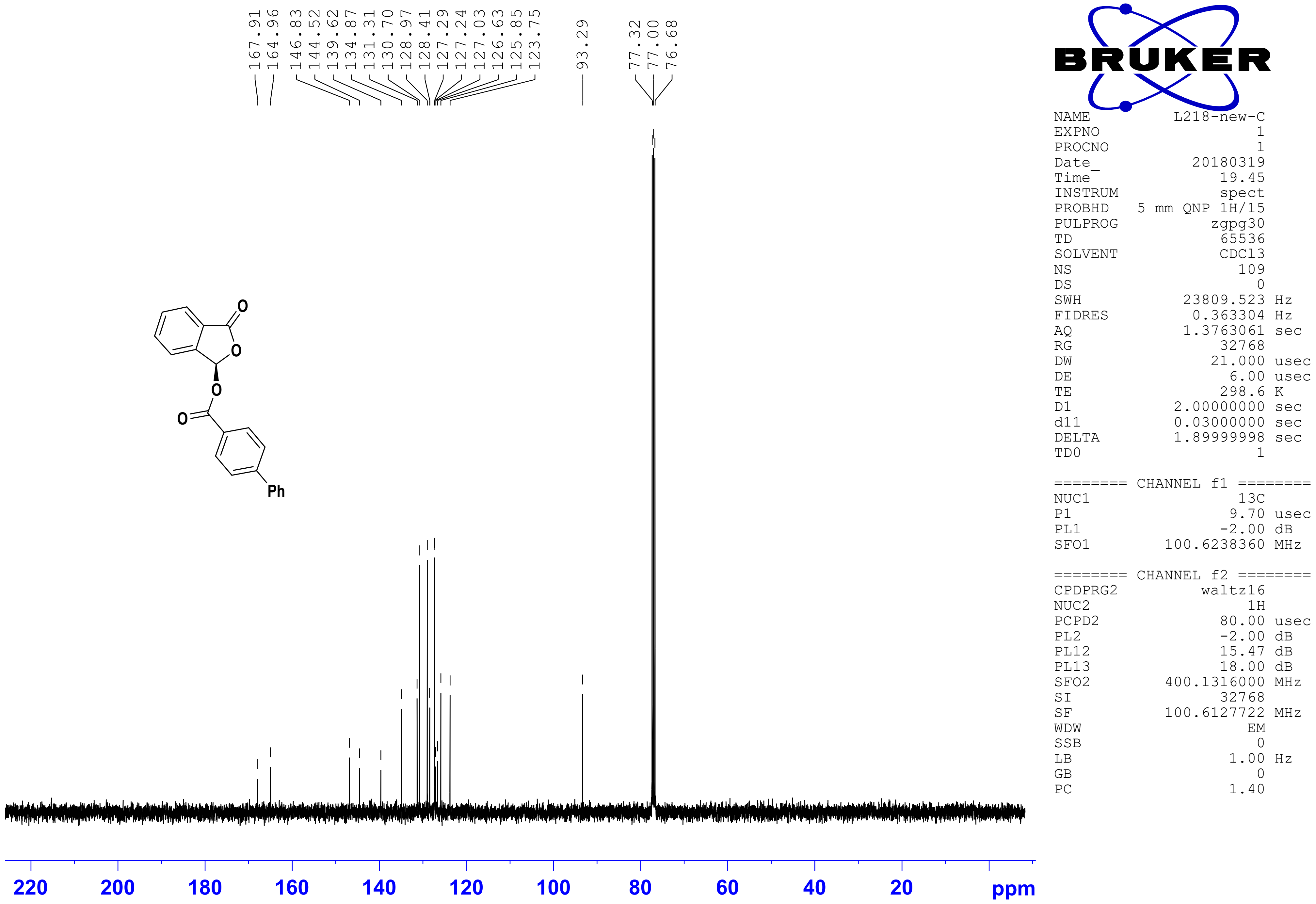

Supplementary Figure 18 ^{13}C NMR spectrum of **10**

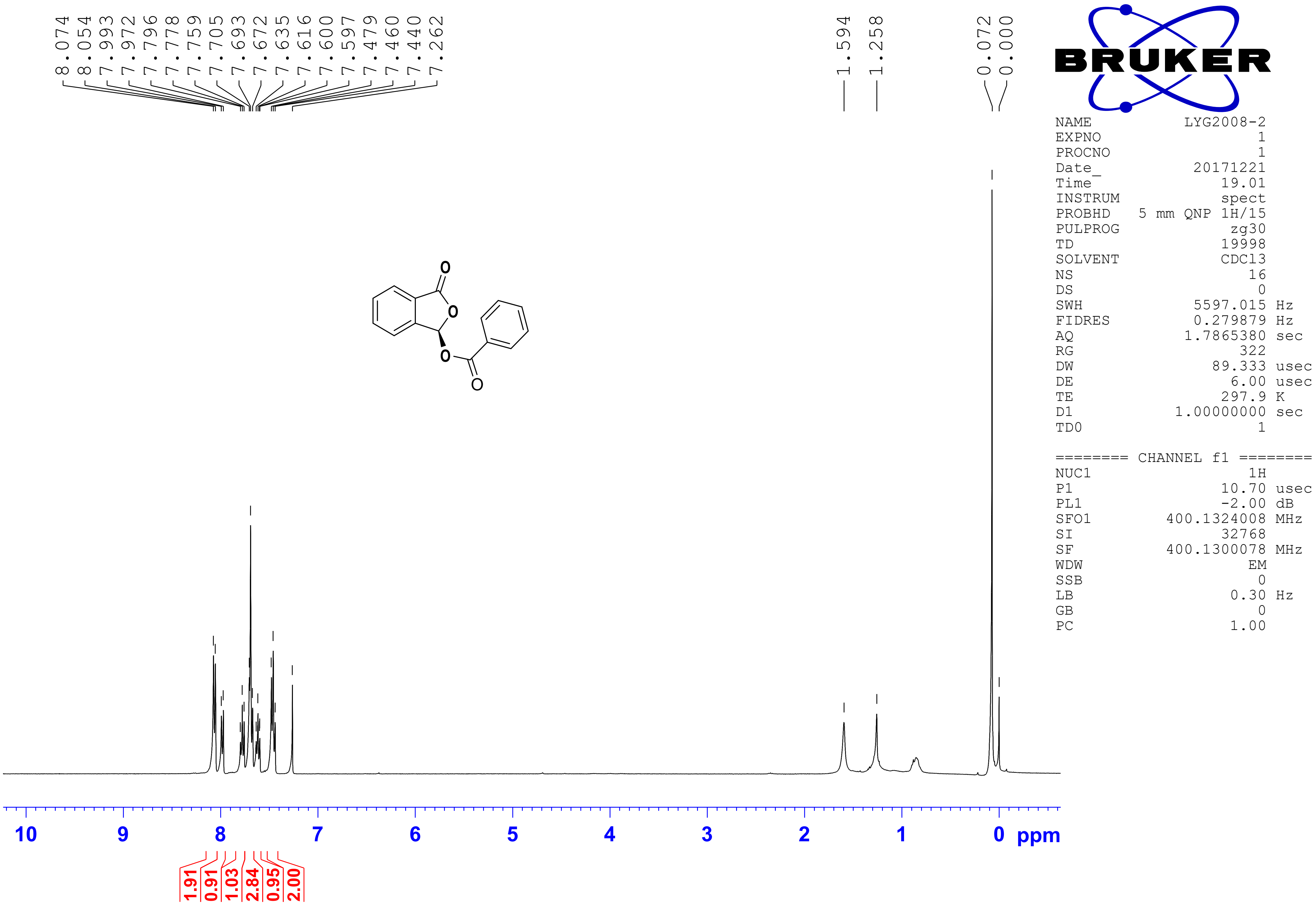

Supplementary Figure 19 ^{1}H NMR spectrum of **11**

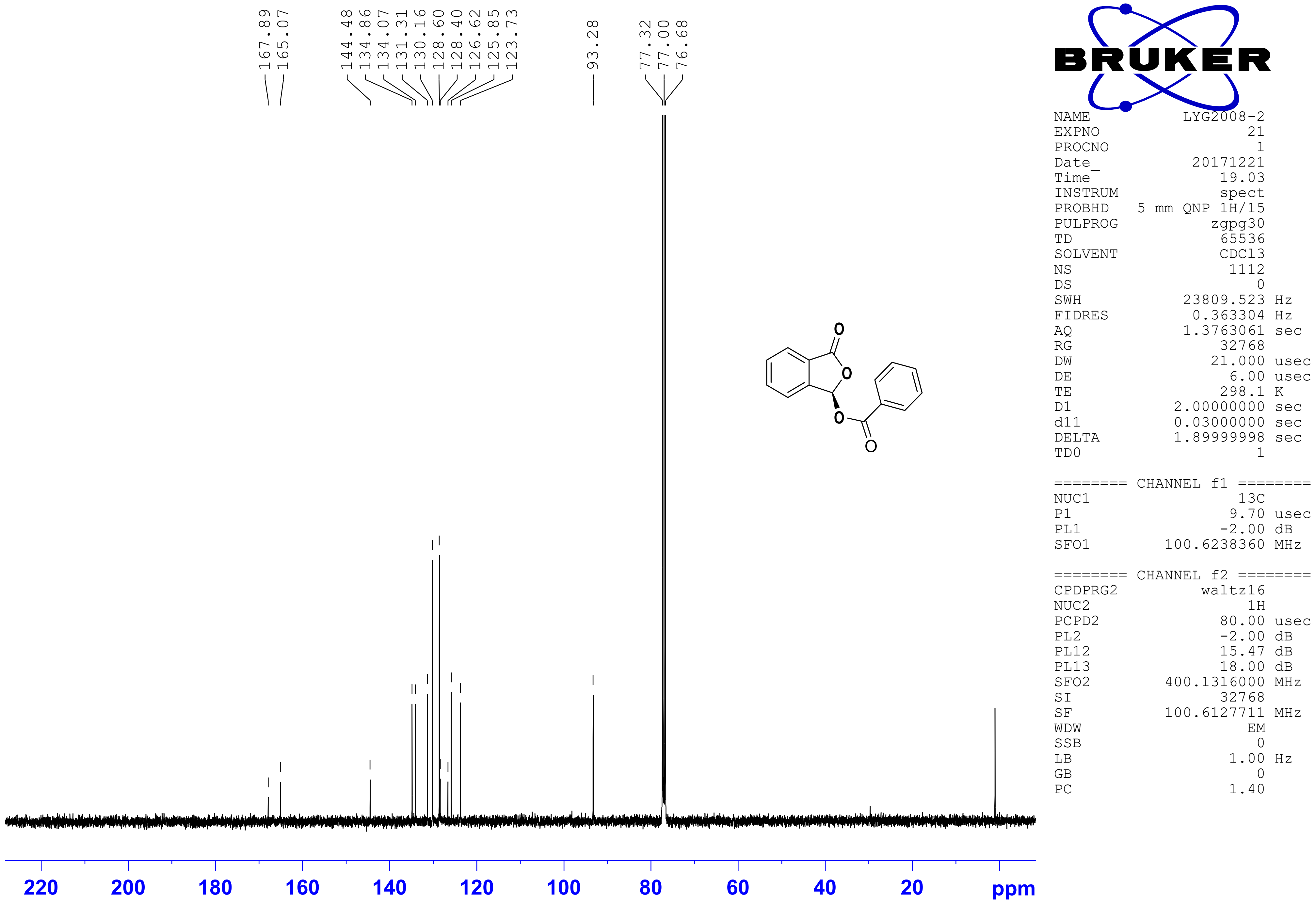

Supplementary Figure 20 ^{13}C NMR spectrum of **11**

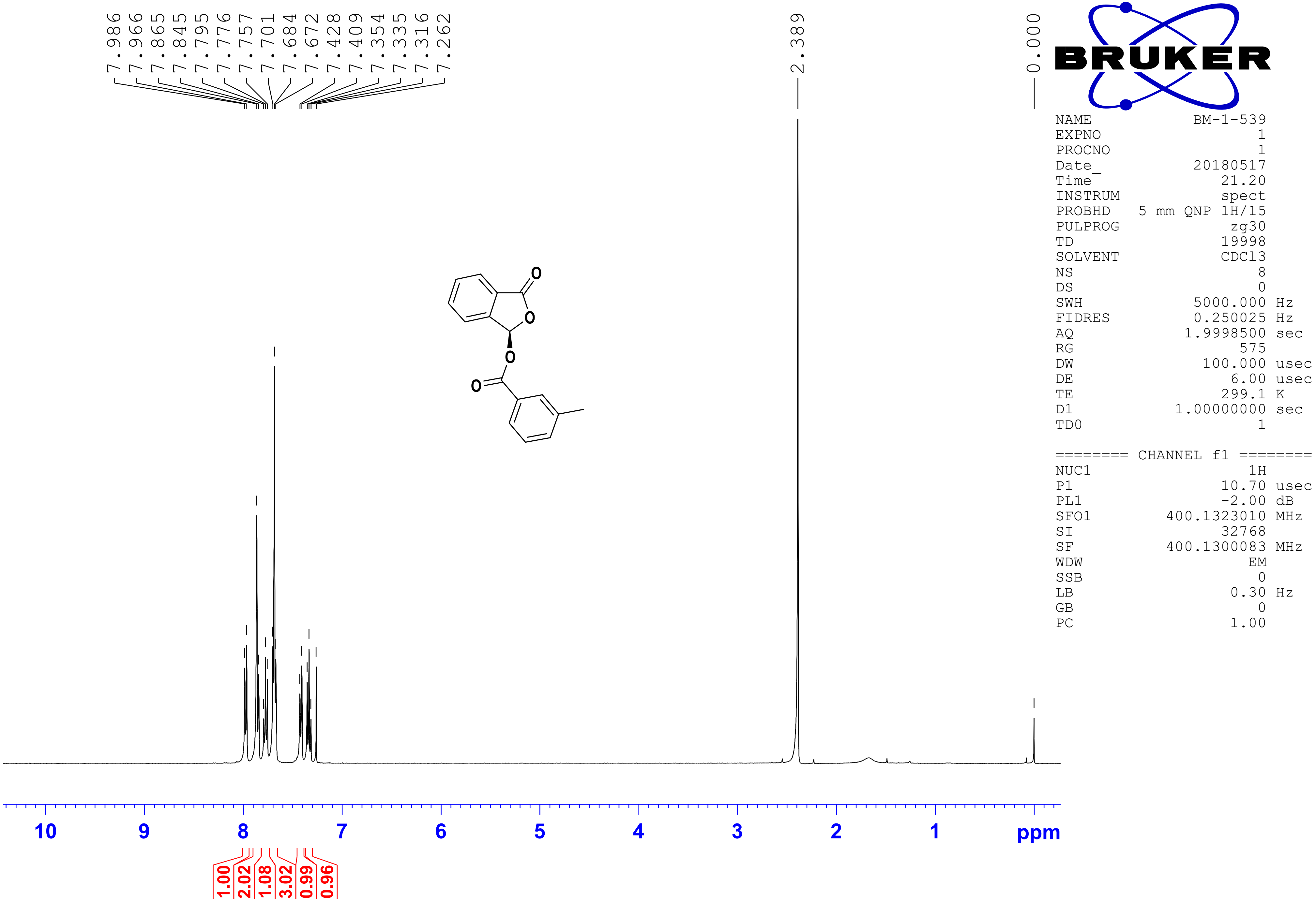

Supplementary Figure 21 ^{1}H NMR spectrum of **12**

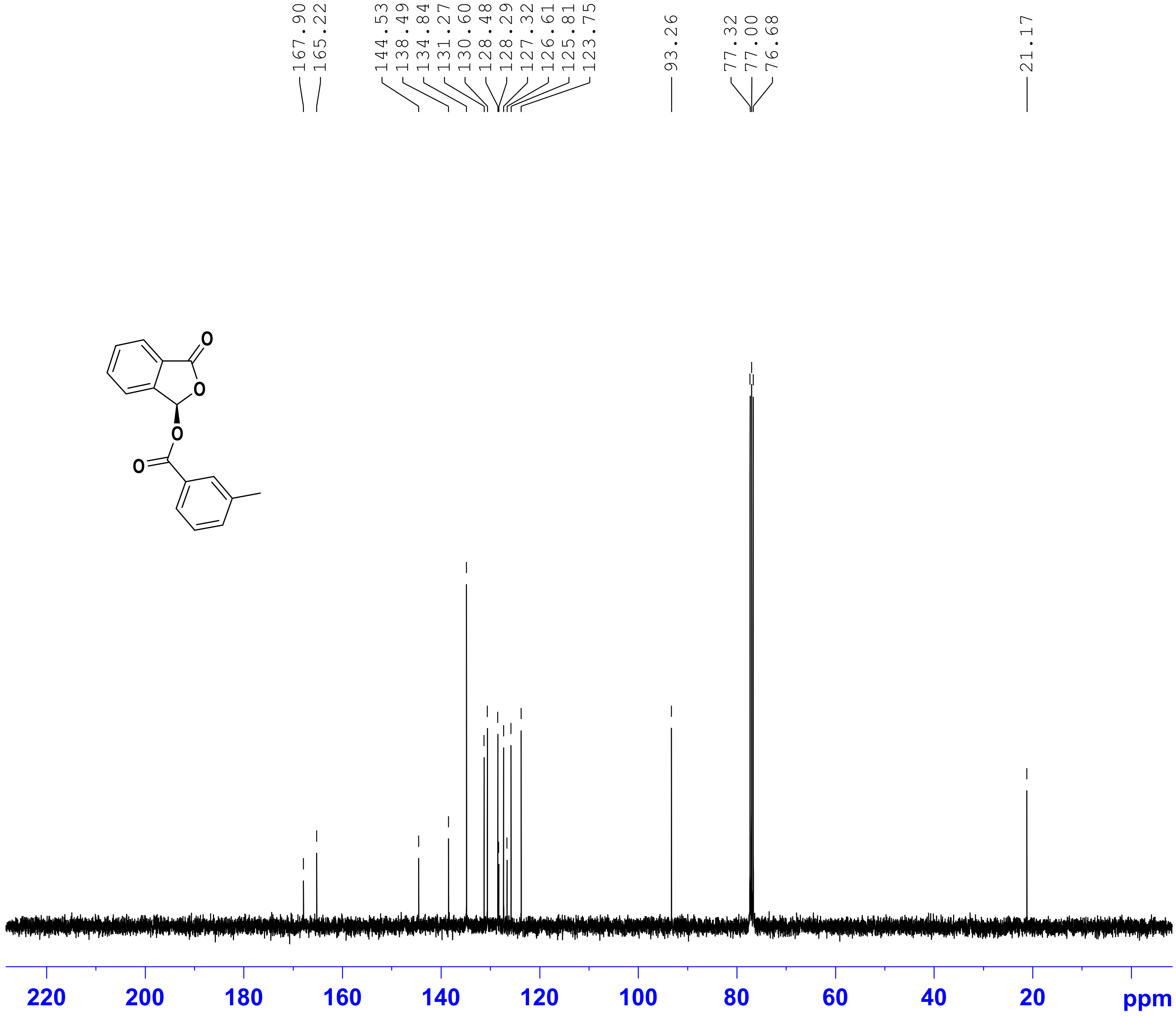

NAME	BM-1-539
EXPNO	2
PROCNO	1
Date_	20180518
Time	16.21
INSTRUM	spect
PROBHD	5 mm QNP 1H/15
PULPROG	zgpg30
TD	65536
SOLVENT	CDCl3
NS	94
DS	0
SWH	23809.523 Hz
FIDRES	0.363304 Hz
AQ	1.3763061 sec
RG	2050
DW	21.000 usec
DE	6.00 usec
TE	299.7 K
D1	2.00000000 sec
d11	0.03000000 sec
DELTA	1.89999998 sec
TD0	1
======== CHANNEL f1 ========	
NUC1	13C
P1	9.70 usec
PL1	-2.00 dB
SFO1	100.6238360 MHz
======== CHANNEL f2 ========	
CPDPRG2	waltz16
NUC2	1H
PCPD2	80.00 usec
PL2	-2.00 dB
PL12	15.47 dB
PL13	18.00 dB
SFO2	400.1316000 MHz
SI	32768
SF	100.6127723 MHz
WDW	EM
SSB	0
LB	1.00 Hz
GB	0
PC	1.40

Supplementary Figure 22 ^{13}C NMR spectrum of **12**

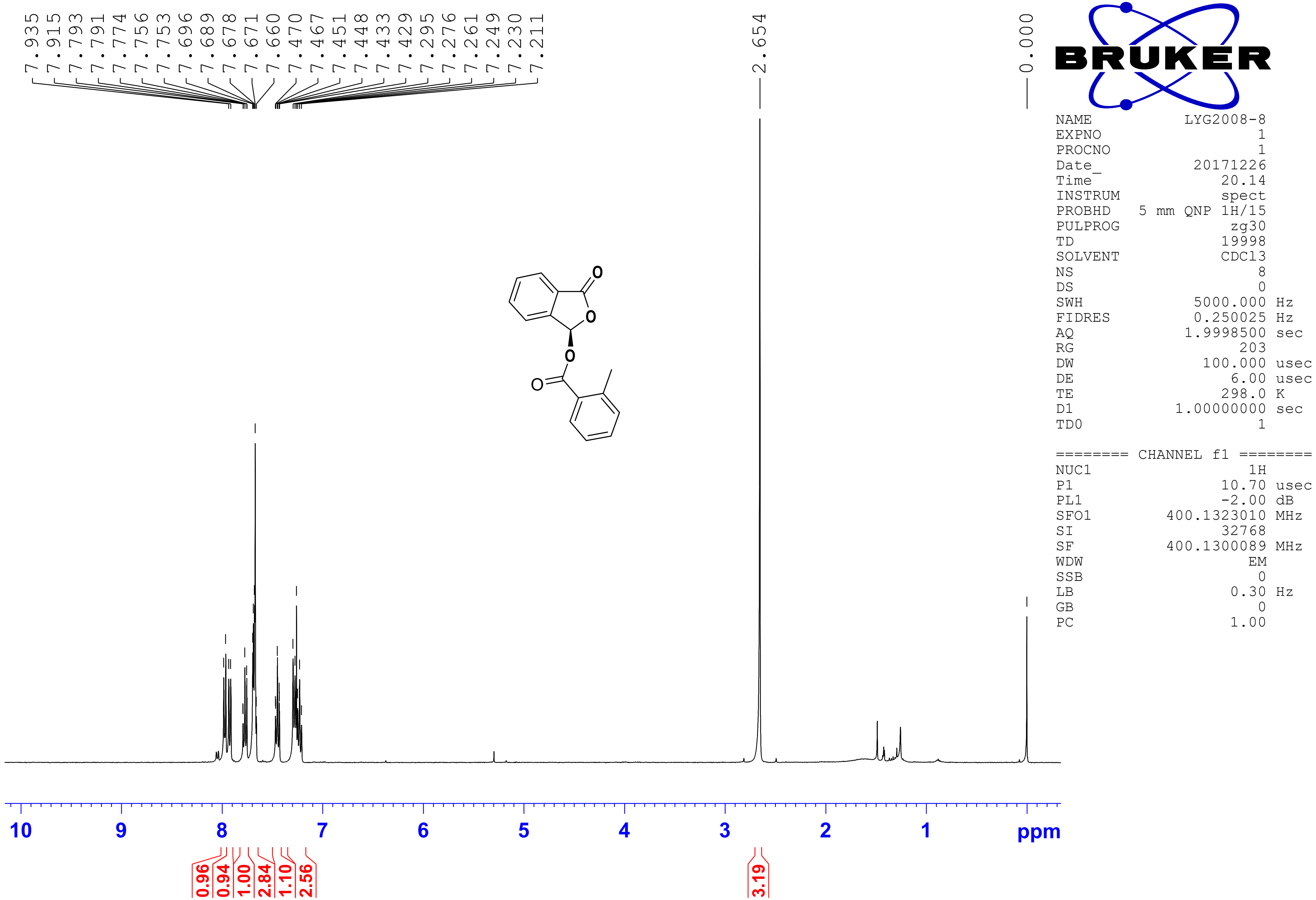

Supplementary Figure 23 ^{1}H NMR spectrum of **13**

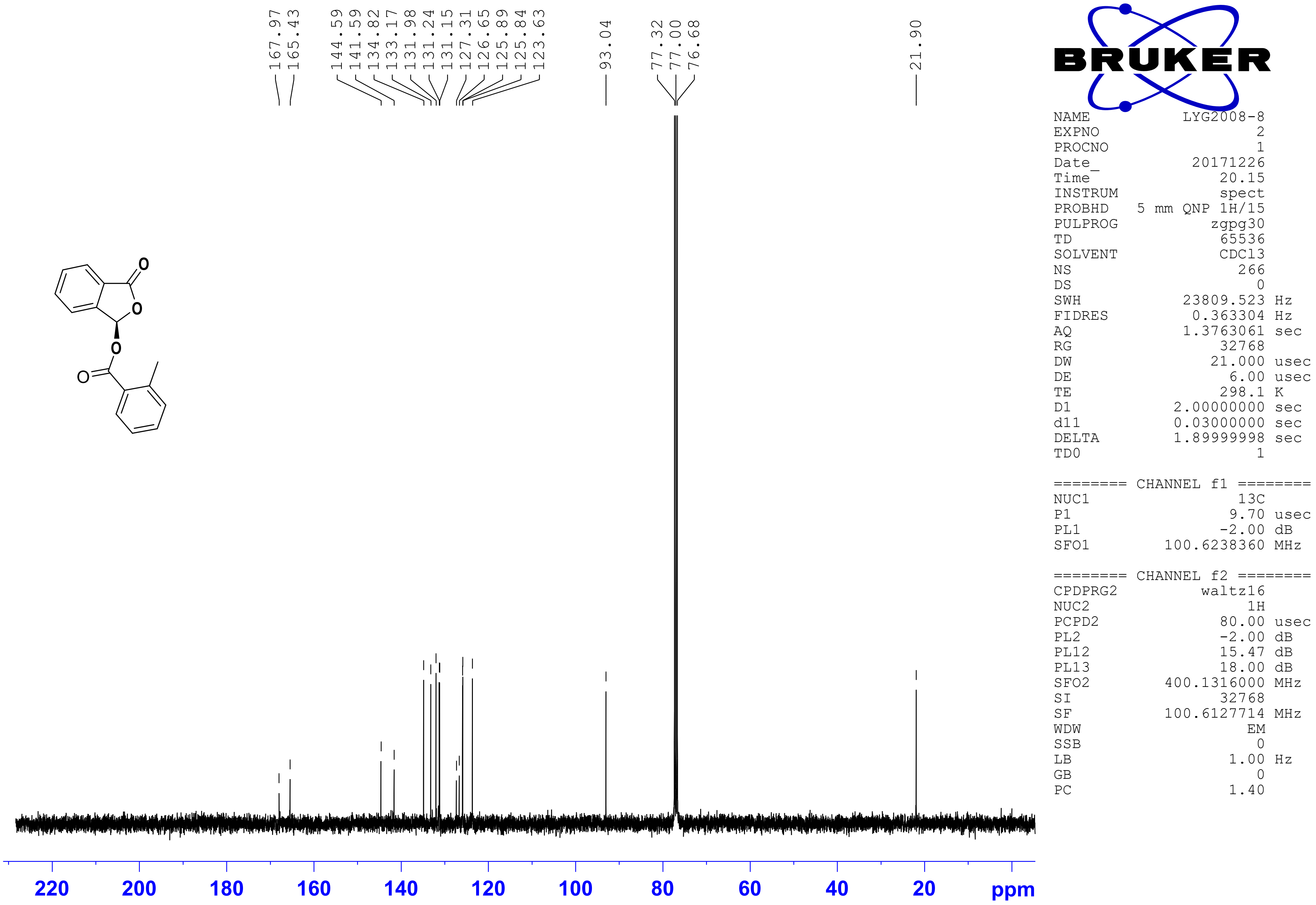

Supplementary Figure 24 ^{13}C NMR spectrum of 13

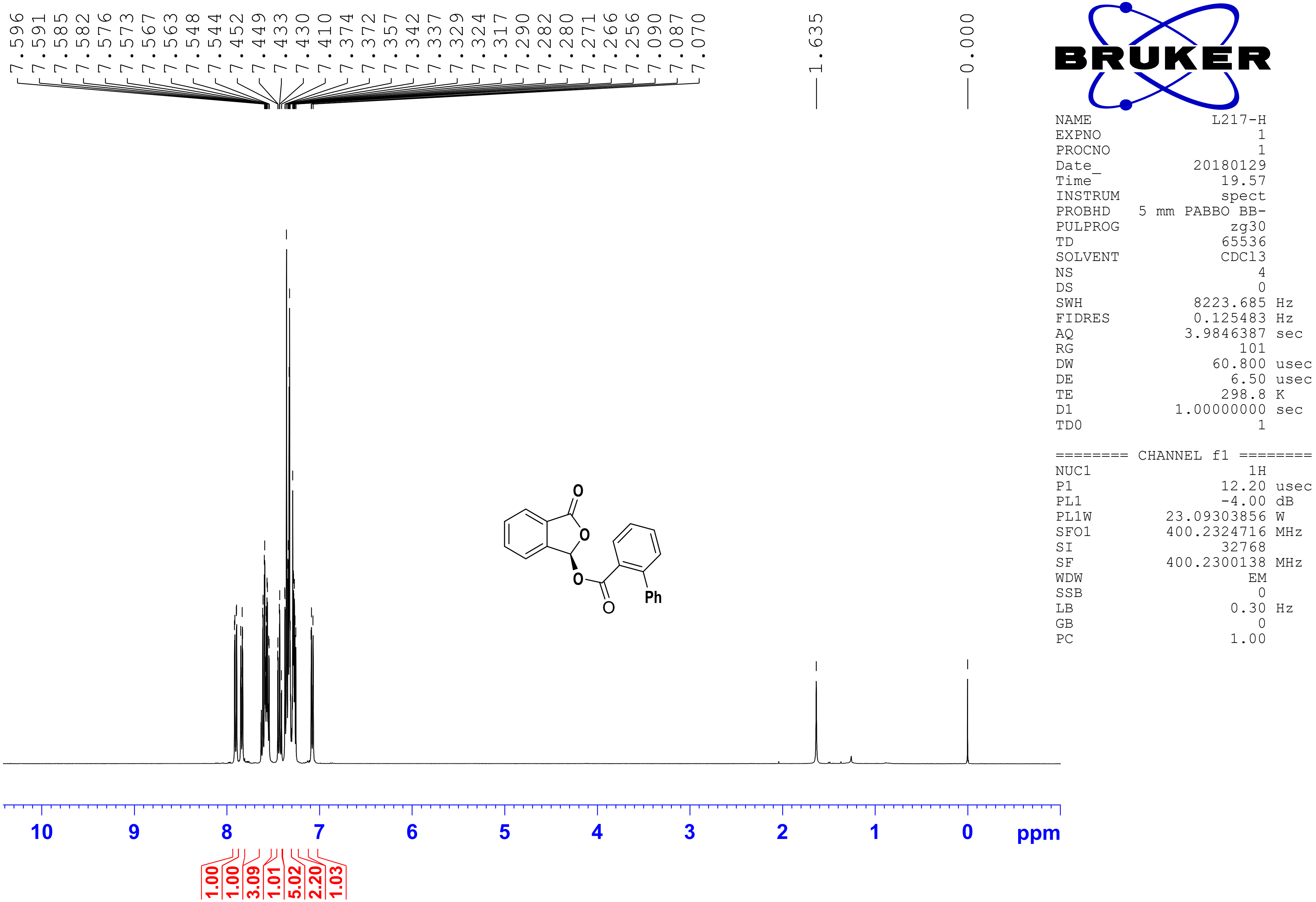

Supplementary Figure 25 ^{1}H NMR spectrum of **14**

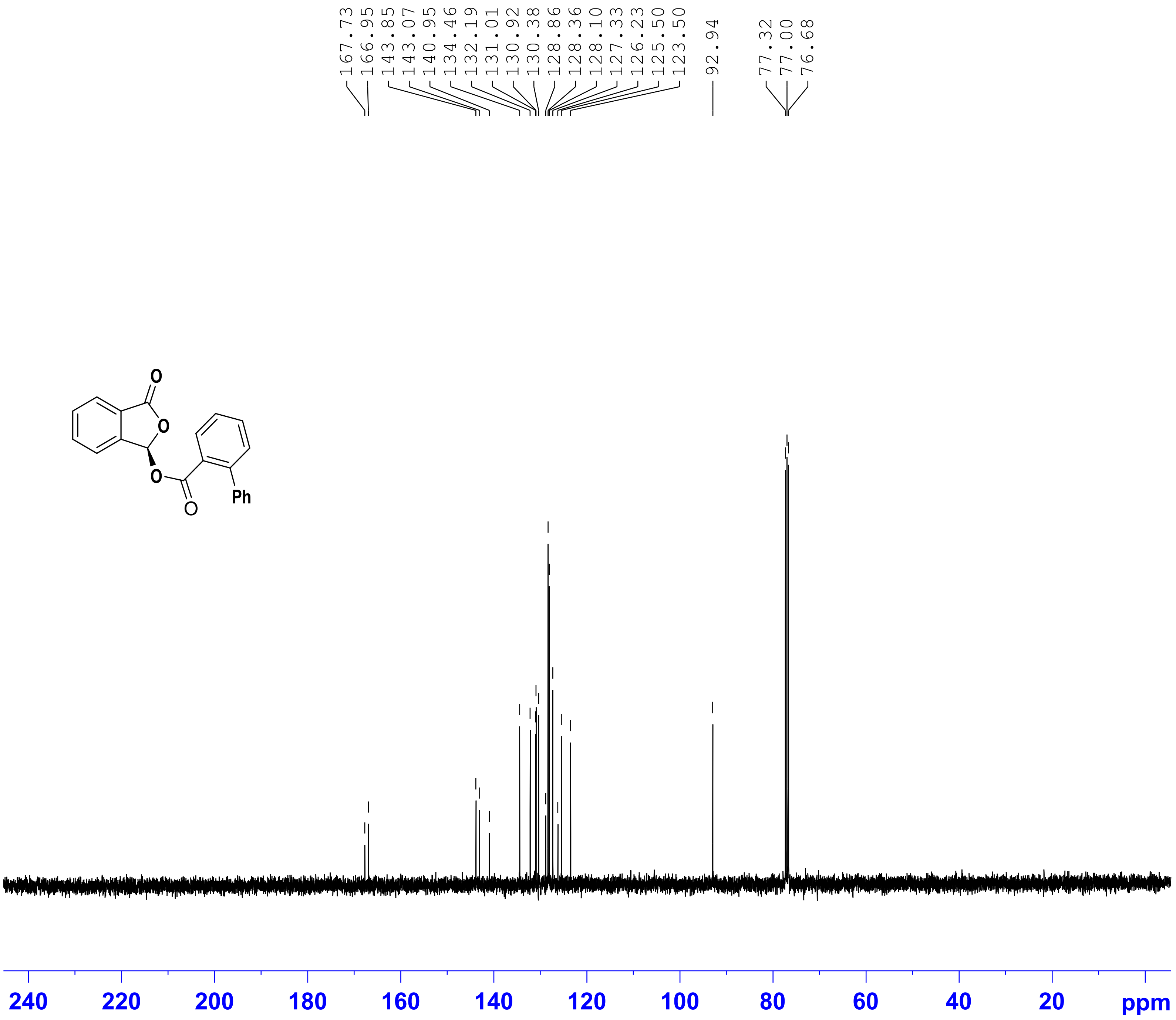

NAME	L217-C
EXPNO	1
PROCNO	1
Date_	20180129
Time	19.42
INSTRUM	spect
PROBHD	5 mm PABBO BB-
PULPROG	zgpg30
TD	65536
SOLVENT	CDCl3
NS	29
DS	4
SWH	25252.525 Hz
FIDRES	0.385323 Hz
AQ	1.2976629 sec
RG	203
DW	19.800 usec
DE	6.50 usec
TE	299.2 K
D1	2.00000000 sec
D11	0.03000000 sec
TD0	100
======== CHANNEL f1 ========	
NUC1	13C
P1	11.30 usec
PL1	-2.00 dB
PL1W	59.71607590 W
SFO1	100.6499905 MHz
======== CHANNEL f2 ========	
CPDPRG2	waltz16
NUC2	1H
PCPD2	80.00 usec
PL2	-4.00 dB
PL12	12.33 dB
PL13	13.89 dB
PL2W	23.09303856 W
PL12W	0.53762704 W
PL13W	0.37538856 W
SFO2	400.2316009 MHz
SI	32768
SF	100.6379210 MHz
WDW	EM
SSB	0
LB	1.00 Hz
GB	0
PC	1.40

Supplementary Figure 26 ^{13}C NMR spectrum of **14**

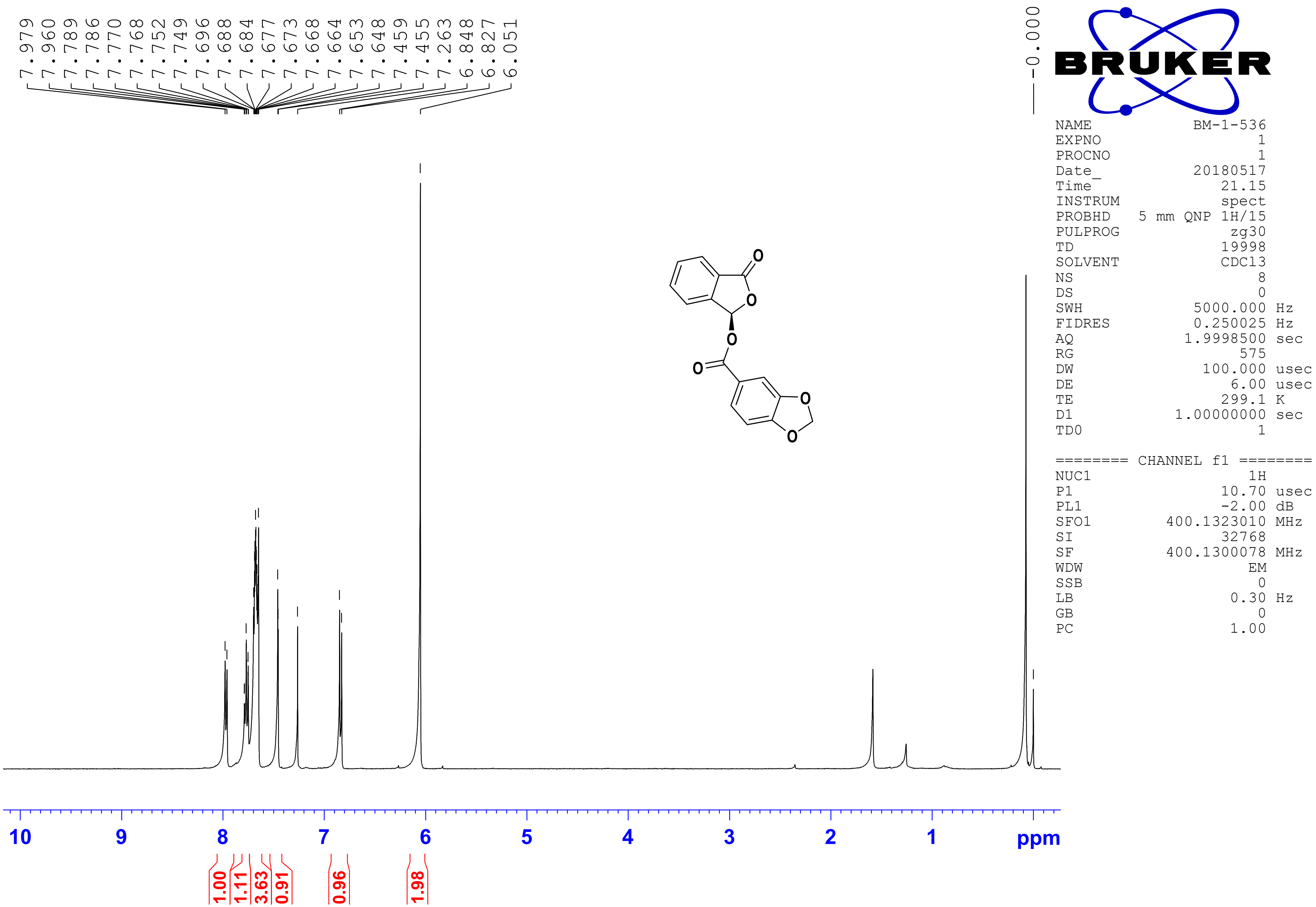

Supplementary Figure 27 ^{1}H NMR spectrum of **15**

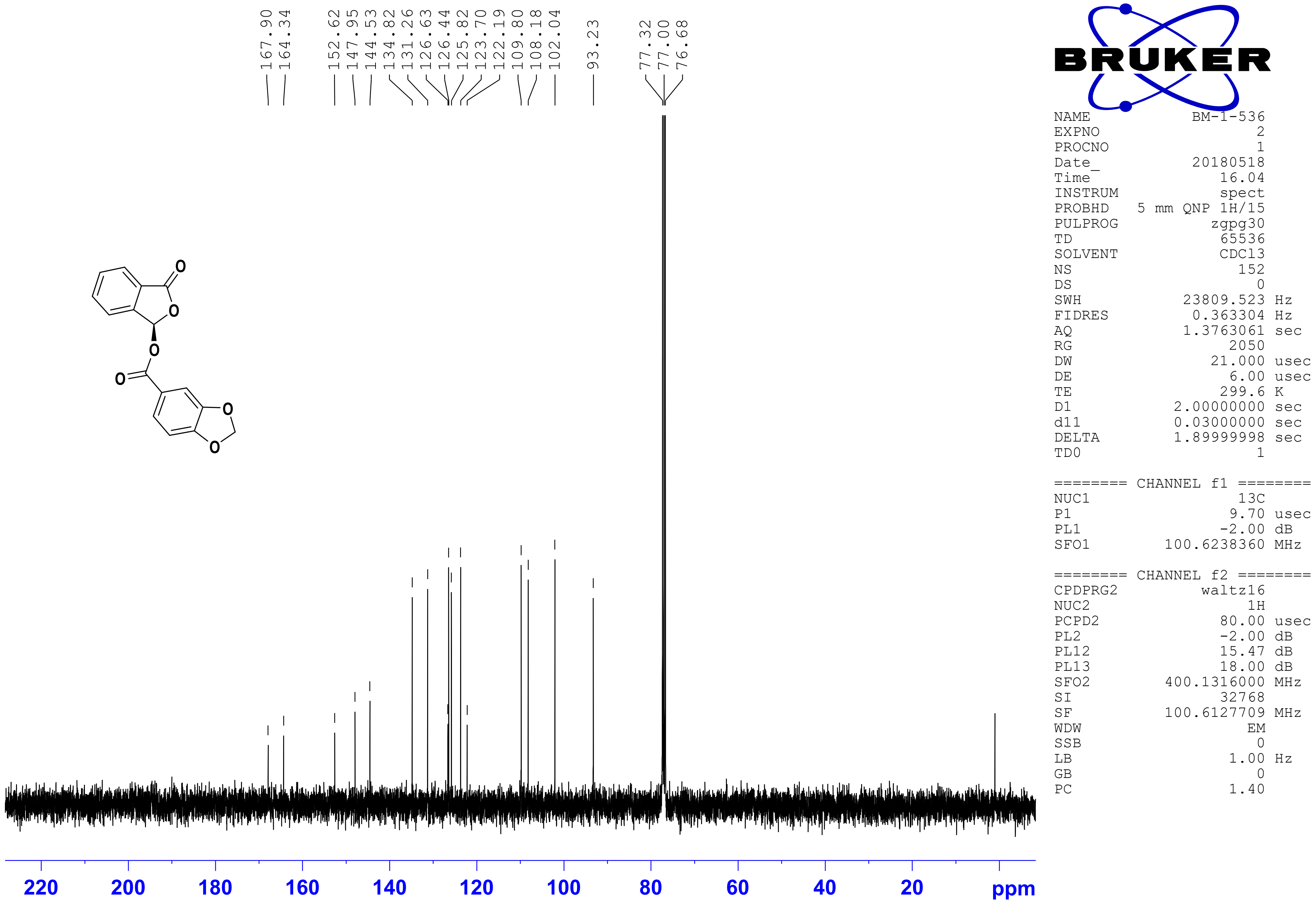

Supplementary Figure 28 ^{13}C NMR spectrum of **15**

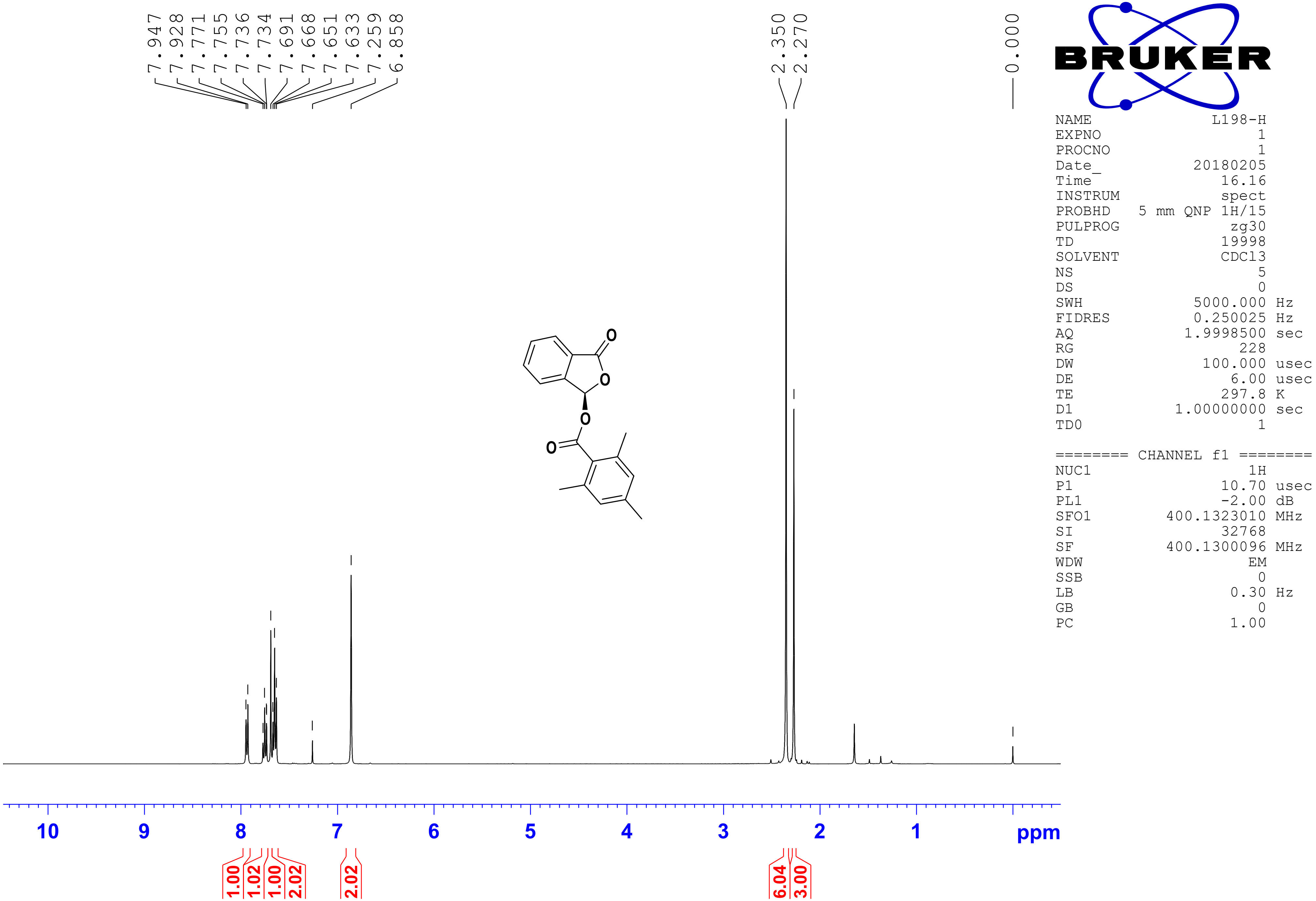

Supplementary Figure 29 ^{1}H NMR spectrum of **16**

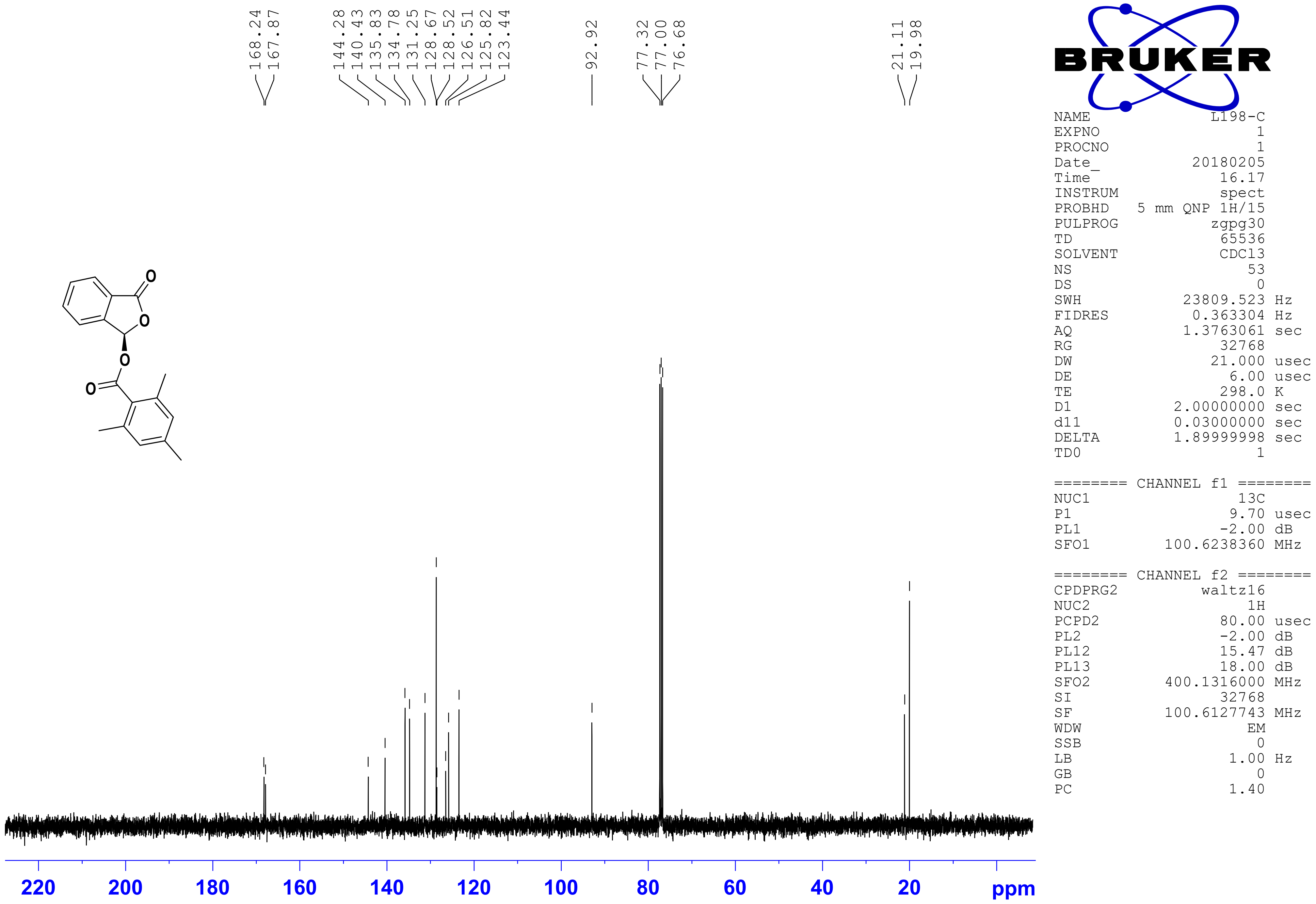

Supplementary Figure 30 ^{13}C NMR spectrum of **16**

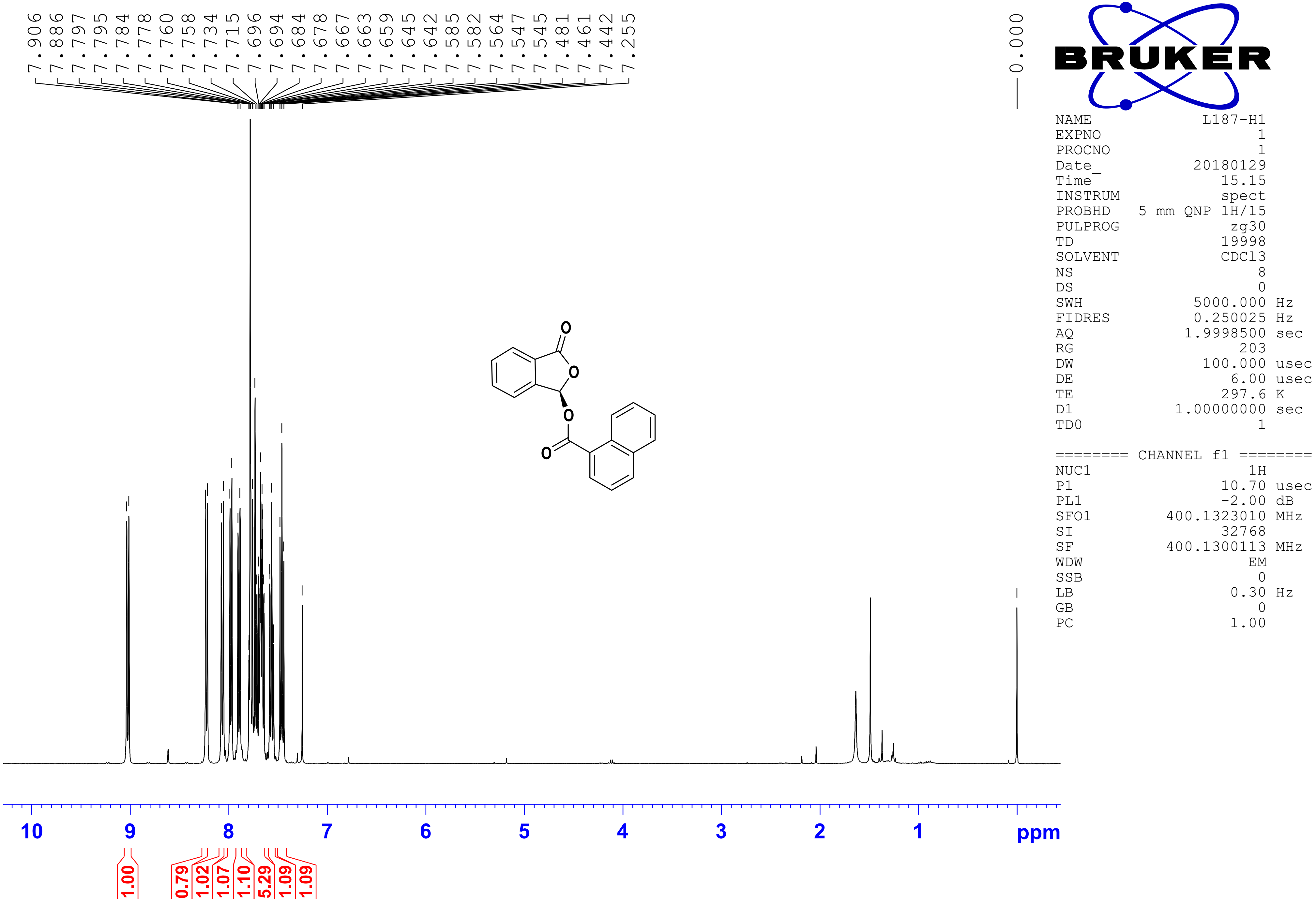

Supplementary Figure 31 ¹H NMR spectrum of **17**

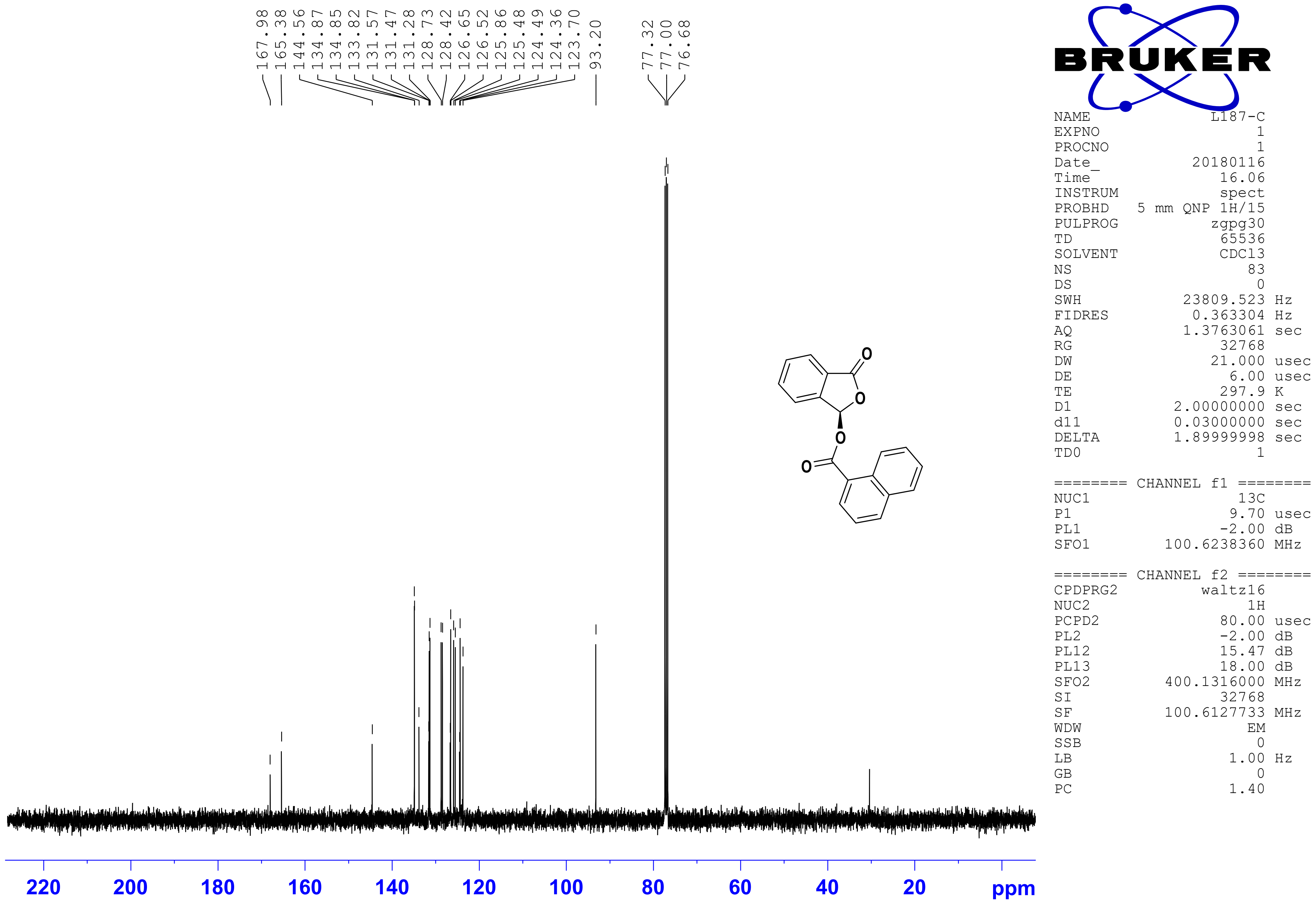

Supplementary Figure 32 ^{13}C NMR spectrum of **17**

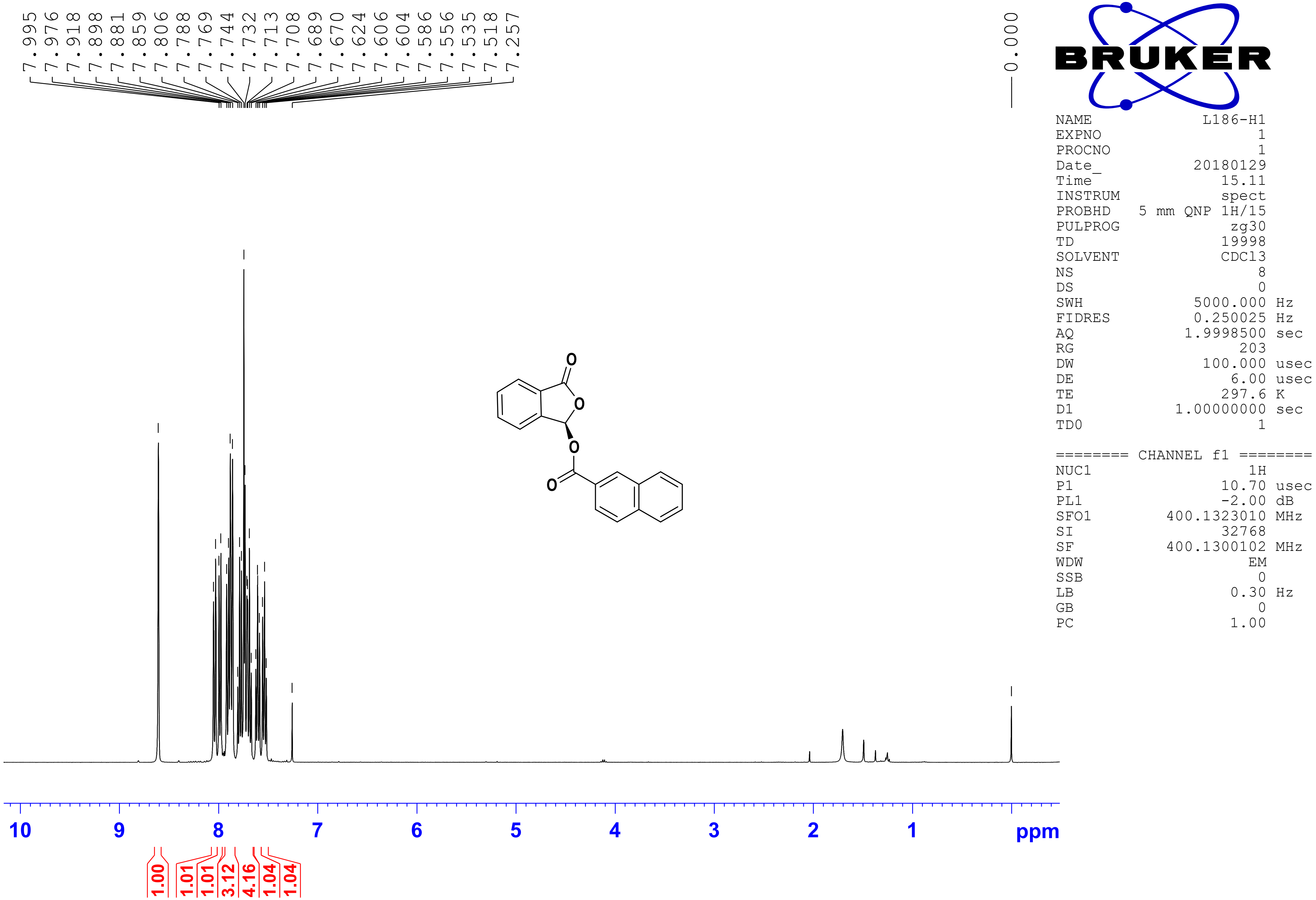

Supplementary Figure 33 ^{1}H NMR spectrum of **18**

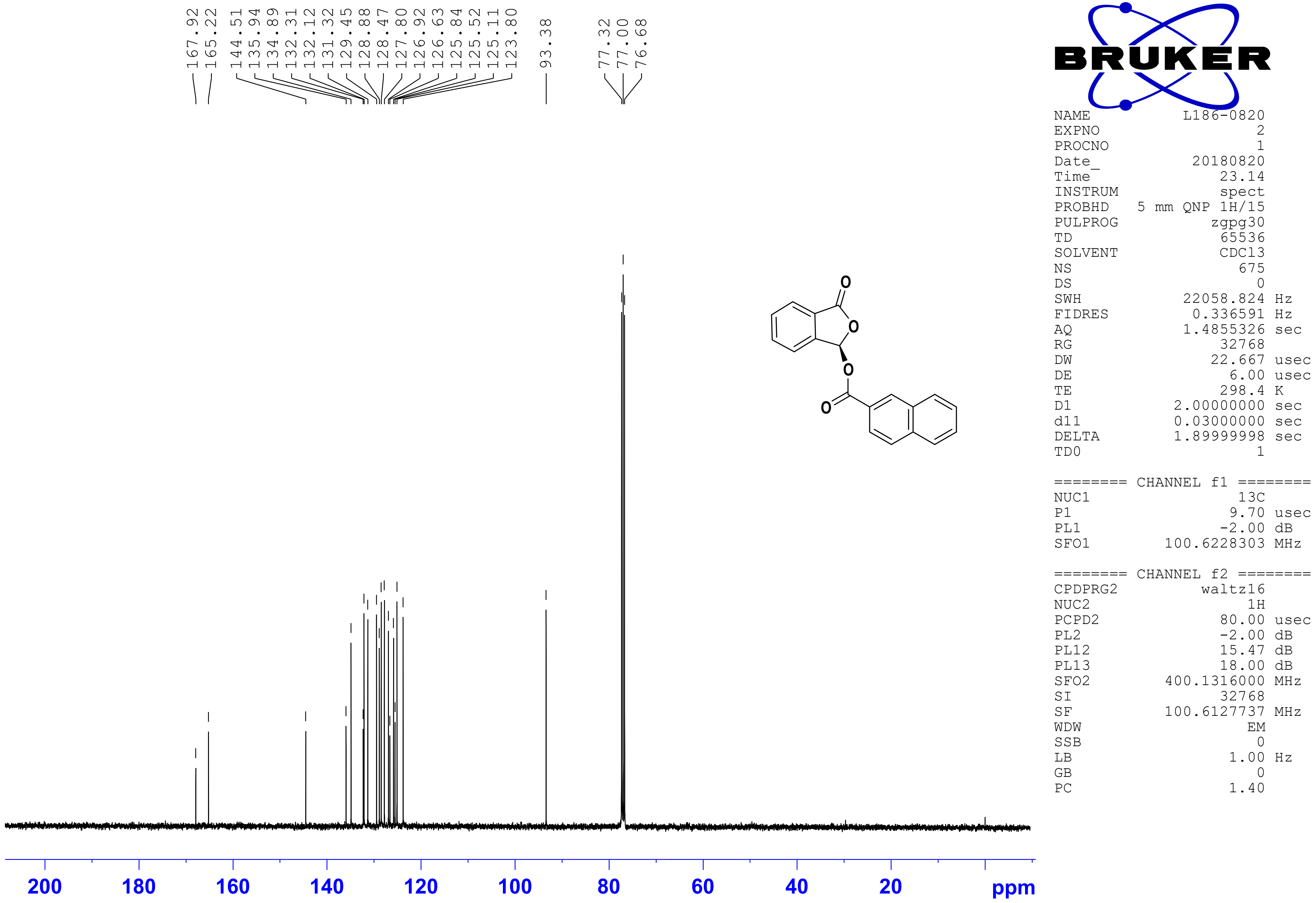

Supplementary Figure 34 ^{13}C NMR spectrum of **18**

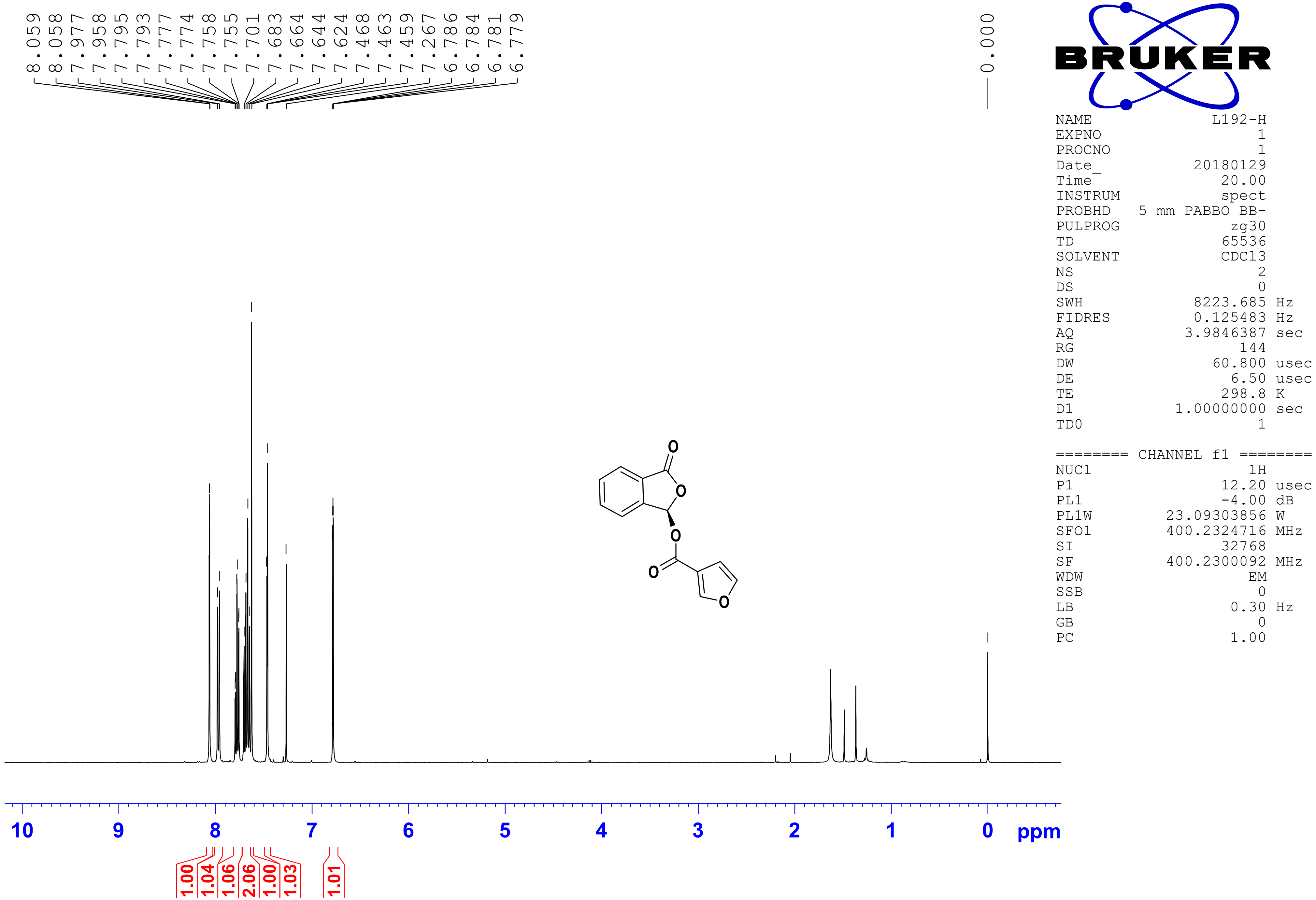

Supplementary Figure 35 ^{1}H NMR spectrum of **19**

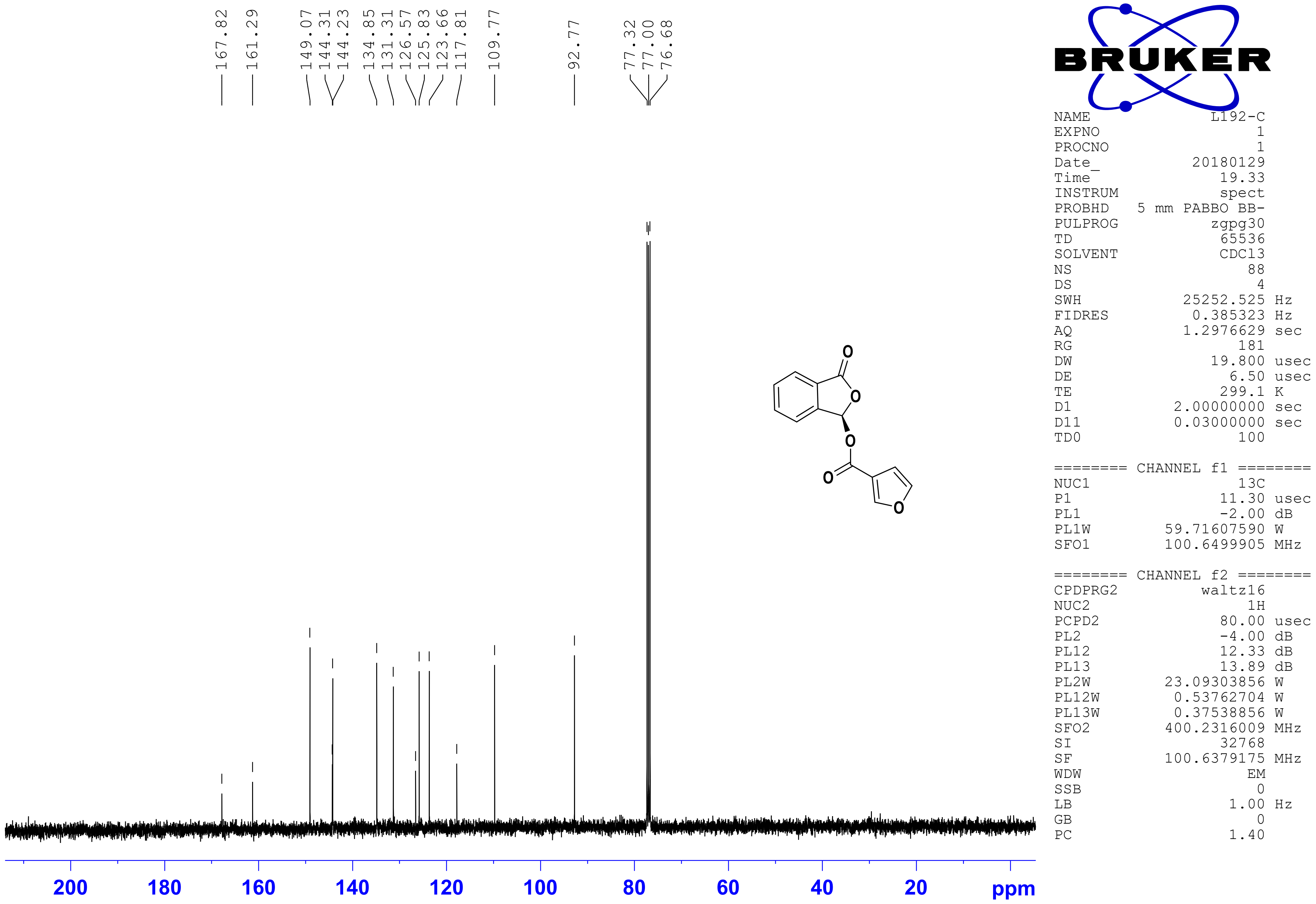

Supplementary Figure 36 ^{13}C NMR spectrum of **19**

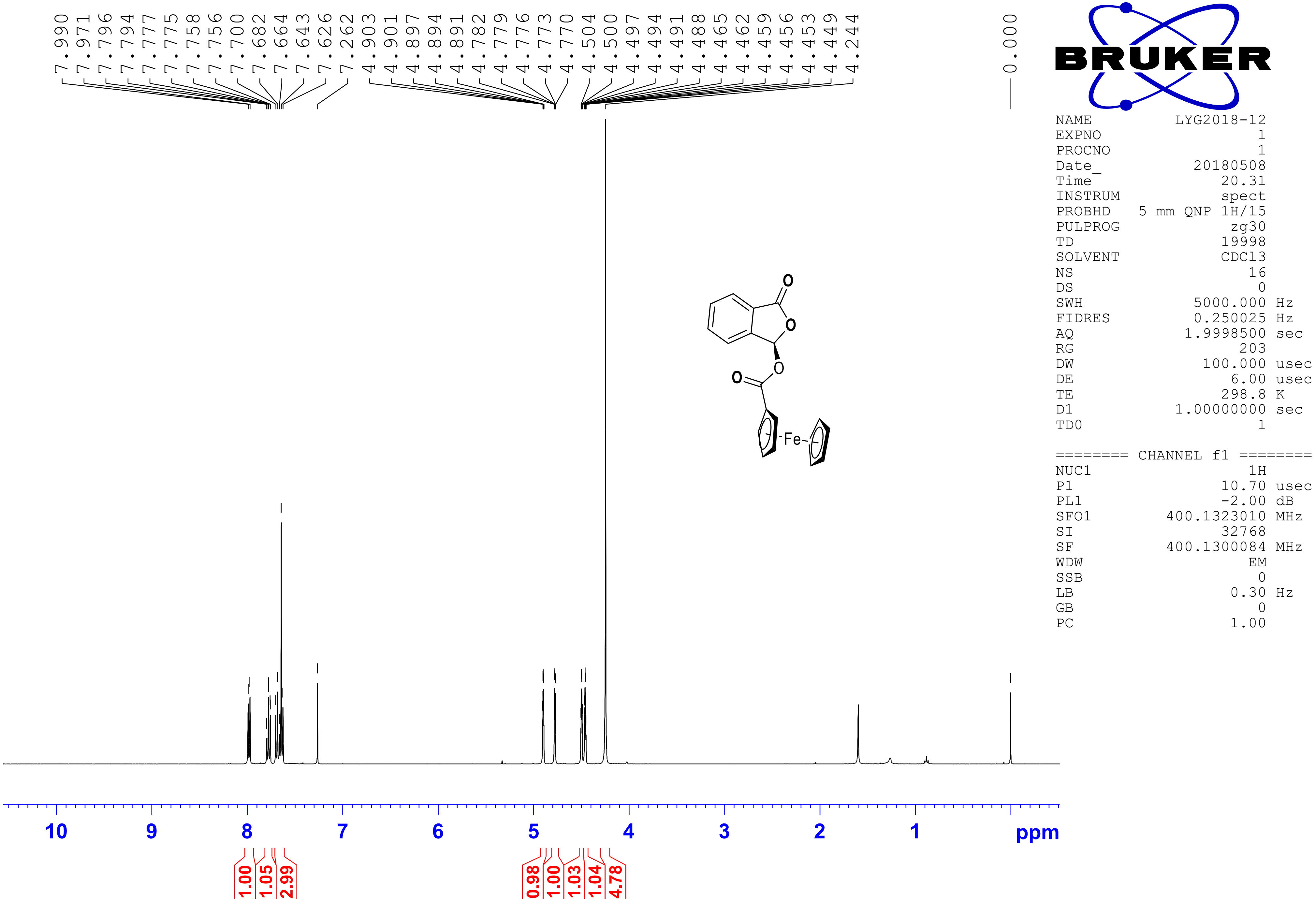

Supplementary Figure 37 ^{1}H NMR spectrum of **20**

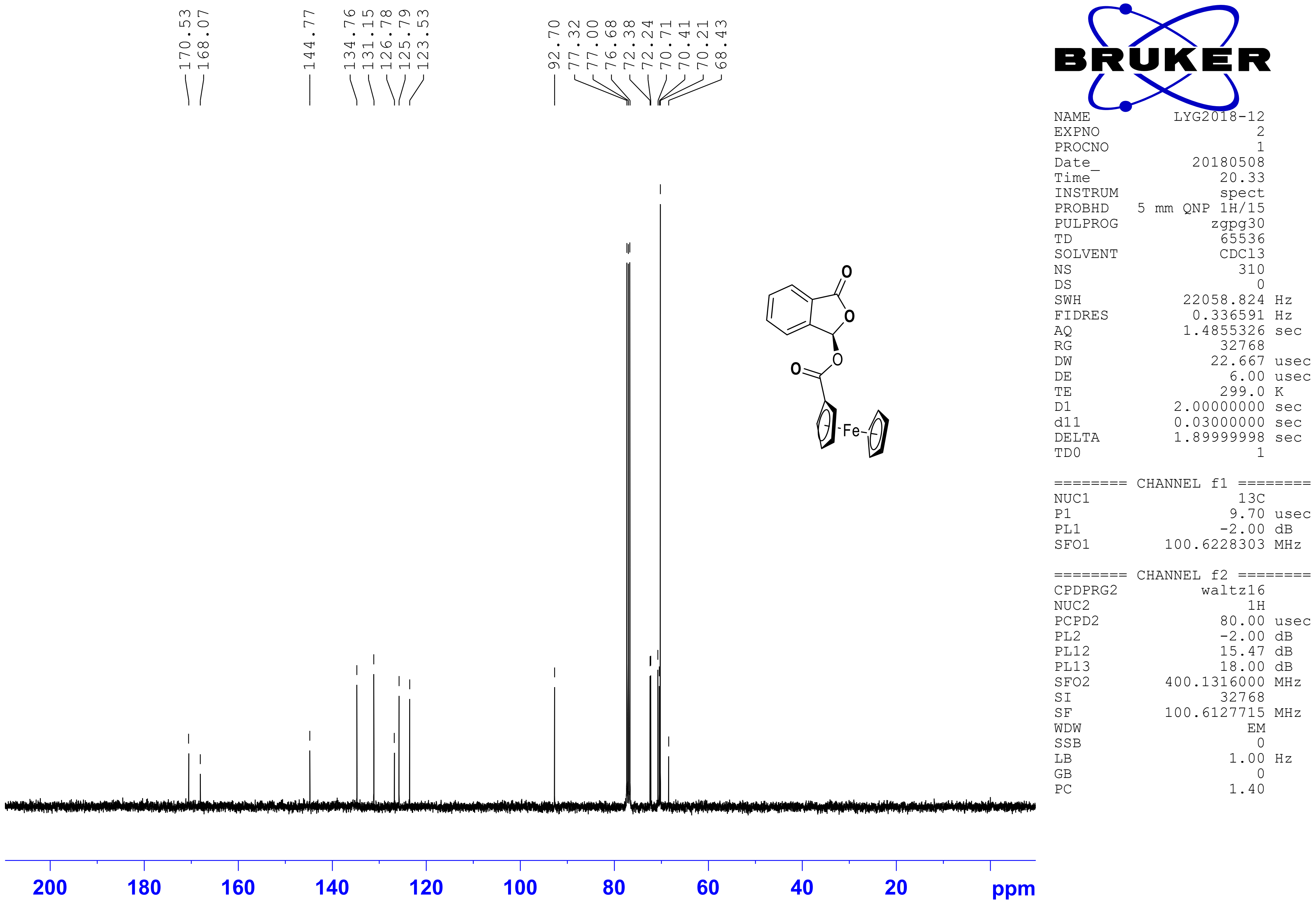

Supplementary Figure 38 ^{13}C NMR spectrum of **20**

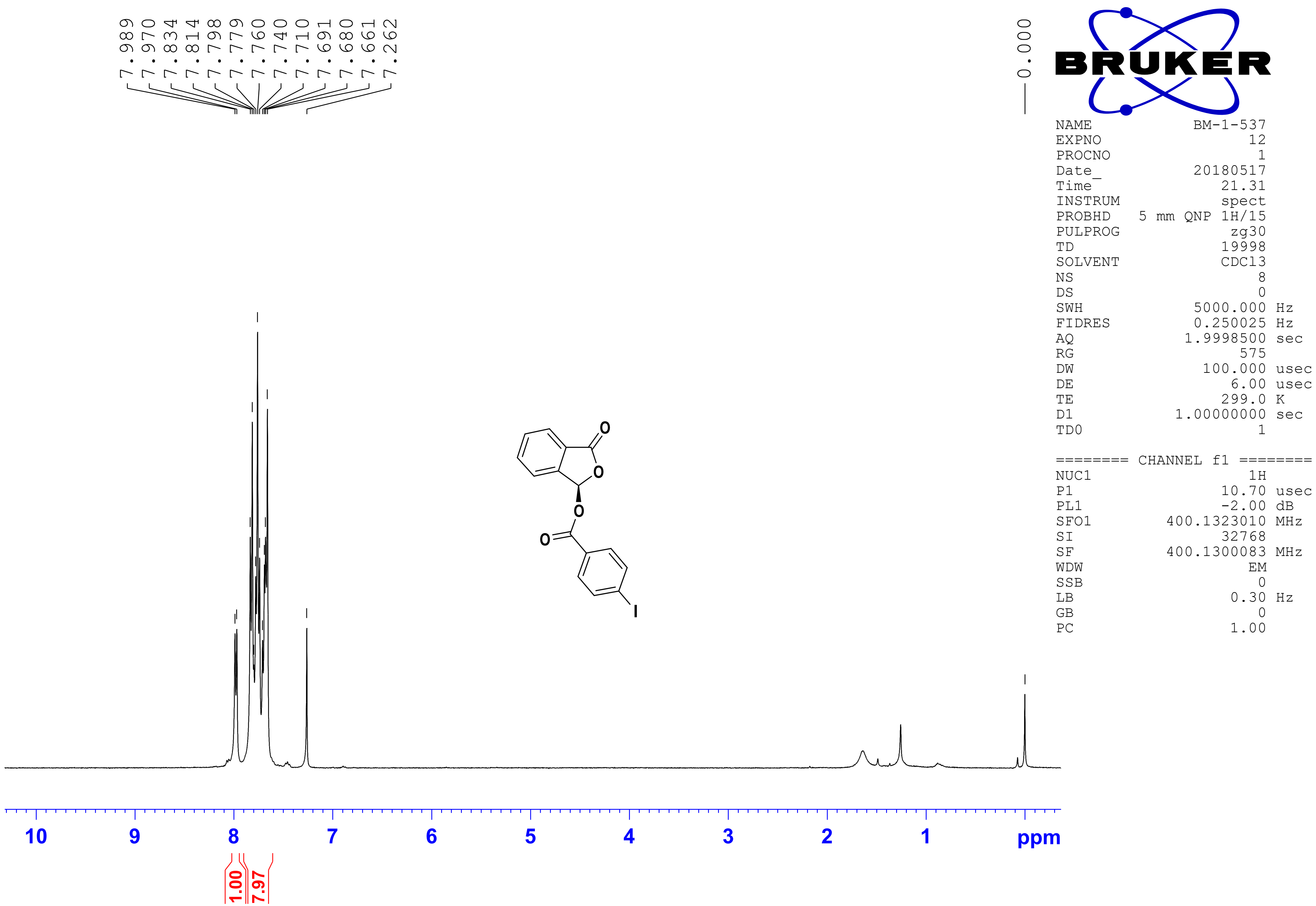

Supplementary Figure 39 ^{1}H NMR spectrum of **21**

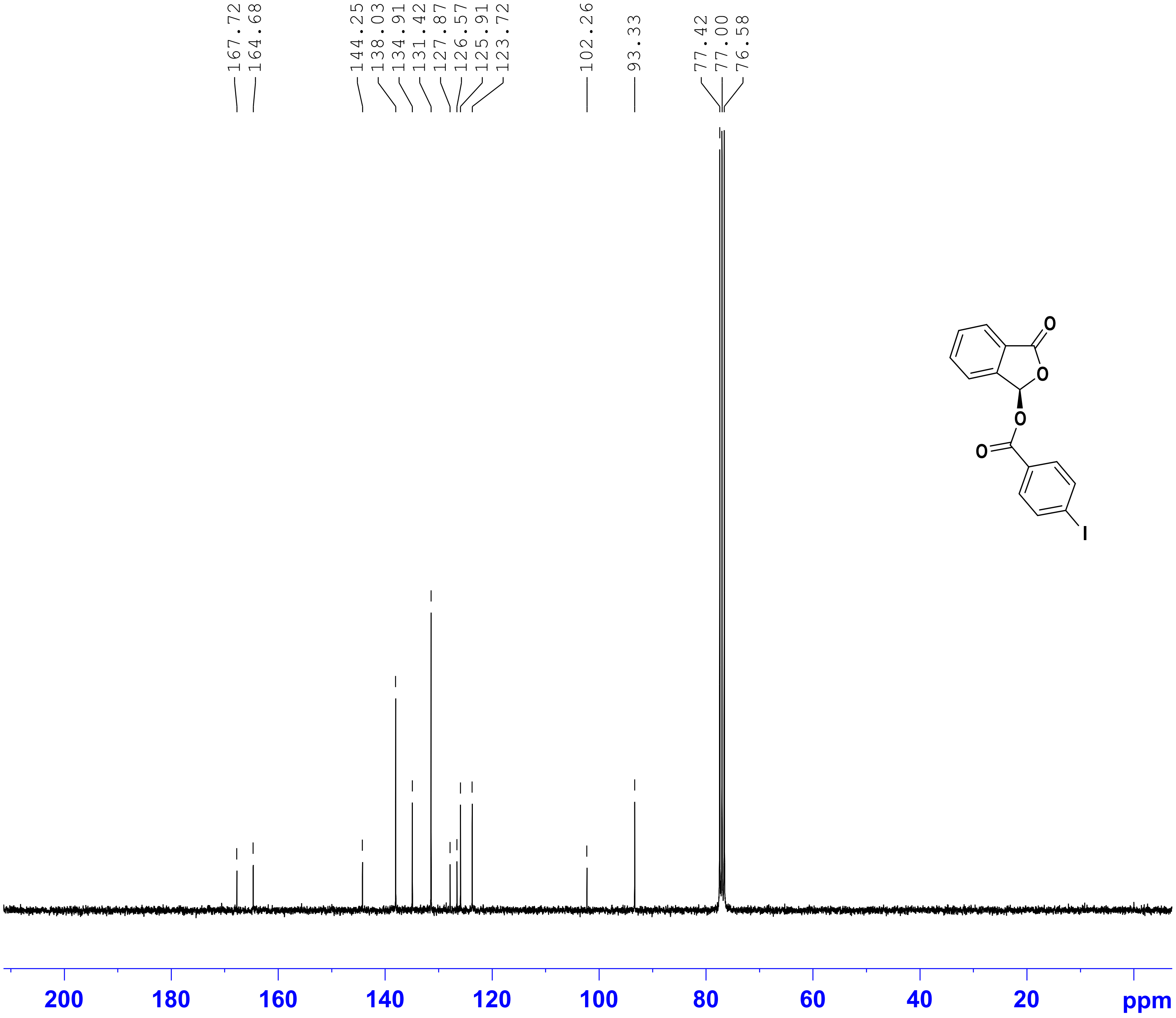

NAME	BM-1-537
EXPNO	2
PROCNO	1
Date_	20180518
Time	21.44
INSTRUM	spect
PROBHD	5 mm PABBO BB-
PULPROG	zgpg30
TD	65536
SOLVENT	CDCl3
NS	1153
DS	0
SWH	18115.941 Hz
FIDRES	0.276427 Hz
AQ	1.8088436 sec
RG	5160.6
DW	27.600 usec
DE	6.00 usec
TE	300.9 K
D1	2.00000000 sec
d11	0.03000000 sec
DELTA	1.89999998 sec
TD0	1
======== CHANNEL f1 ========	
NUC1	13C
P1	8.00 usec
PL1	-4.00 dB
SFO1	75.4752960 MHz
======== CHANNEL f2 ========	
CPDPRG2	waltz16
NUC2	1H
PCPD2	80.00 usec
PL2	-1.00 dB
PL12	15.48 dB
PL13	19.23 dB
SFO2	300.1312000 MHz
SI	32768
SF	75.4677535 MHz
WDW	EM
SSB	0
LB	1.00 Hz
GB	0
PC	1.40

Supplementary Figure 40 ^{13}C NMR spectrum of **21**

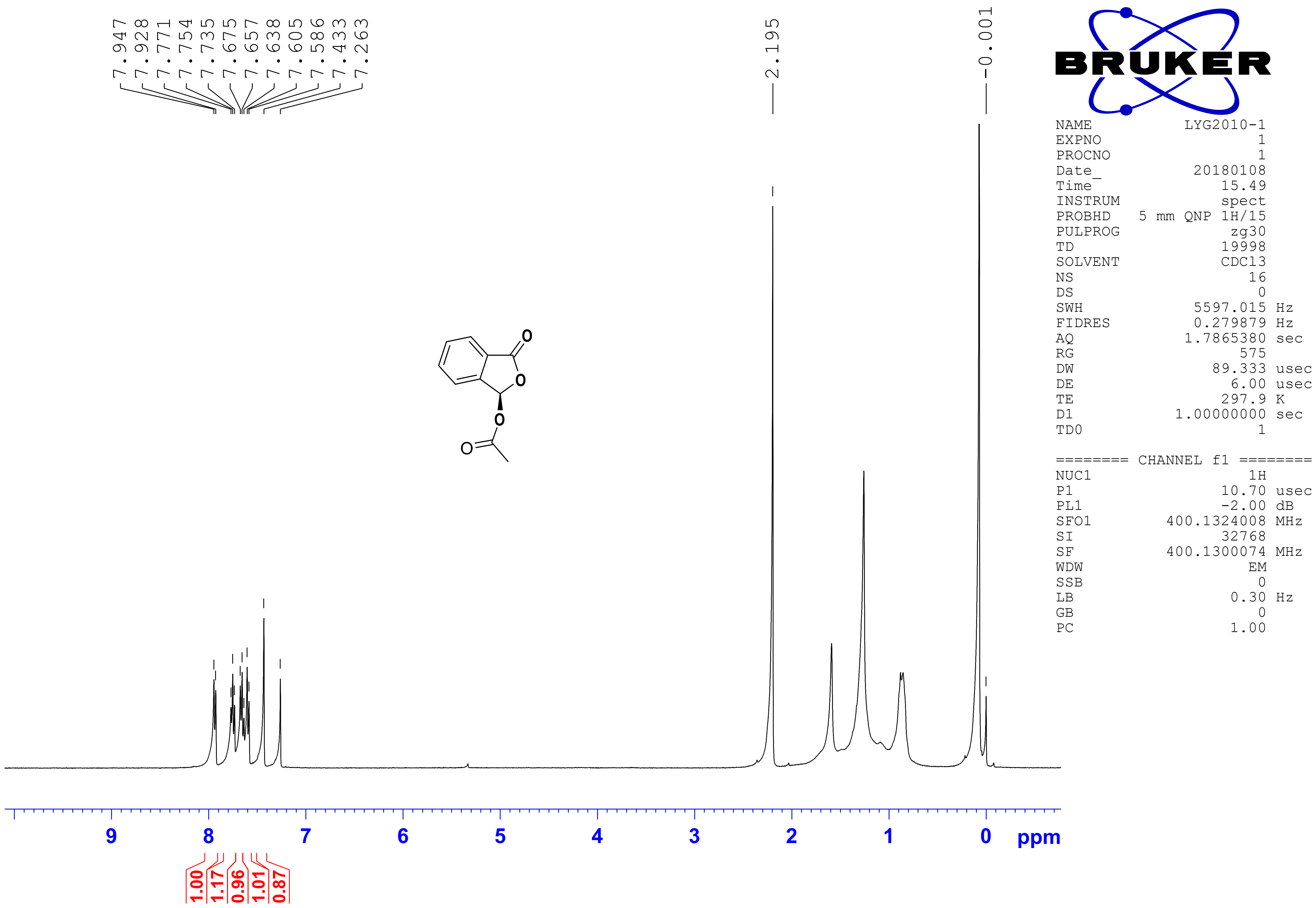

Supplementary Figure 41 ¹H NMR spectrum of **22**

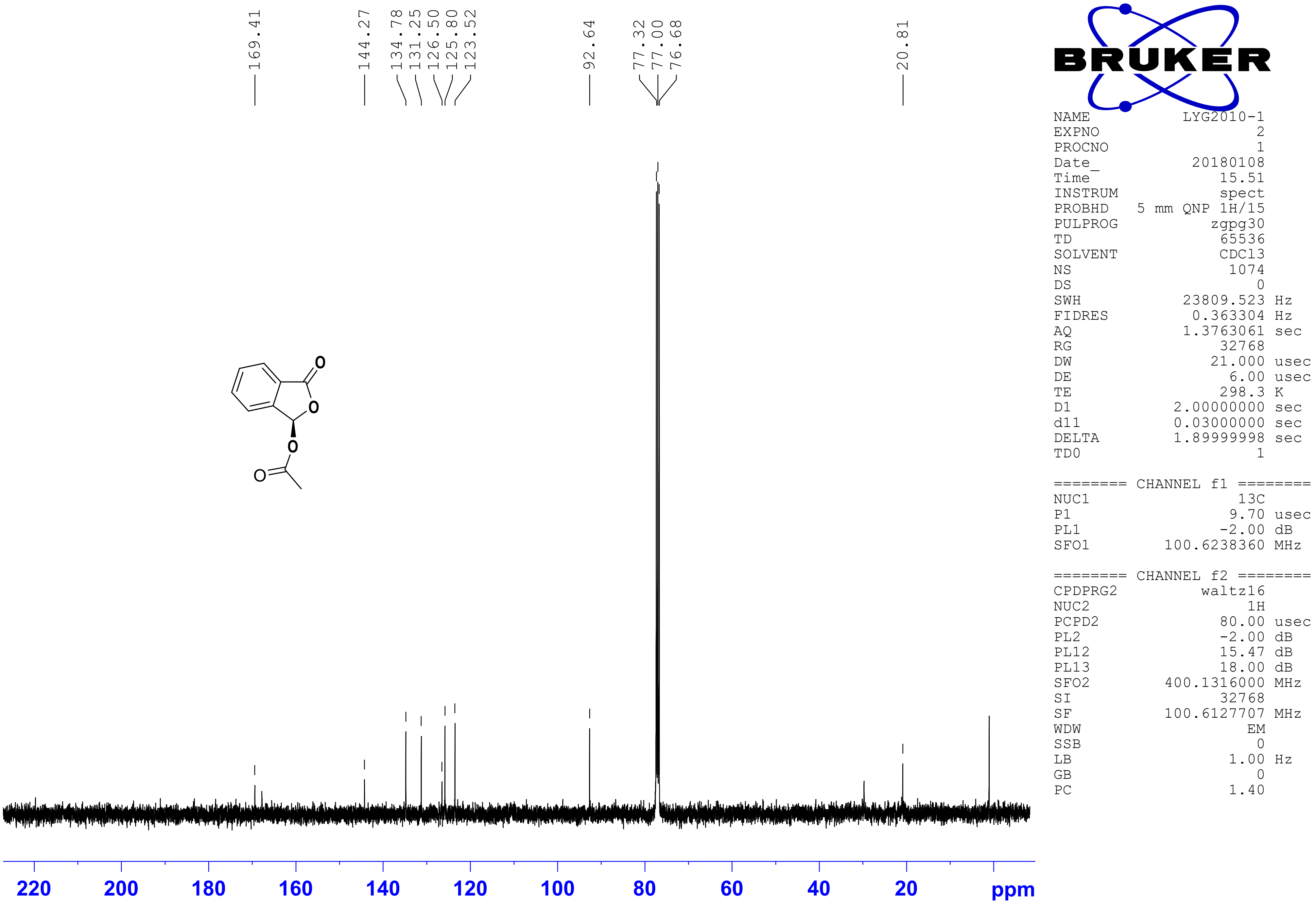

Supplementary Figure 42 ^{13}C NMR spectrum of **22**

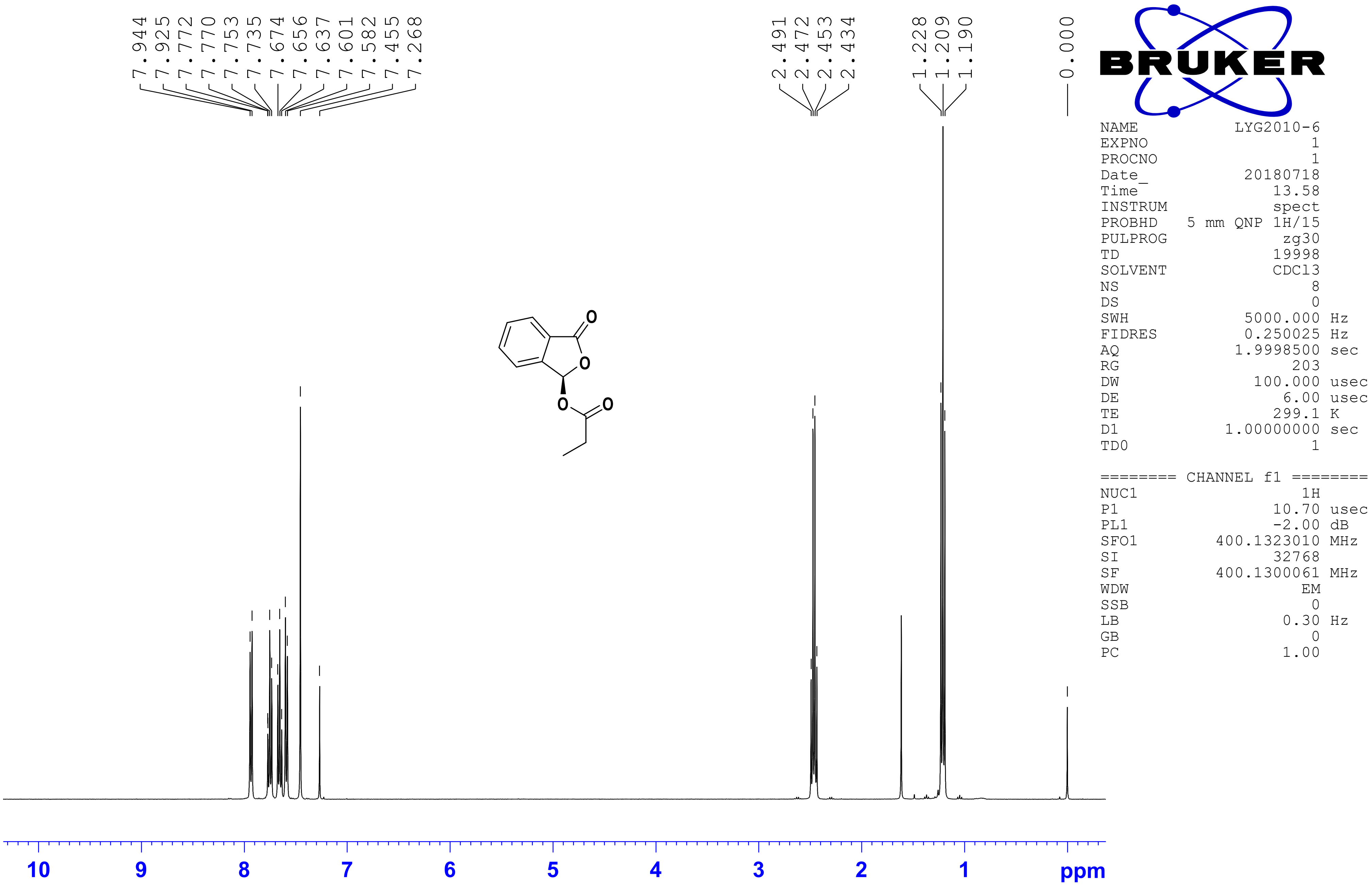

Supplementary Figure 43 ^{1}H NMR spectrum of **23**

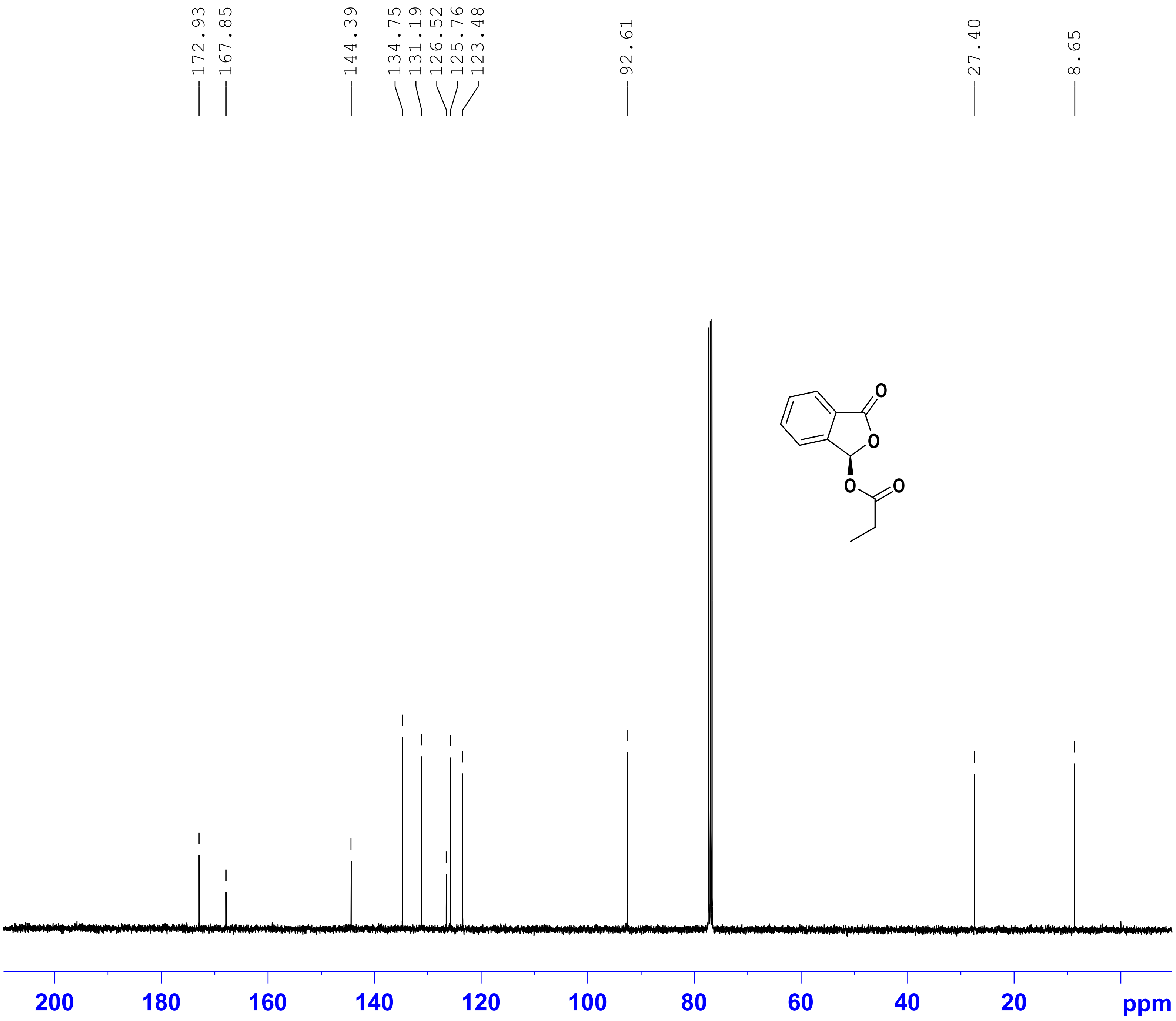

NAME	LYG2010-6
EXPNO	2
PROCNO	1
Date_	20180718
Time	14.00
INSTRUM	spect
PROBHD	5 mm QNP 1H/15
PULPROG	zgpg30
TD	65536
SOLVENT	CDCl3
NS	369
DS	0
SWH	22058.824 Hz
FIDRES	0.336591 Hz
AQ	1.4855326 sec
RG	32768
DW	22.667 usec
DE	6.00 usec
TE	299.5 K
D1	2.00000000 sec
d11	0.03000000 sec
DELTA	1.89999998 sec
TD0	1

======== CHANNEL f1 ========

NUC1	13C
P1	9.70 usec
PL1	-2.00 dB
SFO1	100.6228303 MHz

======== CHANNEL f2 ========

CPDPRG2	waltz16
NUC2	1H
PCPD2	80.00 usec
PL2	-2.00 dB
PL12	15.47 dB
PL13	18.00 dB
SFO2	400.1316000 MHz
SI	32768
SF	100.6127712 MHz
WDW	EM
SSB	0
LB	1.00 Hz
GB	0
PC	1.40

Supplementary Figure 44 ^{13}C NMR spectrum of **23**

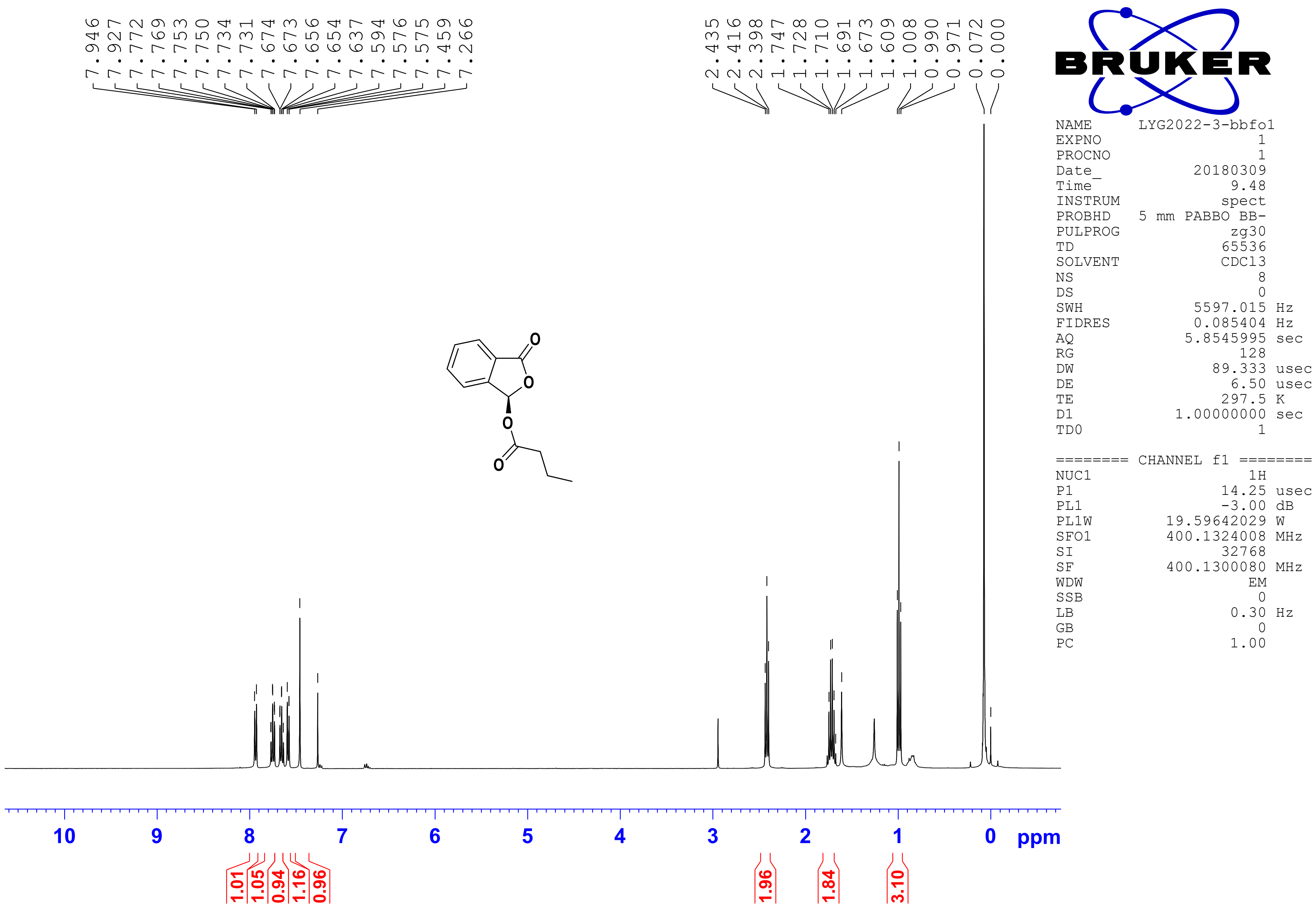

Supplementary Figure 45 ^{1}H NMR spectrum of **24**

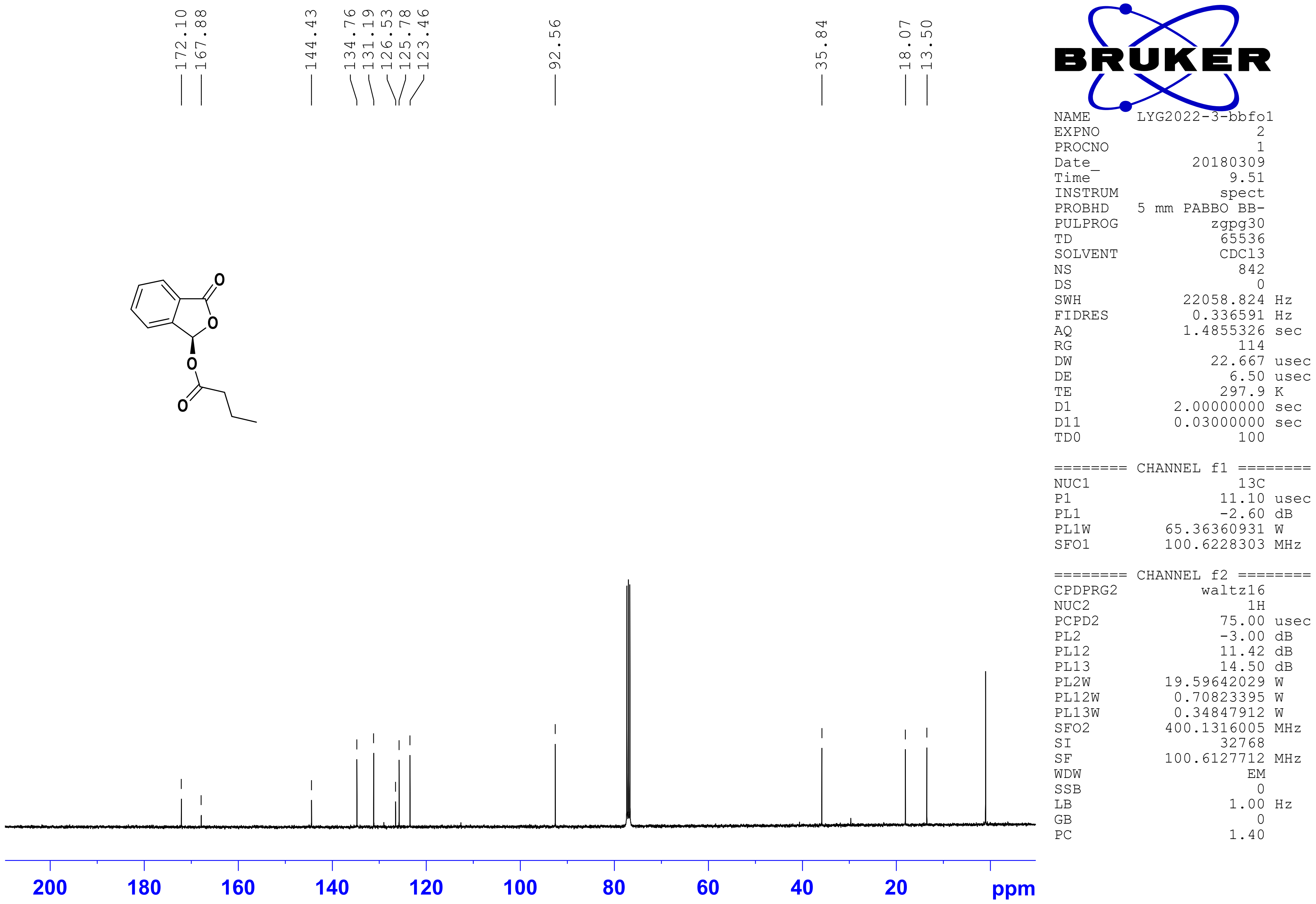

Supplementary Figure 46 ^{13}C NMR spectrum of **24**

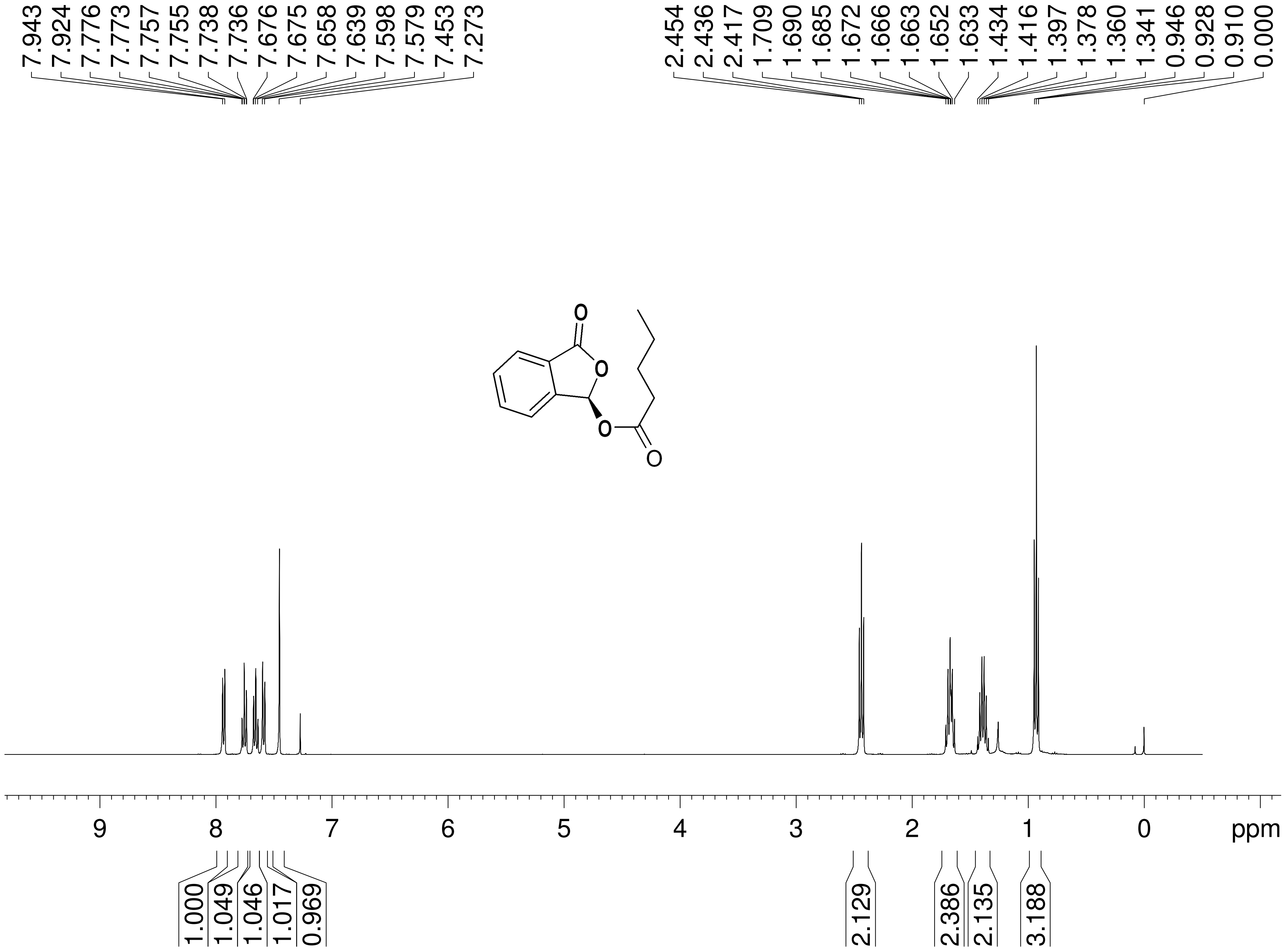

Supplementary Figure 47 ^{1}H NMR spectrum of **25**

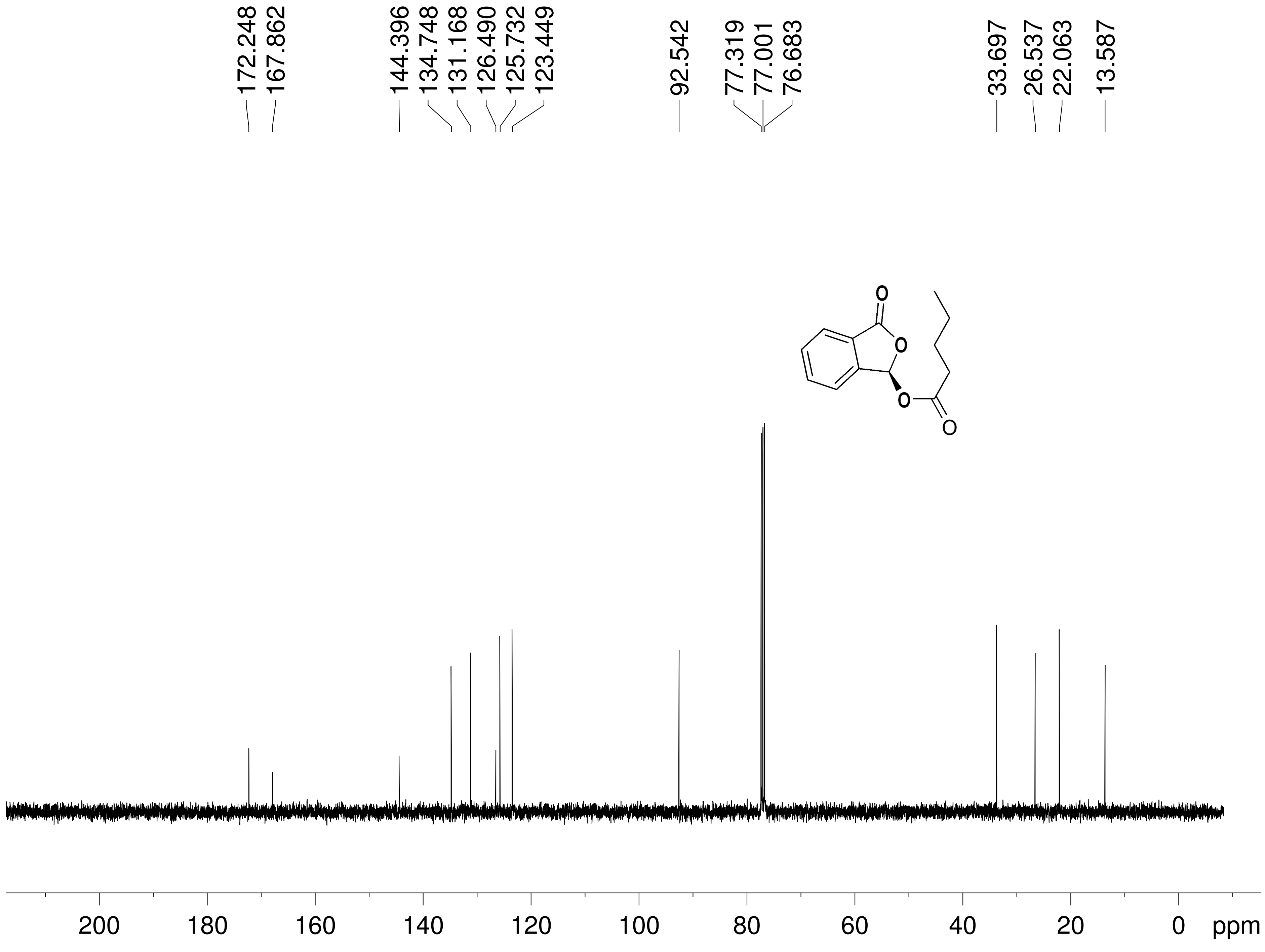

Supplementary Figure 48 ^{13}C NMR spectrum of **25**

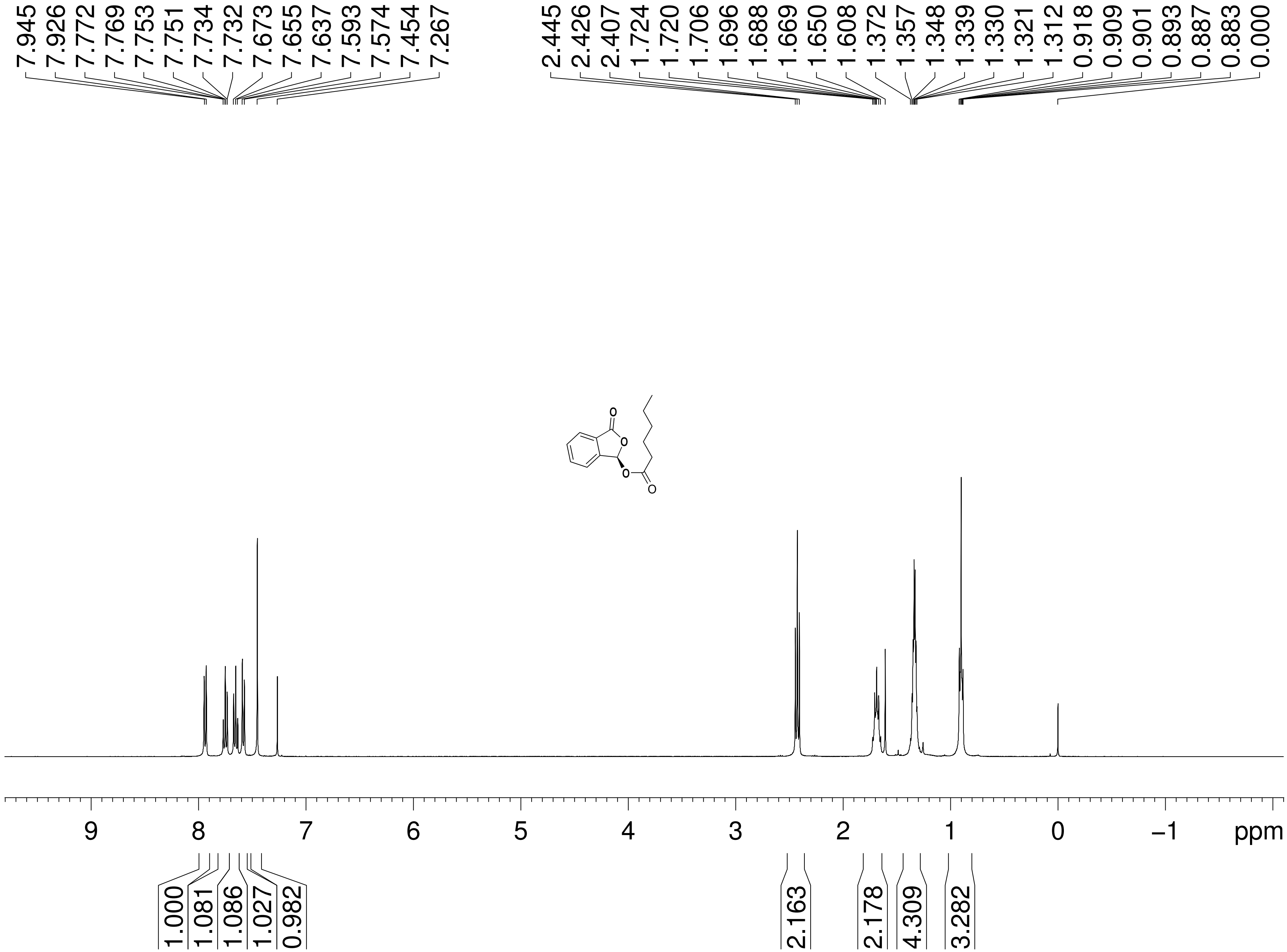

Supplementary Figure 49 ^{1}H NMR spectrum of **26**

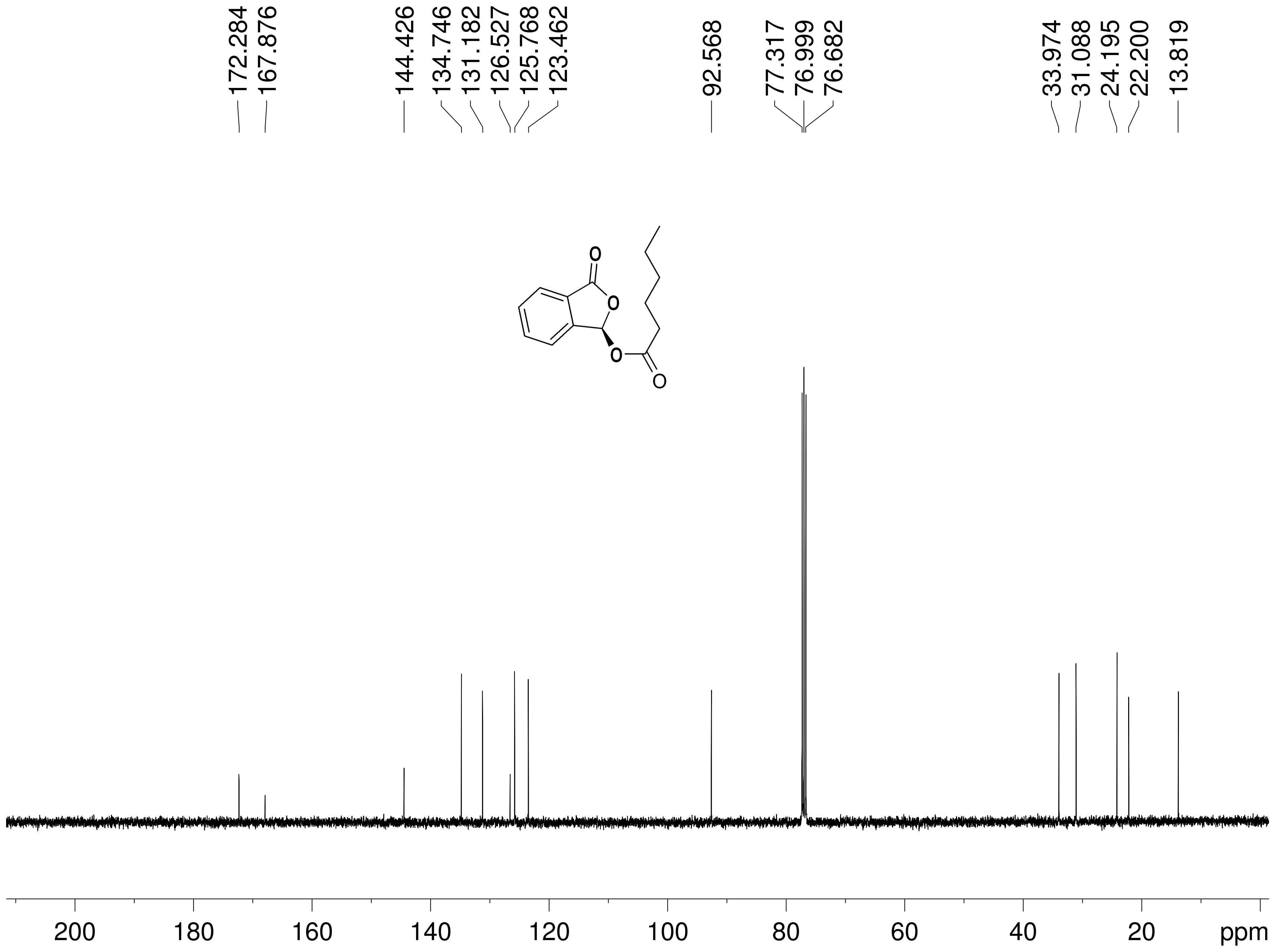

Supplementary Figure 50 ^{13}C NMR spectrum of **26**

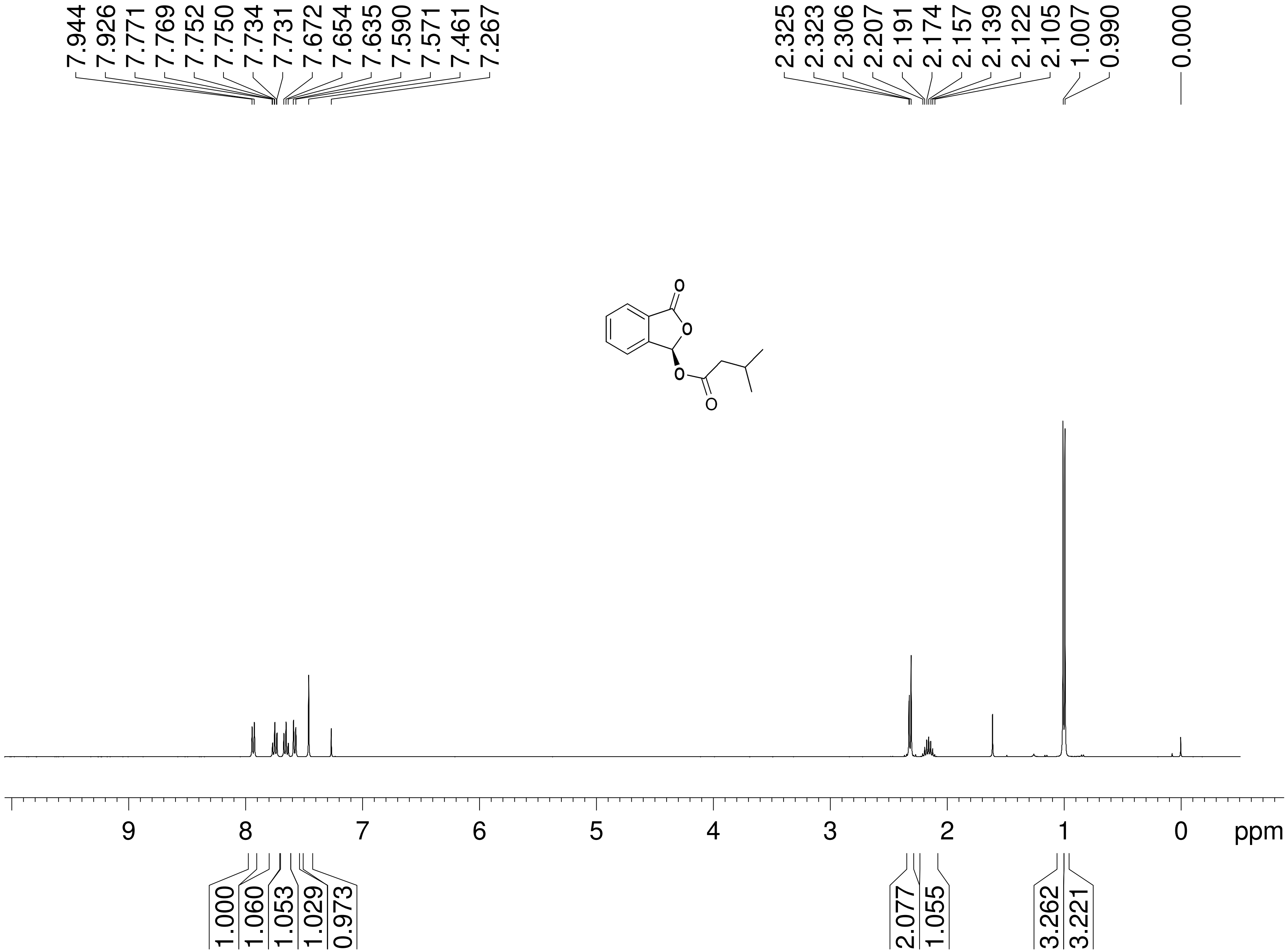

Supplementary Figure 51 ^{1}H NMR spectrum of **27**

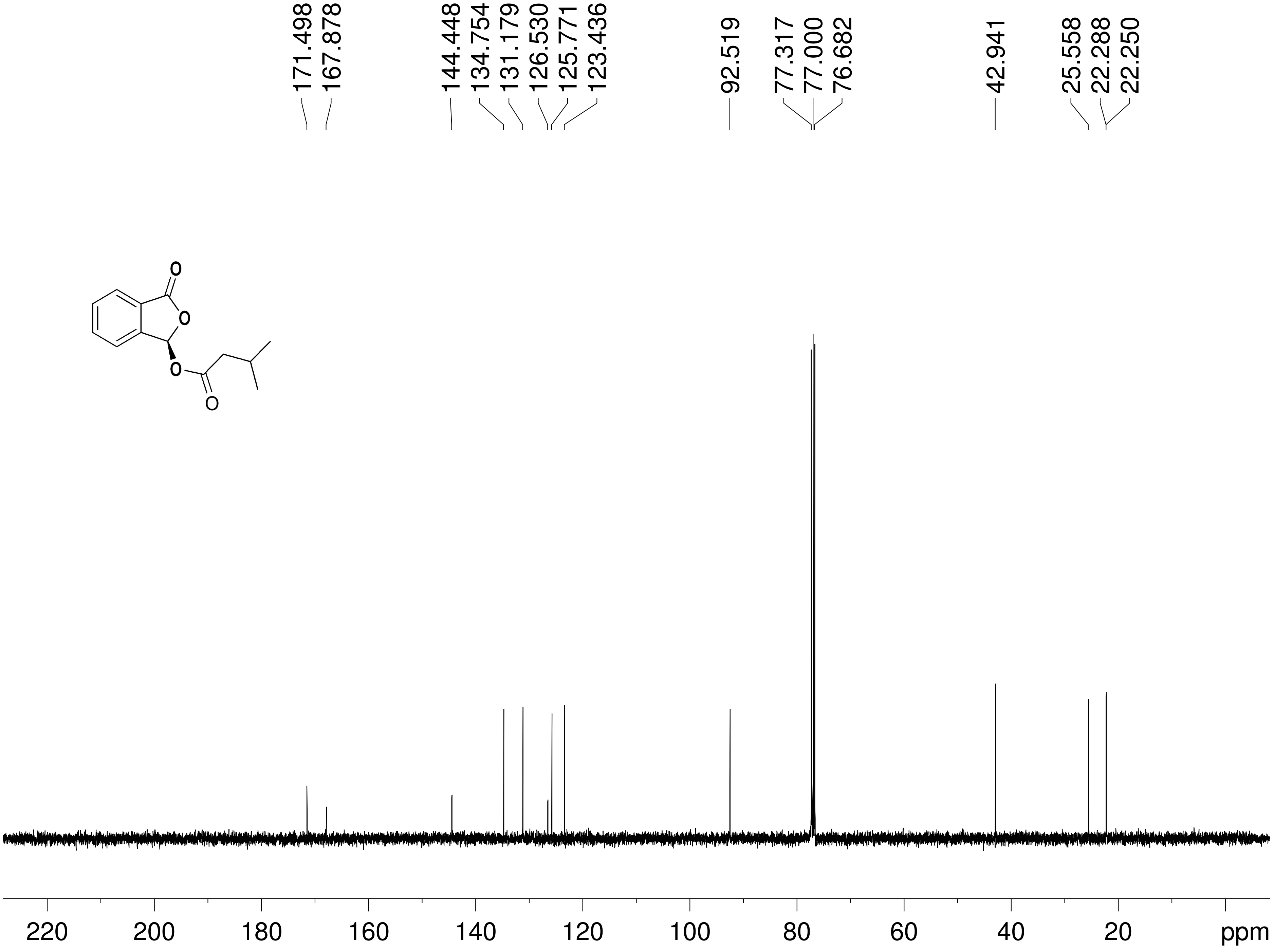

Supplementary Figure 52 ^{13}C NMR spectrum of **27**

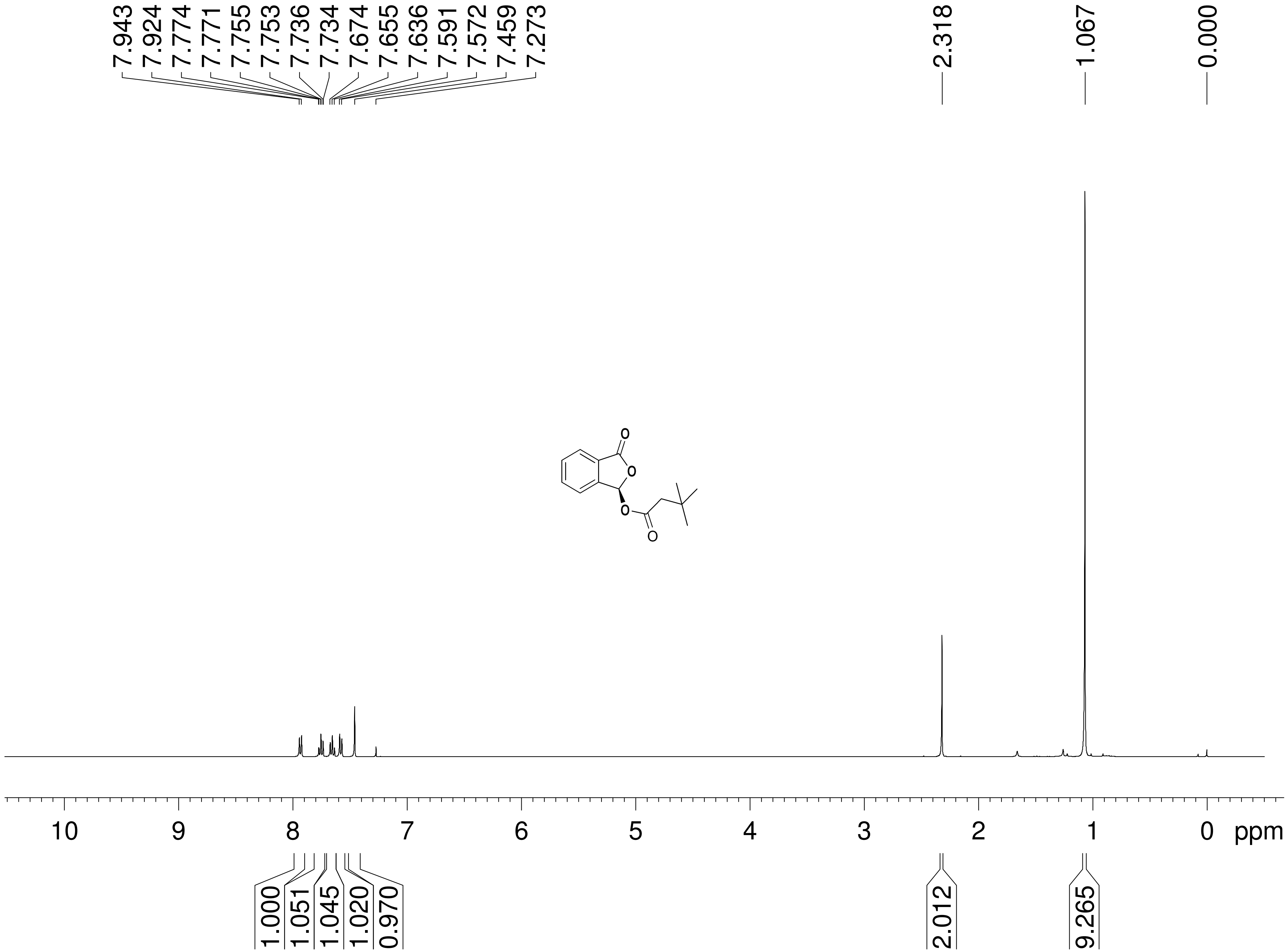

Supplementary Figure 53 ^{1}H NMR spectrum of **28**

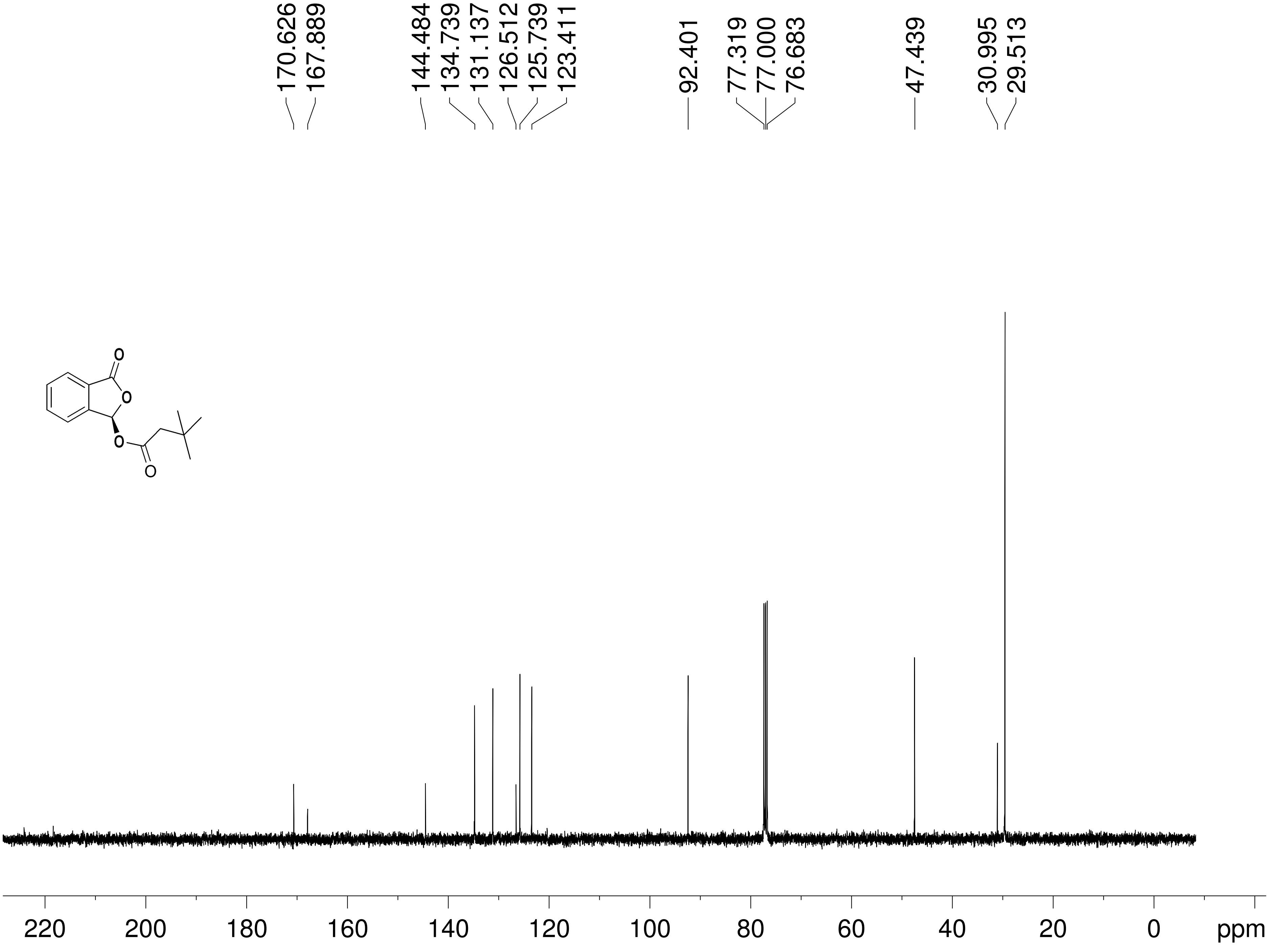

Supplementary Figure 54 ^{13}C NMR spectrum of **28**

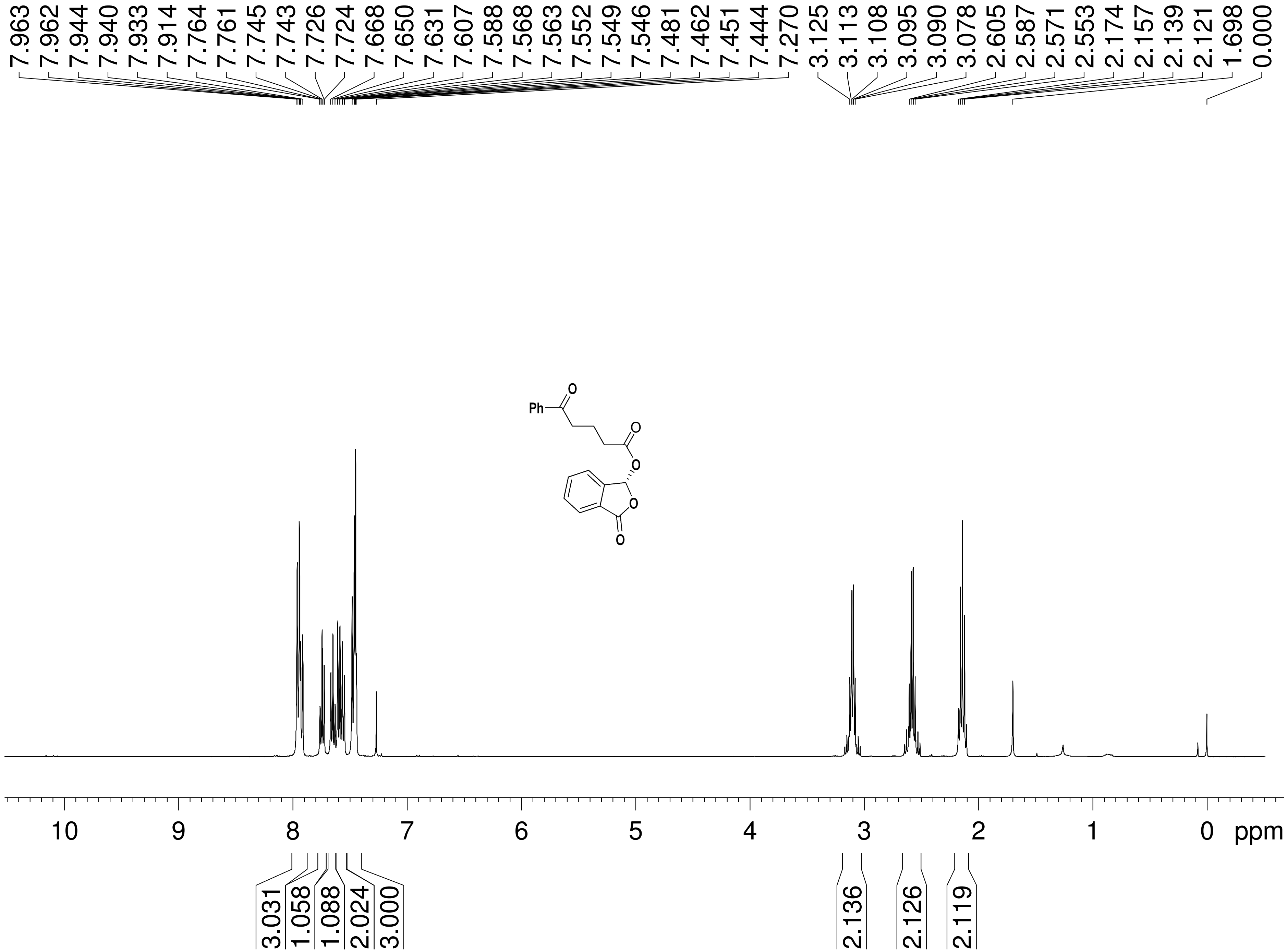

Supplementary Figure 55 ^{1}H NMR spectrum of **29**

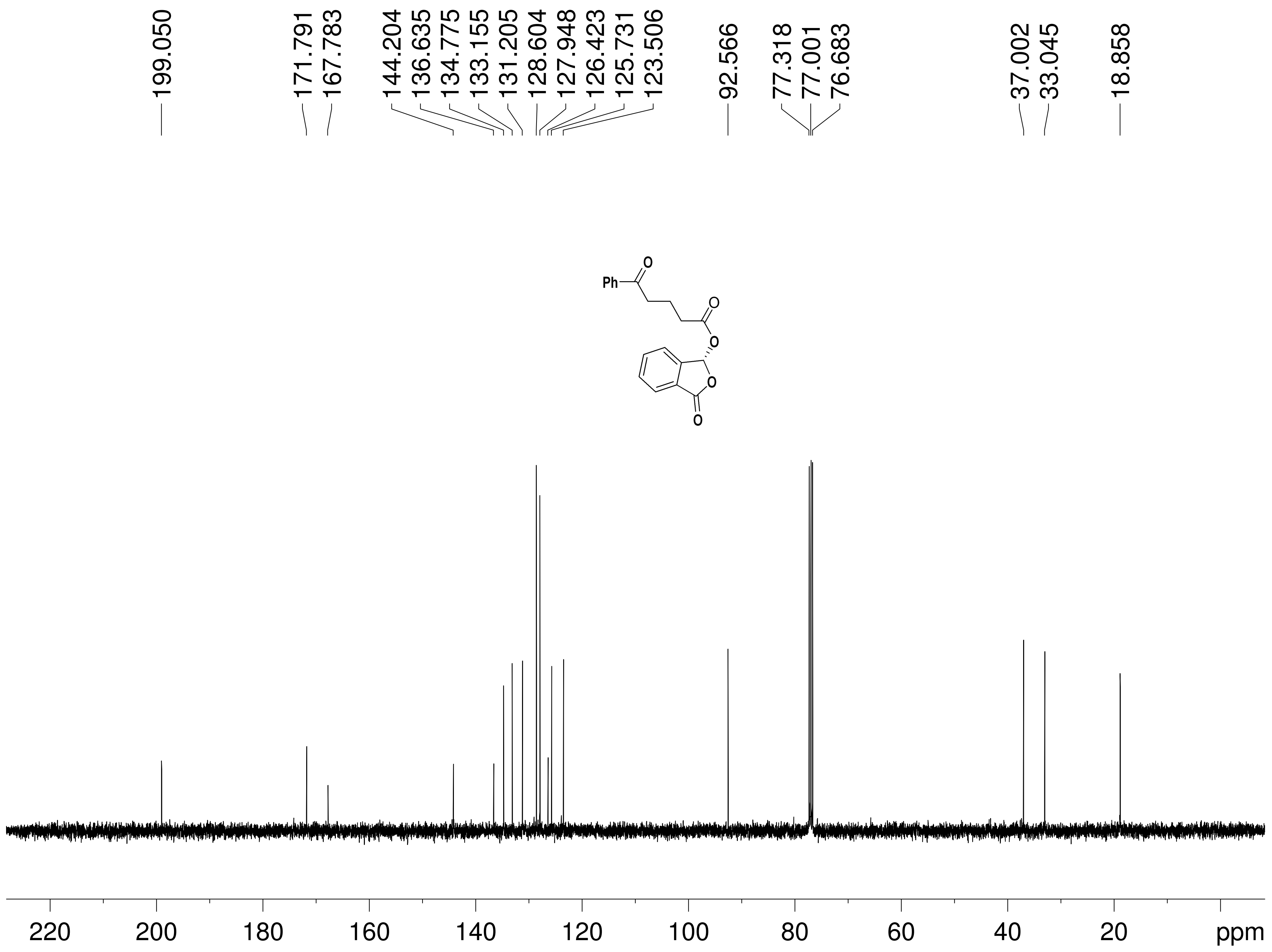

Supplementary Figure 56 ^{13}C NMR spectrum of **29**

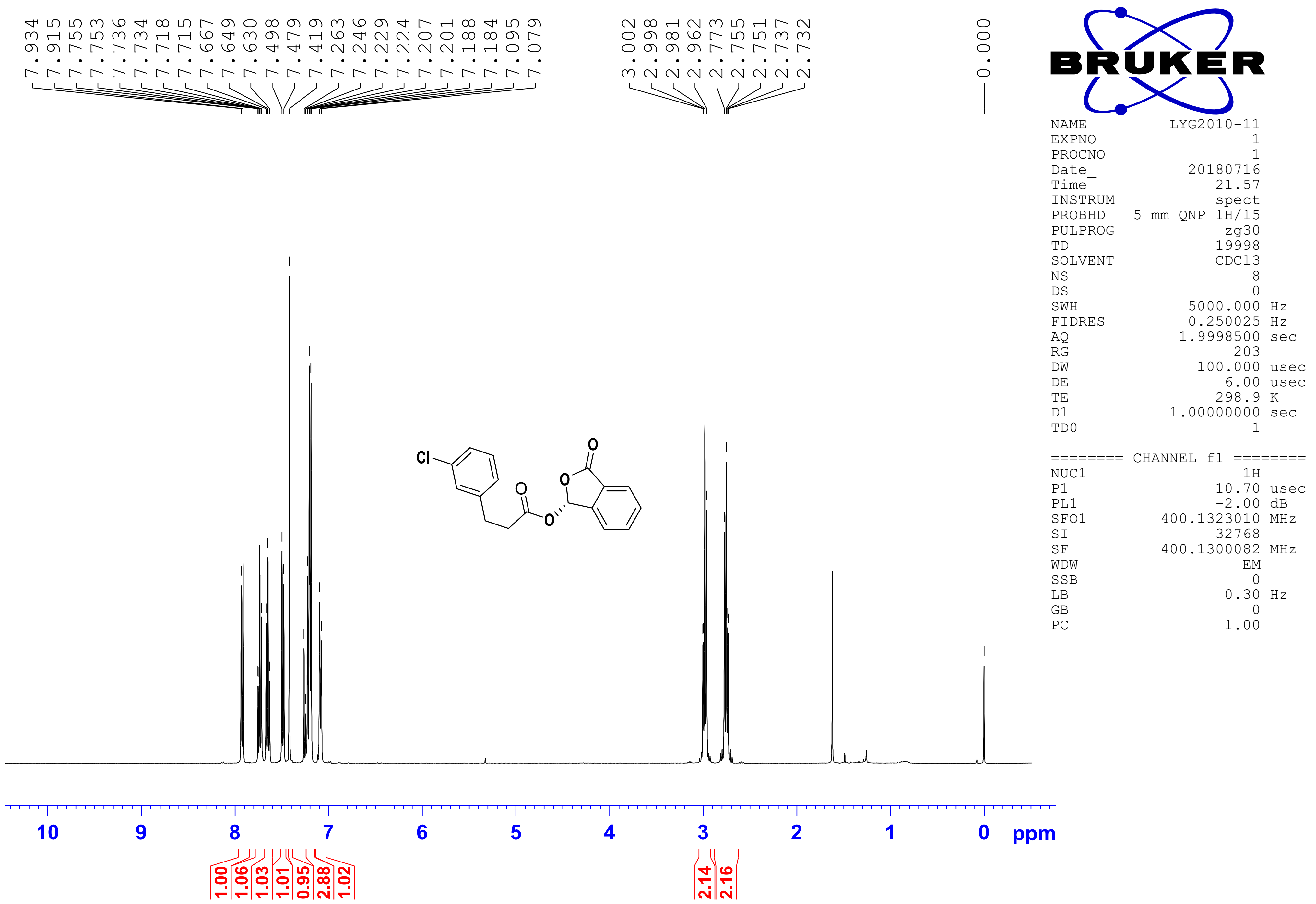

Supplementary Figure 57 ^{1}H NMR spectrum of **30**

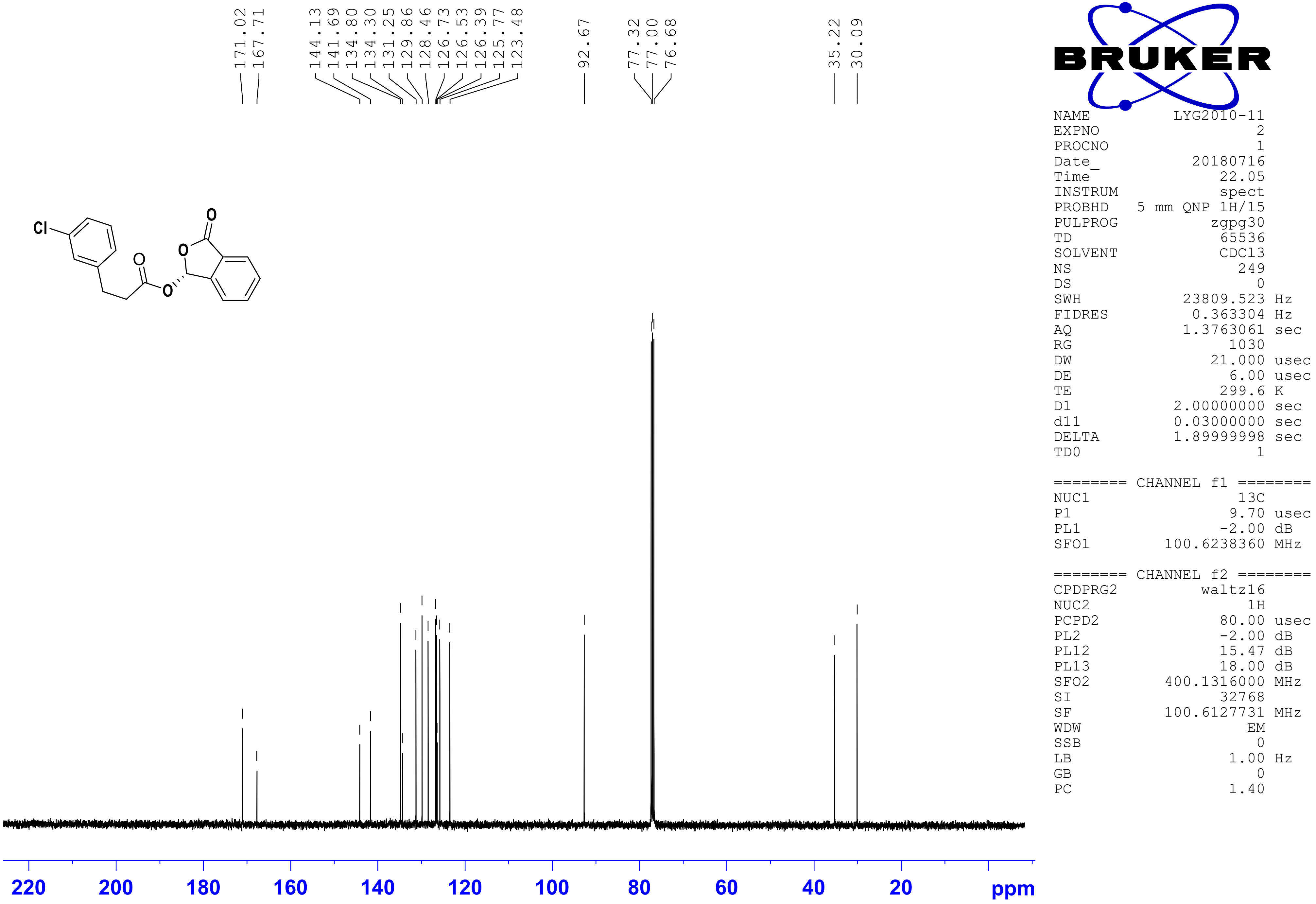

Supplementary Figure 58 ^{13}C NMR spectrum of **30**

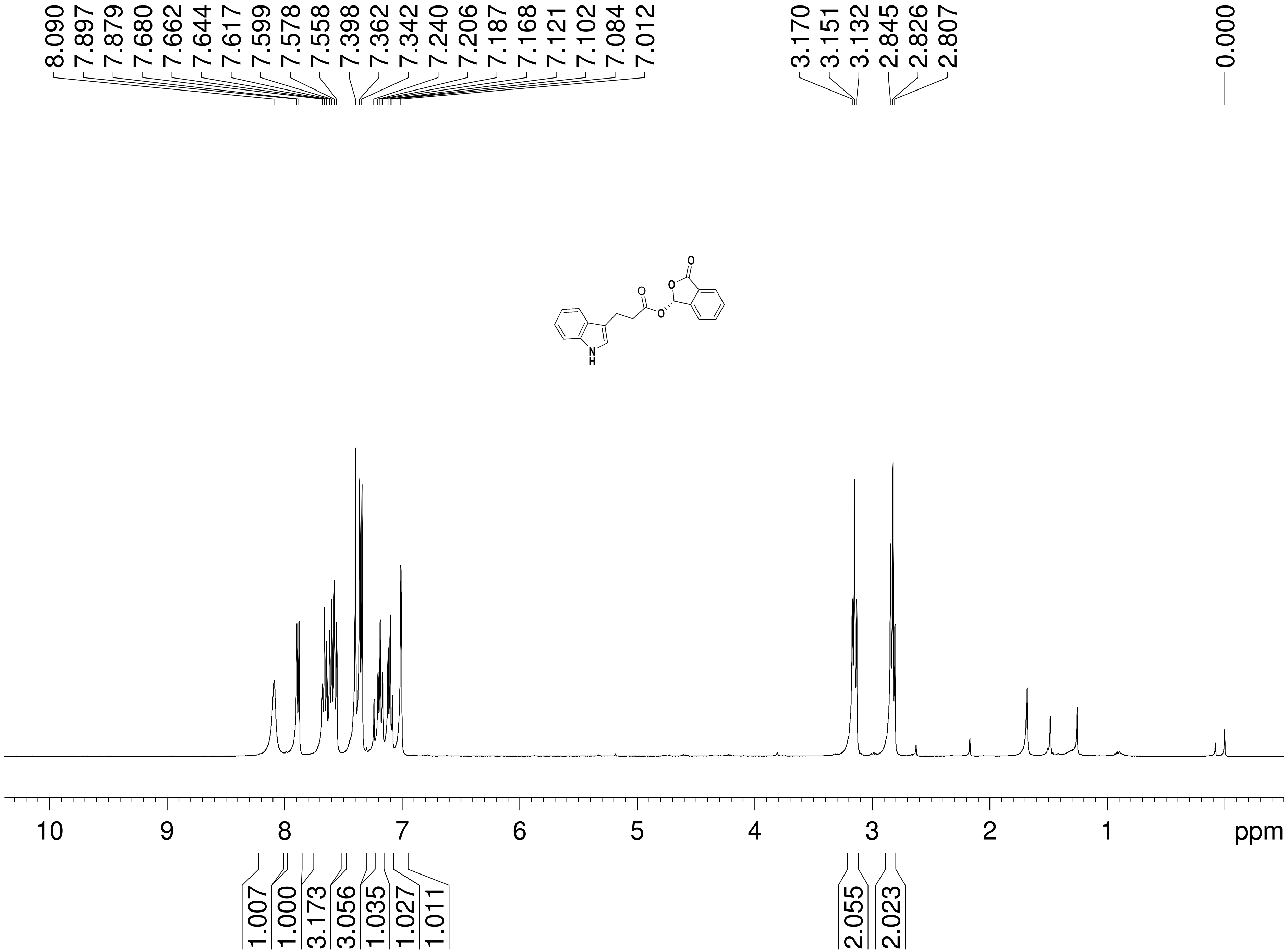

Supplementary Figure 59 ^{1}H NMR spectrum of **31**

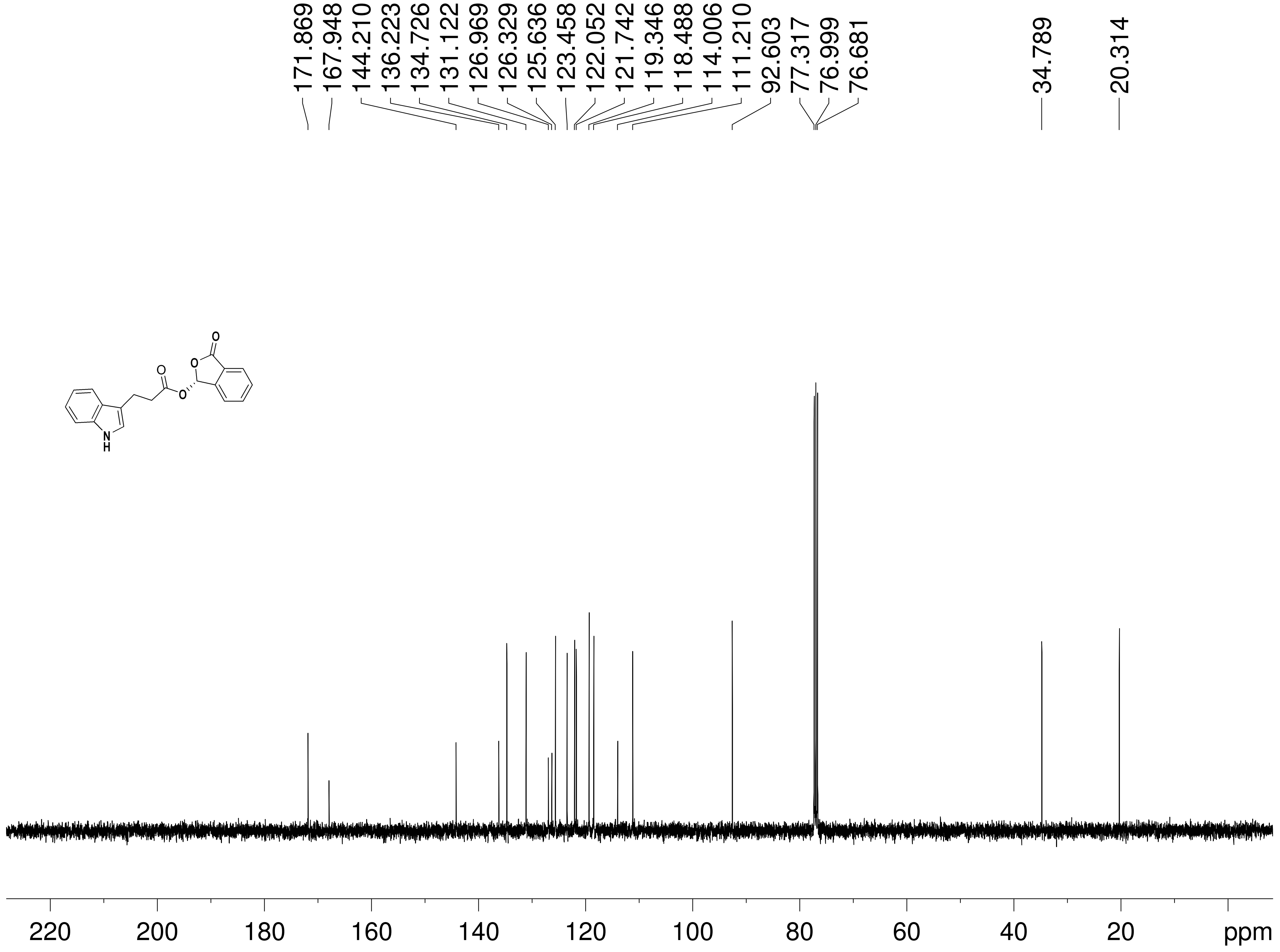

Supplementary Figure 60 ^{13}C NMR spectrum of **31**

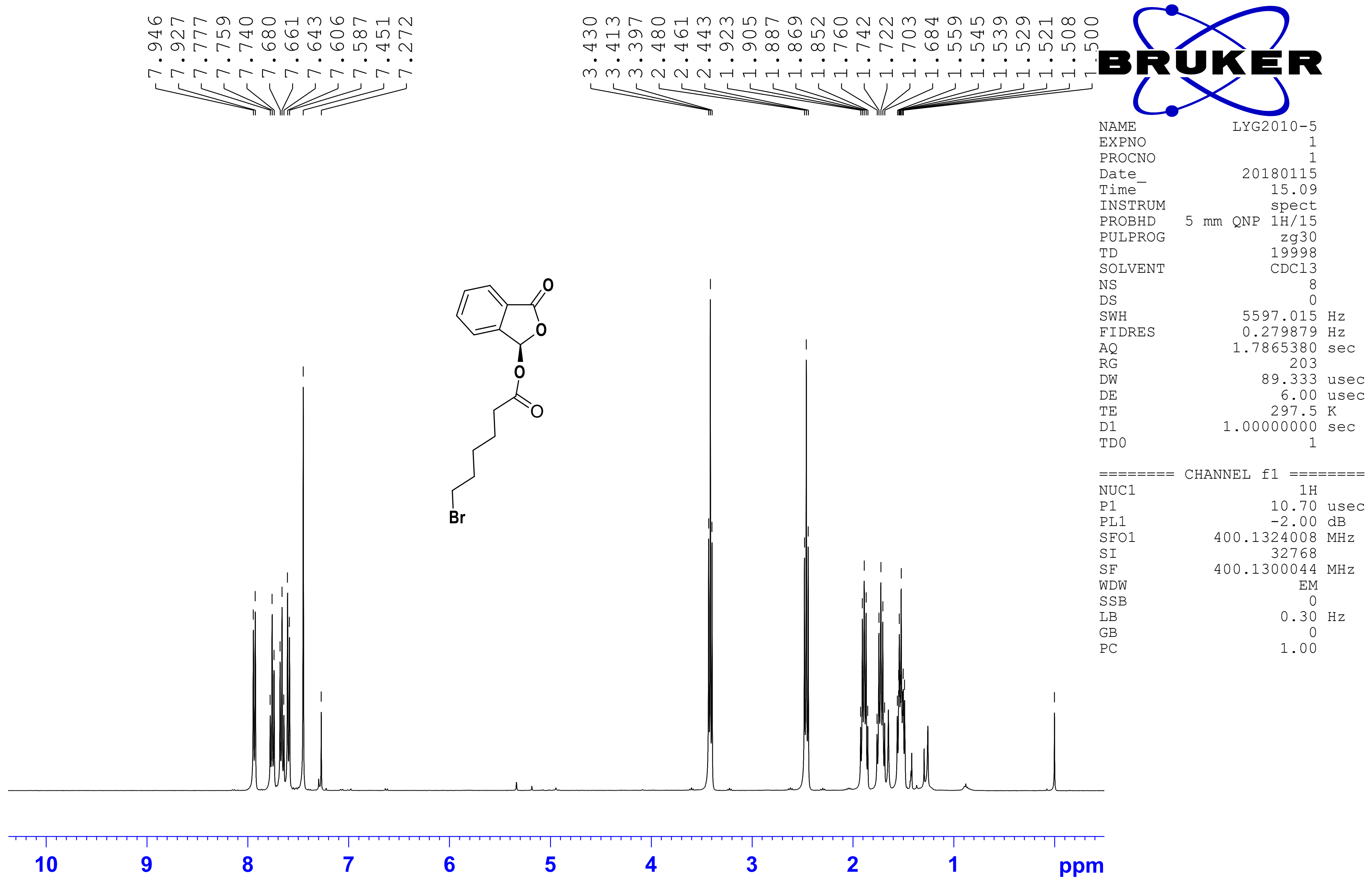

Supplementary Figure 61 ^{1}H NMR spectrum of **32**

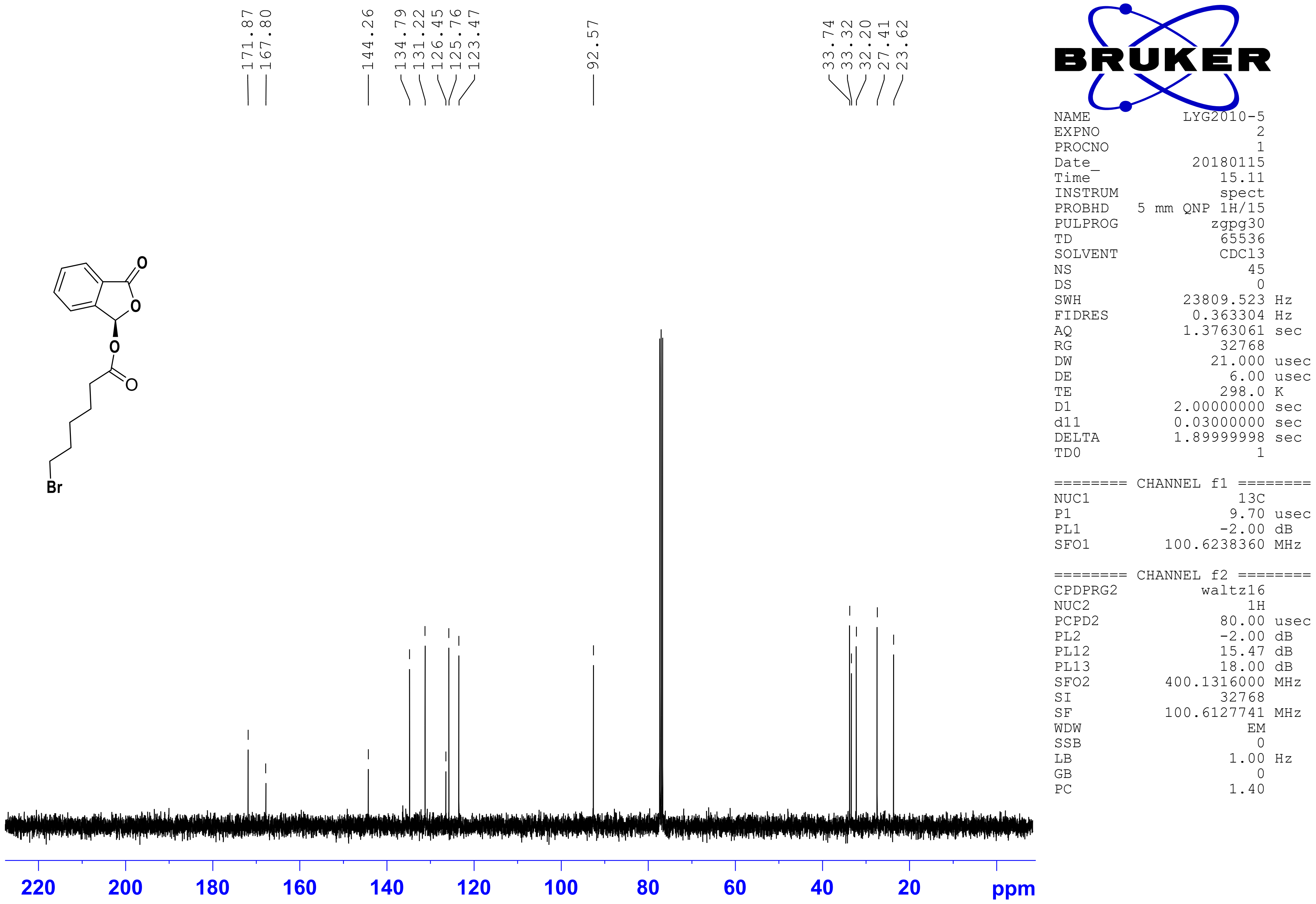

Supplementary Figure 62 ^{13}C NMR spectrum of **32**

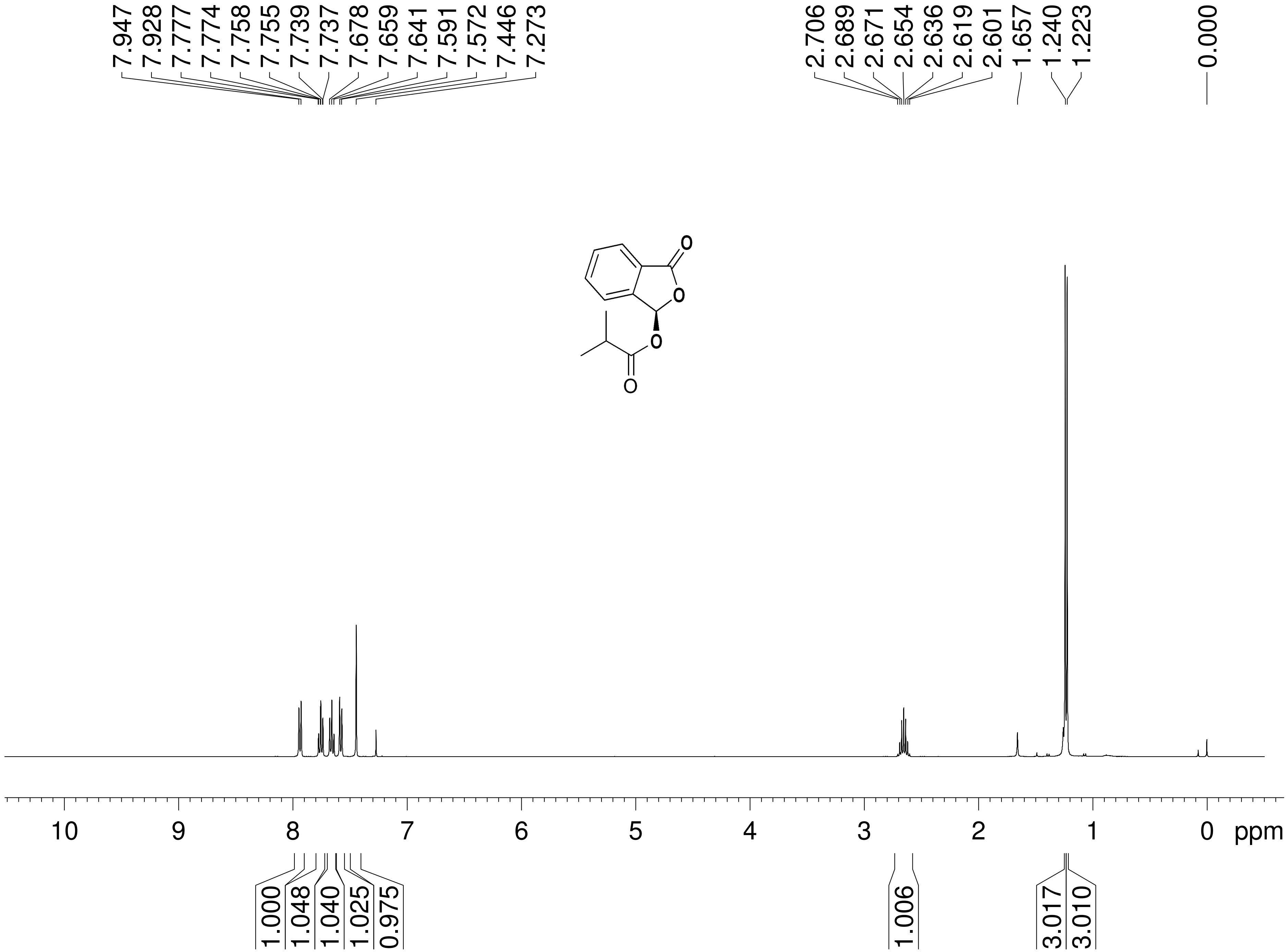

Supplementary Figure 63 ^{1}H NMR spectrum of **33**

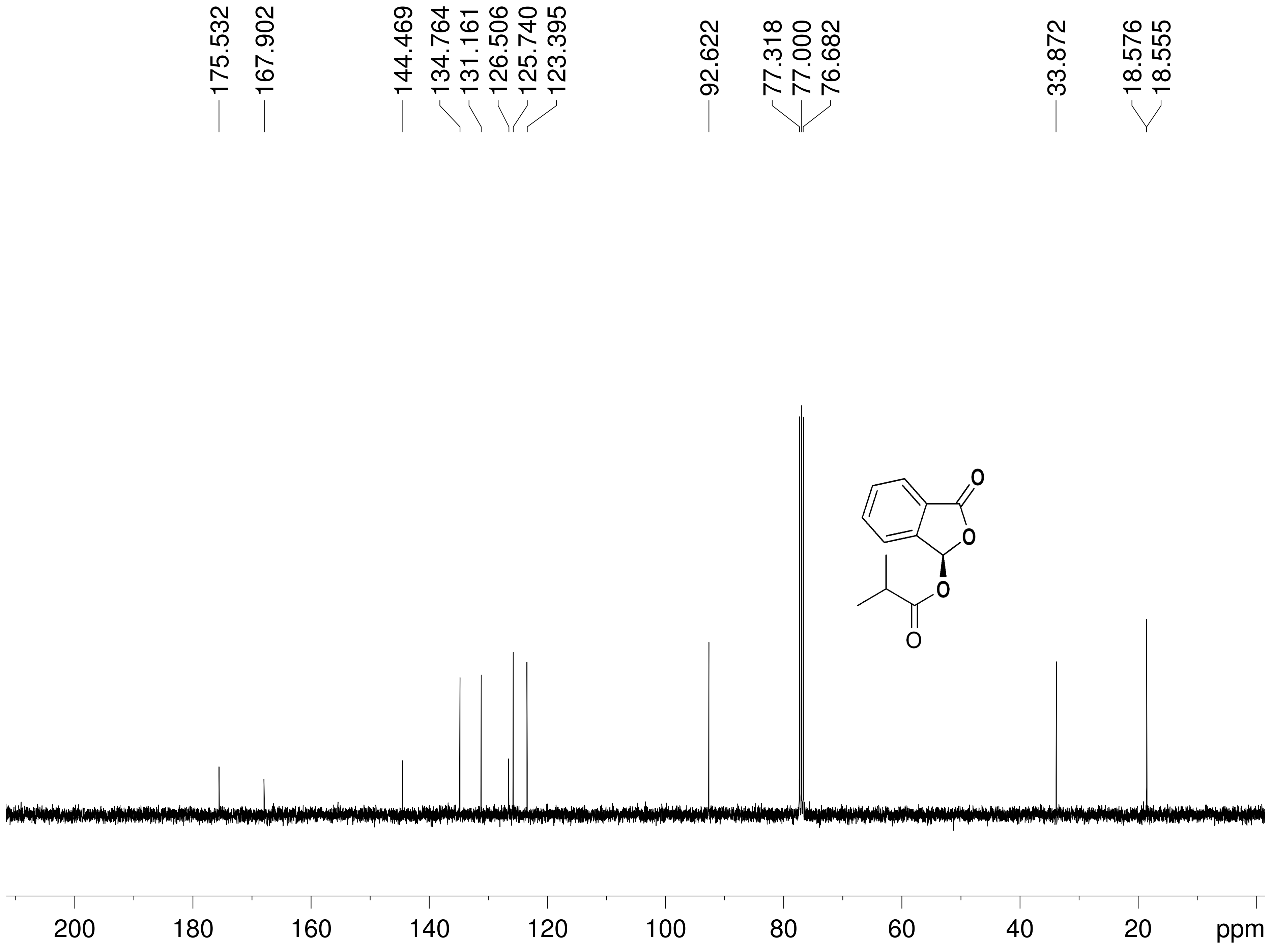

Supplementary Figure 64 ^{13}C NMR spectrum of **33**

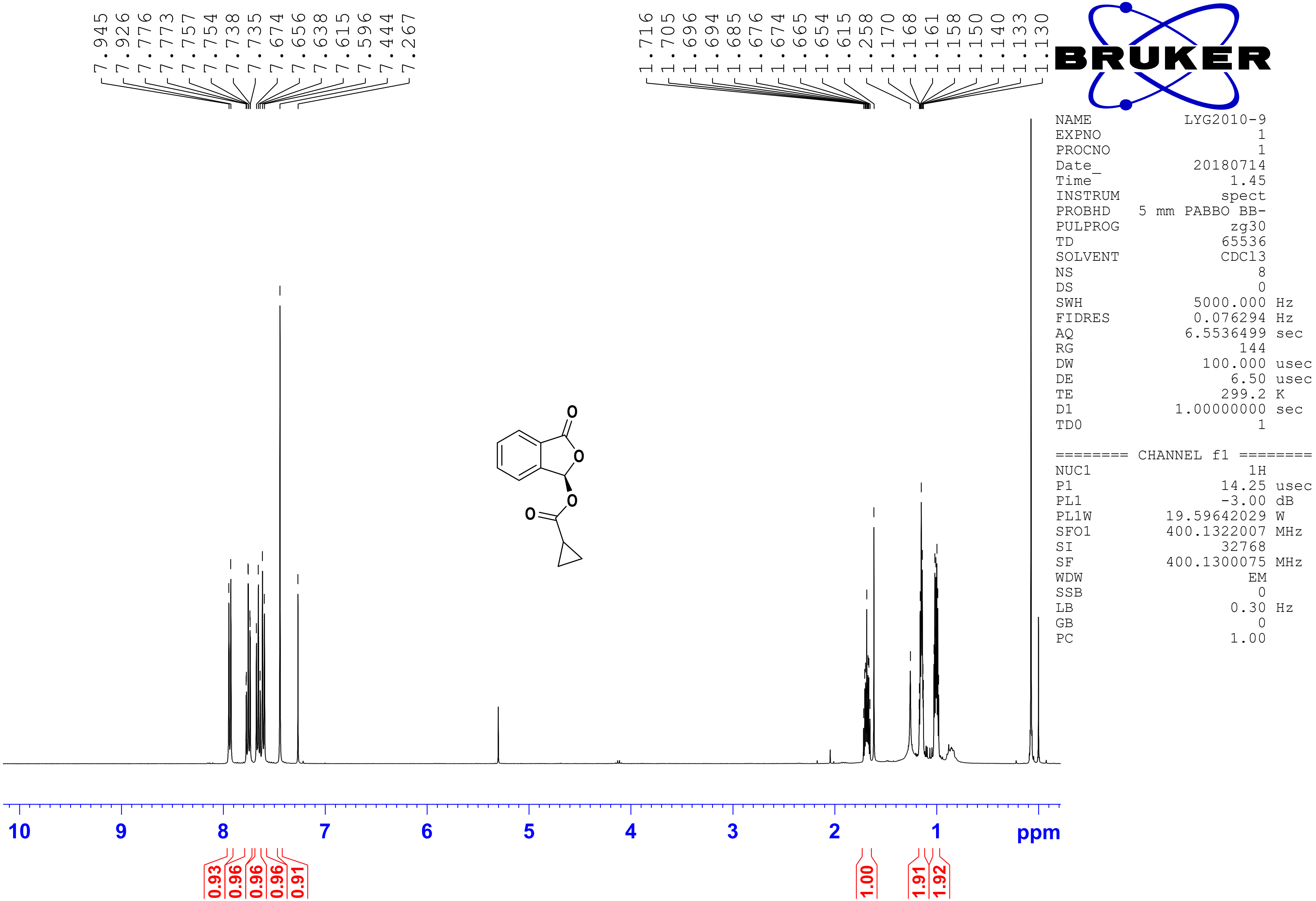

Supplementary Figure 65 ^{1}H NMR spectrum of **34**

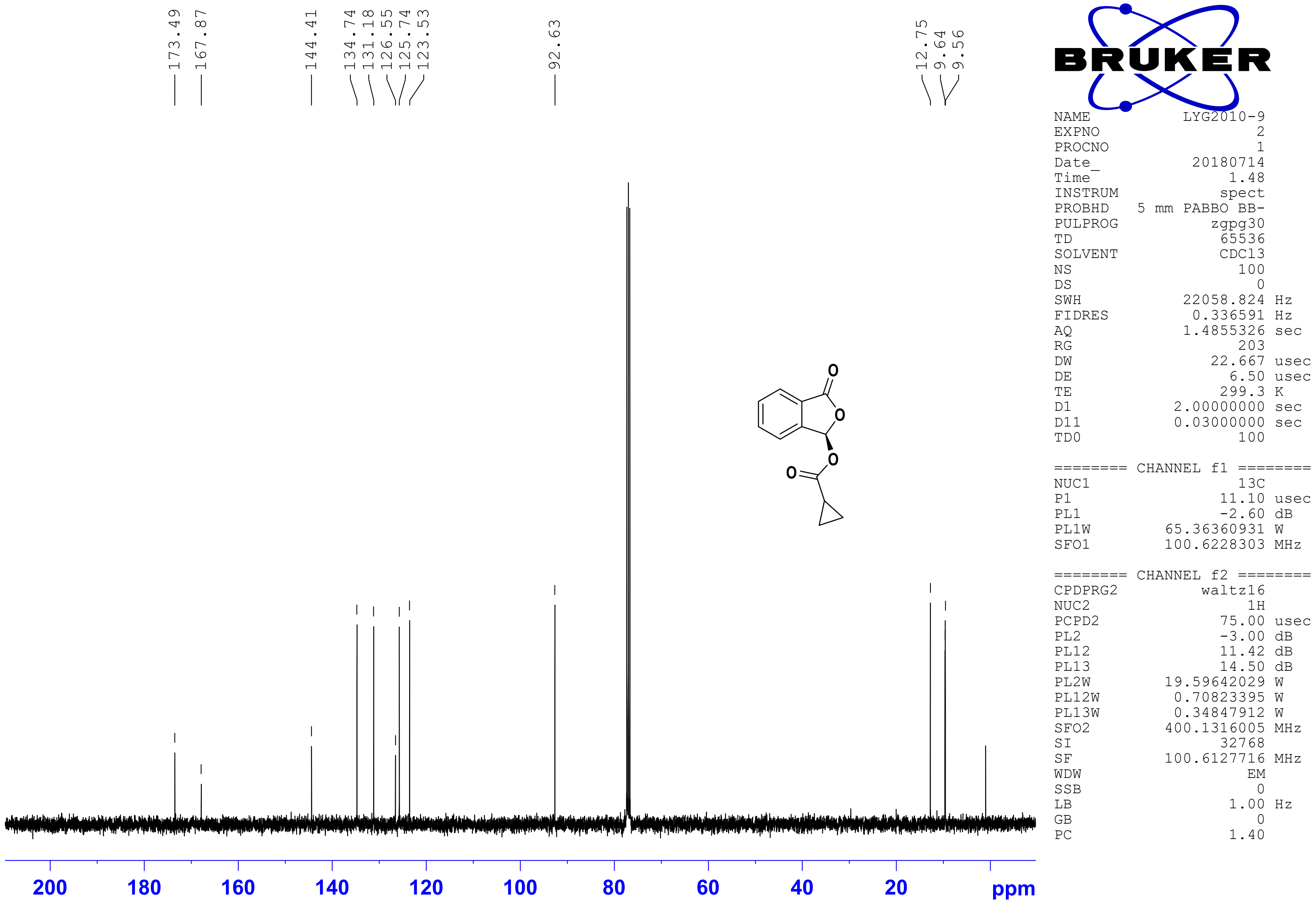

Supplementary Figure 66 ^{13}C NMR spectrum of **34**

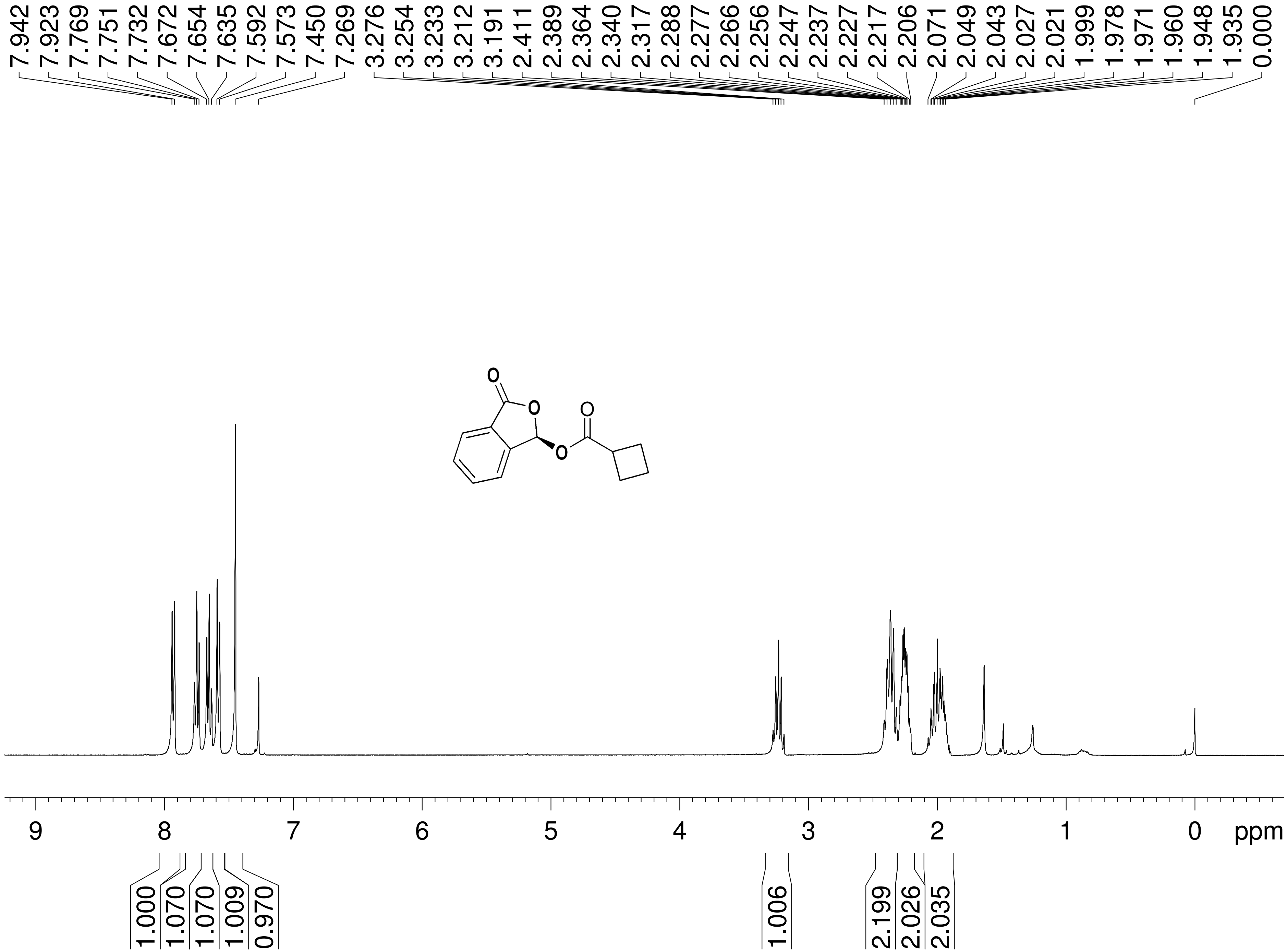

Supplementary Figure 67 ^{1}H NMR spectrum of **35**

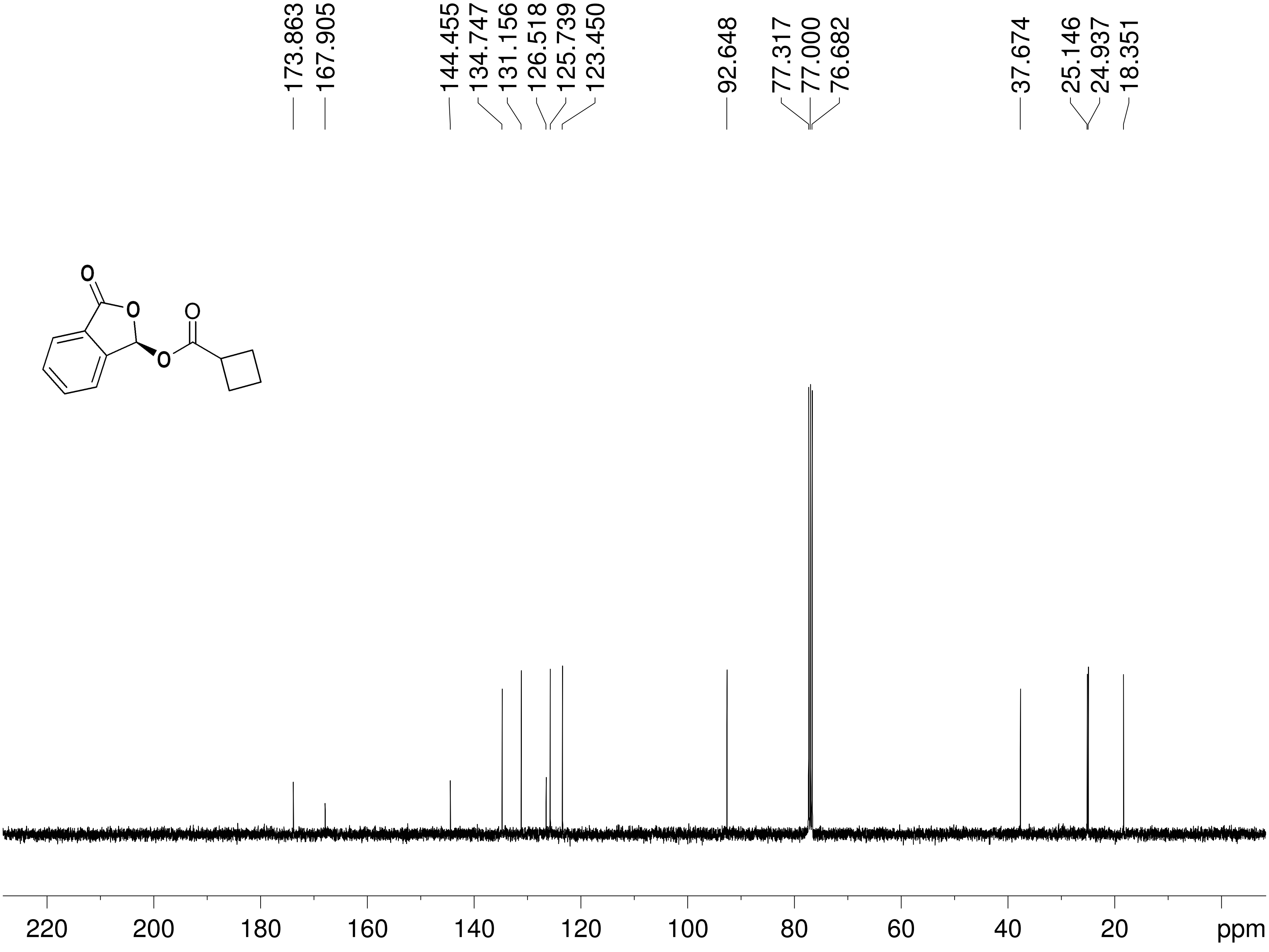

Supplementary Figure 68 ^{13}C NMR spectrum of **35**

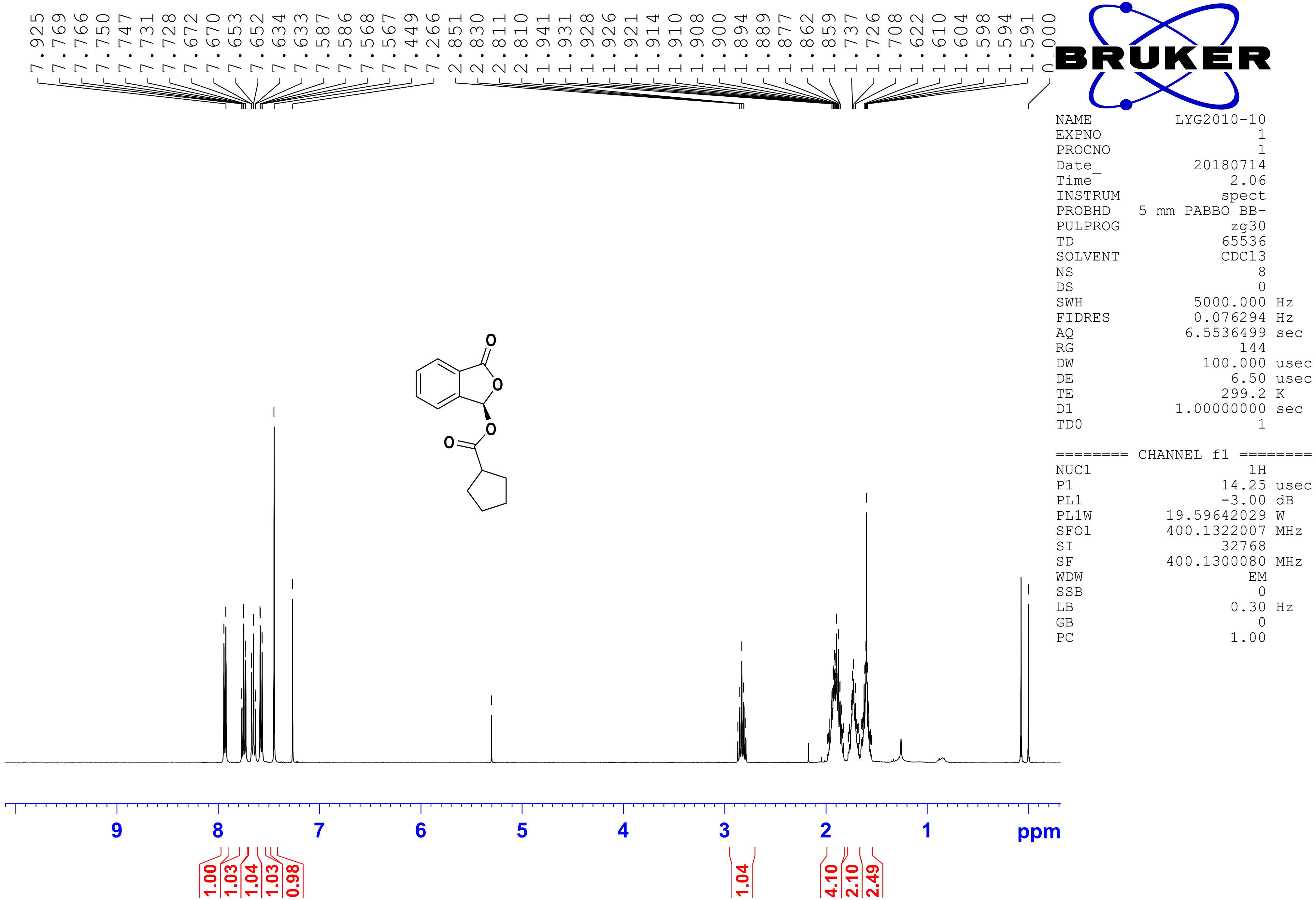

Supplementary Figure 69 ^{1}H NMR spectrum of **36**

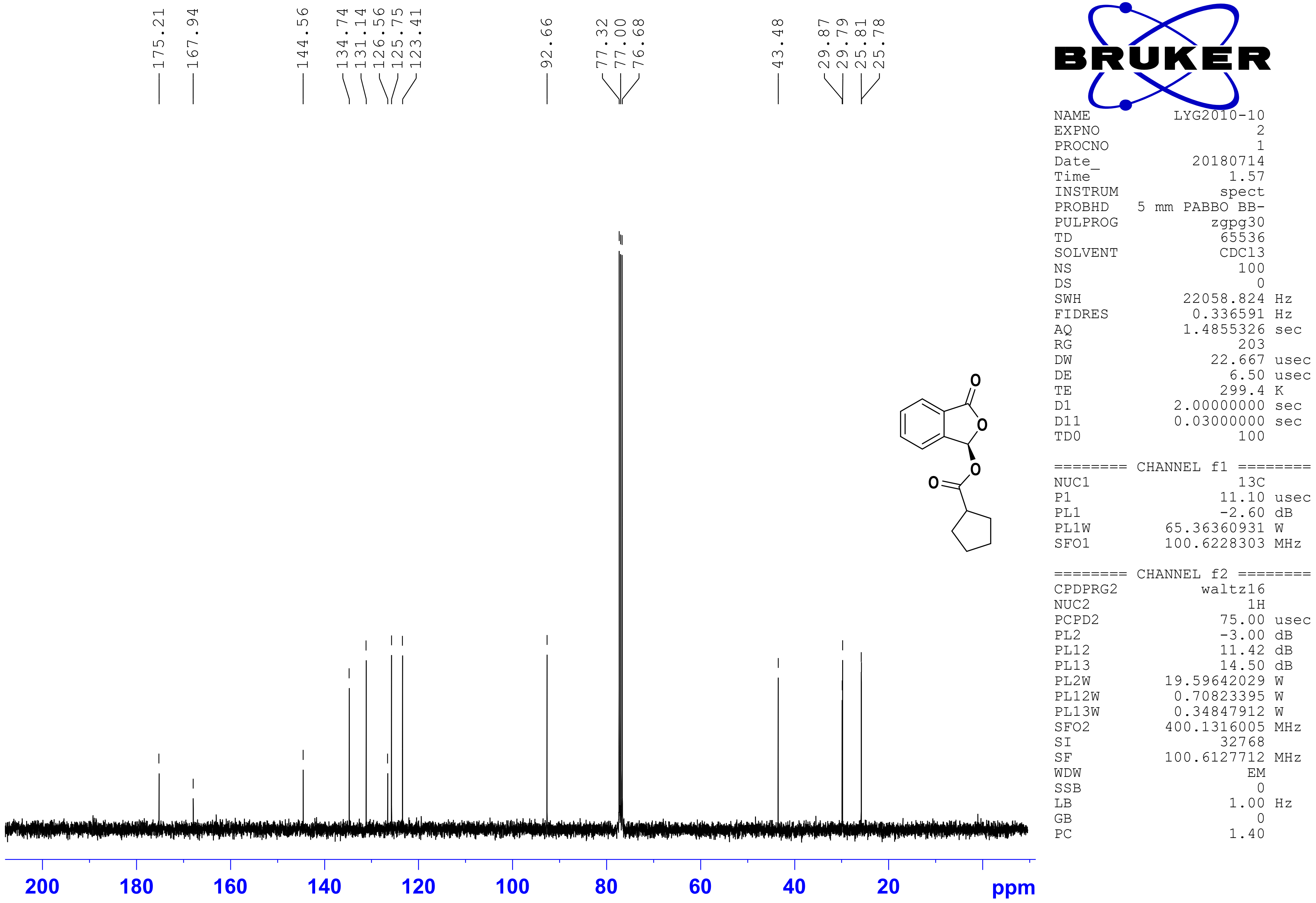

Supplementary Figure 70 ^{13}C NMR spectrum of **36**

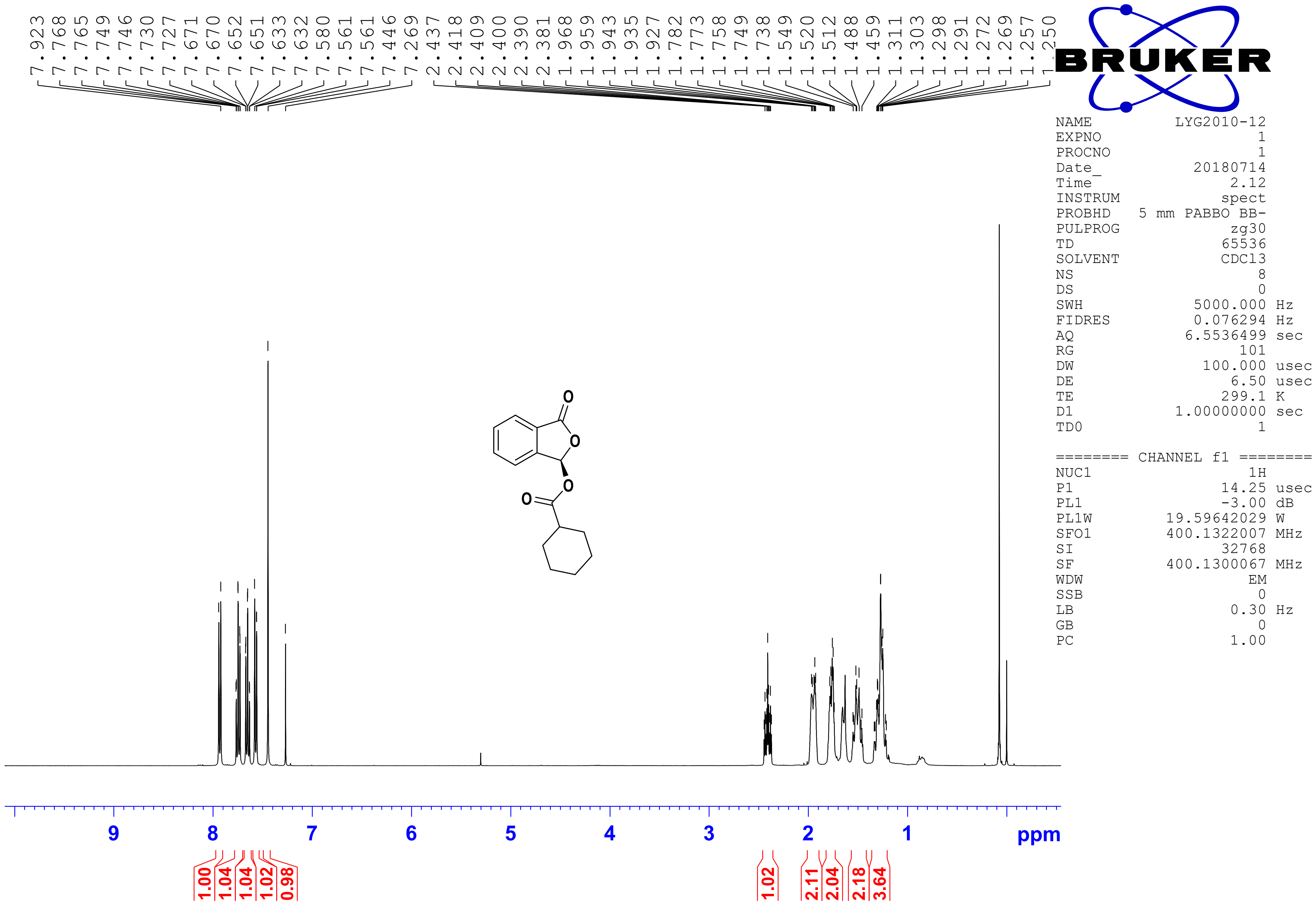

Supplementary Figure 71 ^{1}H NMR spectrum of **37**

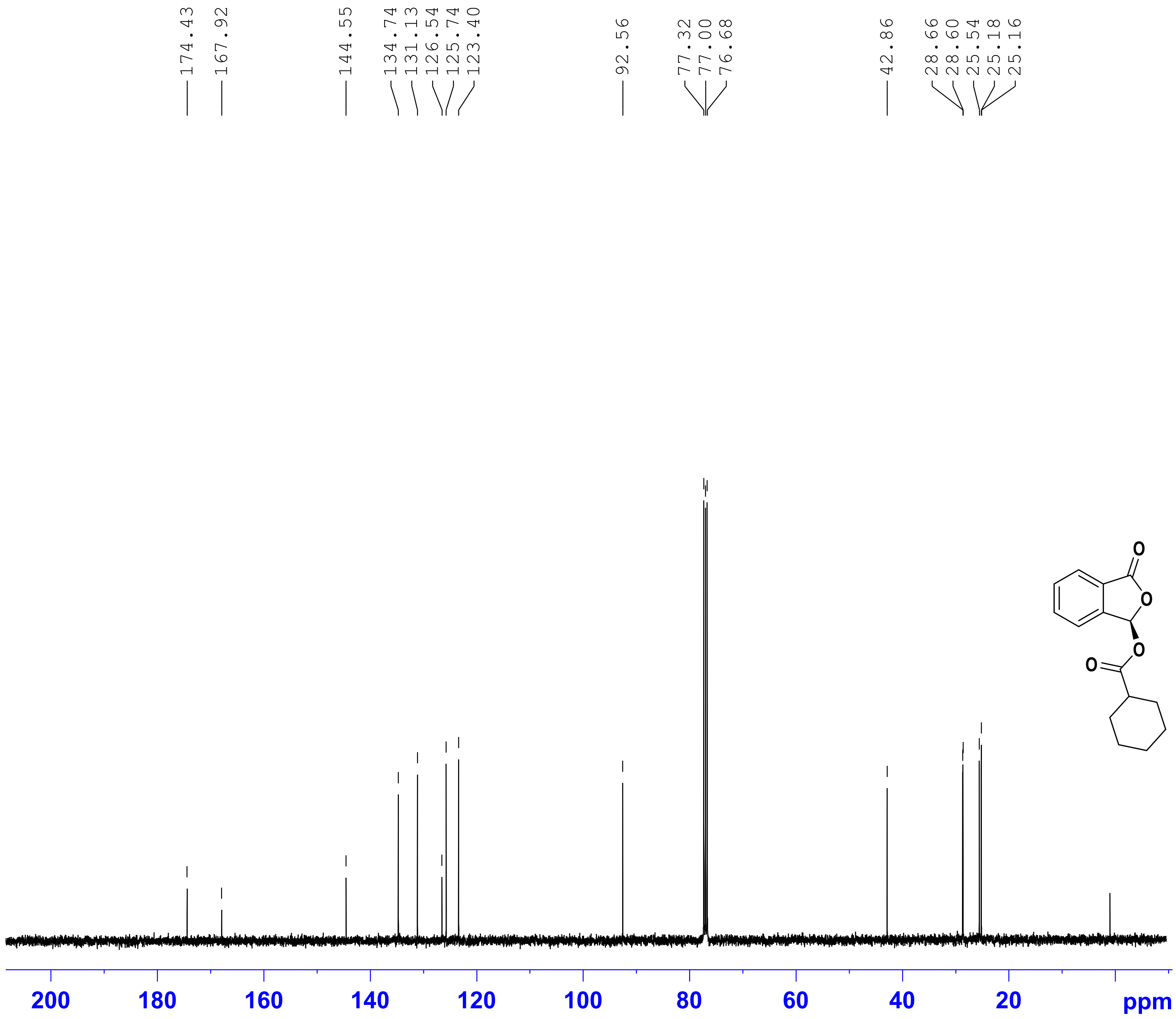

NAME	LYG2010-12
EXPNO	2
PROCNO	1
Date_	20180714
Time	2.16
INSTRUM	spect
PROBHD	5 mm PABBO BB-
PULPROG	zgpg30
TD	65536
SOLVENT	CDCl3
NS	100
DS	0
SWH	22058.824 Hz
FIDRES	0.336591 Hz
AQ	1.4855326 sec
RG	203
DW	22.667 usec
DE	6.50 usec
TE	299.6 K
D1	2.00000000 sec
D11	0.03000000 sec
TD0	100
======== CHANNEL f1 ========	
NUC1	13C
P1	11.10 usec
PL1	-2.60 dB
PL1W	65.36360931 W
SFO1	100.6228303 MHz
======== CHANNEL f2 ========	
CPDPRG2	waltz16
NUC2	1H
PCPD2	75.00 usec
PL2	-3.00 dB
PL12	11.42 dB
PL13	14.50 dB
PL2W	19.59642029 W
PL12W	0.70823395 W
PL13W	0.34847912 W
SFO2	400.1316005 MHz
SI	32768
SF	100.6127721 MHz
WDW	EM
SSB	0
LB	1.00 Hz
GB	0
PC	1.40

Supplementary Figure 72 ^{13}C NMR spectrum of **37**

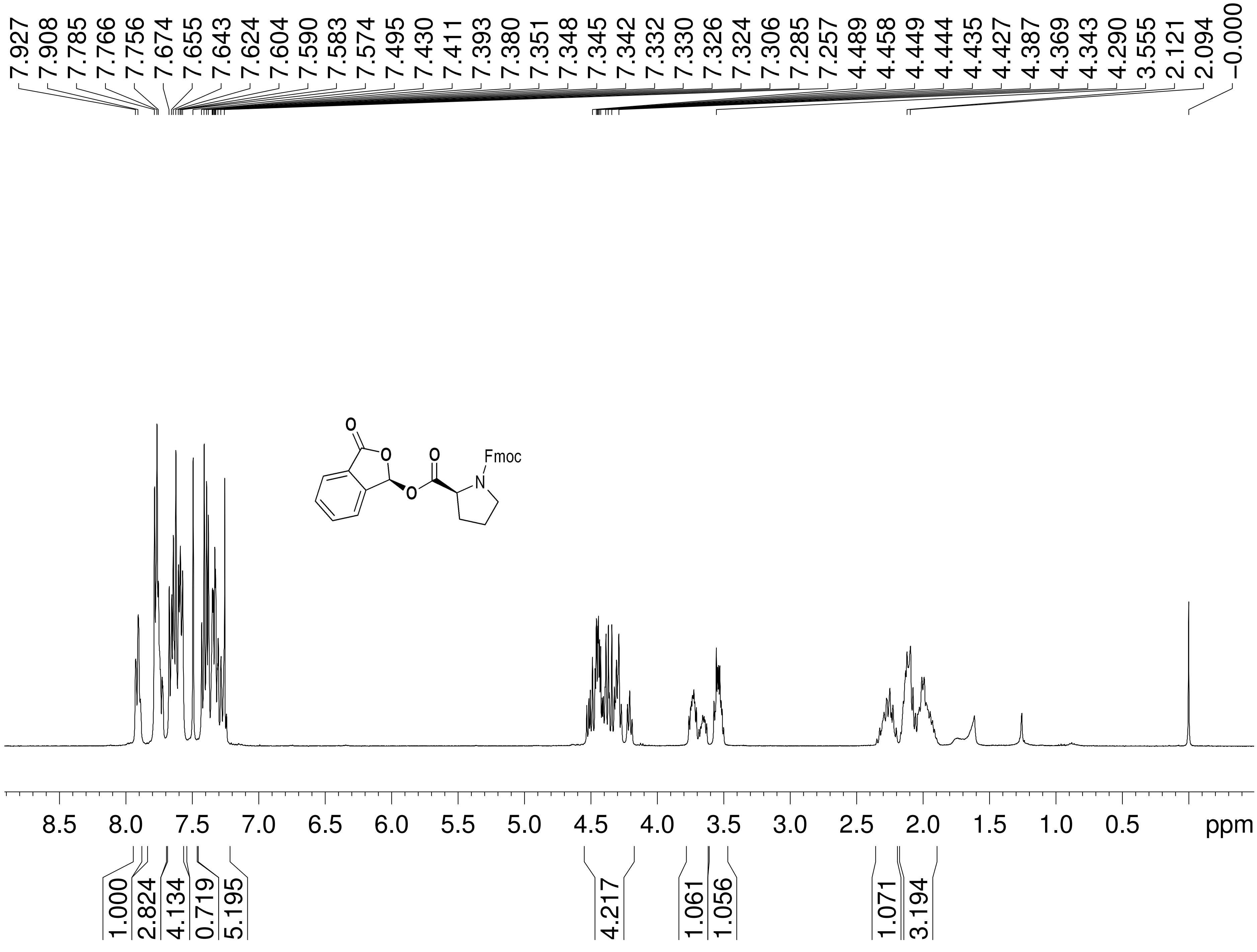

Supplementary Figure 73 ^{1}H NMR spectrum of **38**

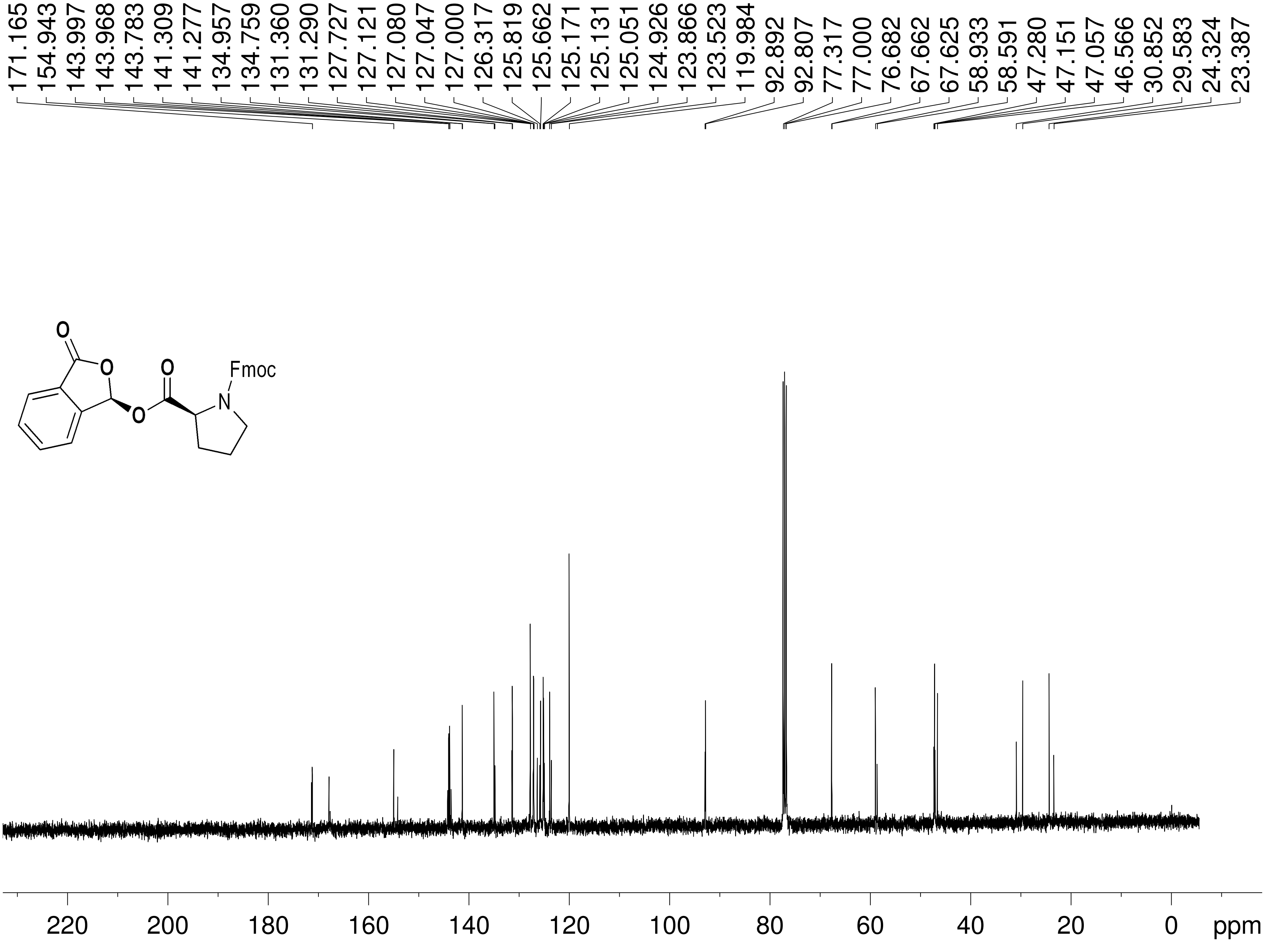

Supplementary Figure 74 ^{13}C NMR spectrum of **38**

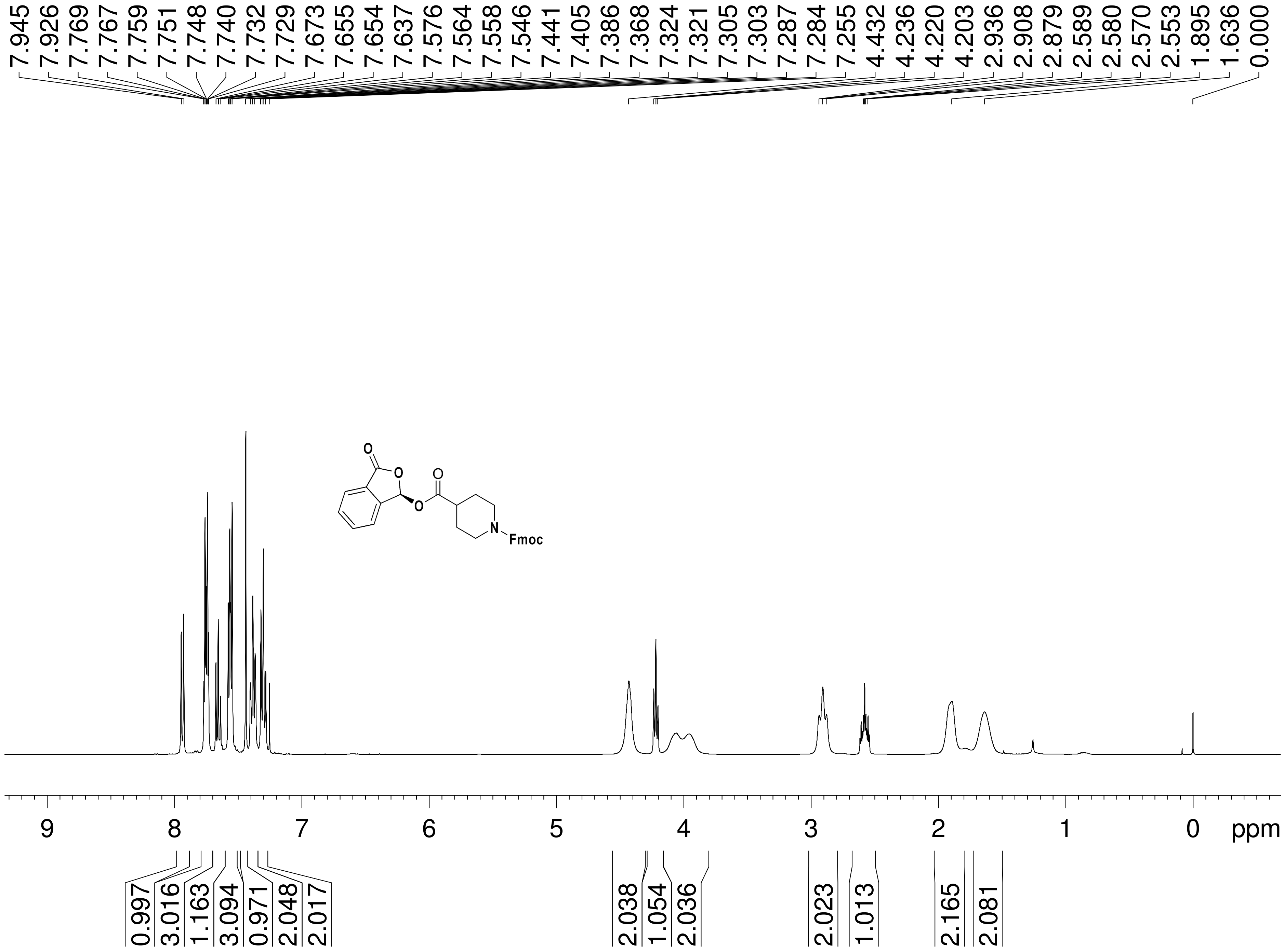

Supplementary Figure 75 ^{1}H NMR spectrum of **39**

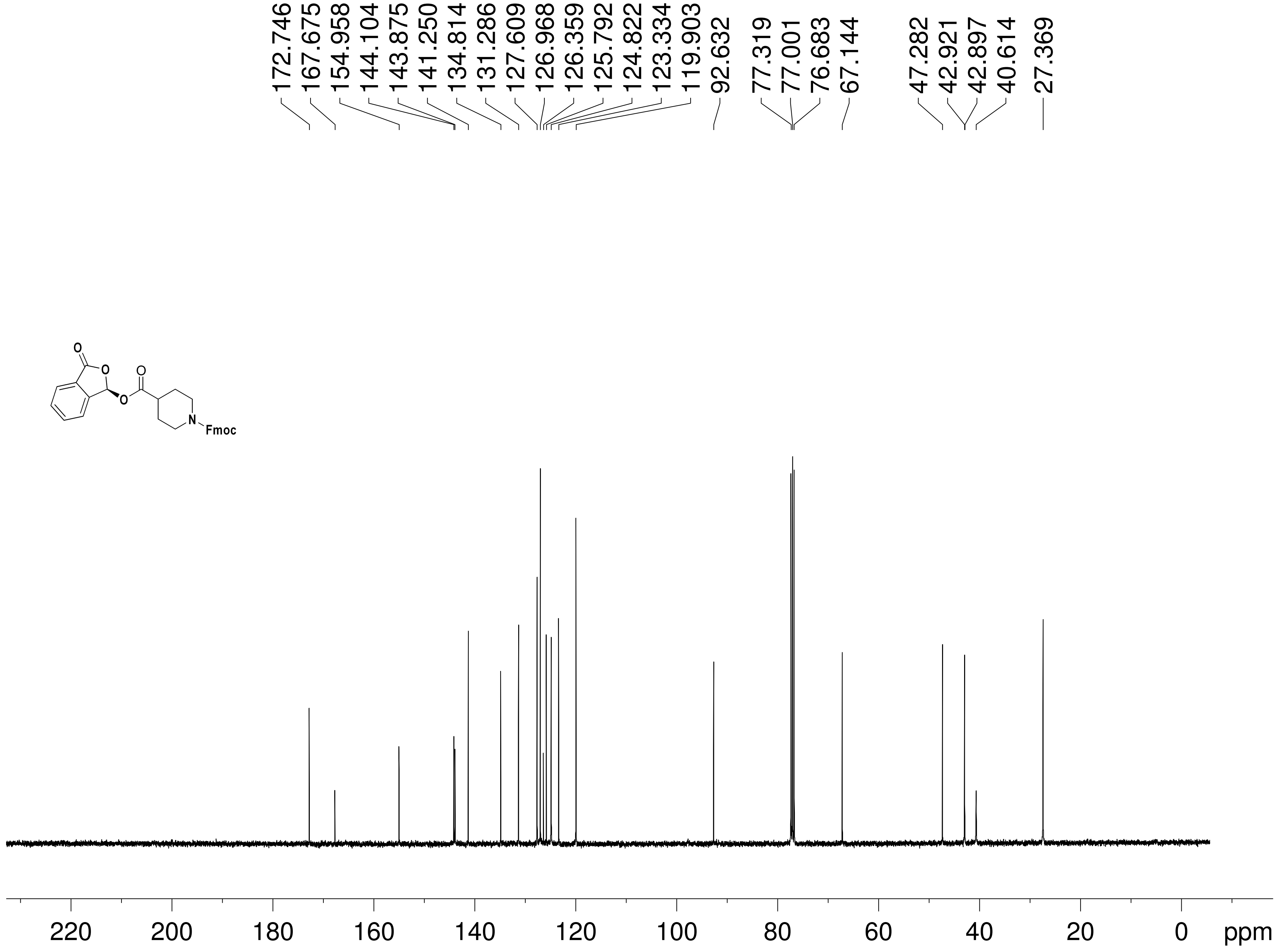

Supplementary Figure 76 ^{13}C NMR spectrum of **39**

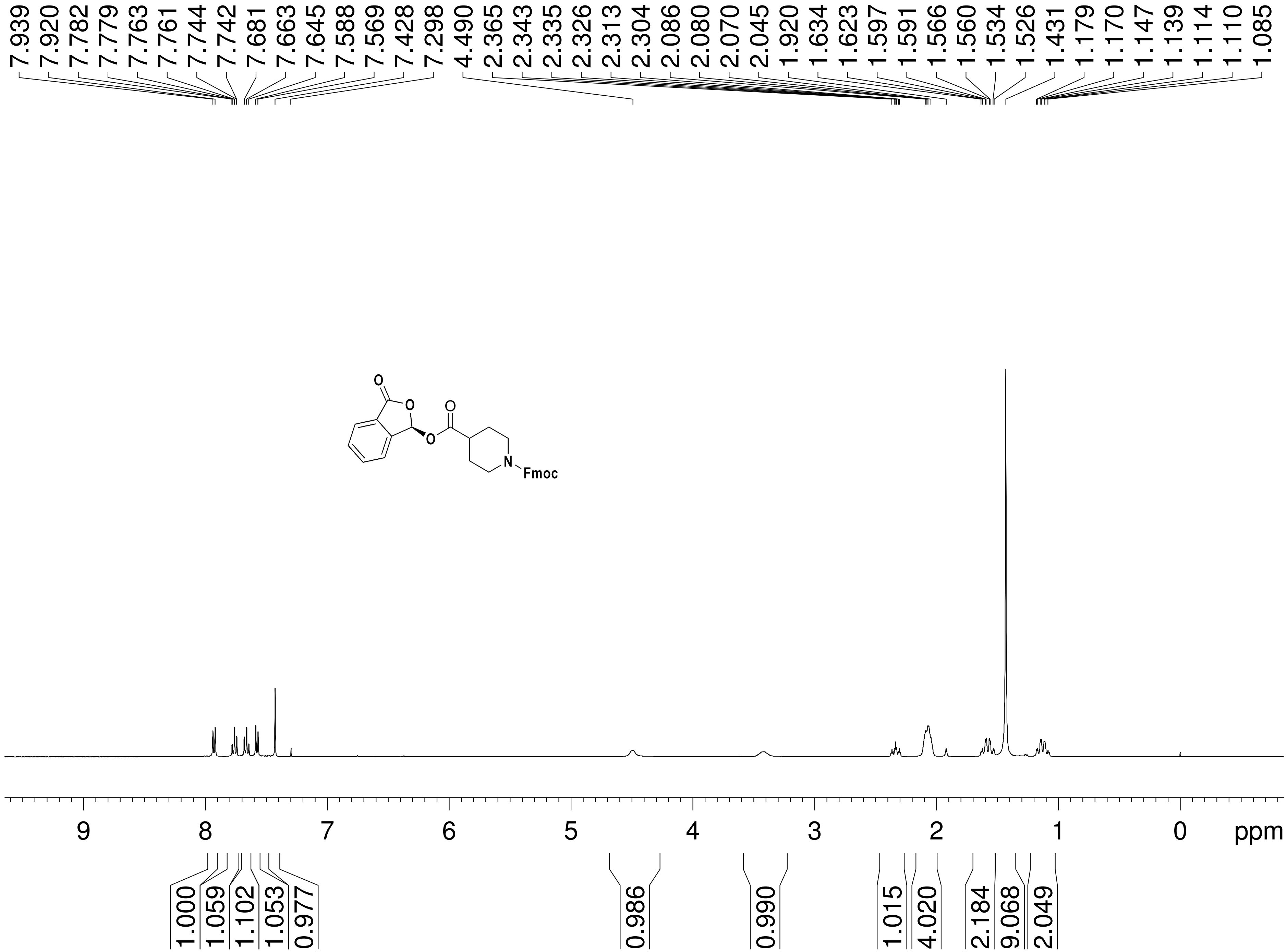

Supplementary Figure 77 ^{1}H NMR spectrum of **40**

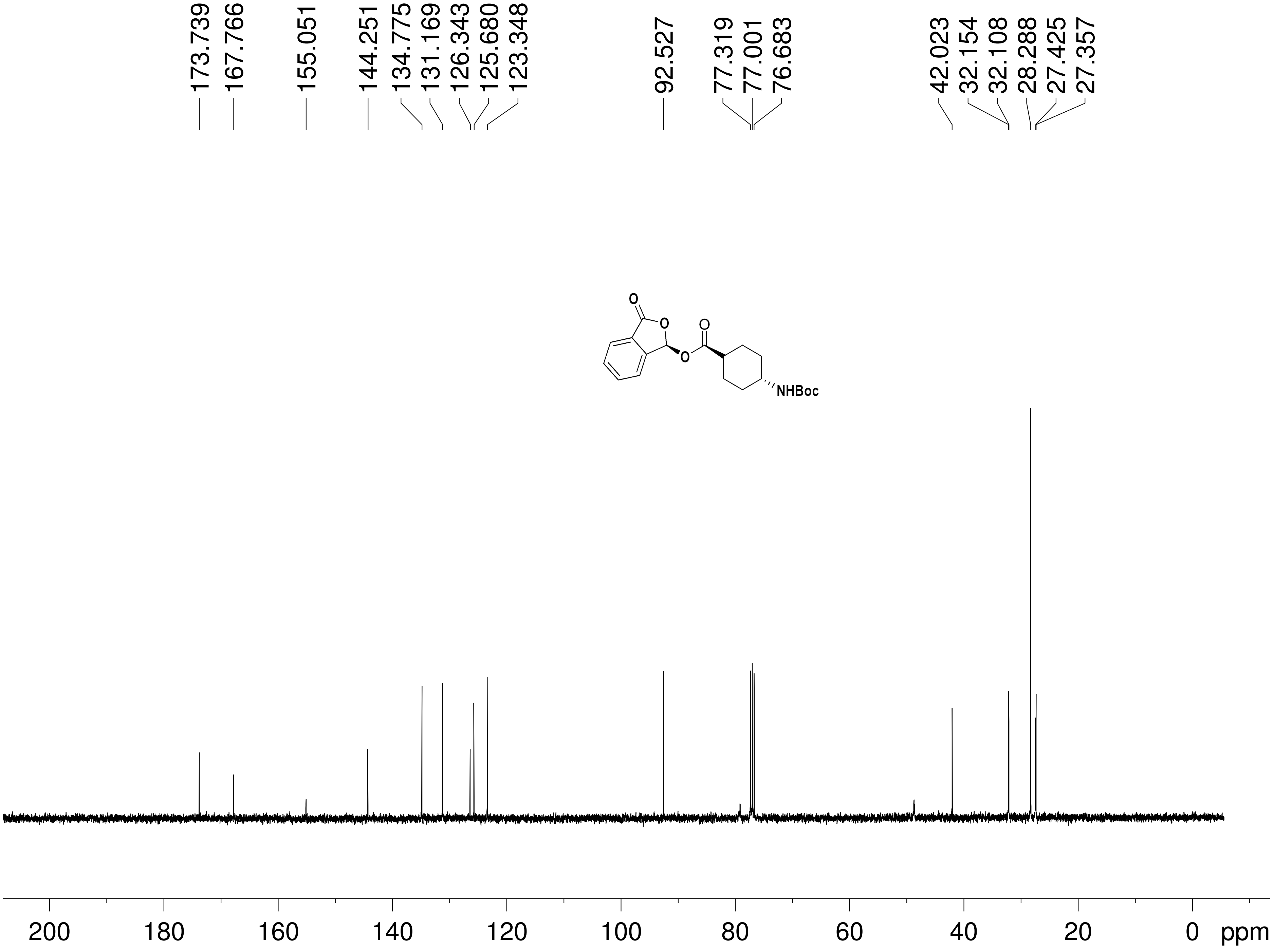

Supplementary Figure 78 ^{13}C NMR spectrum of **40**

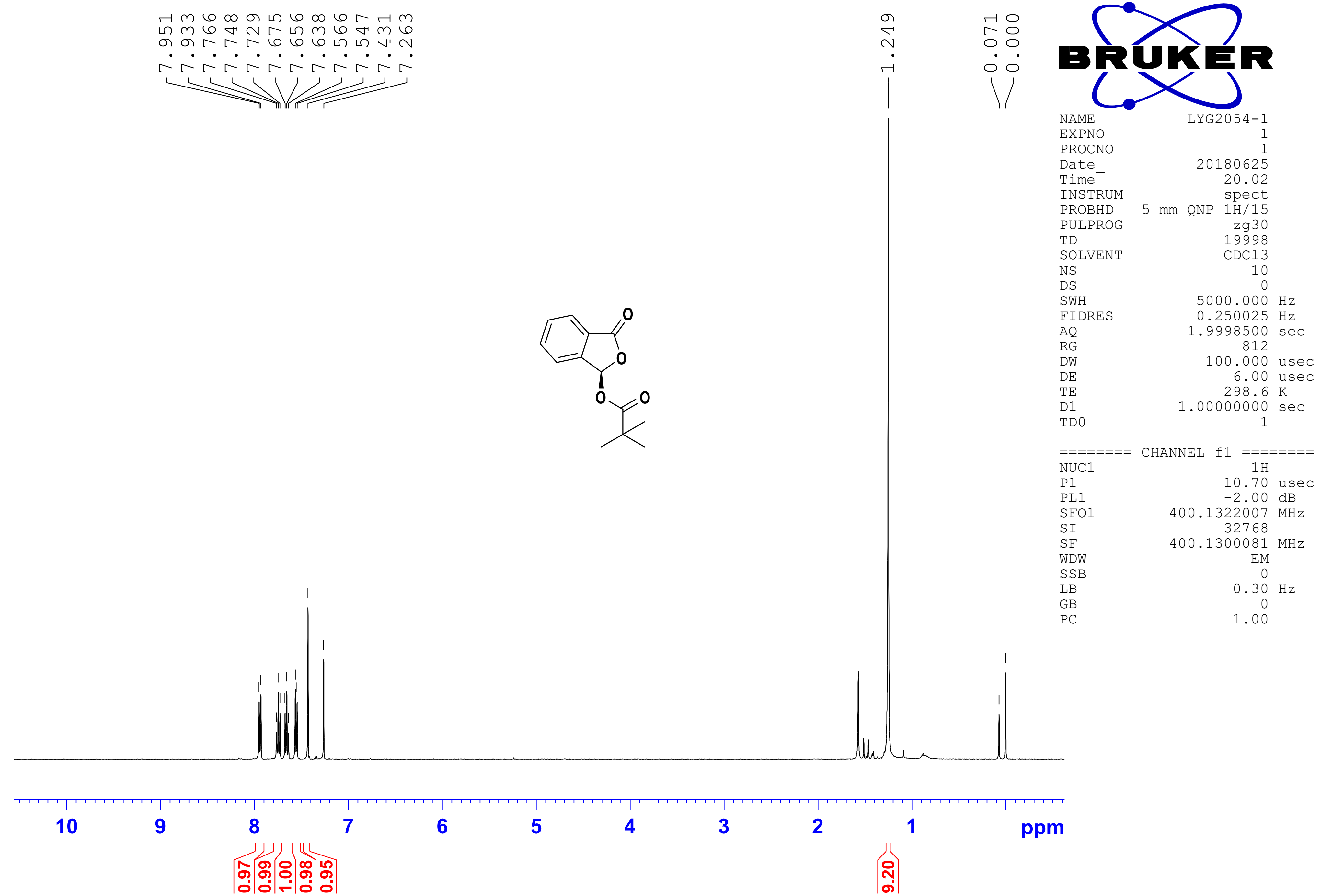

Supplementary Figure 79 ^{1}H NMR spectrum of **41**

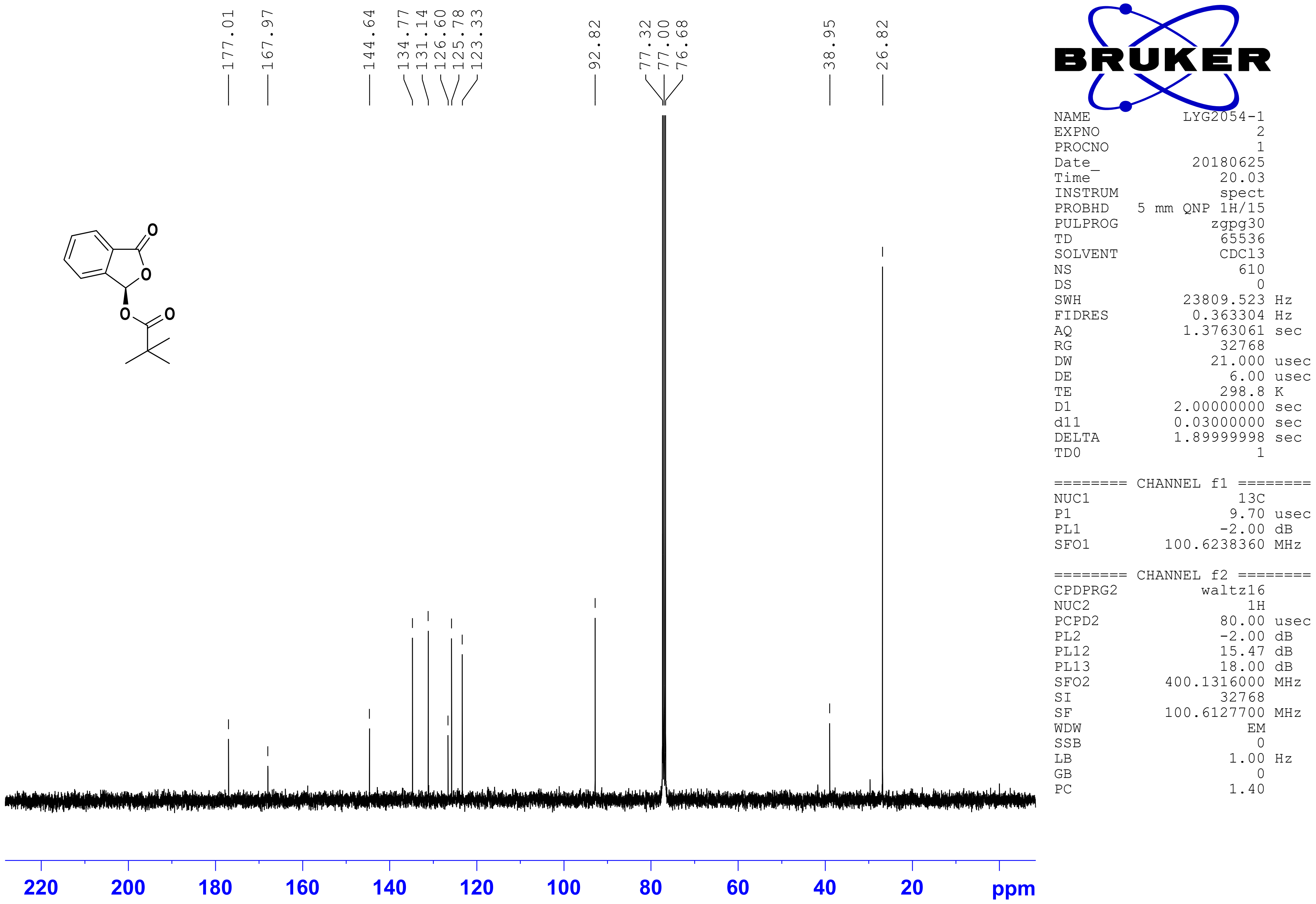

Supplementary Figure 80 ^{13}C NMR spectrum of **41**

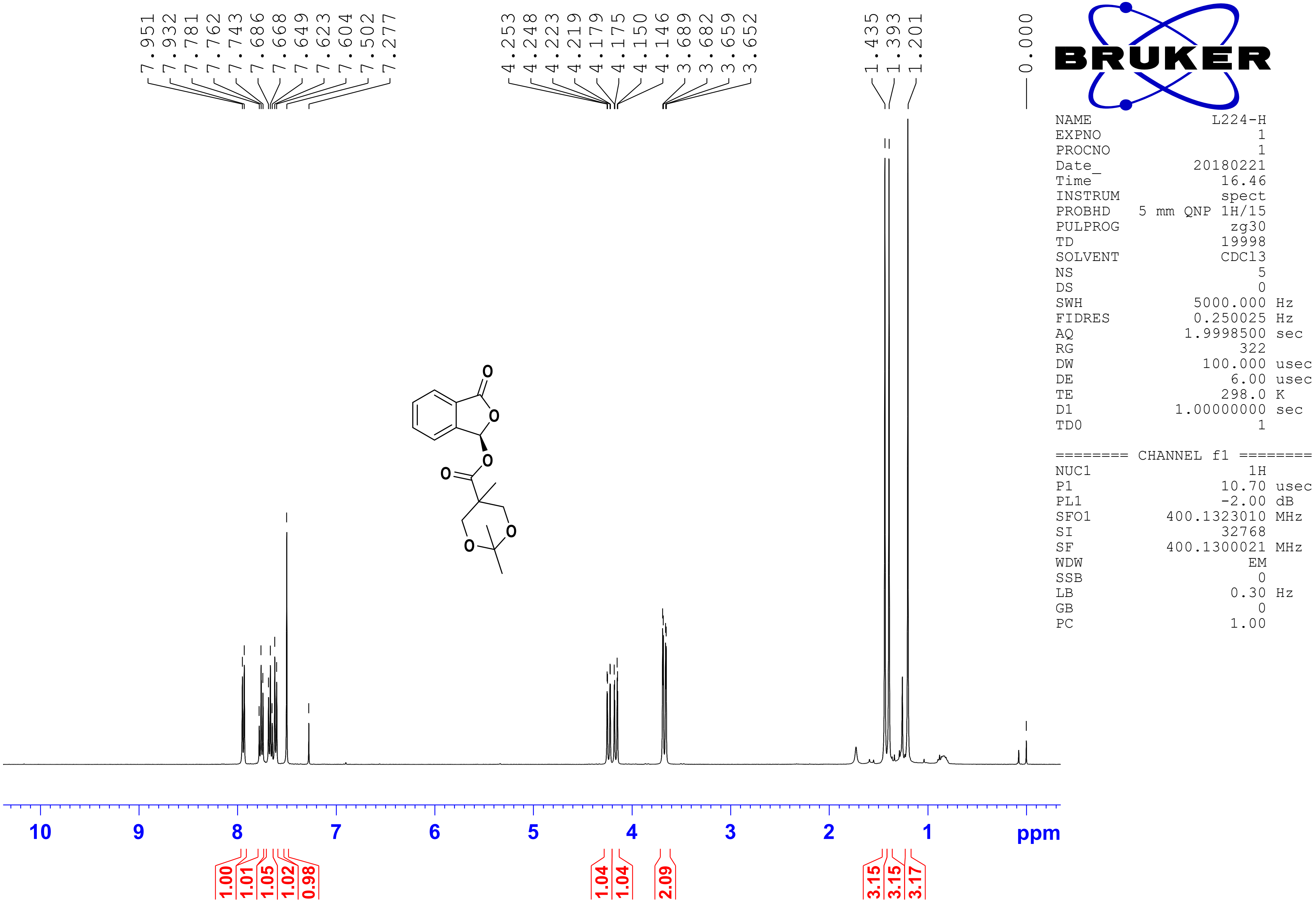

Supplementary Figure 81 ^{1}H NMR spectrum of **42**

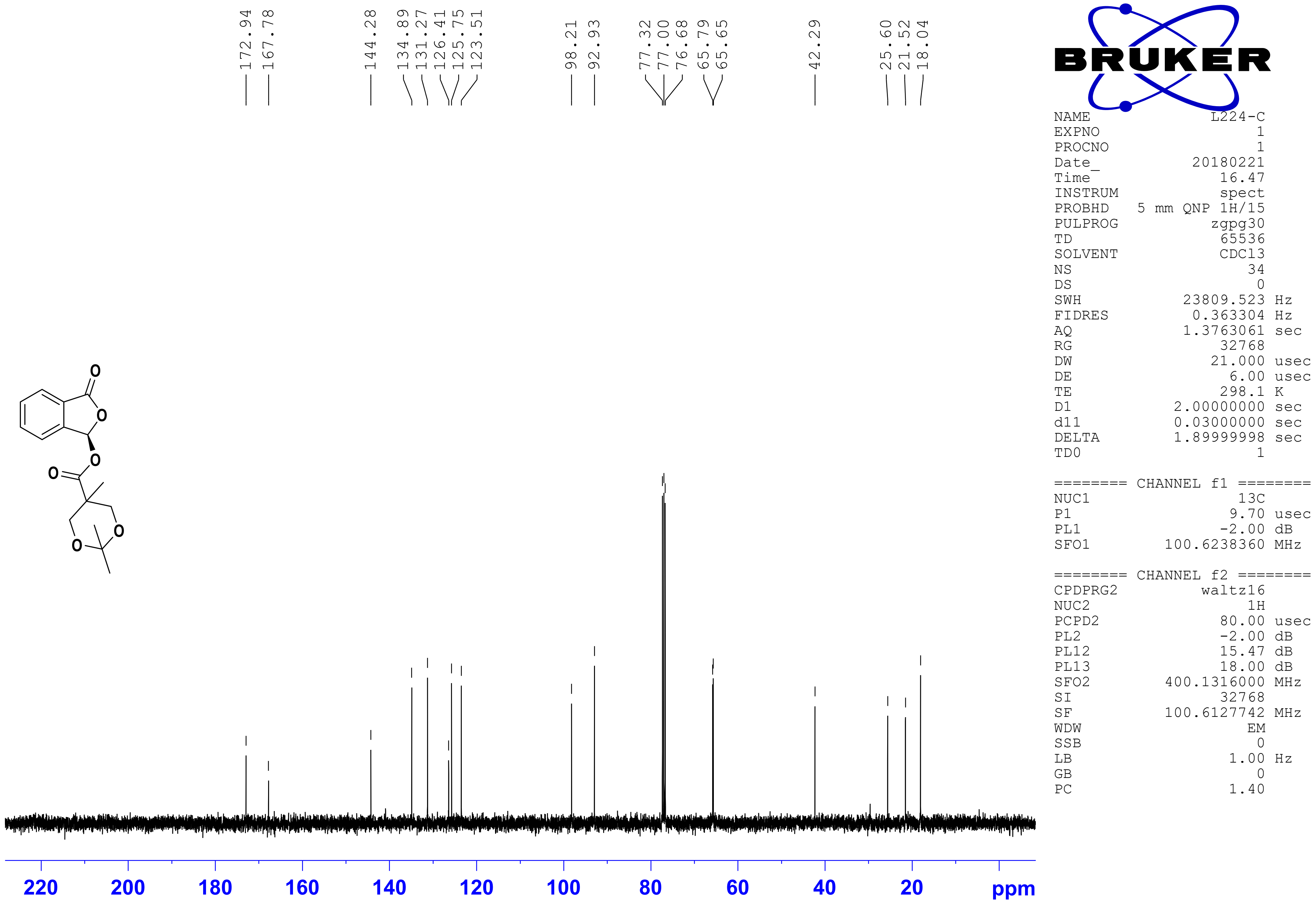

Supplementary Figure 82 ^{13}C NMR spectrum of **42**

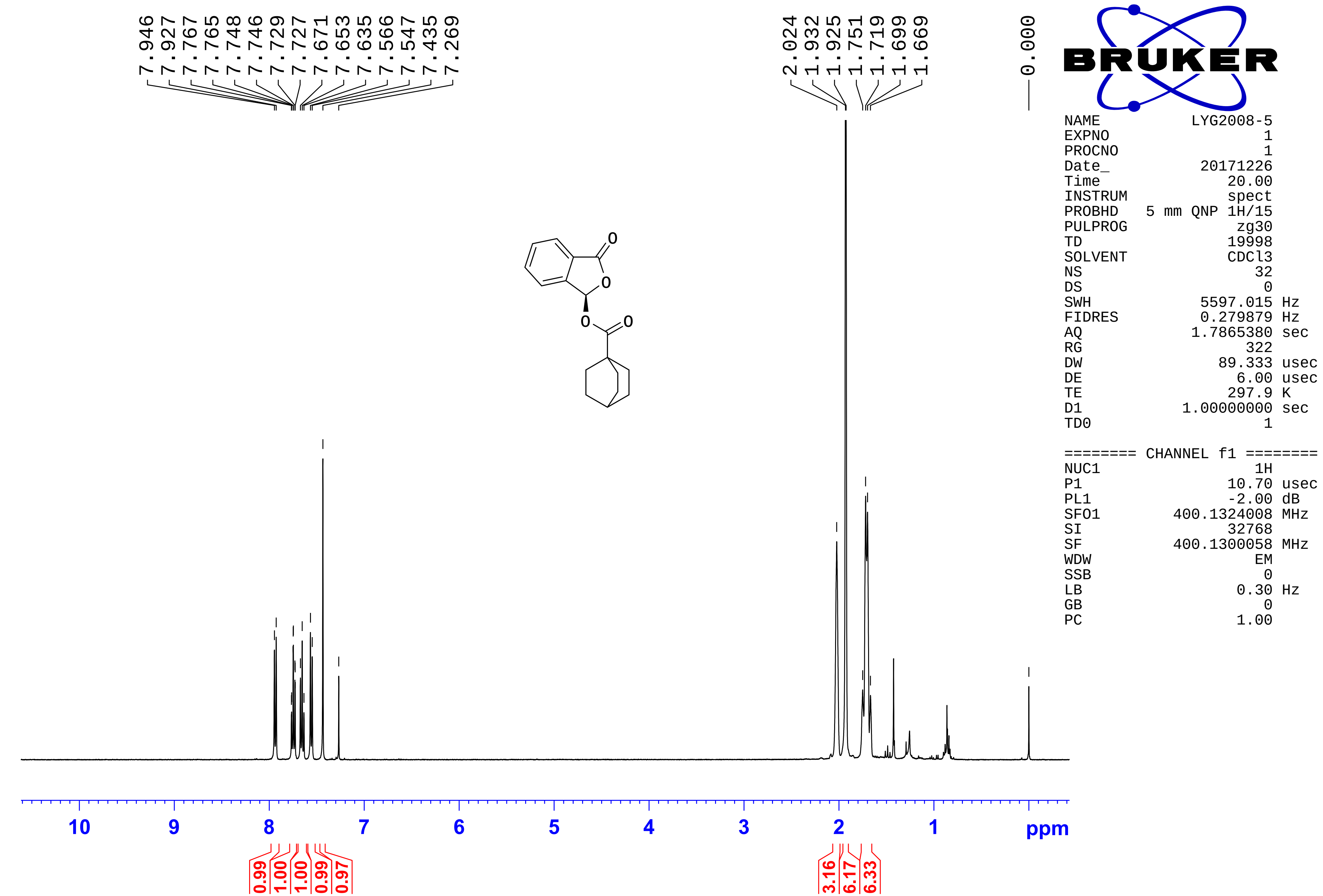

Supplementary Figure 83 ^{1}H NMR spectrum of **43**

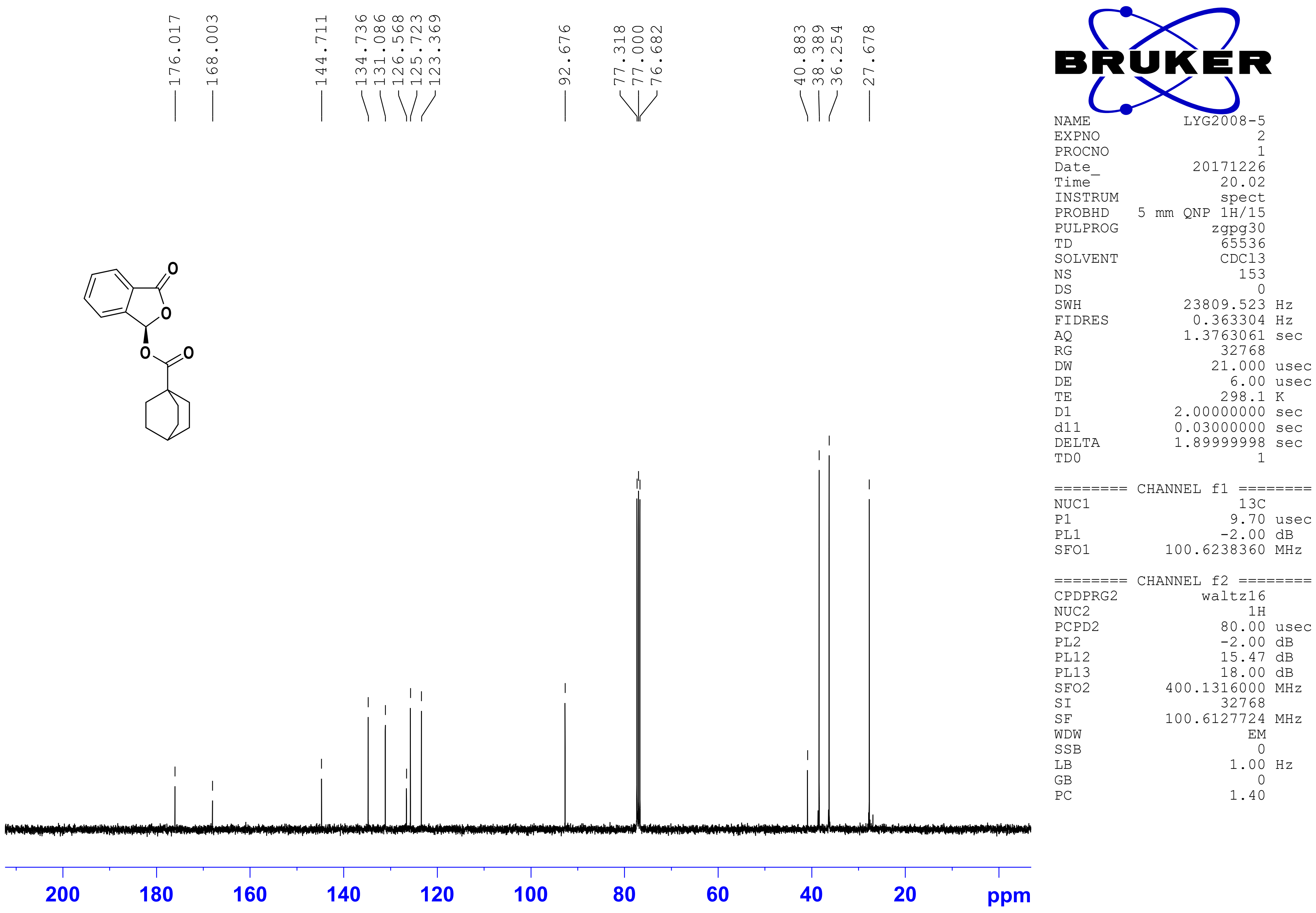

Supplementary Figure 84 ^{13}C NMR spectrum of **43**

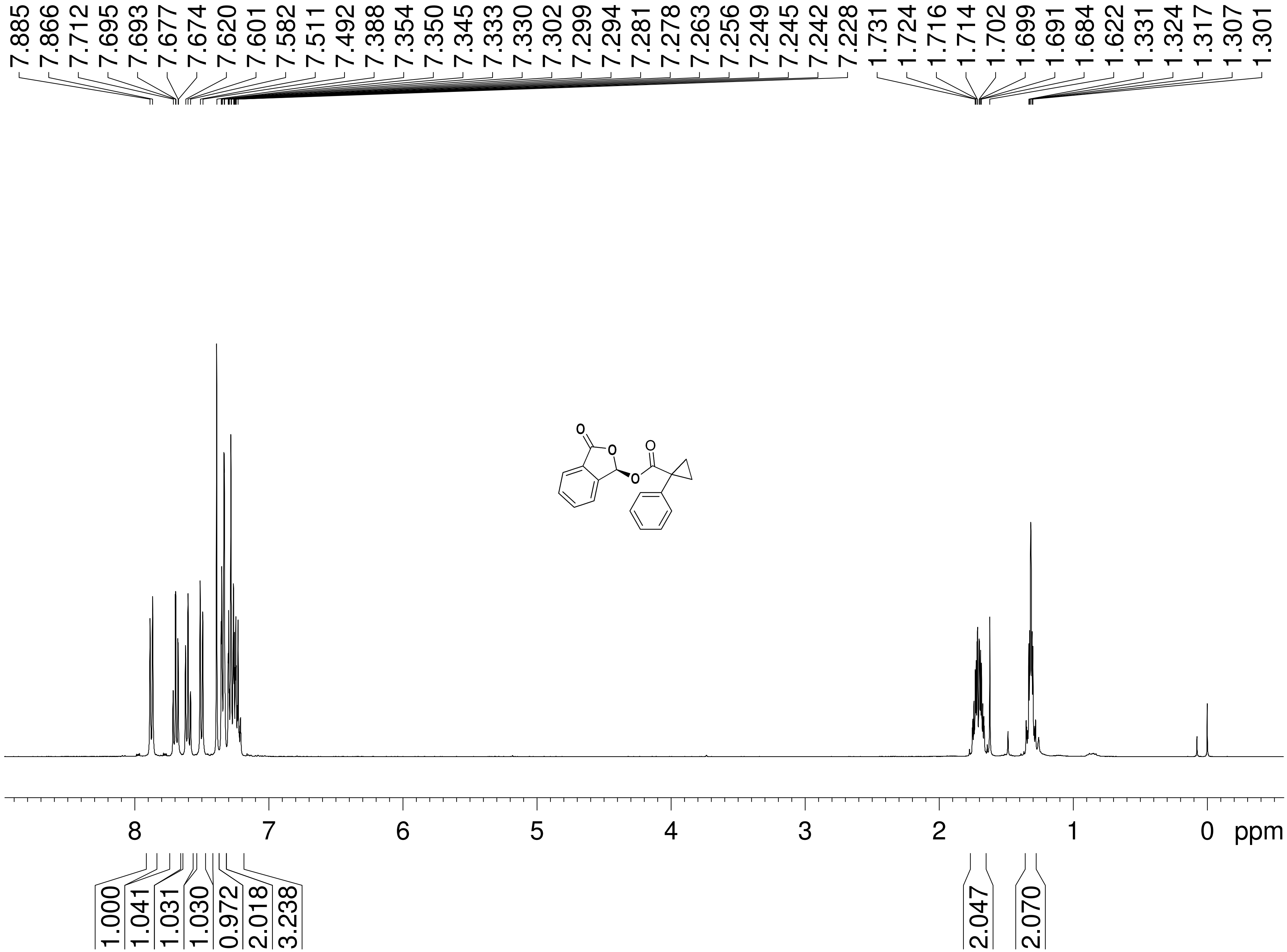

Supplementary Figure 85 ^{1}H NMR spectrum of **44**

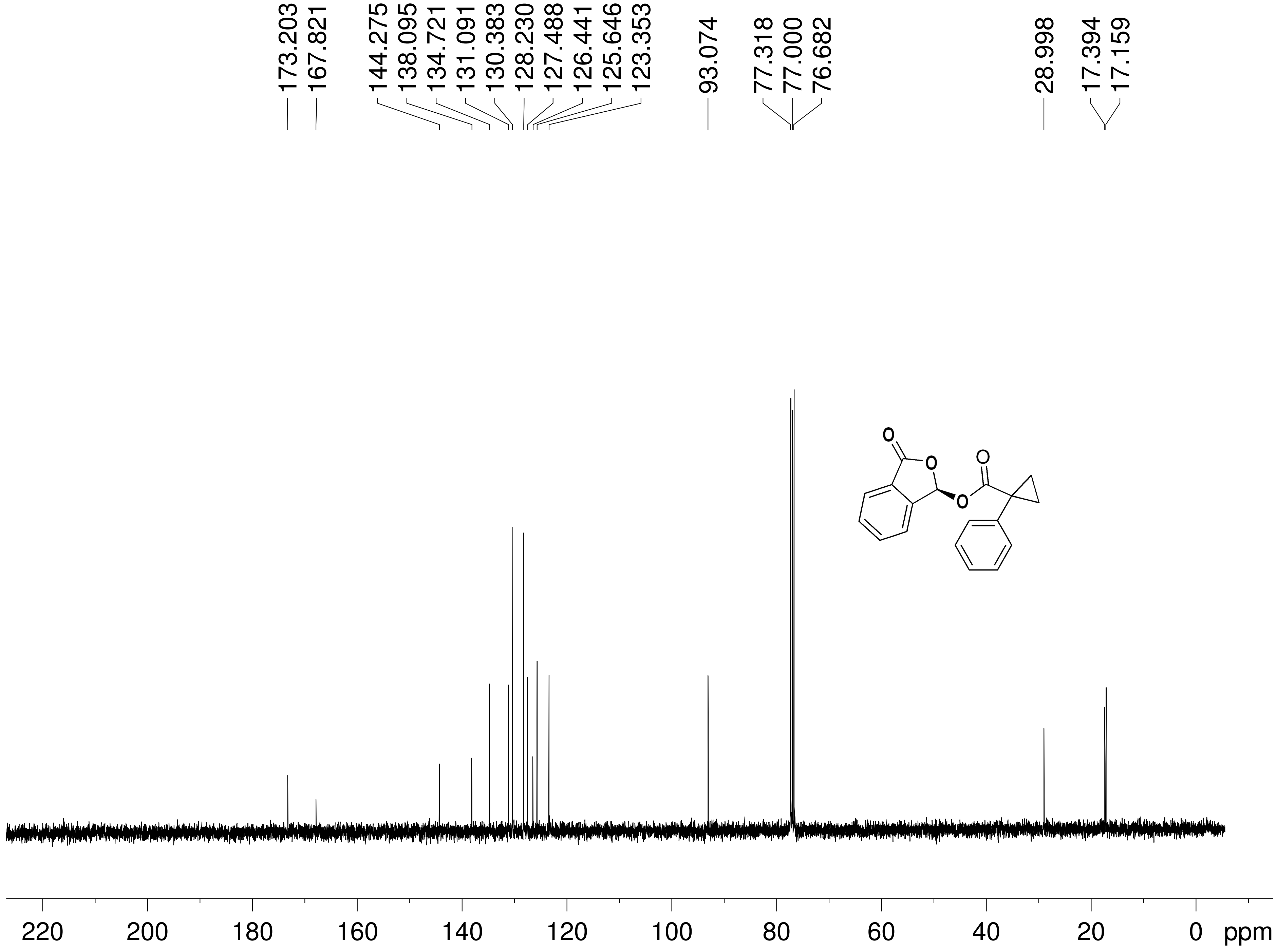

Supplementary Figure 86 ^{13}C NMR spectrum of **44**

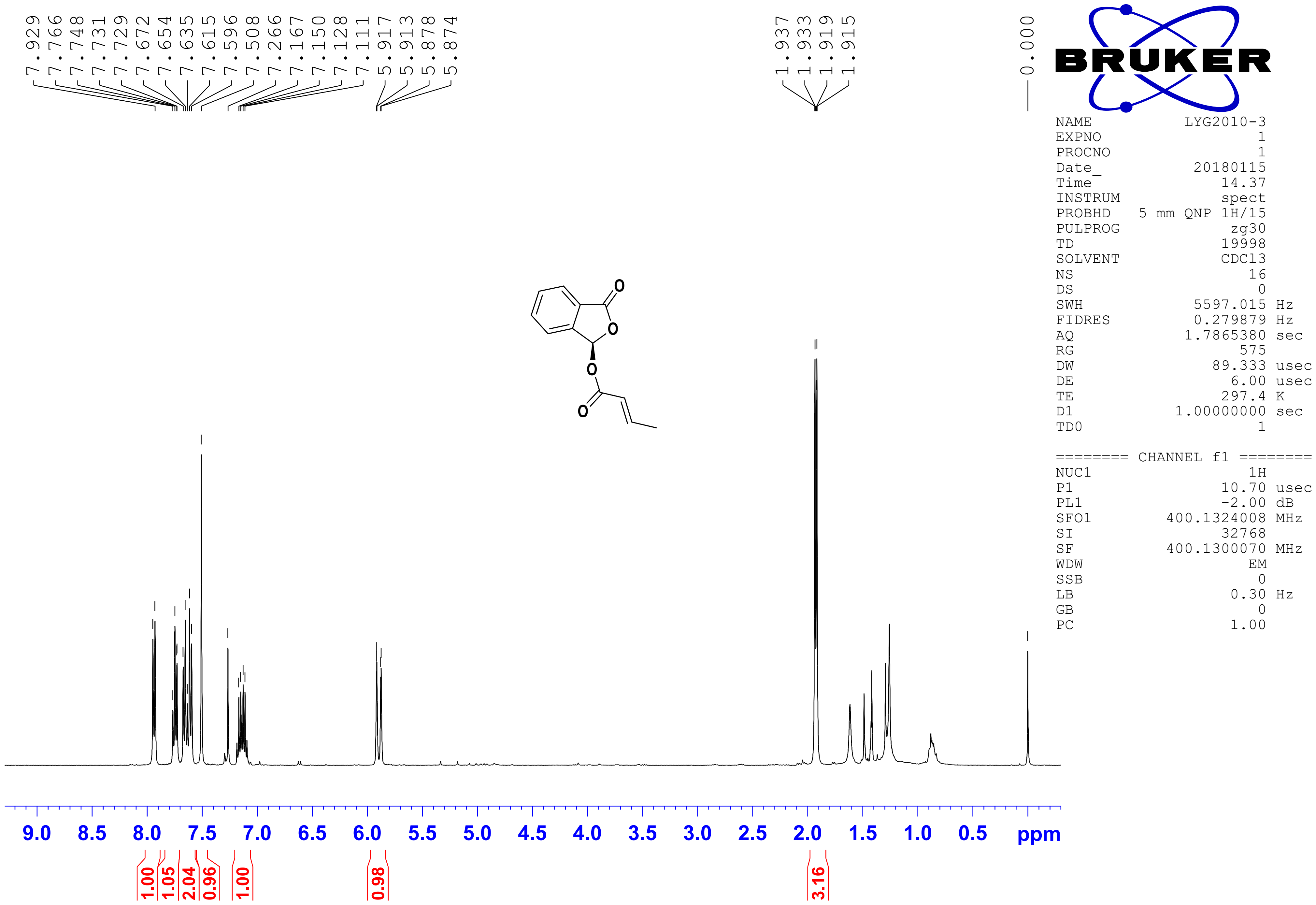

Supplementary Figure 87 ^{1}H NMR spectrum of **45**

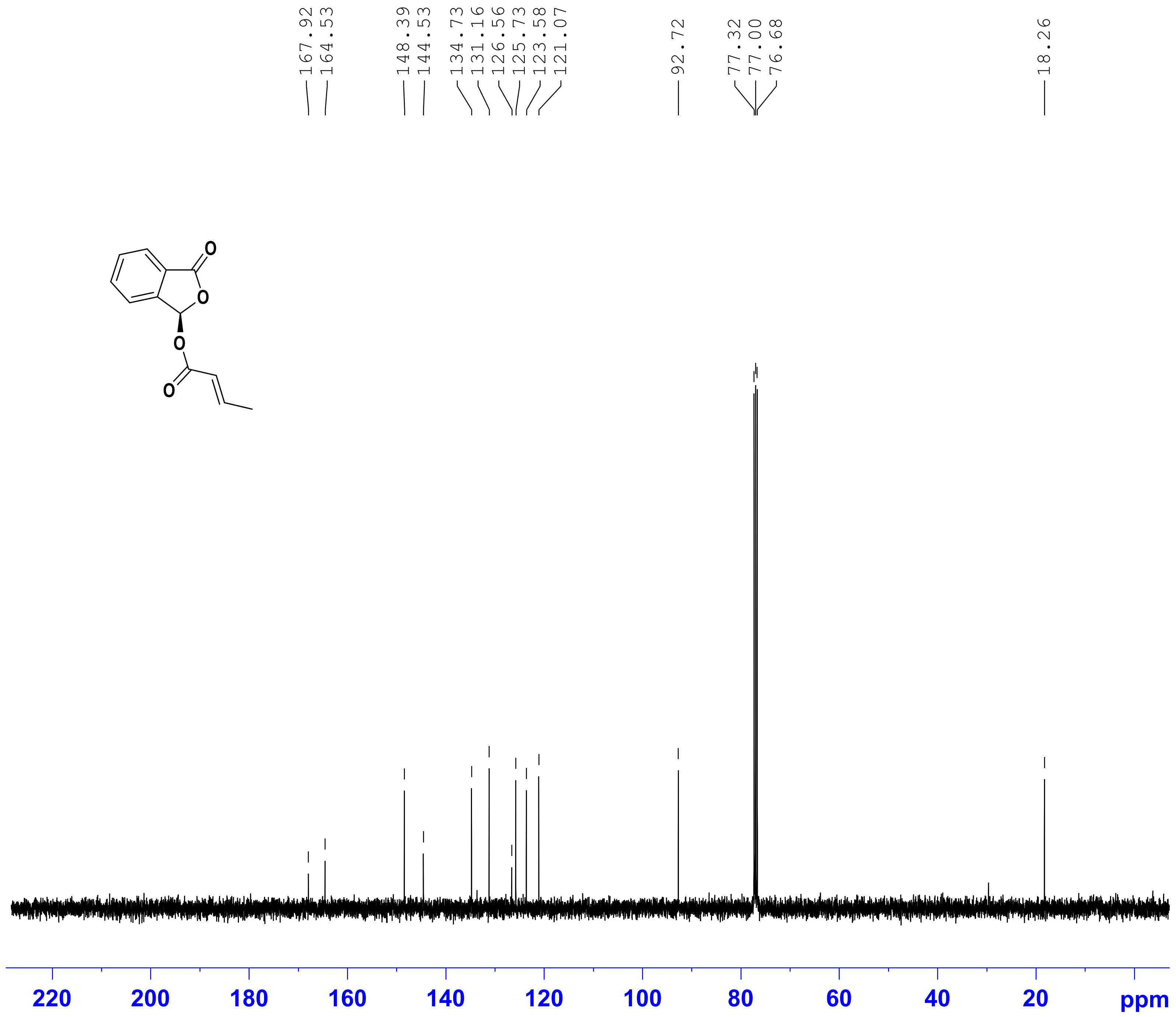

NAME	LYG2010-3
EXPNO	2
PROCNO	1
Date_	20180115
Time	14.38
INSTRUM	spect
PROBHD	5 mm QNP 1H/15
PULPROG	zgpg30
TD	65536
SOLVENT	CDCl3
NS	80
DS	0
SWH	23809.523 Hz
FIDRES	0.363304 Hz
AQ	1.3763061 sec
RG	32768
DW	21.000 usec
DE	6.00 usec
TE	297.5 K
D1	2.00000000 sec
d11	0.03000000 sec
DELTA	1.89999998 sec
TD0	1

======== CHANNEL f1 ========

NUC1	13C
P1	9.70 usec
PL1	-2.00 dB
SFO1	100.6238360 MHz

======== CHANNEL f2 ========

CPDPRG2	waltz16
NUC2	1H
PCPD2	80.00 usec
PL2	-2.00 dB
PL12	15.47 dB
PL13	18.00 dB
SFO2	400.1316000 MHz
SI	32768
SF	100.6127716 MHz
WDW	EM
SSB	0
LB	1.00 Hz
GB	0
PC	1.40

Supplementary Figure 88 ^{13}C NMR spectrum of **45**

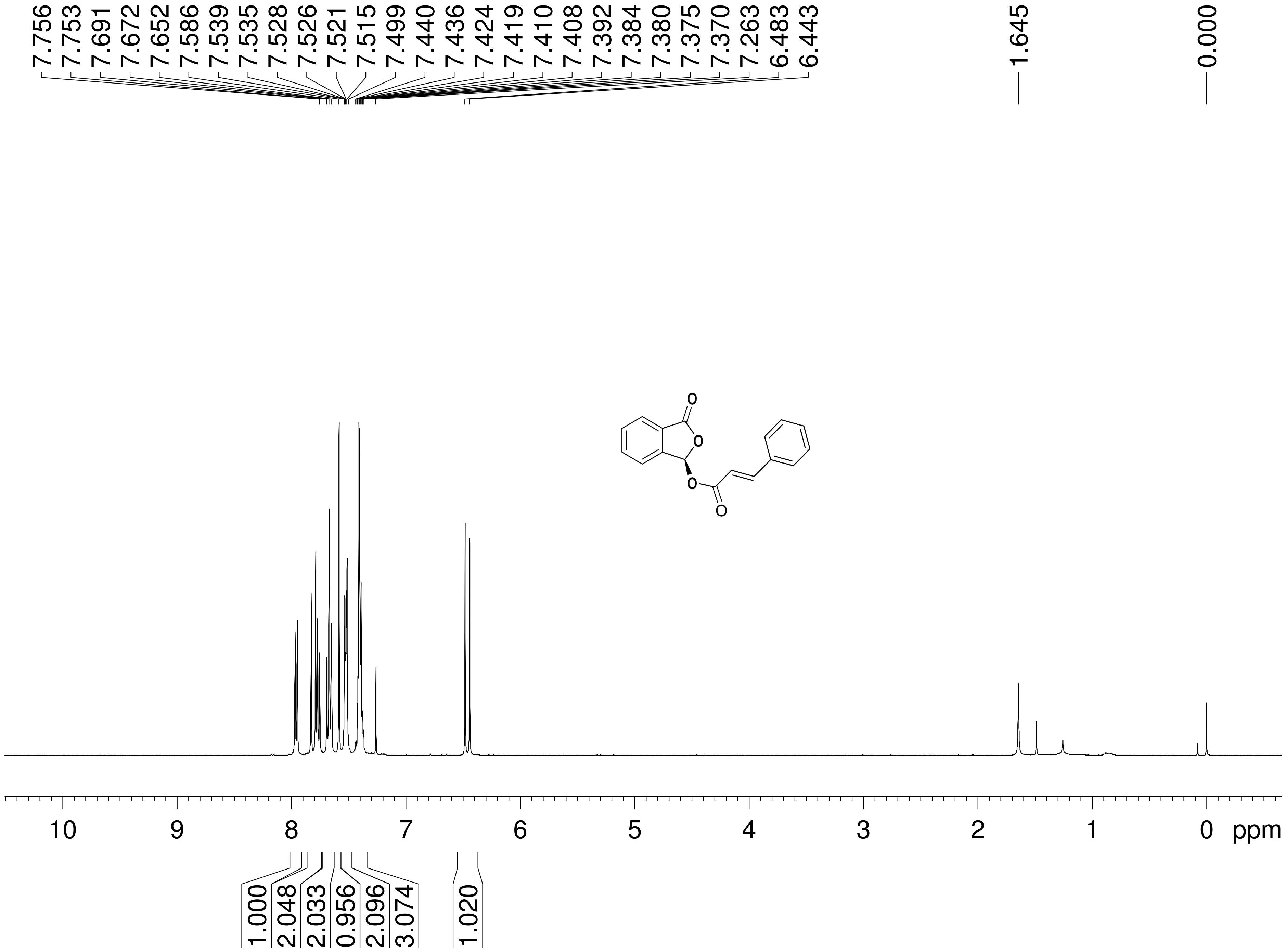

Supplementary Figure 89 ^{1}H NMR spectrum of **46**

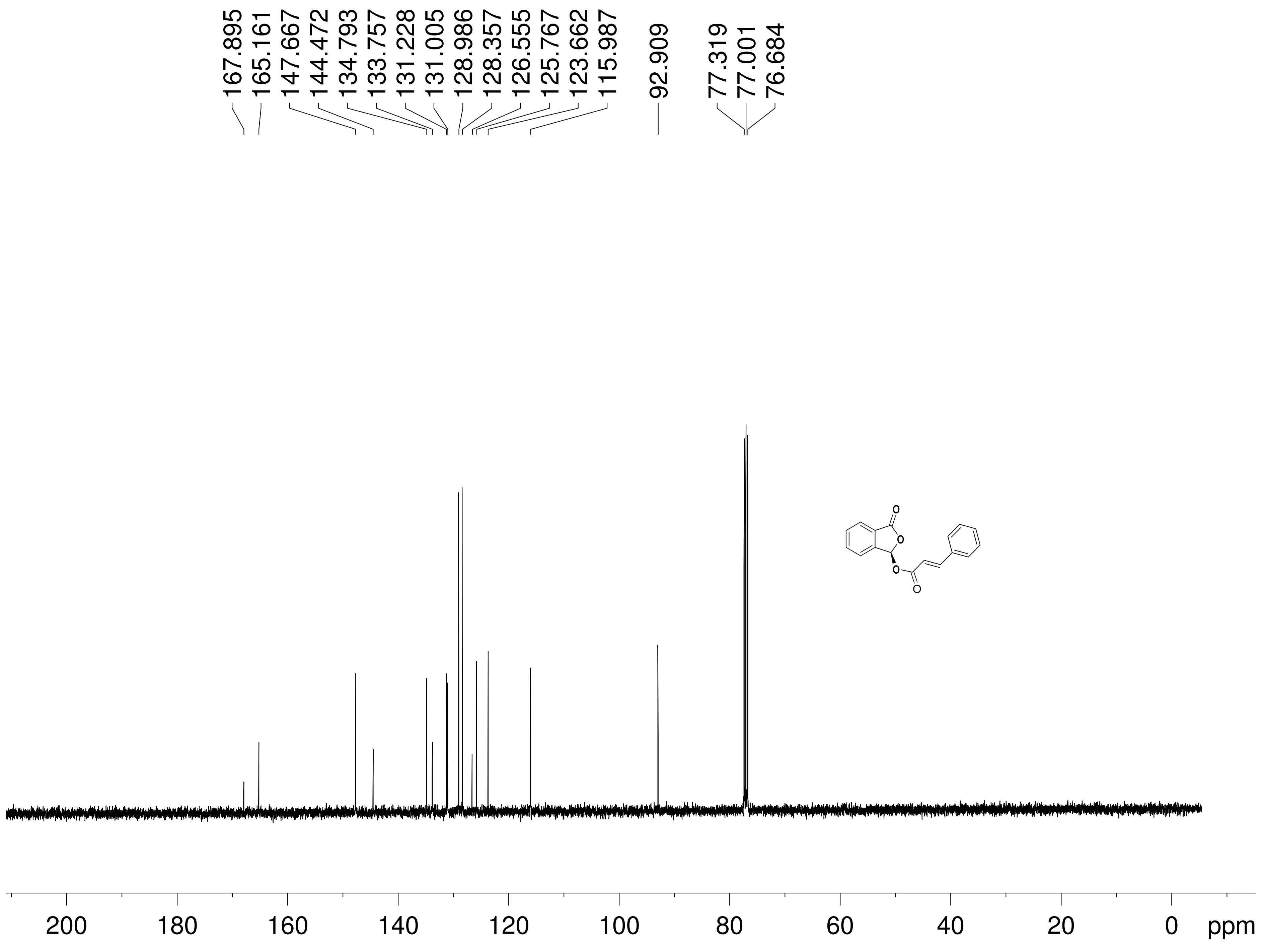

Supplementary Figure 90 ^{13}C NMR spectrum of **46**

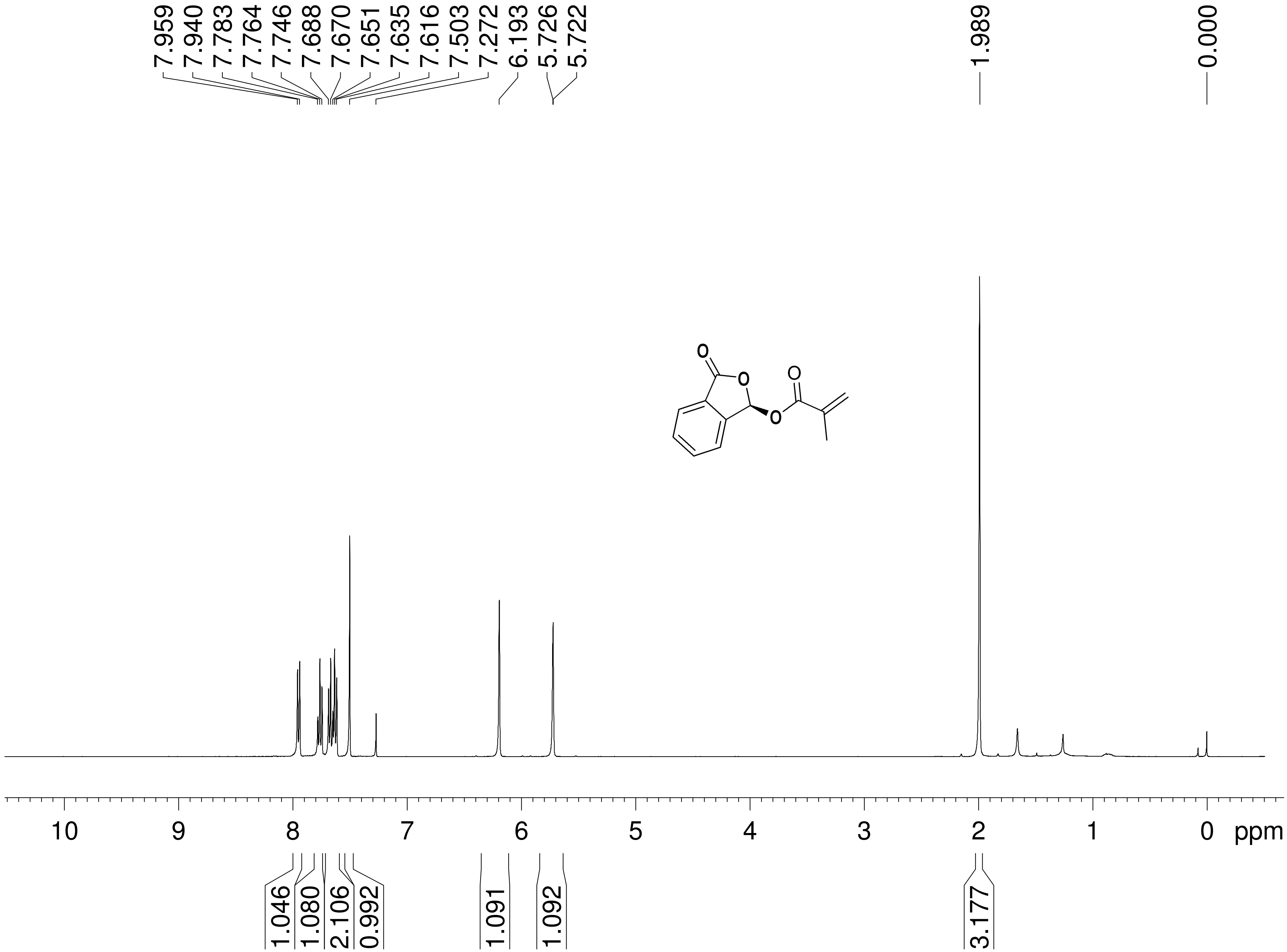

Supplementary Figure 91 ^{1}H NMR spectrum of **47**

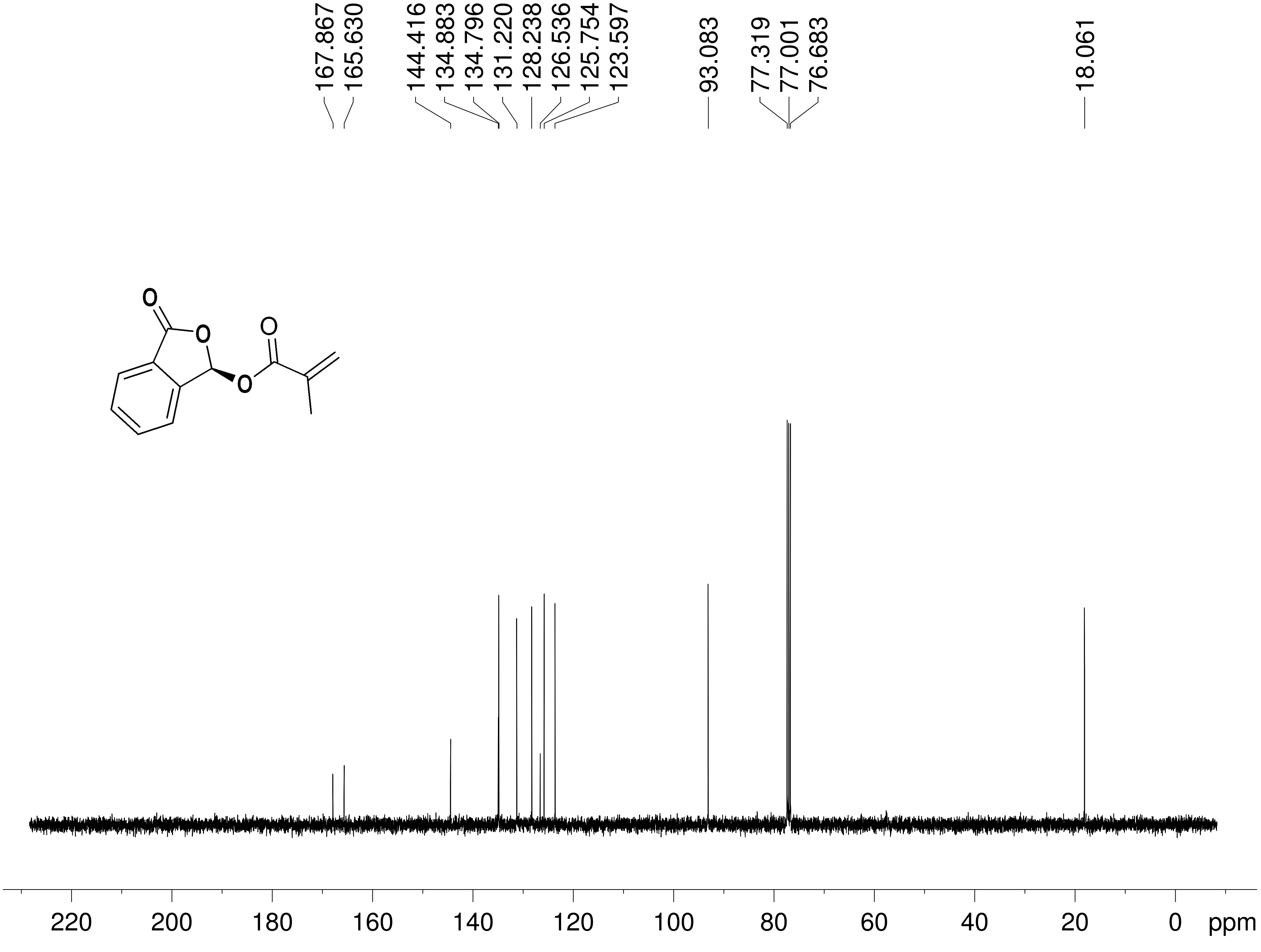

Supplementary Figure 92 ^{13}C NMR spectrum of **47**

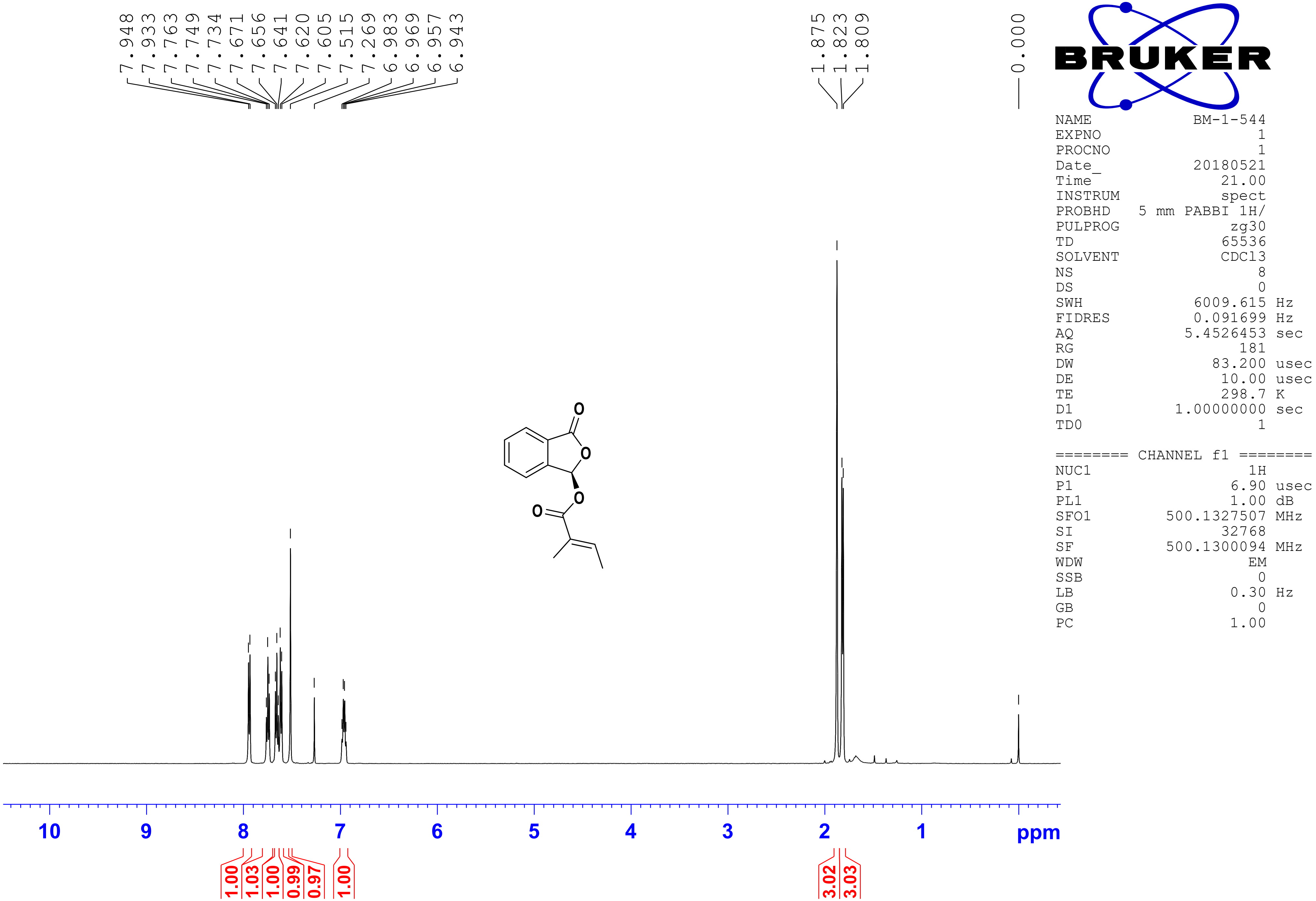

Supplementary Figure 93 ^{1}H NMR spectrum of **48**

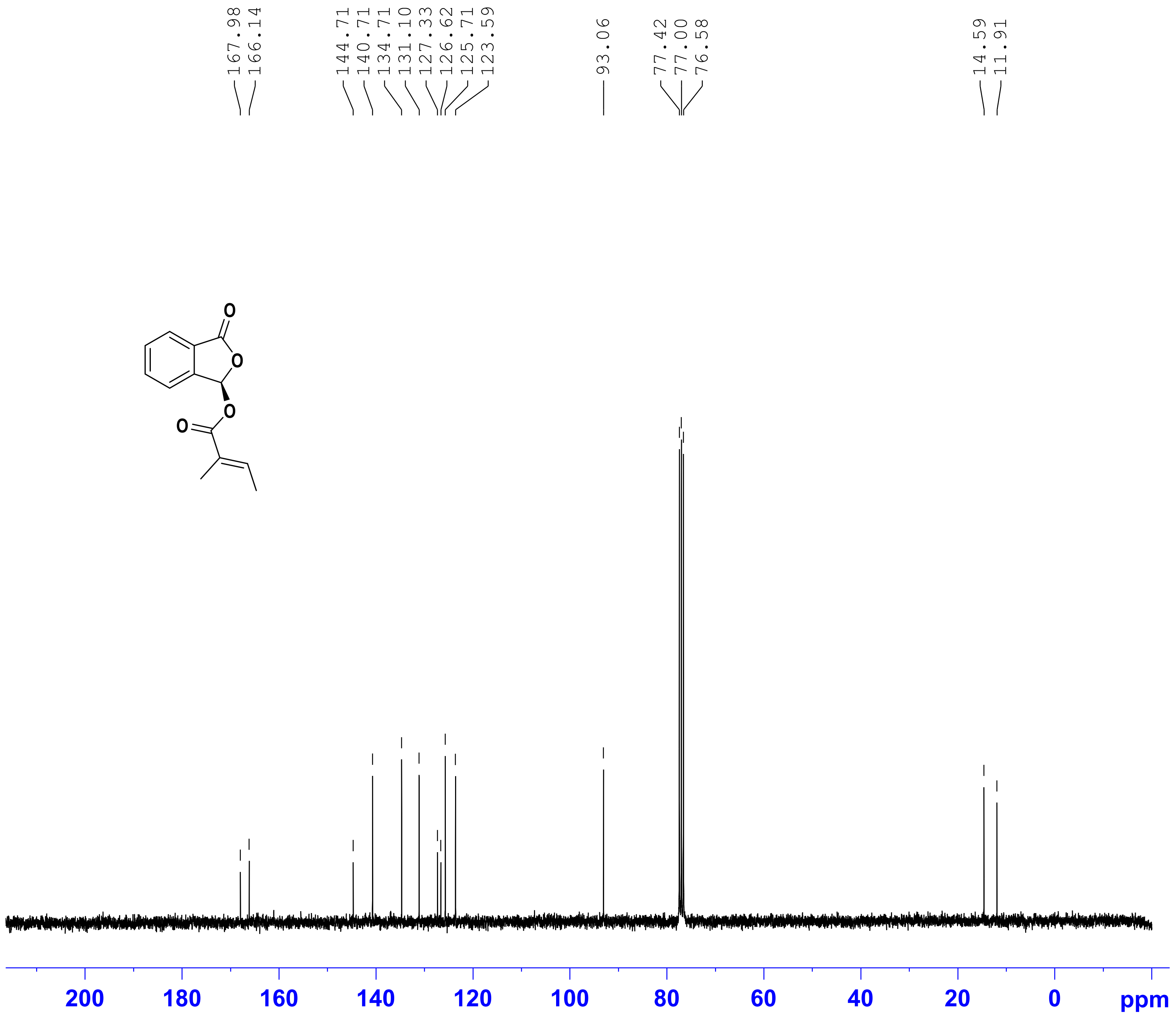

NAME	BM-1-544
EXPNO	2
PROCNO	1
Date_	20180521
Time	21.40
INSTRUM	spect
PROBHD	5 mm PABBO BB-
PULPROG	zgpg30
TD	65536
SOLVENT	CDCl3
NS	226
DS	0
SWH	18115.941 Hz
FIDRES	0.276427 Hz
AQ	1.8088436 sec
RG	4597.6
DW	27.600 usec
DE	6.00 usec
TE	300.5 K
D1	2.00000000 sec
d11	0.03000000 sec
DELTA	1.89999998 sec
TD0	1

======== CHANNEL f1 ========

NUC1	13C
P1	8.00 usec
PL1	-4.00 dB
SFO1	75.4752960 MHz

======== CHANNEL f2 ========

CPDPRG2	waltz16
NUC2	1H
PCPD2	80.00 usec
PL2	-1.00 dB
PL12	15.48 dB
PL13	19.23 dB
SFO2	300.1312000 MHz
SI	32768
SF	75.4677538 MHz
WDW	EM
SSB	0
LB	1.00 Hz
GB	0
PC	1.40

Supplementary Figure 94 ^{13}C NMR spectrum of **48**

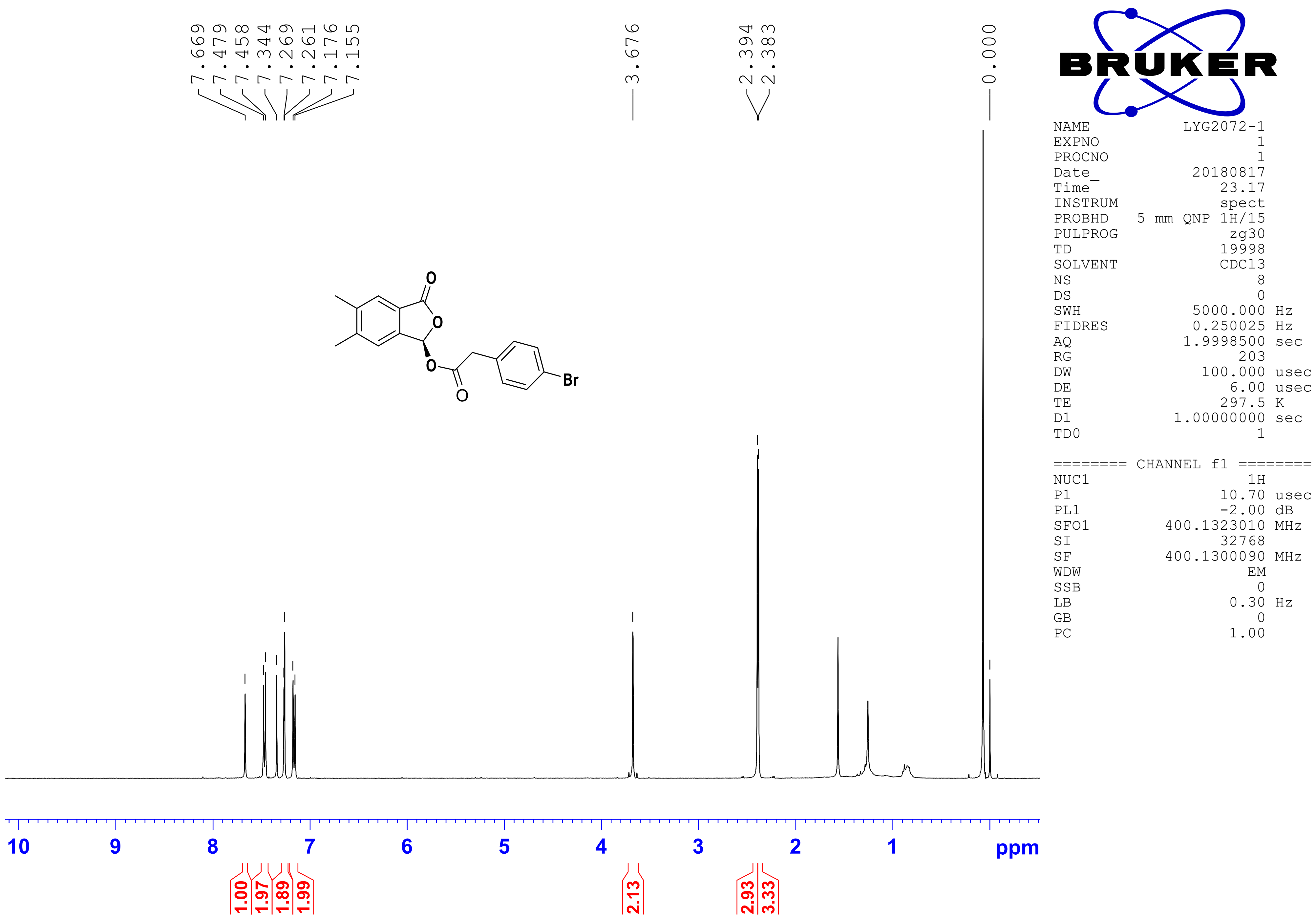

Supplementary Figure 95 ^{1}H NMR spectrum of **49**

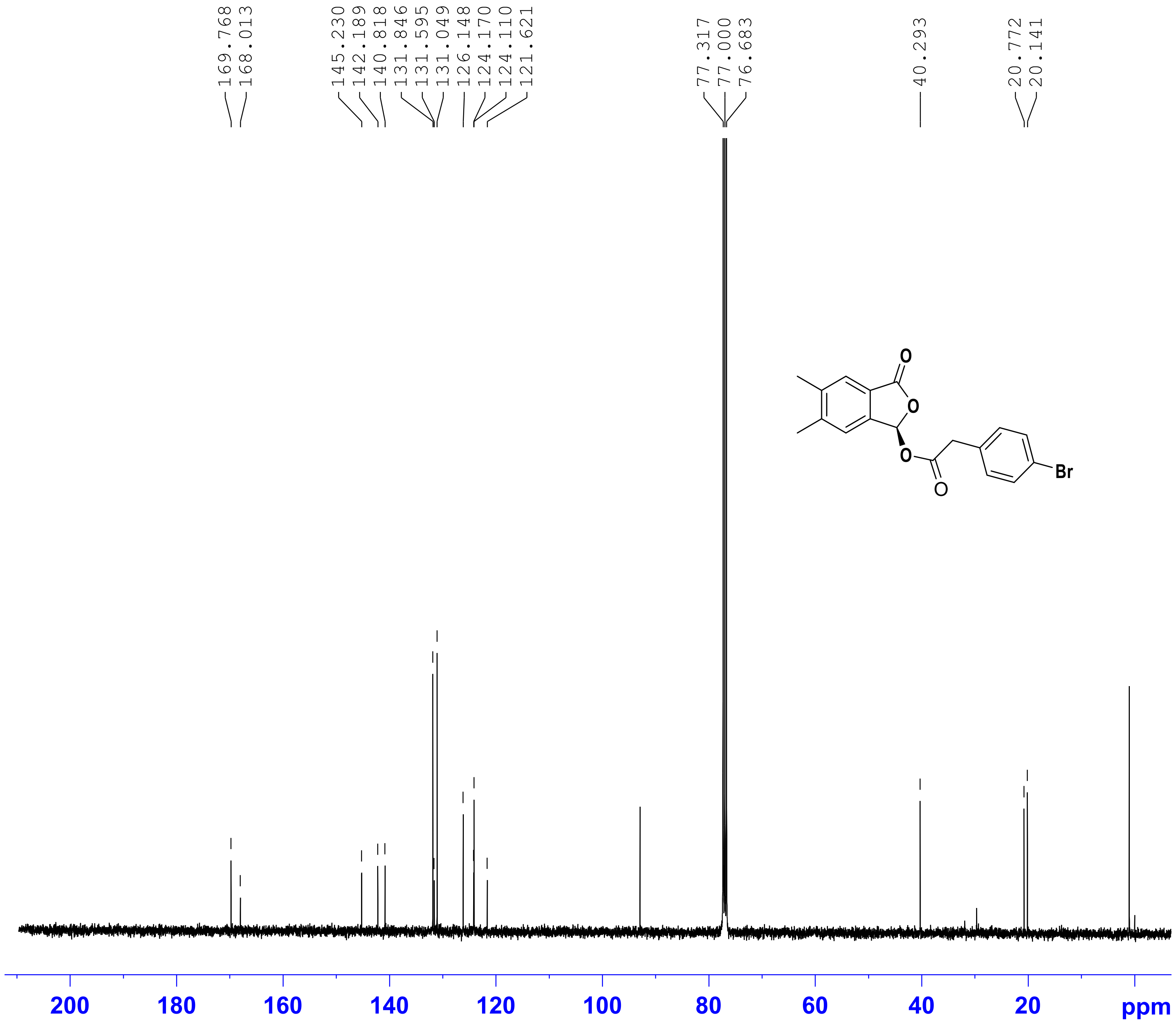


```
NAME            LYG2072-1
EXPNO                   2
PROCNO                  1
Date_            20180817
Time                23.19
INSTRUM             spect
PROBHD   5 mm QNP 1H/15
PULPROG           zgpg30
TD                 65536
SOLVENT            CDCl3
NS                  2000
DS                     0
SWH            22058.824 Hz
FIDRES          0.336591 Hz
AQ             1.4855326 sec
RG                 32768
DW                22.667 usec
DE                  6.00 usec
TE                 297.7 K
D1            2.00000000 sec
d11           0.03000000 sec
DELTA         1.89999998 sec
TD0                    1

======== CHANNEL f1 ========
NUC1                 13C
P1                  9.70 usec
PL1                -2.00 dB
SFO1         100.6228303 MHz

======== CHANNEL f2 ========
CPDPRG2          waltz16
NUC2                  1H
PCPD2              80.00 usec
PL2                -2.00 dB
PL12               15.47 dB
PL13               18.00 dB
SFO2         400.1316000 MHz
SI                 32768
SF           100.6127704 MHz
WDW                   EM
SSB                    0
LB                  1.00 Hz
GB                     0
PC                  1.40
```

Supplementary Figure 96 ^{13}C NMR spectrum of **49**

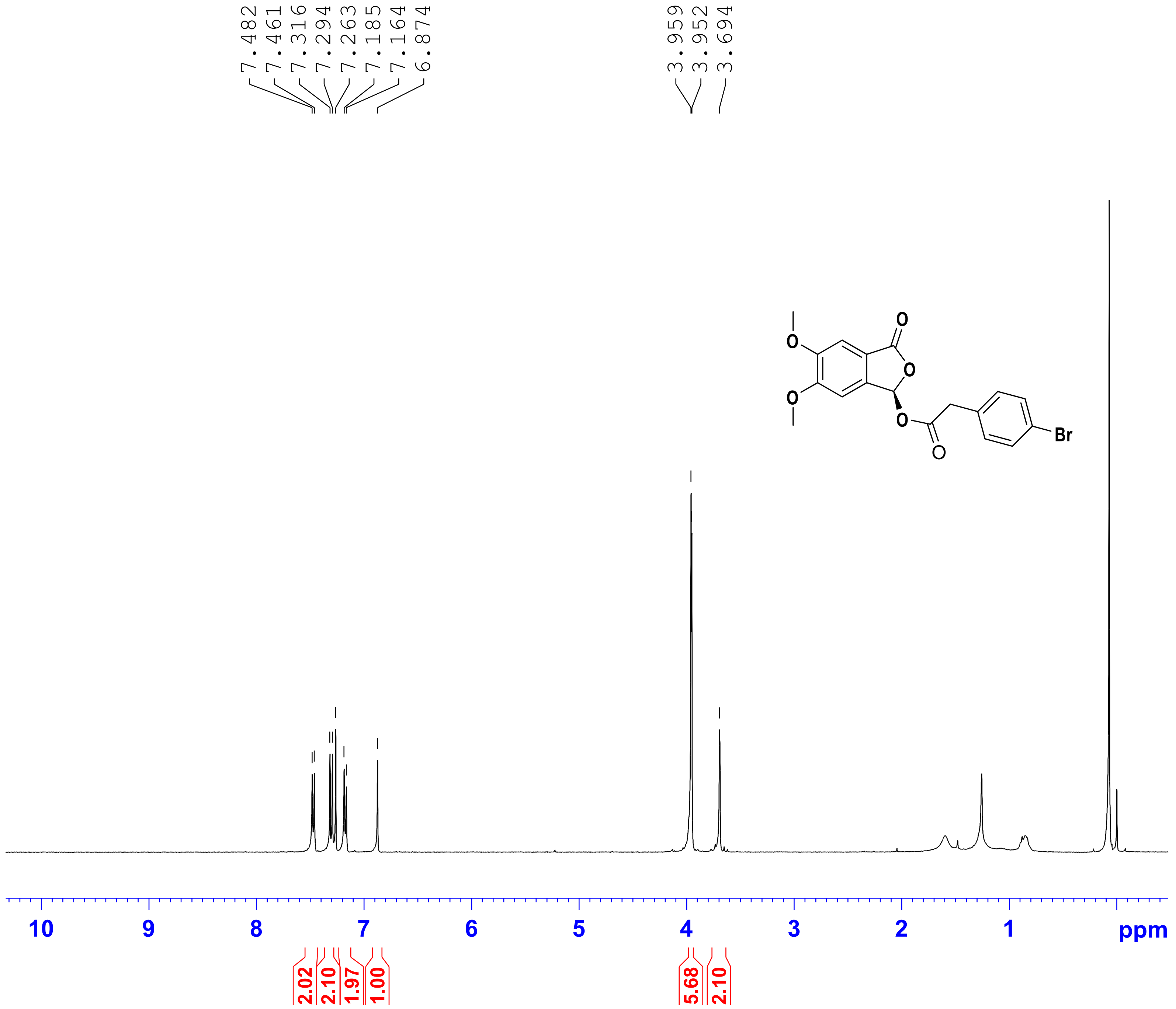

NAME	LYG2072-2
EXPNO	1
PROCNO	1
Date_	20180818
Time	1.24
INSTRUM	spect
PROBHD	5 mm QNP 1H/15
PULPROG	zg30
TD	19998
SOLVENT	CDCl3
NS	8
DS	0
SWH	5000.000 Hz
FIDRES	0.250025 Hz
AQ	1.9998500 sec
RG	203
DW	100.000 usec
DE	6.00 usec
TE	297.6 K
D1	1.00000000 sec
TD0	1

======== CHANNEL f1 ========

NUC1	1H
P1	10.70 usec
PL1	-2.00 dB
SFO1	400.1323010 MHz
SI	32768
SF	400.1300078 MHz
WDW	EM
SSB	0
LB	0.30 Hz
GB	0
PC	1.00

Supplementary Figure 97 ^{1}H NMR spectrum of **50**

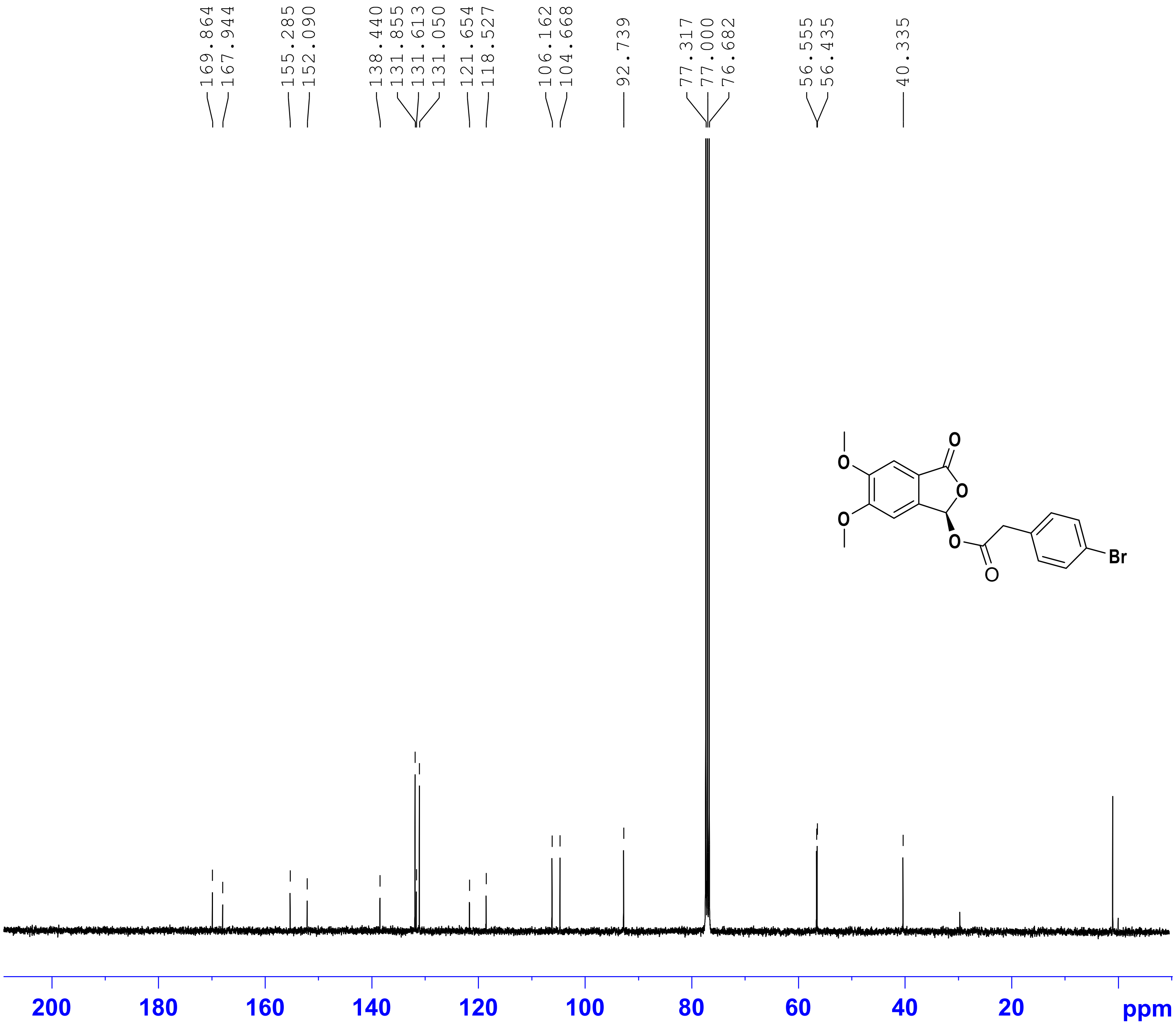


```
NAME              LYG2072-2
EXPNO                     2
PROCNO                    1
Date_              20180818
Time                   3.24
INSTRUM               spect
PROBHD   5 mm QNP 1H/15
PULPROG             zgpg30
TD                    65536
SOLVENT               CDCl3
NS                     2000
DS                        0
SWH               22058.824 Hz
FIDRES             0.336591 Hz
AQ                1.4855326 sec
RG                    32768
DW                   22.667 usec
DE                     6.00 usec
TE                    298.3 K
D1               2.00000000 sec
d11              0.03000000 sec
DELTA            1.89999998 sec
TD0                       1

======== CHANNEL f1 ========
NUC1                    13C
P1                     9.70 usec
PL1                   -2.00 dB
SFO1            100.6228303 MHz

======== CHANNEL f2 ========
CPDPRG2             waltz16
NUC2                     1H
PCPD2                 80.00 usec
PL2                   -2.00 dB
PL12                  15.47 dB
PL13                  18.00 dB
SFO2            400.1316000 MHz
SI                    32768
SF              100.6127708 MHz
WDW                      EM
SSB                       0
LB                     1.00 Hz
GB                        0
PC                     1.40
```

Supplementary Figure 98 ^{13}C NMR spectrum of **50**

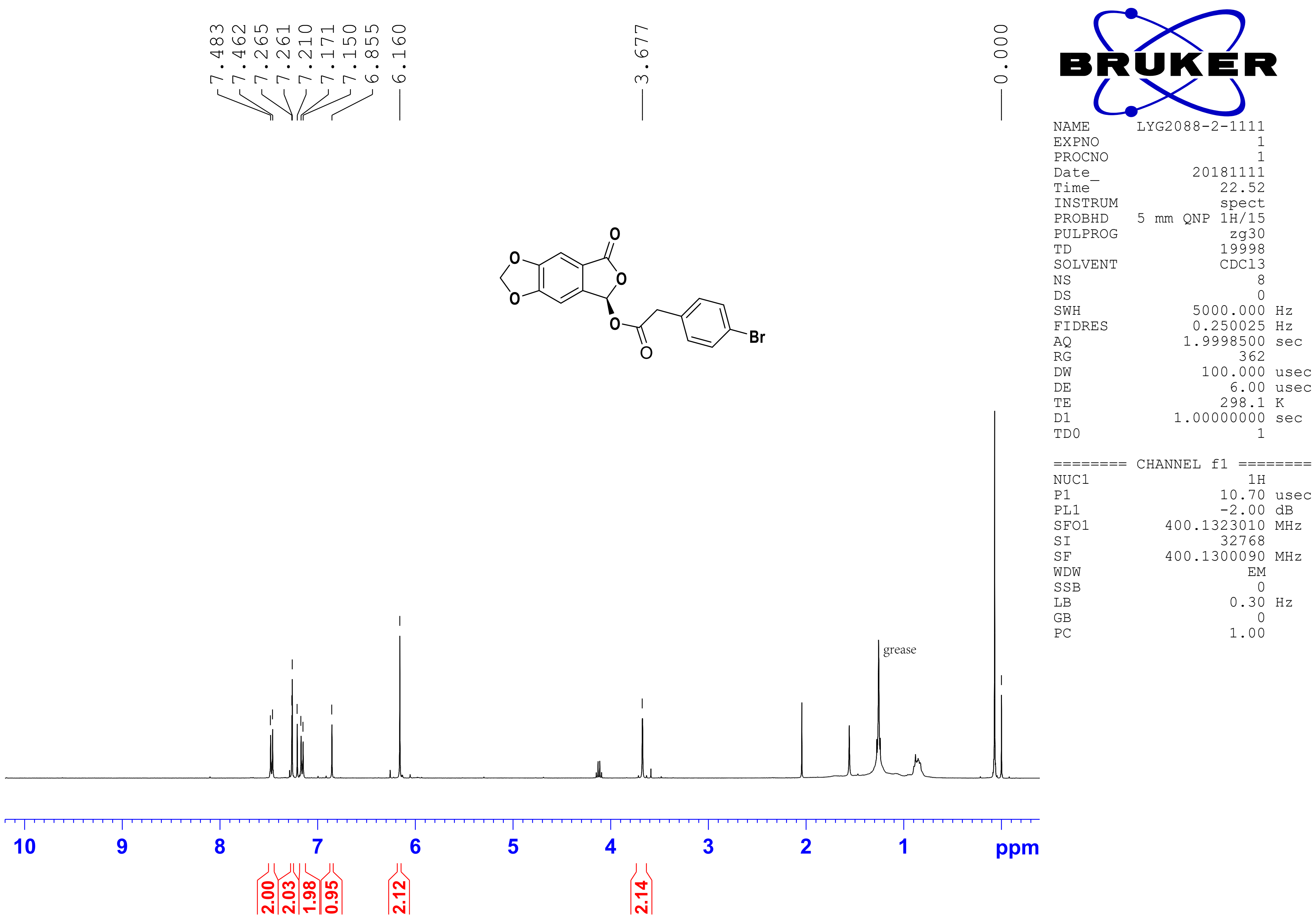

Supplementary Figure 99 ^{1}H NMR spectrum of **51**

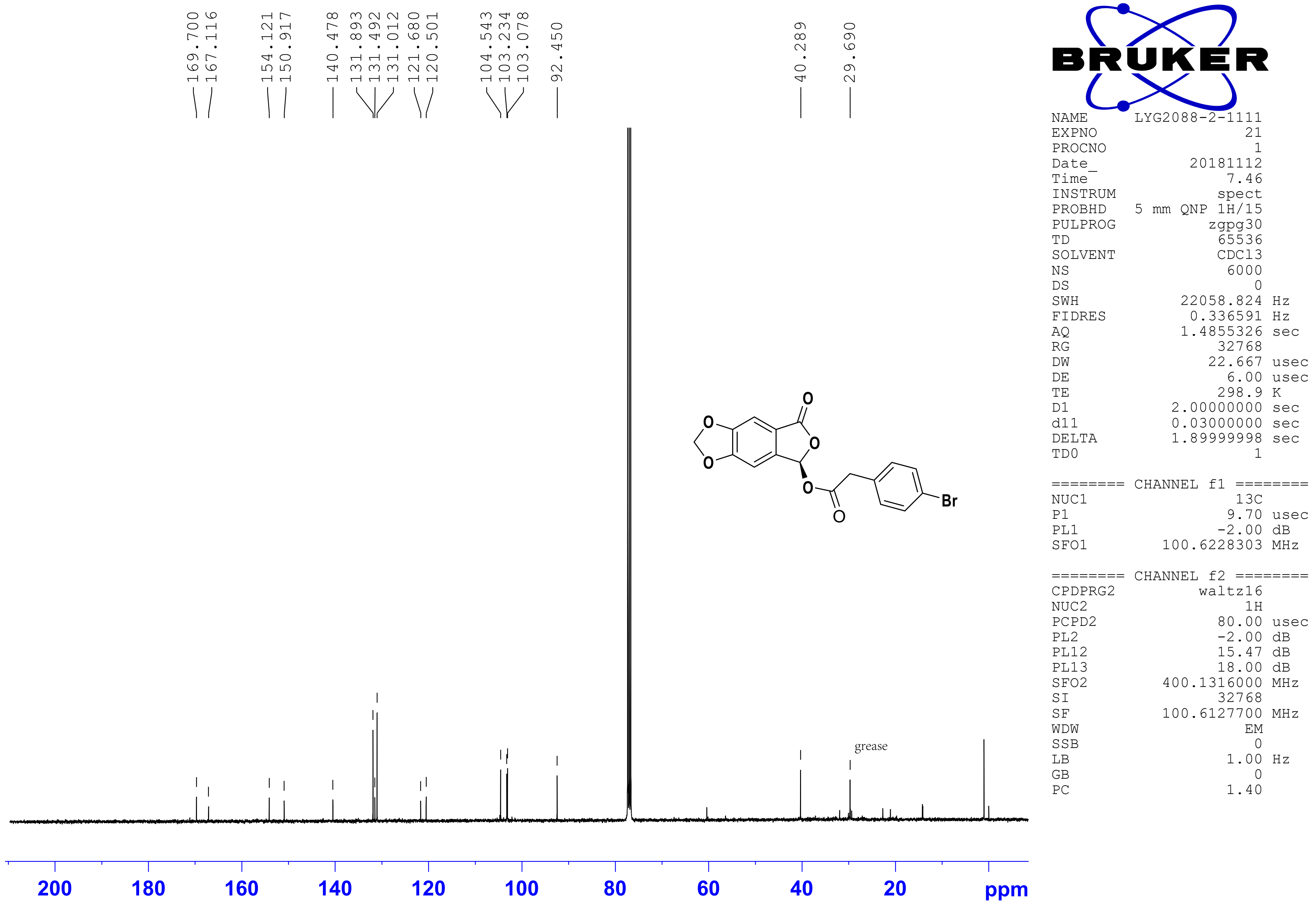

Supplementary Figure 100 ^{13}C NMR spectrum of **51**

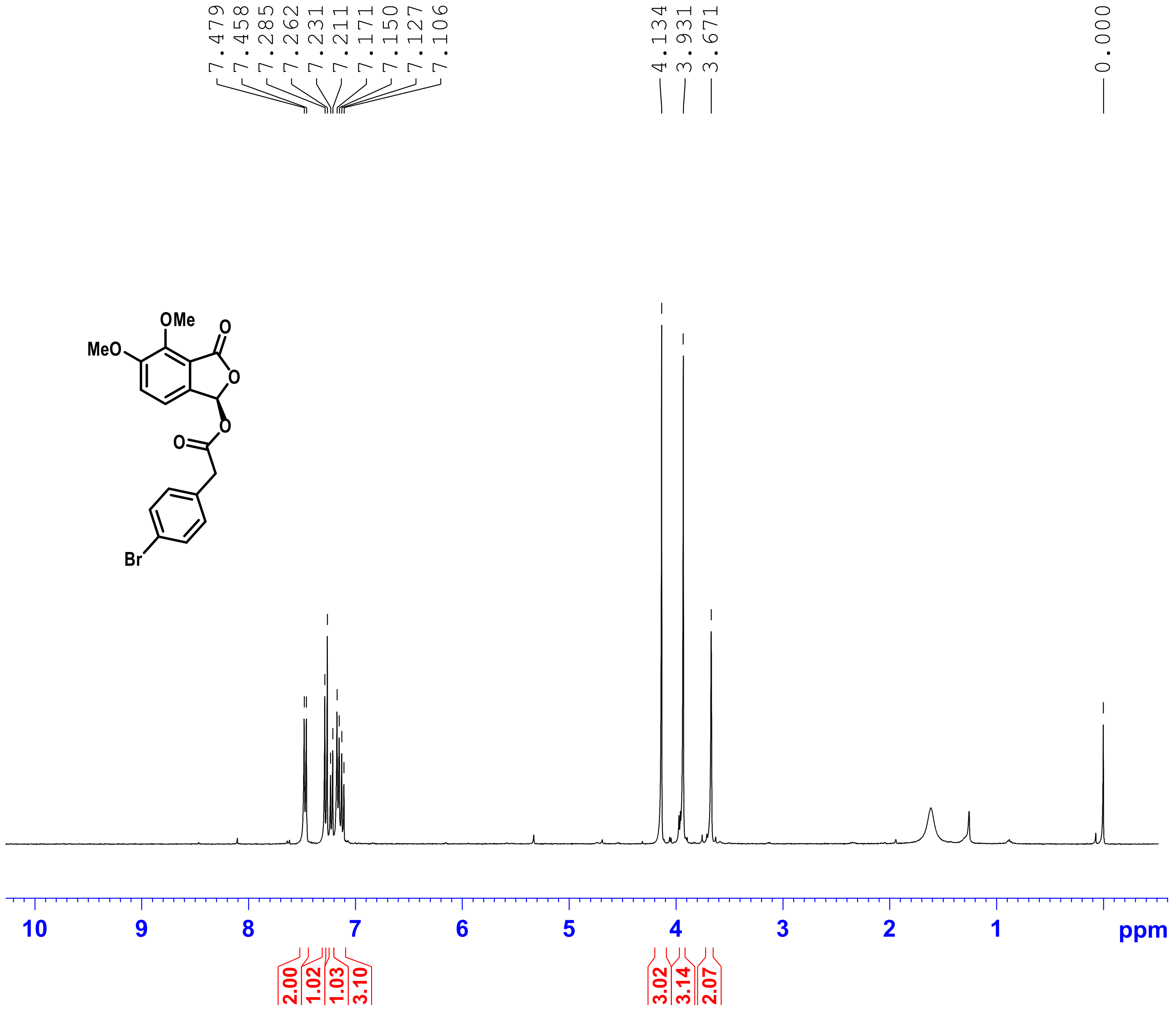

NAME LYG2102-6-2-0115
EXPNO 1
PROCNO 1
Date_ 20190115
Time 21.56
INSTRUM spect
PROBHD 5 mm QNP 1H/15
PULPROG zg30
TD 19998
SOLVENT CDCl3
NS 8
DS 0
SWH 5000.000 Hz
FIDRES 0.250025 Hz
AQ 1.9998500 sec
RG 645
DW 100.000 usec
DE 6.00 usec
TE 298.1 K
D1 1.00000000 sec
TD0 1

======== CHANNEL f1 ========
NUC1 1H
P1 10.70 usec
PL1 -2.00 dB
SFO1 400.1323010 MHz
SI 32768
SF 400.1300082 MHz
WDW EM
SSB 0
LB 0.30 Hz
GB 0
PC 1.00

Supplementary Figure 101 ^{1}H NMR spectrum of **52a**

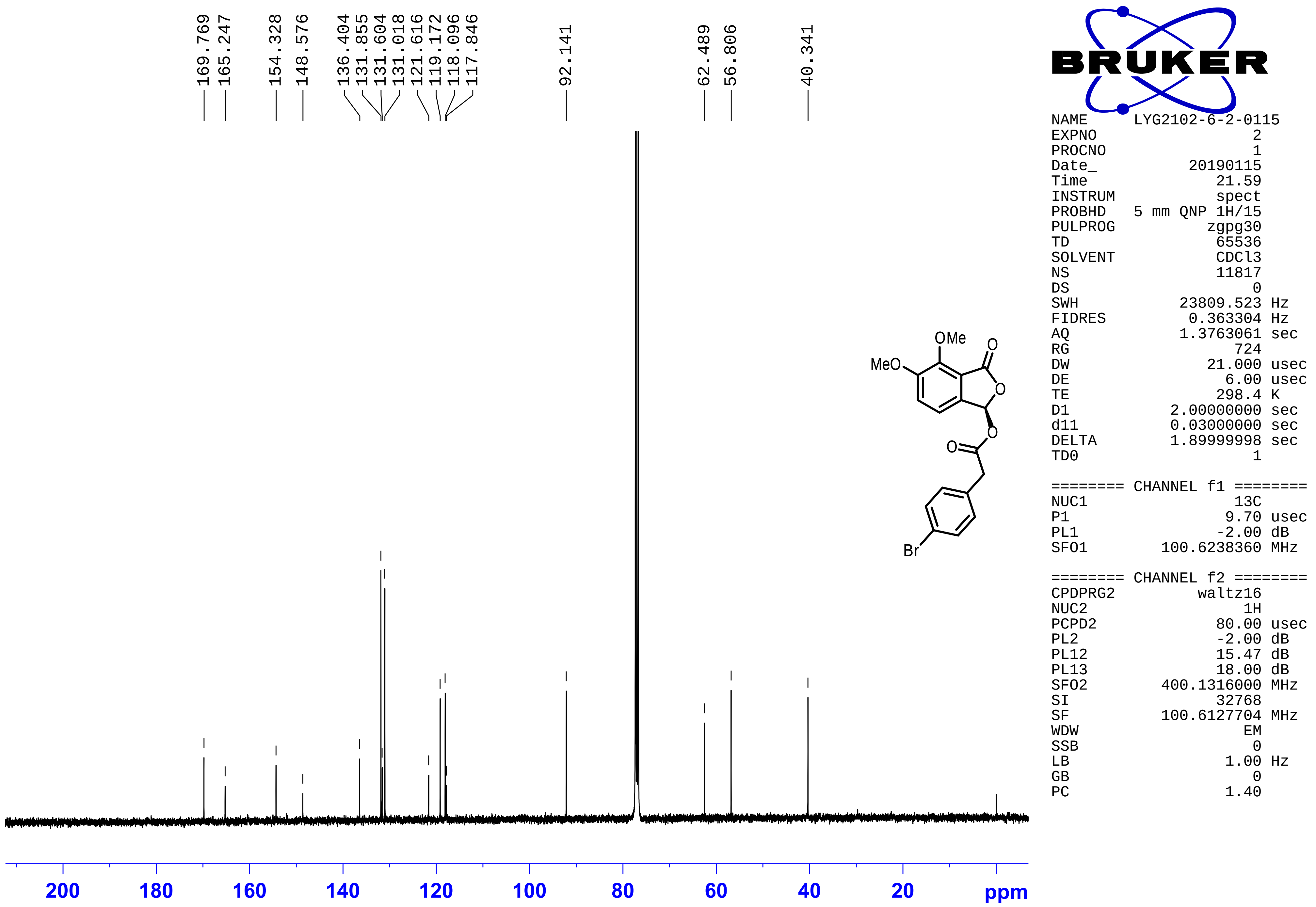

Supplementary Figure 102 ^{13}C NMR spectrum of **52a**

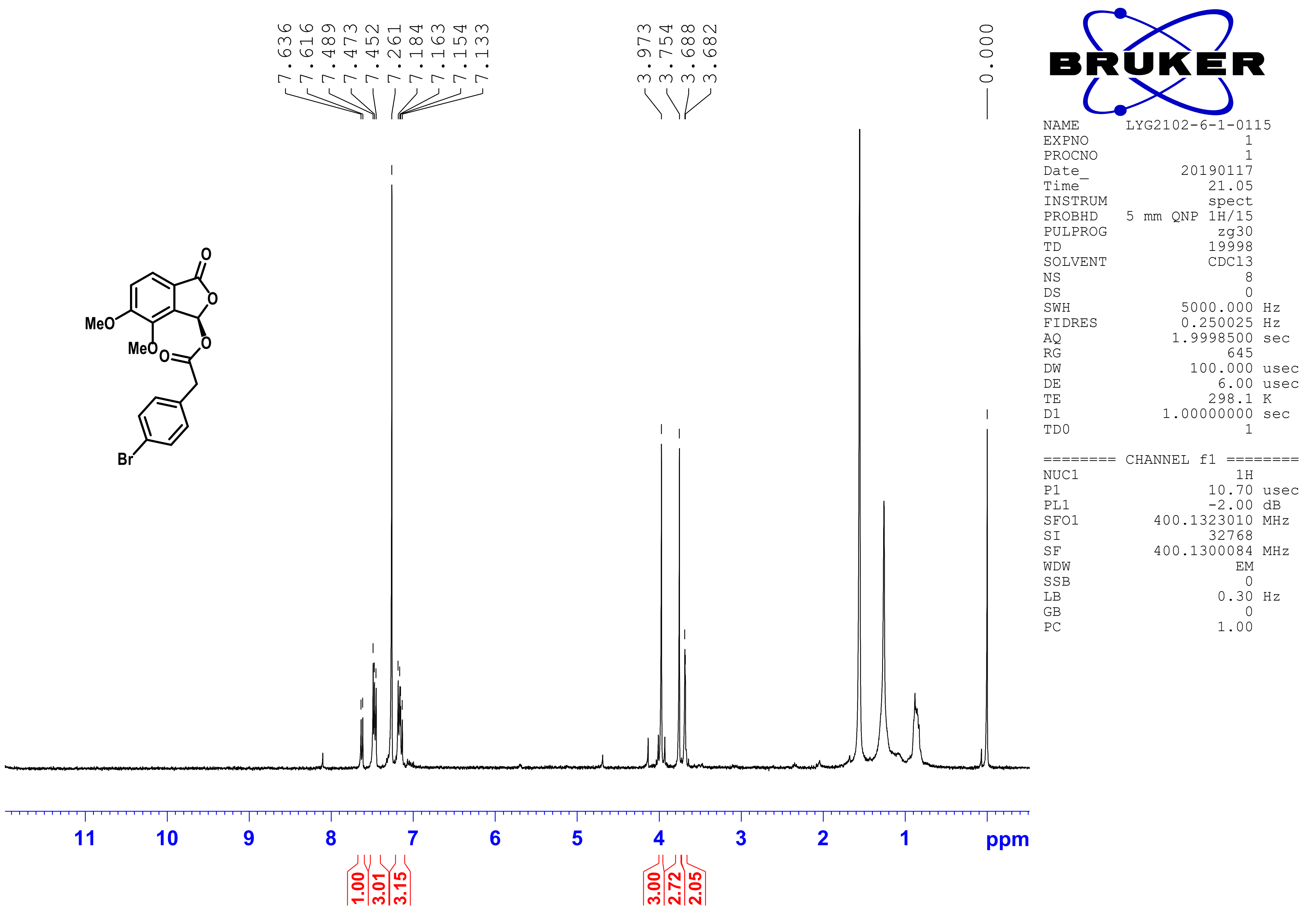

Supplementary Figure 103 ^{1}H NMR spectrum of **52b**

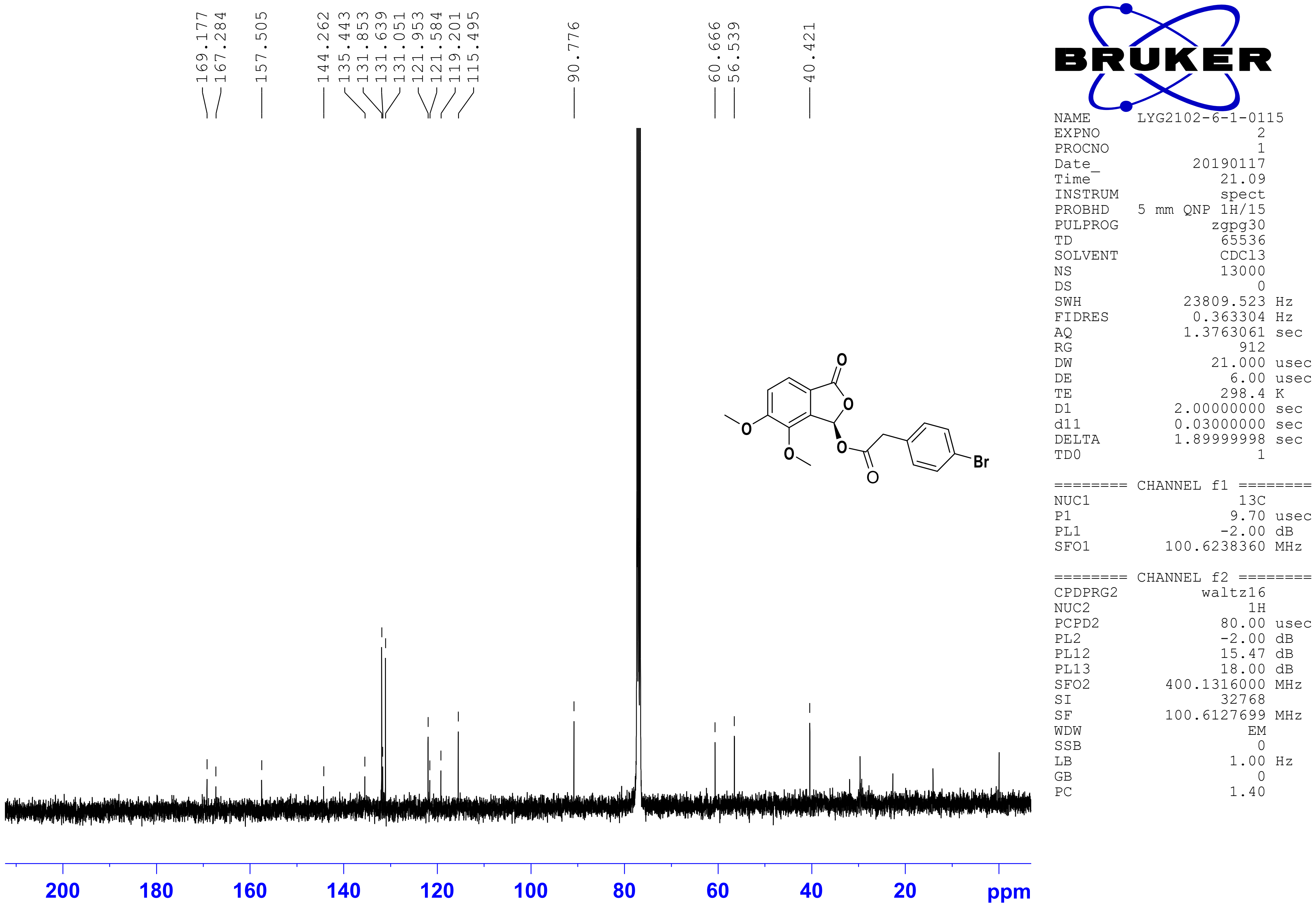

Supplementary Figure 104 ^{13}C NMR spectrum of **52b**

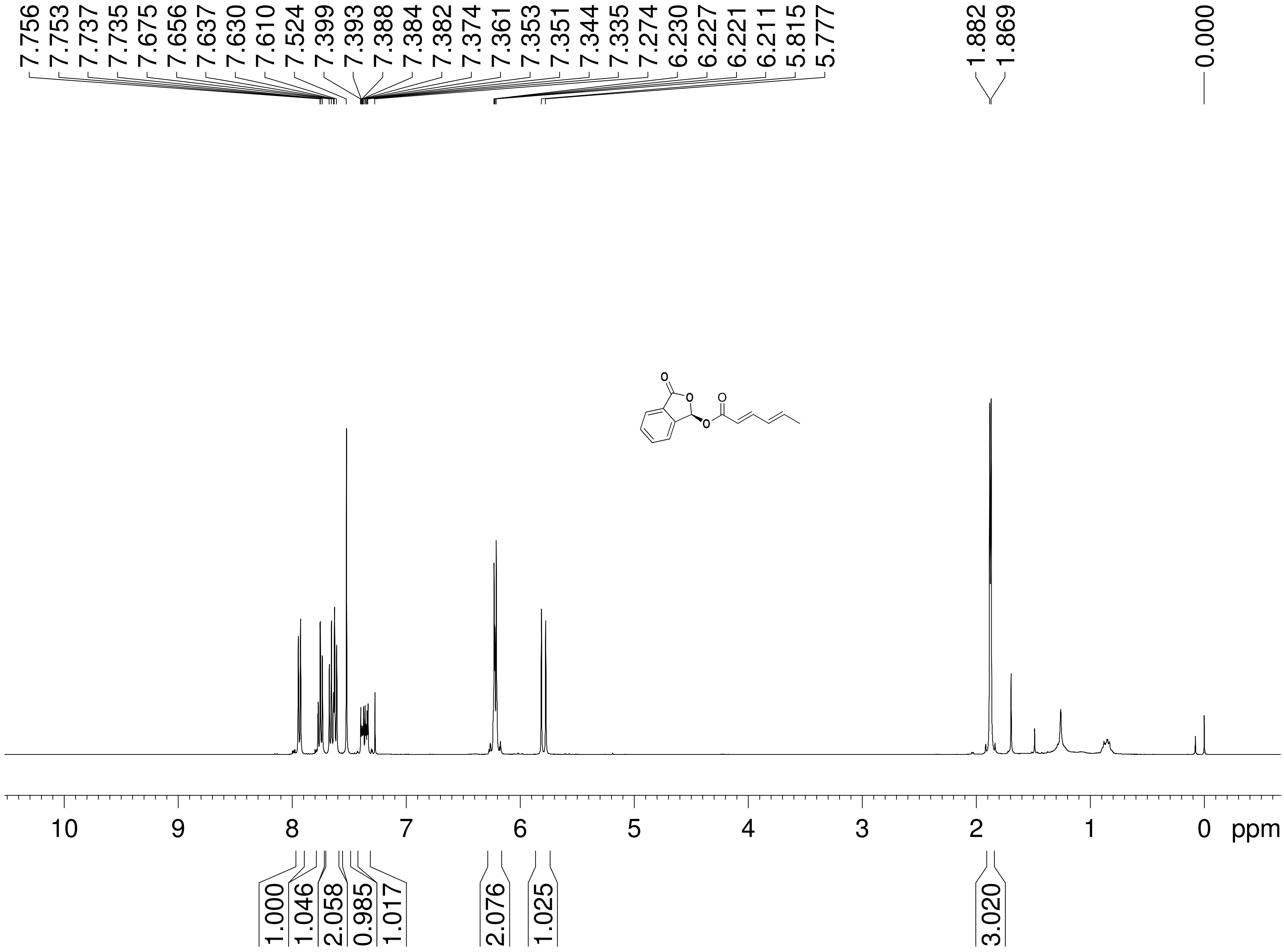

Supplementary Figure 105 ^{1}H NMR spectrum of **53**

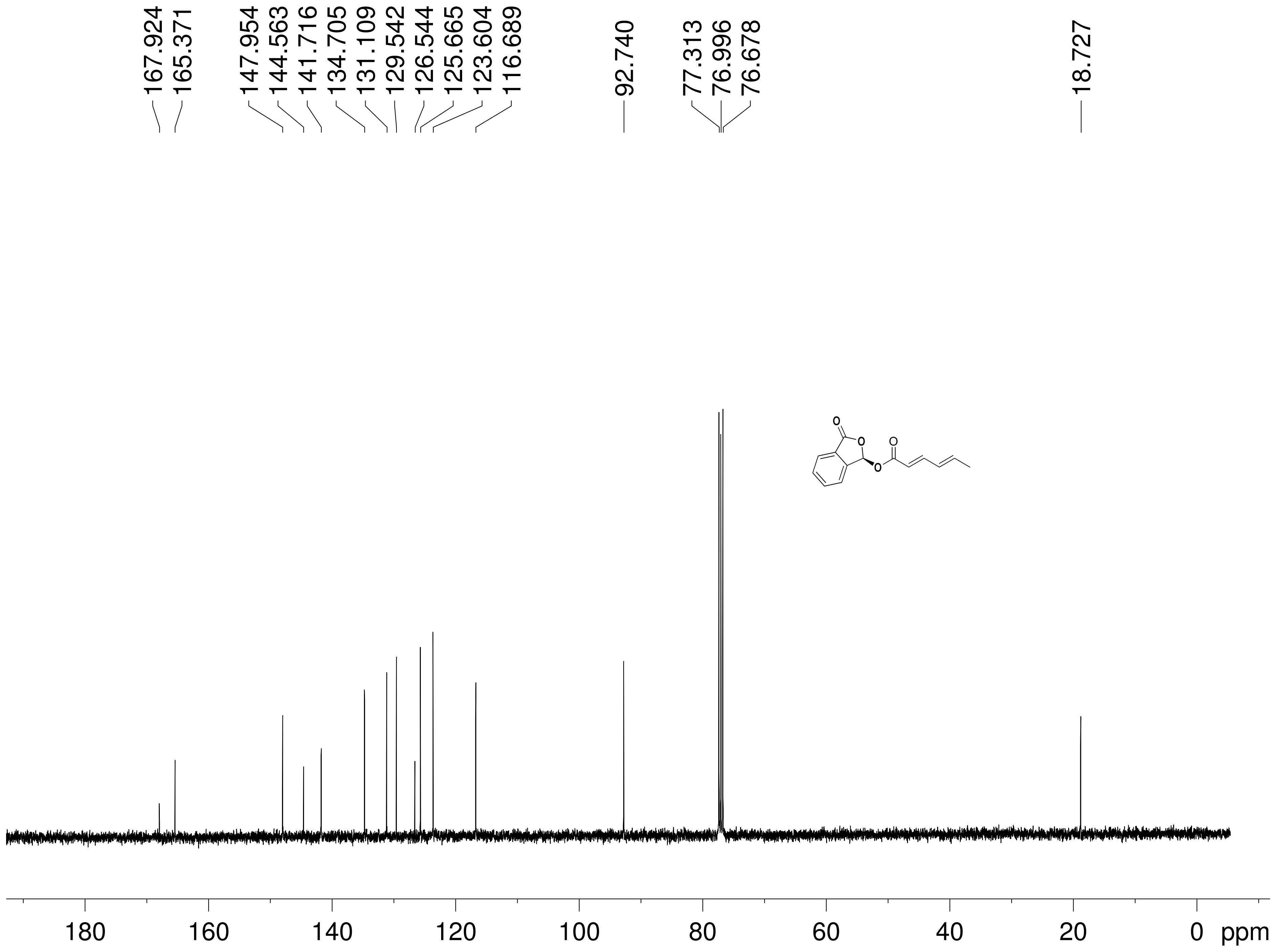

Supplementary Figure 106 ^{13}C NMR spectrum of **53**

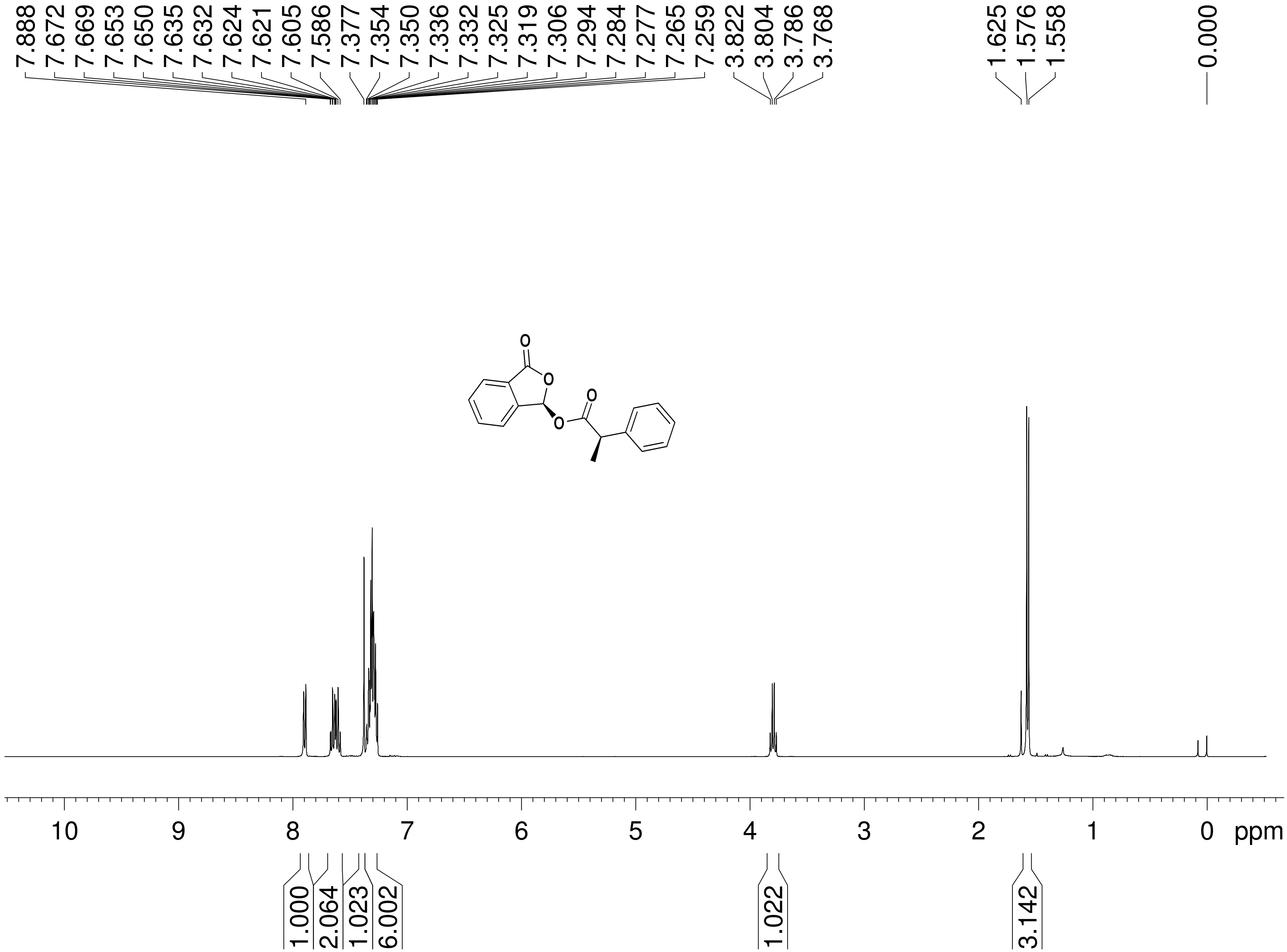

Supplementary Figure 107 ^{1}H NMR spectrum of **54**

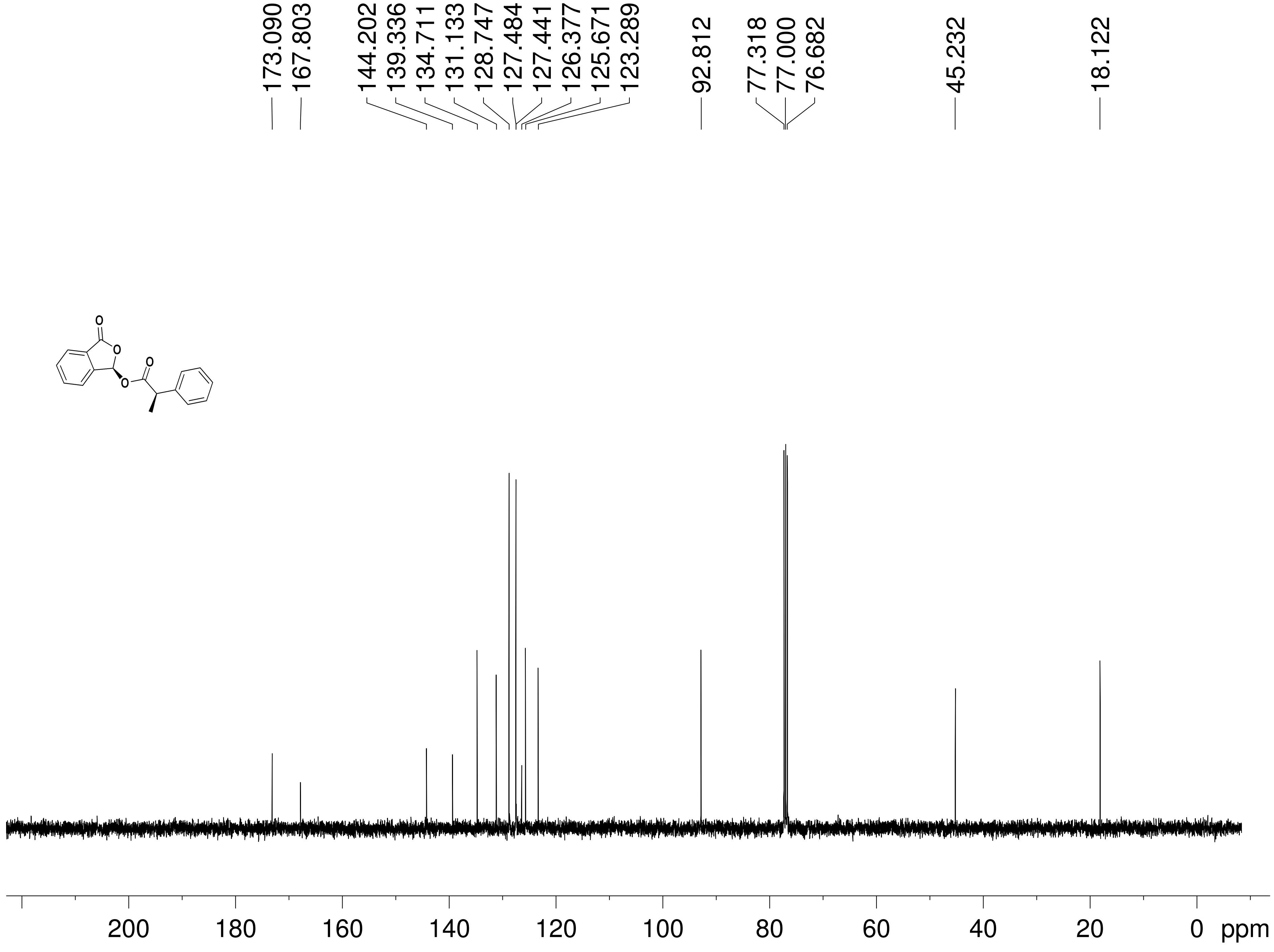

Supplementary Figure 108 ^{13}C NMR spectrum of **54**

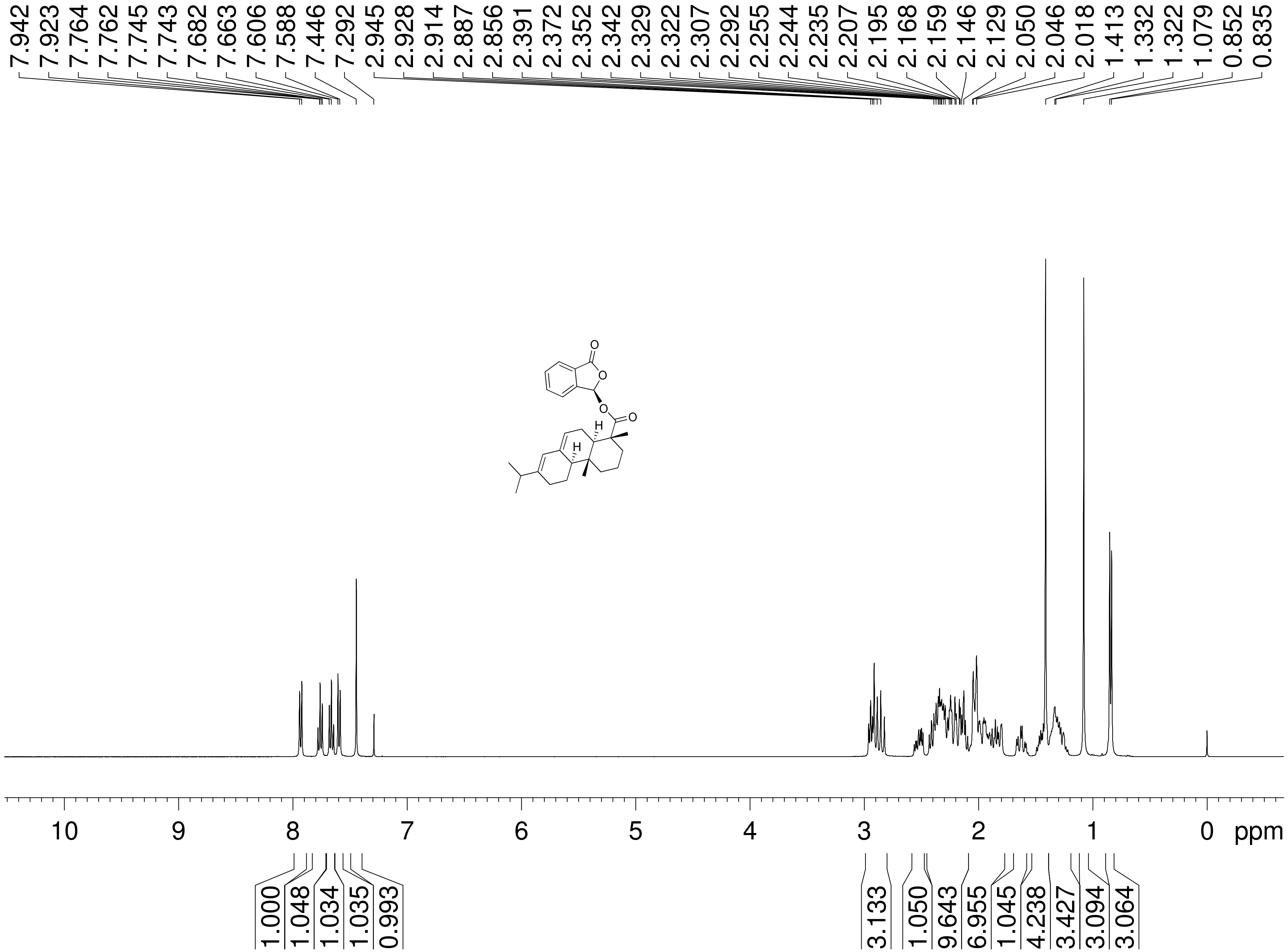

Supplementary Figure 109 ^{1}H NMR spectrum of **55**

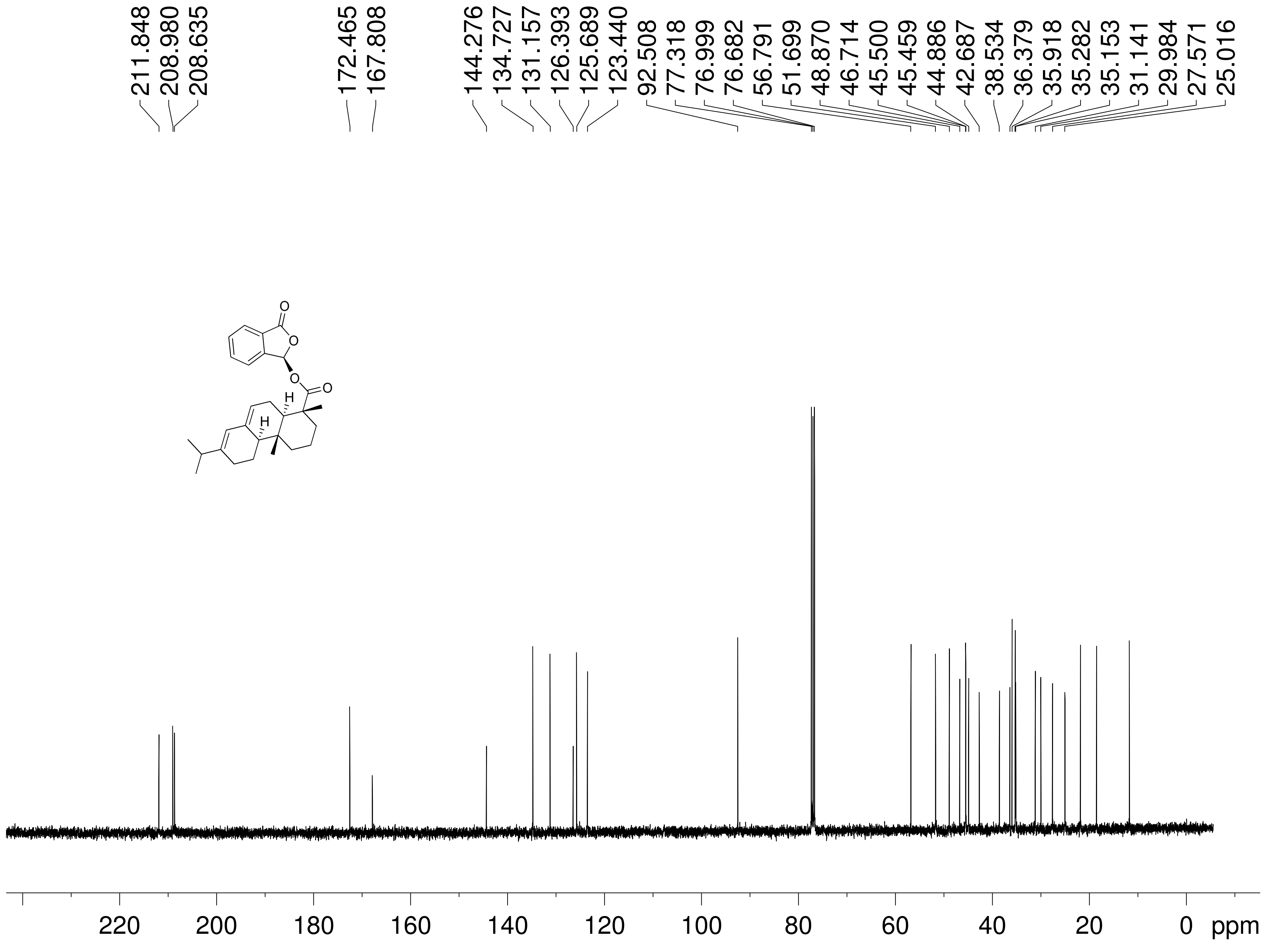

Supplementary Figure 110 ^{13}C NMR spectrum of **55**

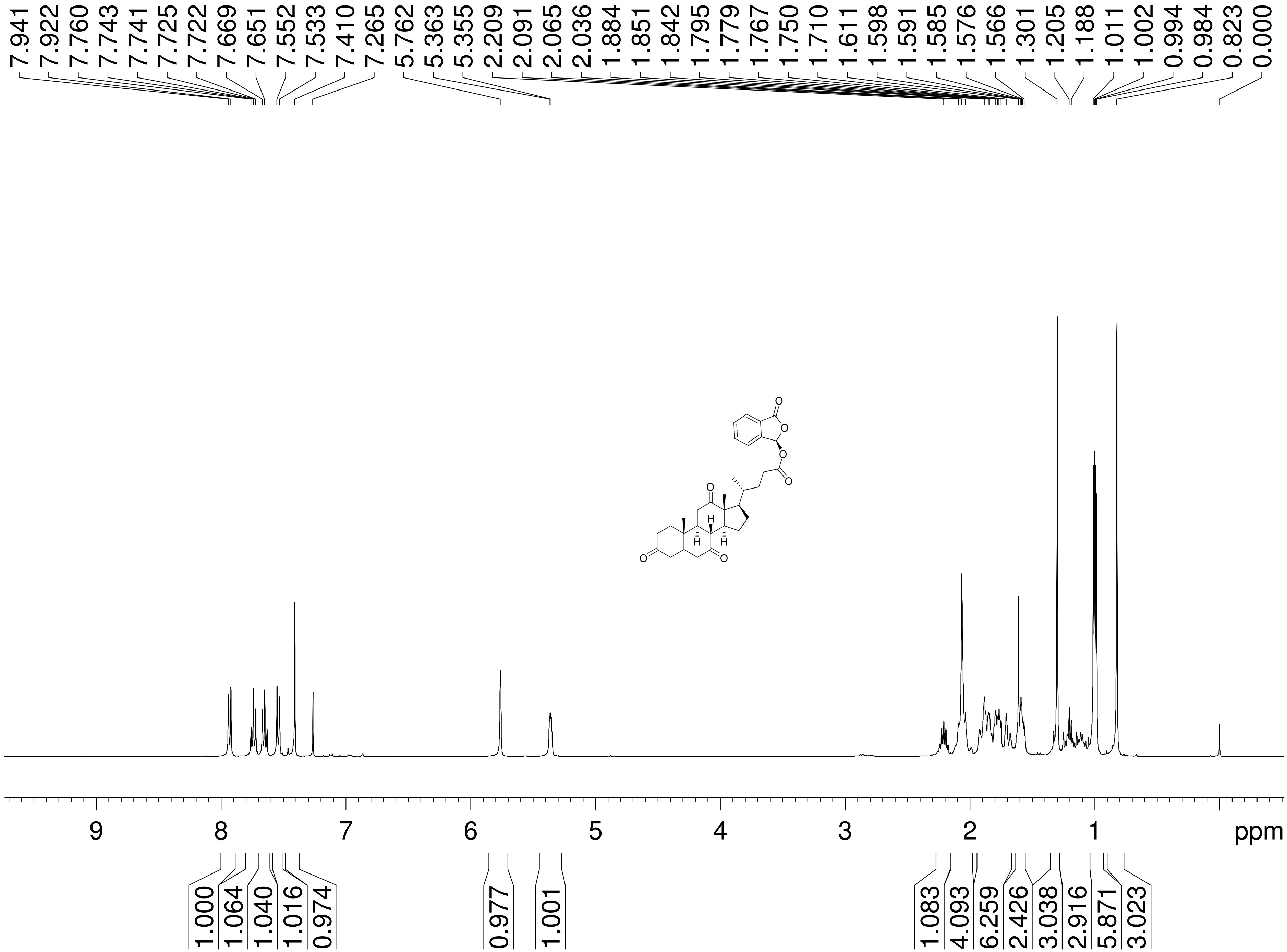

Supplementary Figure 111 ^{1}H NMR spectrum of **56**

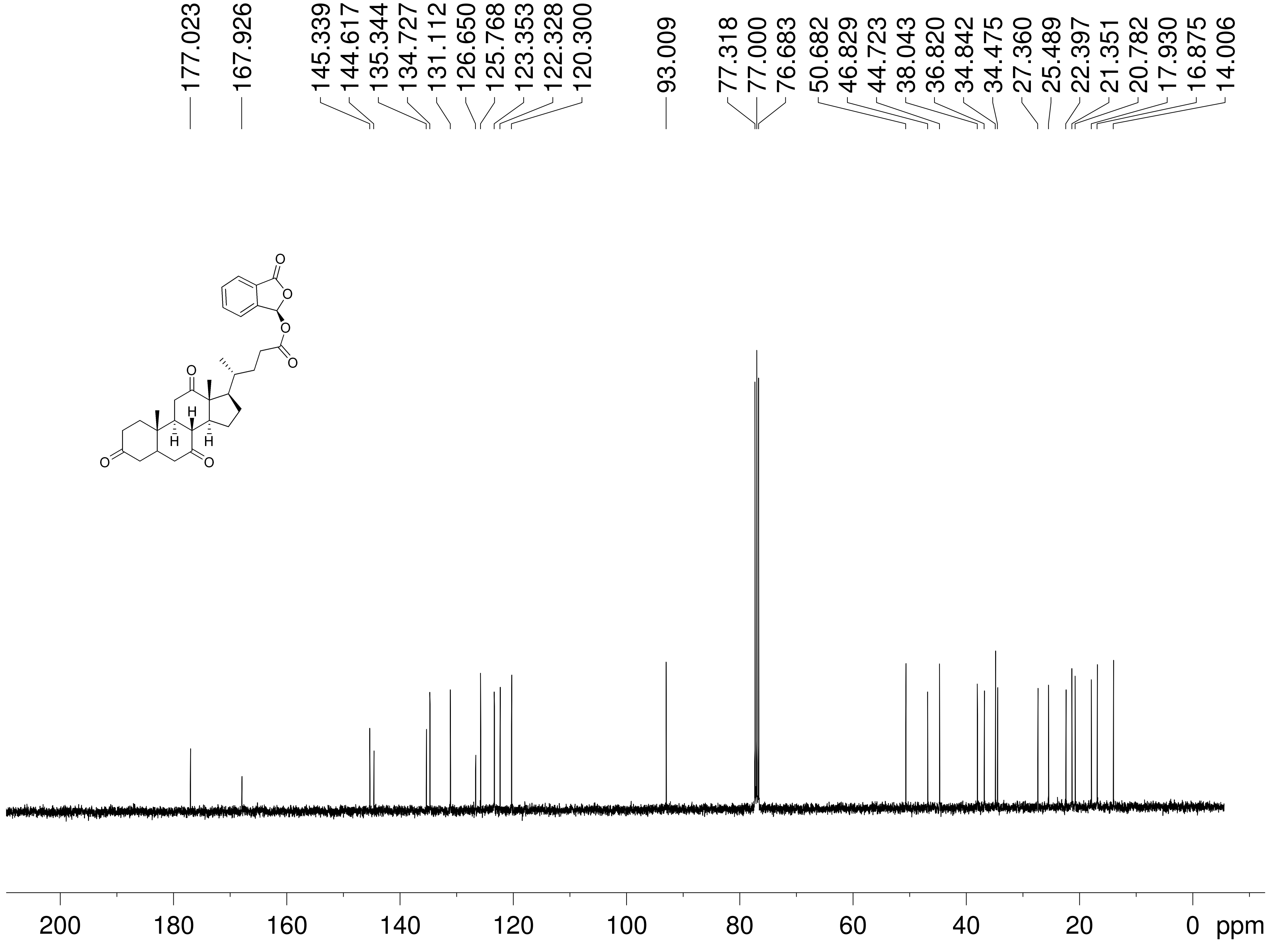

Supplementary Figure 112 ^{13}C NMR spectrum of **56**

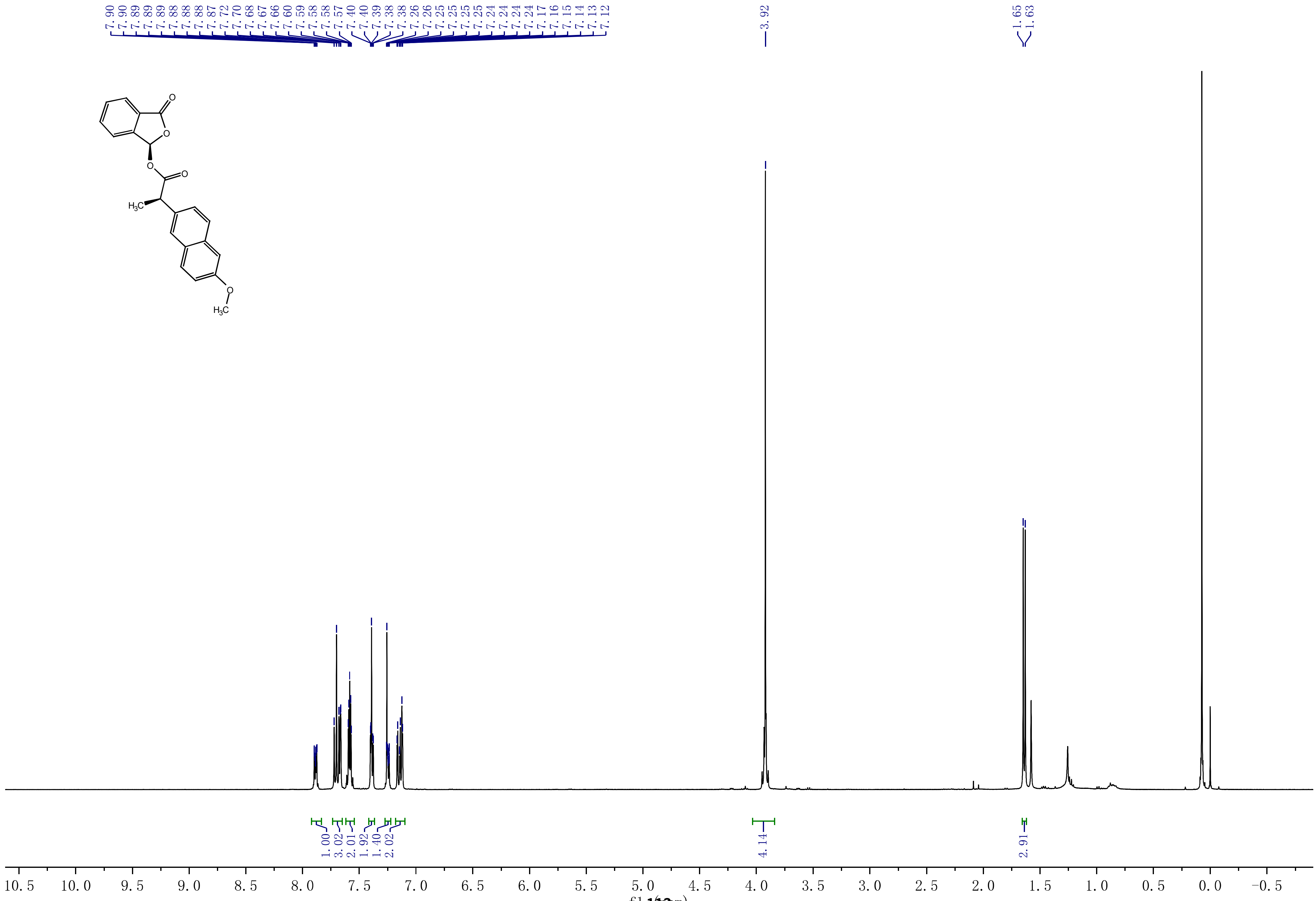

Supplementary Figure 113 ^{1}H NMR spectrum of *R*-**57**

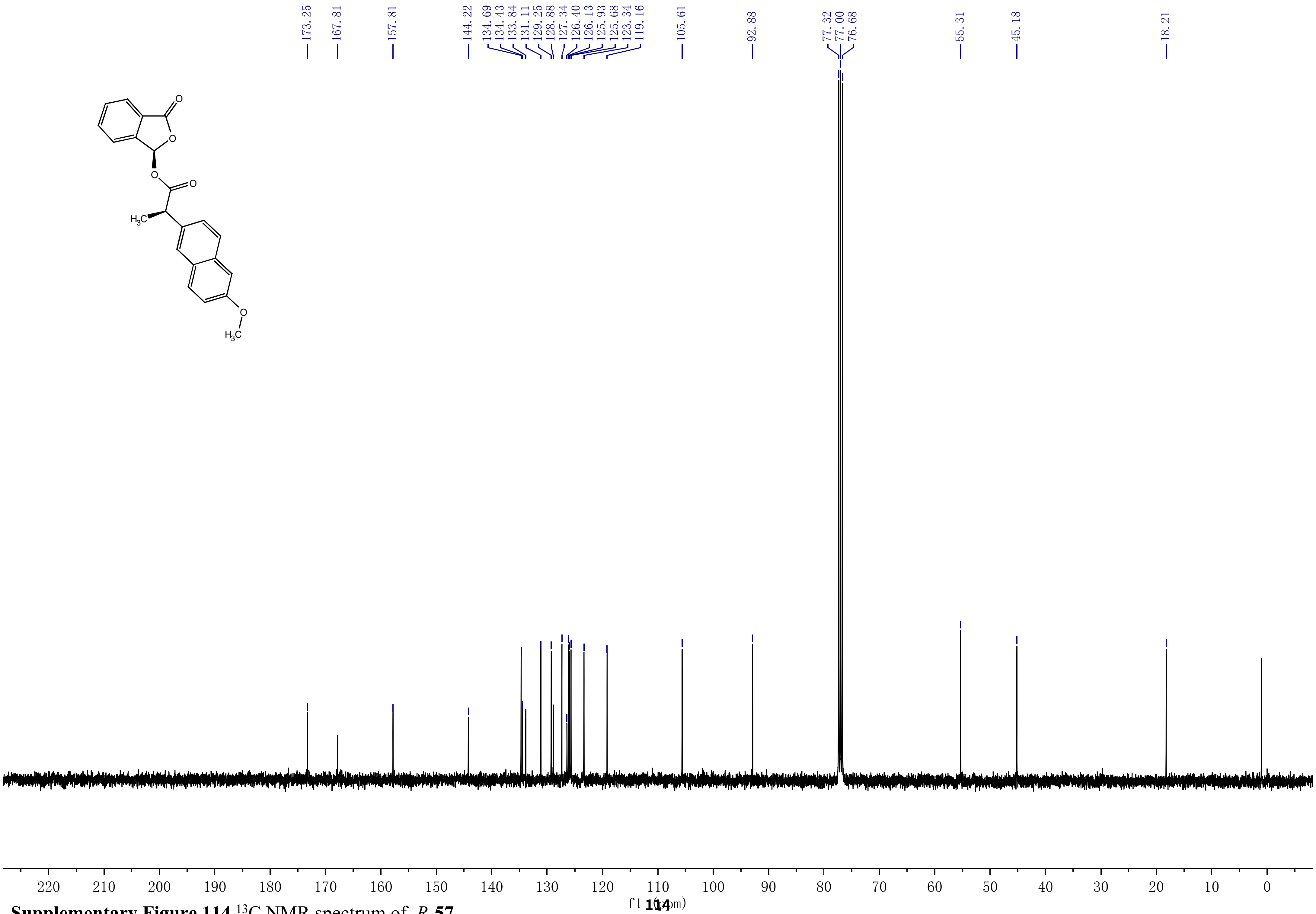

Supplementary Figure 114 ^{13}C NMR spectrum of *R*-**57**

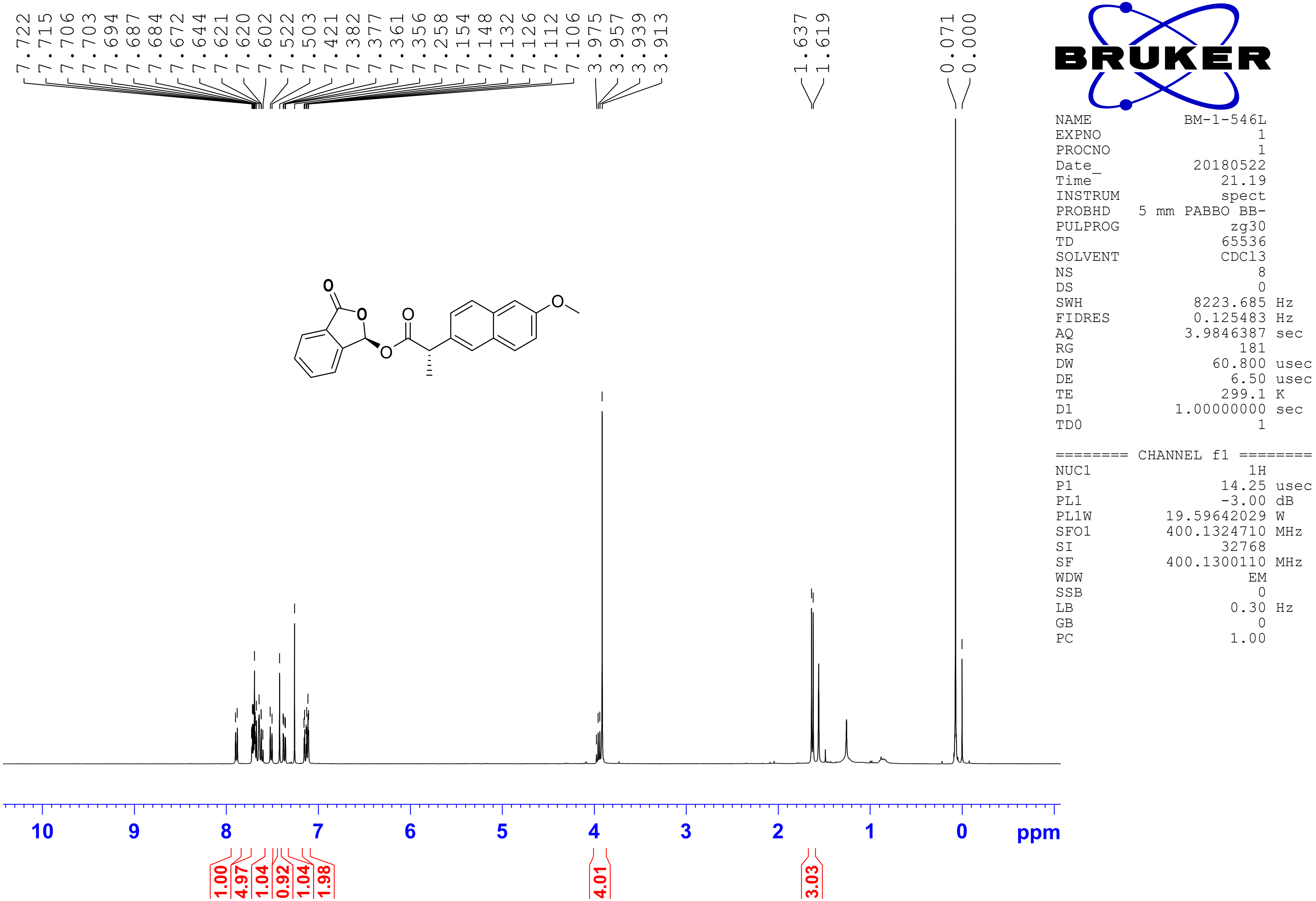

Supplementary Figure 115 ^{1}H NMR spectrum of *S*-**57**

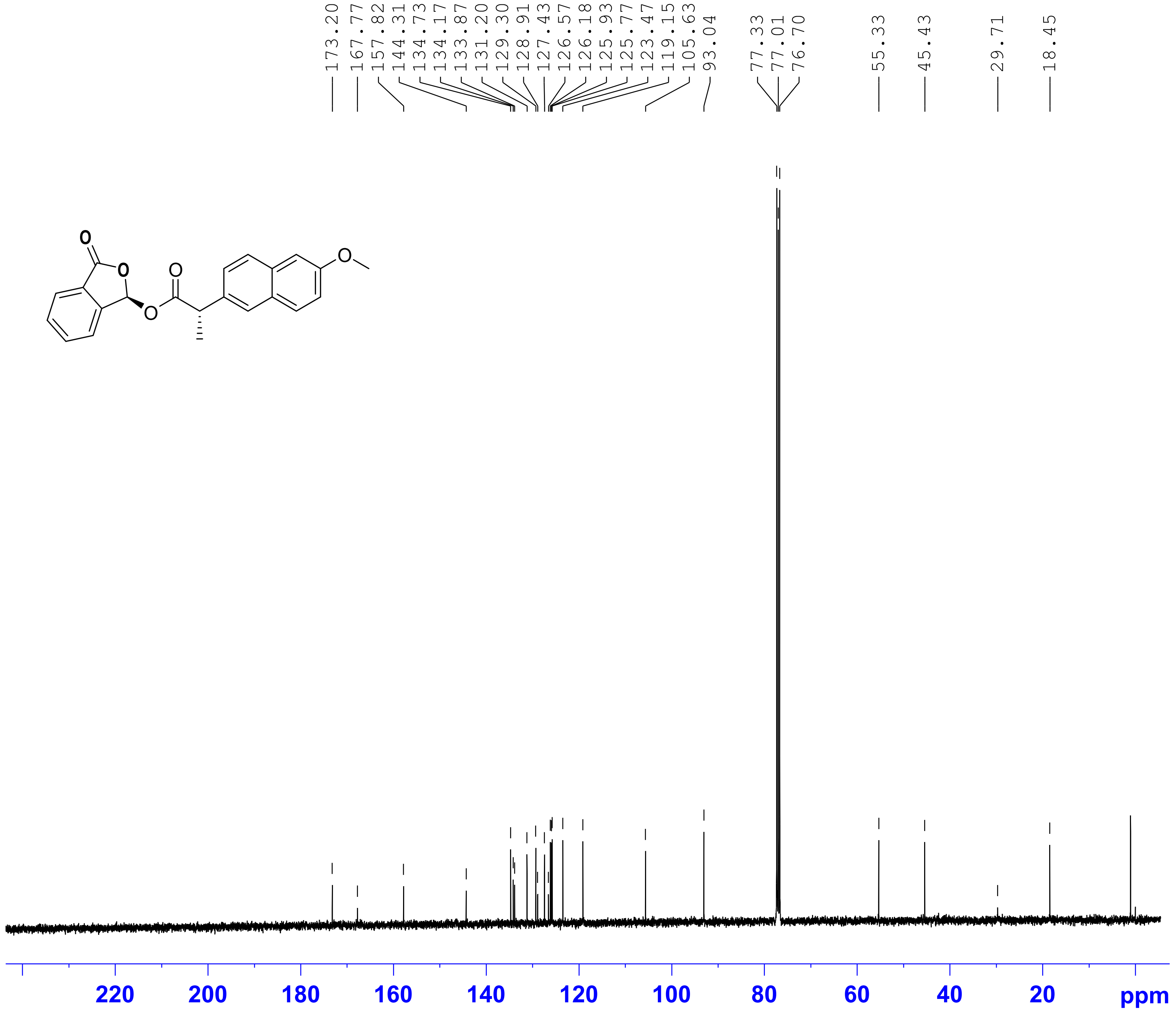

NAME	BM-1-546L
EXPNO	2
PROCNO	1
Date_	20180524
Time	18.16
INSTRUM	spect
PROBHD	5 mm PABBO BB-
PULPROG	zgpg30
TD	65536
SOLVENT	CDCl3
NS	517
DS	0
SWH	25252.525 Hz
FIDRES	0.385323 Hz
AQ	1.2976629 sec
RG	203
DW	19.800 usec
DE	6.50 usec
TE	300.0 K
D1	2.00000000 sec
D11	0.03000000 sec
TD0	100

======== CHANNEL f1 ========

NUC1	13C
P1	11.10 usec
PL1	-2.60 dB
PL1W	65.36360931 W
SFO1	100.6248425 MHz

======== CHANNEL f2 ========

CPDPRG2	waltz16
NUC2	1H
PCPD2	75.00 usec
PL2	-3.00 dB
PL12	11.42 dB
PL13	14.50 dB
PL2W	19.59642029 W
PL12W	0.70823395 W
PL13W	0.34847912 W
SFO2	400.1316005 MHz
SI	32768
SF	100.6127690 MHz
WDW	EM
SSB	0
LB	1.00 Hz
GB	0
PC	1.40

Supplementary Figure 116 ^{13}C NMR spectrum of *S*-**57**

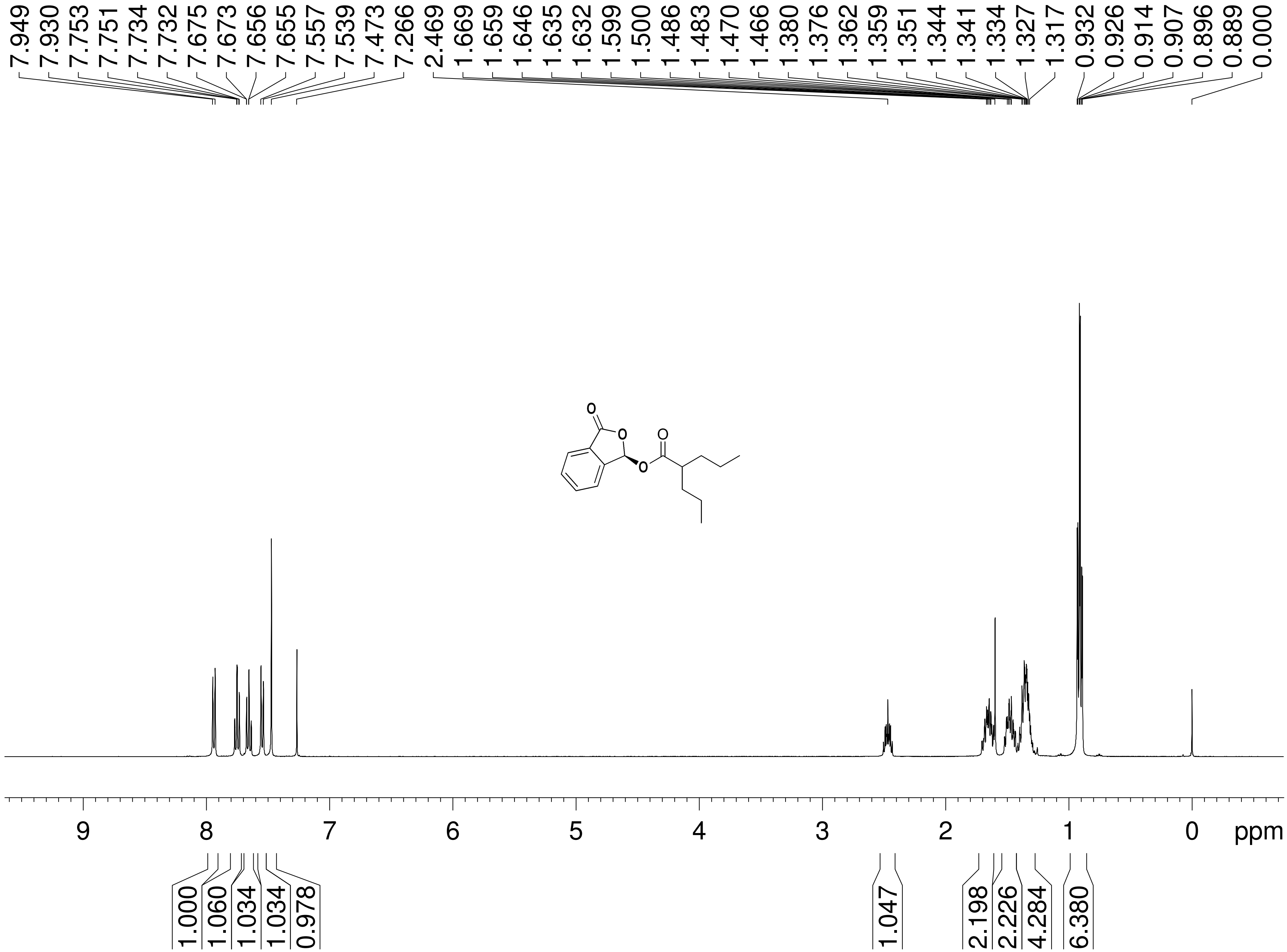

Supplementary Figure 117 ^{1}H NMR spectrum of **58**

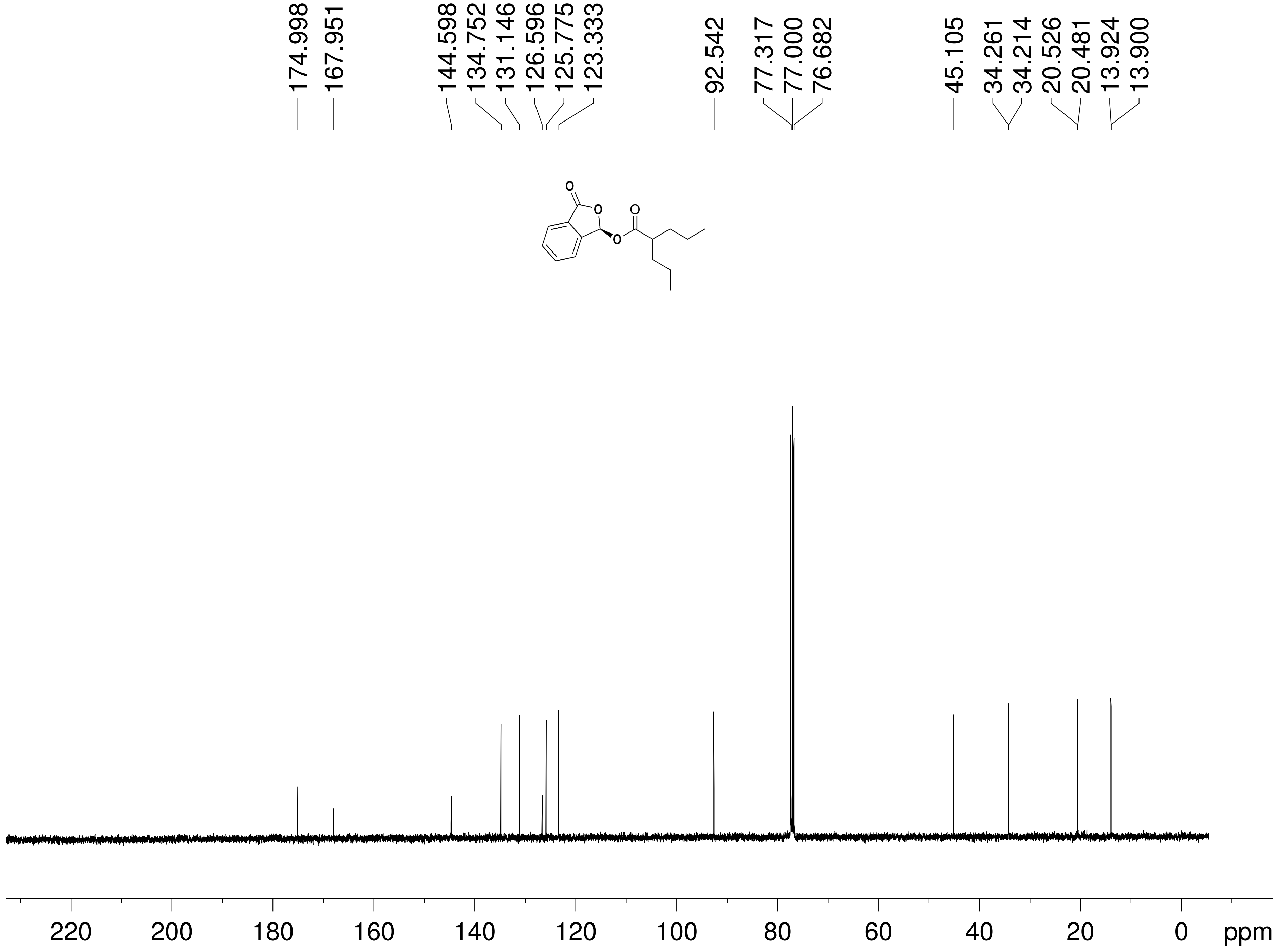

Supplementary Figure 118 ^{13}C NMR spectrum of **58**

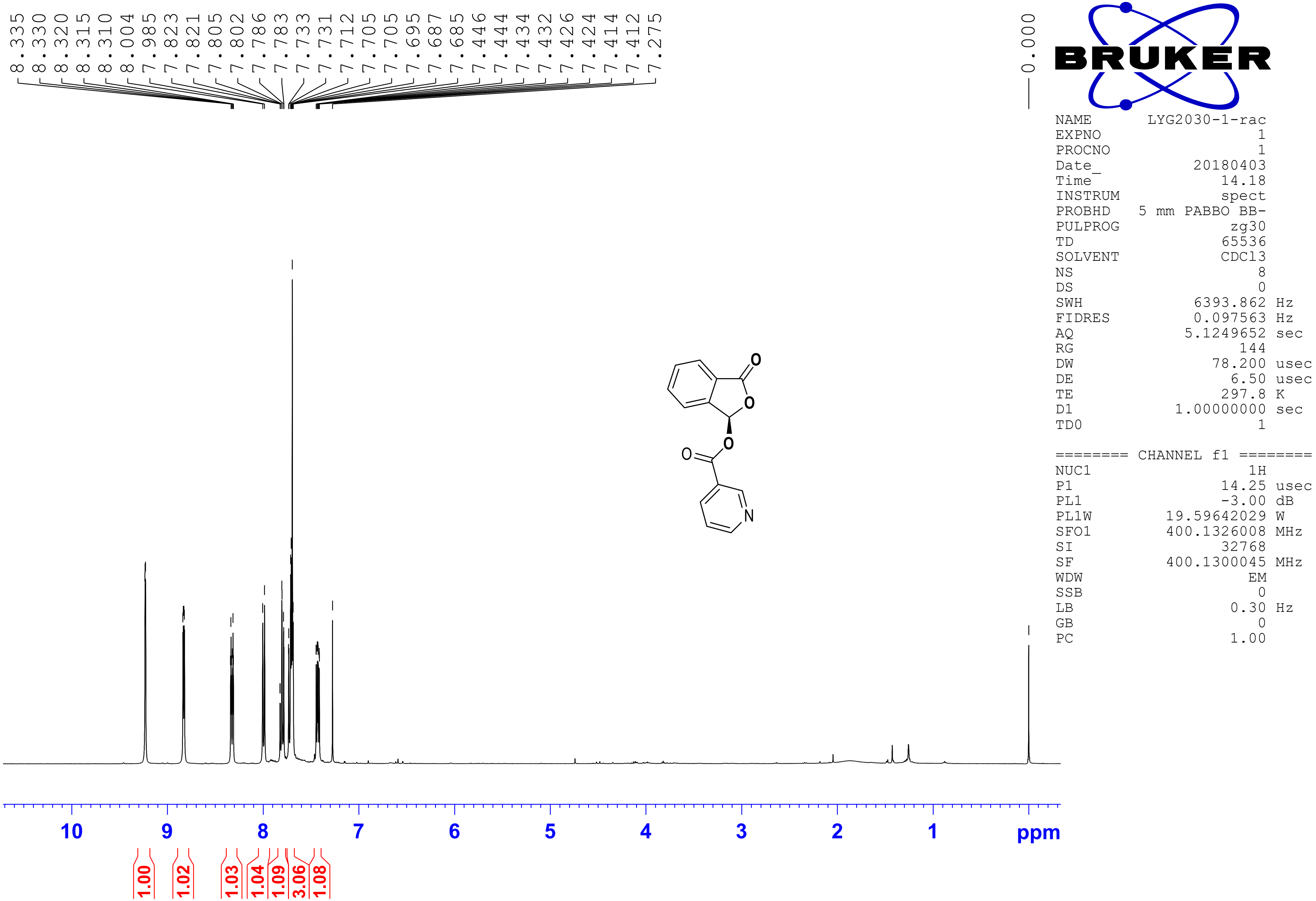

Supplementary Figure 119 ^{1}H NMR spectrum of **59**

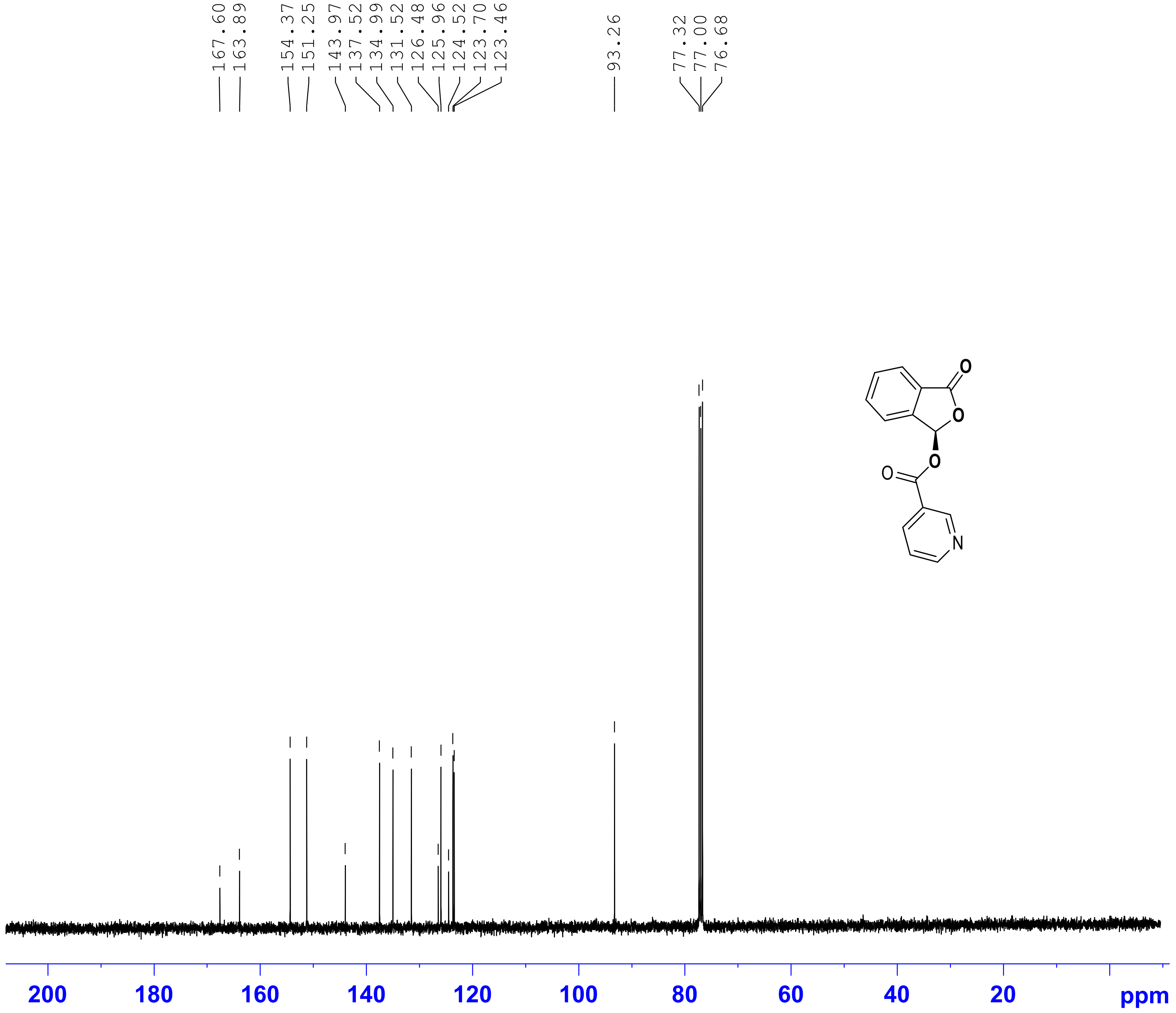

BRUKER

```
NAME          LYG2030-1-rac
EXPNO                     2
PROCNO                    1
Date_              20180403
Time                  14.23
INSTRUM               spect
PROBHD      5 mm PABBO BB-
PULPROG             zgpg30
TD                    65536
SOLVENT               CDCl3
NS                      144
DS                        0
SWH               22058.824 Hz
FIDRES             0.336591 Hz
AQ                1.4855326 sec
RG                      203
DW                   22.667 usec
DE                     6.50 usec
TE                    298.3 K
D1               2.00000000 sec
D11              0.03000000 sec
TD0                     100

======== CHANNEL f1 ========
NUC1                    13C
P1                    11.10 usec
PL1                   -2.60 dB
PL1W            65.36360931 W
SFO1            100.6228303 MHz

======== CHANNEL f2 ========
CPDPRG2             waltz16
NUC2                     1H
PCPD2                 75.00 usec
PL2                   -3.00 dB
PL12                  11.42 dB
PL13                  14.50 dB
PL2W            19.59642029 W
PL12W            0.70823395 W
PL13W            0.34847912 W
SFO2            400.1316005 MHz
SI                    32768
SF              100.6127734 MHz
WDW                      EM
SSB                       0
LB                     1.00 Hz
GB                        0
PC                     1.40
```

Supplementary Figure 120 ^{13}C NMR spectrum of **59**

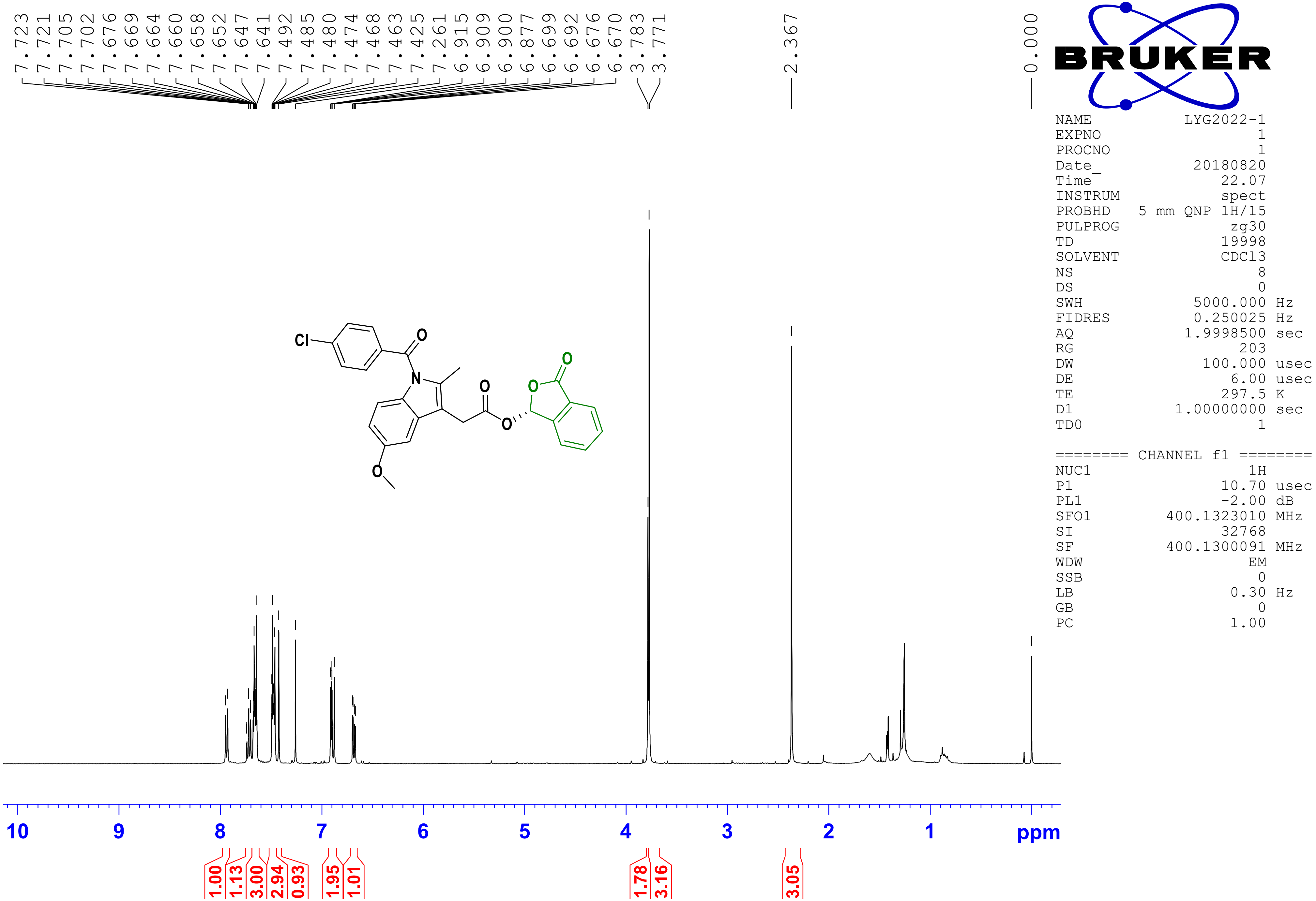

Supplementary Figure 121 ^{1}H NMR spectrum of **60**

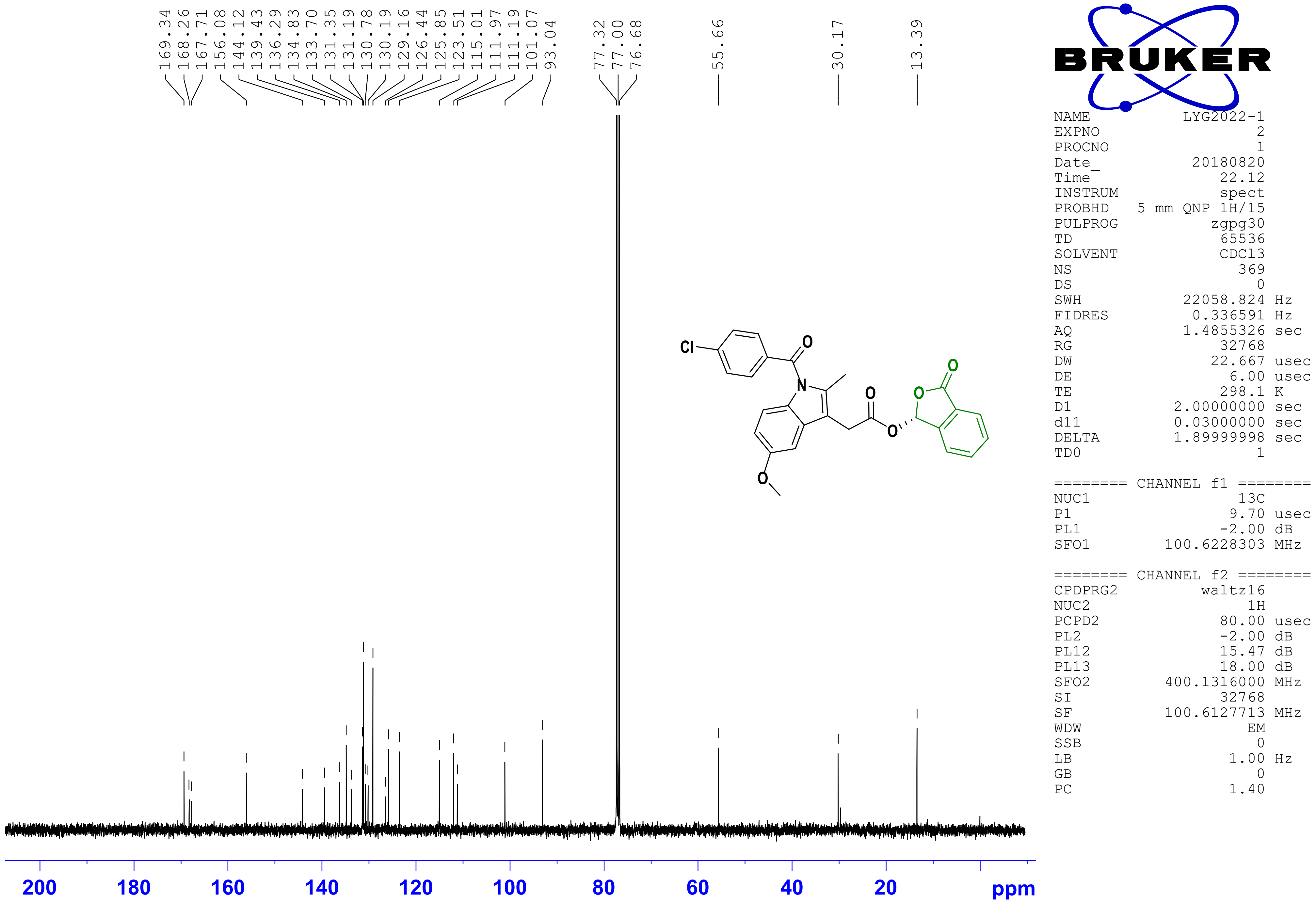

Supplementary Figure 122 ^{13}C NMR spectrum of **60**

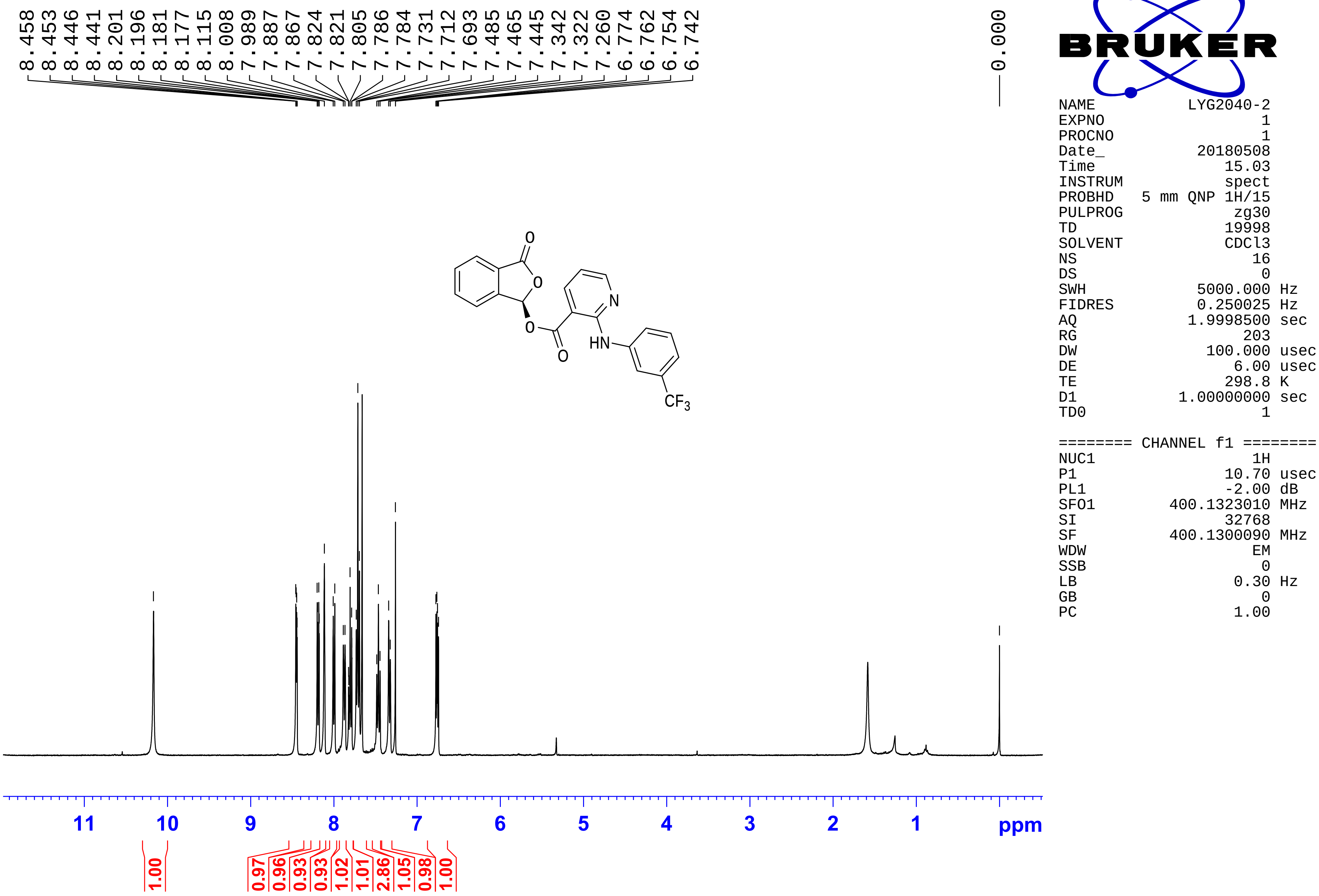

Supplementary Figure 123 ^{1}H NMR spectrum of **61**

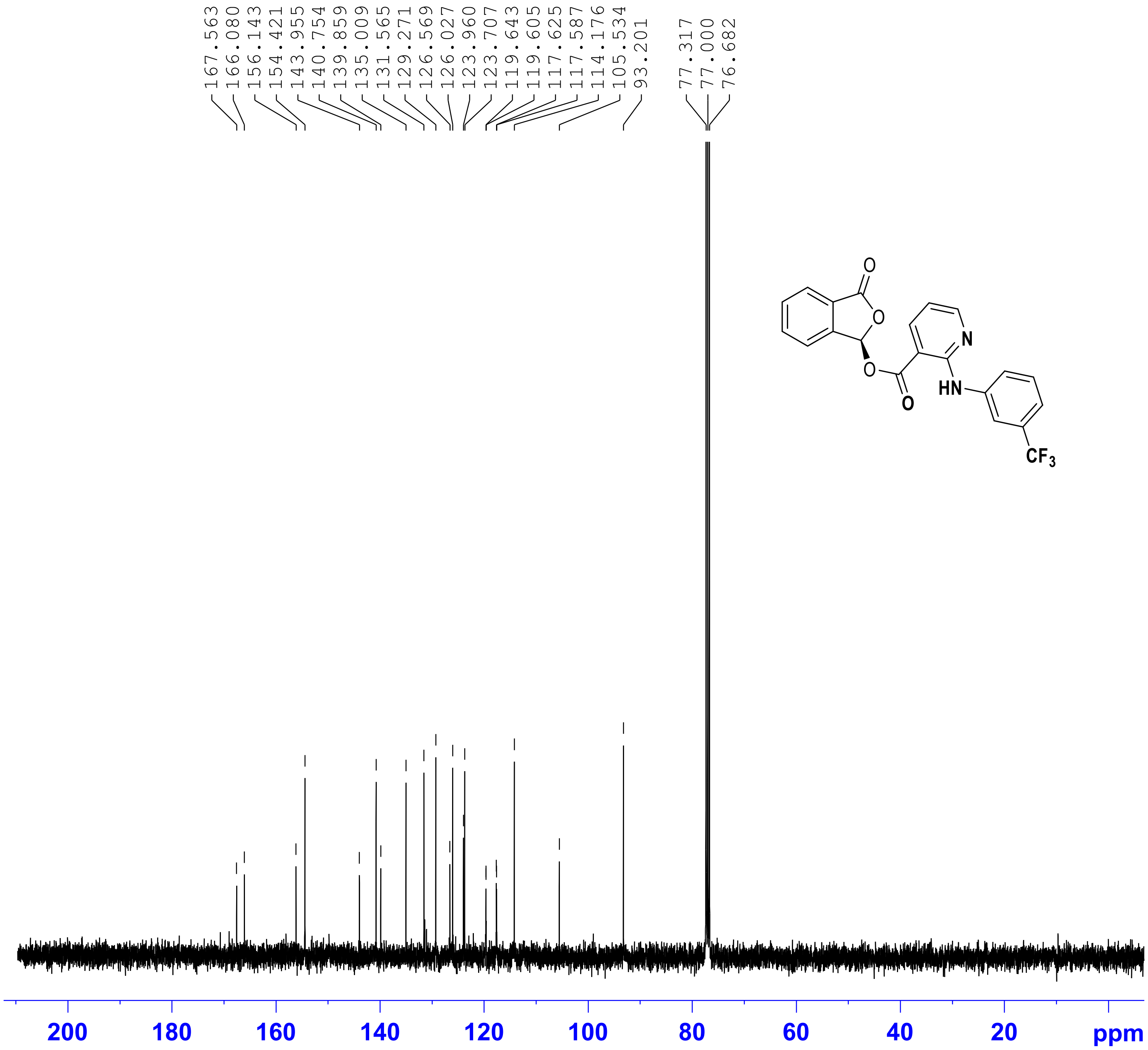

NAME	LYG2040-2
EXPNO	2
PROCNO	1
Date_	20180508
Time	15.05
INSTRUM	spect
PROBHD	5 mm QNP 1H/15
PULPROG	zgpg30
TD	65536
SOLVENT	CDCl3
NS	340
DS	0
SWH	22058.824 Hz
FIDRES	0.336591 Hz
AQ	1.4855326 sec
RG	32768
DW	22.667 usec
DE	6.00 usec
TE	299.1 K
D1	2.00000000 sec
d11	0.03000000 sec
DELTA	1.89999998 sec
TD0	1

======== CHANNEL f1 ========

NUC1	13C
P1	9.70 usec
PL1	-2.00 dB
SFO1	100.6228303 MHz

======== CHANNEL f2 ========

CPDPRG2	waltz16
NUC2	1H
PCPD2	80.00 usec
PL2	-2.00 dB
PL12	15.47 dB
PL13	18.00 dB
SFO2	400.1316000 MHz
SI	32768
SF	100.6127708 MHz
WDW	EM
SSB	0
LB	1.00 Hz
GB	0
PC	1.40

Supplementary Figure 124 ^{13}C NMR spectrum of **61**

62

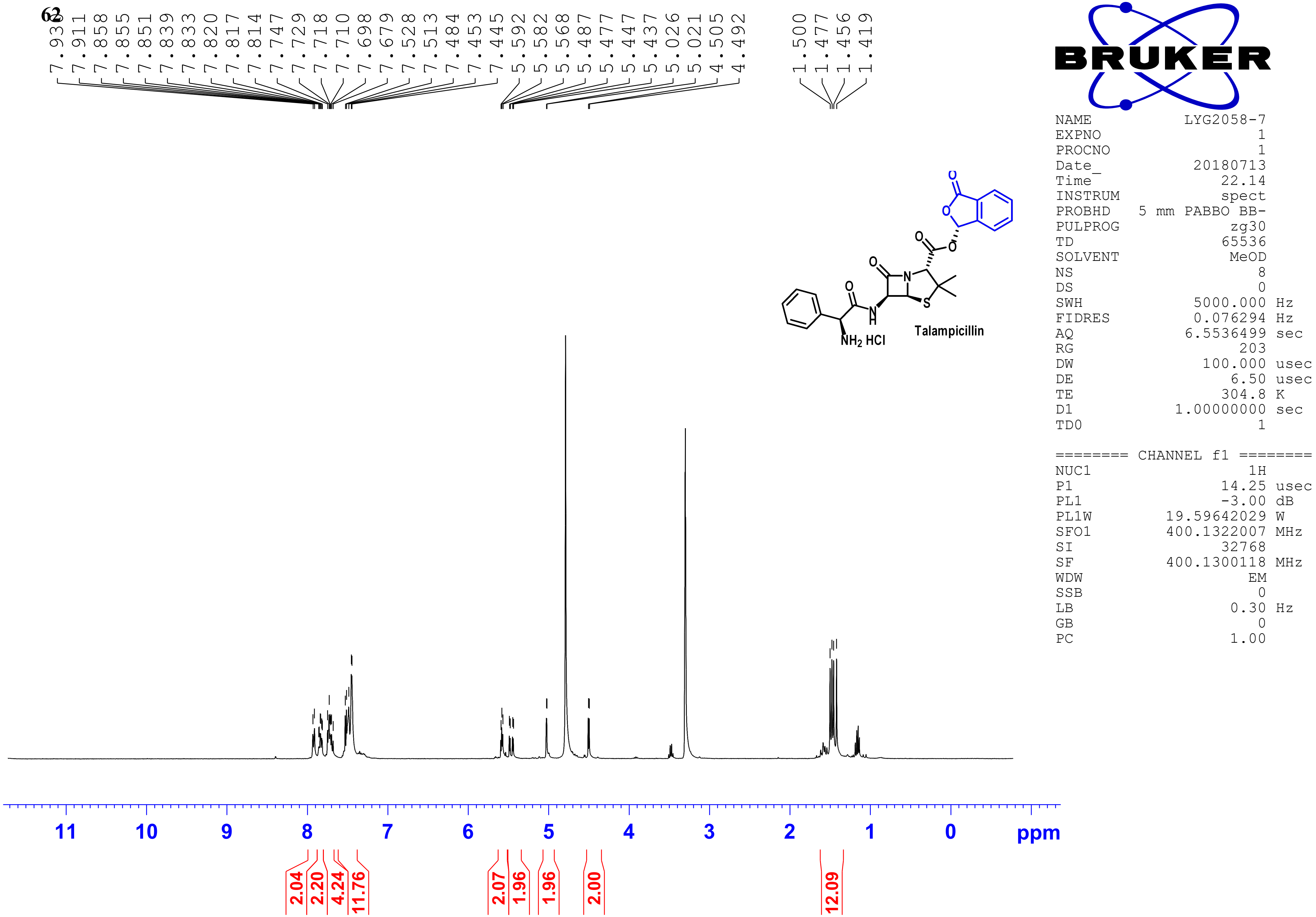

Supplementary Figure 125 ^{1}H NMR spectrum of

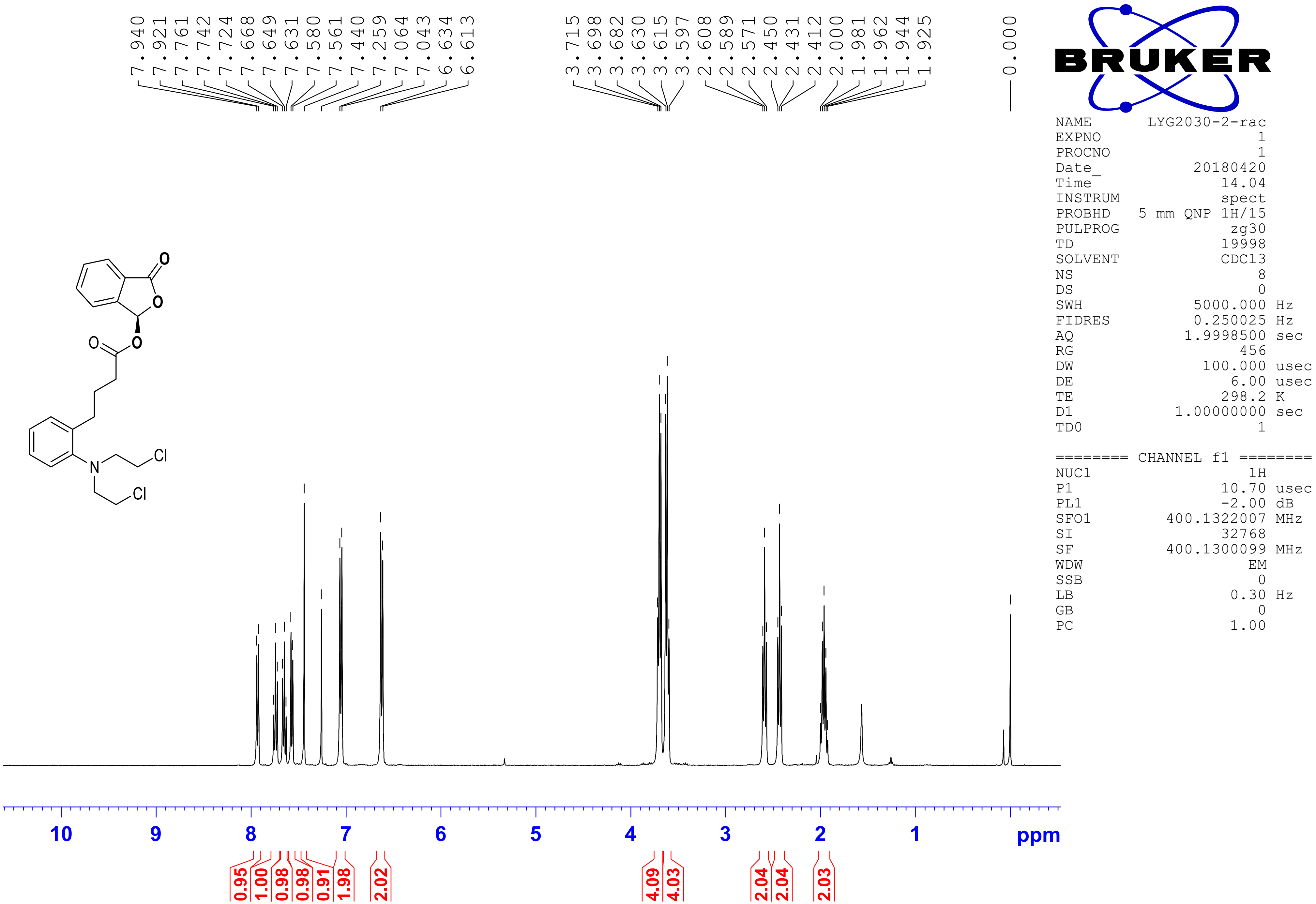

Supplementary Figure 126 ^{1}H NMR spectrum of **63**

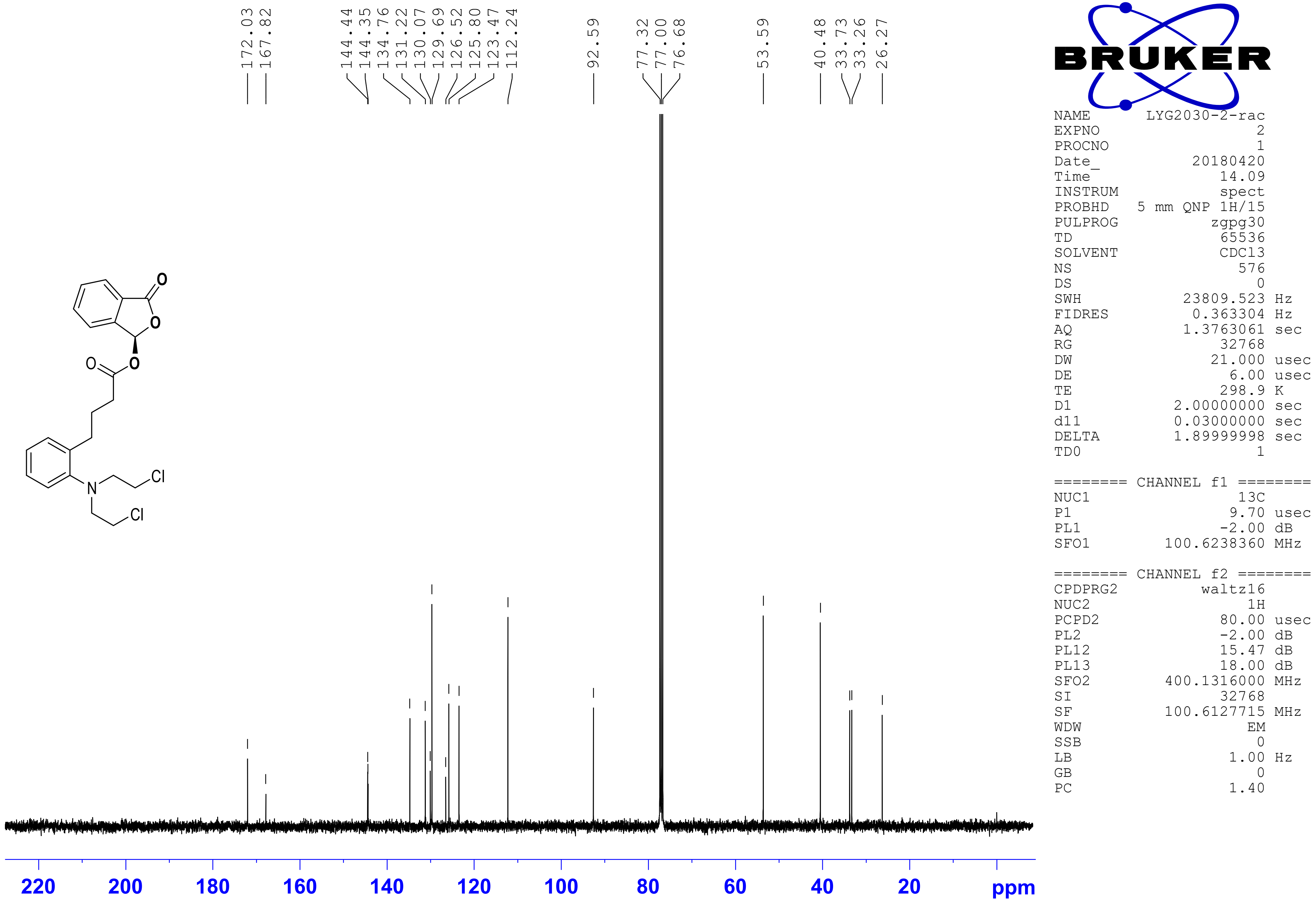

Supplementary Figure 127 ^{13}C NMR spectrum of **63**

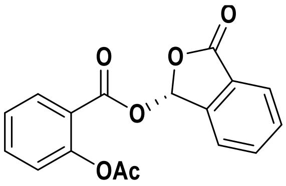

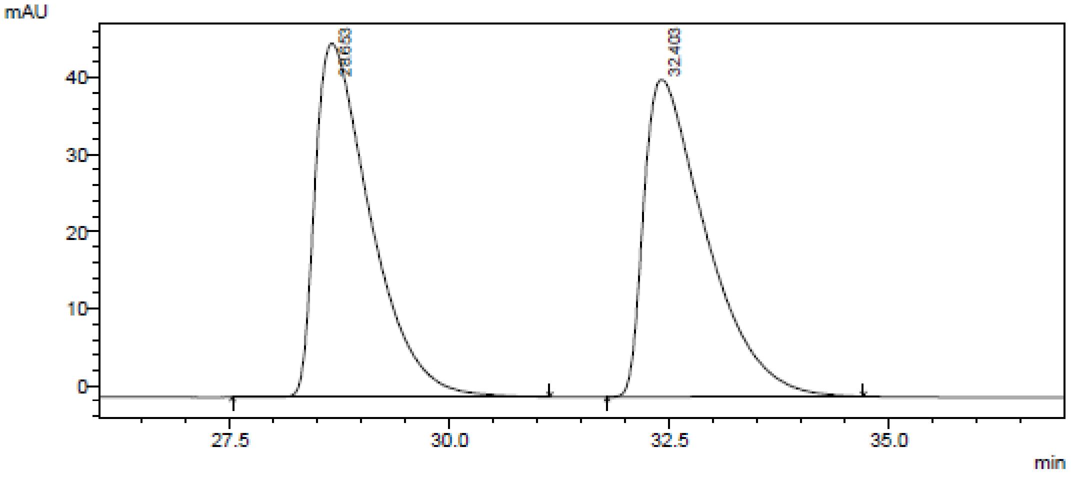

Peak#	Ret. Time	Area	Height	Area %	Height %
1	28.653	2012300	45881	49.676	52.719
2	32.403	2038567	41149	50.324	47.281
Total		4050868	87030	100.000	100.000

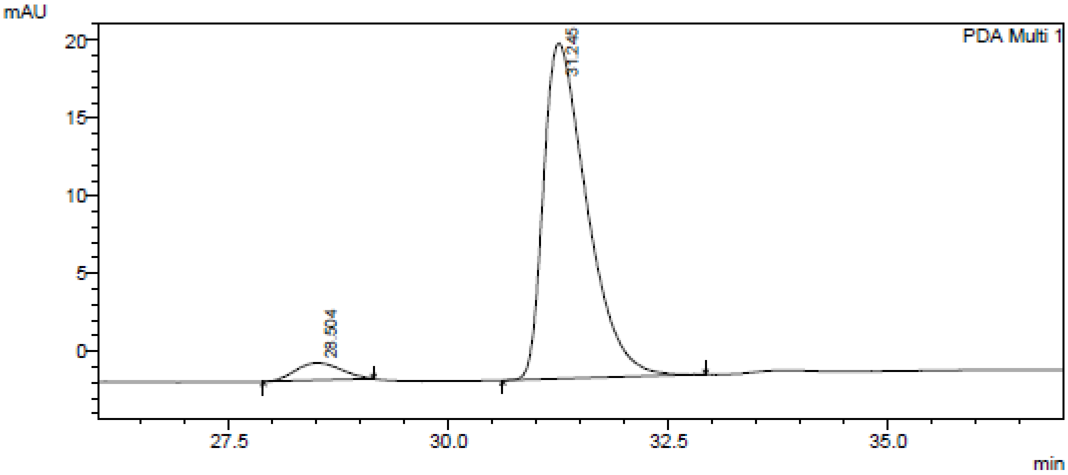

Peak#	Ret. Time	Area	Height	Area %	Height %
1	28.504	40298	1120	5.164	4.943
2	31.245	740065	21535	94.836	95.057
Total		780363	22654	100.000	100.000

Supplementary Figure 128 HPLC Spectra of **3**

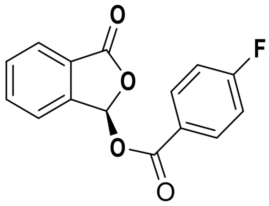

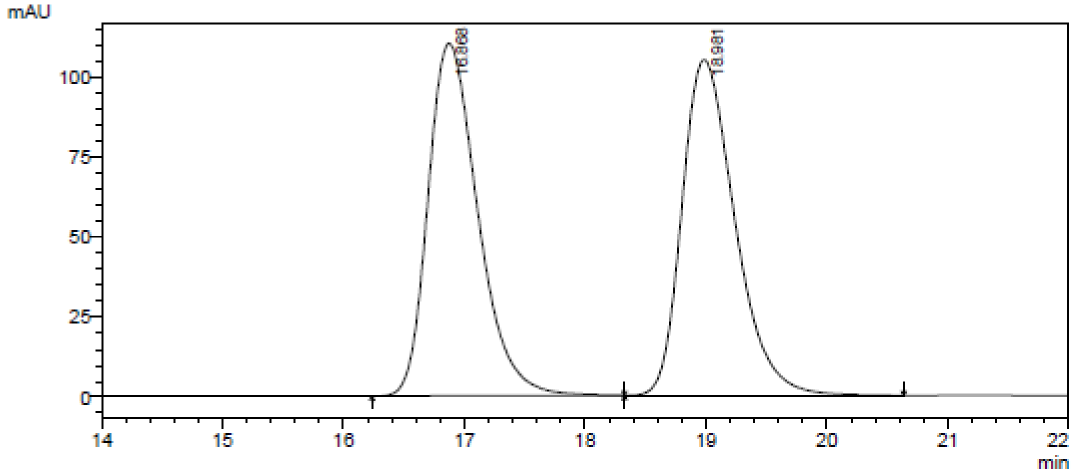

Peak#	Ret. Time	Area	Height	Area %	Height %
1	16.868	3172840	110286	49.726	51.204
2	18.981	3207853	105099	50.274	48.796
Total		6380693	215386	100.000	100.000

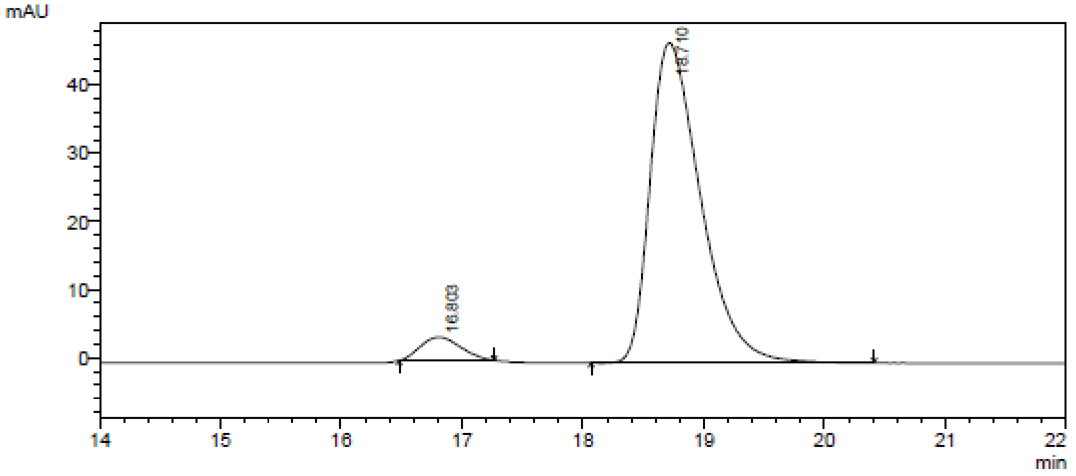

Peak#	Ret. Time	Area	Height	Area %	Height %
1	16.804	616538	26520	5.504	6.781
2	18.710	10585162	364588	94.496	93.219
Total		11201701	391109	100.000	100.000

Supplementary Figure 129 HPLC Spectra of **4**

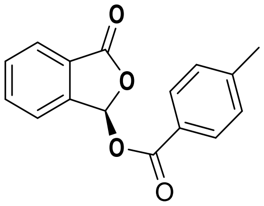

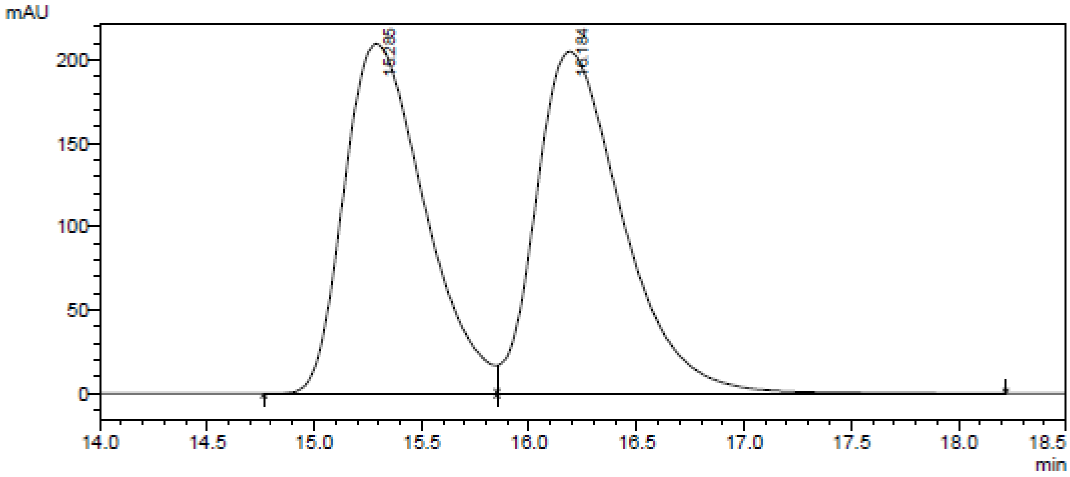

Peak#	Ret. Time	Area	Height	Area %	Height %
1	15.285	5466427	209758	48.820	50.551
2	16.184	5730602	205182	51.180	49.449
Total		11197029	414940	100.000	100.000

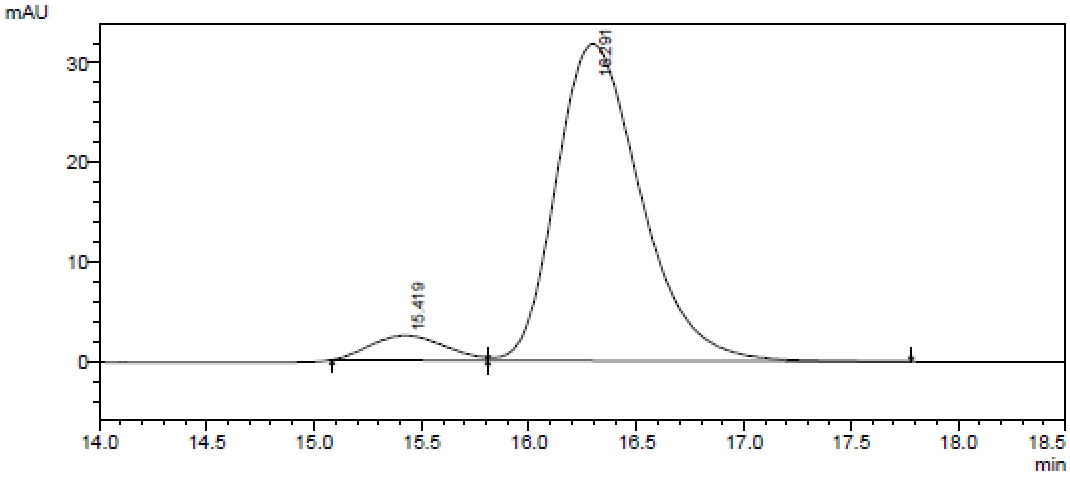

Peak#	Ret. Time	Area	Height	Area %	Height %
1	15.419	58467	2434	6.310	7.124
2	16.291	868056	31738	93.690	92.876
Total		926524	34172	100.000	100.000

Supplementary Figure 130 HPLC Spectra of **5**

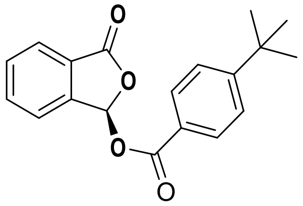

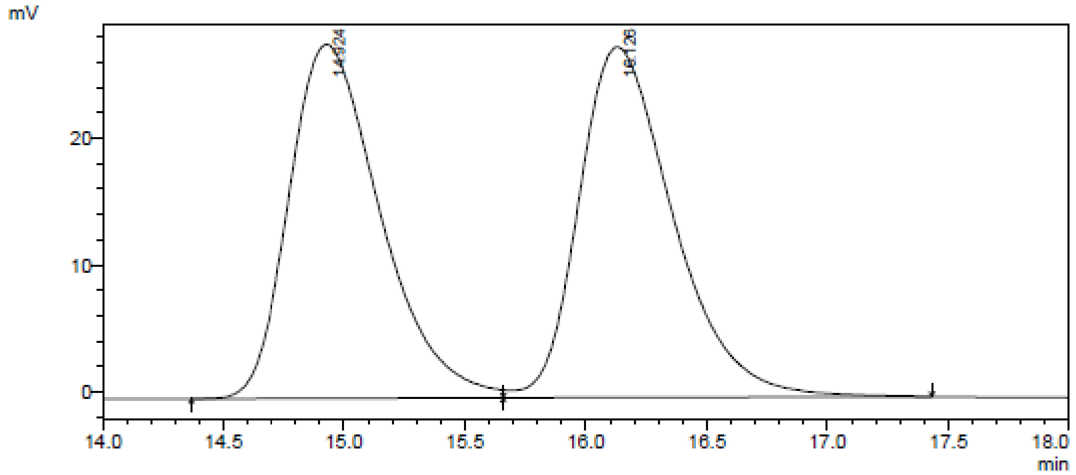

Peak#	Ret. Time	Area	Height	Area %	Height %
1	14.924	734073	27915	49.476	50.250
2	16.126	749632	27637	50.524	49.750
Total		1483705	55552	100.000	100.000

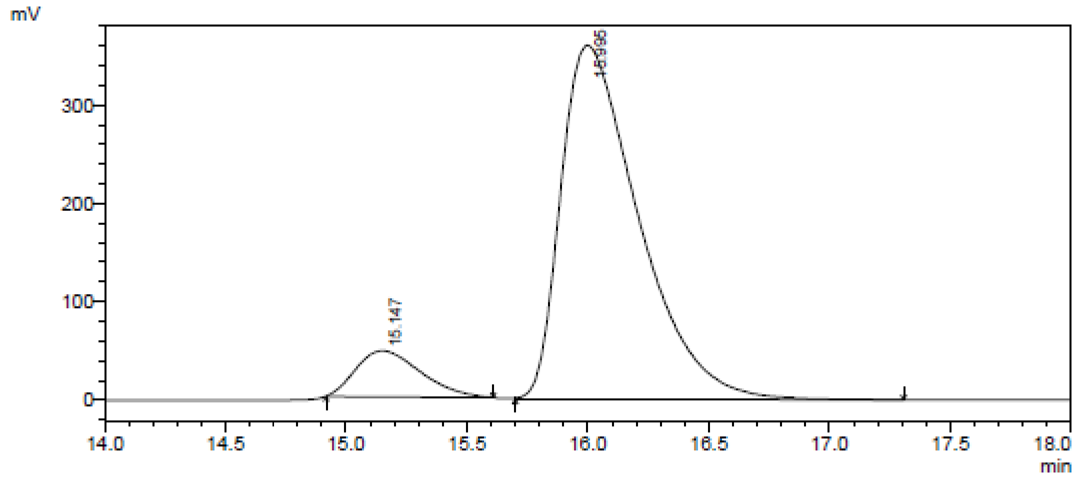

Peak#	Ret. Time	Area	Height	Area %	Height %
1	15.147	867798	46672	9.685	11.471
2	15.995	8092215	360190	90.315	88.529
Total		8960013	406862	100.000	100.000

Supplementary Figure 131 HPLC Spectra of **6**

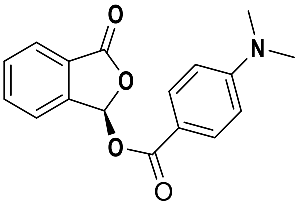

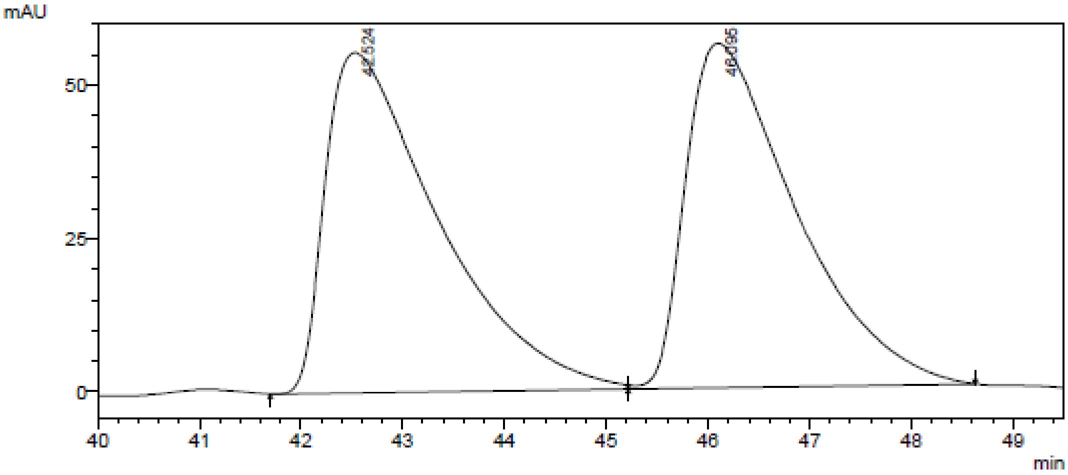

Peak#	Ret. Time	Area	Height	Area %	Height %
1	42.524	4291717	55588	50.374	49.717
2	46.095	4227943	56222	49.626	50.283
Total		8519660	111810	100.000	100.000

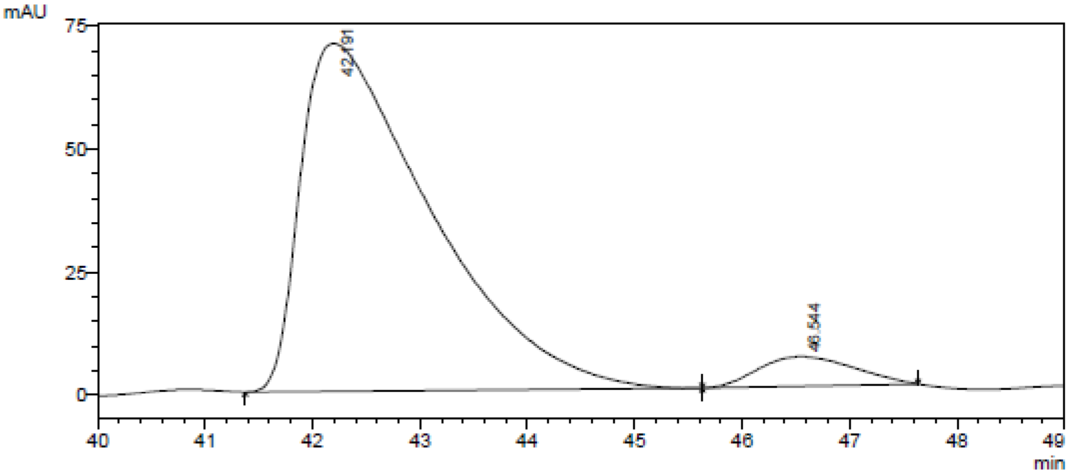

Peak#	Ret. Time	Area	Height	Area %	Height %
1	42.191	5961209	70736	94.136	92.185
2	46.544	371368	5997	5.864	7.815
Total		6332577	76733	100.000	100.000

Supplementary Figure 132 HPLC Spectra of **7**

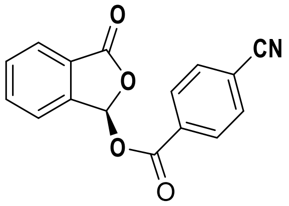

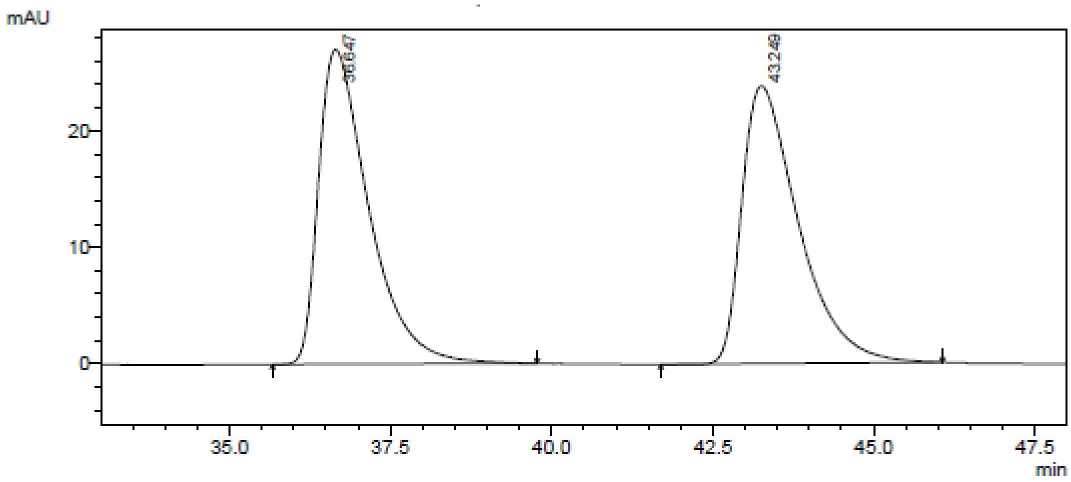

Peak#	Ret. Time	Area	Height	Area %	Height %
1	36.647	1465459	27018	50.045	53.098
2	43.249	1462822	23865	49.955	46.902
Total		2928282	50883	100.000	100.000

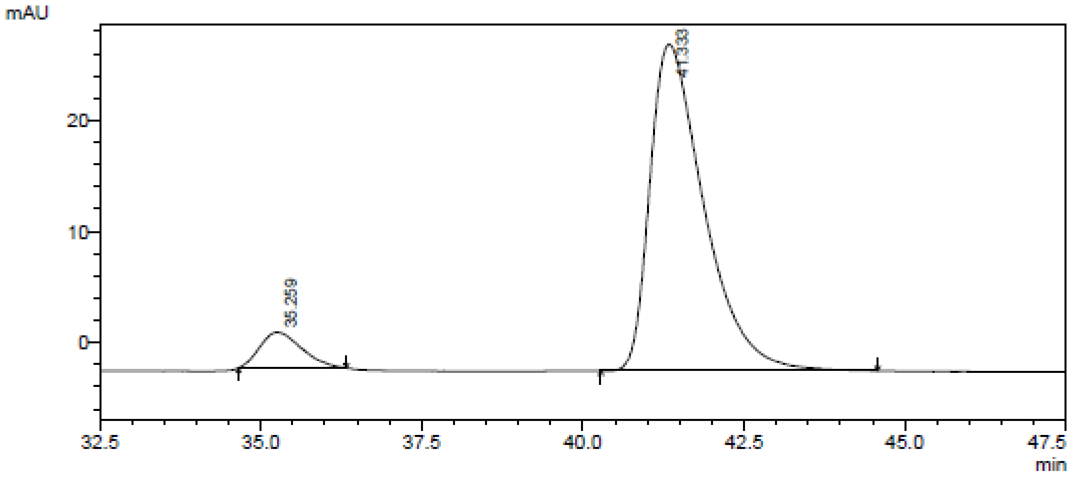

Peak#	Ret. Time	Area	Height	Area %	Height %
1	35.259	149443	3240	7.993	9.934
2	41.333	1720290	29375	92.007	90.066
Total		1869733	32615	100.000	100.000

Supplementary Figure 133 HPLC Spectra of **8**

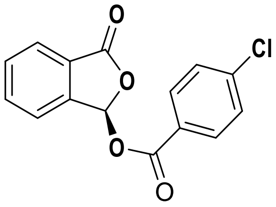

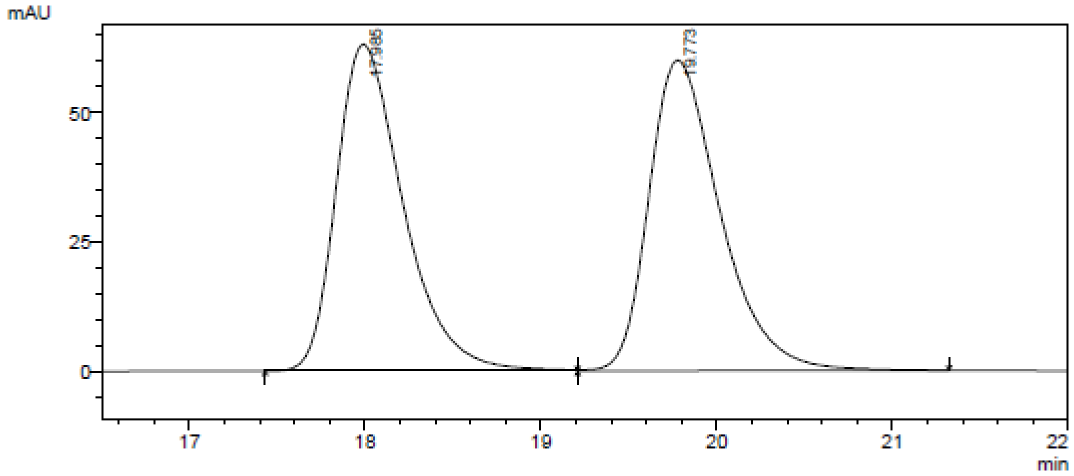

Peak#	Ret. Time	Area	Height	Area %	Height %
1	17.985	1672017	63086	49.735	51.265
2	19.773	1689855	59972	50.265	48.735
Total		3361873	123058	100.000	100.000

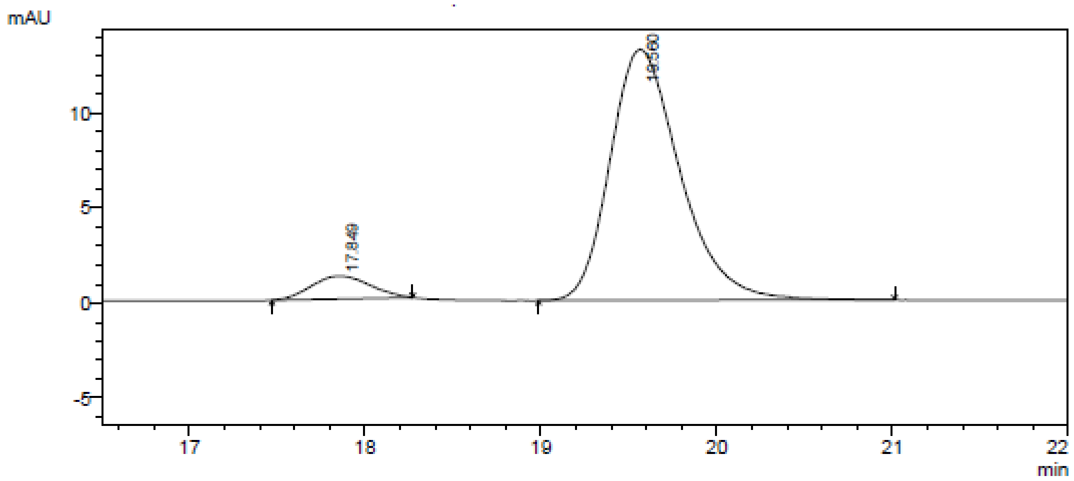

Peak#	Ret. Time	Area	Height	Area %	Height %
1	17.849	27755	1207	7.061	8.380
2	19.560	365334	13196	92.939	91.620
Total		393089	14402	100.000	100.000

Supplementary Figure 134 HPLC Spectra of **9**

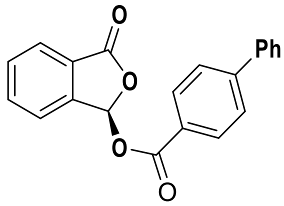

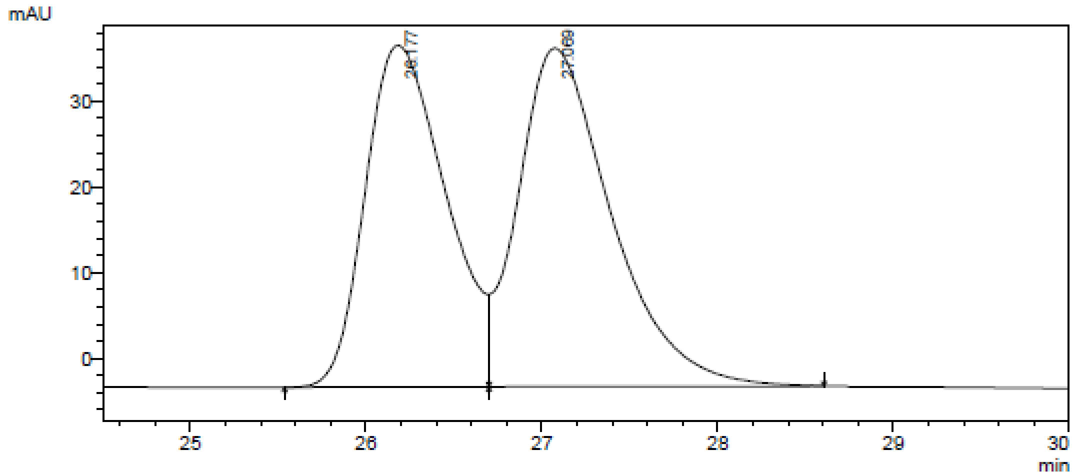

Peak#	Ret. Time	Area	Height	Area %	Height %
1	26.177	1273031	39816	46.681	50.226
2	27.069	1454050	39457	53.319	49.774
Total		2727081	79273	100.000	100.000

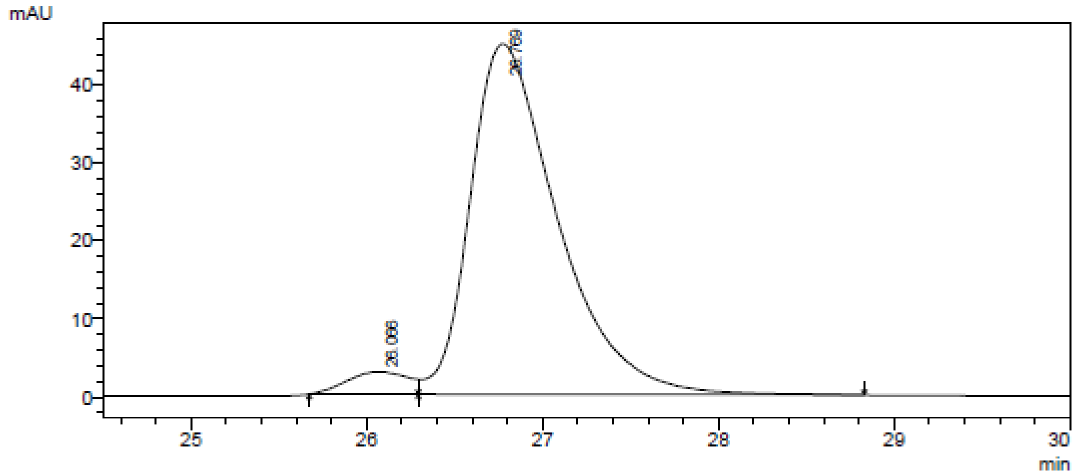

Peak#	Ret. Time	Area	Height	Area %	Height %
1	26.066	68957	2845	4.250	5.952
2	26.769	1553368	44947	95.750	94.048
Total		1622325	47791	100.000	100.000

Supplementary Figure 135 HPLC Spectra of **10**

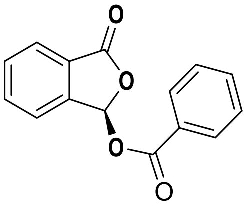

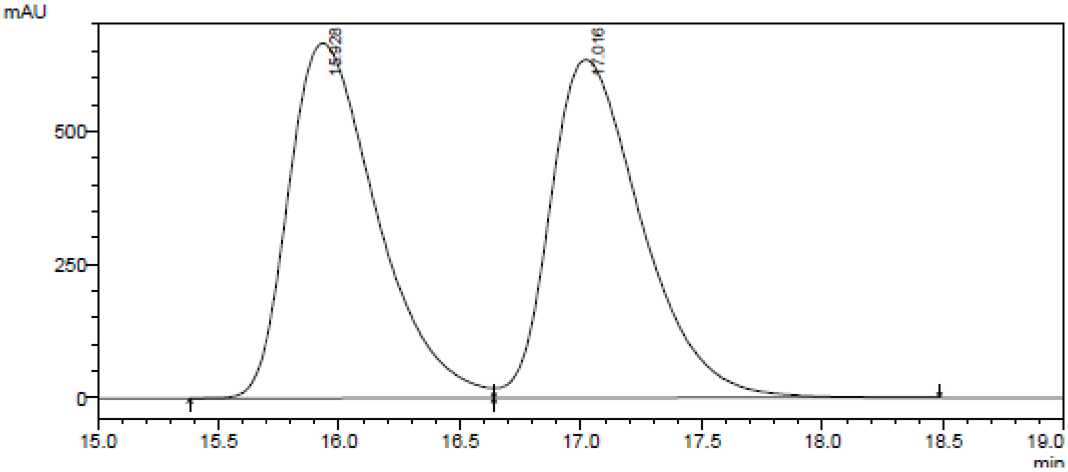

Peak#	Ret. Time	Area	Height	Area %	Height %
1	15.928	16927121	664817	50.388	51.188
2	17.016	16666316	633966	49.612	48.812
Total		33593437	1298783	100.000	100.000

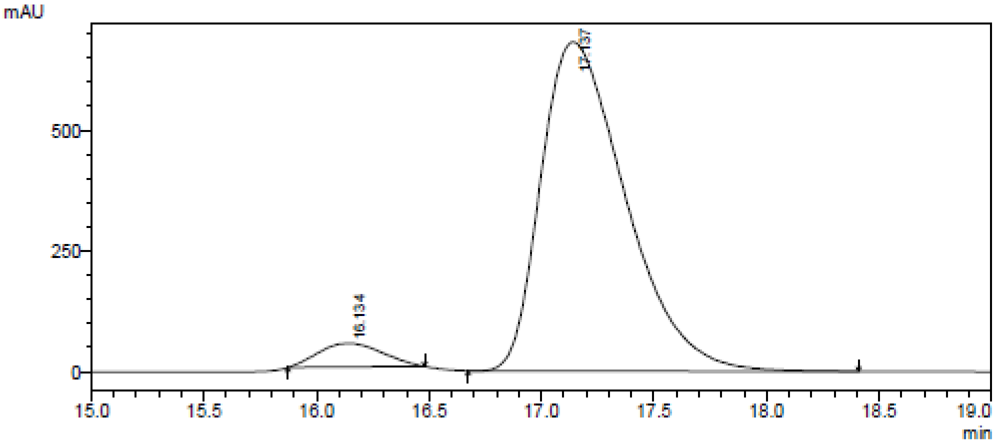

Peak#	Ret. Time	Area	Height	Area %	Height %
1	16.134	976252	49266	5.192	6.744
2	17.137	17825831	681248	94.808	93.256
Total		18802083	730514	100.000	100.000

Supplementary Figure 136 HPLC Spectra of **11**

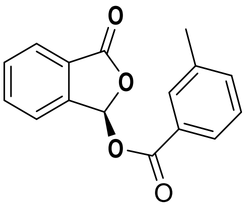

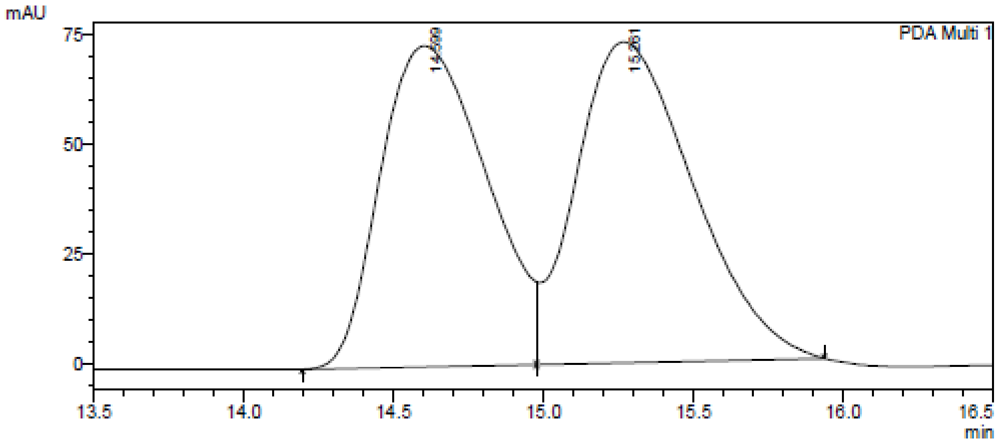

Peak#	Ret. Time	Area	Height	Area %	Height %
1	14.599	1798164	73072	47.593	50.032
2	15.261	1980076	72978	52.407	49.968
Total		3778240	146050	100.000	100.000

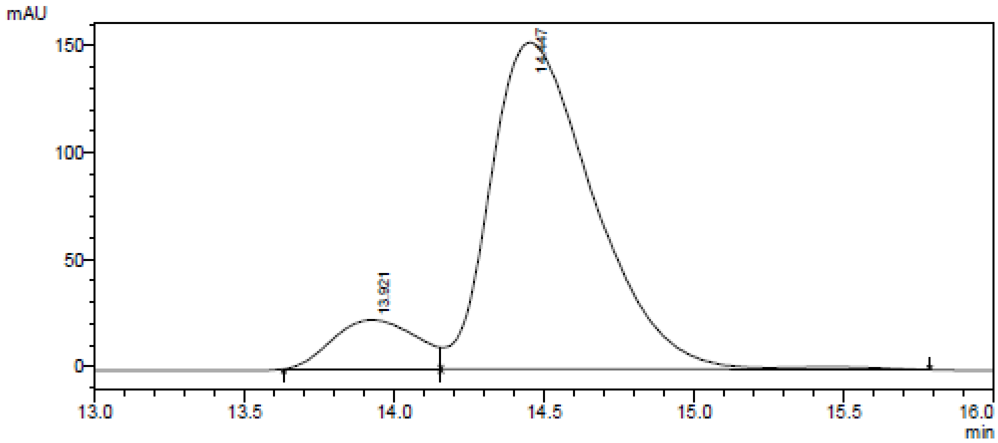

Peak#	Ret. Time	Area	Height	Area %	Height %
1	13.921	451707	23056	10.973	13.107
2	14.447	3664870	152849	89.027	86.893
Total		4116577	175905	100.000	100.000

Supplementary Figure 137 HPLC Spectra of **12**

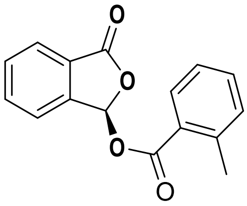

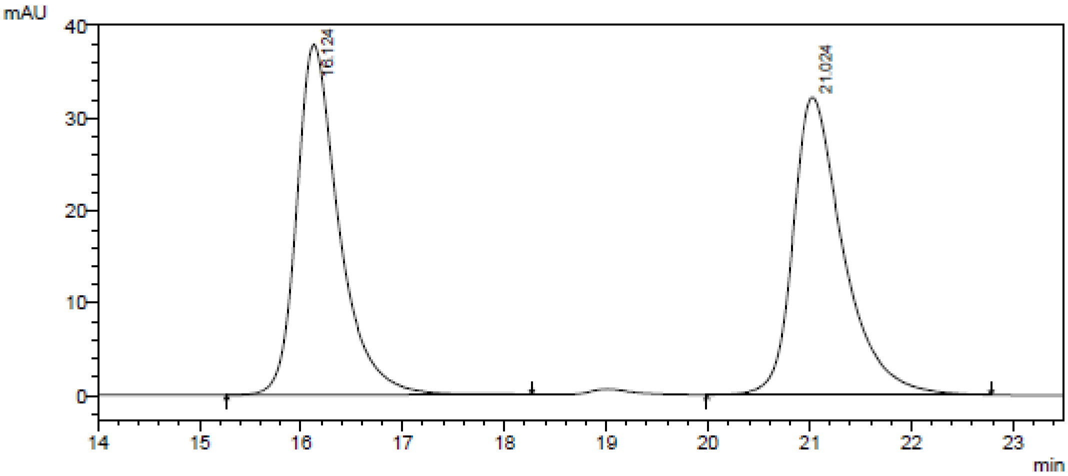

Peak#	Ret. Time	Area	Height	Area %	Height %
1	16.124	9415596	324010	49.709	54.086
2	21.025	9525928	275058	50.291	45.914
Total		18941524	599068	100.000	100.000

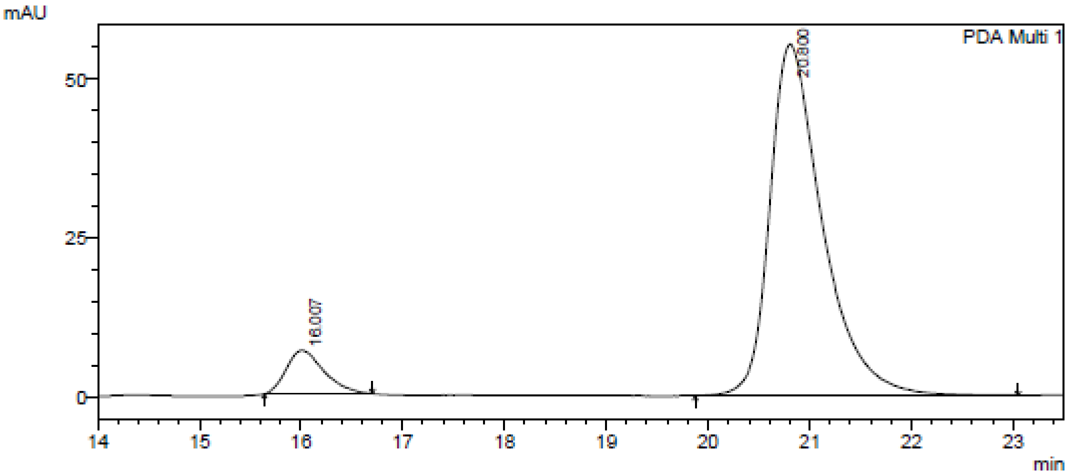

Peak#	Ret. Time	Area	Height	Area %	Height %
1	16.007	177157	6808	8.106	10.975
2	20.800	2008441	55227	91.894	89.025
Total		2185598	62035	100.000	100.000

Supplementary Figure 138 HPLC Spectra of **13**

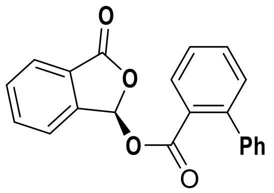

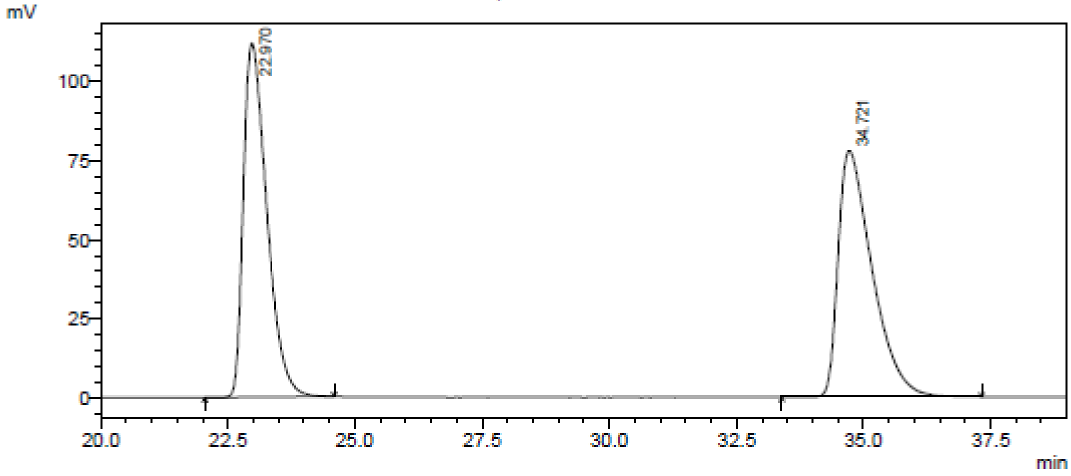

Peak#	Ret. Time	Area	Height	Area %	Height %
1	22.970	3609721	111801	49.893	58.960
2	34.721	3625138	77821	50.107	41.040
Total		7234859	189622	100.000	100.000

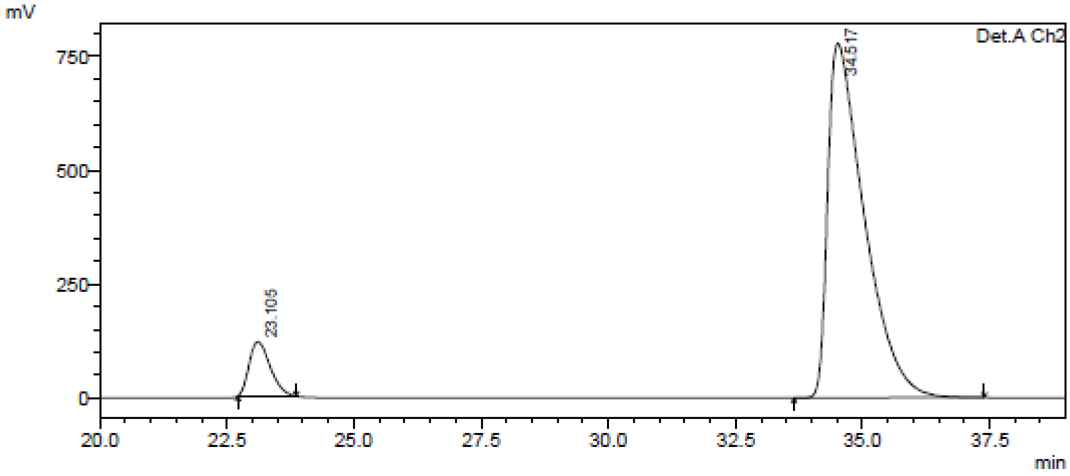

Peak#	Ret. Time	Area	Height	Area %	Height %
1	23.105	3470669	118819	8.198	13.249
2	34.517	38864595	778000	91.802	86.751
Total		42335264	896819	100.000	100.000

Supplementary Figure 139 HPLC Spectra of **14**

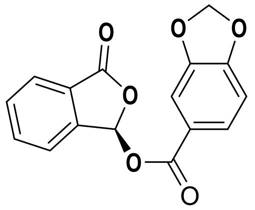

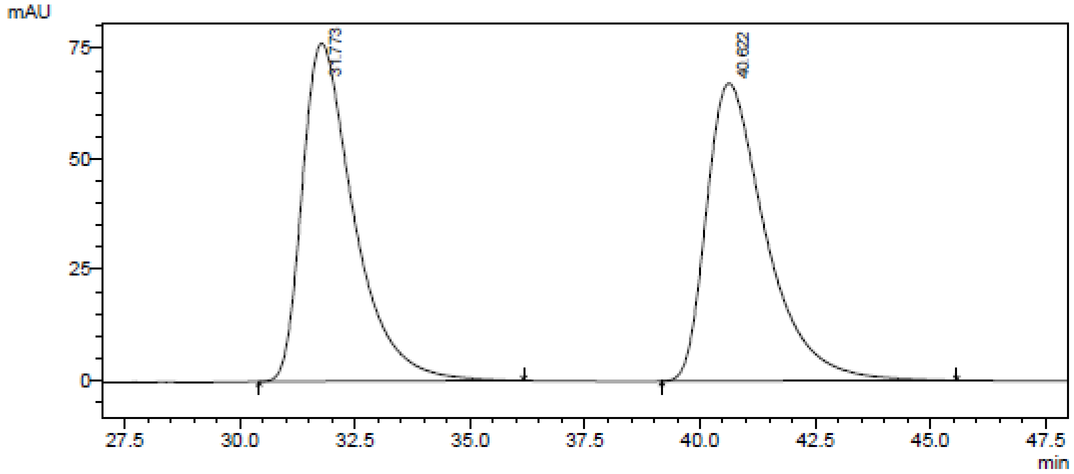

Peak#	Ret. Time	Area	Height	Area %	Height %
1	31.785	1198185	15595	49.765	53.213
2	40.628	1209523	13712	50.235	46.787
Total		2407708	29307	100.000	100.000

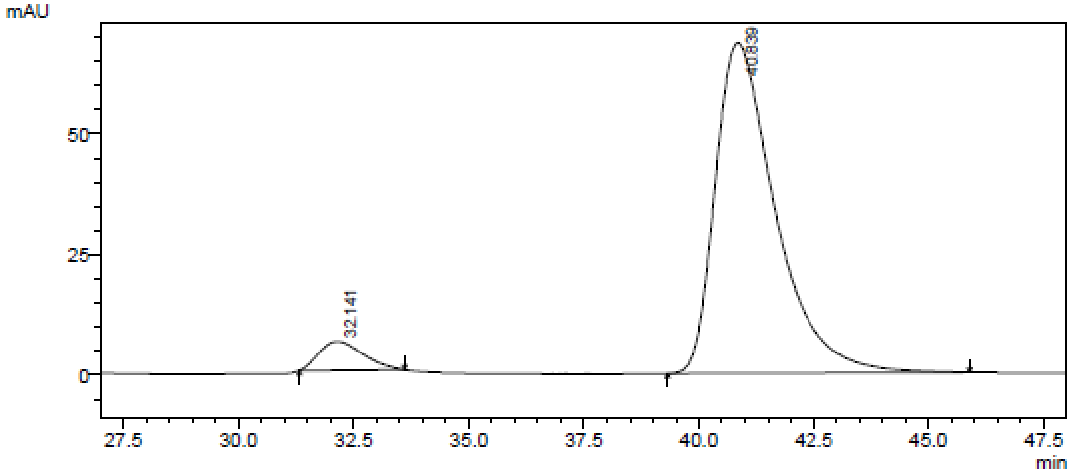

Peak#	Ret. Time	Area	Height	Area %	Height %
1	32.141	401708	6057	6.051	8.138
2	40.839	6237193	68374	93.949	91.862
Total		6638901	74431	100.000	100.000

Supplementary Figure 140 HPLC Spectra of **15**

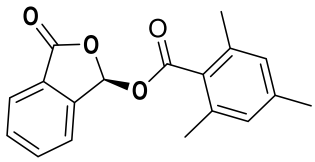

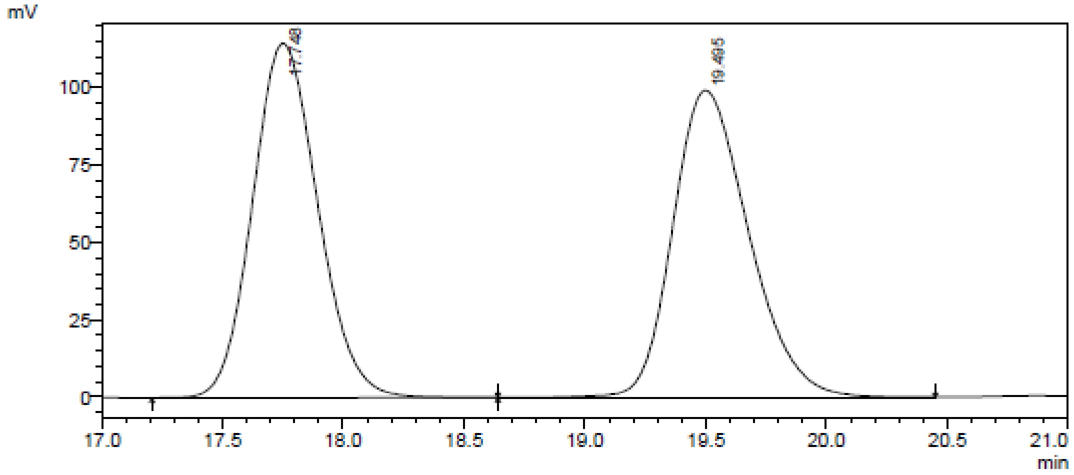

Peak#	Ret. Time	Area	Height	Area %	Height %
1	17.748	2172179	114724	49.816	53.586
2	19.495	2188238	99371	50.184	46.414
Total		4360416	214095	100.000	100.000

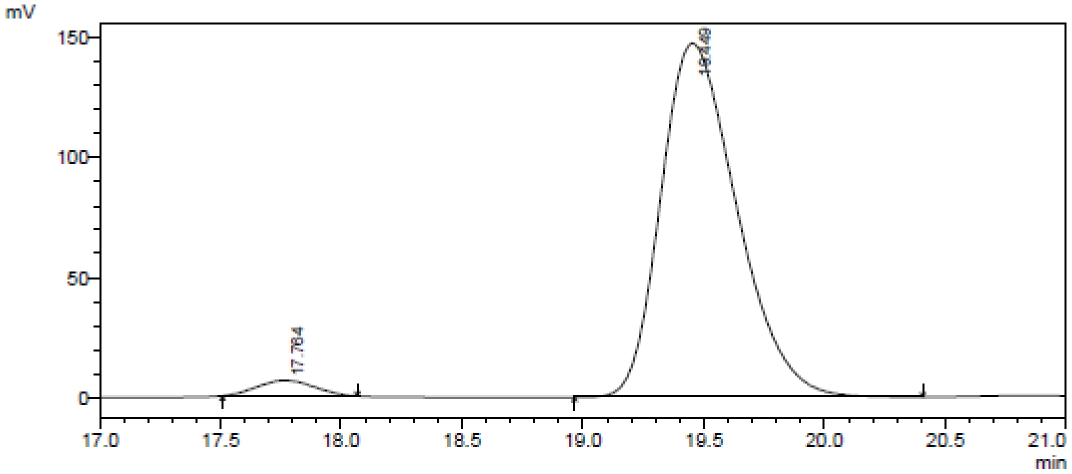

Peak#	Ret. Time	Area	Height	Area %	Height %
1	17.764	109495	6561	3.276	4.261
2	19.449	3233222	147407	96.724	95.739
Total		3342717	153968	100.000	100.000

Supplementary Figure 141 HPLC Spectra of **16**

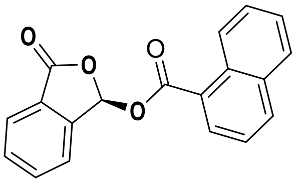

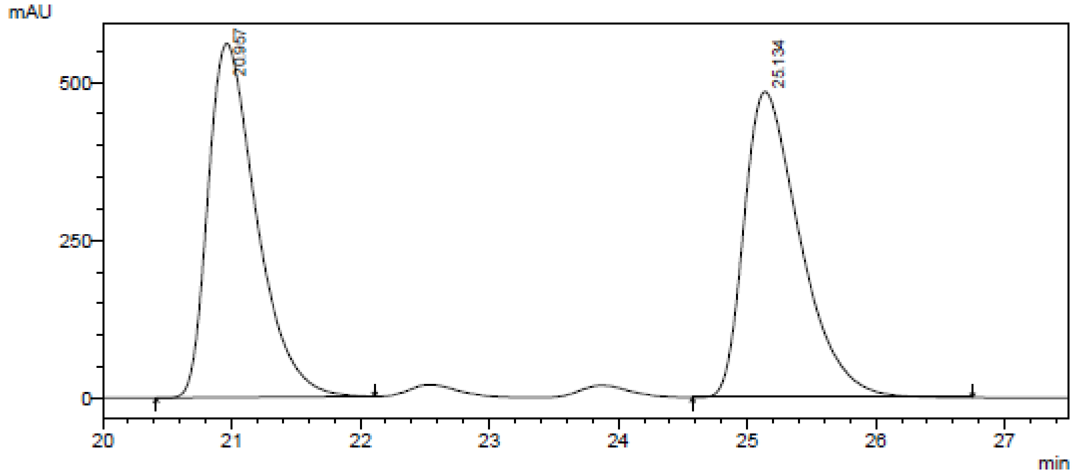

Peak#	Ret. Time	Area	Height	Area %	Height %
1	20.957	14730702	560832	49.975	53.693
2	25.134	14745271	483679	50.025	46.307
Total		29475973	1044511	100.000	100.000

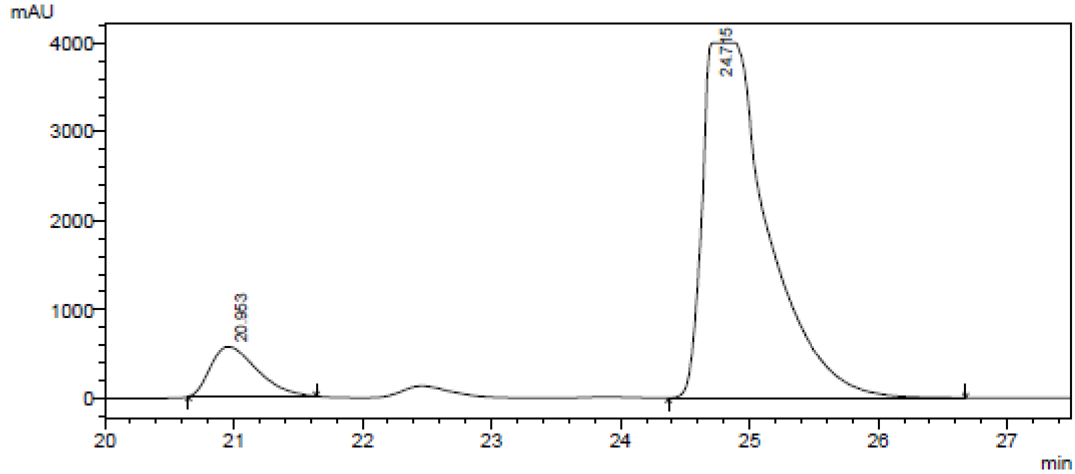

Peak#	Ret. Time	Area	Height	Area %	Height %
1	20.953	14130835	565463	9.075	12.396
2	24.715	141582509	3996159	90.925	87.604
Total		155713344	4561622	100.000	100.000

Supplementary Figure 142 HPLC Spectra of **17**

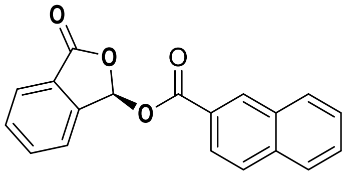

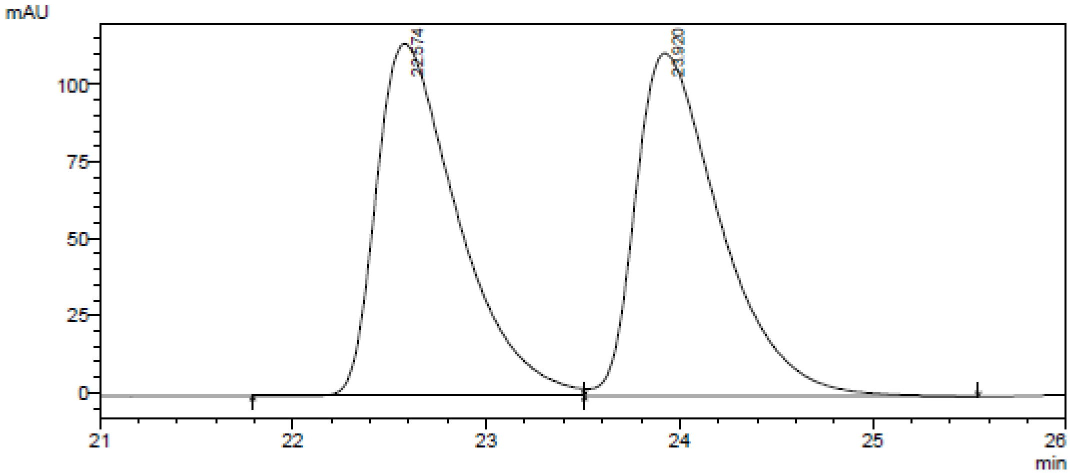

Peak#	Ret. Time	Area	Height	Area %	Height %
1	22.574	3340019	114193	49.634	50.652
2	23.920	3389298	111255	50.366	49.348
Total		6729317	225447	100.000	100.000

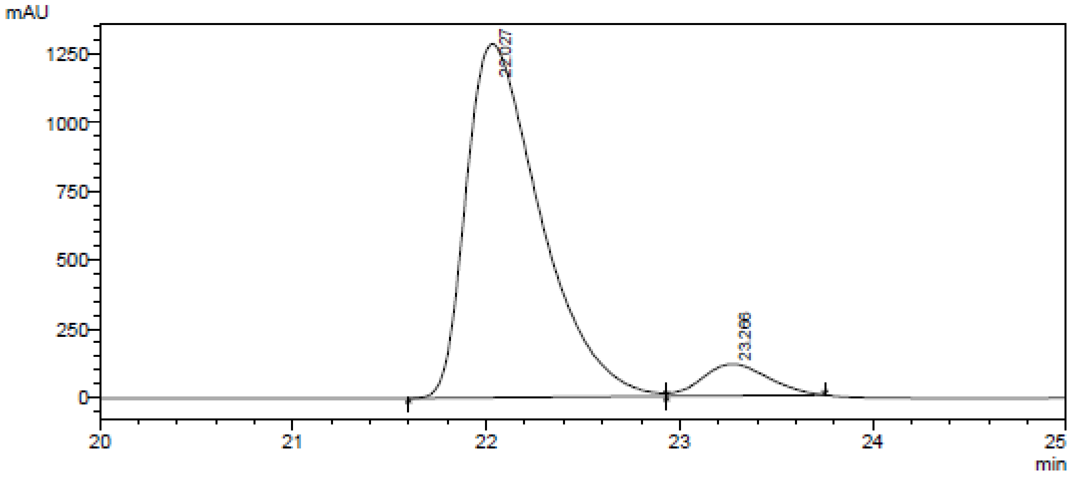

Peak#	Ret. Time	Area	Height	Area %	Height %
1	22.027	34390399	1288742	92.605	91.752
2	23.266	2746119	115843	7.395	8.248
Total		37136518	1404586	100.000	100.000

Supplementary Figure 143 HPLC Spectra of **18**

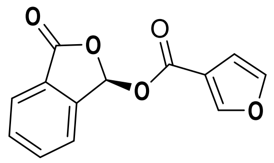

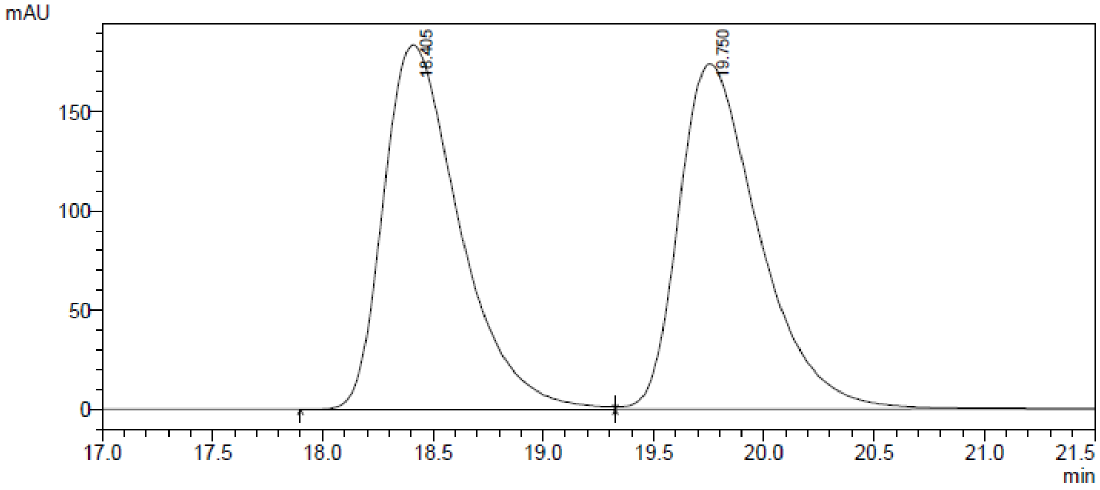

Peak#	Ret. Time	Area	Height	Area %	Height %
1	18.405	4409842	183557	49.956	51.334
2	19.750	4417629	174019	50.044	48.666
Total		8827470	357576	100.000	100.000

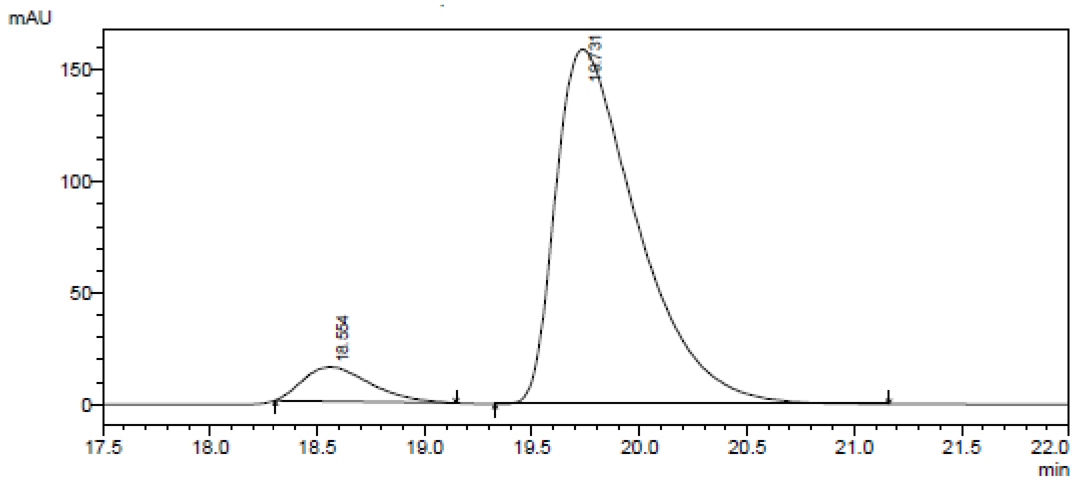

Peak#	Ret. Time	Area	Height	Area %	Height %
1	18.554	331884	15266	7.304	8.762
2	19.731	4211831	158961	92.696	91.238
Total		4543715	174227	100.000	100.000

Supplementary Figure 144 HPLC Spectra of **19**

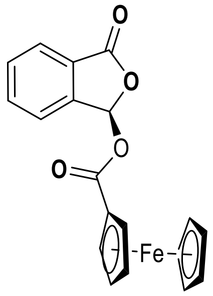

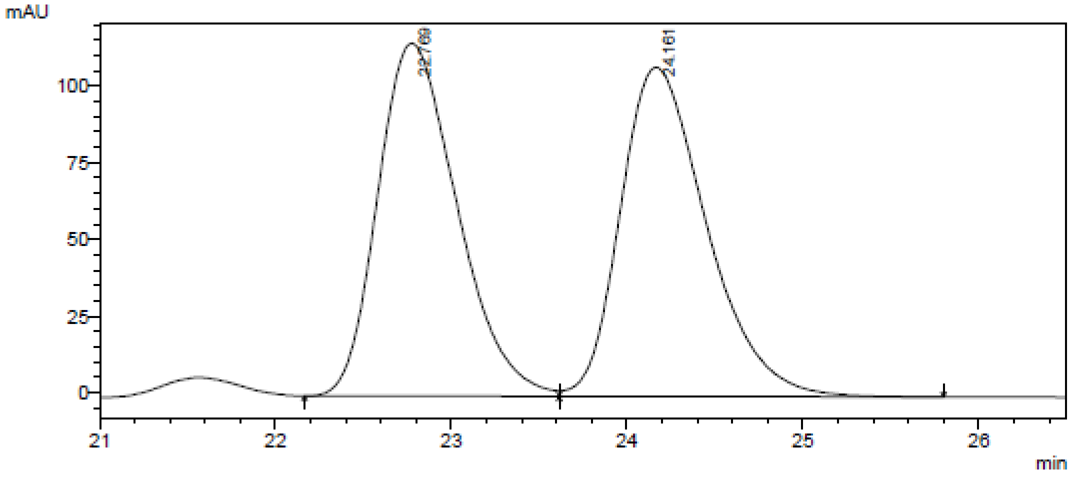

Peak#	Ret. Time	Area	Height	Area %	Height %
1	22.769	610799	19420	49.856	51.773
2	24.162	614332	18090	50.144	48.227
Total		1225131	37510	100.000	100.000

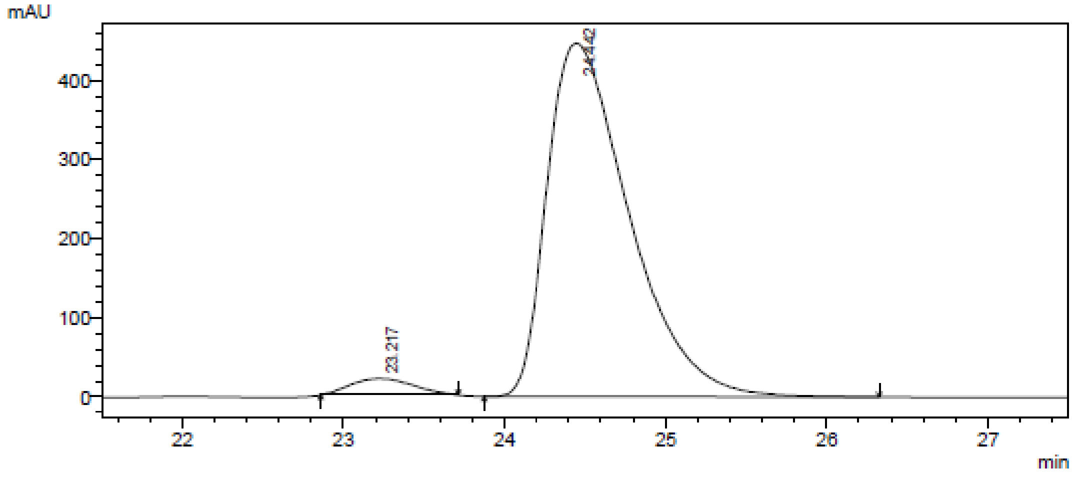

Peak#	Ret. Time	Area	Height	Area %	Height %
1	23.216	94050	3494	3.376	4.380
2	24.442	2691499	76288	96.624	95.620
Total		2785550	79783	100.000	100.000

Supplementary Figure 145 HPLC Spectra of **20**

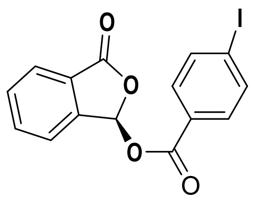

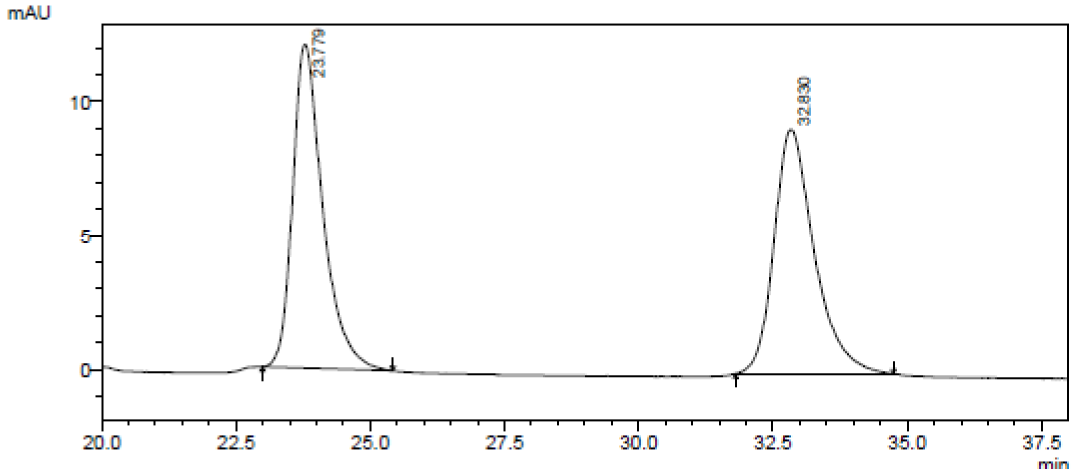

Peak#	Ret. Time	Area	Height	Area %	Height %
1	23.779	471822	12076	49.718	56.959
2	32.830	477173	9125	50.282	43.041
Total		948995	21202	100.000	100.000

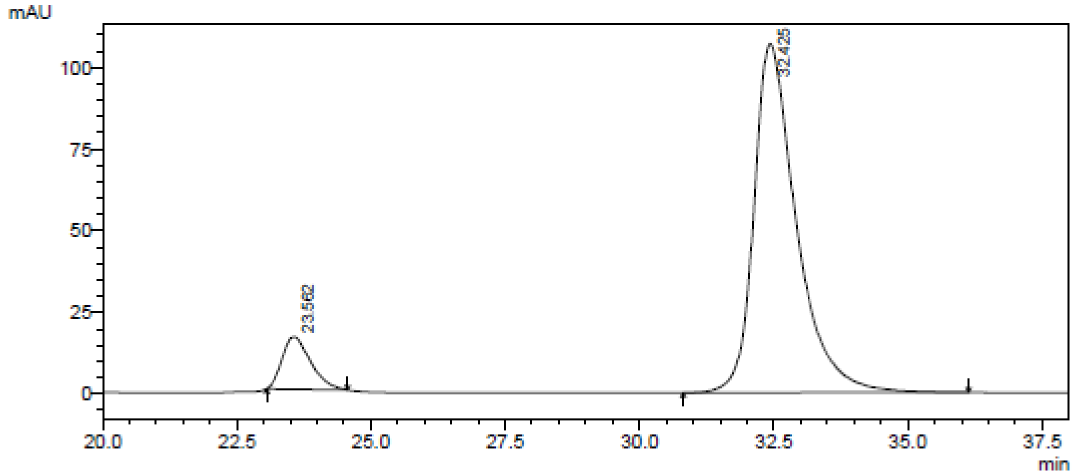

Peak#	Ret. Time	Area	Height	Area %	Height %
1	23.562	599873	16235	9.188	13.179
2	32.425	5928805	106959	90.812	86.821
Total		6528678	123194	100.000	100.000

Supplementary Figure 146 HPLC Spectra of **21**

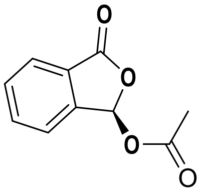

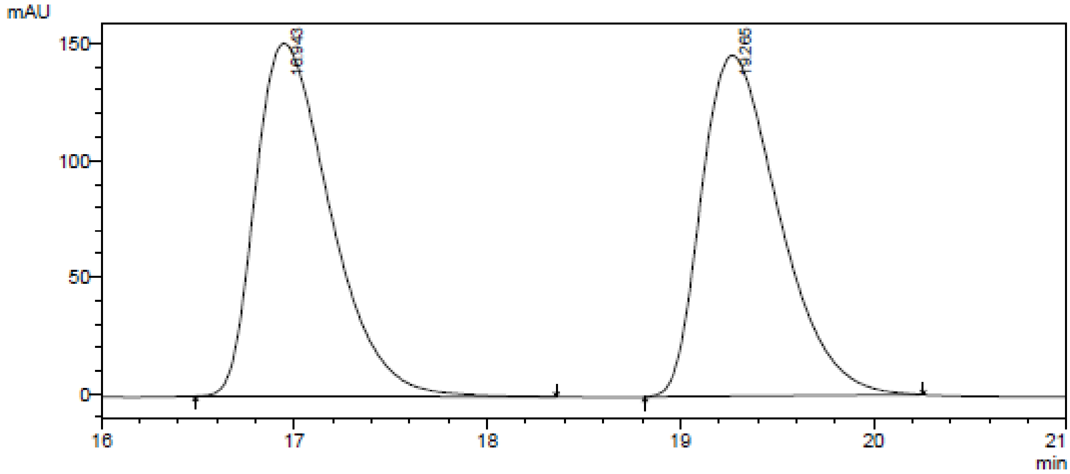

Peak#	Ret. Time	Area	Height	Area %	Height %
1	16.943	4084031	151216	49.843	50.841
2	19.265	4109841	146214	50.157	49.159
Total		8193872	297431	100.000	100.000

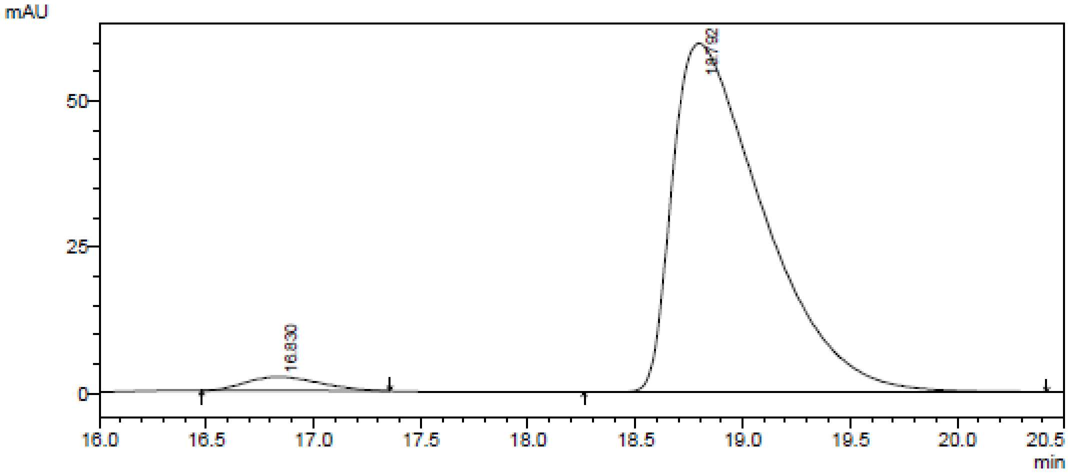

Peak#	Ret. Time	Area	Height	Area %	Height %
1	16.830	56142	2335	3.085	3.766
2	18.792	1763451	59651	96.915	96.234
Total		1819593	61985	100.000	100.000

Supplementary Figure 147 HPLC Spectra of **22**

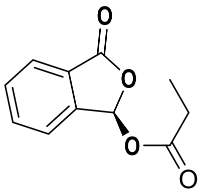

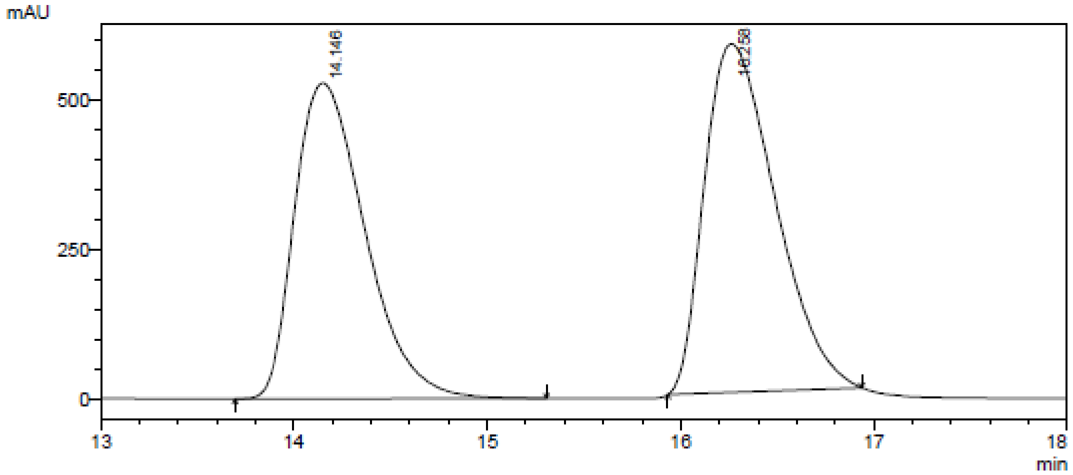

Peak#	Ret. Time	Area	Height	Area %	Height %
1	14.146	13384694	527325	47.355	47.549
2	16.258	14880151	581679	52.645	52.451
Total		28264844	1109003	100.000	100.000

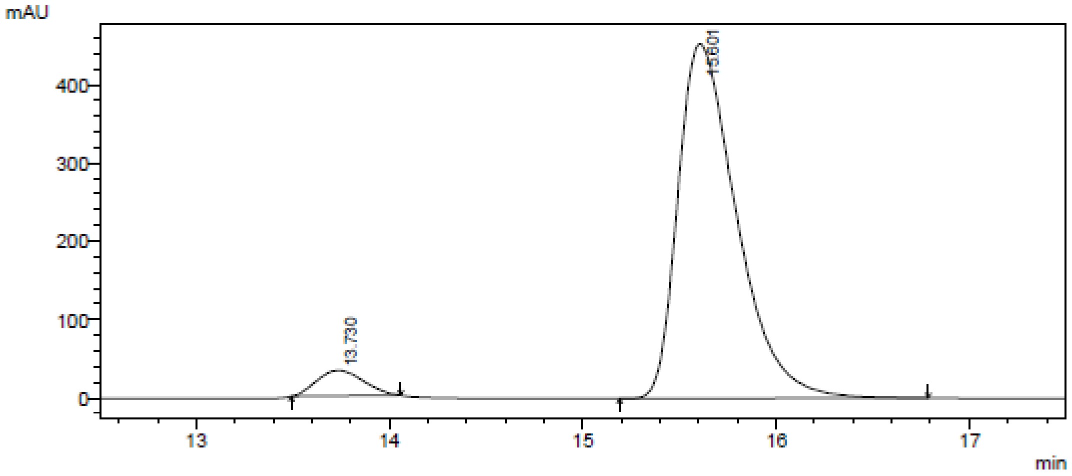

Peak#	Ret. Time	Area	Height	Area %	Height %
1	13.730	548919	32639	5.435	6.722
2	15.601	9551397	452939	94.565	93.278
Total		10100317	485577	100.000	100.000

Supplementary Figure 148 HPLC Spectra of **23**

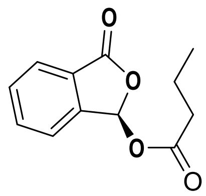

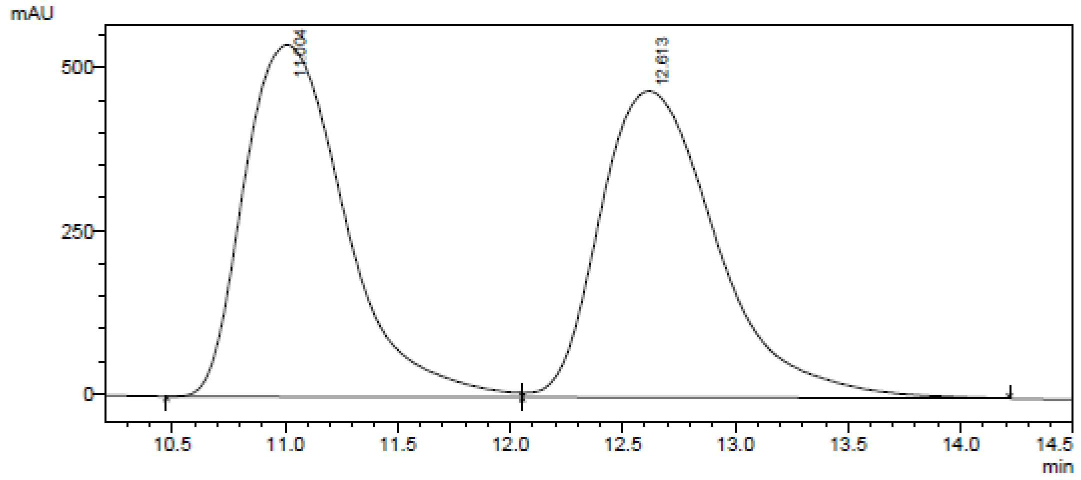

Peak#	Ret. Time	Area	Height	Area %	Height %
1	11.004	16756517	538951	49.623	53.452
2	12.613	17010906	469332	50.377	46.548
Total		33767423	1008283	100.000	100.000

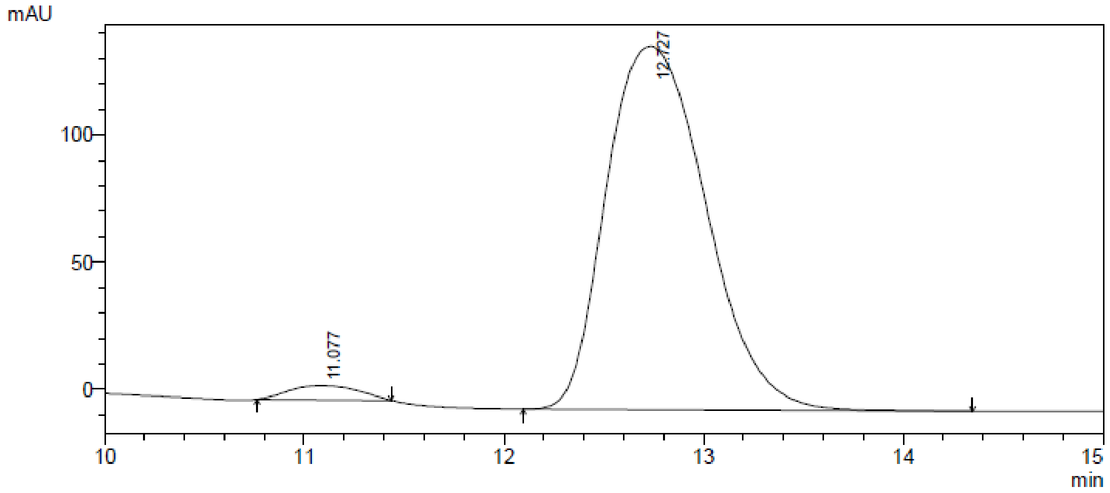

Peak#	Ret. Time	Area	Height	Area %	Height %
1	11.077	177180	6437	3.445	4.313
2	12.727	4966453	142791	96.555	95.687
Total		5143633	149227	100.000	100.000

Supplementary Figure 149 HPLC Spectra of **24**

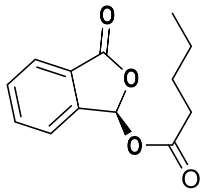

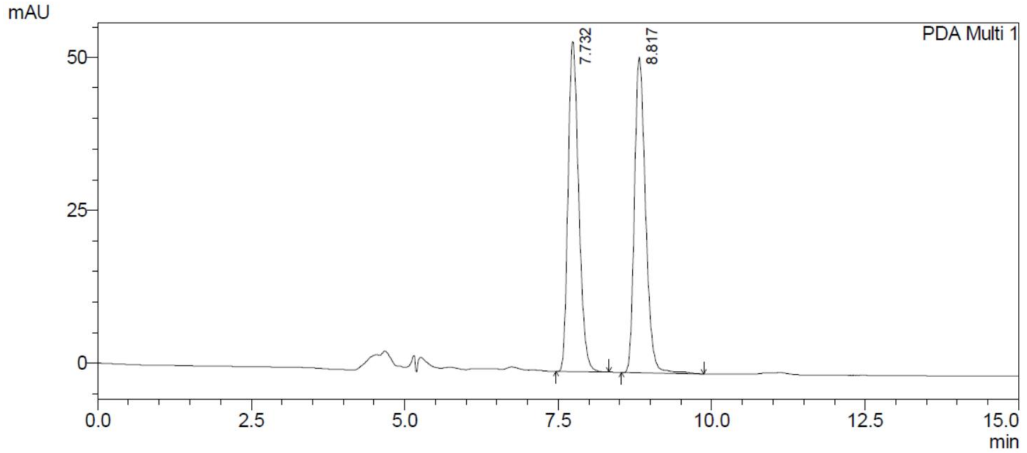

PDA Ch1 254nm 4nm

Peak#	Ret. Time	Area	Height	Area %	Height %
1	7.732	651656	53914	50.020	51.079
2	8.817	651129	51637	49.980	48.921
Total		1302785	105551	100.000	100.000

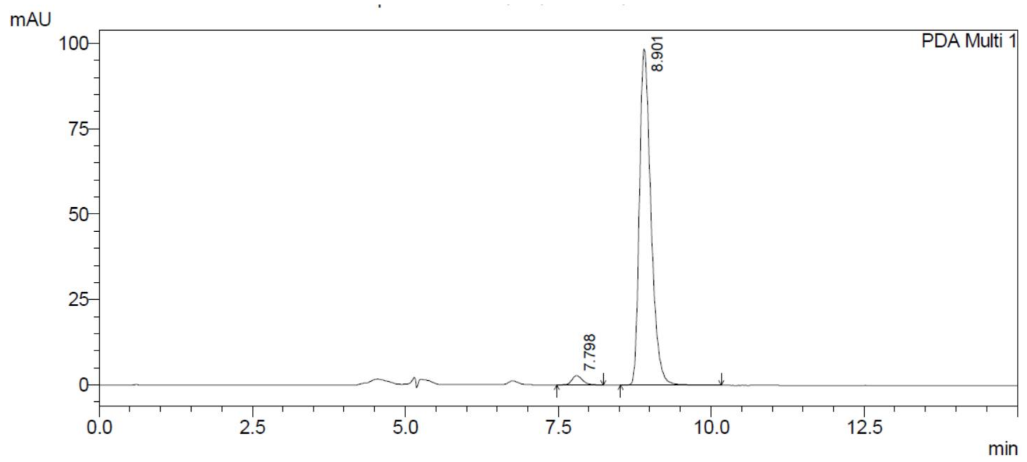

PDA Ch1 254nm 4nm

Peak#	Ret. Time	Area	Height	Area %	Height %
1	7.798	33808	2752	2.555	2.721
2	8.901	1289645	98406	97.445	97.279
Total		1323453	101158	100.000	100.000

Supplementary Figure 150 HPLC Spectra of **25**

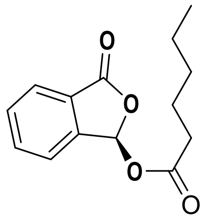

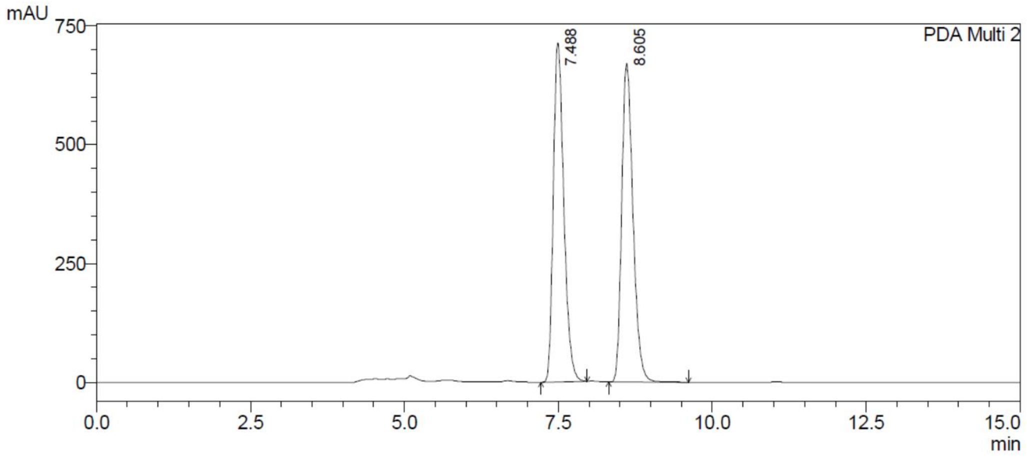

PDA Ch2 220nm 4nm

Peak#	Ret. Time	Area	Height	Area %	Height %
1	7.488	8451266	712522	49.685	51.515
2	8.605	8558513	670607	50.315	48.485
Total		17009779	1383129	100.000	100.000

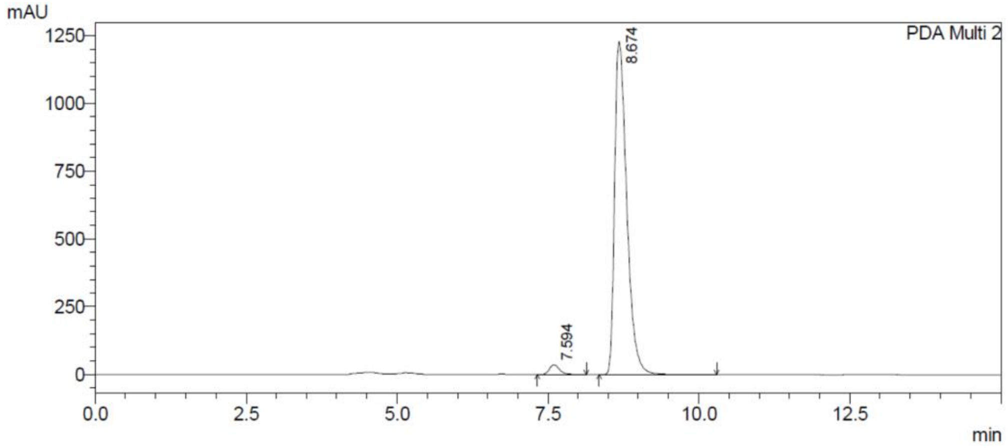

PDA Ch2 220nm 4nm

Peak#	Ret. Time	Area	Height	Area %	Height %
1	7.594	430079	36042	2.438	2.848
2	8.674	17212508	1229683	97.562	97.152
Total		17642588	1265725	100.000	100.000

Supplementary Figure 151 HPLC Spectra of **26**

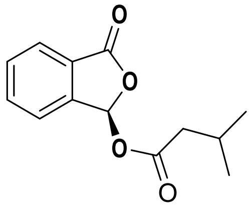

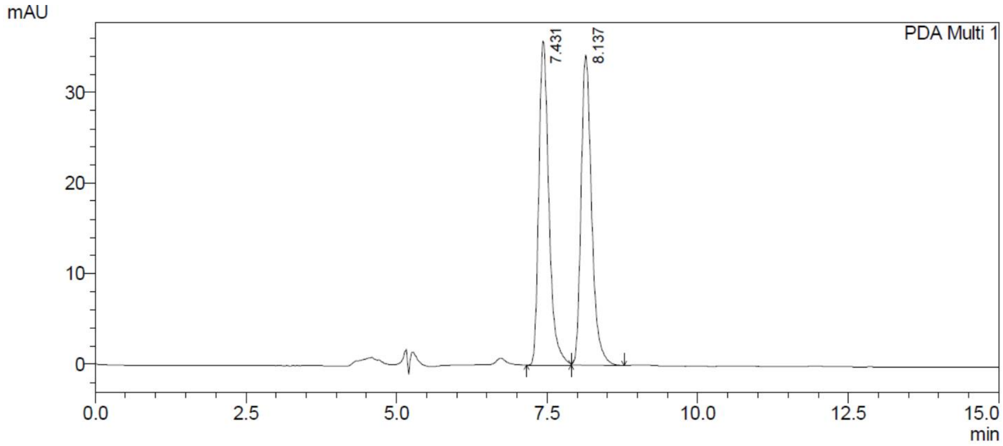

PDA Ch1 254nm 4nm

Peak#	Ret. Time	Area	Height	Area %	Height %
1	7.431	417362	35735	50.022	51.077
2	8.137	416987	34228	49.978	48.923
Total		834349	69964	100.000	100.000

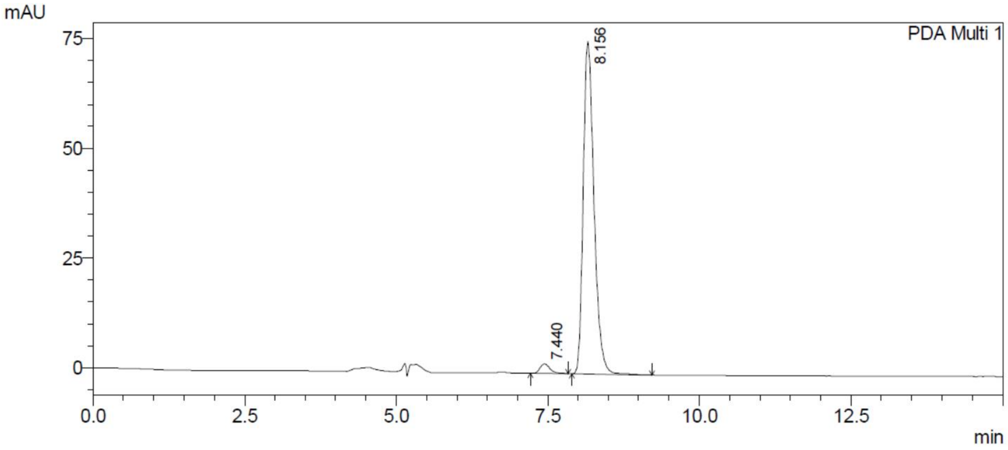

PDA Ch1 254nm 4nm

Peak#	Ret. Time	Area	Height	Area %	Height %
1	7.440	25982	2225	2.699	2.853
2	8.156	936758	75749	97.301	97.147
Total		962741	77974	100.000	100.000

Supplementary Figure 152 HPLC Spectra of **27**

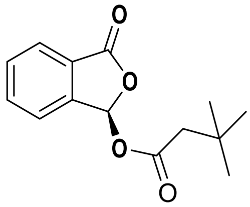

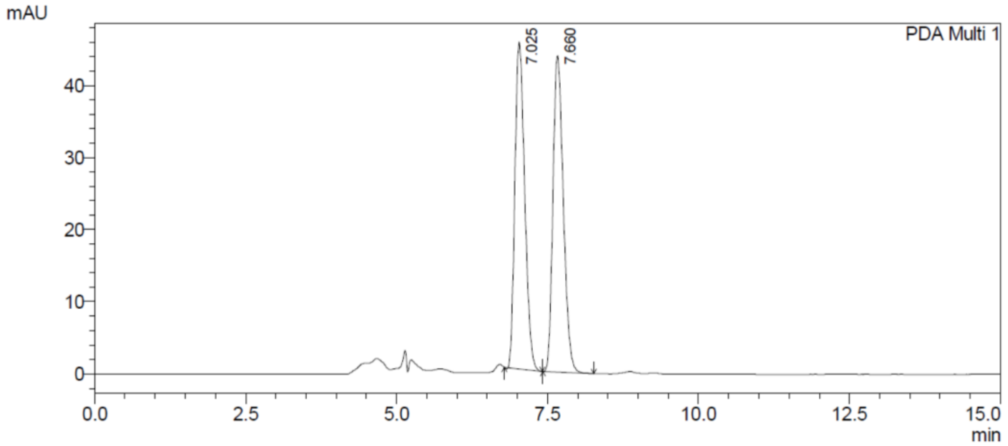

PDA Ch1 254nm 4nm

Peak#	Ret. Time	Area	Height	Area %	Height %
1	7.025	513446	45342	49.494	50.816
2	7.660	523934	43885	50.506	49.184
Total		1037380	89227	100.000	100.000

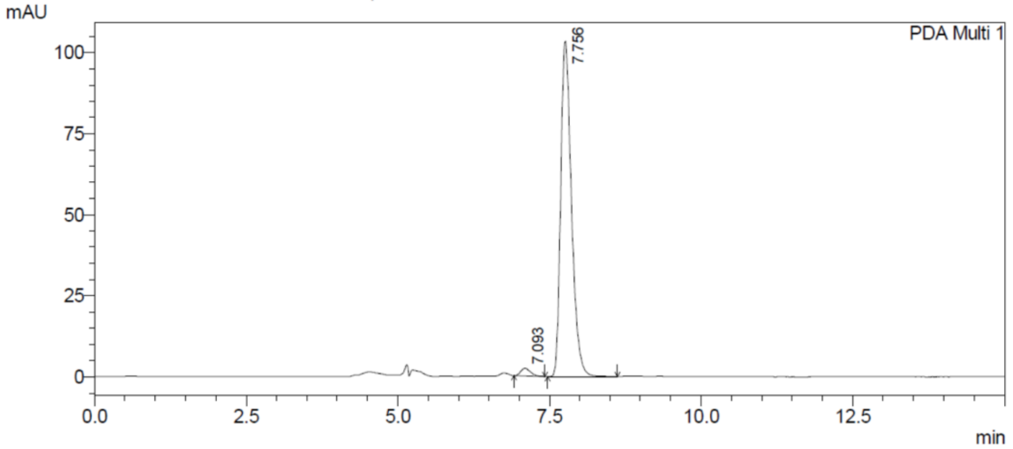

PDA Ch1 254nm 4nm

Peak#	Ret. Time	Area	Height	Area %	Height %
1	7.093	25909	2357	1.991	2.229
2	7.756	1275277	103393	98.009	97.771
Total		1301186	105751	100.000	100.000

Supplementary Figure 153 HPLC Spectra of **28**

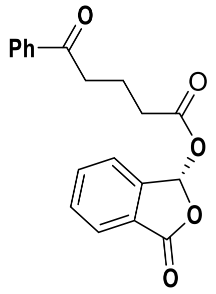

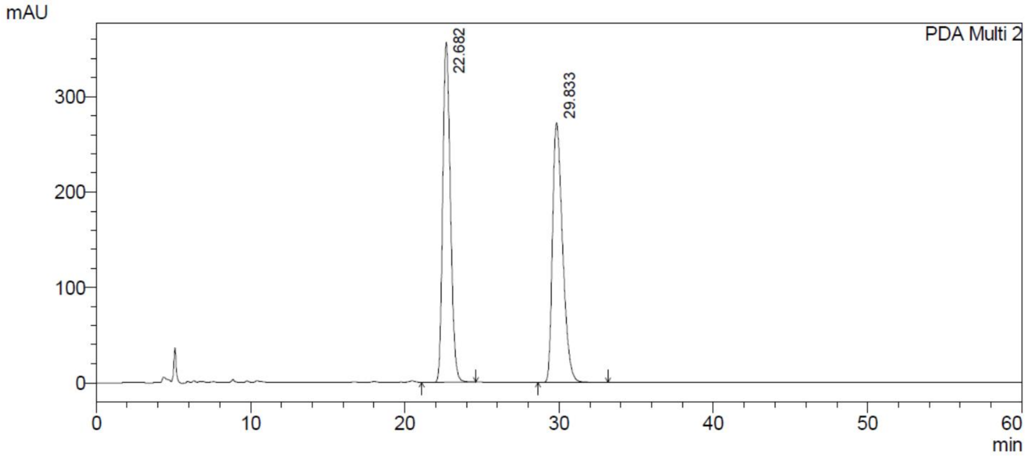

PDA Ch2 220nm 4nm

Peak#	Ret. Time	Area	Height	Area %	Height %
1	22.682	12324216	356400	49.847	56.695
2	29.833	12399672	272230	50.153	43.305
Total		24723888	628630	100.000	100.000

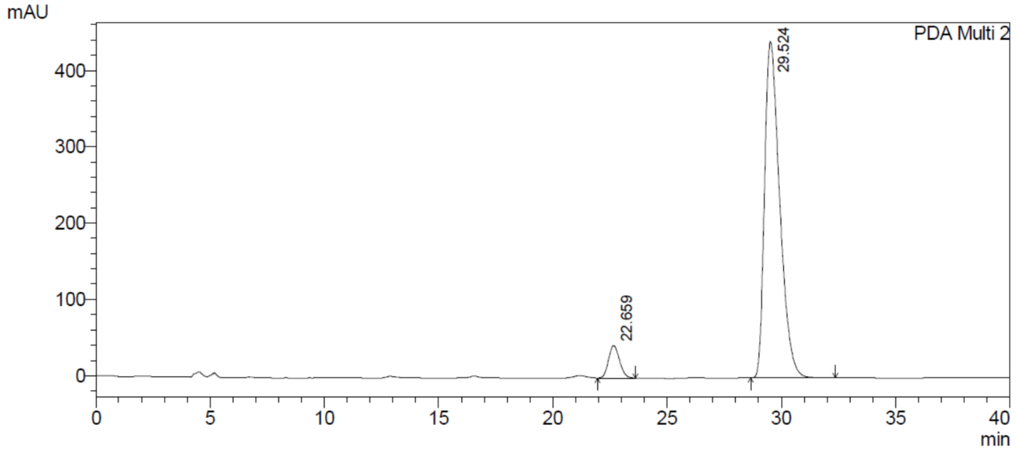

PDA Ch2 220nm 4nm

Peak#	Ret. Time	Area	Height	Area %	Height %
1	22.659	1430082	42564	6.568	8.810
2	29.524	20344940	440538	93.432	91.190
Total		21775022	483101	100.000	100.000

Supplementary Figure 154 HPLC Spectra of **29**

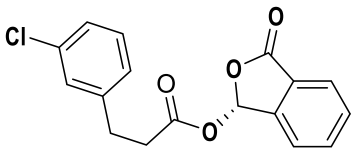

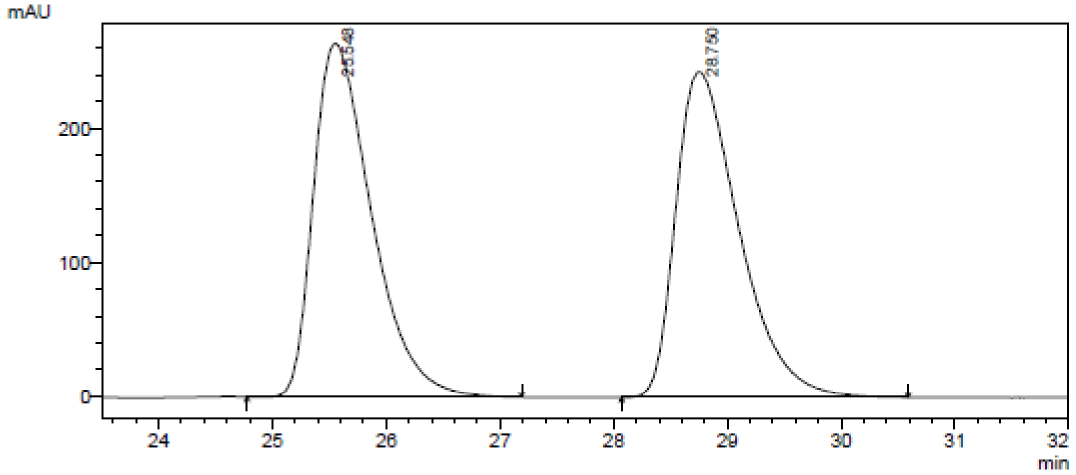

Peak#	Ret. Time	Area	Height	Area %	Height %
1	25.548	9400164	264448	49.902	52.033
2	28.750	9437166	243784	50.098	47.967
Total		18837330	508232	100.000	100.000

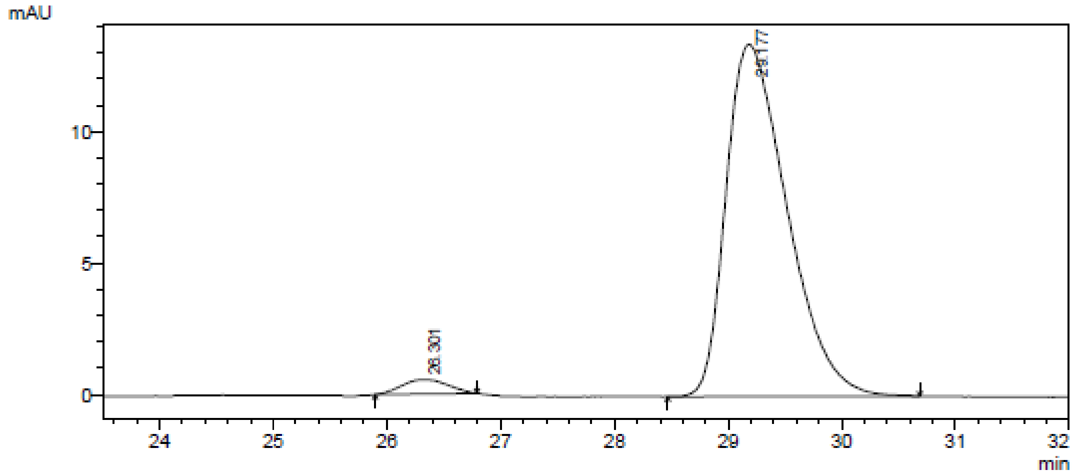

Peak#	Ret. Time	Area	Height	Area %	Height %
1	26.301	15597	554	3.028	3.973
2	29.177	499445	13392	96.972	96.027
Total		515042	13947	100.000	100.000

Supplementary Figure 155 HPLC Spectra of **30**

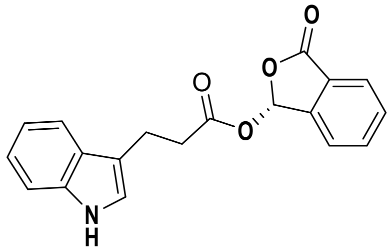

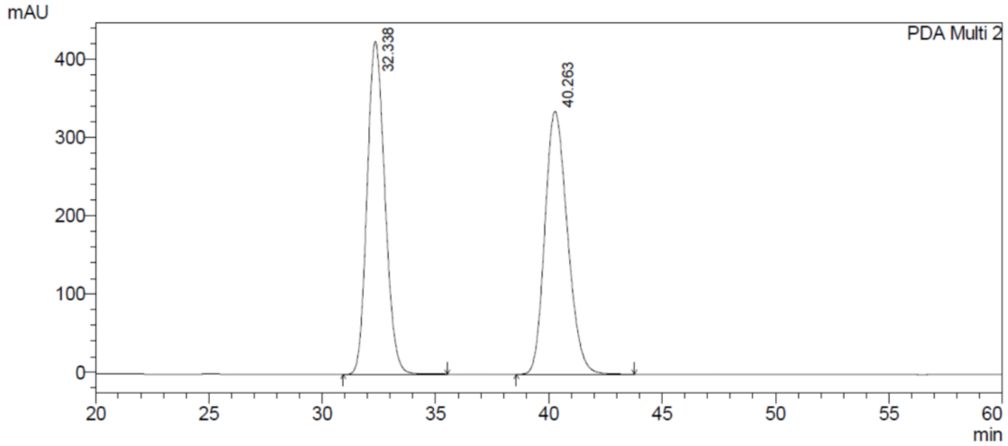

PDA Ch2 220nm 4nm

Peak#	Ret. Time	Area	Height	Area %	Height %
1	32.338	23237657	425153	49.880	55.875
2	40.263	23349774	335746	50.120	44.125
Total		46587431	760899	100.000	100.000

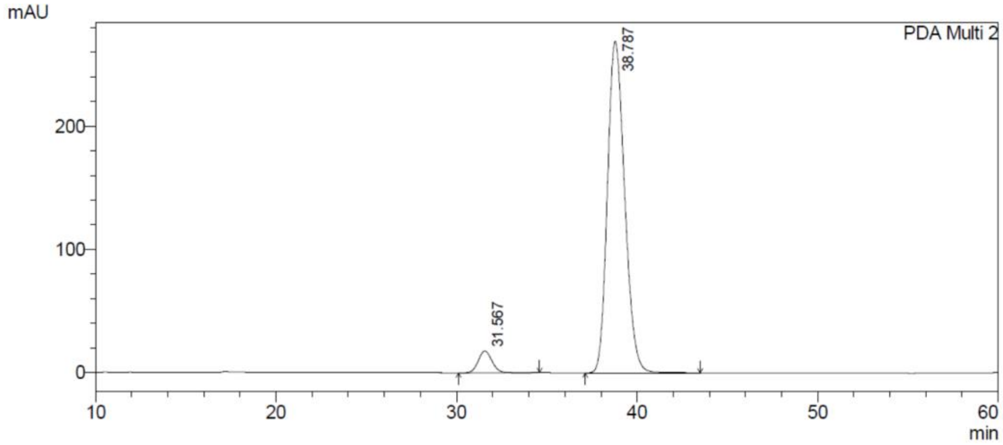

PDA Ch2 220nm 4nm

Peak#	Ret. Time	Area	Height	Area %	Height %
1	31.567	932473	17766	4.964	6.185
2	38.787	17852691	269454	95.036	93.815
Total		18785165	287220	100.000	100.000

Supplementary Figure 156 HPLC Spectra of **31**

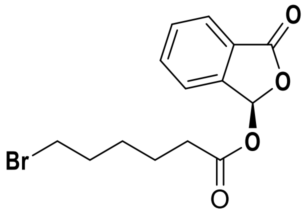

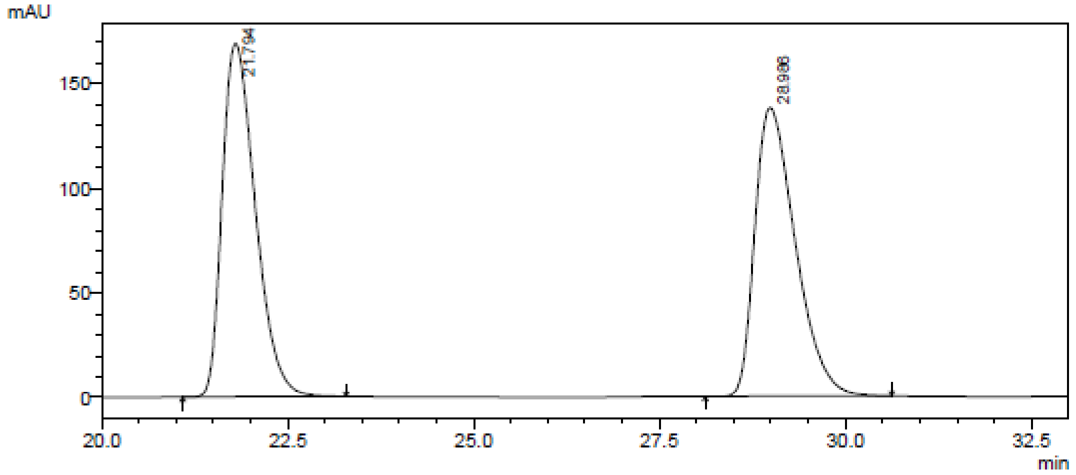

Peak#	Ret. Time	Area	Height	Area %	Height %
1	21.794	5241363	168888	50.035	55.064
2	28.986	5234023	137823	49.965	44.936
Total		10475386	306710	100.000	100.000

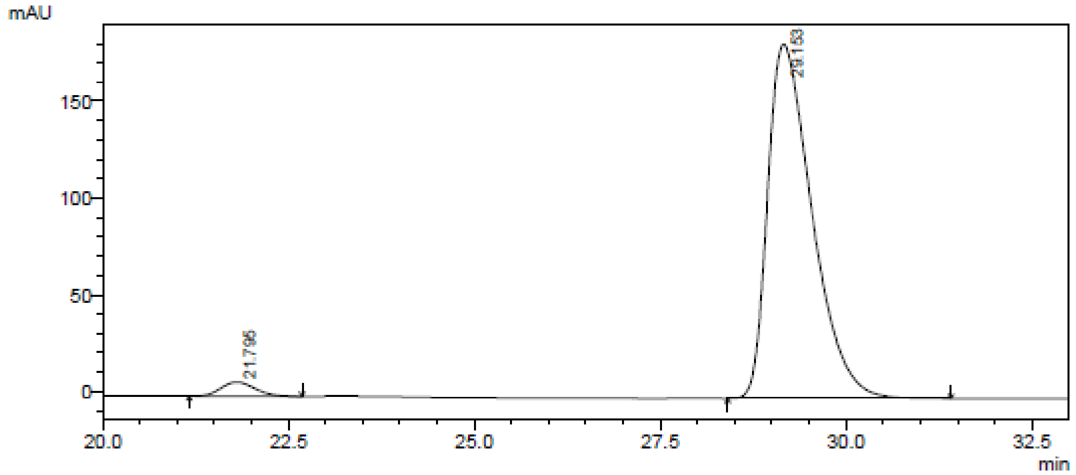

Peak#	Ret. Time	Area	Height	Area %	Height %
1	21.795	238786	7403	3.028	3.885
2	29.153	7647390	183135	96.972	96.115
Total		7886176	190538	100.000	100.000

Supplementary Figure 157 HPLC Spectra of **32**

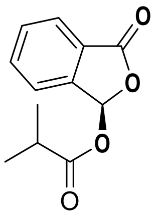

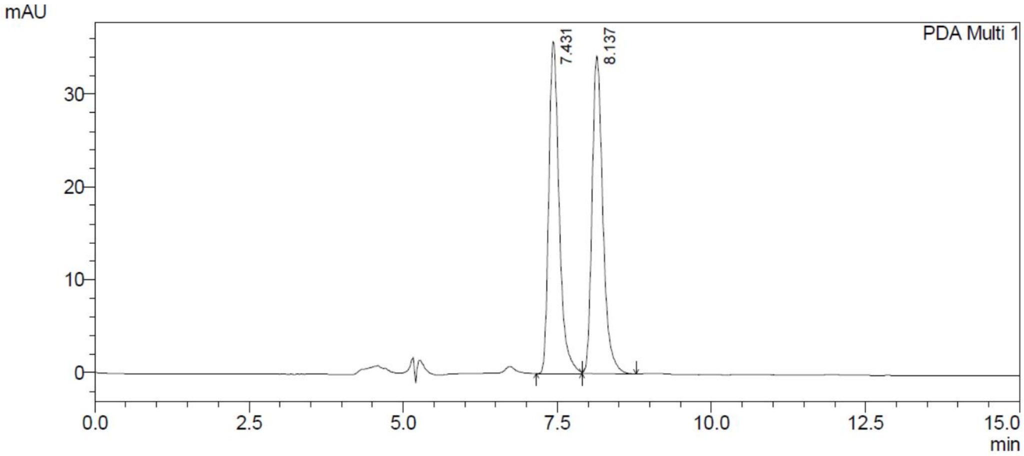

PDA Ch1 254nm 4nm

Peak#	Ret. Time	Area	Height	Area %	Height %
1	7.431	417362	35735	50.022	51.077
2	8.137	416987	34228	49.978	48.923
Total		834349	69964	100.000	100.000

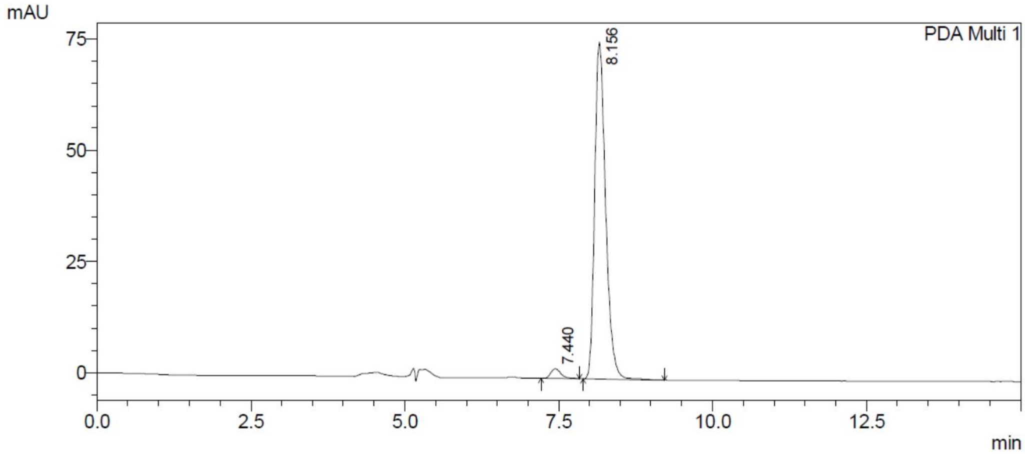

PDA Ch1 254nm 4nm

Peak#	Ret. Time	Area	Height	Area %	Height %
1	7.440	25982	2225	2.699	2.853
2	8.156	936758	75749	97.301	97.147
Total		962741	77974	100.000	100.000

Supplementary Figure 158 HPLC Spectra of **33**

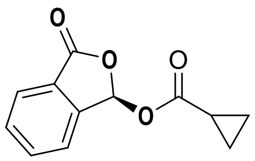

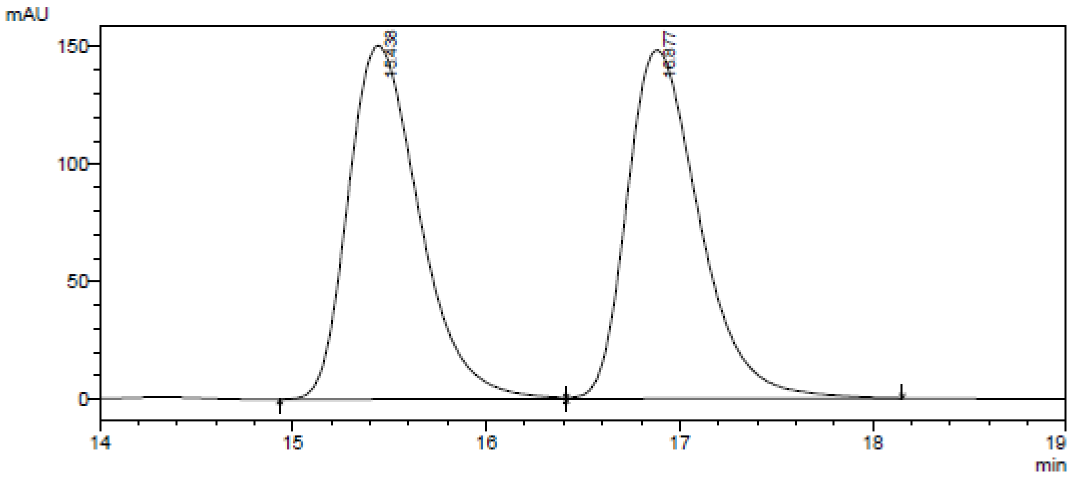

Peak#	Ret. Time	Area	Height	Area %	Height %
1	15.438	3836151	150242	49.815	50.311
2	16.877	3864685	148382	50.185	49.689
Total		7700836	298624	100.000	100.000

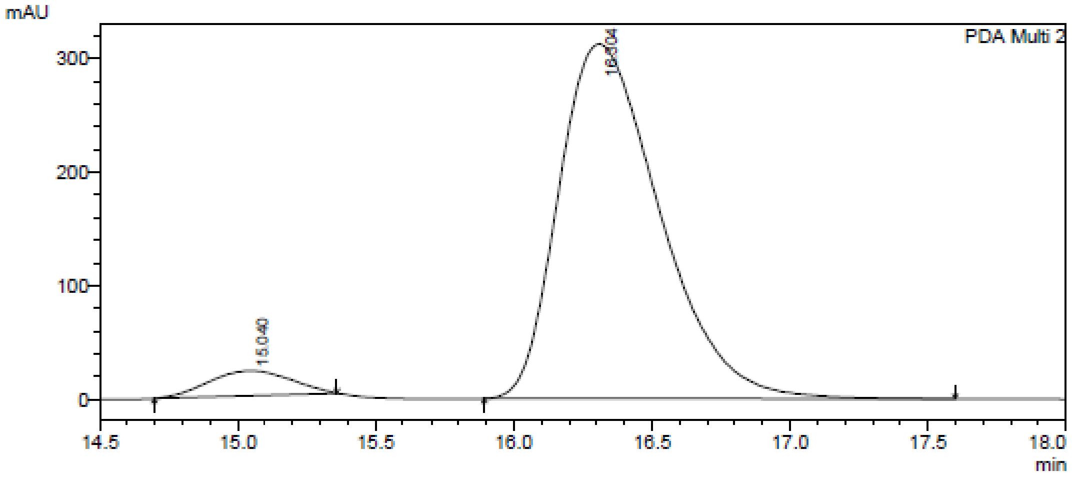

Peak#	Ret. Time	Area	Height	Area %	Height %
1	15.040	452253	21734	5.387	6.508
2	16.304	7943620	312200	94.613	93.492
Total		8395873	333934	100.000	100.000

Supplementary Figure 159 HPLC Spectra of **34**

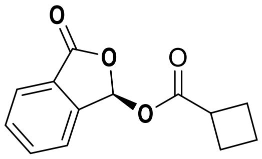

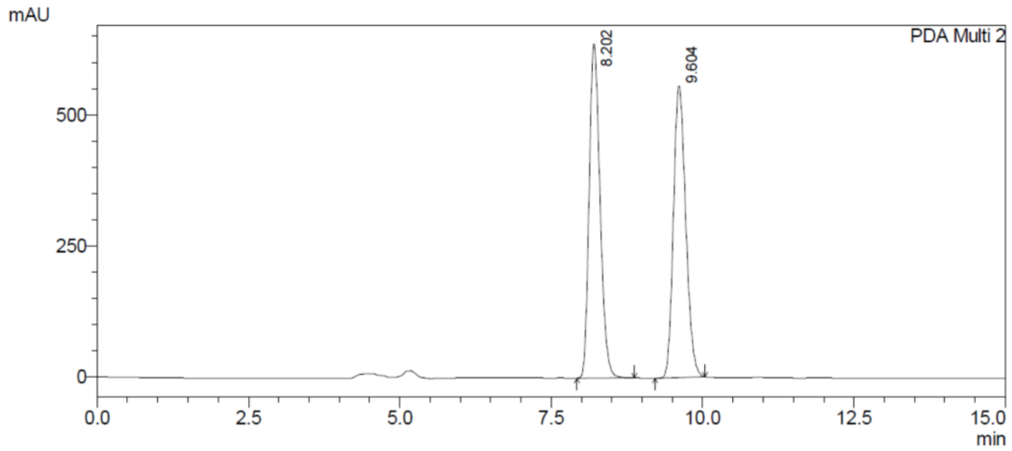

PDA Ch2 220nm 4nm

Peak#	Ret. Time	Area	Height	Area %	Height %
1	8.202	7986314	637337	50.375	53.350
2	9.604	7867295	557293	49.625	46.650
Total		15853610	1194630	100.000	100.000

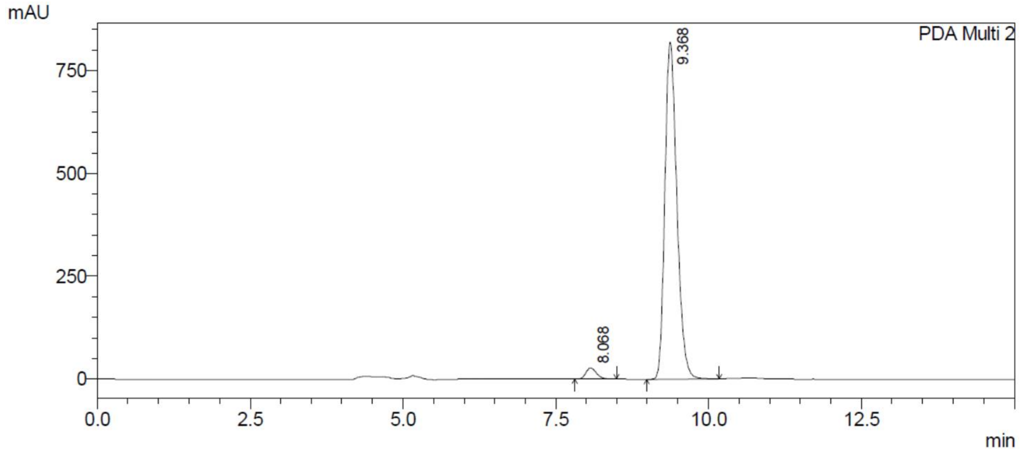

PDA Ch2 220nm 4nm

Peak#	Ret. Time	Area	Height	Area %	Height %
1	8.068	327039	27293	2.841	3.224
2	9.368	11184728	819377	97.159	96.776
Total		11511768	846670	100.000	100.000

Supplementary Figure 160 HPLC Spectra of **35**

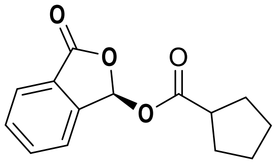

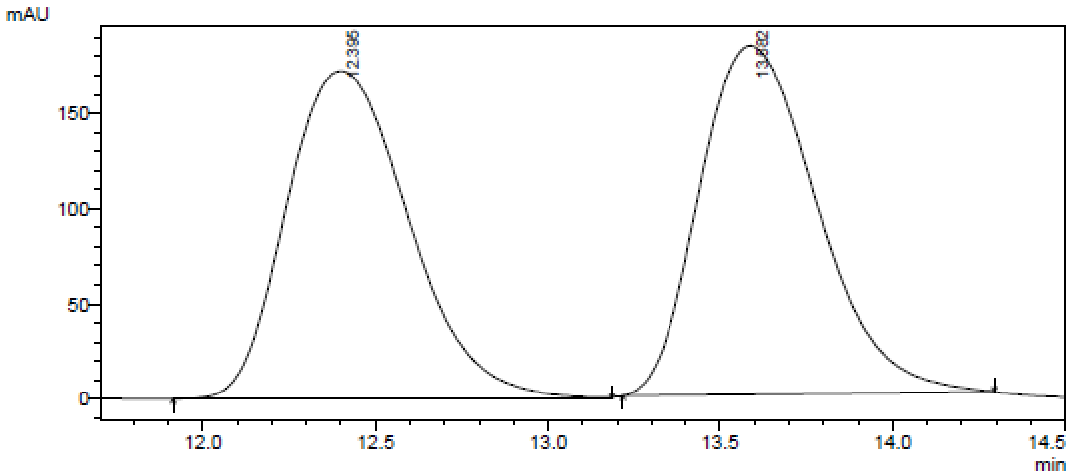

Peak#	Ret. Time	Area	Height	Area %	Height %
1	12.395	4247815	172312	49.436	48.458
2	13.582	4344784	183278	50.564	51.542
Total		8592599	355590	100.000	100.000

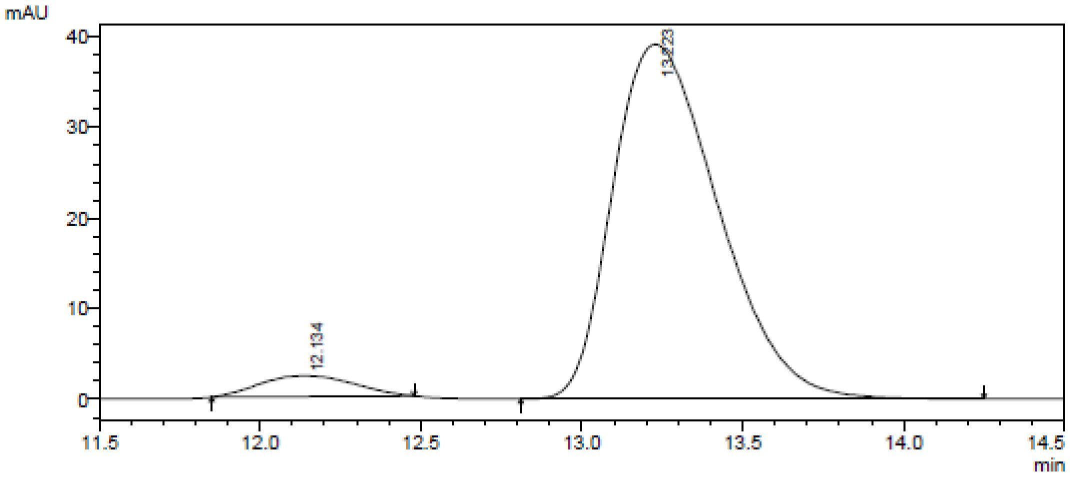

Peak#	Ret. Time	Area	Height	Area %	Height %
1	12.134	46553	2294	4.951	5.545
2	13.223	893820	39067	95.049	94.455
Total		940373	41360	100.000	100.000

Supplementary Figure 161 HPLC Spectra of **36**

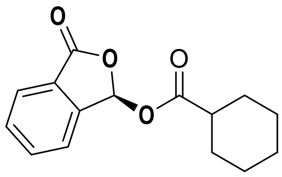

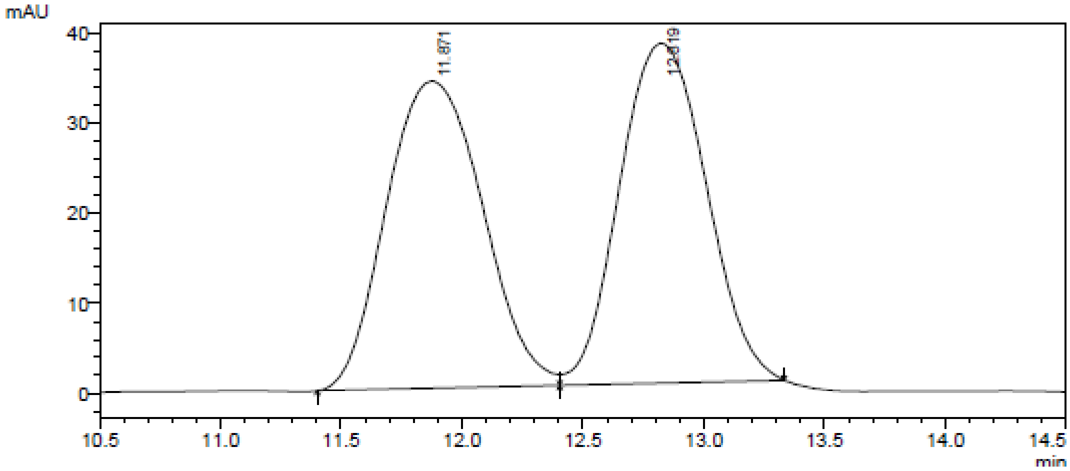

Peak#	Ret. Time	Area	Height	Area %	Height %
1	11.871	944849	33988	50.135	47.444
2	12.819	939762	37651	49.865	52.556
Total		1884611	71640	100.000	100.000

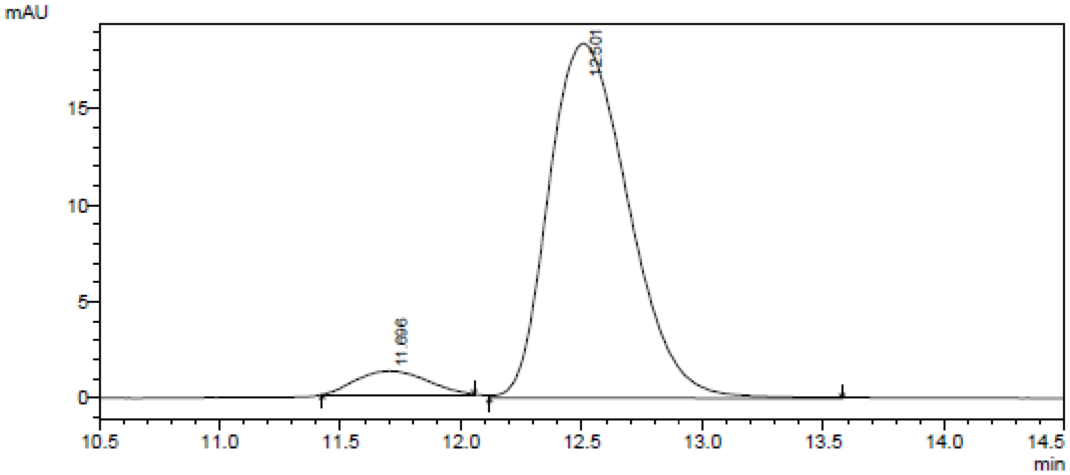

Peak#	Ret. Time	Area	Height	Area %	Height %
1	11.696	24709	1210	5.540	6.191
2	12.501	421282	18337	94.460	93.809
Total		445991	19547	100.000	100.000

Supplementary Figure 162 HPLC Spectra of **37**

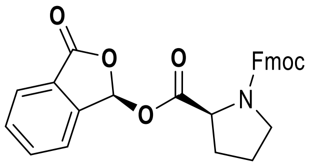

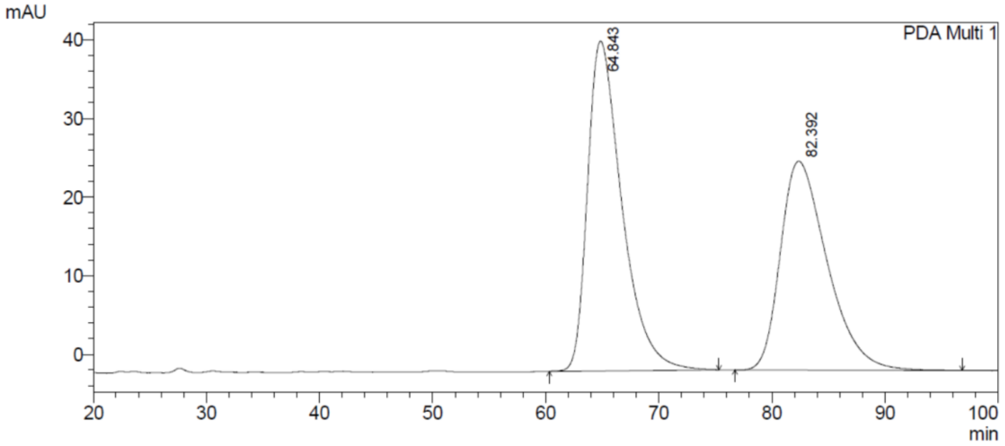

PDA Ch1 254nm 4nm

Peak#	Ret. Time	Area	Height	Area %	Height %
1	64.843	8875148	41948	53.664	61.204
2	82.392	7663170	26590	46.336	38.796
Total		16538318	68538	100.000	100.000

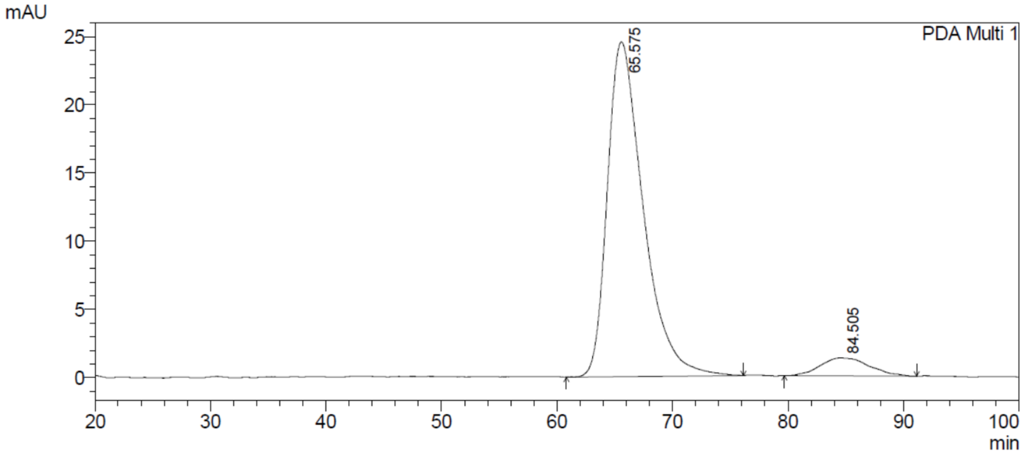

PDA Ch1 254nm 4nm

Peak#	Ret. Time	Area	Height	Area %	Height %
1	65.575	5303976	24538	93.114	94.858
2	84.505	392216	1330	6.886	5.142
Total		5696192	25868	100.000	100.000

Supplementary Figure 163 HPLC Spectra of **38**

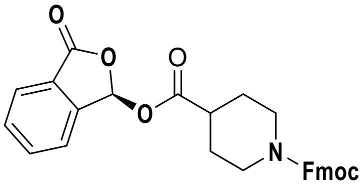

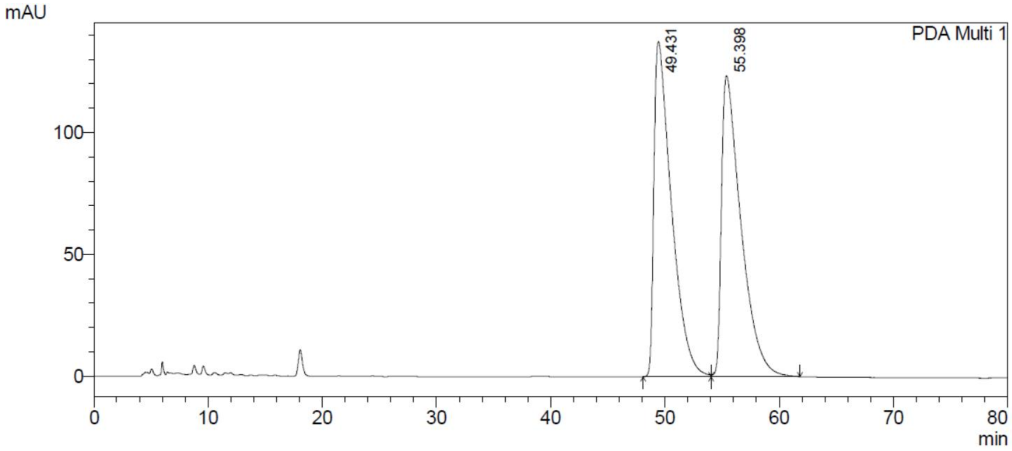

PDA Ch1 254nm 4nm

Peak#	Ret. Time	Area	Height	Area %	Height %
1	49.431	14702223	137479	50.000	52.676
2	55.398	14702501	123508	50.000	47.324
Total		29404723	260987	100.000	100.000

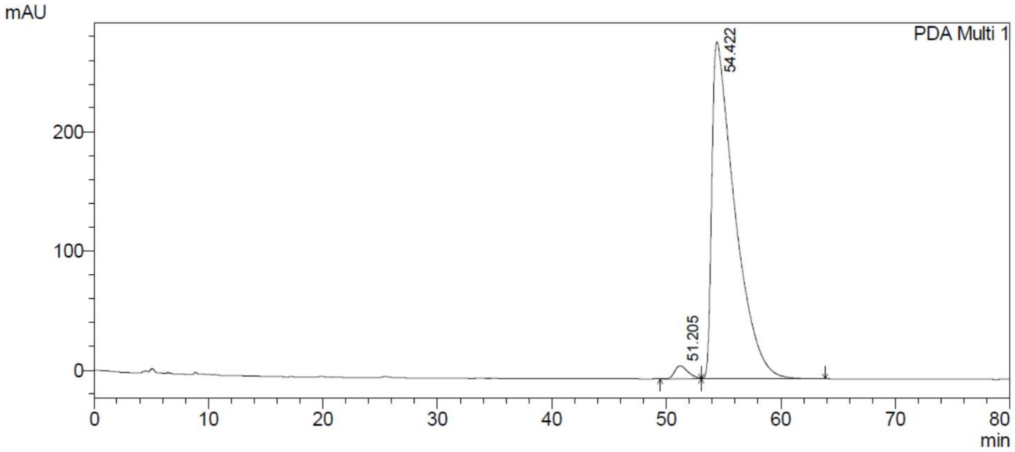

PDA Ch1 254nm 4nm

Peak#	Ret. Time	Area	Height	Area %	Height %
1	51.205	911249	10976	2.252	3.734
2	54.422	39552018	282978	97.748	96.266
Total		40463267	293953	100.000	100.000

Supplementary Figure 164 HPLC Spectra of **39**

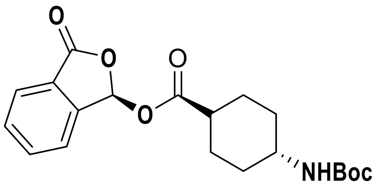

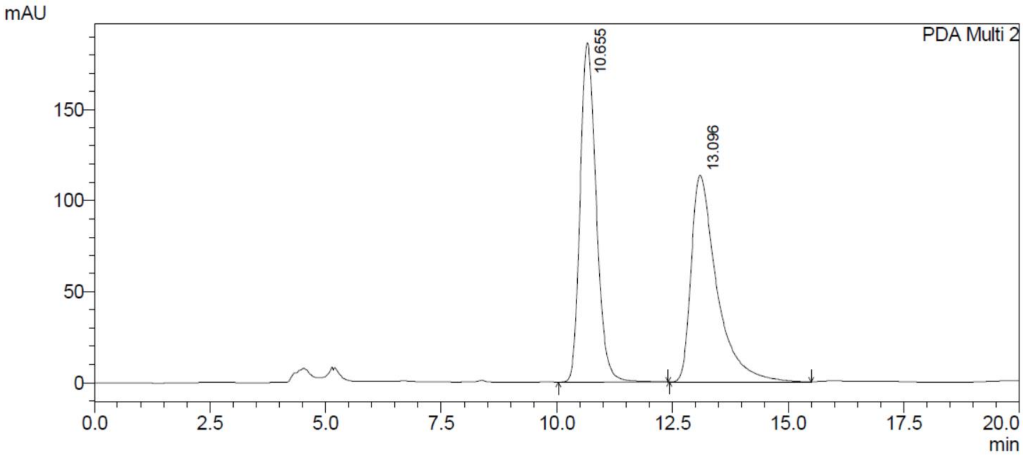

PDA Ch2 220nm 4nm

Peak#	Ret. Time	Area	Height	Area %	Height %
1	10.655	4500857	186250	50.638	62.103
2	13.096	4387401	113655	49.362	37.897
Total		8888258	299905	100.000	100.000

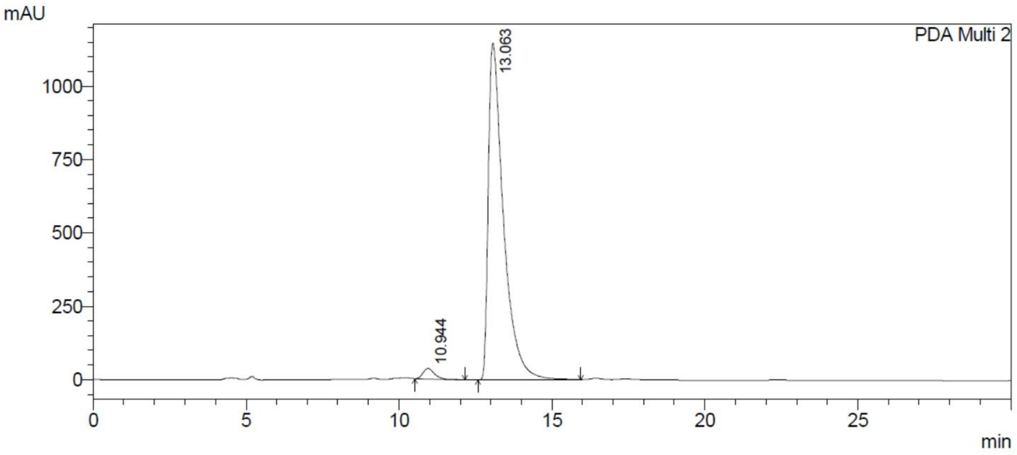

PDA Ch2 220nm 4nm

Peak#	Ret. Time	Area	Height	Area %	Height %
1	10.944	937263	35802	2.311	3.023
2	13.063	39610733	1148348	97.689	96.977
Total		40547996	1184151	100.000	100.000

Supplementary Figure 165 HPLC Spectra of **40**

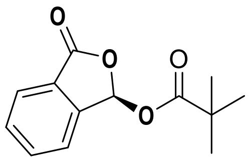

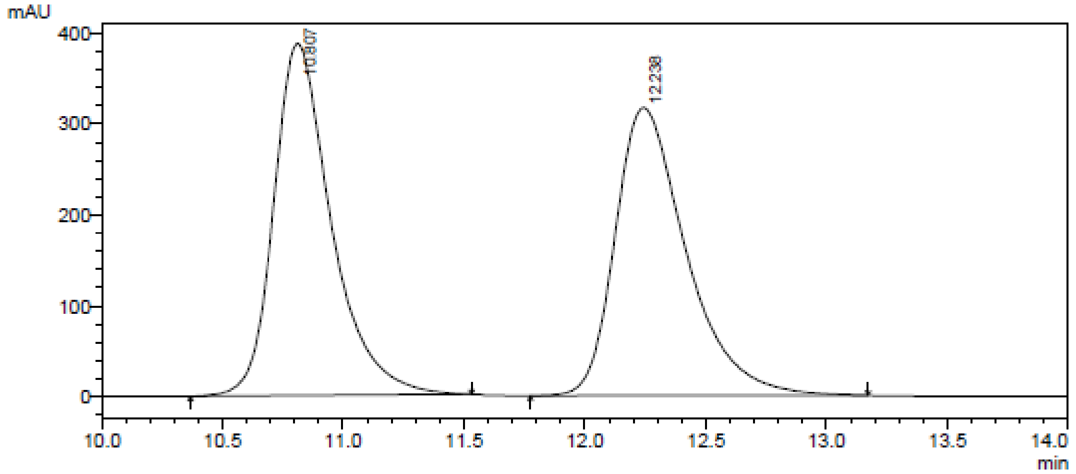

Peak#	Ret. Time	Area	Height	Area %	Height %
1	10.807	6464112	387316	49.888	55.022
2	12.238	6493100	316615	50.112	44.978
Total		12957212	703931	100.000	100.000

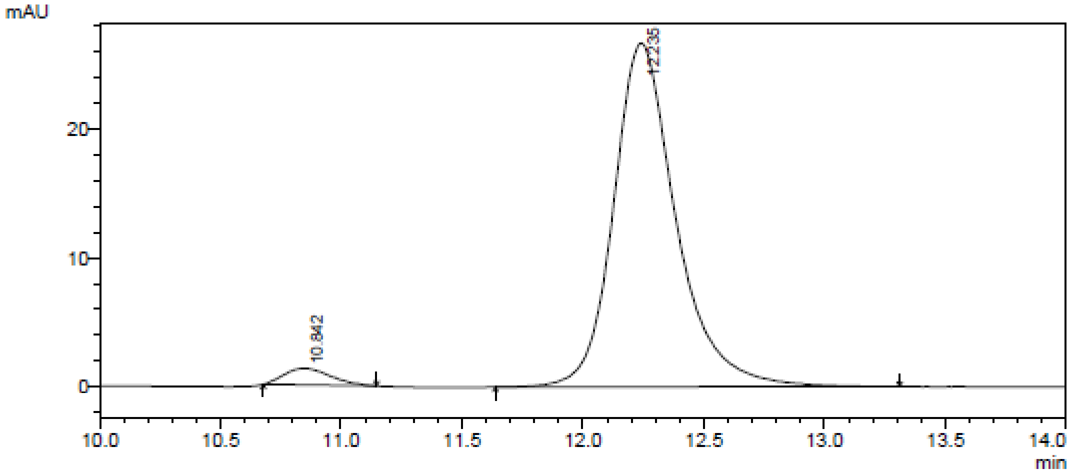

Peak#	Ret. Time	Area	Height	Area %	Height %
1	10.842	16834	1281	3.384	4.588
2	12.235	480592	26635	96.616	95.412
Total		497427	27916	100.000	100.000

Supplementary Figure 166 HPLC Spectra of **41**

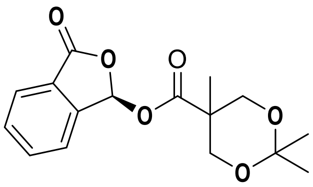

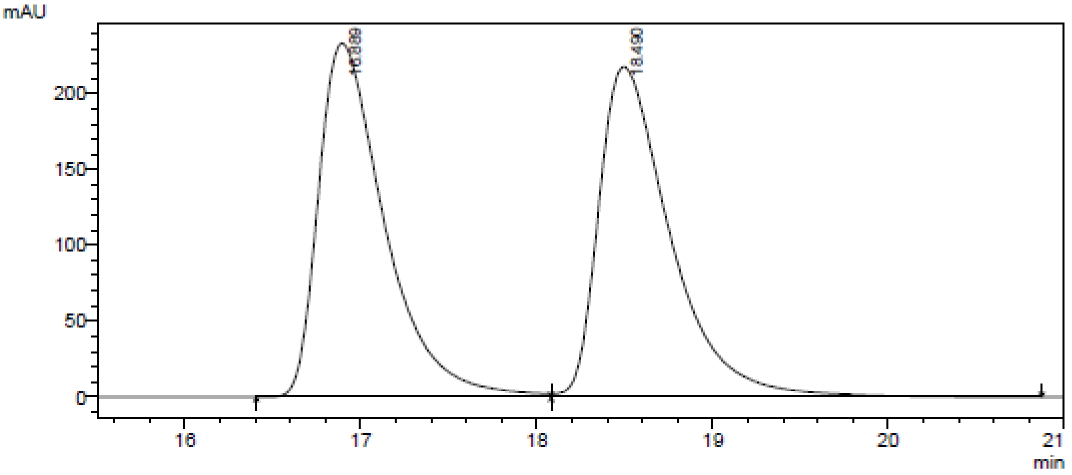

Peak#	Ret. Time	Area	Height	Area %	Height %
1	16.889	6139574	233643	49.712	51.712
2	18.490	6210791	218175	50.288	48.288
Total		12350365	451818	100.000	100.000

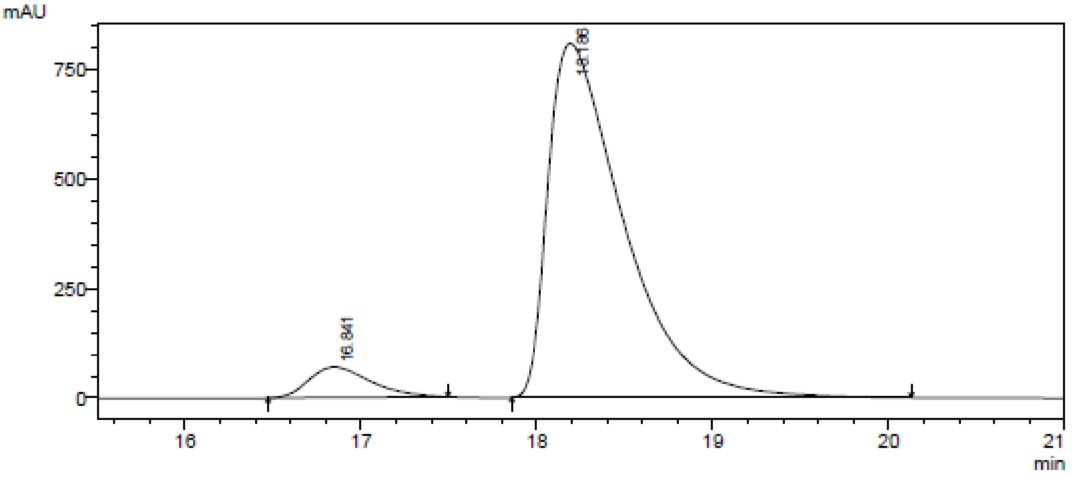

Peak#	Ret. Time	Area	Height	Area %	Height %
1	16.841	1690540	69460	6.509	7.905
2	18.186	24282869	809220	93.491	92.095
Total		25973409	878681	100.000	100.000

Supplementary Figure 167 HPLC Spectra of **42**

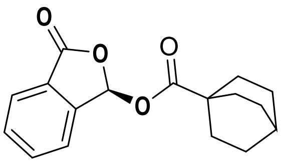

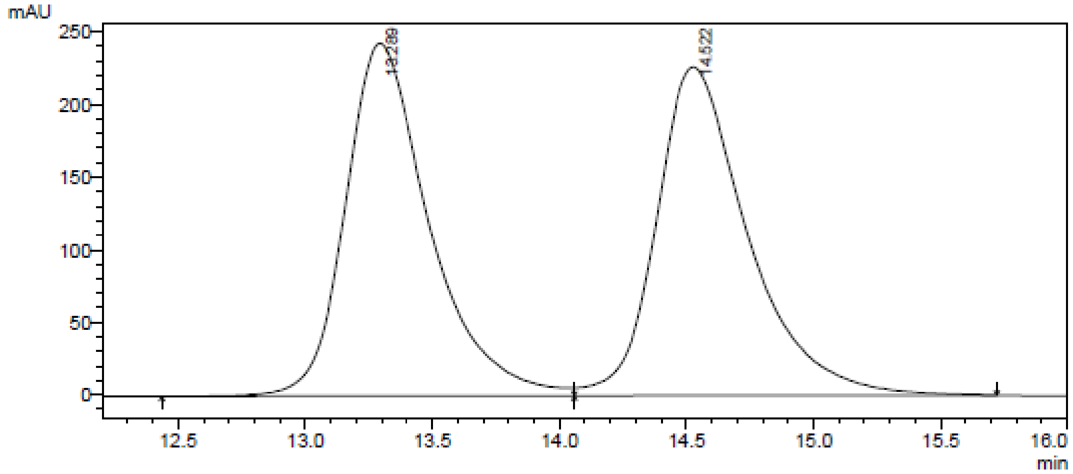

Peak#	Ret. Time	Area	Height	Area %	Height %
1	13.289	264984	11488	49.655	51.805
2	14.522	268662	10688	50.345	48.195
Total		533646	22176	100.000	100.000

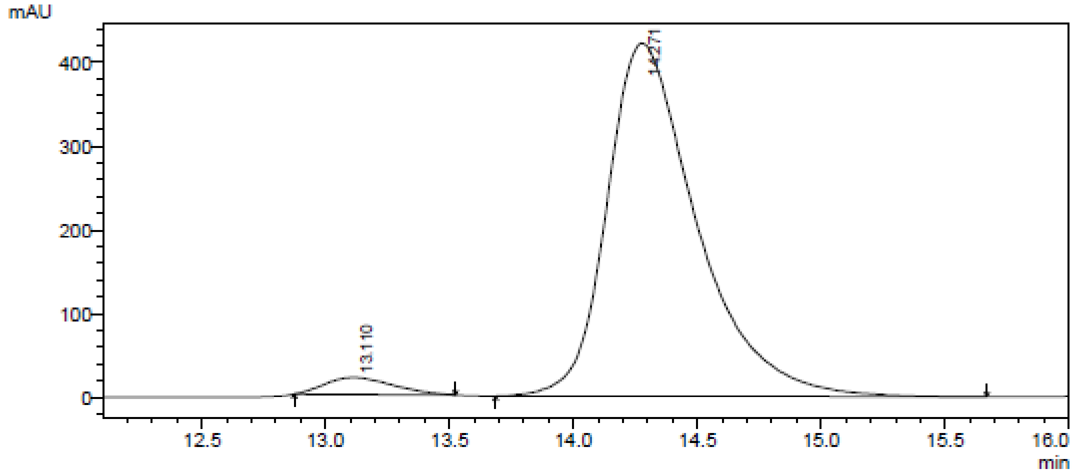

Peak#	Ret. Time	Area	Height	Area %	Height %
1	13.110	387511	20080	3.518	4.557
2	14.271	10628389	420611	96.482	95.443
Total		11015901	440691	100.000	100.000

Supplementary Figure 168 HPLC Spectra of **43**

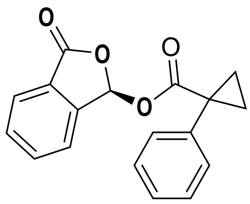

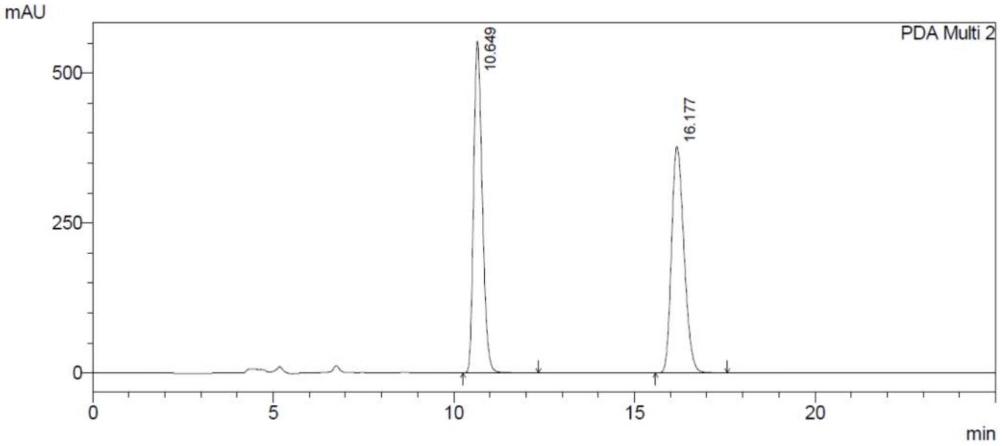

PDA Ch2 220nm 4nm

Peak#	Ret. Time	Area	Height	Area %	Height %
1	10.649	9209217	552792	50.310	59.403
2	16.177	9095789	377793	49.690	40.597
Total		18305007	930585	100.000	100.000

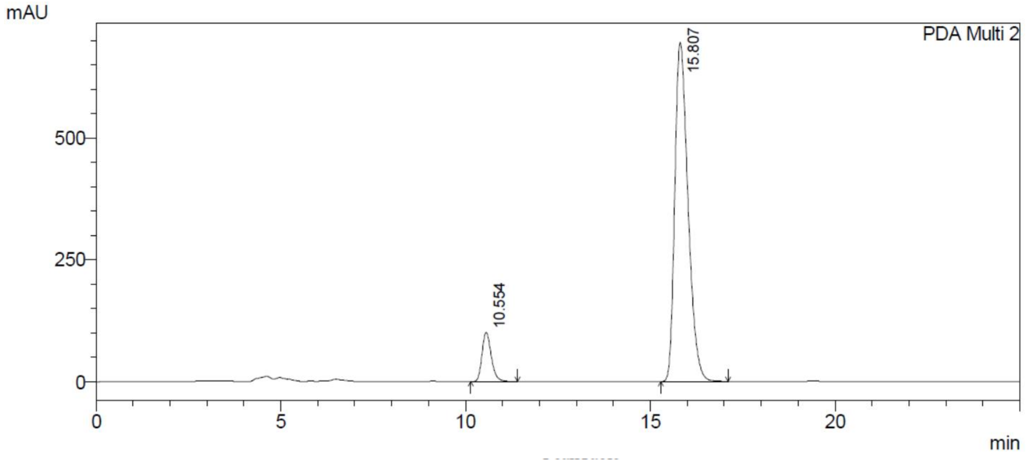

PDA Ch2 220nm 4nm

Peak#	Ret. Time	Area	Height	Area %	Height %
1	10.554	1755374	100561	9.222	12.634
2	15.807	17279915	695407	90.778	87.366
Total		19035289	795969	100.000	100.000

Supplementary Figure 169 HPLC Spectra of **44**

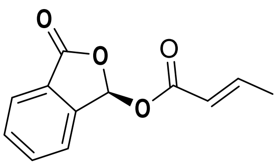

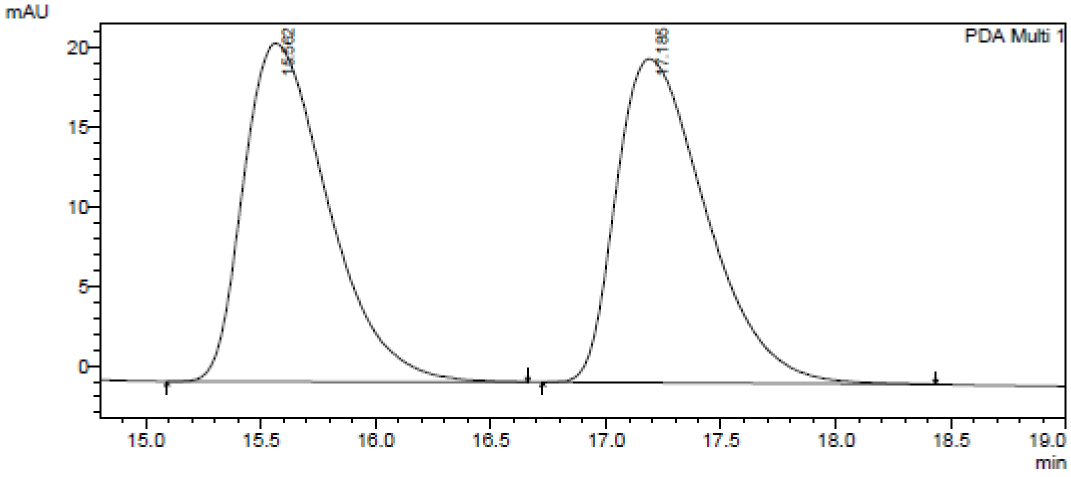

Peak#	Ret. Time	Area	Height	Area %	Height %
1	15.562	546228	21258	49.696	51.070
2	17.185	552903	20367	50.304	48.930
Total		1099131	41625	100.000	100.000

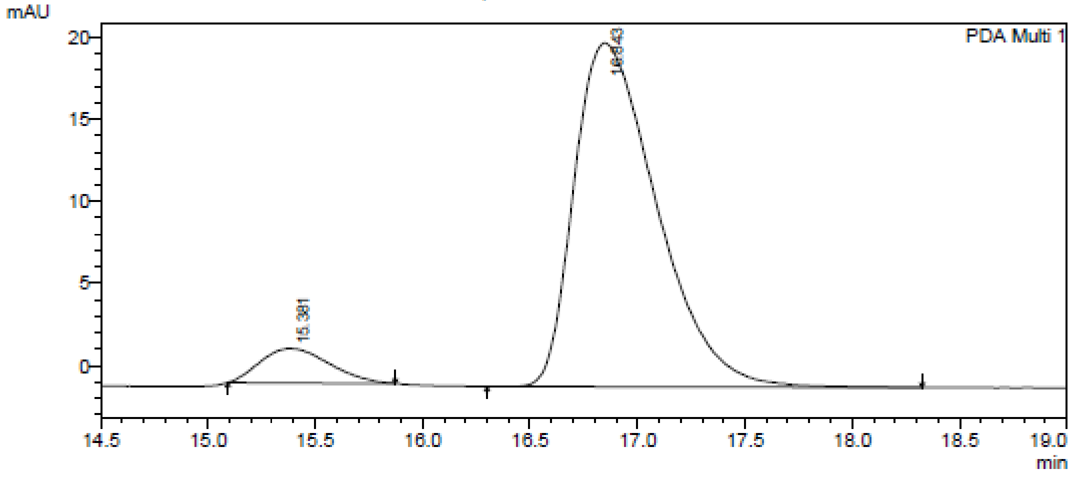

Peak#	Ret. Time	Area	Height	Area %	Height %
1	15.381	50381	2173	8.271	9.420
2	16.843	558780	20896	91.729	90.580
Total		609162	23069	100.000	100.000

Supplementary Figure 170 HPLC Spectra of **45**

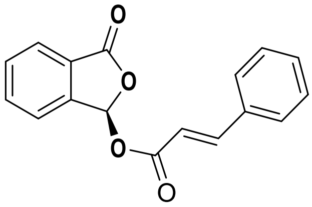

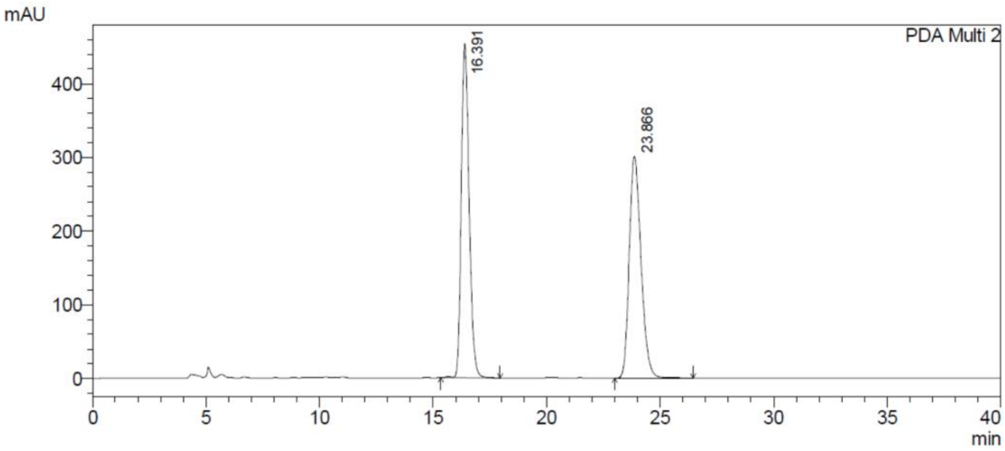

PDA Ch2 220nm 4nm

Peak#	Ret. Time	Area	Height	Area %	Height %
1	16.391	10893458	453500	50.036	60.108
2	23.866	10877794	300979	49.964	39.892
Total		21771252	754479	100.000	100.000

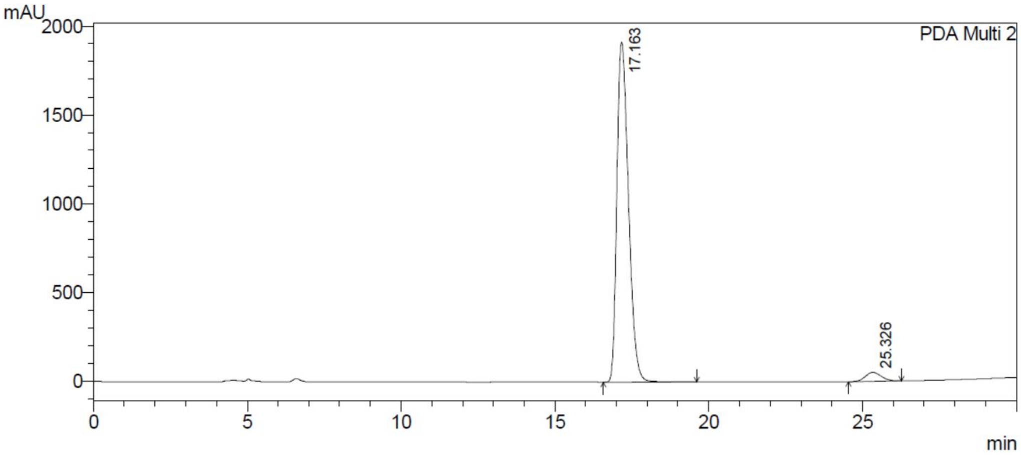

PDA Ch2 220nm 4nm

Peak#	Ret. Time	Area	Height	Area %	Height %
1	17.163	49845791	1913065	96.452	97.427
2	25.326	1833622	50515	3.548	2.573
Total		51679414	1963580	100.000	100.000

Supplementary Figure 171 HPLC Spectra of **46**

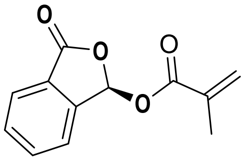

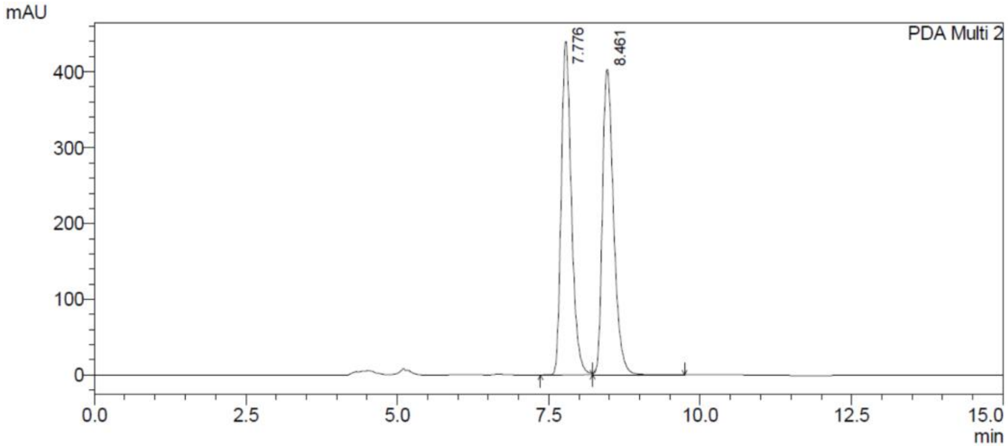

PDA Ch2 220nm 4nm

Peak#	Ret. Time	Area	Height	Area %	Height %
1	7.776	5157374	439696	49.887	52.166
2	8.461	5180652	403186	50.113	47.834
Total		10338027	842882	100.000	100.000

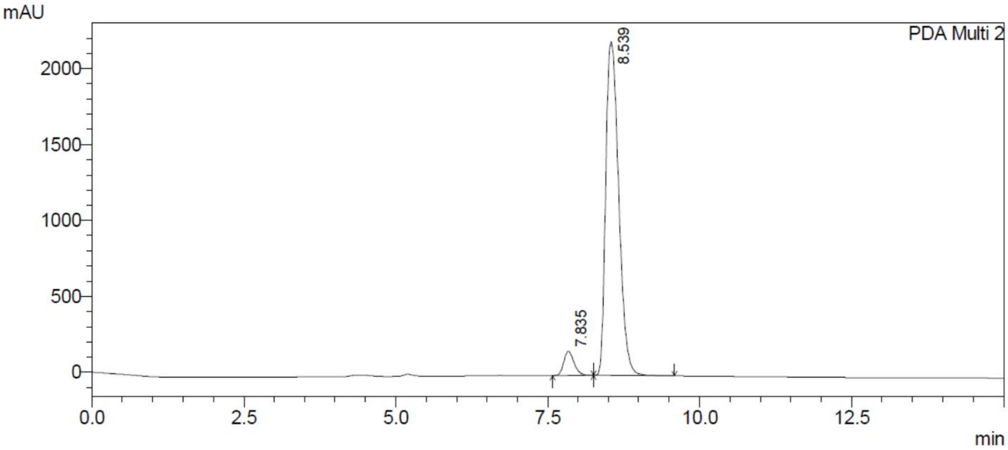

PDA Ch2 220nm 4nm

Peak#	Ret. Time	Area	Height	Area %	Height %
1	7.835	1873026	159136	5.427	6.753
2	8.539	32641212	2197486	94.573	93.247
Total		34514238	2356622	100.000	100.000

Supplementary Figure 172 HPLC Spectra of **47**

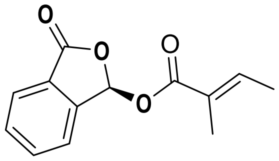

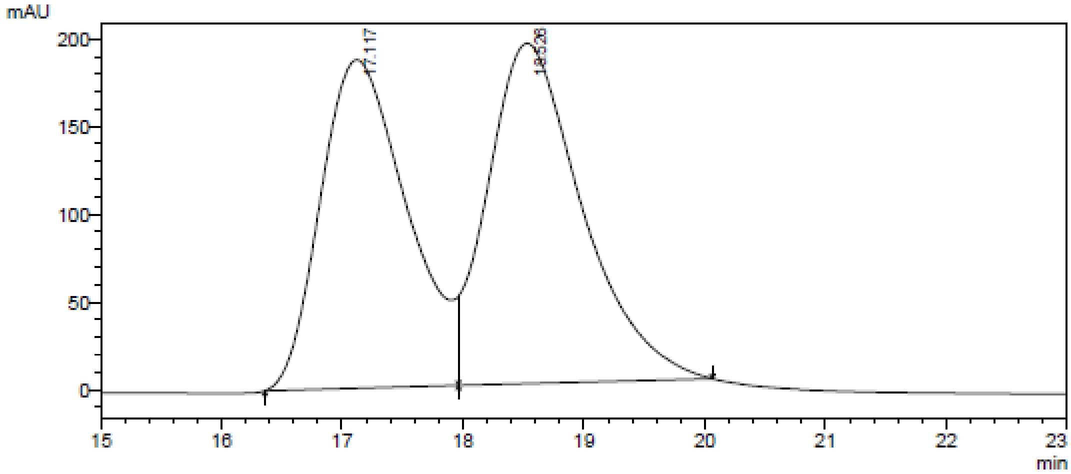

Peak#	Ret. Time	Area	Height	Area %	Height %
1	17.117	9362208	187362	47.054	49.116
2	18.526	10534444	194106	52.946	50.884
Total		19896652	381468	100.000	100.000

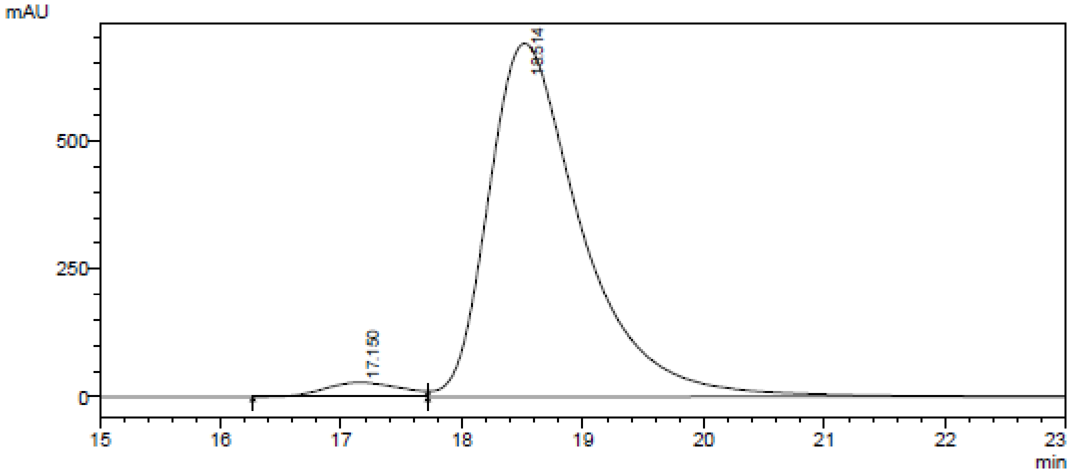

Peak#	Ret. Time	Area	Height	Area %	Height %
1	17.150	1201795	27537	3.147	3.838
2	18.514	36990885	689966	96.853	96.162
Total		38192680	717503	100.000	100.000

Supplementary Figure 173 HPLC Spectra of **48**

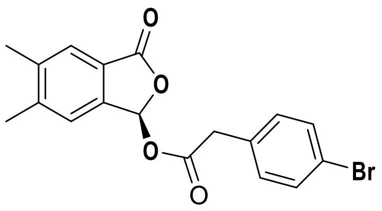

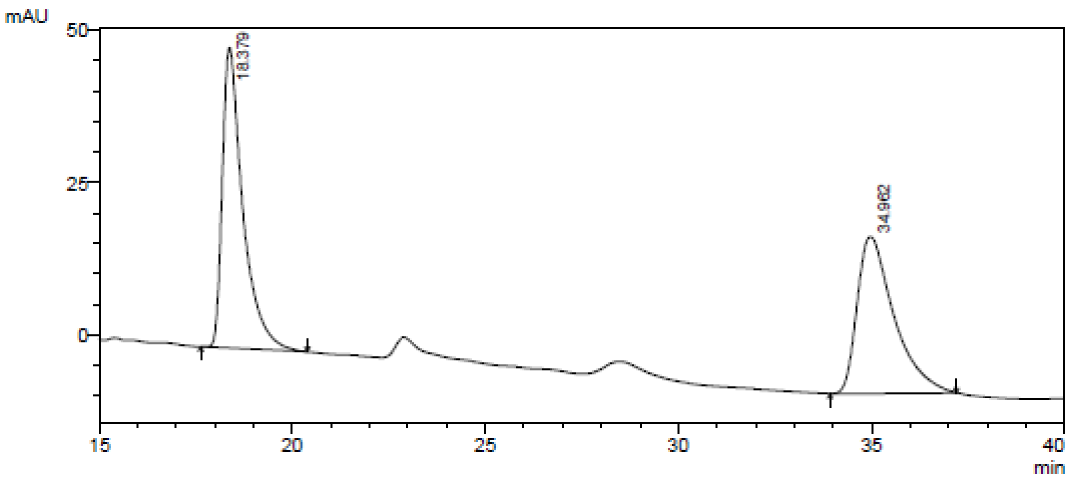

PDA Ch2 220nm 4nm

Peak#	Ret. Time	Area	Height	Area %	Height %
1	18.379	1815382	49262	51.698	65.649
2	34.962	1696128	25777	48.302	34.351
Total		3511509	75039	100.000	100.000

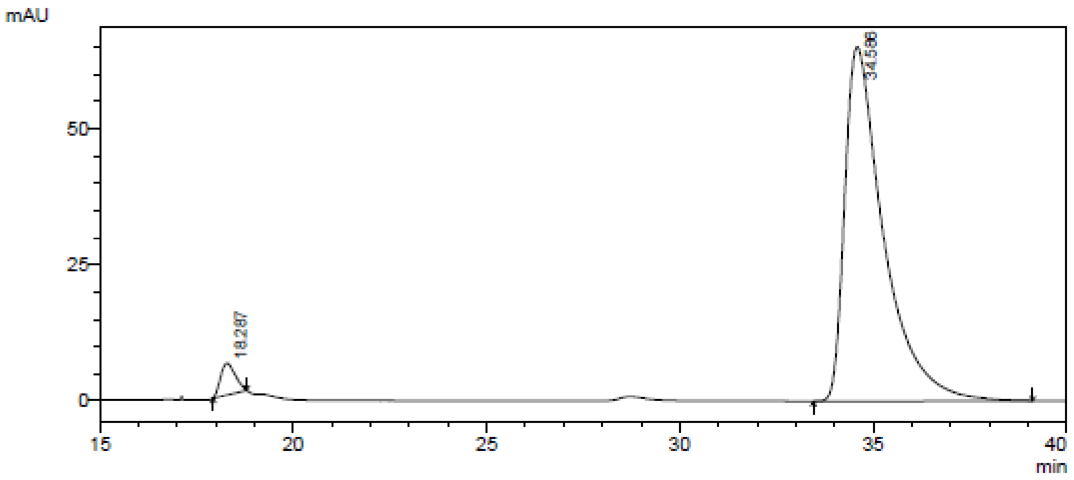

PDA Ch2 220nm 4nm

Peak#	Ret. Time	Area	Height	Area %	Height %
1	18.287	154752	5818	3.289	8.205
2	34.586	4550277	65091	96.711	91.795
Total		4705029	70909	100.000	100.000

Supplementary Figure 174 HPLC Spectra of **49**

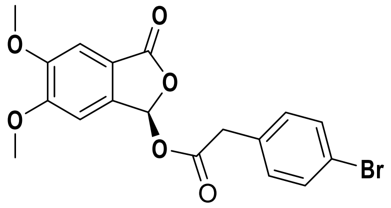

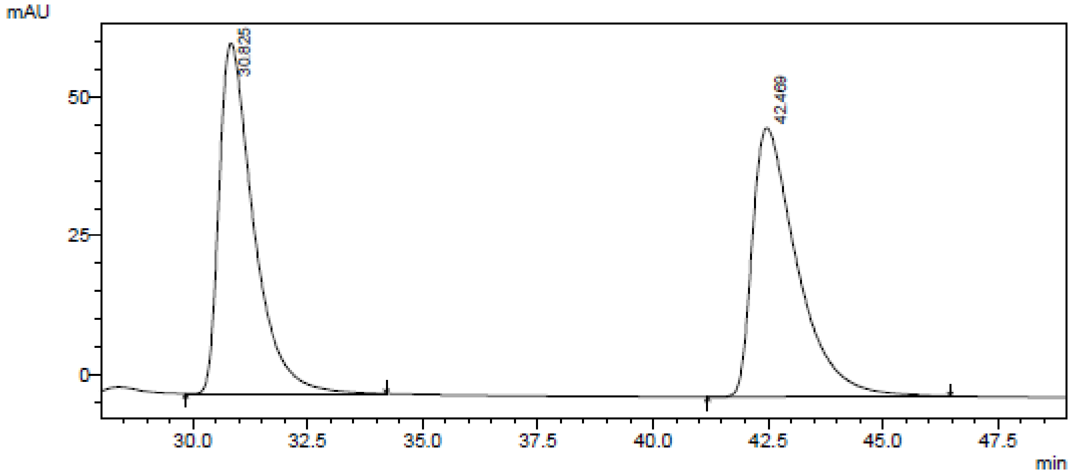

PDA Ch2 220nm 4nm

Peak#	Ret. Time	Area	Height	Area %	Height %
1	30.825	3304541	63176	49.981	56.605
2	42.469	3307037	48432	50.019	43.395
Total		6611578	111608	100.000	100.000

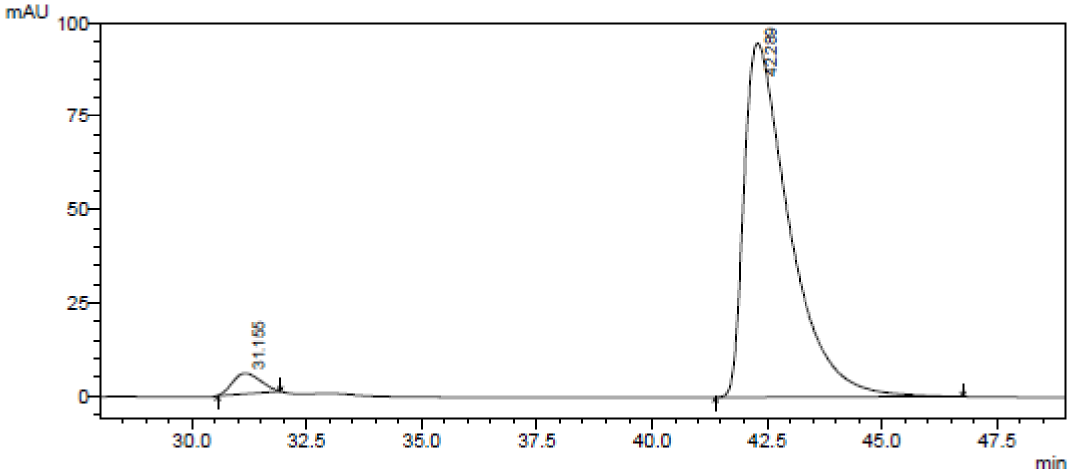

PDA Ch2 220nm 4nm

Peak#	Ret. Time	Area	Height	Area %	Height %
1	31.155	232818	5510	3.444	5.480
2	42.289	6528076	95048	96.556	94.520
Total		6760894	100559	100.000	100.000

Supplementary Figure 175 HPLC Spectra of **50**

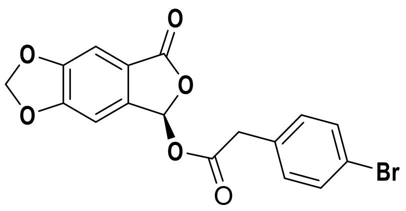

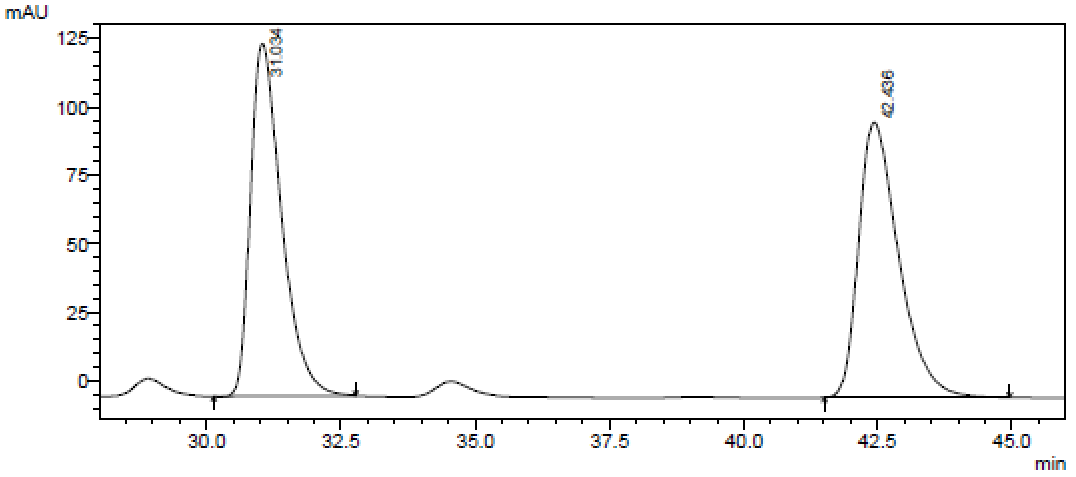

PDA Ch2 220nm 4nm

Peak#	Ret. Time	Area	Height	Area %	Height %
1	31.034	5172372	128595	50.015	56.268
2	42.436	5169209	99944	49.985	43.732
Total		10341581	228539	100.000	100.000

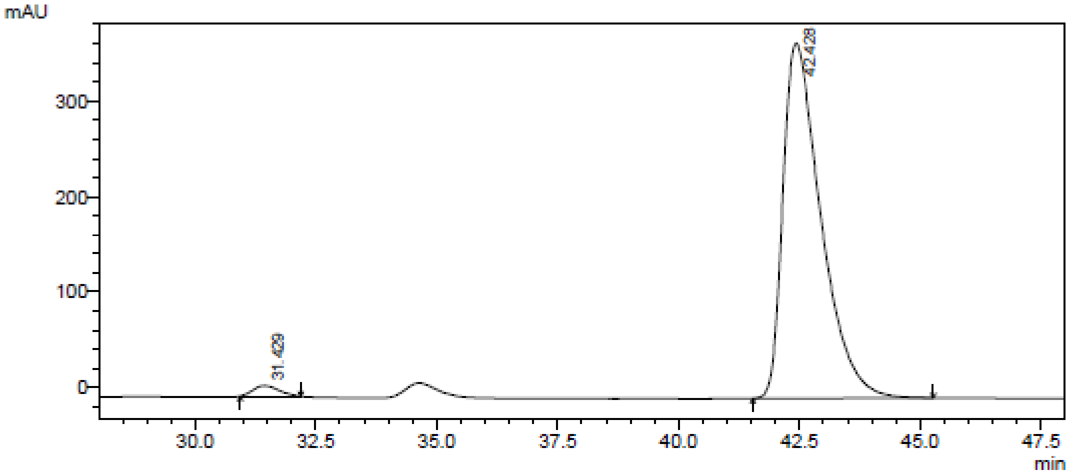

PDA Ch2 220nm 4nm

Peak#	Ret. Time	Area	Height	Area %	Height %
1	31.429	410806	11086	1.965	2.897
2	42.428	20500171	371644	98.035	97.103
Total		20910977	382730	100.000	100.000

Supplementary Figure 176 HPLC Spectra of **51**

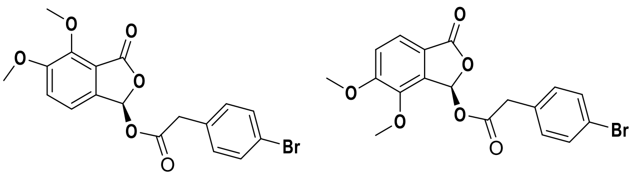

Major: Compound **52a**　　　　Minor: Compound **52b**

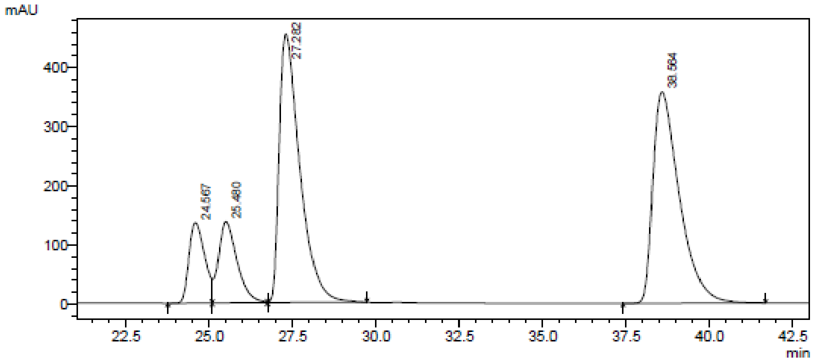

Peak#	Ret. Time	Area	Height	Area %	Height %
1	24.567	4538578	135710	9.178	12.506
2	25.480	5337274	137053	10.793	12.630
3	27.282	19749945	454693	39.940	41.901
4	38.564	19823811	357693	40.089	32.963
Total		49449608	1085150	100.000	100.000

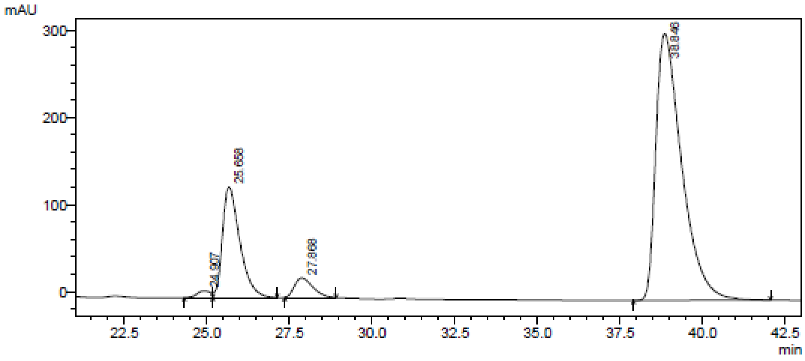

Peak#	Ret. Time	Area	Height	Area %	Height %
1	24.907	229160	7986	1.016	1.722
2	25.658	4670292	127170	20.712	27.416
3	27.868	877286	22555	3.891	4.863
4	38.846	16772135	306146	74.381	66.000
Total		22548874	463857	100.000	100.000

Racemic compound **52a**

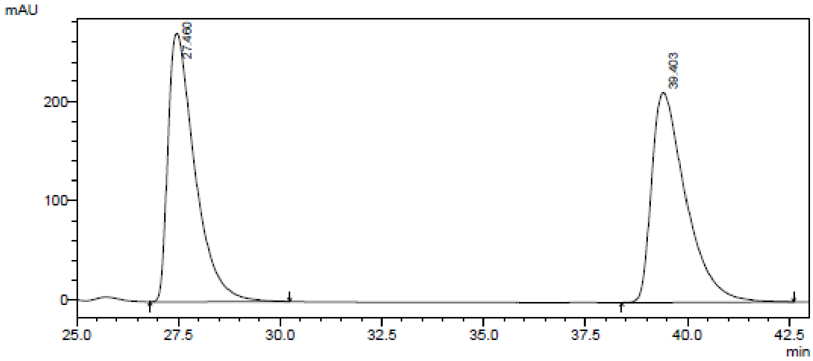

Peak#	Ret. Time	Area	Height	Area %	Height %
1	27.462	917811	19698	49.710	55.846
2	39.404	928502	15575	50.290	44.154
Total		1846313	35273	100.000	100.000

Supplementary Figure 177 HPLC Spectra of **52a and 52b**

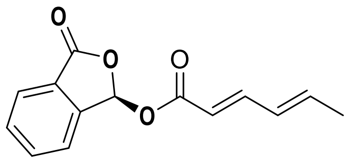

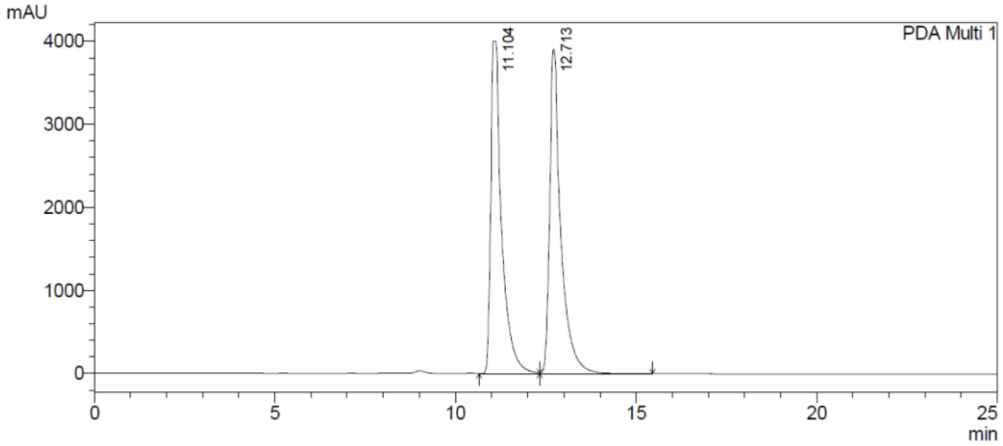

PDA Ch1 254nm 4nm

Peak#	Ret. Time	Area	Height	Area %	Height %
1	11.104	84038280	4004158	50.076	50.596
2	12.713	83784409	3909863	49.924	49.404
Total		167822689	7914022	100.000	100.000

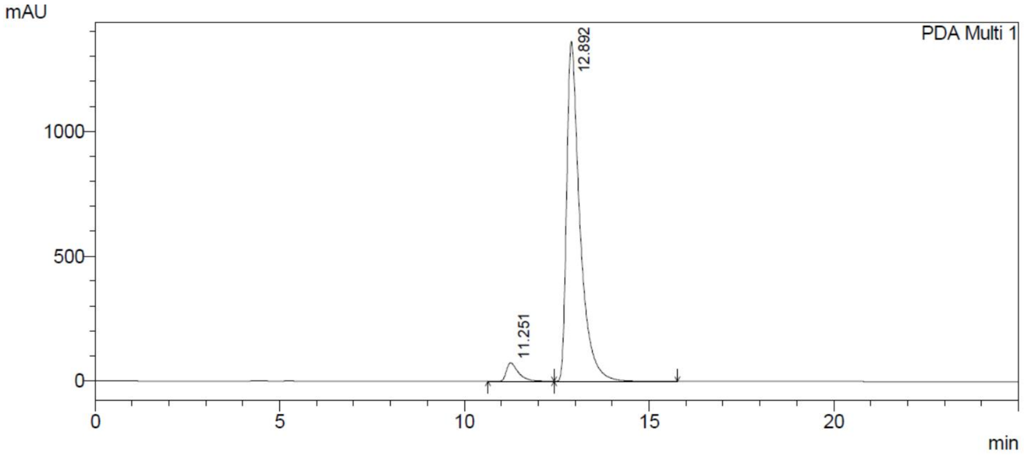

PDA Ch1 254nm 4nm

Peak#	Ret. Time	Area	Height	Area %	Height %
1	11.251	1700149	74273	4.771	5.175
2	12.892	33936779	1360857	95.229	94.825
Total		35636928	1435129	100.000	100.000

Supplementary Figure 178 HPLC Spectra of **53**

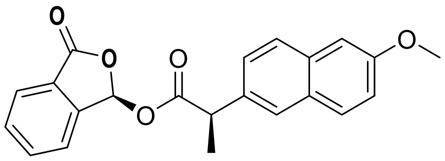

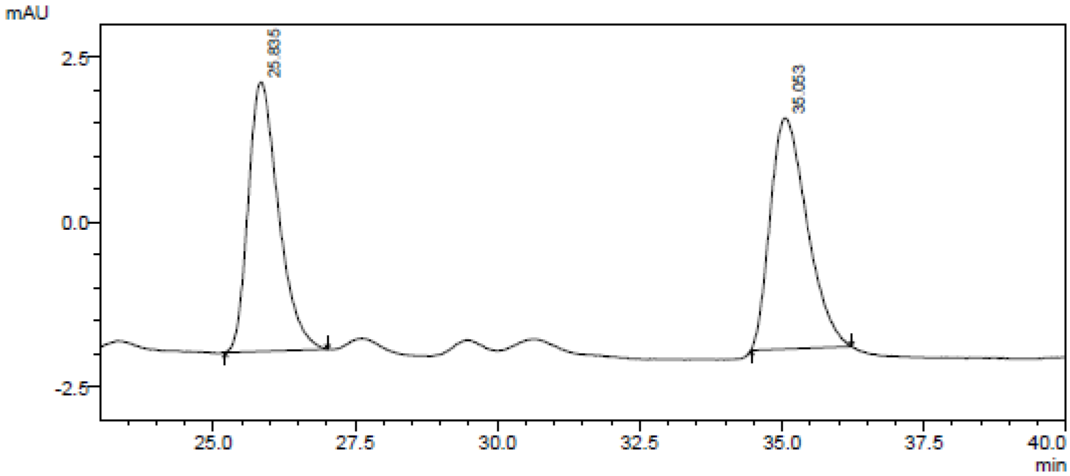

Peak#	Ret. Time	Area	Height	Area %	Height %
1	25.835	153037	4085	48.963	53.789
2	35.053	159519	3510	51.037	46.211
Total		312556	7595	100.000	100.000

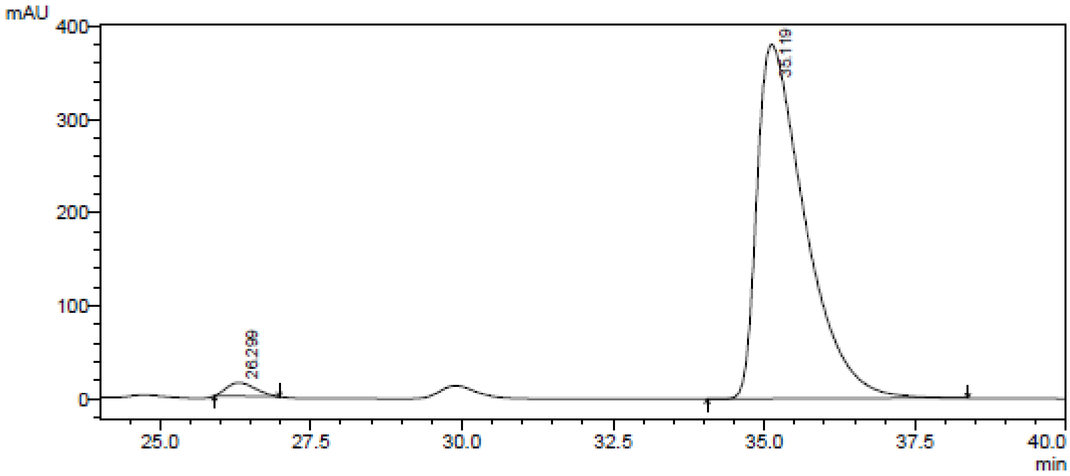

Peak#	Ret. Time	Area	Height	Area %	Height %
1	26.299	492753	14792	2.278	3.748
2	35.119	21140778	379857	97.722	96.252
Total		21633531	394649	100.000	100.000

Supplementary Figure 179 HPLC Spectra of *R*-**57**

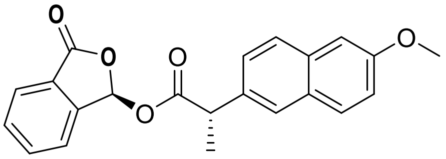

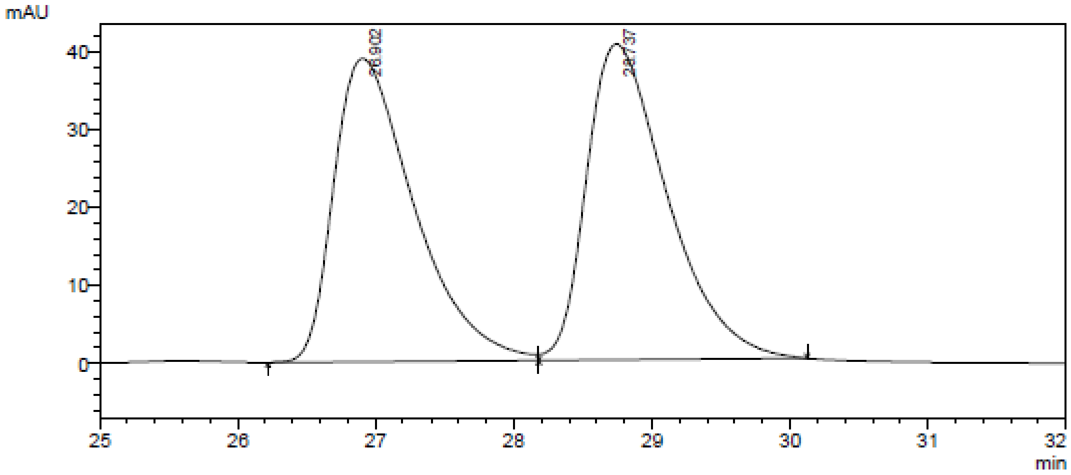

Peak#	Ret. Time	Area	Height	Area %	Height %
1	26.902	1575972	38953	48.901	48.968
2	28.737	1646829	40595	51.099	51.032
Total		3222801	79548	100.000	100.000

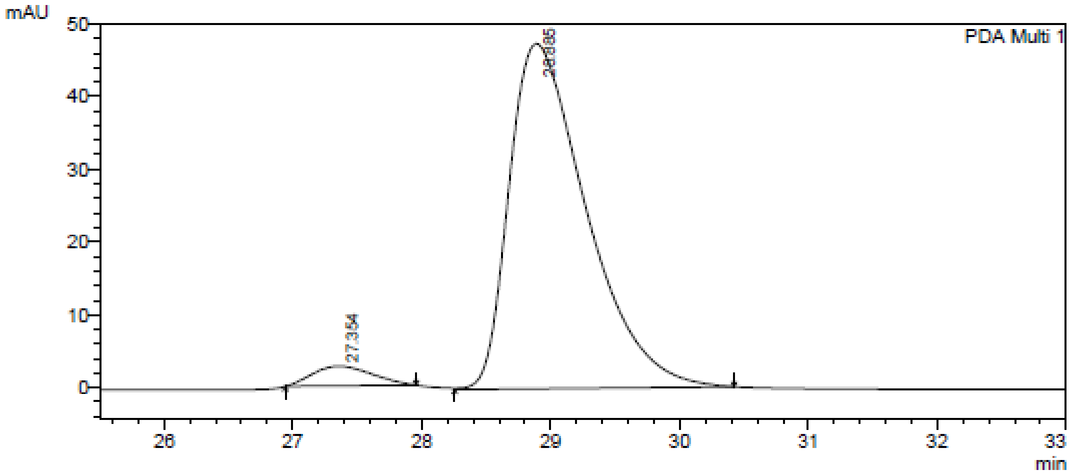

Peak#	Ret. Time	Area	Height	Area %	Height %
1	27.354	86886	2679	4.234	5.354
2	28.885	1965260	47356	95.766	94.646
Total		2052147	50035	100.000	100.000

Supplementary Figure 180 HPLC Spectra of *S*-**57**

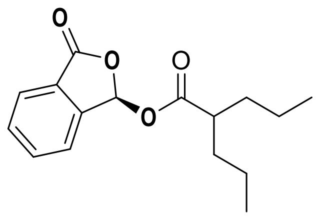

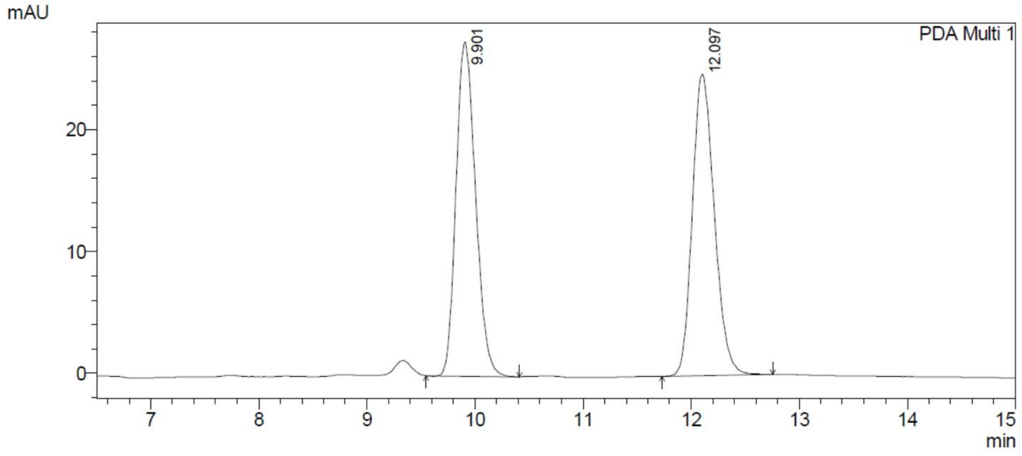

PDA Ch1 254nm 4nm

Peak#	Ret. Time	Area	Height	Area %	Height %
1	9.901	345298	27407	49.672	52.561
2	12.097	349860	24736	50.328	47.439
Total		695158	52143	100.000	100.000

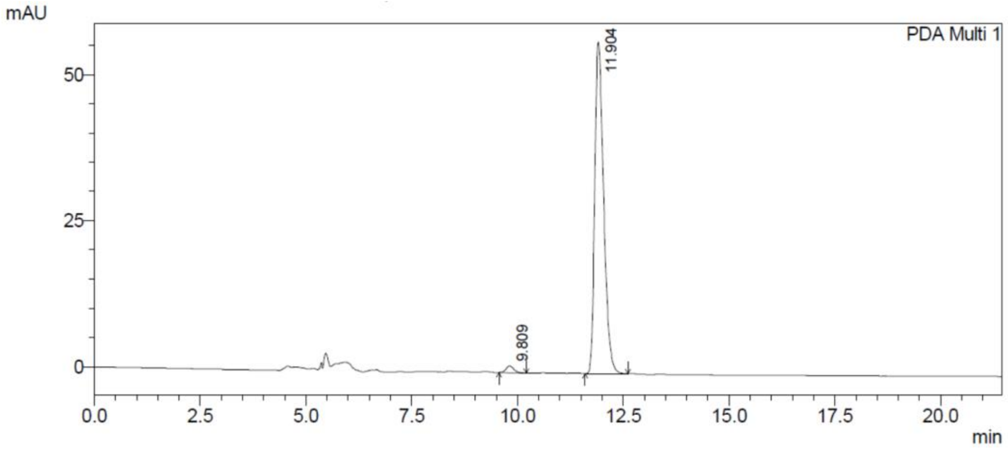

PDA Ch1 254nm 4nm

Peak#	Ret. Time	Area	Height	Area %	Height %
1	9.809	14440	1138	1.714	1.967
2	11.904	827962	56702	98.286	98.033
Total		842402	57840	100.000	100.000

Supplementary Figure 181 HPLC Spectra of **58**

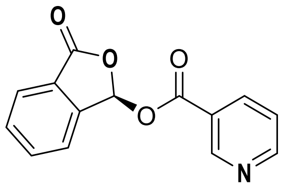

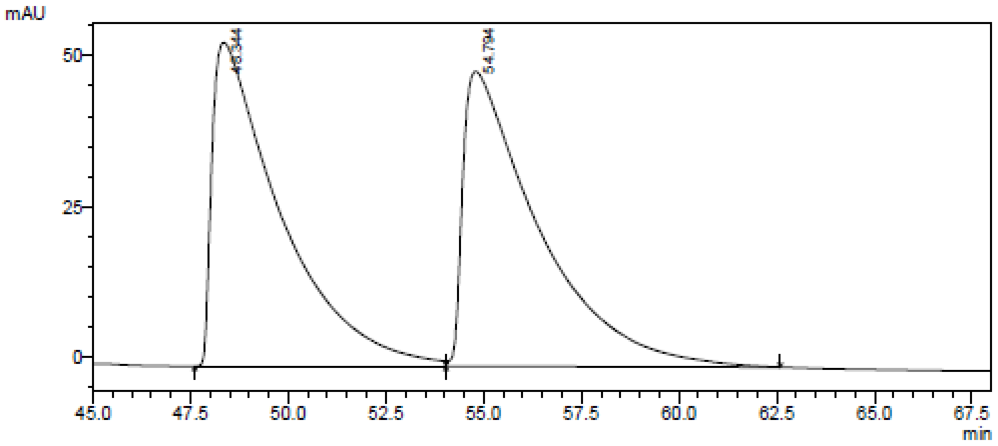

Peak#	Ret. Time	Area	Height	Area %	Height %
1	48.344	6513528	53635	49.785	52.303
2	54.794	6569718	48911	50.215	47.697
Total		13083246	102546	100.000	100.000

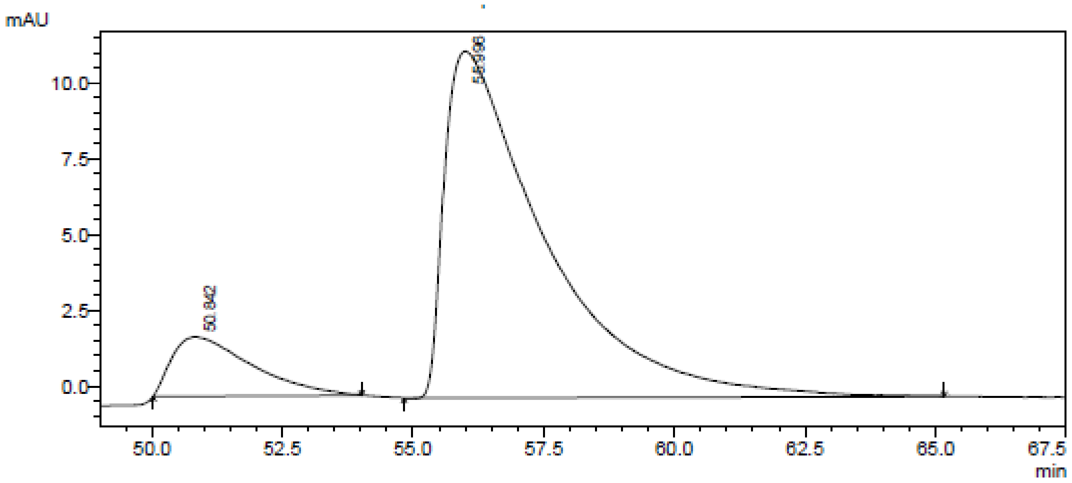

Peak#	Ret. Time	Area	Height	Area %	Height %
1	50.842	206918	1952	11.948	14.571
2	55.996	1524852	11442	88.052	85.429
Total		1731770	13394	100.000	100.000

Supplementary Figure 182 HPLC Spectra of **59**

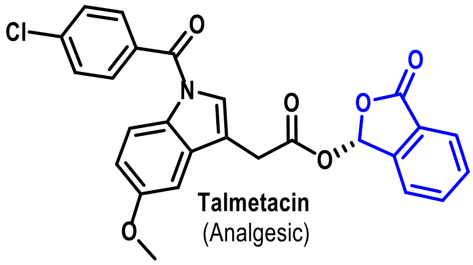

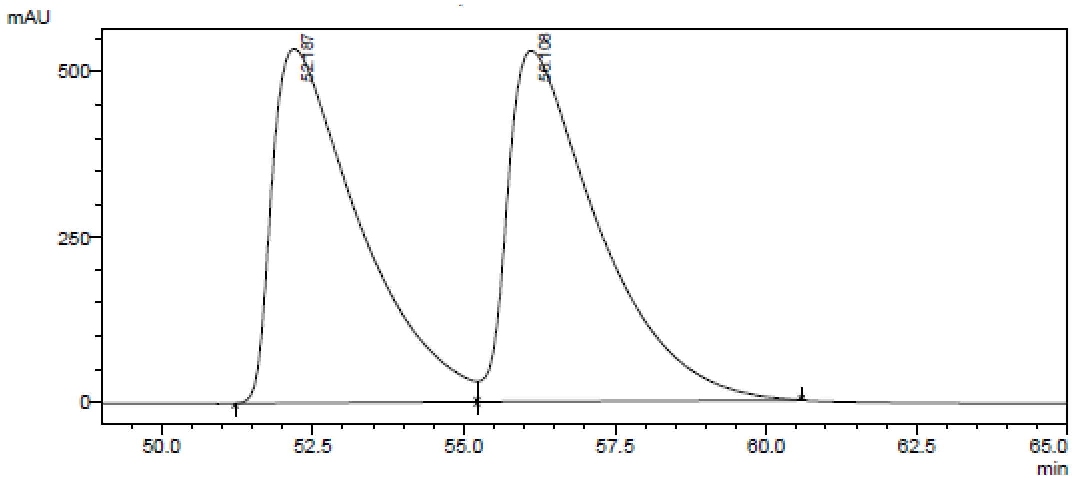

Peak#	Ret. Time	Area	Height	Area %	Height %
1	52.187	54208328	536643	48.741	50.131
2	56.108	57007906	533832	51.259	49.869
Total		111216233	1070475	100.000	100.000

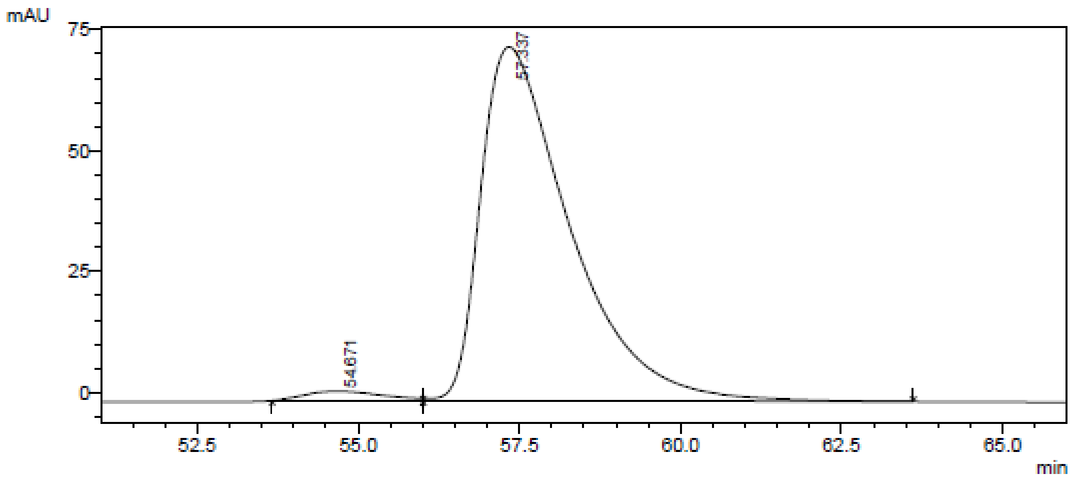

Peak#	Ret. Time	Area	Height	Area %	Height %
1	54.671	161874	1929	2.243	2.567
2	57.337	7055090	73203	97.757	97.433
Total		7216964	75132	100.000	100.000

Supplementary Figure 183 HPLC Spectra of **60**

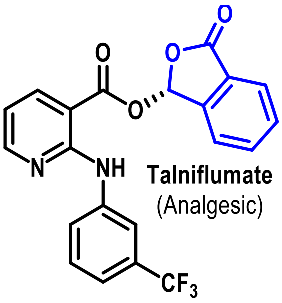

Talniflumate
(Analgesic)

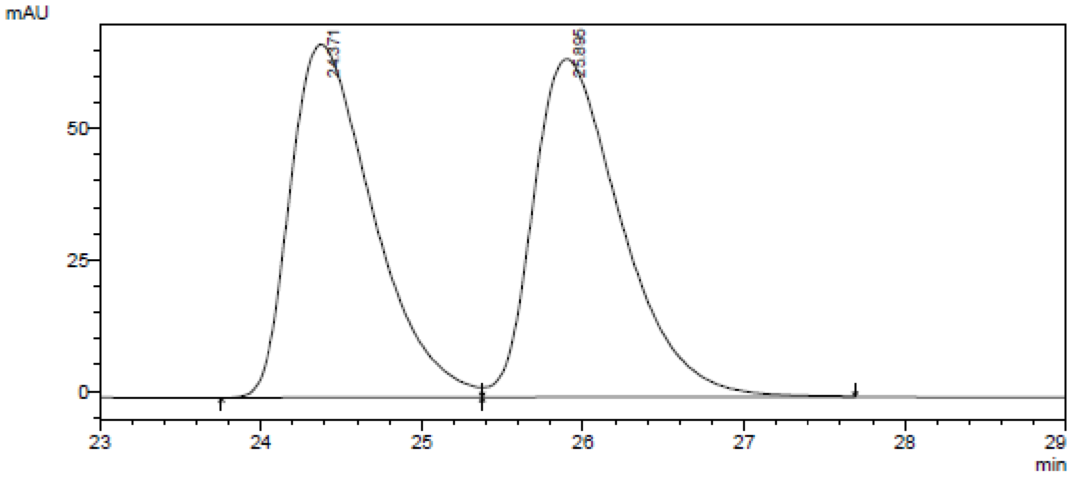

Peak#	Ret. Time	Area	Height	Area %	Height %
1	24.371	2405152	67224	49.565	51.083
2	25.895	2447398	64374	50.435	48.917
Total		4852550	131598	100.000	100.000

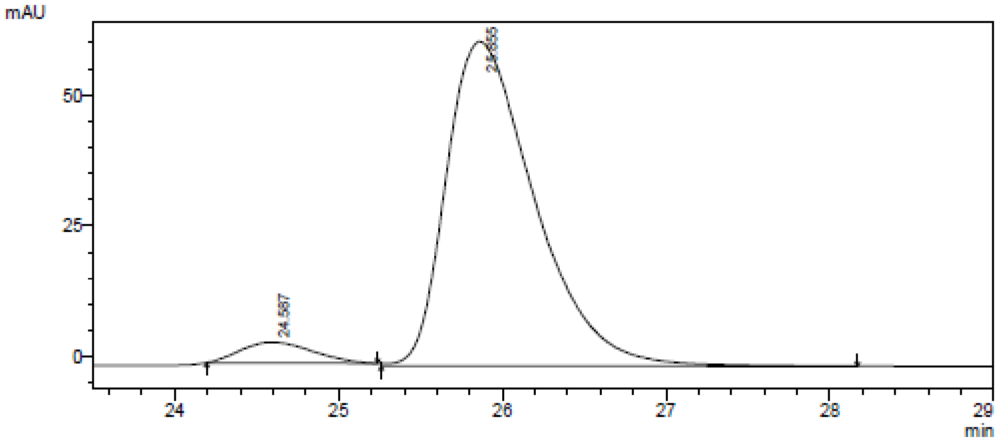

Peak#	Ret. Time	Area	Height	Area %	Height %
1	24.587	121095	3943	4.922	5.972
2	25.855	2339264	62081	95.078	94.028
Total		2460359	66024	100.000	100.000

Supplementary Figure 184 HPLC Spectra of **61**

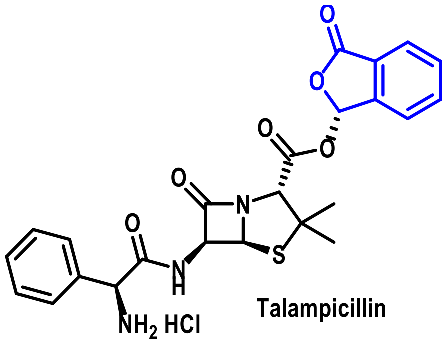

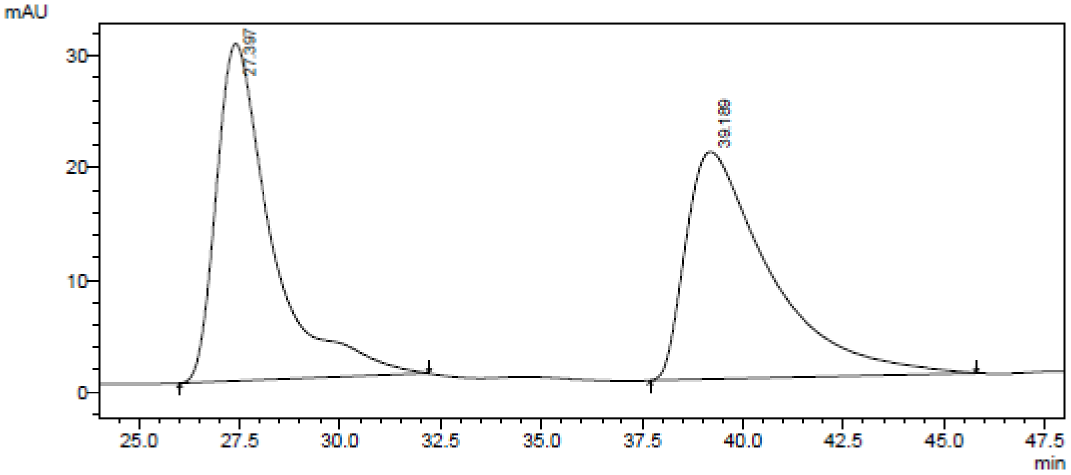

Peak#	Ret. Time	Area	Height	Area %	Height %
1	27.397	2886199	30113	49.495	59.840
2	39.189	2945151	20209	50.505	40.160
Total		5831350	50322	100.000	100.000

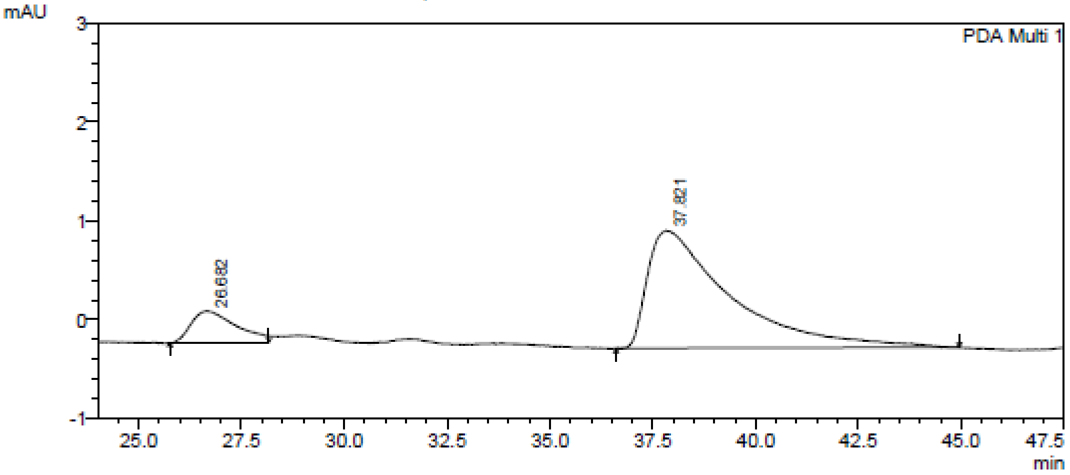

Peak#	Ret. Time	Area	Height	Area %	Height %
1	26.682	23541	314	12.510	20.839
2	37.821	164637	1192	87.490	79.161
Total		188178	1506	100.000	100.000

Supplementary Figure 185 HPLC Spectra of **62**

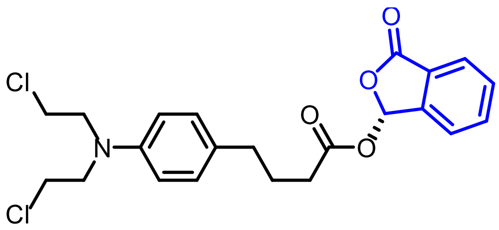

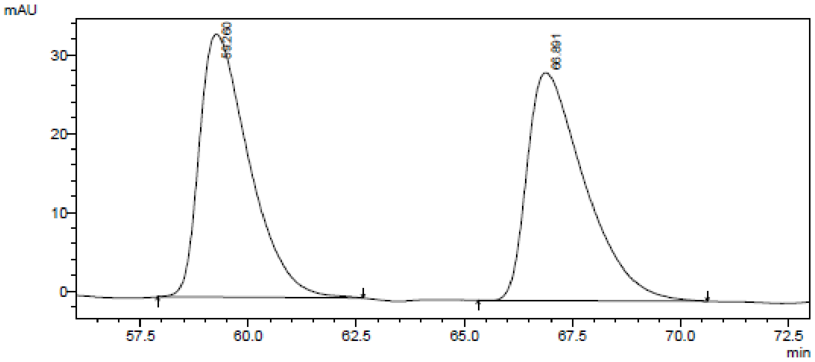

Peak#	Ret. Time	Area	Height	Area %	Height %
1	59.260	3724292	46351	50.105	53.685
2	66.880	3708746	39987	49.895	46.315
Total		7433038	86338	100.000	100.000

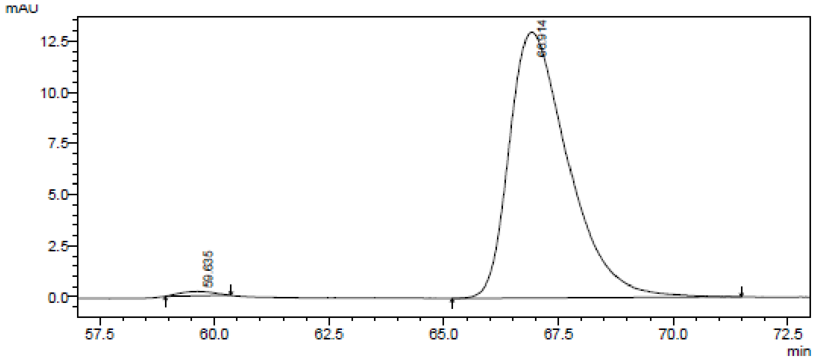

Peak#	Ret. Time	Area	Height	Area %	Height %
1	59.635	11221	226	0.957	1.705
2	66.914	1160863	13001	99.043	98.295
Total		1172084	13227	100.000	100.000

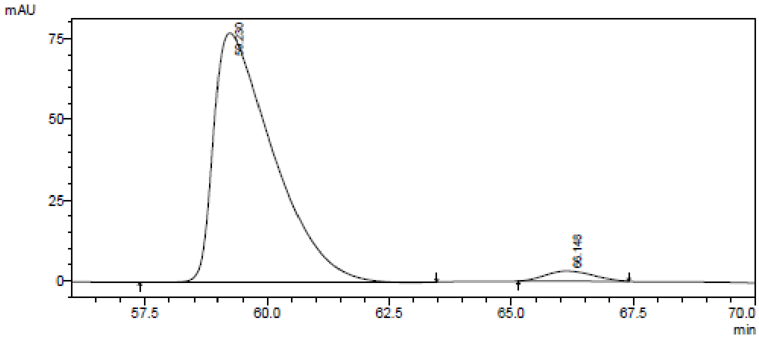

Peak#	Ret. Time	Area	Height	Area %	Height %
1	59.230	6498757	77215	96.820	96.089
2	66.148	213468	3143	3.180	3.911
Total		6712224	80357	100.000	100.000

Supplementary Figure 186 HPLC Spectra of ***R*-63 and *S*-63**

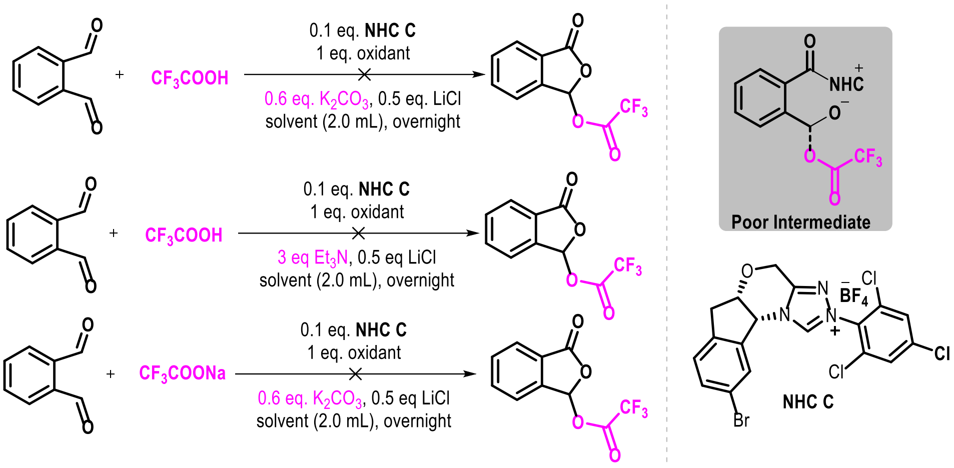

Supplementary Figure 187 Investigation of strong acids under our conditions

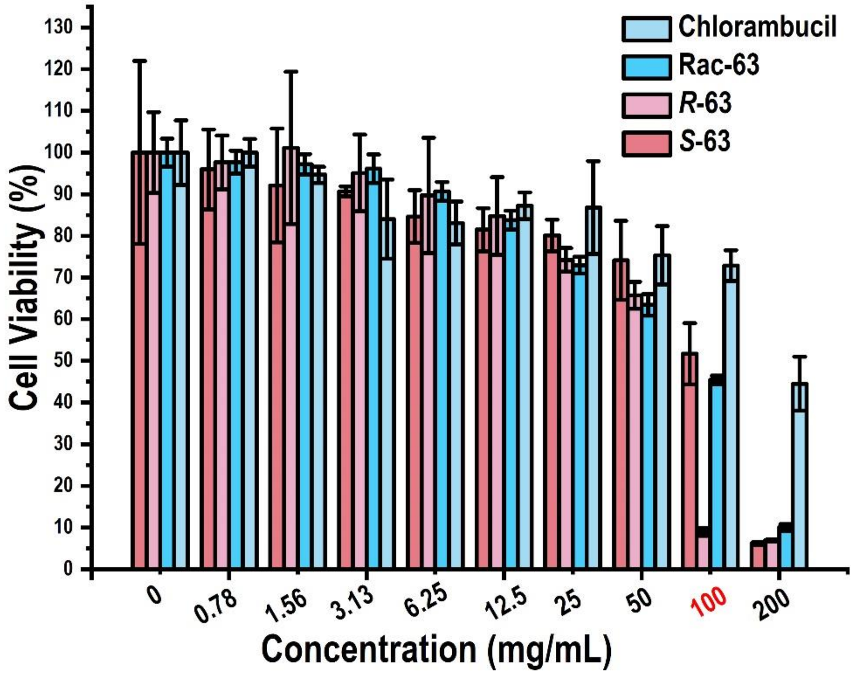

Supplementary Figure 188 Examination of the chiral prodrug, rac-**63**, *R*-**63** and *S*-**63** using Hela cells (n = 3 biological replicates, Mean ±SD). Source data are provided as a Source Data file

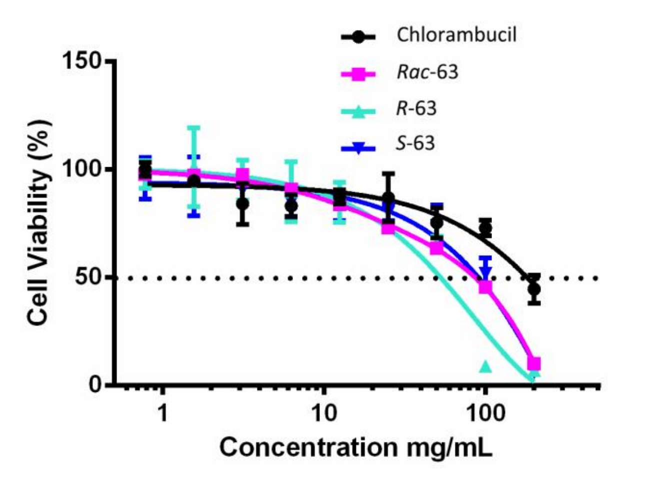

Supplementary Figure 189 IC_{50} curve for chiral phthalidyl ester prodrugs against the growth of Hela cells (n = 3 biological replicates, Mean ±SD). IC_{50} for Chlorambucil: 157 mg/mL; *Rac*-**63**: 83.6 mg/mL; *R*-**63**: 53.6 mg/mL, *S*-**63**: 91.7 mg/mL, Source data are provided as a Source Data file.

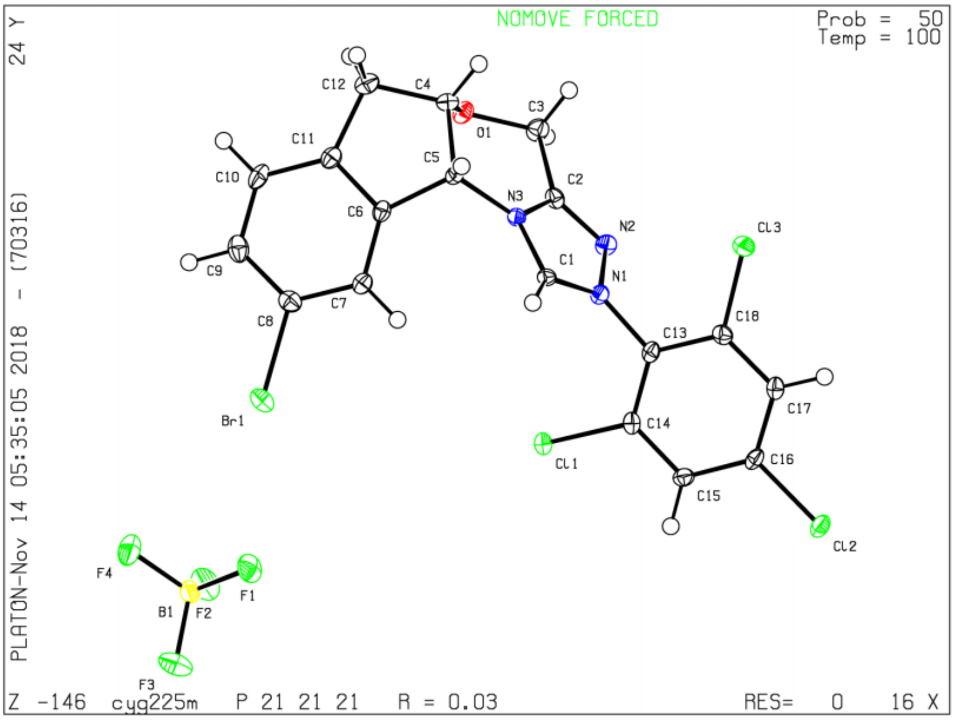

Supplementary Figure 190 X-ray crystallographic structure for NHC **C**

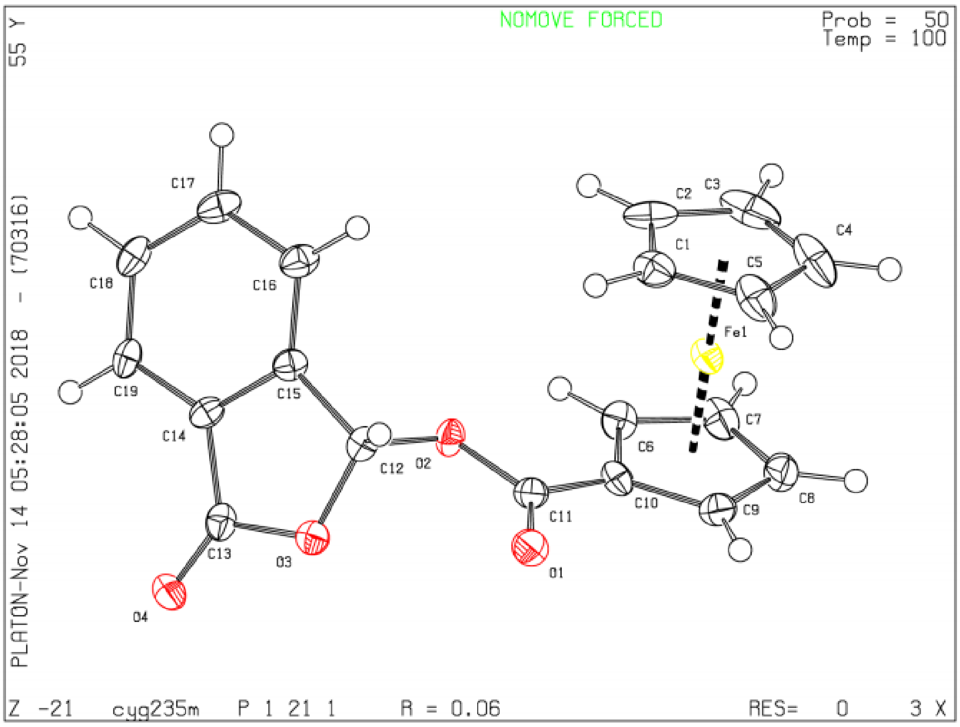

Supplementary Figure 191 X-ray crystallographic structure for Compound **20**

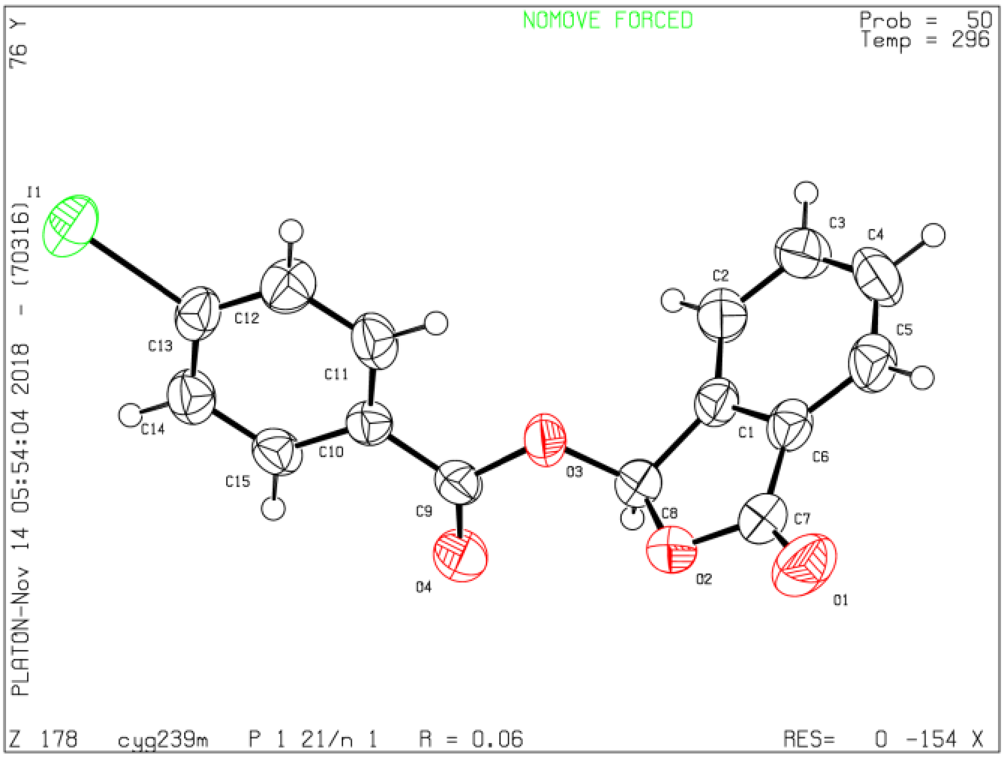

Supplementary Figure 192 X-ray crystallographic structure for Compound **21**

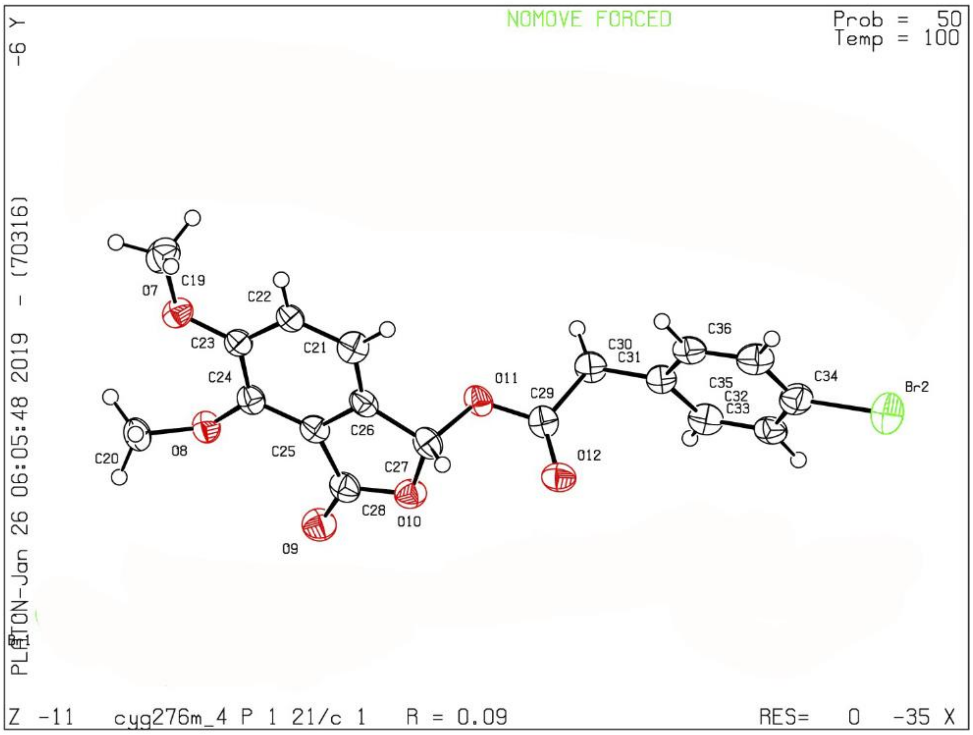

Supplementary Figure 193 X-ray crystallographic structure for Compound **52a**

Supplementary Tables

Supplementary Table 1 Optimization of NHCs

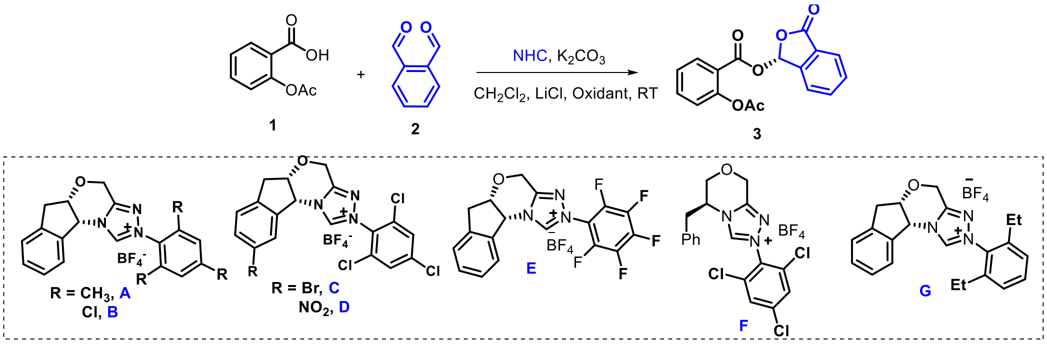

Entry	NHC	Yield (%)	e.r.
1	A	66	60:40
2	B	84	77:23
3	C	92	85:15
4	D	Trace	n.d.
5	E	75	77:23
6	F	Trace	n.d.
7	G	Trace	n.d.
8	no NHC, various Conditions	n.r.	n.d.

Reaction conditions: **1** (0.1 mmol), **2** (0.15 mmol), 0.1 equiv. NHC, 1 equiv. 3,3',5,5'-Tetra-tert-butyldiphenoquinone as oxidant, 0.6 equiv. K_2CO_3, 0.5 equiv. LiCl, CH_2Cl_2 (2 mL), rt, 12 h. Yields determined by isolation. e.r. determined by HPLC. n.r. = no reation. n.d. = not determined.

Supplementary Table 2 Optimization of base and additives at room temperature.

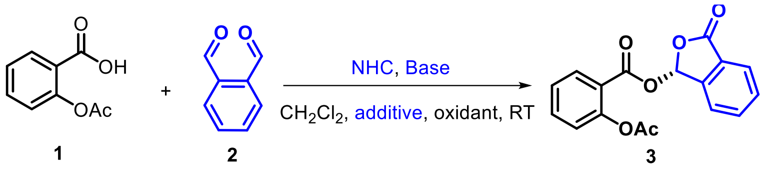

Entry	NHC	Base	Additive	Yield (%)	e.r.
1	**B**	$KHCO_3$	-	83	78:22
2	**B**	K_2CO_3	-	86	77:23
3	**B**	Cs_2CO_3	-	95	77:23
4	**B**	Li_2CO_3	-	Trace	n.d.
5	**B**	$LiOH{\cdot}H_2O$	-	20	77:23
6	**B**	DIPEA	-	n.d.	77:23
7	**B**	TEA	-	n.d.	75:25
8	**B**	K_2CO_3	LiCl	84	78:22
9	**C**	$NaHCO_3$	LiCl	55	82:18
10	**C**	Na_2CO_3	LiCl	61	85:15
11	**C**	$KHCO_3$	LiCl	81	85:15
12	**C**	K_2CO_3	LiCl	92	85:15
13	**C**	$LiOH{\cdot}H_2O$	-	45	84:16
14	**C**	Li_2CO_3	-	36	84:16

Reaction conditions: **1** (0.1 mmol), **2** (0.15 mmol), 0.1 equiv. NHC, 1 equiv. 3,3',5,5'-Tetra-tert-butyldiphenoquinone as oxidant, 0.6 equiv. base, 0.5 equiv. LiCl, CH_2Cl_2 (2 mL), rt, 12 h. Yields determined by isolation. e.r. determined by HPLC n.d. = not determined. TEA = Triethylamine, DIPEA = N, N-Diisopropylethylamine.

Supplementary Table 3 Optimization of solvents with NHC **B** and **C.**

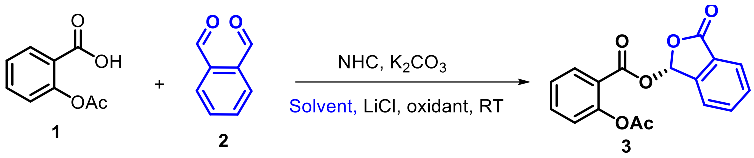

Entry	NHC	Solvent	Yield (%)	e.r.
1	**B**	acetone	n.d.	43:57
2	**B**	CCl_4	n.d.	82:18
3	**B**	ethyl acetate	n.d.	74:26
4	**B**	$PhCF_3$	n.d.	77:23
5	**B**	THF	n.d.	74:26
6	**B**	1,2-dichloroethane	n.d.	76:24
7	**B**	MeCN	n.d.	56:44
8	**B**	diethyl ether	n.d.	71:29
9	**B**	toluene	n.d.	82:18
10	**B**	$CHCl_3$	n.d.	85:15
11	**B**	CH_2Cl_2	n.d.	77:23
12	**C**	$PhCF_3$	76	84:16
13	**C**	dioxane	79	75:15
14	**C**	PhCl	n.d.	83:17
15	**C**	CH_2Cl_2	92	85:15
16	**C**	$CHCl_3$	91	90:10
17	**C**	$CHCl_3$	89	95:15[a]

Reaction conditions: **1** (0.1 mmol), **2** (0.15 mmol), 0.1 equiv. NHC, 1 equiv. 3,3',5,5'-Tetra-tert-butyldiphenoquinone as oxidant, 0.6 equiv. K_2CO_3, 0.5 equiv. LiCl, solvent (2 mL), rt, 12 h. Yields determined by isolation. e.r. determined by HPLC n.d. = not determined. [a] at -20 °C.

Supplementary Methods

Chemicals

Chemicals were commercially purchased from Sigma-Aldrich, TCI, and directly used without further purification unless otherwise stated. N-heterocyclic carbenes were synthesized following the reported procedure.

Chromatography

Analytical thin-layer chromatography (TLC) was carried out on Merck 60 F254 pre-coated silica gel plate (0.2 mm thickness). Visualization was performed using a UV lamp. Column chromatography was carried out on silica gel (60 Å, 40-63 micron) purchased from Davisil with analytical solvents as the eluent. All the yields referred to spectroscopically and chromatographically pure compounds.

Nuclear Magnetic Resonance (NMR) Spectroscopy

Proton nuclear magnetic resonance (^{1}H NMR) spectra were recorded on a Bruker BBFO (400 MHz) spectrometer or AV400 NMR (400 MHz, QNP probe). Chemical shifts were recorded in parts per million (ppm, δ) relative to tetramethylsilane (δ 0.00) or chloroform (δ = 7.26, singlet). ^{1}H NMR splitting patterns are designated as singlet (s), doublet (d), triplet (t), quartet (q), dd (doublet of doublets); m (multiplets), etc. All first-order splitting patterns were assigned on the basis of the appearance of the multiplet. Splitting patterns that could not be easily interpreted are designated as multiplet (m) or broad (br). Carbon nuclear magnetic resonance (^{13}C NMR) spectra were recorded on a Bruker BBFO (100 MHz) spectrometer. Chemical shifts are reported in ppm with the solvent resonance as the internal reference ($CDCl_3$ δ 77.0, CD_3OD δ 49.0).

High Resolution Mass Spectrometry (HRMS)

High resolution mass spectral analysis (HRMS) was performed on Finnigan MAT 95 XP mass spectrometer (Thermo Electron Corporation). The calculated values are based on the most abundant isotope.

Optical rotations

Optical rotations were measured using a 1 mL cell using a sodium lamp (sodium D line, λ = 589 nm) in the indicated solvent at the indicated temperature with a 1 dm path length on a Jasco P-1030 polarimeter and are reported as follows: $[\alpha]^{rt}_{D}$ (*c* in g per 100 mL solvent).

High Performance Liquid Chromatography (HPLC)

HPLC analysis was performed on a Shimadzu LC-15C liquid chromatograph with chiralcel IA, IB, ID, AD-H and OD-H column (Daicel Chemical Industries, Ltd.). The solvents (*n*-hexane and *iso*-propanol, HPLC-grade) used as the eluent were purchased from Sigma Aldrich. The column type and the eluent (a mixture of *n*-hexane and *iso*-propanol) are indicated for each experiment.

X-ray crystallography

X-ray crystallography analysis was performed on Bruker X8 APEX *X*-ray diffractometer.

Synthesis of NHC **C**

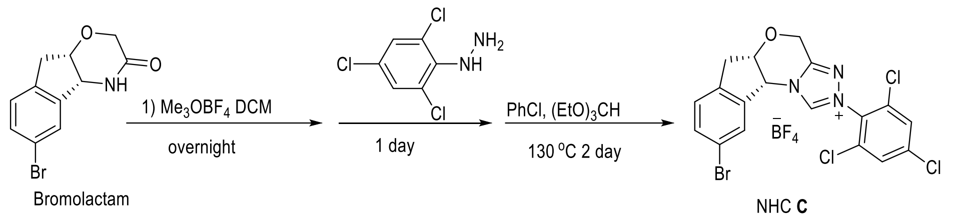

Supplementary Figure 194 Synthesis of NHC **C** The detailed procedure was shown below.

The NHC **C** was synthesized following similar procedure as other NHC catalysts (see Supplementary Figure 187). Bromolactam (4.18g, 15.62 mmol, 1 equiv.), prepared according to Bode[1], is dissolved in CH_2Cl_2 (80 mL) and then Trimethyloxonium tetrafluroborate (2.30g, 15.62 mmol, 1 equiv.) was added. The resulting mixture was stirred at room temperature overnight. 2,4,6-Trichlorophenylhydrazine (3.30g, 15.62 mmol, 1 equiv.) was added and the reaction is stirred for 24 hrs. After completion, CH_2Cl_2 was removed before the crude hydrazide is then taken up in chlorobenzene (60 mL) and triethylorthoformate (10 mL) and the reaction was stirred at 130 ^{o}C for 2 days. The reaction mixture could cool down and the NHC **C** was crystallized from the solution. The filter cake was washed by ethyl acetate and collected in 57% yield as an off-white solid.

Supplementary notes

Supplementary Note 1: N-heterocyclic carbene (NHC) **C**

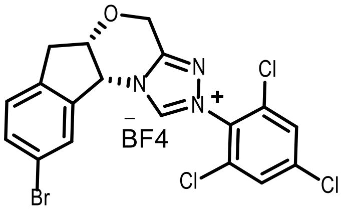

Off-white solid, 57% overall yield

^{1}H NMR (400 MHz, $CDCl_3$) δ 10.56 (s, 1H), 7.57 - 7.44 (m, 4H), 7.21 (d, J = 8.0 Hz, 1H), 6.16 (d, J = 3.9 Hz, 1H), 5.11 (t, J = 4.3 Hz, 1H), 5.06 (s, 2H), 3.32 (d, J = 4.8 Hz, 1H), 3.20-3.10 (m, 1H);

^{13}C NMR (100 MHz, $CDCl_3$) δ 150.28, 145.51, 139.44, 139.08, 137.35, 132.89, 129.34, 129.20, 127.06, 127.03, 121.49, 62.17, 60.18, 37.21.;

HRMS (ESI, m/z): calcd. for $[C_{18}H_{12}BrCl_3N_3O]^+$ 469.9224, found 469.9232;

CCDC codes: CCDC 1866589, the crystallographic data be obtained free of charge from The Cambridge Crystallographic Data Centre via www.ccdc.cam.ac.uk/data_request/cif.

Supplementary Note 2: **Compound 3:**

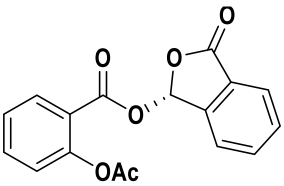

Yellow solid, 89% yield

^{1}H NMR (400 MHz, $CDCl_3$) δ 8.03 (dd, J = 8.0, 1.9 Hz, 1H), 7.97 (dd, J = 7.4, 1.6 Hz, 1H), 7.78 (t, J = 7.5 Hz, 1H), 7.73 – 7.56 (m, 4H), 7.39 – 7.29 (m, 1H), 7.20 – 7.07 (m, 1H), 2.22 (s, 3H);

^{13}C NMR (100 MHz, $CDCl_3$) δ 169.52, 167.72, 162.84, 151.19, 144.27, 135.04, 134.93, 132.18, 131.39, 126.47, 126.17, 125.85, 124.11, 123.89, 121.58, 93.18, 20.81;

IR ν_{max} (film, cm^{-1}): 2091, 1637, 1367, 1192, 970; **$[\alpha]^{21}_{D}$** = -35.5 (c = 3.2 in $CHCl_3$);

HRMS (ESI, m/z): calcd. for $[C_{17}H_{13}O_6]^+$ 313.0712, found 313.0711;

HPLC analysis: 95:5 er, [CHIRALPAK IB column; 0.6 mL/min; solvent system: *i*-PrOH/hexane 5:95; retention times: 28.5 min (minor), 31.2 min (major)].

Supplementary Note 3: **Compound 4:**

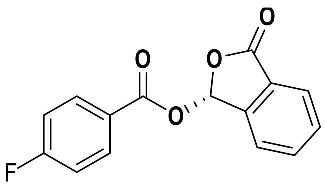

Yellow solid, 88% yield

^{1}H NMR (400 MHz, $CDCl_3$) δ 8.12 – 8.05 (m, 2H), 7.98 (dd, J = 7.3, 1.5 Hz, 1H), 7.82 – 7.75 (m, 1H), 7.72 – 7.66 (m, 3H), 7.14 (d, J = 8.6 Hz, 1H).;

^{13}C NMR (100 MHz, $CDCl_3$) δ 168.10, 167.77, 164.08, 144.35, 134.88, 132.91, 132.78, 131.37, 126.59, 125.88, 124.69, 124.65, 123.70, 116.04, 115.75, 93.29;

IR ν_{max} (film, cm^{-1}): 2091, 1788, 1664, 1400, 1361, 1246, 974; **$[\alpha]^{21}_{D}$** = -59.9 (c = 2.2 in $CHCl_3$);

HRMS (ESI, m/z): calcd. for $[C_{15}H_{10}FO_4]^+$ 273.0563, found 273.0562;

HPLC analysis: 95:5 er, [CHIRALPAK IB column; 0.6 mL/min; solvent system: *i*-PrOH/hexane 5:95; retention times: 16.8 min (minor), 18.7 min (major)].

Supplementary Note 4: **Compound 5:**

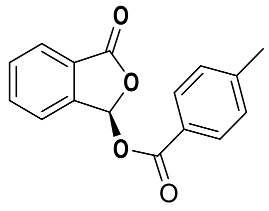

Colorless oil, 72% yield

^{1}H NMR (400 MHz, $CDCl_3$) δ 7.99 – 7.92 (m, 3H), 7.77 (t, J = 7.4, 1H), 7.70 – 7.66 (m, 3H), 7.25 (d, J = 8.1 Hz, 2H), 2.42 (s, 3H);

^{13}C NMR (100 MHz, $CDCl_3$) δ 167.96, 165.09, 145.06, 144.60, 134.84, 131.26, 130.21, 129.33, 126.64, 125.80, 125.63, 123.75, 93.22, 21.77;

IR ν_{max} (film, cm^{-1}): 2091, 1636, 1261, 1083, 955; **$[\alpha]^{21}_D$** = -27.6 (c = 0.5 in $CHCl_3$);

HRMS (ESI, m/z): calcd. for $[C_{16}H_{13}O_4]^+$ 269.0814, found 269.0818;

HPLC analysis: 94:6 er, [CHIRALPAK IB column; 0.6 mL/min; solvent system: i-PrOH/hexane 5:95; retention times: 15.4 min (minor), 16.3 min (major)].

Supplementary Note 5: **Compound 6:**

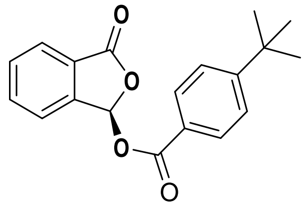

Off-white solid, 82% yield

^{1}H NMR (400 MHz, $CDCl_3$) δ 8.02 – 7.95 (m, 3H), 7.79-7.75 (m, 1H), 7.70 – 7.65 (m, 3H), 7.48 – 7.45 (m, 2H), 1.33 (s, 9H);

^{13}C NMR (100 MHz, $CDCl_3$) δ 167.92, 164.98, 157.94, 144.56, 134.79, 131.20, 130.02, 126.56, 125.73, 125.55, 125.50, 123.67, 93.16, 35.16, 30.98;

IR ν_{max} (film, cm^{-1}): 2972, 2093, 1790, 1639, 1261, 972; **$[\alpha]^{21}_D$** = -61.8 (c = 1.5 in $CHCl_3$);

HRMS (ESI, m/z): calcd. for $[C_{19}H_{19}O_4]^+$ 311.1283, found 311.1284;

HPLC analysis: 90:10 er, [CHIRALPAK IB column; 0.6 mL/min; solvent system: i-PrOH/hexane 5:95; retention times: 13.3 min (minor), 13.9 min (major)].

Supplementary Note 6: **Compound 7:**

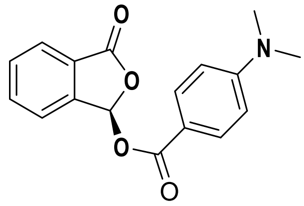

off-white solid, 75% yield

^{1}H NMR (400 MHz, $CDCl_3$) δ 7.98 – 7.87 (m, 3H), 7.75 (td, J = 7.5, 1.2 Hz, 1H), 7.70 – 7.61 (m, 3H), 6.65 – 6.58 (m, 2H), 3.05 (s, 6H);

^{13}C NMR (100 MHz, $CDCl_3$) δ 168.21, 165.14, 153.93, 145.06, 134.65, 132.02, 130.98, 126.72, 125.62, 123.70, 114.45, 110.65, 93.04, 39.96;

IR ν_{max} (film, cm^{-1}): 2093, 1776, 1636, 1267, 1184, 961; **$[\alpha]^{21}_D$** = -72.7 (c = 2.2 in $CHCl_3$);

HRMS (ESI, m/z): calcd. for $[C_{17}H_{16}O_4]^+$ 298.1079, found 298.1079;

HPLC analysis: 94:6 er, [CHIRALPAK IB column; 0.6 mL/min; solvent system: i-PrOH/hexane 5:95; retention times: 42.2 min (major), 46.5 min (minor)].

Supplementary Note 7: **Compound 8:**

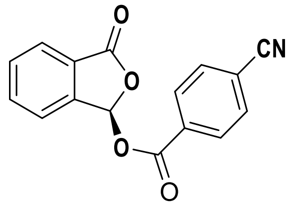

Light yellow solid, 90% yield

^{1}H NMR (400 MHz, $CDCl_3$) δ 8.19 – 8.14 (m, 2H), 7.99 (d, J = 7.5 Hz, 1H), 7.84 – 7.75 (m, 3H), 7.74-7.66 (m, 3H);

^{13}C NMR (100 MHz, $CDCl_3$) δ 167.49, 163.56, 143.87, 135.02, 132.38, 132.20, 131.59, 130.58, 126.47, 126.00, 123.70, 117.58, 117.46, 93.49;

IR ν_{max} (film, cm^{-1}): 2096, 1780, 1738, 1643, 1260, 976; **$[\alpha]^{21}_{D}$** = -41.1 (*c* = 1.9 in $CHCl_3$);
HRMS (ESI, m/z): calcd. for $[C_{16}H_{10}NO_4]^+$ 280.0610, found 280.0618;
HPLC analysis: 92:8 er, [CHIRALPAK IB column; 0.6 mL/min; solvent system: *i*-PrOH/hexane 5:95; retention times: 35.2 min (minor), 41.3 min (major)].

Supplementary Note 8: **Compound 9:**

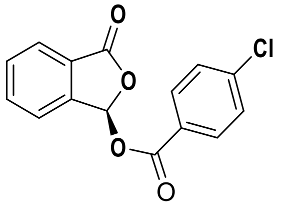

Light yellow solid, 81% yield
^{1}H NMR (400 MHz, $CDCl_3$) δ 8.01-7.97 (m, 3H), 7.78 (t, *J* = 7.5, 1H), 7.72 – 7.66 (m, 3H), 7.46 – 7.42 (m, 2H);
^{13}C NMR (100 MHz, $CDCl_3$) δ 167.73, 164.25, 144.26, 140.71, 134.90, 131.50, 131.40, 129.02, 126.85, 126.57, 125.90, 123.71, 93.32;

IR ν_{max} (film, cm^{-1}): 2091, 1786, 1635, 1260, 1092, 976; **$[\alpha]^{21}_{D}$** = -58.1 (*c* = 0.5 in $CHCl_3$);
HRMS (ESI, m/z): calcd. for $[C_{15}H_{10}O_4Cl]^+$ 280.0610, found 280.0618;
HPLC analysis: 93:7 er, [CHIRALPAK IB column; 0.6 mL/min; solvent system: *i*-PrOH/hexane 5:95; retention times: 17.8 min (minor), 19.6 min (major)].

Supplementary Note 9: **Compound 10:**

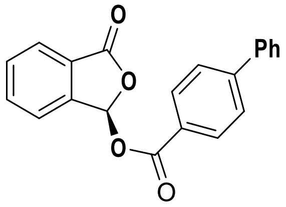

Yellow solid, 77% yield
^{1}H NMR (400 MHz, $CDCl_3$) δ 8.15 – 8.10 (m, 2H), 8.01 – 7.97 (m, 1H), 7.81 – 7.75 (m, 1H), 7.74 – 7.65 (m, 5H), 7.64 – 7.59 (m, 2H), 7.50 – 7.44 (m, 2H), 7.43 – 7.37 (m, 1H);
^{13}C NMR (100 MHz, $CDCl_3$) δ 167.89, 164.95, 146.82, 144.50, 139.61, 134.86, 131.30, 130.69, 128.96, 128.40, 127.28, 127.23, 127.02, 126.62, 125.84, 123.74, 93.29;
IR ν_{max} (film, cm^{-1}): 2092, 1782, 1634, 1258, 1088, 970; **$[\alpha]^{21}_{D}$** = -46.7 (*c* = 0.8 in $CHCl_3$);
HRMS (ESI, m/z): calcd. for $[C_{21}H_{15}O_4]^+$ 331.0970, found 331.0975;
HPLC analysis: 96:4 er, [CHIRALPAK IB column; 0.6 mL/min; solvent system: *i*-PrOH/hexane 5:95; retention times: 26.1 min (minor), 26.8 min (major)]

Supplementary Note 10: **Compound 11:**

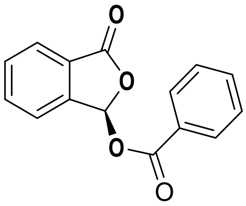

Off-white solid, 90% yield
^{1}H NMR (400 MHz, $CDCl_3$) δ 8.03 (dd, *J* = 8.0, 1.9 Hz, 1H), 7.97 (dd, *J* = 7.4, 1.6 Hz, 1H), 7.78 (t, *J* = 7.5 Hz, 1H), 7.73 – 7.56 (m, 4H), 7.39 – 7.29 (m, 1H), 7.20 – 7.07 (m, 1H), 2.22 (s, 3H);
^{13}C NMR (100 MHz, $CDCl_3$) δ 169.52, 167.72, 162.84, 151.19, 144.27, 135.04, 134.93, 132.18, 131.39, 126.47, 126.17, 125.85, 124.11, 123.89, 121.58, 93.18, 20.81;
IR ν_{max} (film, cm^{-1}): 2091, 1782, 1630, 1261, 970; **$[\alpha]^{21}_{D}$** = -45.9 (*c* = 0.8 in $CHCl_3$);
HRMS (ESI, m/z): calcd. for $[C_{15}H_{11}O_4]^+$ 255.0657, found 255.0658;
HPLC analysis: 95:5 er, [CHIRALPAK IB column; 0.6 mL/min; solvent system: *i*-PrOH/hexane 5:95; retention times: 16.1 min (minor), 17.1 min (major)].

Supplementary Note 11: **Compound 12:**

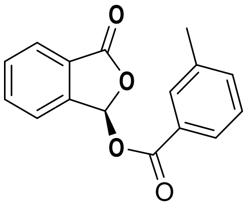

Yellow solid, 91% yield

<u>^{1}H NMR</u> (400 MHz, $CDCl_3$) δ 8.00 – 7.95 (m, 1H), 7.90 – 7.83 (m, 2H), 7.80-7.76 (m, 1.0 Hz, 1H), 7.72 – 7.66 (m, 3H), 7.42 (d, J = 7.5 Hz, 1H), 7.34 (t, J = 7.7 Hz, 1H), 2.39 (s, 3H);

<u>^{13}C NMR</u> (100 MHz, $CDCl_3$) δ 167.89, 165.21, 144.52, 138.48, 134.82, 131.26, 130.59, 128.47, 128.28, 127.31, 126.61, 125.80, 123.74, 93.26, 21.17;

IR ν_{max} (film, cm^{-1}): 2090, 1786, 1734, 1635, 1269, 972; **$[\alpha]^{21}_{D}$** = -45.4 (c = 1.5 in $CHCl_3$);

HRMS (ESI, m/z): calcd. for $[C_{16}H_{13}O_4]^+$ 269.0814, found 269.0816;

HPLC analysis: 89:11 er, [CHIRALPAK IB column; 0.6 mL/min; solvent system: *i*-PrOH/hexane 5:95; retention times: 13.9 min (minor), 14.4 min (major)]

Supplementary Note 12: **Compound 13:**

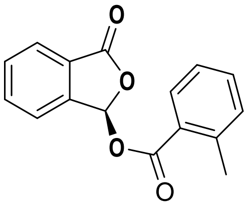

Off-white solid, 94% yield

<u>^{1}H NMR</u> (400 MHz, $CDCl_3$) δ 8.04 – 7.95 (m, 1H), 7.92 (dd, J = 7.9, 1.4 Hz, 1H), 7.81 – 7.74 (m, 1H), 7.72 – 7.63 (m, 3H), 7.45 (td, J = 7.5, 1.5 Hz, 1H), 7.31 – 7.19 (m, 2H), 2.65 (s, 3H);

<u>^{13}C NMR</u> (100 MHz, $CDCl_3$) δ 167.96, 165.43, 144.59, 141.58, 134.81, 133.17, 131.98, 131.23, 131.15, 127.31, 126.65, 125.89, 125.84, 123.63, 93.04, 21.91;

IR ν_{max} (film, cm^{-1}): 2089, 1780, 1734, 1635, 1240, 1053, 972; **$[\alpha]^{21}_{D}$** = -64.6 (c = 1.4 in $CHCl_3$);

HRMS (ESI, m/z): calcd. for $[C_{16}H_{13}O_4]^+$ 269.0814, found 269.0810;

HPLC analysis: 92:8 er, [CHIRALPAK IB column; 0.6 mL/min; solvent system: *i*-PrOH/hexane 5:95; retention times: 16.0 min (minor), 20.8 min (major)]

Supplementary Note 13: **Compound 14:**

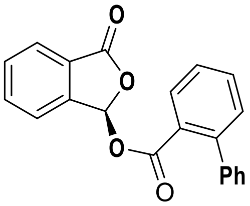

Off-white solid, 82% yield

<u>^{1}H NMR</u> (400 MHz, $CDCl_3$) δ 7.91 (dd, J = 7.8, 1.4 Hz, 1H), 7.86 – 7.83 (m, 1H), 7.64 – 7.54 (m, 3H), 7.43 (td, J = 7.6, 1.3 Hz, 1H), 7.38 – 7.31 (m, 5H), 7.30 – 7.25 (m, 2H), 7.10 – 7.07 (m, 1H);

<u>^{13}C NMR</u> (100 MHz, $CDCl_3$) δ 167.71, 166.93, 143.84, 143.05, 140.94, 134.45, 132.17, 131.00, 130.90, 130.37, 128.85, 128.35, 128.09, 127.33, 127.32, 126.22, 125.49, 123.49, 92.94.;

IR ν_{max} (film, cm^{-1}): 2091, 1790, 1637, 1265, 1234, 1047, 970, 744; **$[\alpha]^{21}_{D}$** = -17.1 (c = 1.1 in $CHCl_3$);

HRMS (ESI, m/z): calcd. for $[C_{21}H_{15}O_4]^+$ 331.0970, found 331.0975;

HPLC analysis: 92:8 er, [CHIRALPAK IB column; 0.6 mL/min; solvent system: *i*-PrOH/hexane 5:95; retention times: 23.1 min (minor), 34.5 min (major)]

Supplementary Note 14: **Compound 15:**

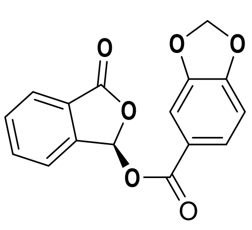

Off-white solid, 79% yield

^{1}H NMR (400 MHz, $CDCl_3$) δ 7.97 (d, J = 7.7 Hz, 1H), 7.83 – 7.74 (m, 1H), 7.67 (m, 4H), 7.46 (d, J = 1.7 Hz, 1H), 6.84 (d, J = 8.2 Hz, 1H), 6.05 (s, 2H);

^{13}C NMR (100 MHz, $CDCl_3$) δ 167.88, 164.32, 152.61, 147.94, 144.52, 134.81, 131.25, 126.62, 126.43, 125.80, 123.69, 122.18, 109.79, 108.17, 102.04, 93.22;

IR ν_{max} (film, cm^{-1}): 2091, 1643, 1260, 1151, 972; **$[\alpha]^{21}_D$** = -21.3 (c = 0.4 in $CHCl_3$);

HRMS (ESI, m/z): calcd. for $[C_{16}H_{11}O_6]^+$ 299.0556, found 299.0562;

HPLC analysis: 94:6 er, [CHIRALPAK IA column; 0.6 mL/min; solvent system: *i*-PrOH/hexane 5:95; retention times: 32.1 min (minor), 40.8 min (major)]

Supplementary Note 15: **Compound 16:**

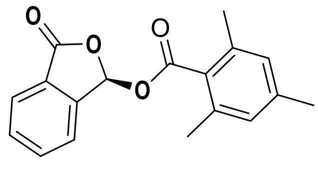

Yellow solid, 69% yield

^{1}H NMR (400 MHz, $CDCl_3$) δ 7.96 – 7.92 (m, 1H), 7.75 (t, J = 7.3, 1H), 7.69 (s, 1H), 7.65 (t, J = 7.0 Hz, 2H), 6.86 (s, 2H), 2.35 (s, 6H), 2.27 (s, 3H);

^{13}C NMR (100 MHz, $CDCl_3$) δ 168.22, 167.86, 144.27, 140.42, 135.82, 134.77, 131.24, 128.66, 128.51, 126.50, 125.81, 123.43, 92.91, 21.11, 19.98;

IR ν_{max} (film, cm^{-1}): 2085, 1789, 1732, 1638, 1163, 972; **$[\alpha]^{21}_D$** = -46.7 (c = 0.8 in $CHCl_3$);

HRMS (ESI, m/z): calcd. for $[C_{21}H_{15}O_4]^+$ 331.0970, found 331.0975;

HPLC analysis: 97:3 er, [CHIRALPAK ID column; 0.6 mL/min; solvent system: *i*-PrOH/hexane 5:95; retention times: 17.7 min (minor), 19.4 min (major)].

Supplementary Note 16: **Compound 17:**

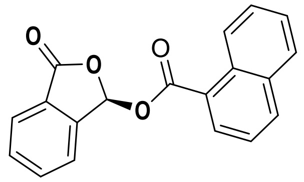

Off-white solid, 58% yield

^{1}H NMR (400 MHz, $CDCl_3$) δ 9.03 (dd, J = 8.7, 1.0 Hz, 1H), 8.23 (dd, J = 7.4, 1.3 Hz, 1H), 8.07 (dt, J = 8.2, 1.1 Hz, 1H), 7.98 (dd, J = 7.6, 1.0 Hz, 1H), 7.92 – 7.89 (m, 1H), 7.82 – 7.64 (m, 6H), 7.59 - 7.55 (m, 1H), 7.47 (dd, J = 8.2, 7.4 Hz, 1H);

^{13}C NMR (100 MHz, $CDCl_3$) δ 167.97, 165.37, 144.55, 134.86, 134.84, 133.81, 131.56, 131.46, 131.27, 128.71, 128.41, 126.64, 126.51, 125.85, 125.47, 124.48, 124.35, 123.69, 93.19;

IR ν_{max} (film, cm^{-1}): 2092, 1788, 1732, 1639, 1238, 972, 777; **$[\alpha]^{21}_D$** = -80.4 (c = 2.0 in $CHCl_3$);

HRMS (ESI, m/z): calcd. for $[C_{19}H_{13}O_4]^+$ 305.0814, found 305.0818;

HPLC analysis: 91:9 er, [CHIRALPAK IB column; 0.6 mL/min; solvent system: *i*-PrOH/hexane 5:95; retention times: 20.9 min (minor), 24.7 min (major)].

Supplementary Note 17: **Compound 18:**

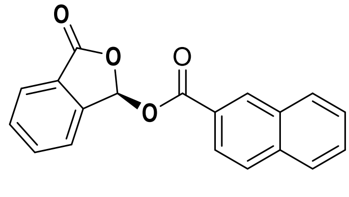

Off-white solid, 66% yield

^{1}H NMR (400 MHz, $CDCl_3$) δ 8.61 (d, J = 1.7 Hz, 1H), 8.04 (dd, J = 8.5, 1.7 Hz, 1H), 8.00 – 7.97 (m, 1H), 7.93 – 7.85 (m, 3H), 7.81 – 7.66 (m, 4H), 7.60 (ddd, J = 8.2, 6.8, 1.4 Hz, 1H), 7.53 (ddd, J = 8.1, 6.9, 1.3 Hz, 1H);

^{13}C NMR (100 MHz, $CDCl_3$) δ 167.91, 165.22, 144.51, 135.94, 134.88, 132.30, 132.11, 131.31, 129.45, 128.87, 128.46, 127.79, 126.92, 126.62, 125.83, 125.51, 125.11, 123.80, 93.38;
IR ν_{max} (film, cm^{-1}): 2093, 1784, 1732, 1636, 1276, 974, 750; **$[\alpha]^{21}_D$** = -72.9 (c = 4.4 in $CHCl_3$);
HRMS (ESI, m/z): calcd. for $[C_{19}H_{13}O_4]^+$ 305.0814, found 305.0819;
HPLC analysis: 93:7 er, [CHIRALPAK IB column; 0.6 mL/min; solvent system: *i*-PrOH/hexane 5:95; retention times: 20.9 min (minor), 24.7 min (major)].

Supplementary Note 18: **Compound 19:**

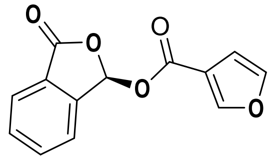

Off-white solid, 64% yield
^{1}H NMR (400 MHz, $CDCl_3$) δ 8.06 (dd, J = 1.6, 0.8 Hz, 1H), 7.97 (dt, J = 7.5, 1.0 Hz, 1H), 7.78 (td, J = 7.5, 1.1 Hz, 1H), 7.71 – 7.64 (m, 2H), 7.62 (s, 1H), 7.46 (t, J = 1.7 Hz, 1H), 6.78 (dd, J = 2.0, 0.8 Hz, 1H);
^{13}C NMR (100 MHz, $CDCl_3$) δ 167.80, 161.27, 149.06, 144.29, 144.21, 134.84, 131.30, 126.56, 125.81, 123.65, 117.80, 109.76, 92.76;
IR ν_{max} (film, cm^{-1}): 2085, 1784, 1639, 1301, 1284, 1161, 974; **$[\alpha]^{21}_D$** = -61.2 (c = 0.7 in $CHCl_3$);
HRMS (ESI, m/z): calcd. for $[C_{13}H_9O_5]^+$ 245.0450, found 245.0457;
HPLC analysis: 93:7 er, [CHIRALPAK IB column; 0.6 mL/min; solvent system: *i*-PrOH/hexane 5:95; retention times: 18.5 min (minor), 19.7 min (major)].

Supplementary Note 19: **Compound 20:**

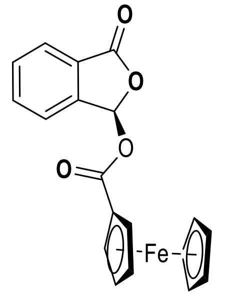

Yellow solid, 88% yield
^{1}H NMR (400 MHz, $CDCl_3$) δ 7.98 (dd, J = 7.6, 1.0 Hz, 1H), 7.78 (td, J = 7.5, 1.1 Hz, 1H), 7.71 – 7.61 (m, 3H), 4.90 (dt, J = 2.6, 1.3 Hz, 1H), 4.78 (dt, J = 2.6, 1.3 Hz, 1H), 4.50 (td, J = 2.6, 1.3 Hz, 1H), 4.46 (td, J = 2.6, 1.4 Hz, 1H), 4.24 (s, 5H);
^{13}C NMR (100 MHz, $CDCl_3$) δ 170.52, 168.06, 144.75, 134.75, 131.14, 126.77, 125.78, 123.52, 92.70, 72.37, 72.24, 70.71, 70.40, 70.20, 68.43;

IR ν_{max} (film, cm^{-1}): 2091, 1782, 1636, 1265, 1107, 966; **$[\alpha]^{21}_D$** = -157.3 (c = 0.8 in $CHCl_3$);
HRMS (ESI, m/z): calcd. for $[C_{20}H_{17}FeO_4]^+$ 377.0476, found 377.0514;
HPLC analysis: 97:3 er, [CHIRALPAK IB column; 0.6 mL/min; solvent system: *i*-PrOH/hexane 5:95; retention times: 23.2 min (minor), 24.4 min (major)].
CCDC codes: CCDC 1866428, the crystallographic data be obtained free of charge from The Cambridge Crystallographic Data Centre via www.ccdc.cam.ac.uk/data_request/cif.

Supplementary Note 20: **Compound 21:**

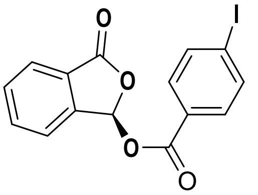

Off-white solid, 68% yield
^{1}H NMR (400 MHz, $CDCl_3$) δ 7.98 (d, J = 7.5 Hz, 1H), 7.86 – 7.64 (m, 8H);
^{13}C NMR (100 MHz, $CDCl_3$) δ 167.71, 164.67, 144.24, 138.02, 134.91, 131.41, 127.86, 126.56, 125.90, 123.71, 102.25, 93.33;

IR ν_{max} (film, cm^{-1}): 2093, 1782, 1738, 1639, 1584, 1256, 976, 748; **$[\alpha]^{21}_D$** = -40.8023 (c = 1.8 in $CHCl_3$);

HRMS (ESI, m/z): calcd. for $[C15H_{10}IO_4]^+$ 380.9624, found 380.9636;

HPLC analysis: 91:9 er, [CHIRALPAK IA column; 0.6 mL/min; solvent system: *i*-PrOH/hexane 5:95; retention times: 23.6 min (minor), 32.4 min (major)].

CCDC codes: CCDC 1866429, the crystallographic data be obtained free of charge from The Cambridge Crystallographic Data Centre via www.ccdc.cam.ac.uk/data_request/cif.

Supplementary Note 21: **Compound 22:**

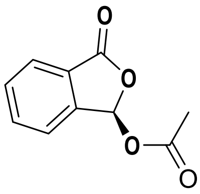

Colorless oil, 82% yield

^{1}H NMR (400 MHz, $CDCl_3$) δ 7.94 (d, J = 7.5 Hz, 2H), 7.84 – 7.72 (m, 2H), 7.64 (m, 4H), 7.43 (s, 1H), 2.20 (s, 3H);

^{13}C NMR (100 MHz, $CDCl_3$) δ 169.40, 167.78, 144.26, 134.77, 131.24, 126.49, 125.80, 123.51, 92.63, 20.81;

IR ν_{max} (film, cm^{-1}): 2091, 1628, 1570,1356, 1217; **$[\alpha]^{21}_D$** = -32.4 (c = 0.6 in $CHCl_3$);

HRMS (ESI, m/z): calcd. for $[C_{10}H_9O_4]^+$ 193.0512, found 193.0423;

HPLC analysis: 97:3 er, [CHIRALPAK IB column; 0.6 mL/min; solvent system: *i*-PrOH/hexane 5:95; retention times: 16.8 min (minor), 18.8 min (major)].

Supplementary Note 22: **Compound 23:**

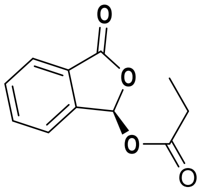

Colorless oil, 90% yield

^{1}H NMR (400 MHz, $CDCl_3$) δ 7.93 (d, J = 7.6 Hz, 1H), 7.75 (t, J = 7.5, 1H), 7.66 (t, J = 7.5, 1H), 7.59 (d, J = 7.6 Hz, 1H), 7.46 (s, 1H), 2.46 (q, J = 7.5 Hz, 2H), 1.21 (t, J = 7.5 Hz, 3H);

^{13}C NMR (100 MHz, $CDCl_3$) δ 172.91, 167.84, 144.37, 134.74, 131.18, 126.51, 125.75, 123.47, 92.60, 27.40, 8.65;

IR ν_{max} (film, cm^{-1}): 2089, 1634, 1357, 1284, 1144, 968; **$[\alpha]^{21}_D$** = -71.6 (c = 1.5 in $CHCl_3$);

HRMS (ESI, m/z): calcd. for $[C_{11}H_{11}O_4]^+$ 207.0657, found 207.0664;

HPLC analysis: 95:5 er, [CHIRALPAK IB column; 0.6 mL/min; solvent system: *i*-PrOH/hexane 5:95; retention times: 13.7 min (minor), 15.6 min (major)].

Supplementary Note 23: **Compound 24:**

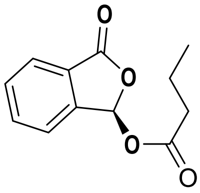

Colorless oil, 95% yield

^{1}H NMR (400 MHz, $CDCl_3$) δ 7.94 (dt, J = 7.6, 1.0 Hz, 1H), 7.75 (td, J = 7.5, 1.1 Hz, 1H), 7.66 (td, J = 7.4, 1.0 Hz, 1H), 7.59 (m, 1H), 7.46 (s, 1H), 2.42 (t, J = 7.3 Hz, 2H), 1.72 (m, 2H), 0.99 (t, J = 7.4 Hz, 3H);

^{13}C NMR (100 MHz, $CDCl_3$) δ 172.09, 167.87, 144.42, 134.75, 131.18, 126.52, 125.77, 123.45, 92.55, 35.84, 18.07, 13.50;

IR ν_{max} (film, cm^{-1}): 2965, 1638, 1568, 1261, 1099, 787; **$[\alpha]^{21}_D$** = -37.0 (c = 1.1 in $CHCl_3$);

HRMS (ESI, m/z): calcd. for $[C_{12}H_{12}O_4]^+$ 221.0814, found 221.0811;

HPLC analysis: 97:3 er, [CHIRALPAK IB column; 0.6 mL/min; solvent system: *i*-PrOH/hexane 5:95; retention times: 11.0 min (minor), 12.7 min (major)].

Supplementary Note 24: **Compound 25:**

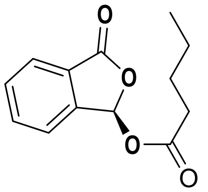

Yellow solid, 68% yield

^{1}H NMR (400 MHz, $CDCl_3$) δ 7.93 (d, J = 7.6 Hz, 1H), 7.76 (td, J = 7.5, 1.0 Hz, 1H), 7.66 (dd, J = 11.0, 4.0 Hz, 1H), 7.59 (d, J = 7.6 Hz, 1H), 7.45 (s, 1H), 2.44 (t, J = 7.5 Hz, 2H), 1.73 – 1.61 (m, 2H), 1.45 – 1.32 (m, 2H), 0.93 (t, J = 7.3 Hz, 3H);

^{13}C NMR (100 MHz, $CDCl_3$) δ 172.25, 167.86, 144.40, 134.75, 131.17, 126.49, 125.73, 123.45, 92.54, 33.70, 26.54, 22.06, 13.59;

IR ν_{max} (film, cm^{-1}): 2091, 1638, 1466, 1356, 1054, 970; **$[\alpha]^{21}_{D}$** = -15.6 (c = 1 in $CHCl_3$);

HRMS (ESI, m/z): calculated for $[C_{13}H_{15}O_4]^+$: 235.0965, found: 235.0970.

HPLC analysis: 98:2 er, [CHIRALPAK OD-H column; 0.7 mL/min; solvent system: *i*-PrOH/hexane 20:80; retention times: 7.8 min (minor), 8.9 min (major)].

Supplementary Note 25: **Compound 26:**

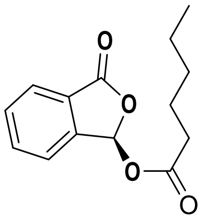

Yellow solid, 65% yield

^{1}H NMR (400 MHz, $CDCl_3$) δ 7.94 (d, J = 7.6 Hz, 1H), 7.75 (td, J = 7.5, 1.0 Hz, 1H), 7.65 (t, J = 7.3 Hz, 1H), 7.58 (d, J = 7.6 Hz, 1H), 7.45 (s, 1H), 2.43 (t, J = 7.5 Hz, 2H), 1.74 – 1.63 (m, 2H), 1.39 – 1.29 (m, 4H), 0.94 – 0.86 (m, 3H);

^{13}C NMR (100 MHz, $CDCl_3$) δ 172.28, 167.88, 144.43, 134.75, 131.18, 126.53, 125.77, 123.46, 92.57, 33.97, 31.09, 24.20, 22.20, 13.82;

IR ν_{max} (film, cm^{-1}): 2874, 2091, 1782, 1643, 1355, 1284, 1213, 972; **$[\alpha]^{21}_{D}$** = -13.1 (c = 1 in $CHCl_3$);

HRMS (ESI, m/z): calculated for $[C_{14}H_{17}O_4]^+$: 249.1121, found: 249.1121.

HPLC analysis: 98:2 er, [CHIRALPAK OD-H column; 0.7 mL/min; solvent system: *i*-PrOH/hexane 20:80; retention times: 7.6 min (minor), 8.7 min (major)].

Supplementary Note 26: **Compound 27:**

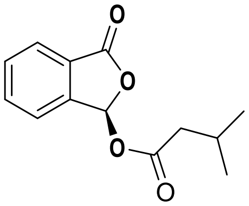

Yellow oil, 75% yield

^{1}H NMR (400 MHz, $CDCl_3$) δ 7.94 (d, J = 7.6 Hz, 1H), 7.76 (td, J = 7.5, 1.0 Hz, 1H), 7.66 (t, J = 7.5 Hz, 1H), 7.58 (d, J = 7.6 Hz, 1H), 7.45 (s, 1H), 2.73 – 2.58 (m, 1H), 1.24 (s, 3H), 1.22 (s, 3H);

^{13}C NMR (100 MHz, $CDCl_3$) δ 175.53, 167.90, 144.47, 134.77, 131.16, 126.51, 125.74, 123.40, 92.62, 33.87, 18.58, 18.56;

IR ν_{max} (film, cm^{-1}): 2964, 2935, 2874, 2081, 1782, 1643, 1470, 1360, 1284, 976; **$[\alpha]^{21}_{D}$** = -16.9 (c = 1 in $CHCl_3$);

HRMS (ESI, m/z): calculated for $[C_{13}H_{15}O_4]^+$: 235.0965, found: 235.0970;

HPLC analysis: 97:3 er, [CHIRALPAK OD-H column; 0.7 mL/min; solvent system: *i*-PrOH/hexane 20:80; retention times: 7.4 min (minor), 8.6 min (major)].

Supplementary Note 27: **Compound 28:**

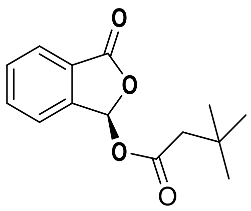

Yellow oil, 76% yield

^{1}H NMR (400 MHz, $CDCl_3$) δ 7.93 (d, J = 7.6 Hz, 1H), 7.75 (td, J = 7.5, 1.0 Hz, 1H), 7.66 (t, J = 7.5 Hz, 1H), 7.58 (d, J = 7.6 Hz, 1H), 7.46 (s, 1H), 2.32 (s, 2H), 1.07 (s, 10H);

^{13}C NMR (100 MHz, $CDCl_3$) δ 170.63, 167.89, 144.48, 134.74, 131.14, 126.51, 125.74, 123.41, 92.40, 47.44, 31.00, 29.51;

IR ν_{max} (film, cm^{-1}): 2963, 2872, 2081, 1790, 1643, 1215, 1117, 974; **$[\alpha]^{21}_D$** = -15.4 (c = 1 in $CHCl_3$);

HRMS (ESI, m/z): calculated for $[C_{14}H_{17}O_4]^+$: 249.1121, found: 249.1122;

HPLC analysis: 98:2 er, [CHIRALPAK OD-H column; 0.7 mL/min; solvent system: *i*-PrOH/hexane 20:80; retention times: 7.0 min (minor), 7.8 min (major)].

Supplementary Note 28: **Compound 29:**

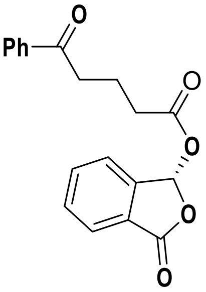

Yellow solid, 96% yield

^{1}H NMR (400 MHz, $CDCl_3$) δ 7.98 – 7.89 (m, 3H), 7.74 (td, J = 7.5, 1.0 Hz, 1H), 7.65 (t, J = 7.3 Hz, 1H), 7.63 – 7.53 (m, 2H), 7.50 – 7.42 (m, 3H), 3.19 – 3.01 (m, 2H), 2.67 – 2.48 (m, 2H), 2.14 (p, J = 7.1 Hz, 2H);

^{13}C NMR (100 MHz, $CDCl_3$) δ 199.05, 171.79, 167.78, 144.21, 136.64, 134.78, 133.16, 131.21, 128.61, 127.95, 126.43, 125.73, 123.51, 92.57, 37.00, 33.05, 18.86;

IR ν_{max} (film, cm^{-1}): 2091, 1782, 1762, 1635, 1130, 1053, 970; **$[\alpha]^{21}_D$** = 0.9 (c = 1 in $CHCl_3$);

HRMS (ESI, m/z): calculated for $[C_{19}H_{17}O_5]^+$: 325.1071, found: 325.1083;

HPLC analysis: 93:7 er, [CHIRALPAK OD-H column; 0.7 mL/min; solvent system: *i*-PrOH/hexane 20:80; retention times: 7.0 min (minor), 7.8 min (major)].

Supplementary Note 29: **Compound 30:**

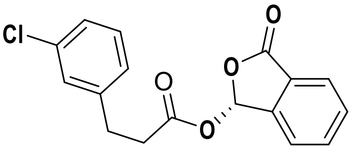

Yellow solid, 92% yield

^{1}H NMR (400 MHz, $CDCl_3$) δ 7.94 – 7.91 (m, 1H), 7.74 (t, J = 7.5, 1H), 7.65 (t, J = 7.5, 1H), 7.49 (d, J = 7.6 Hz, 1H), 7.42 (s, 1H), 7.23 – 7.18 (m, 3H), 7.09 (d=t, J = 6.9, 1H), 2.98 (t, J = 8.0 Hz, 2H), 2.78 – 2.73 (m, 2H);

^{13}C NMR (100 MHz, $CDCl_3$) δ 171.00, 167.70, 144.12, 141.68, 134.79, 134.29, 131.24, 129.85, 128.45, 126.72, 126.51, 126.38, 125.76, 123.47, 92.66, 35.22, 30.09;

IR ν_{max} (film, cm^{-1}): 2085, 1780, 1635, 1360, 1285, 1134, 974; **$[\alpha]^{21}_D$** = -31.9 (c = 4.3 in $CHCl_3$);

HRMS (ESI, m/z): calculated for $[C_{17}H_{14Cl}O_4]^+$: 317.0581, found: 317.0585;

HPLC analysis: 97:3 er, [CHIRALPAK IB column; 0.6 mL/min; solvent system: *i*-PrOH/hexane 5:95; retention times: 26.3 min (minor), 29.2 min (major)].

Supplementary Note 30: **Compound 31:**

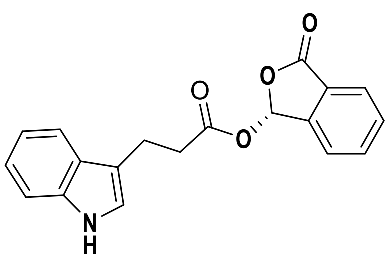

Yellow oil, 68% yield

^{1}H NMR (400 MHz, $CDCl_3$) δ 8.09 (br, 1H), 7.89 (d, J = 7.4 Hz, 1H), 7.70 – 7.54 (m, 3H), 7.42 – 7.32 (m, 3H), 7.19 (t, J = 7.5 Hz, 1H), 7.10 (t, J = 7.4 Hz, 1H), 7.01 (s, 1H), 3.15 (t, J = 7.4 Hz, 2H), 2.83 (t, J = 7.4 Hz, 2H);

^{13}C NMR (100 MHz, $CDCl_3$) δ 171.87, 167.95, 144.21, 136.22, 134.73, 131.12, 126.97, 126.33, 125.64, 123.46, 122.05, 121.74, 119.35, 118.49, 114.01, 111.21, 92.60, 34.79, 20.31;

IR ν_{max} (film, cm^{-1}): 2091, 1636, 1355, 1261, 1215, 962; **$[\alpha]^{21}_D$** = -58.0 (c = 1 in $CHCl_3$);

HRMS (ESI, m/z): calculated for $[C_{19}H_{16}NO_4]^+$: 322.1074, found: 322.1062;

HPLC analysis: 95:5 er, [CHIRALPAK OD-H column; 0.7 mL/min; solvent system: *i*-PrOH/hexane 20:80; retention times: 31.6 min (minor), 38.8 min (major)].

Supplementary Note 31: **Compound 32:**

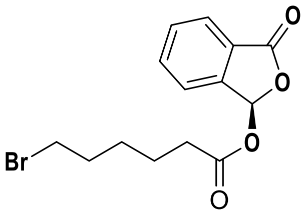

Yellow oil, 85% yield

^{1}H NMR (400 MHz, $CDCl_3$) δ 7.94 (d, J = 7.6 Hz, 1H), 7.76 (td, J = 7.5, 1.1 Hz, 1H), 7.70 – 7.63 (m, 1H), 7.60 (d, J = 7.6 Hz, 1H), 7.45 (s, 1H), 3.41 (t, J = 6.7 Hz, 2H), 2.46 (t, J = 7.4 Hz, 2H), 1.89 (m, 2H), 1.72 (m, 2H), 1.57 – 1.47 (m, 2H);

^{13}C NMR (100 MHz, $CDCl_3$) δ 171.86, 167.78, 144.25, 134.78, 131.21, 126.44, 125.75, 123.46, 92.56, 33.74, 33.32, 32.20, 27.41, 23.62;

IR ν_{max} (film, cm^{-1}): 2090, 1757, 1637, 1465, 1356, 1053, 972; **$[\alpha]^{21}_D$** = -36.9 (c = 0.4 in $CHCl_3$);

HRMS (ESI, m/z): calculated for $[C_{14}H_{16}BrO_4]^+$: 327.0232, found: 327.0234;

HPLC analysis: 97:3 er, [CHIRALPAK IB column; 0.6 mL/min; solvent system: *i*-PrOH/hexane 5:95; retention times: 21.8 min (minor), 29.1 min (major)].

Supplementary Note 32: **Compound 33:**

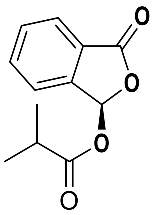

Yellow oil, 78% yield

^{1}H NMR (400 MHz, $CDCl_3$) δ 7.94 (d, J = 7.6 Hz, 1H), 7.76 (td, J = 7.5, 1.0 Hz, 1H), 7.66 (t, J = 7.5 Hz, 1H), 7.58 (d, J = 7.6 Hz, 1H), 7.45 (s, 1H), 2.73 – 2.58 (m, 1H), 1.24 (s, 3H), 1.22 (s, 3H);

^{13}C NMR (100 MHz, $CDCl_3$) δ 175.53, 167.90, 144.47, 134.77, 131.16, 126.51, 125.74, 123.40, 92.62, 33.87, 18.58, 18.56;

IR ν_{max} (film, cm^{-1}): 2093, 1784, 1643, 1360, 1285, 1053, 974; **$[\alpha]^{21}_D$** = -19.8 (c = 1 in $CHCl_3$);

HRMS (ESI, m/z): calculated for $[C_{12}H_{13}O_4]^+$: 221.0808, found: 221.0821;

HPLC analysis: 97:3 er, [CHIRALPAK OD-H column; 0.7 mL/min; solvent system: *i*-PrOH/hexane 20:80; retention times: 7.4 min (minor), 8.2 min (major)].

Supplementary Note 33: **Compound 34:**

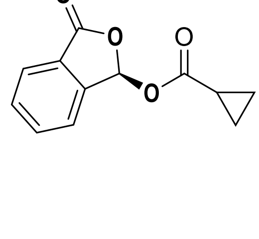

White solid, 90% yield

^{1}H NMR (400 MHz, $CDCl_3$) δ 7.94 (d, J = 7.5, 1H), 7.76 (td, J = 7.5, 1.1 Hz, 1H), 7.66 (t, J = 7.5, 1H), 7.61 (d, J = 7.6, 1H), 7.44 (s, 1H), 1.72-1.66 (m, 1H), 1.18-1.12 (m, 2H), 1.03 – 0.98 (m, 2H);

^{13}C NMR (100 MHz, $CDCl_3$) δ 173.48, 167.86, 144.40, 134.73, 131.16, 126.54, 125.73, 123.52, 92.63, 12.75, 9.64, 9.56;

IR ν_{max} (film, cm^{-1}): 2924, 2855, 1784, 1643, 1470, 1260, 750; **$[\alpha]^{21}_D$** = -62.7 (*c* = 1.6 in $CHCl_3$);

HRMS (ESI, m/z): calculated for $[C_{12}H_{11}O_4]^+$: 219.0657, found: 219.0654;

HPLC analysis: 95:6 er, [CHIRALPAK IB column; 0.6 mL/min; solvent system: *i*-PrOH/hexane 5:95; retention times: 15.0 min (minor), 16.3 min (major)].

Supplementary Note 34: **Compound 35:**

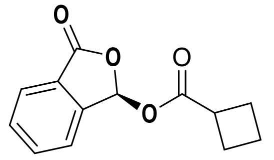

Yellow oil, 80% yield

^{1}H NMR (400 MHz, $CDCl_3$) δ 7.93 (d, *J* = 7.6 Hz, 1H), 7.75 (t, *J* = 7.5 Hz, 1H), 7.65 (t, *J* = 7.5 Hz, 1H), 7.58 (d, *J* = 7.6 Hz, 1H), 7.45 (s, 1H), 3.23 (p, *J* = 8.5 Hz, 1H), 2.43 – 2.30 (m, 2H), 2.30 – 2.19 (m, 2H), 2.09 – 1.88 (m, 2H);

^{13}C NMR (100 MHz, $CDCl_3$) δ 173.86, 167.91, 144.46, 134.75, 131.16, 126.52, 125.74, 123.45, 92.65, 37.67, 25.15, 24.94, 18.35;

IR ν_{max} (film, cm^{-1}): 2962, 2872, 2087, 1643, 1470, 1358, 1141, 970; **$[\alpha]^{21}_D$** = -10.4 (*c* = 1 in $CHCl_3$);

HRMS (ESI, m/z): calculated for $[C_{13}H_{13}O_4]^+$: 233.0808, found: 233.0806

HPLC analysis: 97:3 er, [CHIRALPAK OD-H column; 0.7 mL/min; solvent system: *i*-PrOH/hexane 20:80; retention times: 7.4 min (minor), 8.2 min (major)].

Supplementary Note 35: **Compound 36:**

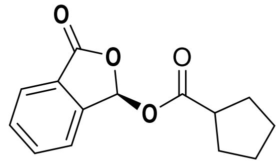

colorless oil, 91% yield

^{1}H NMR (400 MHz, $CDCl_3$) δ 7.93 (dt, *J* = 7.5, 1.0 Hz, 1H), 7.75 (td, *J* = 7.5, 1.1 Hz, 1H), 7.65 (td, *J* = 7.5, 1.0 Hz, 1H), 7.58 (d, *J* = 7.6, 1H), 7.45 (s, 1H), 2.89 – 2.78 (m, 1H), 2.00 – 1.81 (m, 4H), 1.79 – 1.68 (m, 2H), 1.66 – 1.56 (m, 2H);

^{13}C NMR (100 MHz, $CDCl_3$) δ 175.20, 167.92, 144.54, 134.73, 131.13, 126.55, 125.74, 123.40, 92.66, 43.49, 29.87, 29.79, 25.82, 25.78;

IR ν_{max} (film, cm^{-1}): 3030, 2965, 2872, 2099, 1769, 1643, 1470, 1360, 1284, 748; **$[\alpha]^{21}_D$** = -44.2 (*c* = 1.9 in $CHCl_3$);

HRMS (ESI, m/z): calculated for $[C_{14}H_{15}O_4]^+$: 247.0970, found: 247.0975

HPLC analysis: 95:5 er, [CHIRALPAK IB column; 0.6 mL/min; solvent system: *i*-PrOH/hexane 5:95; retention times: 12.1 min (minor), 13.2 min (major)].

Supplementary Note 36: **Compound 37:**

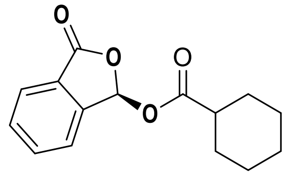

Light yellow semisolid, 90% yield

^{1}H NMR (400 MHz, $CDCl_3$) δ 7.93 (dt, *J* = 7.6, 1.0 Hz, 1H), 7.75 (td, *J* = 7.5, 1.1 Hz, 1H), 7.68 – 7.63 (m, 1H), 7.59 – 7.56 (m, 1H), 7.45 (s, 1H), 2.41 (tt, *J* = 11.2, 3.6 Hz, 1H), 1.99 – 1.91 (m, 2H), 1.82 – 1.72 (m, 2H), 1.68 – 1.62 (m, 1H), 1.57 - 1.44 (m, 2H), 1.36 – 1.20 (m, 3H);

^{13}C NMR (100 MHz, $CDCl_3$) δ 174.41, 167.90, 144.54, 134.72, 131.12, 126.53, 125.73, 123.39, 92.55, 42.86, 28.66, 28.60, 25.54, 25.18, 25.16;

IR ν_{max} (film, cm^{-1}): 3066, 3005, 2947, 2855, 2100, 1738, 1635, 1470, 1310, 894; **$[\alpha]^{21}_D$** = -42.8 (*c* = 2.3 in $CHCl_3$);

HRMS (ESI, m/z): calculated for $[C_{15}H_{17}O_4]^+$: 261.1127, found: 261.1133

HPLC analysis: 95:5 er, [CHIRALPAK IB column; 0.6 mL/min; solvent system: *i*-PrOH/hexane 5:95; retention times: 12.1 min (minor), 13.2 min (major)].

Supplementary Note 37: **Compound 38:**

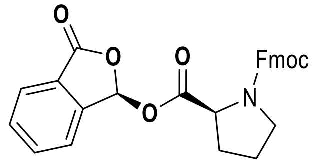

Light yellow oil, 80% yield

^{1}H NMR (400 MHz, $CDCl_3$) δ 7.95 – 7.88 (m, 1H), 7.87 – 7.70 (m, 3H), 7.69 – 7.55 (m, 4H), 7.49 (s, 1H), 7.45 – 7.22 (m, 5H), 4.55 – 4.17 (m, 4H), 3.78 – 3.61 (m, 1H), 3.59 – 3.48 (m, 1H), 2.35 – 2.18 (m, 1H), 2.17 – 1.89 (m, 3H);

^{13}C NMR (100 MHz, $CDCl_3$) δ 171.33, 171.17, 167.85, 154.94, 154.15, 144.19, 144.00, 143.97, 143.79, 143.50, 141.31, 141.28, 134.96, 134.76, 131.36, 131.29, 127.73, 127.08, 127.05, 127.00, 126.32, 125.82, 125.66, 125.17, 125.13, 125.05, 124.93, 123.87, 123.53, 119.99, 92.89, 92.81, 67.66, 58.93, 58.59, 47.28, 47.15, 47.06, 46.57, 30.85, 29.58, 24.32, 23.39;

IR ν_{max} (film, cm^{-1}): 2857, 2090, 1788, 1664, 1417, 1354, 1146, 974; **$[\alpha]^{21}_D$** = 2.1 (*c* = 1 in $CHCl_3$);

HRMS (ESI, m/z): calculated for $[C_{28}H_{24}NO_6]^+$: 470.1598, found: 470.1602;

HPLC analysis: >13:1 dr, [CHIRALPAK OD-H column; 0.7 mL/min; solvent system: *i*-PrOH/hexane 30:70; retention times: 65.6 min (major), 84.5 min (minor)].

Supplementary Note 38: **Compound 39:**

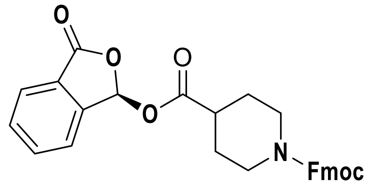

Yellow solid, 86% yield

^{1}H NMR (400 MHz, $CDCl_3$) δ 7.94 (d, *J* = 7.6 Hz, 1H), 7.79 – 7.71 (m, 3H), 7.69 – 7.62 (m, 1H), 7.60 – 7.53 (m, 3H), 7.44 (s, 1H), 7.39 (t, *J* = 7.5 Hz, 2H), 7.30 (td, *J* = 7.4, 1.0 Hz, 2H), 4.43 (br, 2H), 4.22 (t, *J* = 6.6 Hz, 1H), 4.08 – 3.94 (m, 2H), 2.91 (t, *J* = 11.4 Hz, 2H), 2.58 (tt, *J* = 10.8, 3.9 Hz, 1H), 1.89 (br, 2H), 1.64 (br, 2H);

^{13}C NMR (100 MHz, $CDCl_3$) δ 172.75, 167.67, 154.96, 144.11, 143.88, 141.25, 134.81, 131.29, 127.61, 126.97, 126.36, 125.79, 124.82, 123.33, 119.90, 92.63, 67.15, 47.29, 42.92, 42.90, 40.62, 27.37;

IR ν_{max} (film, cm^{-1}): 2087, 1784, 1645, 1446, 1284, 974; **$[\alpha]^{21}_D$** = -5.7 (*c* = 2 in $CHCl_3$);

HRMS (ESI, m/z): calculated for $[C_{29}H_{26}NO_6]^+$: 484.1755, found: 484.1753;

HPLC analysis: 98:2 er, [CHIRALPAK OD-H column; 0.7 mL/min; solvent system: *i*-PrOH/hexane 30:70; retention times: 31.2 min (minor), 54.4 min (major)].

Supplementary Note 39: **Compound 40:**

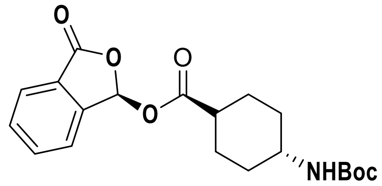

Colorless semisolid, 80% yield

^{1}H NMR (400 MHz, $CDCl_3$) δ 7.93 (d, *J* = 7.6 Hz, 1H), 7.76 (td, *J* = 7.5, 1.0 Hz, 1H), 7.67 (dt, *J* = 7.5, 3.7 Hz, 1H), 7.58 (d, *J* = 7.6 Hz, 1H), 7.43 (s, 1H), 4.49 (br, 1H), 3.42 (br, 1H), 2.33 (tt, *J* = 12.0, 3.2 Hz, 1H), 2.11 – 2.04 (m, 4H), 1.65 – 1.51 (m, 2H), 1.43 (s, 9H), 1.20 – 1.05 (m, 2H);

^{13}C NMR (100 MHz, $CDCl_3$) δ 173.74, 167.77, 155.05, 144.25, 134.78, 131.17, 126.35, 125.68, 123.35, 92.53, 42.02, 32.15, 32.11, 28.29, 27.42, 27.36;

IR ν_{max} (film, cm^{-1}): 2089, 1784, 1651, 1365, 1169, 1047, 974; **$[\alpha]^{21}_D$** = -8.5 (*c* = 2 in $CHCl_3$);

HRMS (ESI, m/z): calculated for $[C_{20}H_{26}NO_6]^+$: 376.1755, found: 376.1772;
HPLC analysis: >30:1 dr, [CHIRALPAK OD-H column; 0.7 mL/min; solvent system: *i*-PrOH/hexane 20:80; retention times: 10.9 min (minor), 13.1 min (major)].

Supplementary Note 40: **Compound 41:**

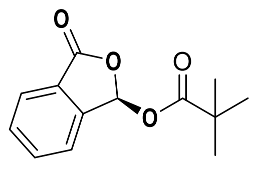

Colorless semisolid, 66% yield
^{1}H NMR (400 MHz, $CDCl_3$) δ 7.94 (dd, J = 7.6, 1.0 Hz, 1H), 7.75 (tt, J = 7.5, 1.0 Hz, 1H), 7.66 (t, J = 7.5 Hz, 1H), 7.56 (d, J = 7.6 Hz, 1H), 7.43 (s, 1H), 1.25 (s, 9H);
^{13}C NMR (100 MHz, $CDCl_3$) δ 176.99, 167.96, 144.63, 134.76, 131.13, 126.59, 125.77, 123.32, 92.82, 38.95, 26.82;
IR ν_{max} (film, cm^{-1}): 2089, 1782, 1636, 1274, 1215, 1120, 974; **$[\alpha]^{21}_D$** = -48.7 (c = 1.2 in $CHCl_3$);
HRMS (ESI, m/z): calculated for $[C_{13}H_{14}O_4]^+$: 235.0970, found: 235.0967;
HPLC analysis: 97:3 er, [CHIRALPAK IA column; 0.6 mL/min; solvent system: *i*-PrOH/hexane 5:95; retention times: 10.8 min (minor), 12.2 min (major)].

Supplementary Note 41: **Compound 42:**

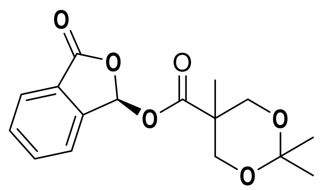

Yellow solid, 75% yield
^{1}H NMR (400 MHz, $CDCl_3$) δ 7.94 (d, J = 7.6, 1H), 7.76 (t, J = 7.5, 1H), 7.67 (t, J = 7.5, 1H), 7.61 (d, J = 7.6 Hz, 1H), 7.50 (s, 1H), 4.24 (dd, J = 11.7, 1.7 Hz, 1H), 4.16 (dd, J = 11.8, 1.8 Hz, 1H), 3.67 (dd, J = 11.8, 2.8 Hz, 2H), 1.43 (s, 3H), 1.39 (s, 3H), 1.20 (s, 3H);
^{13}C NMR (100 MHz, $CDCl_3$) δ 172.93, 167.76, 144.27, 134.88, 131.26, 126.39, 125.74, 123.50, 98.20, 92.93, 65.79, 65.65, 42.29, 25.60, 21.52, 18.05;
IR ν_{max} (film, cm^{-1}): 2085, 1790, 1751, 1636, 1213, 1080, 976; **$[\alpha]^{21}_D$** = -29.7 (c = 1 in $CHCl_3$);
HRMS (ESI, m/z): calculated for $[C_{16}H_{19}O_6]^+$: 307.1182, found: 307.1180;
HPLC analysis: 94:6 er, [CHIRALPAK IB column; 0.6 mL/min; solvent system: *i*-PrOH/hexane 5:95; retention times: 16.8 min (minor), 18.2 min (major)].

Supplementary Note 42: **Compound 43:**

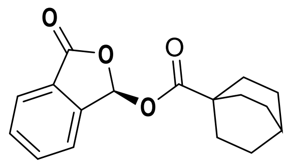

Yellow solid, 82% yield
^{1}H NMR (400 MHz, $CDCl_3$) δ 7.94 (d, J = 7.6 Hz, 1H), 7.75 (td, J = 7.5, 1.1 Hz, 1H), 7.65 (t, J = 7.3 Hz, 1H), 7.56 (d, J = 7.6 Hz, 1H), 7.44 (s, 1H), 2.02 (m, 3H), 1.93 (m, 6H), 1.78 – 1.65 (m, 6H);
^{13}C NMR (100 MHz, $CDCl_3$) δ 176.01, 167.99, 144.70, 134.73, 131.08, 126.56, 125.72, 123.36, 92.67, 40.89, 38.39, 36.26, 27.68;
IR ν_{max} (film, cm^{-1}): 2934, 2852, 2091, 1788, 1636, 1121, 970; **$[\alpha]^{21}_D$** = -46.7 (c = 2.8 in $CHCl_3$);
HRMS (ESI, m/z): calculated for $[C_{17}H_{19}O_4]^+$: 287.1283, found: 287.1275;
HPLC analysis: 97:3 er, [CHIRALPAK IA column; 0.6 mL/min; solvent system: *i*-PrOH/hexane 5:95; retention times: 13.1 min (minor), 14.3 min (major)].

Supplementary Note 43: **Compound 44:**

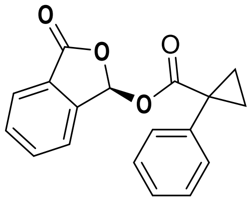

Yellow solid, 70% yield

^{1}H NMR (400 MHz, $CDCl_3$) δ 7.88 (d, J = 7.6 Hz, 1H), 7.69 (td, J = 7.5, 1.0 Hz, 1H), 7.60 (t, J = 7.5 Hz, 1H), 7.50 (d, J = 7.6 Hz, 1H), 7.39 (s, 1H), 7.37 – 7.31 (m, 2H), 7.31 – 7.19 (m, 3H), 1.77 – 1.64 (m, 2H), 1.37 – 1.26 (m, 2H);

^{13}C NMR (100 MHz, $CDCl_3$) δ 173.20, 167.82, 144.28, 138.10, 134.72, 131.09, 130.38, 128.23, 127.49, 126.44, 125.65, 123.35, 93.08, 29.00, 17.39, 17.16;

IR ν_{max} (film, cm^{-1}): 2093, 1996, 1782, 1638, 1275, 1146, 974; **$[\alpha]^{21}_{D}$** = -1.3 (c = 1 in $CHCl_3$);

HRMS (ESI, m/z): calculated for $[C_{18}H_{15}O_4]^+$: 295.0965, found: 295.0963;

HPLC analysis: 91:9 er, [CHIRALPAK OD-H column; 0.7 mL/min; solvent system: *i*-PrOH/hexane 30:70; retention times: 10.8 min (minor), 15.6 min (major)].

Supplementary Note 44: **Compound 45:**

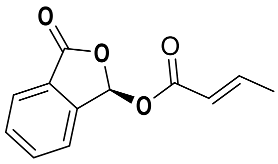

Yellow solid, 91% yield

^{1}H NMR (400 MHz, $CDCl_3$) δ 7.94 (d, J = 7.6 Hz, 1H), 7.75 (td, J = 7.5, 1.1 Hz, 1H), 7.70 – 7.56 (m, 2H), 7.51 (s, 1H), 7.14 (dq, J = 15.7, 6.9 Hz, 1H), 5.90 (dq, J = 15.7, 1.8 Hz, 1H), 1.93 (dd, J = 6.9, 1.8 Hz, 3H);

^{13}C NMR (100 MHz, $CDCl_3$) δ 167.91, 164.52, 148.38, 144.52, 134.72, 131.15, 126.55, 125.73, 123.57, 121.06, 92.71, 18.26;

IR ν_{max} (film, cm^{-1}): 2980, 2922, 1784, 1732, 1651, 1284, 972; **$[\alpha]^{21}_{D}$** = -64.0 (c = 3.0 in $CHCl_3$);

HRMS (ESI, m/z): calculated for $[C_{12}H_{11}O_4]^+$: 219.0657, found: 219.0654;

HPLC analysis: 92:8 er, [CHIRALPAK IB column; 0.6 mL/min; solvent system: *i*-PrOH/hexane 5:95; retention times: 15.4 min (minor), 16.8 min (major)].

Supplementary Note 45: **Compound 46:**

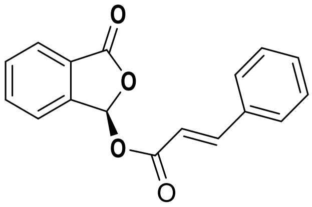

Yellow solid, 65% yield

^{1}H NMR (400 MHz, $CDCl_3$) δ 7.96 (d, J = 7.5 Hz, 1H), 7.85 – 7.73 (m, 2H), 7.67 (t, J = 7.9 Hz, 2H), 7.59 (s, 1H), 7.56 – 7.48 (m, 2H), 7.46 – 7.35 (m, 3H), 6.46 (d, J = 16.0 Hz, 1H);

^{13}C NMR (100 MHz, $CDCl_3$) δ 167.90, 165.16, 147.67, 144.47, 134.80, 133.76, 131.23, 131.01, 128.99, 128.36, 126.56, 125.77, 123.66, 115.99, 92.91;

IR ν_{max} (film, cm^{-1}): 2093, 1782, 1636, 1307, 1142, 1051, 968; **$[\alpha]^{21}_{D}$** = -27.7 (c = 1 in $CHCl_3$);

HRMS (ESI, m/z): calculated for $[C_{17}H_{13}O_4]^+$: 281.0808, found: 281.0812;

HPLC analysis: 97:3 er, [CHIRALPAK OD-H column; 0.7 mL/min; solvent system: *i*-PrOH/hexane 30:70; retention times: 17.1 min (major), 25.3 min (minor)].

Supplementary Note 46: **Compound 47:**

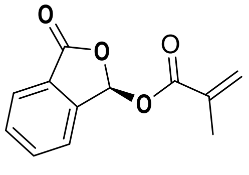

Light yellow solid, 75% yield

^{1}H NMR (400 MHz, $CDCl_3$) δ 7.95 (d, J = 7.6 Hz, 1H), 7.76 (t, J = 7.5 Hz, 1H), 7.71 – 7.60 (m, 2H), 7.50 (s, 1H), 6.19 (s, 1H), 5.72 (d, J = 1.3 Hz, 1H), 1.99 (s, 3H);

^{13}C NMR (100 MHz, $CDCl_3$) δ 167.87, 165.63, 144.42, 134.88, 134.80, 131.22, 128.24, 126.54, 125.76, 123.60, 93.08, 18.06;

IR ν$_{max}$ (film, cm^{-1}): 2090, 1784, 1636, 1307, 1283, 1132, 974; **[α]$^{21}_{D}$** = -38.1 (*c* = 1 in $CHCl_3$);
HRMS (ESI, m/z): calculated for $[C_{12}H_{11}O_4]^+$: 219.0652, found: 219.0650;
HPLC analysis: 95:5 er, [CHIRALPAK OD-H column; 0.7 mL/min; solvent system: *i*-PrOH/hexane 20:80; retention times: 7.8 min (minor), 8.5 min (major)].

Supplementary Note 47: **Compound 48:**

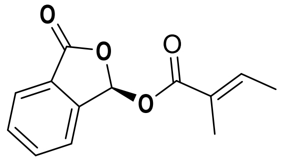

Off-white solid, 78% yield
^{1}H NMR (400 MHz, $CDCl_3$) δ 7.94 (d, *J* = 7.6 Hz, 1H), 7.75 (t, *J* = 7.5 Hz, 1H), 7.66 (t, *J* = 7.5 Hz, 1H), 7.61 (d, *J* = 7.6 Hz, 1H), 7.52 (s, 1H), 7.01 – 6.93 (m, 1H), 1.88 (s, 3H), 1.82 (d, *J* = 6.6 Hz, 3H);
^{13}C NMR (100 MHz, $CDCl_3$) δ 167.96, 166.13, 144.70, 140.70, 134.70, 131.09, 127.32, 126.61, 125.70, 123.59, 93.06, 14.59, 11.92;
IR ν$_{max}$ (film, cm^{-1}): 2938, 2087, 1771, 1645, 1260, 976; **[α]$^{21}_{D}$** = -53.8 (*c* = 0.6 in $CHCl_3$);
HRMS (ESI, m/z): calculated for $[C_{13}H_{13}O_4]^+$: 233.0814, found: 233.0819;
HPLC analysis: 97:3 er, [CHIRALPAK IB column; 0.6 mL/min; solvent system: *i*-PrOH/hexane 5:95; retention times: 17.1 min (minor), 18.5 min (major)].

Supplementary Note 48: **Compound 49:**

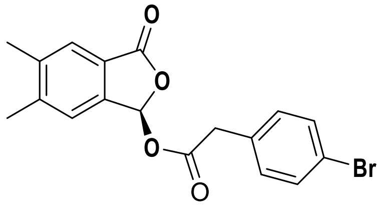

Yellow solid, 82% yield
^{1}H NMR (400 MHz, $CDCl_3$) δ 7.67 (s, 1H), 7.49 – 7.45 (m, 2H), 7.34 (s, 1H), 7.27 (s, 1H), 7.18 – 7.15 (m, 2H), 3.68 (s, 2H), 2.39 (s, 3H), 2.38 (s, 3H);
^{13}C NMR (100 MHz, $CDCl_3$) δ 169.75, 168.00, 145.22, 142.18, 140.81, 131.84, 131.58, 131.04, 126.14, 124.16, 124.10, 121.61, 92.92, 40.29, 20.77, 20.14;
IR ν$_{max}$ (film, cm^{-1}): 2092, 1772, 1643, 1261, 1217, 968; **[α]$^{21}_{D}$** = -12.0 (*c* = 0.8 in $CHCl_3$);
HRMS (ESI, m/z): calcd. for $[C_{18}H_{16}BrO_4]^+$ 375.0226, found 375.0221;
HPLC analysis: 97:3 er, [CHIRALPAK ADH column; 0.6 mL/min; solvent system: *i*-PrOH/hexane 15:85; retention times: 18.3 min (minor), 34.6 min (major)]

Supplementary Note 49: **Compound 50:**

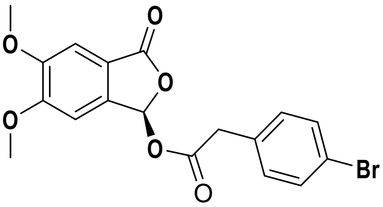

Yellow solid, 79% yield
^{1}H NMR (400 MHz, $CDCl_3$) δ 7.48 – 7.46 (m, 2H), 7.32 (s, 1H), 7.29 (s, 1H), 7.21 – 7.15 (m, 2H), 6.87 (s, 1H), 3.96 (s, 3H), 3.95 (s, 3H), 3.69 (s, 1H);
^{13}C NMR (100 MHz, $CDCl_3$) δ 169.85, 167.93, 155.27, 152.08, 138.43, 131.85, 131.60, 131.04, 121.64, 118.52, 106.16, 104.66, 92.73, 56.55, 56.43, 40.33;
IR ν$_{max}$ (film, cm^{-1}): 1789.9, 1737.9, 1643.4, 1259.5, 1089.8, 976.0; **[α]$^{21}_{D}$** = -6.0 (*c* = 0.7 in $CHCl_3$);
HRMS (ESI, m/z): calcd. for $[C_{18}H_{16}BrO_6]^+$ 407.0125, found 407.0127;
HPLC analysis: 96:4 er, [CHIRALPAK IB column; 0.6 mL/min; solvent system: *i*-PrOH/hexane 15:85; retention times: 31.1 min (minor), 42.3 min (major)]

Supplementary Note 50: **Compound 51:**

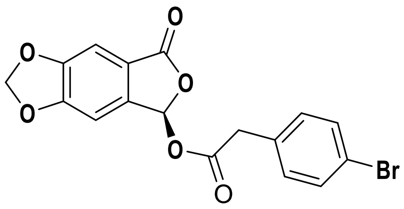

White solid, 78% yield

^{1}H NMR (400 MHz, $CDCl_3$) δ 7.49 – 7.45 (m, 2H), 7.27 (s, 1H), 7.21 (s, 1H), 7.18 – 7.15 (m, 2H), 6.86 (s, 1H), 6.16 (s, 2H), 3.68 (s, 2H);

^{13}C NMR (100 MHz, $CDCl_3$) δ 169.69, 167.10, 154.11, 150.90, 140.47, 131.88, 131.48, 131.00, 121.67, 120.49, 104.54, 103.23, 103.07, 92.44, 40.29;

IR ν_{max} (film, cm^{-1}): 2960.7, 2924.1, 1637.6, 1338.6, 1037.7, 769.6; **$[\alpha]^{21}_{D}$** = -21.8 (c = 0.7 in $CHCl_3$);

HRMS (ESI, m/z): calcd. for $[C_{17}H_{12}BrO_6]^+$ 390.9812, found 390.9815;

HPLC analysis: 98:2 er, [CHIRALPAK IB column; 0.6 mL/min; solvent system: *i*-PrOH/hexane 15:85; retention times: 31.4 min (minor), 42.4 min (major)]

Supplementary Note 51: **Compound 52a:**

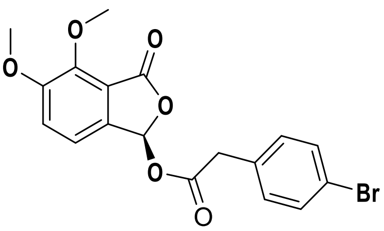

Colorless oil, 59% yield

^{1}H NMR (400 MHz, $CDCl_3$) δ 7.47 (d, J = 8.5 Hz, 2H), 7.29 (s, 1H), 7.24 – 7.10 (m, 4H), 4.13 (s, 3H), 3.93 (s, 3H), 3.67 (s, 2H).;

^{13}C NMR (100 MHz, $CDCl_3$) δ 169.75, 165.23, 154.31, 148.56, 136.39, 131.84, 131.59, 131.01, 121.61, 119.16, 118.09, 117.84, 92.13, 62.49, 56.80, 40.34;

IR ν_{max} (film, cm^{-1}): 2852.7, 2088.9, 1784.2, 1635.6, 14987, 1265, 983.7; **$[\alpha]^{21}_{D}$** = -5.0 (c = 1.0 in $CHCl_3$);

HRMS (ESI, m/z): calcd. for $[C_{18}H_{16}BrO_6]^+$ 407.0125, found 407.0123;

HPLC analysis: 95:5 er, [CHIRALPAK IB column; 0.6 mL/min; solvent system: *i*-PrOH/hexane 15:85; retention times: 27.9 min (minor), 38.8 min (major)]

CCDC codes: CCDC 1893685, the crystallographic data be obtained free of charge from The Cambridge Crystallographic Data Centre via www.ccdc.cam.ac.uk/data_request/cif. (note: recrystallized in ethyl acetate / ethanol but in racemic form)

Supplementary Note 52: **Compound 52b:**

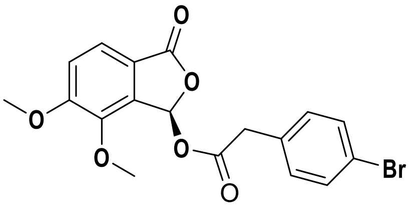

Colorless oil, 27% yield

^{1}H NMR (400 MHz, $CDCl_3$) δ 7.63 (d, J = 8.2 Hz, 1H), 7.50 – 7.44 (m, 3H), 7.16 (dd, J = 12.4, 8.1 Hz, 3H), 3.97 (s, 3H), 3.75 (s, 3H), 3.69 (s, 2H);

^{13}C NMR (100 MHz, $CDCl_3$) δ 169.18, 167.28, 157.51, 144.26, 135.44, 131.85, 131.64, 131.1, 121.95, 121.58, 119.2, 115.50, 60.67, 56.54, 40.42;

IR ν_{max} (film, cm^{-1}): 2856.6, 2088.9, 1772.6, 1645.3, 1635.6, 1278.8; **$[\alpha]^{21}_{D}$** = -75.6 (c = 0.5 in $CHCl_3$);

HRMS (ESI, m/z): calcd. for $[C_{18}H_{16}BrO_6]^+$ 407.0125, found 407.0121;

HPLC analysis: 95:5 er, [CHIRALPAK IB column; 0.6 mL/min; solvent system: *i*-PrOH/hexane 15:85; retention times: 24.9 min (minor), 25.6 min (major)]

Supplementary Note 53: **Compound 53:**

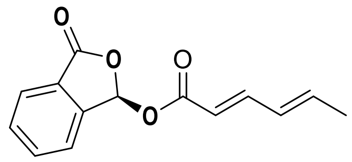

Yellow solid, 75% yield

^{1}H NMR (400 MHz, $CDCl_3$) δ 7.94 (d, J = 7.6 Hz, 1H), 7.75 (td, J = 7.5, 1.0 Hz, 1H), 7.69 – 7.59 (m, 2H), 7.52 (s, 1H), 7.42 – 7.32 (m, 1H), 6.25 – 6.19 (m, 2H), 5.80 (d, J = 14.9 Hz, 1H), 1.88 (d, J = 4.9 Hz, 3H);

^{13}C NMR (100 MHz, $CDCl_3$) δ 167.92, 165.37, 147.95, 144.57, 141.72, 134.71, 131.11, 129.54, 126.55, 125.67, 123.61, 116.69, 92.74, 18.73;

IR ν_{max} (film, cm^{-1}): 2093, 1778, 1771, 1643, 1236, 1124, 970; **$[\alpha]^{21}_D$** = - 29.0 (c = 1 in $CHCl_3$);

HRMS (ESI, m/z): calculated for $[C_{14}H_{13}O_4]^+$: 245.0808, found: 245.0817;

HPLC analysis: 95:5 er, [CHIRALPAK OD-H column; 0.7 mL/min; solvent system: i-PrOH/hexane 20:80; retention times: 11.3 min (minor), 12.9 min (major)].

Supplementary Note 54: **Compound 54:**

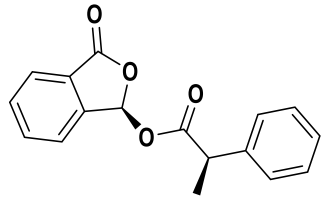

Colorless glassy solid, 76% yield

^{1}H NMR (400 MHz, $CDCl_3$) δ 7.90 (d, J = 7.2 Hz, 1H), 7.69 – 7.57 (m, 2H), 7.38 (s, 1H), 7.36 – 7.24 (m, 6H), 3.79 (q, J = 7.1 Hz, 1H), 1.57 (d, J = 7.2 Hz, 3H);

^{13}C NMR (100 MHz, $CDCl_3$) δ 173.09, 167.81, 144.20, 139.34, 134.71, 131.14, 128.75, 127.49, 127.44, 126.38, 125.67, 123.29, 92.81, 45.23, 18.12;

IR ν_{max} (film, cm^{-1}): 2093, 1788, 1759, 1643, 1358, 1284, 1140, 974; **$[\alpha]^{21}_D$** = - 0.22 (c = 1 in $CHCl_3$);

HRMS (ESI, m/z): calculated for $[C_{17}H_{15}O_4]^+$: 283.0965, found: 283.0964;

Supplementary Note 55: **Compound 55:**

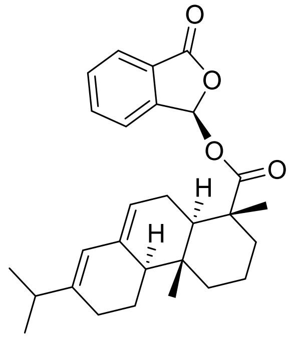

Off-white solid, 64 % yield

^{1}H NMR (400 MHz, $CDCl_3$) δ 7.93 (d, J = 7.6 Hz, 1H), 7.74 (td, J = 7.5, 1.0 Hz, 1H), 7.65 (t, J = 7.3 Hz, 1H), 7.54 (d, J = 7.6 Hz, 1H), 7.41 (s, 1H), 5.76 (s, 1H), 5.38– 5.33 (m, 1H), 2.22 (dq, J = 13.6, 6.8 Hz, 1H), 2.11 – 1.97 (m, 4H), 1.94 – 1.66 (m, 6H), 1.63 – 1.55 (m, 2H), 1.30 (s, 3H), 1.25 – 1.09 (m, 2H), 1.00 (dd, J = 6.8, 3.9 Hz, 6H), 0.82 (s, 3H);

^{13}C NMR (100 MHz, $CDCl_3$) δ 177.02, 167.93, 145.34, 144.62, 135.35, 134.73, 131.11, 126.65, 125.77, 123.35, 122.33, 120.30, 93.01, 50.68, 46.83, 44.72, 38.04, 36.82, 34.84, 34.48, 27.36, 25.49, 22.40, 21.35, 20.78, 17.93, 16.87, 14.01;

IR ν_{max} (film, cm^{-1}): 2987, 2087, 1784, 1697, 1643, 1284, 974; **$[\alpha]^{21}_D$** = -34.9 (c = 1 in $CHCl_3$);

HRMS (ESI, m/z): calculated for $[C_{28}H_{35}O_4]^+$: 435.2530, found: 435.2527.;

Supplementary Note 56: **Compound 56:**

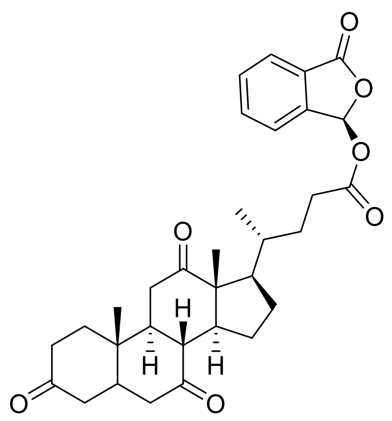

Off-white solid, 72 % yield

^{1}H NMR (400 MHz, $CDCl_3$) δ 7.93 (d, J = 7.6 Hz, 1H), 7.76 (td, J = 7.5, 0.9 Hz, 1H), 7.66 (t, J = 7.4 Hz, 1H), 7.60 (d, J = 7.6 Hz, 1H), 7.45 (s, 1H), 2.98 – 2.80 (m, 3H), 2.58 – 2.47 (m, 1H), 2.45 – 2.08 (m, 9H), 2.08 – 1.78 (m, 6H), 1.62 (td, J = 14.3, 4.8 Hz, 1H), 1.51 – 1.40 (m, 4H), 1.38 – 1.20 (m, 3H), 1.08 (s, 3H), 0.84 (d, J = 6.6 Hz, 3H);

^{13}C NMR (100 MHz, $CDCl_3$) δ 211.85, 208.98, 208.64, 172.47, 167.81, 144.28, 134.73, 131.16, 126.40, 125.69, 123.44, 92.51, 56.79, 51.70, 48.87, 46.72, 45.50, 45.46, 44.89, 42.69, 38.53, 36.38, 35.92, 35.28, 35.15, 31.14, 29.98, 27.57, 25.02, 21.81, 18.49, 11.74;

IR ν_{max} (film, cm^{-1}): 2091, 1782, 1643, 1530, 1358, 1215, 971; **$[\alpha]^{21}_D$** = 9.3 (c = 2 in $CHCl_3$);

HRMS (ESI, m/z): calculated for $[C_{32}H_{39}O_7]^+$: 535.2690, found: 535.2696;

Supplementary Note 57: **Compound *R*-57**

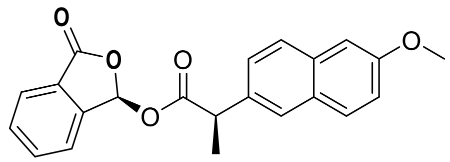

White solid, 87% yield

^{1}H NMR (400 MHz, $CDCl_3$) δ 7.92 – 7.85 (m, 1H), 7.74 – 7.65 (m, 3H), 7.62 – 7.52 (m, 2H), 7.42 – 7.36 (m, 2H), 7.28 – 7.22 (m, 1H), 7.19 – 7.10 (m, 2H), 3.92 (s, 4H), 1.64 (d, J = 7.1 Hz, 3H);

^{13}C NMR (100 MHz, $CDCl_3$) δ 173.25, 167.81, 157.81, 144.22, 134.69, 134.43, 133.84, 131.11, 129.25, 128.88, 127.34, 126.40, 126.13, 125.93, 125.68, 123.34, 119.16, 105.61, 92.88, 55.31, 45.18, 18.21;

IR ν_{max} (film, cm^{-1}): 2854, 2081, 1636, 1261, 1263, 972, 850; **$[\alpha]^{21}_D$** = -19.7 (c = 1.5 in $CHCl_3$);

HRMS (ESI, m/z): calculated for $[C_{22}H_{19}O_5]^+$: 363.1232, found: 363.1230;

HPLC analysis: > 40:1 dr [CHIRALPAK IB column; 0.6 mL/min; solvent system: *i*-PrOH/hexane 5:95; retention times: 26.3 min (major), 35.1 min (minor)].

Supplementary Note 58: **Compound *S*-57**

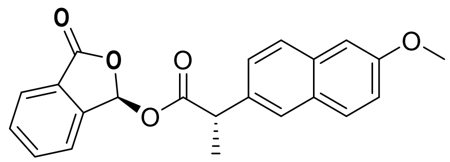

White solid, 82% yield

^{1}H NMR (400 MHz, $CDCl_3$) δ 7.89 (dt, J = 7.4, 1.0 Hz, 1H), 7.73 – 7.59 (m, 5H), 7.51 (dq, J = 7.7, 0.8 Hz, 1H), 7.42 (s, 1H), 7.37 (dd, J = 8.4, 1.9 Hz, 1H), 7.17 – 7.09 (m, 2H), 3.91 (s, 4H), 1.63 (d, J = 7.1 Hz, 3H);

^{13}C NMR (100 MHz, $CDCl_3$) δ 173.17, 167.75, 157.80, 144.29, 134.71, 134.14, 133.85, 131.18, 129.28, 128.89, 127.41, 126.55, 126.16, 125.91, 125.75, 123.45, 119.13, 105.62, 93.03, 55.32, 45.42, 18.44;

IR ν_{max} (film, cm^{-1}): 2854, 2081, 1636, 1261, 1263, 972, 850; **$[\alpha]^{21}_D$** = - 25.9 (c = 1.3 in $CHCl_3$);

HRMS (ESI, m/z): calculated for $[C_{22}H_{19}O_5]^+$: 363.1232, found: 363.1226;

HPLC analysis: >20:1 [CHIRALPAK IB column; 0.6 mL/min; solvent system: *i*-PrOH/hexane 5:95; retention times: 27.3 min (major), 28.9 min (minor)].

Supplementary Note 59: **Compound 58:**

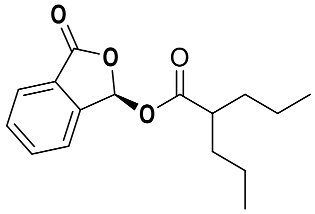

White solid, 79% yield

^{1}H NMR (400 MHz, $CDCl_3$) δ 7.94 (d, J = 7.6 Hz, 1H), 7.75 (td, J = 7.5, 1.1 Hz, 1H), 7.69 – 7.62 (m, 1H), 7.57 – 7.52 (m, 1H), 7.47 (s, 1H), 2.52 – 2.41 (m, 1H), 1.73 – 1.61 (m, 2H), 1.54 – 1.42 (m, 2H), 1.42 – 1.26 (m, 4H), 0.91 (td, J = 7.3, 2.5 Hz, 6H);

^{13}C NMR (100 MHz, $CDCl_3$) δ 175.00, 167.95, 144.60, 134.75, 131.15, 126.60, 125.78, 123.34, 92.54, 45.10, 34.26, 34.21, 20.53, 20.48, 13.92, 13.90;

IR ν_{max} (film, cm^{-1}): 3055, 2986, 2034, 2117, 1788, 1643, 1265, 895; **$[\alpha]^{21}_{D}$** = -1.9 (c = 1 in $CHCl_3$);

HRMS (ESI, m/z): calculated for $[C_{16}H_{21}O_4]^+$: 277.1434, found: 277.1435;

HPLC analysis: 98:2 er, [CHIRALPAK OD-H column; 0.7 mL/min; solvent system: i-PrOH/hexane 20:80; retention times: 9.8 min (minor), 11.9 min (major)].

Supplementary Note 60: **Compound 59**

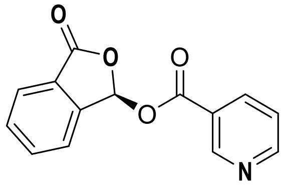

White solid, 62% yield

^{1}H NMR (400 MHz, $CDCl_3$) δ 9.23 (dd, J = 2.2, 0.9 Hz, 1H), 8.83 (dd, J = 4.9, 1.7 Hz, 1H), 8.33 (dt, J = 8.0, 2.0 Hz, 1H), 7.99 (dt, J = 7.7, 1.1 Hz, 1H), 7.80 (td, J = 7.5, 1.2 Hz, 1H), 7.75 – 7.67 (m, 3H), 7.43 (ddd, J = 8.0, 4.9, 0.9 Hz, 1H);

^{13}C NMR (100 MHz, $CDCl_3$) δ 167.59, 163.87, 154.35, 151.24, 143.96, 137.51, 134.98, 131.51, 126.47, 125.95, 124.51, 123.69, 123.45, 93.25;

IR ν_{max} (film, cm^{-1}): 2857, 2093, 1784, 1638, 1261, 1097, 974, 783; **$[\alpha]^{21}_{D}$** = - 31.5 (c = 0.7 in $CHCl_3$);

HRMS (ESI, m/z): calculated for $[C_{14}H_{10}NO_4]^+$: 256.0610, found: 256.0607;

HPLC analysis: 88:12 er, [CHIRALPAK IB column; 0.6 mL/min; solvent system: i-PrOH/hexane 5:95; retention times: 50.8 min (minor), 55.9 min (major)].

Supplementary Note 61: **Compound 60**

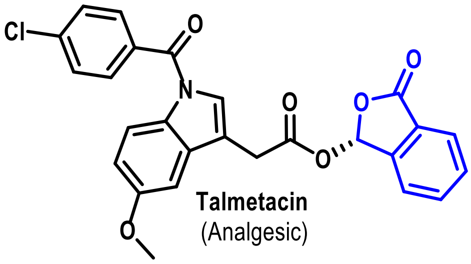

Light yellow solid, 80% yield

^{1}H NMR (400 MHz, $CDCl_3$) δ 7.94 (d, J = 7.7 Hz, 1H), 7.72 (td, J = 7.5, 1.2 Hz, 1H), 7.68 – 7.64 (m, 3H), 7.51 – 7.45 (m, 3H), 7.43 (s, 1H), 6.92 – 6.88 (m, 2H), 6.68 (dd, J = 9.0, 2.5 Hz, 1H), 3.78 (s, 2H), 3.77 (s, 3H), 2.37 (s, 3H);

^{13}C NMR (100 MHz, $CDCl_3$) δ 169.33, 168.25, 167.69, 156.07, 144.11, 139.42, 136.28, 134.82, 133.69, 131.34, 131.18, 130.77, 130.18, 129.15, 126.43, 125.84, 123.50, 115.00, 111.96, 111.18, 101.07, 93.04, 55.66, 30.17, 13.39;

IR ν_{max} (film, cm^{-1}): 2091, 1784, 1637, 1478, 1355, 1215, 974, 750; **$[\alpha]^{21}_{D}$** = - 29.0 (c = 3.2 in $CHCl_3$);

HRMS (ESI, m/z): calculated for $[C_{27}H_{21}ClNO_6]^+$: 490.1057, found: 490.1052;

HPLC analysis: 98:2 er, [CHIRALPAK IB column; 0.6 mL/min; solvent system: i-PrOH/hexane 5:95; retention times: 54.6 min (major), 57.3 min (minor)].

Supplementary Note 62: **Compound 61**

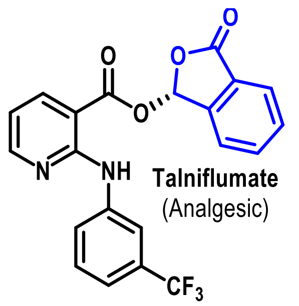

Yellow solid, 96% yield

^{1}H NMR (400 MHz, $CDCl_3$) δ 10.16 (s, 1H), 8.45 (dd, J = 4.6, 2.0 Hz, 1H), 8.19 (dd, J = 7.9,2.0 Hz, 1H), 8.11 (s, 1H), 7.99 (d, J = 7.5 Hz, 1H), 7.87 (d, J = 8.1 Hz, 1H), 7.82 (dt, J = 6.9, 1.0 Hz, 1H), 7.71 (t, J = 7.5 Hz, 2H), 7.66 (s, 1H), 7.46 (t, J = 8.0 Hz, 1H), 7.33 (d, J =7.7 Hz, 1H), 6.76 (dd, J = 7.9, 4.7 Hz, 1H);

^{13}C NMR (100 MHz, $CDCl_3$) δ 167.6, 116.1, 156.1, 154.4, 144.0, 140.8, 139.8, 135.0, 131.6, 129.3, 126.0, 124.0, 123.7, 119.64, 119.60, 117.62, 117.58, 114.2, 105.5, 93.2;

IR ν_{max} (film, cm^{-1}): 2093, 1786, 1630, 1446, 1328, 1126, 970; **$[\alpha]^{21}_D$** = - 44.0 (c = 2.2 in $CHCl_3$);

HRMS (ESI, m/z): calculated for $[C_{21}H_{13}F3NO_4]^+$: 400.0833, found: 400.0821;

HPLC analysis: 95:5 er, [CHIRALPAK IB column; 0.6 mL/min; solvent system: *i*-PrOH/hexane 5:95; retention times: 24.5 min (minor), 25.9 min (major)].

Supplementary Note 63: **Compound 62**

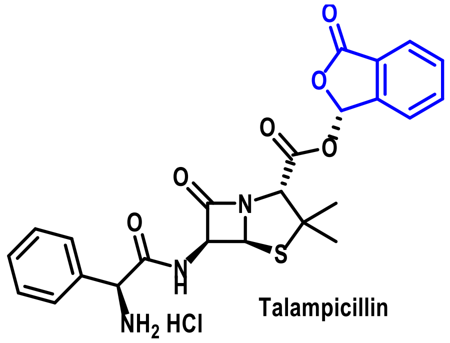

Off-white solid, 48% yield (two steps)

^{1}H NMR (400 MHz, MeOD) δ 7.95 – 7.88 (m, 3H), 7.83 (tdd, J = 7.6, 2.5, 1.3 Hz, 2H), 7.76 – 7.67 (m, 5H), 7.55 – 7.41 (m, 16H), 5.58 (ddd, J = 5.6, 4.0, 1.1 Hz, 2H), 5.46 (ddd, J = 15.9, 4.1, 1.1 Hz, 2H), 5.02 (d, J = 2.3 Hz, 2H), 4.50 (dd, J = 5.1, 1.1 Hz, 2H), 1.51 – 1.38 (m, 11H);**IR ν_{max}** (film, cm^{-1}): 2629, 2085, 1782, 1771, 1643, 1358, 1151, 980. The data was consistent with the reported literature [2-3]. **$[\alpha]^{21}_D$** = 185 (c = 0.5 in MeOH);

HRMS (ESI, m/z): calculated for $[C_{24}H_{24}N_3O_6S]^+$: 482.1386, found: 482.1386;

HPLC analysis: 6:1 dr, drHIRALPAK IB column; 0.6 mL/min; solvent system: *i*-PrOH/hexane 5:95; retention times: 26.7 min (minor), 37.8 min (major)].

Supplementary Note 64: **Compound 63**

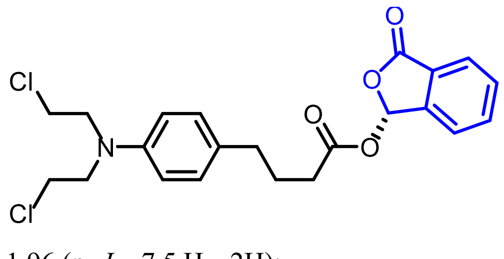

Yellow gum, 87% yield

^{1}H NMR (400 MHz, $CDCl_3$) δ 7.93 (d, J = 7.5 Hz, 1H), 7.74 (t, J = 7.5 Hz, 1H), 7.65 (t, J = 7.5 Hz, 1H), 7.57 (d, J = 7.6 Hz, 1H), 7.44 (s, 1H), 7.05 (d, J = 8.3 Hz, 2H), 6.65 – 6.60 (m, 2H), 3.78 – 3.54 (m, 8H), 2.59 (t, J = 7.5 Hz, 2H), 2.43 (t, J = 7.5 Hz, 2H), 1.96 (p, J = 7.5 Hz, 2H);

^{13}C NMR (100 MHz, $CDCl_3$) δ 172.02, 167.81, 144.42, 144.34, 134.75, 131.21, 130.06, 129.68, 126.51, 125.79, 123.45, 112.23, 92.58, 53.59, 40.48, 33.73, 33.26, 26.27;

IR ν_{max} (film, cm^{-1}): 2085, 1884, 1636, 1355, 1259, 1107, 1012; **$[\alpha]^{21}_D$** = - 16.7 (c = 0.6 in $CHCl_3$);

HRMS (ESI, m/z): calculated for $[C_{21}H_{22}Cl_2NO_4]^+$: 422.0926, found: 422.0921;

HPLC analysis: >99:1 er, [CHIRALPAK IB column; 0.6 mL/min; solvent system: *i*-PrOH/hexane 5:95; retention times: 59.6 min (major), 66.9 min (minor)].

Supplementary Reference

1. Hsieh, S. Y., Binanzer, M., Kreituss, I. & Bode, J. W. Expanded substrate scope and catalyst optimization for the catalytic kinetic resolution of N-heterocycles. *Chem. Commun.* **48**, 8892-8894 (2012).
2. Clayton, J. P. *et al.* Preparation, Hydrolysis, and Oral Absorption of Lactonyl Esters of Penicillins. *J. Med. Chem.* **19**, 1385-1391 (1976).
3. I. Isaka, K. N., T. Kashiwagi, A. Koda, H. Horiguchi, H. Matsui, K. Takahashi & M. Murakami. Lactol Esters of Ampicillin. *Chem. Pharm. Bull.* **24**, 102-107 (1976).
