# Supplementary material for: Catalytic asymmetric acetalization of carboxylic acids for access to chiral phthalidyl ester prodrugs
Source: Nat Commun. 2019 Apr 11;10:1675. doi: 10.1038/s41467-019-09445-x (PMC6459872; doi:10.1038/s41467-019-09445-x)
Supplement: Supplementary file 2 — Reporting Summary [file 41467_2019_9445_MOESM2_ESM.pdf]

nature research

Corresponding author(s): NCOMMS-18-36553A

Last updated by author(s): Feb 27, 2019

Reporting Summary

Nature Research wishes to improve the reproducibility of the work that we publish. This form provides structure for consistency and transparency in reporting. For further information on Nature Research policies, see Authors & Referees and the Editorial Policy Checklist.

Statistics

For all statistical analyses, confirm that the following items are present in the figure legend, table legend, main text, or Methods section.

n/a	Confirmed	
☐	☒	The exact sample size (*n*) for each experimental group/condition, given as a discrete number and unit of measurement
☐	☒	A statement on whether measurements were taken from distinct samples or whether the same sample was measured repeatedly
☒	☐	The statistical test(s) used AND whether they are one- or two-sided<br>*Only common tests should be described solely by name; describe more complex techniques in the Methods section.*
☐	☒	A description of all covariates tested
☒	☐	A description of any assumptions or corrections, such as tests of normality and adjustment for multiple comparisons
☐	☒	A full description of the statistical parameters including central tendency (e.g. means) or other basic estimates (e.g. regression coefficient) AND variation (e.g. standard deviation) or associated estimates of uncertainty (e.g. confidence intervals)
☒	☐	For null hypothesis testing, the test statistic (e.g. *F*, *t*, *r*) with confidence intervals, effect sizes, degrees of freedom and *P* value noted<br>*Give P values as exact values whenever suitable.*
☒	☐	For Bayesian analysis, information on the choice of priors and Markov chain Monte Carlo settings
☒	☐	For hierarchical and complex designs, identification of the appropriate level for tests and full reporting of outcomes
☒	☐	Estimates of effect sizes (e.g. Cohen's *d*, Pearson's *r*), indicating how they were calculated

Our web collection on statistics for biologists contains articles on many of the points above.

Software and code

Policy information about availability of computer code

Data collection	The data of MTT test was collected by Tecan Infinite M200
Data analysis	The data of MTT test was processed by origin8.0 (originlab) and IBM SPSS Statistics 17.0

For manuscripts utilizing custom algorithms or software that are central to the research but not yet described in published literature, software must be made available to editors/reviewers. We strongly encourage code deposition in a community repository (e.g. GitHub). See the Nature Research guidelines for submitting code & software for further information.

Data

Policy information about availability of data

All manuscripts must include a data availability statement. This statement should provide the following information, where applicable:

- Accession codes, unique identifiers, or web links for publicly available datasets
- A list of figures that have associated raw data
- A description of any restrictions on data availability

The X-ray crystallographic coordinates for NHC C, compound 20, 21 and 52a reported in this study have been deposited at the Cambridge Crystallographic Data Centre (CCDC), under CCDC 1866589, 1866428, 1866429 and 1893685. These data can be obtained free of charge from The Cambridge Crystallographic Data Centre via www.ccdc.cam.ac.uk/data_request/cif Full experimental details for new compounds, and their spectroscopic, chromatographic data and bio-evaluation data, can be found in the supplementary information.

Field-specific reporting

Please select the one below that is the best fit for your research. If you are not sure, read the appropriate sections before making your selection.

☒ Life sciences ☐ Behavioural & social sciences ☐ Ecological, evolutionary & environmental sciences

For a reference copy of the document with all sections, see nature.com/documents/nr-reporting-summary-flat.pdf

Life sciences study design

All studies must disclose on these points even when the disclosure is negative.

Sample size	The HeLa cells (obtained from ATCC, Rockville, MD, maintained in Dulbecco's Modified Eagle's Mediem) were seeded in 96-well plates (100 μL of medium) and incubated for 24 h. After the cell density reached 60%-70%, the cells were fed with the prodrugs and chlorambucil.
Data exclusions	No data was excluded
Replication	n = 5
Randomization	After the cell density reached 60%-70%, the cells were fed with the prodrugs and chlorambucil.
Blinding	The samples were sent to our collaborator under the unidentifiable sample codes. after they finished the experiments, reported the data to us and we disclosed the results.

Reporting for specific materials, systems and methods

We require information from authors about some types of materials, experimental systems and methods used in many studies. Here, indicate whether each material, system or method listed is relevant to your study. If you are not sure if a list item applies to your research, read the appropriate section before selecting a response.

Materials & experimental systems

n/a	Involved in the study	
☒	☐	Antibodies
☐	☒	Eukaryotic cell lines
☒	☐	Palaeontology
☒	☐	Animals and other organisms
☒	☐	Human research participants
☒	☐	Clinical data

Methods

n/a	Involved in the study	
☒	☐	ChIP-seq
☒	☐	Flow cytometry
☒	☐	MRI-based neuroimaging

Eukaryotic cell lines

Policy information about cell lines

Cell line source(s)	HeLa cells were btained from ATCC, Rockville, MD, maintained in Dulbecco's Modified Eagle's Medium)
Authentication	none of the cell lines was authenticated.
Mycoplasma contamination	The HeLa cells were test negative.
Commonly misidentified lines (See ICLAC register)	No other line was used in our case
